# A comprehensive comparison of multilocus association methods with summary statistics in genome-wide association studies

**DOI:** 10.1186/s12859-022-04897-3

**Published:** 2022-08-30

**Authors:** Zhonghe Shao, Ting Wang, Jiahao Qiao, Yuchen Zhang, Shuiping Huang, Ping Zeng

**Affiliations:** 1 Department of Biostatistics, School of Public Health, Xuzhou Medical University, Xuzhou, 221004 Jiangsu China; 2 Center for Medical Statistics and Data Analysis, Xuzhou Medical University, Xuzhou, 221004 Jiangsu China; 3 Key Laboratory of Human Genetics and Environmental Medicine, Xuzhou Medical University, Xuzhou, 221004 Jiangsu China; 4 Key Laboratory of Environment and Health, Xuzhou Medical University, Xuzhou, 221004 Jiangsu China; 5 Engineering Research Innovation Center of Biological Data Mining and Healthcare Transformation, Xuzhou Medical University, Xuzhou, 221004 Jiangsu China

**Keywords:** Genome-wide association study, Multilocus method, SNP-set analysis, Summary statistics, *P* value combination method, Common and rare variant association study, Integrative analysis, Expression quantitative trait loci

## Abstract

**Background:**

Multilocus analysis on a set of single nucleotide polymorphisms (SNPs) pre-assigned within a gene constitutes a valuable complement to single-marker analysis by aggregating data on complex traits in a biologically meaningful way. However, despite the existence of a wide variety of SNP-set methods, few comprehensive comparison studies have been previously performed to evaluate the effectiveness of these methods.

**Results:**

We herein sought to fill this knowledge gap by conducting a comprehensive empirical comparison for 22 commonly-used summary-statistics based SNP-set methods. We showed that only seven methods could effectively control the type I error, and that these well-calibrated approaches had varying power performance under the simulation scenarios. Overall, we confirmed that the burden test was generally underpowered and score-based variance component tests (e.g., sequence kernel association test) were much powerful under the polygenic genetic architecture in both common and rare variant association analyses. We further revealed that two linkage-disequilibrium-free *P* value combination methods (e.g., harmonic mean *P* value method and aggregated Cauchy association test) behaved very well under the sparse genetic architecture in simulations and real-data applications to common and rare variant association analyses as well as in expression quantitative trait loci weighted integrative analysis. We also assessed the scalability of these approaches by recording computational time and found that all these methods can be scalable to biobank-scale data although some might be relatively slow.

**Conclusion:**

In conclusion, we hope that our findings can offer an important guidance on how to choose appropriate multilocus association analysis methods in post-GWAS era. All the SNP-set methods are implemented in the R package called MCA, which is freely available at https://github.com/biostatpzeng/.

**Supplementary Information:**

The online version contains supplementary material available at 10.1186/s12859-022-04897-3.

## Background

Over the past two decades, genome-wide association studies (GWASs) have successfully identified a large number of genetic loci associated with many complex traits/diseases by examining millions of single nucleotide polymorphisms (SNPs) across the whole genome [1–4]. However, the contribution of associated SNPs to disease susceptibility and phenotypic variation is far less than expected, leading to the so-called problem of “missing heritability” [5–8]. One plausible interpretation for such an issue is that the single-marker analysis commonly used in GWAS is underpowered [9]; many potential genetic variants that exhibit significant but weak effects on traits/diseases have yet been discovered. As an effective supplementary strategy of single-marker analysis, multilocus methods have been increasingly applied [10]. Multilocus analysis often jointly examines a set of SNPs that are pre-defined within a functional unit such as gene to evaluate the overall association evidence at the gene level; it is thus also referred to as SNP-set or gene-based approach.

Compared to the conventional single-marker analysis, SNP-set analysis has several statistical and biological advantages. First, susceptibility genes may contain multiple independent pathogenic variants; SNP-set inference can hence substantially increase power by gathering different signals within the gene. The potential of improving power also results from the reduced burden of multiple comparisons. Second, SNP-set analysis can solve the problem of allelic heterogeneity [11], producing more consistent results across distinct studies [12]. Third, many biological processes are driven by complicated mechanisms involving more than one genetic variant; gene (or SNP-set) based inference can thus offer more biologically meaningful interpretation as genes are important functional units in living organisms [13]. Fourth, SNP-set analysis can be easily extended to pathway or network analysis [14–20]. Fifth, SNP-set analysis has already become the standard operation for rare variant association in whole genome sequencing studies [21–27]. Sixth, SNP-set analysis can easily take functional information into account [21, 28–33], which hence improves power and facilitates interpretation of GWAS discoveries. Finally, besides its own importance, SNP-set analysis is a critical step toward many other post-GWAS functional explorations, including gene-centric pleiotropy identification [34, 35], TWAS with bulk-cell sequencing RNA data [36, 37] and integrative gene analysis of GWAS with single-cell RNA sequencing data [38, 39].

Due to the usefulness, distinct SNP-set methods have been recently developed [17, 21, 25, 29, 40–51], many of which can be implemented with only GWAS summary statistics [17, 45, 52–54], greatly generalizing their applicability due to the widespread availability of summary-level data [55]. With distinct SNP-set approaches for multilocus association studies, one naturally wonders which one should be chosen in practice. Moreover, existing SNP-set methods are not used without deficiencies, potential limitations include insufficient power [56], inability to provide statistically valid tests under certain parameter settings [57], and reliance on permutation sampling [58]. Unfortunately, despite the importance of multilocus analysis in GWAS and the vast number of SNP-set methods, few comprehensive comparison studies have been performed to evaluate their effectiveness. Subsequently, due to the lack of consensus on the most suitable SNP-set method, the realization of the above advantages and benefits is to some extent currently hindered.

In the present work, we sought to fill this knowledge gap by conducting a comprehensive comparison for 22 commonly-used summary-statistics based SNP-set methods in the hope that our results could serve as an important guidance for practitioners on how to choose appropriate SNP-set analysis methods in post-GWAS era. In the following, we first evaluated the performance of these various methods in type I error control. We further assessed the power of these SNP-set methods which could maintain well-calibrated control of type I error under various simulation scenarios including common variant association analysis, rare variant association analysis and expression quantitative trait loci (eQTL) weighted integrative association analysis. We also assessed the scalability of these SNP-set approaches by recording computational time in simulation studies. Finally, corresponding to the three main simulation scenarios above, we applied these well-calibrated SNP-set methods to common variant summary statistics of six psychiatric disorders available from the Psychiatric Genomics consortium (PGC) [59, 60], rare variant summary statistics of four plasma lipid traits yielded from the Global Lipids Genetics consortium (GLGC) [61], and two-stage transcriptome-wide association study (TWAS) [31–33, 62–64] by integrating eQTL weights obtained from the Geuvadis project [65] and common variant summary statistics of nine immune-related diseases [63].

## Materials and methods

### Overview of SNP-set analysis methods

As a flexible and powerful strategy alternative to single-marker analysis in association studies, many SNP-set methods have been developed over the past few years [17, 21, 40–45, 51, 66–74], where a group of pre-assigned genetic variants are analyzed collectively to examine their joint influence on diseases/traits. We here have retrieved and compiled a list of 22 widely-used SNP-set methods (Table 1), which can be grouped into distinct categories in terms of input, requirement of external linkage disequilibrium (LD) and computational manner for *P* value of the aggregated test statistic. Particularly, these approaches include LD-dependent linear or quadratic combination of *Z*-scores with an additional correlation matrix accounting for dependence among SNPs (e.g., SKAT and optimal SKAT (SKATO)) [17, 45, 51], and LD-free *P* value combination methods which might be robust against correlation between SNPs (e.g., HMP and aggregated Cauchy association test (ACAT)) [54, 75].

**Table 1:** An overview of 22 SNP-set methods and their corresponding modeling characteristics

No	Year	Method	Input					Calculate *P* value		References
			*P*	*Z*	*W*	R	Other	Analytical	Simulation	
1	1960	MLR		√	√	√	*N*	√		[76]
2	2008	FLM		√	√	√	*N*	√		[45, 77]
3	2004	HC		√	√			√		[66]
4	2017	GHC		√	√	√		√		[49]
5	2019	BJ		√	√			√		[51]
6	2019	GBJ		√	√	√		√		[51]
7	2020	DOT		√	√	√		√		[67]
8	2017	BT		√	√	√		√		[45]
9	2013	SKATO		√	√	√		√		[45]
10	2018	SKAT		√	√	√		√		[45]
11	1986	Simes	√					√		[68]
12	1992	FCP	√					√		[69]
13	2002	TPM	√				*τ*	√		[70]
14	2003	RTP	√				*k*	√		[71]
15	2007	minP	√			√		√		[72]
16	2019	ART	√				*k*	√		[78]
17	2019	ART-A	√		√		*k*	√	√	[78]
18	2007	GM	√				*a*	√		[73]
19	2008	SimpleM	√			√		√		[74]
20	2011	GATES	√			√			√	[79]
21	2019	HMP	√		√			√		[75]
22	2020	ACAT	√		√			√		[54]

*P* denotes a vector of *P* values, Z denotes a vector of *Z* scores, *W* is a vector of weights, R denotes the SNP-by-SNP correlation matrix, *τ* indicates a fixed value that *P* is less than in TPM, with the default being 0.2; *k* is the number of *P* values to be combined in RTP, ARTP, ART, ART-A, with the default value being 2/*M*, where *M* is the number of SNPs for a given gene; *a* is a shape parameter in GM, with the default being 0.0383; *N* is the sample size

*MLR* Multiple linear regression, *FLM* Functional multiple linear regression model, *HC* Higher criticism test, *GHC* Generalized higher criticism test, *BJ* Berk–Jones test, *GBJ* Generalized Berk–Jones test, *DOT* Decorrelation by orthogonal transformation, *BT* Burden test, *SKATO* Optimal sequence kernel association test, *SKAT* Sequence kernel association test, *Simes* Simes’s test, *FCP* Fisher combined probability, *TPM* Truncated product method, *RTP* Rank truncated product, *ART* Augmented rank truncation, *ART-A* Adaptive augmented rank truncation, *GM* Gamma method, *GATES* Gene-based association test that uses extended Simes procedure, *HMP* The harmonic mean *P* value test, *ACAT* Aggregated Cauchy association test

On the other hand, some methods efficiently obtain their *P* value in an analytical way (e.g., SKAT and HMP) [17, 45, 51, 54, 75], whereas other yield *P* value via a simulation-based algorithm (e.g., GATES) [79], which would be time-consuming. Moreover, besides the general input of *Z*-scores (or *P* values) and LD matrix, some methods additionally require tuning parameters to first remove potentially null SNPs which have large *P* values [78]. From a modeling perspective, some methods (e.g., MLP and FLM) were built under the framework of fixed-effects model [45, 76, 77], whereas other (e.g., SKAT and SKATO) were established within the context of random-effect model [21, 24].

**Fig. 1:**
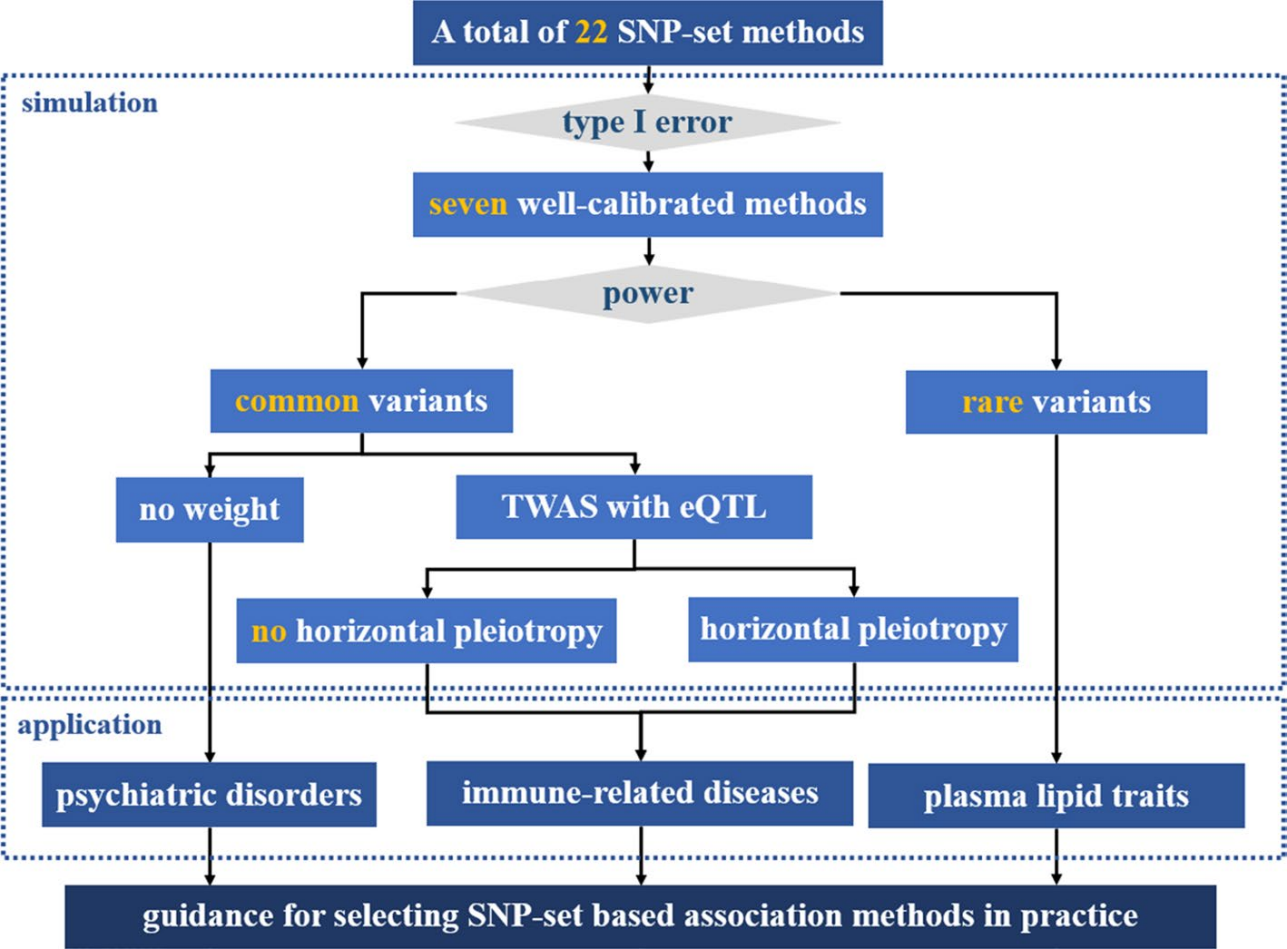
Statistical analysis framework for the theoretical and application comparison of SNP-set based association methods with summary statistics

An overview of the 22 SNP-set methods with their corresponding modeling characteristic is summarized in Table 1, with technical details given in the Additional files 1 and 2. Three important applications of these SNP-set based association approaches to various genomic research fields would be discussed below (Fig. 1). The R code for implementing each method is freely available at https://github.com/biostatpzeng/MCA. It needs to first point out that we here did not consider some other SNP-set methods as they enjoy the similar principle of approaches described in the present work. For instance, fastBAT [80] and MAGMA [17] were constructed based on the same rationale of SKAT.

### LD matrix estimation

In general, the LD matrix required in some of the these SNP-set methods (e.g., SKAT) is computed with genotypes of ancestry-matched individuals from an external reference panel such as the 1000 Genomes Project [81]. Denote **G** the standardized genotypes matrix of a given gene, and *n* the sample size of the reference panel. Intuitively, the empirical LD, ${\hat{\mathbf{R}}}$ = **G**^*T*^**G**/(*n *− 1), can be used, which however is in general not well-conditioned in the sense that the smaller eigenvalues of ${\hat{\mathbf{R}}}$ are underestimated because *n* is often not sufficiently large [82]. As a result, it would lead to inflated false discoveries. To handle this issue, many sophisticated approaches have been proposed to calculate large-dimensional covariance and correlation matrices [83]. We here estimate LD using a simple, shrinkage fashion relying on the empirical one: ${\mathbf{R}} =\updelta \times {\hat{\mathbf{R}}} + \left( {1 -\updelta } \right) \times {\mathbf{I}}$, where δ is the shrinkage parameter and **I** is the identify matrix. We set δ to 0.95 throughout our analyses following prior studies [63, 84, 85].

### Numerical studies for evaluating type I error control and power

#### Simulation with common variants

To evaluate the performance of each SNP-set method, we first conducted numerical studies to investigate their behaviors in type I error control and power with common SNPs (those with minor allele frequency (MAF) ≥ 0.05). To make our numerical studies as realistic as possible, we produced the phenotype (***Y***) based on real genotypes of 4901 individuals available from the Wellcome Trust Case Control Consortium (WTCCC) study [86]. To this goal, we obtained a set of 550 genetic variants that were located within either 100 kb upstream of the transcription start site or 100 kb downstream of the transcription end site of the gene *CEPT1* on chr1. Note that, the selection of this gene was to some extent arbitrary. To generate the genotype matrix (**G**), we randomly selected *M* (= 50, 200 or 500) continuous SNPs to maintain LD structure, and simulated ***Y*** via a linear model ***Y*** = **G*β*** + ***ε***, with ***β*** the vector of effect sizes and ***ε*** the vector of normally distributed residual errors.

To assess power for every method, we made three diverse scenarios of modeling assumptions on effect sizes: (i) sparse case: among these *M* selected SNPs, only 5%, 20% or 50% were at random selected to have substantial impacts on ***Y*** while the remaining had zero effects, corresponding the sparse setting where only a fraction of genetic variants were causal; the non-zero effect sizes were distributed in terms of a standard normal distribution; (ii) polygenic case: all SNPs had non-zero effects on ***Y*** and their effects sizes following a standard normal distribution, or a standard double exponential distribution, or a standard *t*-distribution, corresponding the polygenic setting where the effect sizes of SNPs might have distinct distributions; (iii) mixed case: all SNPs had relatively small non-zero impacts on ***Y*** with their effects sizes following a standard normal distribution, but 5%, 20% or 50% SNPs were randomly selected to have additional influences, corresponding the hybrid modeling assumption made by Bayesian sparse linear mixed model [87] and latent Dirichlet process regression [64].

In all scenarios, we re-scaled the simulated SNP effect sizes on ***Y*** and residual errors so that the phenotypic variance explained (PVE) by genetic component was 0.3%, 0.5% or 1%; where PVE = var(**G*β***)/(var(**G*β***) + var(**ε**)) [31]. Afterwards, we performed the single-marker analysis on the phenotype with the selected *M* SNPs to obtain their marginal *Z*-scores or *P* values using a linear regression model [9]. These summary statistics would be taken as input to fit various SNP-set association methods, with corresponding genotypes of 503 European individuals from the 1000 Genomes Project as the reference panel to calculate LD if needed. We simply set ***β* = **0 and run 10^5^ replications when assessing the type I error control, with the type I error primarily evaluated via the ratio between the empirical type I error and the given significance level. We repeated our numerical study 10^3^ times for power evaluation, with the power calculated by the proportion of *P* values less than a given significance level *α* of 10^–5^.

#### Simulation with rare variants

Some SNP-set methods (e.g., burden, SKAT and SKATO) were specially designed for analyzing rare variants although they were also often used for common variant association analysis as we assessed above; we hence performed a simulation to examine the performance of these methods in rare variant association study. First, we obtained a set of 759 rare variants (MAF < 0.05) located within the gene *SUSD2* on chr22 from 337,198 independent individuals of European descent in the UK Biobank cohort [88]. Then, we randomly selected 15,000 individuals to generate phenotype and another 5,000 individuals as the reference panel to calculate LD. Note that these individuals were always fixed throughout this simulation. Like the same single-marker analysis in the first simulation, we conducted the simple linear regression to obtain marginal *Z*-scores or *P* values for each rare variant. Following previous work [25, 45], we calculated the weight via the beta distribution density function of MAF with the two shape parameters being 1 and 25, and further scaled these weights so that their summation was one. The parameters for type I error and power evaluations were set the same as those used in the first simulation.

#### Simulation by incorporating eQTL weights

For multilocus association analysis, it generally incorporates other types of omics data or functional annotation as weights, which is often more powerful than using GWAS summary statistics alone and can provide more biologically meaningful results [28, 31–33]. For example, the recently popular TWAS can be viewed as a linear weighted SNP-set analysis [89], which methodologically amounts to BT [31]; naturally, SKAT and SKATO can be considered a quadratically weighted version of TWAS [90]. The attractive property of TWAS is that it can prioritize causal genes in GWASs by incorporating eQTL weights in terms of the viewpoint of Mendelian randomization [91]. However, we do not recognize that other SNP-set methods could be interpreted in such a similar manner.

Therefore, we here conducted an additional simulation within the two-stage TWAS framework. The detail of simulation setting was described in our previous work [31]. For simplicity, in the first stage of TWAS, we only considered the polygenic case with PVE = 5% and selected 200 continuous genetic variants in the transcriptome data. Specifically, we generated eQTL weights (***w***) and simulated gene expression (***e***) using genotypes (**G**_1_) of 465 individuals from the Geuvadis project [65]; that is, *E*(***e***) = **G**_1_***w*** with **G**_1_ the genotypes of SNPs around the gene *CEPT1* on chr1. In the second stage of TWAS, we produced the phenotype (***Y***) based on genotypes (**G**_2_) of *CEPT1* from WTCCC; that is ***Y*** = (**G**_**2**_***w***)*θ* + ***ε***, with ***ε*** the residual simulated from a standard normal distribution and *θ* = 0.10 or 0.20.

The above simulation of TWAS explicitly assumed the absence of direct cis-SNP effects [92], which might be not true because of ubiquitous horizontal pleiotropy [31, 93–96]. Thus, we carried out another simulation under the case of horizontal pleiotropy by generating ***Y*** = (**G**_**2**_***w***)*θ* + **G**_**2**_***b*** + ***ε***, where ***b*** was considered random effect following a normal distribution with mean zero and variance 0.05. The setting of other parameters was the same as the case without horizontal pleiotropy.

We applied the maximum likelihood method through the computationally efficient PX-EM algorithm [96–101] to estimate joint effects (i.e., eQTL weights ***w***) for the simulated transcriptome data in the first stage, and used the linear regression model to obtain marginal *Z*-scores or *P* values for the GWAS data in the second stage [9]. Then, the estimated eQTL weights were included into these SNP-set methods via suitable transformations. Specifically, the squared eQTL weights were used for SKAT and SKATO, and the scaled absolute weights were applied in ACAT and HMP, while the original eQTL weights were employed in BT.

### Real data applications

#### Common variant association analysis for psychiatric disorders

Psychiatric disorders are one of the most enigmatic maladies in medicine [102]; although their existence has been known for many years [60, 103] and their impact on the public health well-documented [104], relatively little remains known with regards to their causal factors and fundamental neurobiology in despite of a considerable corpus of genomic research [59, 105, 106]. Therefore, identifying potential genetic loci for early diagnosis and unraveling risk factors for prevention and treatment becomes critical in the clinic. To this goal, we applied the SNP-set methods that were demonstrated to be well-calibrated to European-only summary statistics of six psychiatric disorders yielded from PGC [59, 60] (Additional file 2: Table S1), including ADHD (*N* = 53,293), ASD (*N* = 46,350), BIP (*N* = 51,710), CU (*N* = 184,765), MDD (*N* = 480,359), and SCZ (*N* = 77,096).

We defined the set of SNPs located within a gene according to the annotation file provided by VAGIS [107], in which we considered 100 kb extension upstream of the transcription start site and 100 kb downstream of the transcription end site of that gene. Again, we leveraged genotypes of 503 European descents from the 1000 Genomes Project as the reference panel when the LD matrix was required. To avoid numerical instability, we only considered genes with at least ten SNPs following our prior work [34, 35], and further performed an enrichment analysis for all identified genes using FUMA [108].

#### Rare variant association analysis for four plasma lipid traits

Using these SNP-set methods, we here performed rare variant SNP-set association analysis for four plasma lipid traits (Additional file 2: Table S1), including HDL, LDL, TC, and TG. The summary statistics were publicly available from GLGC [61], which analyzed ~ 300,000 individuals of European ancestry genotyped with the HumanExome BeadChip (exome array). Following previous studies [61, 109], we considered 179,884 rare variants with MAF < 0.05 and defined the set of SNPs located within either 500 kb extension upstream of the transcription start site or 500 kb downstream of the transcription end site of a given gene in terms of the annotation file provided by GENCODE (version 12) [110]. We only analyzed 15,378 genes that contained at least two rare variants, and used genotypes from the UK Biobank [88] as the reference panel in this rare variant association analysis.

#### TWAS analysis for nine immune-related diseases

We finally applied these SNP-set approaches under the TWAS context. Following our prior work [31, 64], we focused on 15,810 genes and estimated eQTL weights for every gene with BSLMM [87, 111] in the Geuvadis project [65]. Because the gene expression of Geuvadis was measured in lymphoblastoid cell line, which was an immune-related cell type, we thus only considered GWAS summary statistics of nine immune-related diseases (Additional file 2: Table S1), including inflammatory bowel disease (IBD: *N* = 34,652), ulcerative colitis (UC: *N* = 27,432), Crohn’s disease (CD: *N* = 20,883), systemic lupus erythematosus (SLE: *N* = 23,210), PBC (*N* = 13,239), primary sclerosing cholangitis (PSC: *N* = 24,751), rheumatoid arthritis (RA: *N* = 37,681), multiple sclerosis (MS: *N* = 68,379), and OST (*N* = 63,608). Details with regards to these data can be found in the original papers and the quality control procedure for data processing was described in our previous studies [31, 63, 64]. We here focused only on common SNPs and applied genotypes from the 1000 Genomes Project as the reference panel.

## Results

### Results of numerical studies

#### Assessing the type I error rate

We first evaluated the performance of type I error control for all these compared methods with common variants (Table 2) and rare variants (Additional file 2: Table S2) under the simulated null scenarios. Note that, we here defined a type I error well-controlled method as the ratio of empirical type I errors (divided by the significance level α) between 0.8 and 1.2 as done in [112, 113]. Notably, the performance of type I error control (i.e., inflated, well-controlled, or conservative) of these methods was almost consistent regardless of using common or rare variants. Among the LD-free *P* value combination methods, we found that only HMP, ACAT, minP and Simes generated a well-calibrated type I error control. SimpleM was conservative; in contrast, FCP, TPM, RTP, ART, ART-A, GM, and gene-based association test that uses extended Simes procedure (GATES) were inflated.

**Table 2:** Ratio between the empirical type I error and the given significance level estimated over 10^5^ simulations under common variants

Method	Significance level α				Performance of type I error control		
	0.05	0.01	0.001	Average	Inflated	Well-controlled	Conservative
MLR	0.00	0.00	0.00	0.00			√
FLM	0.00	0.00	0.00	0.00			√
HC	1.33	1.82	2.33	1.83	√		
GHC	1.26	1.65	1.94	1.62	√		
BJ	1.29	1.64	1.97	1.63	√		
GBJ	0.85	1.32	1.71	1.29	√		
DOT	0.00	0.00	0.00	0.00			√
BT	1.04	1.07	1.10	1.07		√	
SKAT-O	1.08	1.18	1.11	1.12		√	
SKAT	1.02	1.08	1.08	1.06		√	
Simes	0.82	0.82	0.82	0.82		√	
FCP	5.29	21.88	174.81	67.33	√		
TPM	2.45	10.39	86.81	33.22	√		
RTP	3.76	14.71	110.07	42.85	√		
minP	0.88	0.82	0.77	0.82		√	
ART	4.15	16.51	126.91	49.19	√		
ART-A	1.17	3.05	12.97	5.73	√		
GM	2.01	7.43	52.03	20.49	√		
SimpleM	0.39	0.41	0.41	0.40			√
GATES	1.47	1.53	1.51	1.50	√		
HMP	0.87	1.01	1.06	0.98		√	
ACAT	1.04	1.08	1.07	1.06		√	

Determine whether a SNP-set method was inflated, well-controlled or conservative according to the average ratio between the empirical type I error and the given significance level over 10^5^ simulations. inflated: ratio > 1.2; well-controlled: 0.8 ≤ ratio ≤ 1.2; conservative: ratio < 0.8

We also observed that not all the LD-dependent methods could behave well in controlling type I error. For example, BJ and HC, as well as their generalized versions (i.e., GBJ and GHC), were inflated under our simulation scenarios, while DOT, multiple linear regression (MLP) and functional multiple linear regression model (FLM) were much conservative. Three methods (i.e., BT, SKAT and SKATO) could effectively maintain the control of type I error. Because some of these methods failed to control the type I error at a nominal level (inflation or much conservativeness), we therefore only considered seven well-calibrated methods, including BT, SKATO, SKAT, Simes, minP, HMP and ACAT in our subsequent analyses.

#### Estimated statistical power for common variants with no weights

**Fig. 2:**
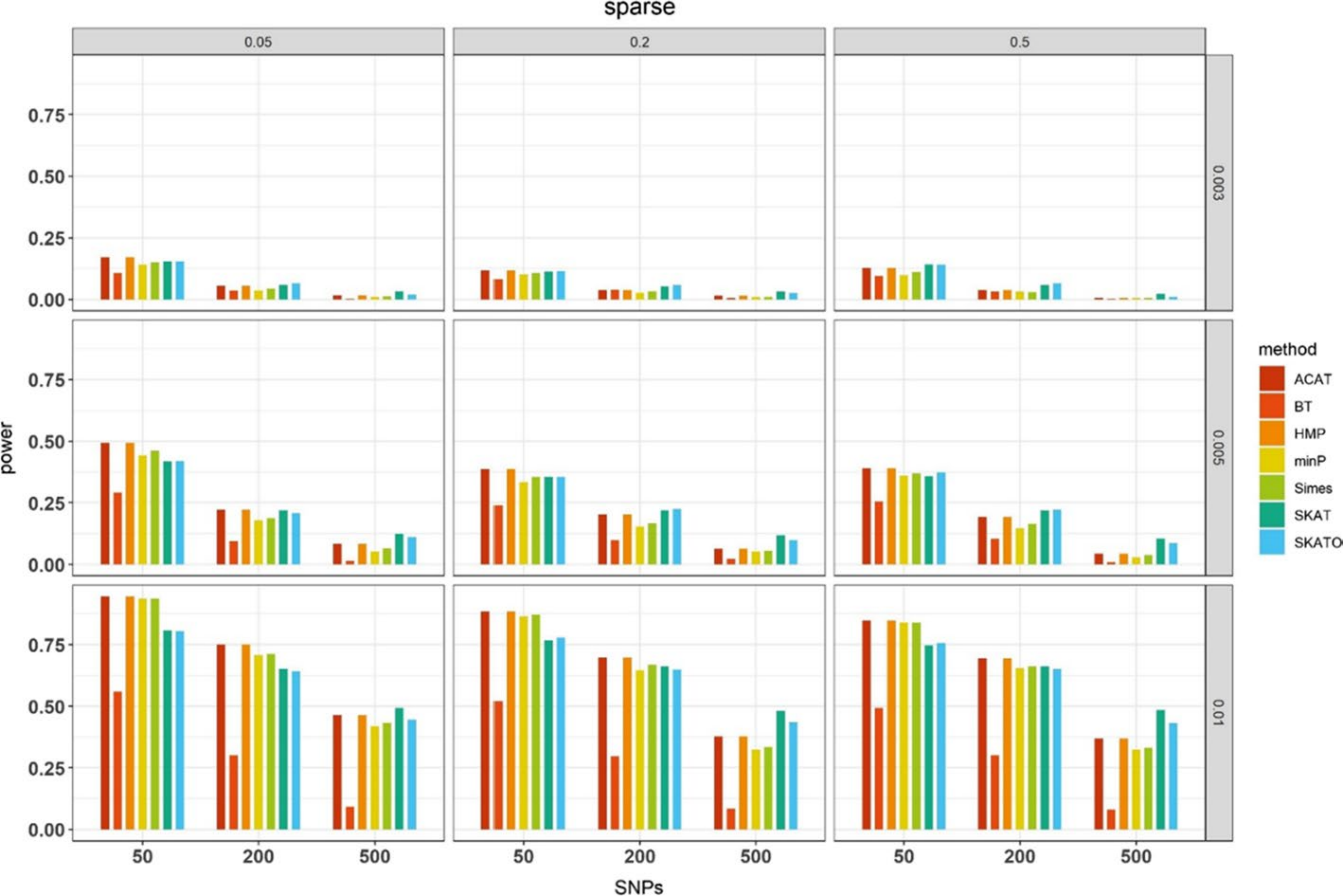
Estimated power for the seven SNP-set methods under the sparse case with a significance level *α* of 10^−5^. Here, PVE = 0.3%, 0.5% or 1% at the right side, the number of causal SNPs (prop) = 0.05, 0.20 or 0.50 on the top, the number of the total analyzed SNPs = 50, 200 or 500 on the x-axis. The power was estimated across 10^3^ replications

When comparing the power of the rest seven methods (Additional file 2: Table S3), we primarily displayed their results obtained under the sparse simulation setting (Fig. 2), but relegated the results of the polygenic and mixed cases to Additional file 2: Fig. S1. Particularly, we observed several important patterns as follows. First, in general, when PVE was small (e.g., 0.3%), we found that HMP and ACAT had higher power compared to SKAT and SKATO when the number of analyzed SNPs (denoted by *M*) and (or) the proportion of causal SNPs (denoted by prop) were small; that is, HMP and ACAT outperform other methods when there were very less effective SNPs. However, SKAT and SKATO were better than HMP and ACAT as the increase in *M* and (or) prop. For example, the powers of SKAT and SKATO were 0.155 and 0.154 respectively when prop = 5% and *M* = 50, which were lower than HMP (0.171) and ACAT (0.171); whereas the powers of SKAT and SKATO were 0.020 and 0.033 respectively when prop = 5% and *M* = 500, which were more powerful than HMP (0.016) and ACAT (0.016). The similar patterns were consistently observed under the polygenic and mixed cases. Second, unlike prior studies [22, 114], as our simulations were relatively general and no very extreme settings were considered, we did not find there existed a consistent advantage of SKATO over SKAT, or vice versa; we also did not observe a substantial difference between HMP and ACAT.

Third, under the same simulation setting for causal SNPs, all these methods suffered from power loss as the number of null genetic variants increased. For example, when PVE = 1.0% and 5% of selected SNPs were causal, the power of ACAT reduced from 0.946 for 50 selected SNPs to 0.463 when the total number increased to 500. Such an observation is not unexpected because the increased noise SNPs diluted the true association signals. Fourth, both Simes and minP behaved well across all simulation settings; however, they were underpowered compared to SKAT, SKATO, HMP and ACAT even under the relatively sparse settings where only 5% of selected SNPs were causal.

Some studies previously stated that minP could exhibit higher power in the very extreme case where only one SNP showed an impact on the phenotype [51]. In order to validate such finding, we conducted an additional numerical study, in which one out of 200 SNPs was randomly causal. Under this case, we found that the power of minP was indeed higher (0.465) compared to BT (0.118), SKATO (0.267), SKAT (0.291) and Simes (0.455), but still slightly lower than HMP (0.494) or ACAT (0.495).

Fifth, as both positive and negative SNP effect sizes were simulated in all our simulation settings, BT had the lowest power across these scenarios, similar as that observed in prior work [21, 22, 25]. In order to assess the power of these methods under the situation that effect sizes of all the causal SNPs were in the same direction, we took the absolute value of simulated SNP effect sizes in the sparse case where PVE = 0.3% and prop = 5%, 20% or 50%. As expected, we observed that the power of BT was now considerably higher than that of other methods across these simulation scenarios (Table 3), in line with the prior finding [21, 25].

**Table 3:** Estimated powers of the seven methods under sparse case where PVE = 0.3%, and prop = 5%, 20% or 50% of SNPs were randomly selected to be causal with the same direction of effect sizes

Prop	BT	SKATO	SKAT	Simes	minP	HMP	ACAT
0.05	0.350	0.065	0.059	0.044	0.037	0.054	0.054
0.20	0.379	0.058	0.062	0.039	0.035	0.051	0.051
0.50	0.363	0.066	0.058	0.038	0.038	0.047	0.047

To be more intuitive to compare the power difference in diverse SNP-set methods, we ranked their estimated powers in each setting and averaged the rank across simulation scenarios (Additional file 2: Fig. S2). Totally, except BT, we found that SKAT, SKATO, HMP, ACAT, Simes and minP were robust and powerful under distinct simulation cases, while the SKAT, SKATO, HMP and ACAT were much better than Simes and minP. Particularly, SKAT and SKATO had a remarkable advantage under the polygenic and mixed situations, whereas HMP and ACAT seemed to outperform others in the sparse setting.

#### Estimated statistical power for rare variants

**Fig. 3:**
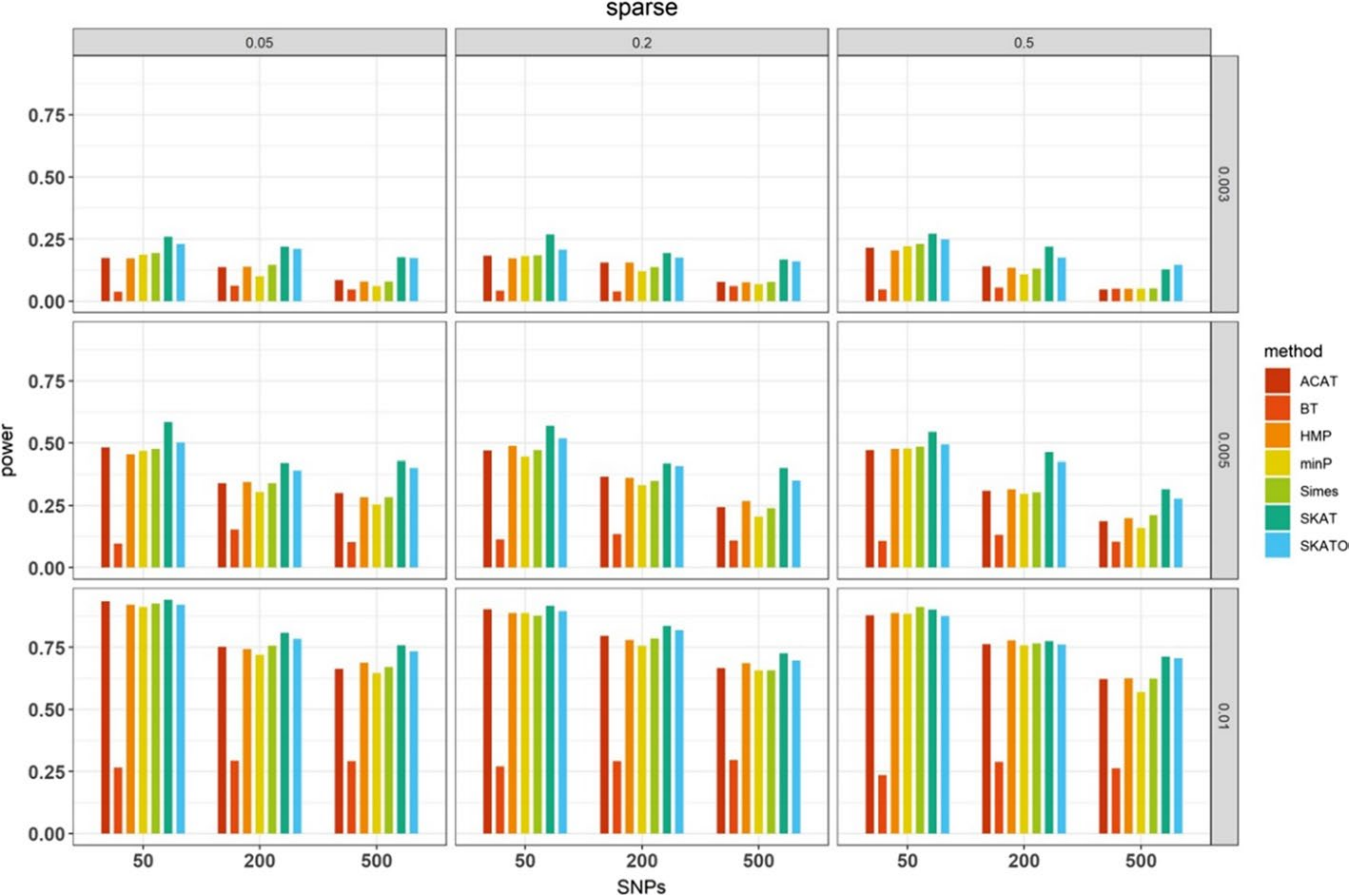
Estimated power for the seven SNP-set methods in the case of rare variant association study under the sparse case with a significance level α of 10^−5^. Here, PVE = 0.3%, 0.5% or 1% at the right side, the number of causal SNPs = 0.05, 0.20 or 0.50 on the top, the number of the total analyzed SNPs = 50, 200 or 500 on the x-axis. The power was estimated across 10^3^ replications

Finally, as can be anticipated, SKAT and SKATO, two specially designed methods for rare variant association analysis, showed evident advantage over other approaches especially when PVE was low (Fig. 3 and Additional file 2: Fig. S3). Despite not originally designing for rare variants, the two LD-free methods including ACAT and HMP also behaved satisfactorily although they were inferior relative to SKAT and SKATO across most simulation settings. For example, under the sparse case, when the number of SNPs was 200 and 50% of them were causal, the power gain of SKAT over ACAT increased from 1.7% to 56.9% when PVE reduced from 1 to 0.3% (Fig. 3). In addition, we observed that BT, Simes and minP were generally underpowered in our simulated scenarios for identifying significant association of rare variants with phenotype.

#### Estimated statistical power under the TWAS framework

**Fig. 4:**
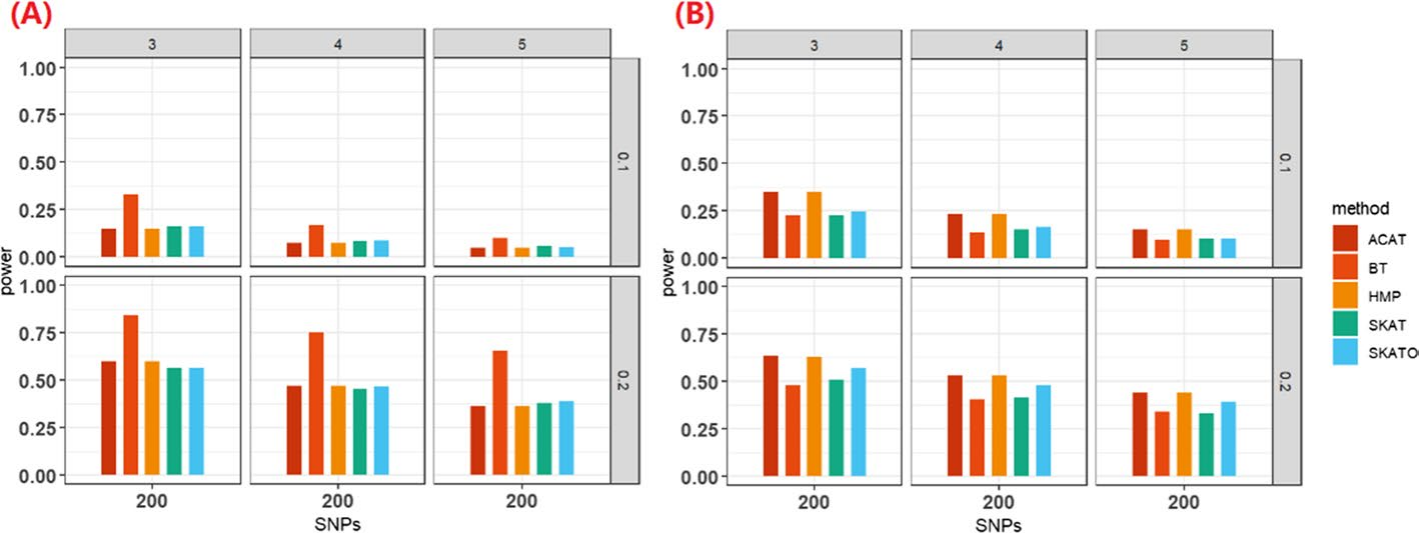
(**A**) Estimated power for SNP-set methods under the polygenic TWAS framework of no horizontal pleiotropy. (**B**) Estimated power for SNP-set methods under the TWAS polygenic framework of horizontal pleiotropy. Here, *θ* = 0.1 or 0.2 at the right side, the − log_10_(α) = 3, 4, or 5 on the top, the number of the total analyzed SNPs = 50, 200 or 500 on the x-axis. The power was estimated across 10^3^ replications

Within the simulation of TWAS framework, we primarily performed methods which could take eQTL weights as input (i.e., ACAT, HMP, BT, SKAT and SKATO). Under the case of no horizontal pleiotropy, ACAT, HMP, SKAT and SKATO behaved comparably (Fig. 4A); while BT, analogously to the original TWAS method [32, 33], had much higher power, and the advantage became more pronounced as the increase of genetic effect. For example, the power advantage of BT over SKAT increased from 0.044 to 0.276 if the effect size changed from 0.1 to 0.2. In contrast, under the case of horizontal pleiotropy, BT suffered from substantial power reduction compared to other multilocus methods (Fig. 4B), in line with our previous finding [31]. Furthermore, ACAT and HMP behaved better than SKATO, and SKAT had a relatively low power among these approaches. However, as mentioned before, unlike SKAT and SKATO, ACAT and HMP cannot be explained from the perspective of TWAS analysis. To facilitate comparison, we further summarized the power performance of these methods evaluated under distinct simulation scenarios in Table 4.

**Table 4:** Summary performance of these SNP-set based association methods in the power evaluation of simulation studies and in real-data applications to distinct fields

	Common variants			Rare variants
	Unweighted	TWAS with eQTL weights		
		No horizontal pleiotropy	Horizontal pleiotropy	
Simulation	HMP ACAT	BT	HMP ACAT	SKAT SKATO
Application	HMP ACAT	HMP SKATO		SKAT SKATO

The methods listed in the table were selected in terms of their power in the simulation studies or based on the number of identified genes in the real-data applications

#### Comparing the computing time

We here compared the running time of the seven SNP-set methods based on an Intel(R) Xeon(R) Gold 5118 CPU (2.30 GHz) with 125 GB of RAM. The total computation times across 10^3^ replications are shown in Table 5 and Additional file 2: Table S4. As anticipated, it is found that the number of SNPs had substantial impact on computation time, while other simulation parameters had a negligible influence. For example, under the sparse case when the number of SNPs was 50, the average computation time of ACAT was only 3.98 s; it increased to 279.46 s when the number of SNPs was 500. Overall, LD-free methods (e.g., HMP and ACAT) were much faster than those LD-dependent ones (e.g., SKAT and SKATO). Except SKATO which was an optimization-search method, all other methods were computationally quick under various simulation scenarios, with ACAT the fastest one under the same settings.

**Table 5:** Total computation times (second) of 10^3^ simulations under the sparse case with PVE = 0.5% and only 20% of simulated SNPs were selected to have substantial impacts on phenotype

*M*	BT	SKATO	SKAT	Simes	minP	HMP	ACAT
50	4.10	483.83	7.16	3.98	4.48	4.12	3.83
200	58.59	1234.77	70.84	58.35	60.55	58.62	52.71
500	297.73	1561.44	524.48	296.46	312.76	295.70	279.46

### Results of real data applications

#### Identified genes associated with psychiatric disorders

**Fig. 5:**
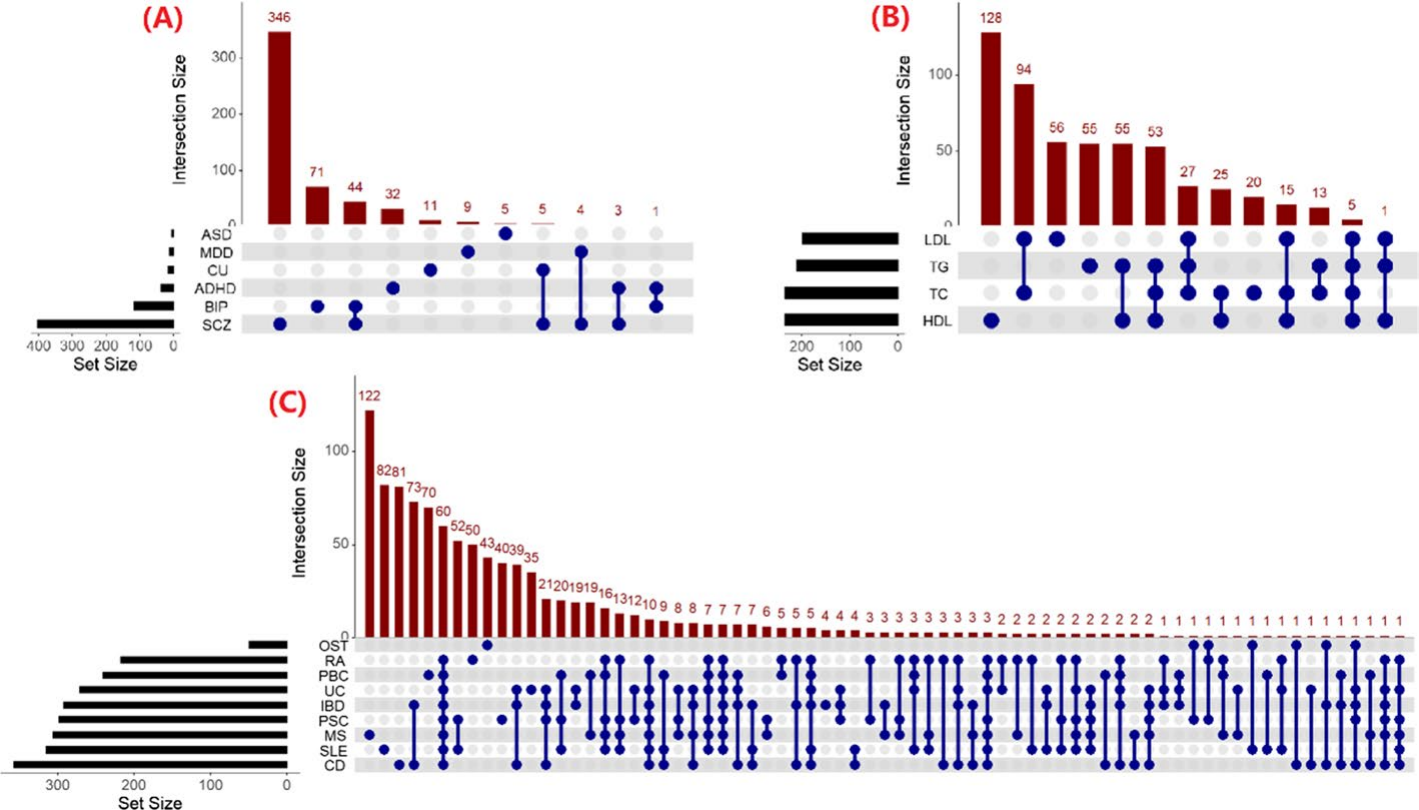
Upset plot to illustrate the number of identified genes shared across distinct SNP-set methods for six psychiatric disorders (**A**), four plasma lipid traits (**B**), and nine immune-related diseases (**C**)

Applying the seven methods to psychiatric disorders, we identified a total of 588 (531 unique) genes associated with these disorders (Bonferroni-corrected *P* value < 0.05) (Fig. 5A), including 172 novel genes simply defined as loci not including SNPs with *P* value > 5 × 10^–8^. More results were given in Additional file 2: Fig. S4 and Table S5. Particularly, there were 305 schizophrenia (SCZ)-associated genes but only 2 major depression disorder (MDD)-associated genes. In addition, we found that approximately 10.7% of identified genes showed pleiotropic association with at least two disorders. For example, there were 43 genes showing simultaneously significant association with SCZ and bipolar disorder (BIP), which was consistent with the highly common genetic foundation underlying the two disorders [34, 35, 105, 115–119]. We discovered that HMP identified the most associated genes for four disorders including attention-deficit/hyperactivity disorder (ADHD: 27 genes), cannabis use (CU: 14 genes), BIP (81 genes) and SCZ (307 genes), while SKAT detected more associated genes for the remaining two disorders including autism spectrum disorder (ASD: 4 genes) and MDD (10 genes) (Table 6). The enrichment analysis demonstrated that some of these detected genes were significantly enriched in the pancreas, brain, and liver tissues (Additional file 2: Fig. S5), consistent with prior findings [34, 35].

**Table 6:** Identified genes associated with six psychiatric disorders, four plasma lipid traits and nine immune-related diseases under various real-data applications

Phenotype	BT	SKATO	SKAT	Simes	minP	HMP	ACAT	Total
*Six psychiatric disorders under the context of common variant association analysis*								
ADHD	6	25	25	24	25	**27**	26	36
ASD	1	**4**	3	2	2	3	3	5
BIP	7	65	74	57	59	**81**	80	116
CU	0	10	12	10	11	**14**	**14**	16
MDD	2	**10**	9	1	3	5	5	13
SCZ	11	282	299	295	299	**307**	298	402
*Four plasma lipid traits within the framework of rare variant association analysis*								
HDL	22	221	222	193	192	**239**	215	282
LDL	65	147	**152**	146	147	150	144	198
TG	78	203	**205**	168	168	168	168	209
TC	146	**219**	218	198	198	197	198	252
*Nine immune-related diseases under the setting of TWAS analysis*								
IBD	22	146	94			**253**	249	292
UC	13	175	129			**222**	219	271
CD	22	222	144			**282**	272	357
SLE	101	180	104			**267**	266	315
PBC	102	**149**	65			122	121	240
PSC	92	210	138			**223**	221	298
RA	46	157	139			**165**	141	217
MS	106	137	183			**205**	201	306
OST	10	**36**	0			5	5	49

The maximum number of associated genes is highlighted in bold for each disease. Methods including Simes and minP which cannot incorporate eQTL weights were excluded from the TWAS analysis of the nine immune-related diseases

*ADHD* Attention-deficit/hyperactivity disorder, *ASD* Autism spectrum disorder, *BIP* Bipolar disorder, *CU* Cannabis use, *MDD* Major depression disorder, *SCZ* Schizophrenia, *HDL* High-density-lipoprotein cholesterol, *LDL* Low-density-lipoprotein cholesterol, *TG* Triglycerides, *TC* Total cholesterol, *IBD* Inflammatory bowel disease, *UC* Ulcerative colitis, *CD* Crohn’s disease, *SLE* Systemic lupus erythematosus, *PBC* Primary biliary cirrhosis, *PSC* Primary sclerosing cholangitis, *RA* Rheumatoid arthritis, *MS* Multiple sclerosis, *OST* Osteoarthritis

In order to further compare HMP and SKAT in our application of psychiatric disorders, we created a bar plot for the proportion of significant cis-SNPs (*P* < 5 × 10^–8^) for each of the 531 unique genes (Additional file 2: Fig. S6). It was observed that the *P* value obtained by SKAT became more significant (smaller) than that of HMP as the increase of the proportion of significant *cis-*SNPs of an associated gene (the genetic architecture of a gene becomes from sparsity to polygenicity), which is consistent with the finding described in the simulation study above.

#### Identified genes associated with plasma lipid traits

When applying these SNP-set methods to rare variants of four plasma lipid traits (Fig. 5B), we found that SKAT and SKATO identified more genes associated with three lipids (except high-density-lipoprotein cholesterol (HDL)) than other approaches, and BT detected the minimal genes among all compared methods, consistent with the results given in the simulation of rare variant association analysis. Specifically, we identified 282 associated genes for HDL (Bonferroni-corrected *P* value < 0.05), 198 for low-density-lipoprotein cholesterol (LDL), 209 for triglyceride (TG), and 252 for total cholesterol (TC), respectively (Table 6), which involved a total of 547 unique genes (496 novel) (Table S6). Among these, 288 (52.7%) were shared in as least two lipids, and five genes (*MAP3K2*, *IMP4*, *ITGB1BP1*, *TP53I3*, and *MLLT4-AS1*) were associated with all the four lipid traits, which were confirmed by previous studies [120, 121]. In terms of the enrichment analysis, we did not find these identified genes were significantly differentially expressed in any GTEx tissues (Additional file 2: Fig. S7), which can be expected as FUMA only included common genetic variants [108]. Nevertheless, we observed suggestive evidence that these genes likely enriched in the liver, pancreas, and lymphocytes tissues, supporting by prior work [122–125].

#### Identified genes associated with immune-related diseases

When applying these SNP-set methods to nine immune-related diseases by incorporating eQTL information under the TWAS context, we discovered a total of 1,029 genes (446 novel) (Bonferroni-corrected *P* value < 0.05) (Table 6, Additional file 2: Table S7 and Fig. 5C), approximately half (48.8%) of which showed pleiotropic association with at least two diseases. It was observed that HMP identified the most associated genes (except primary biliary cirrhosis (PBC) and osteoarthritis (OST)), followed by ACAT. This observation was consistent with the simulation result. In addition, SKATO discovered more genes compared to SKAT, again in line with the corresponding simulation result. In contrast, BT detected much less significant genes. These findings further implied that these SNP loci likely showed widespread horizontal pleiotropy on the analyzed immune-related diseases. Furthermore, these detected genes were significantly enriched in the lymphocytes tissue (Additional file 2: Fig. S8), consistent with the pathological mechanism that the immune system was se associated with these diseases [126–128]. Based on the number of identified genes, we finally summarized the performance of the seven SNP-set methods in various real-data applications in Table 4.

## Discussion

As part of great efforts to explain more heritability of phenotypes and enhance power in association studies by integrating other types of omics data [5], SNP-set analysis has already become a powerful alternative to single-marker analysis. In the present study, we performed a comprehensive comparison for 22 SNP-set methods that can be applied with only summary statistics. Through extensive simulation studies, we demonstrated that some LD-free methods were inflated in controlling type I error, which might be a direct consequence of not accounting for correlation between SNPs. The similar inflation pattern was also observed for some of conventional LD-free *P* value combination methods (i.e., Fisher’s method) in TWAS when multiple gene expression prediction models were employed to construct weights for expression quantitative trait loci [63]. In addition, as the number of SNPs in a gene might be very large up to hundreds of thousands and often highly correlated due to pervasive LD, it was discovered that fixed-effect based methods (e.g., MLP and FLM) were generally conservative because of the loss of degree of freedom in these methods.

Particularly, among these compared methods, we only identified seven methods which could correctly control type I error, including BT, SKATO, SKAT, Simes, minP, HMP and ACAT. In total, these well-calibrated methods had varying performance in power evaluation. For example, prior studies showed minP was powerful in the case in which the association signals were extremely sparse [51, 129]. However, because of only considering the top signal across genetic variants, minP would add little to our knowledge of the association at the gene level when the top signal was genome-wide significant. In fact, minP cannot solve the primary task of SNP-set analysis because it did not consider every locus in a region and thus cannot effectively combine all available information. As a result, minP often had limited power as demonstrated in our simulations and real data applications.

By contrast, in many cases we found that integrating individual genetic variants (e.g., BT, SKAT and SKATO) together might be a more suitable manner for SNP-set analysis [21, 22, 130–132]. For instance, BT used the weighted or unweighted sum of linear test statistics [133, 134], which would have high power if all SNPs had the same effect size and the same effect direction. We also discovered that BT had better performance in eQTL integrative TWAS analysis in the absence of horizontal pleiotropy; however, BT suffered from a great power loss if the effect sizes were directionally different as demonstrated in both common and rare variant association analyses. On the other hand, SKAT and SKATO, two variance component score methods that were established with the sum of quadratic test statistics [21], were robust and particularly powerful in the presence of protective, deleterious and null variants. We demonstrate that SKAT and SKATO showed a significant advantage under the polygenic and mixed genetic architecture in common variant association study; we also confirmed the superiority of these two methods in detecting the association of rare variants with complex phenotype [21, 22, 41, 114].

Furthermore, we revealed that two LD-free methods (i.e., HMP [75] and ACAT [54]) appeared to be superior to other methods under the sparse genetic architecture in common variant association analysis. Despite not especially developing for rare variants, based on our limited experience of simulations with common variants and real-data applications, we demonstrated that ACAT and HMP also likely had the potential to be powerful methods for rare variant association analysis. In addition, the two approaches also showed better behavior in the two-stage TWAS analysis relative to other methods; unfortunately, they cannot be interpreted from the perspective of TWAS. Compared to other SNP-set aggregation methods, an important feature of ACAT and HMP is that their test statistics approximately or asymptotically follow certain null distributions (e.g., Landau distribution for HMP [75]) regardless of correlation structure between these SNPs and such an approximation is rather accurate even at very small tail area of the distribution. Consequently, one can obtain the *P* values of HMP and ACAT based on the right tail area of the respective approximate null distributions. Under regularity conditions, their performance is robust with respect to the number of SNPs, the weights, as well as the correlation among SNPs [54, 75]. Moreover, because of without requiring the knowledge of explicit correlation, compared to these LD-dependent methods (e.g., SKAT), HMP and ACAT have a wider applicability to many other cases where the correlation is too complicated to fit or reference panels cannot be available, such as multiple-tissue or multiple-model TWAS [62, 63] and spatial expression pattern identification in transcriptomic studies with multiple candidate kernels [135].

Finally, although we showed that some of these methods might be relatively slow, as all methods can be applied using GWAS summary statistics, they can be thus scalable to biobank-scale data. In summary, we evaluated 22 SNP-set methods using simulations and real data applications, and compared the robustness and effectiveness of these methods under diverse genetic architectures of phenotypes. However, our study had several limitations. First, in the real data analysis of six psychiatric disorders, we detected a number of significant genes and further showed that the identified genes may be functionally important for these disorders. However, there is no gold standard to accurately assess these methods in our real data application as the true associations of these discovered genes with the disorders are unknown; further follow-up studies are needed. Second, because of being extremely computationally expensive, we did not compared some computation-intensive SNP-set approaches (e.g., aSPUs [136] and VEGAS [107]) that utilized permutation testing rather than analytical solutions to obtain *P* values. For example, at least 10^7^ samplings would be needed to calculate a sufficiently accurate *P* value for aSPUs or VEGAS if the significance level was set to 10^–5^ in each test. In fact, according to our limited simulations we found that both aSPUs (performed with the R aSPU package (Version 1.50, Updated in 2021-06-28)) and VEGAS (performed with the COMBAT package (Version 0.04, Updated in 2018-01-14)) did not have much advantage over other methods. Third, because there were too many distinct genetic backgrounds needed to study; to be simple we only focused limited settings in our simulations. Some methods might be powerful in other uncovered scenarios. For instance, GBJ exhibited excellent single-gene effect separation but showed slight inflation in our simulation settings. In addition, DOT [67] was expected to gain power as the number of SNPs increases in scenarios where effect sizes varied markedly from SNP to SNP. However, if effect sizes for all SNPs were in fact very close to each other, the power of DOT decreased and behaved conservative. Fourth, since our work focused only on summary-level data, we cannot guarantee that our conclusions could be completely generalize to the setting with individual-level data. For example, we showed that summary-statistics based SKAT outperformed summary-statistics based minP in most simulation cases of the common variant association analysis with no weights; we were however not fully clear whether this conclusion remained true in individual-level data. Nevertheless, due to the concern of privacy in individual-data sharing and widespread availability of summary-level data, our finding was certainly more important and meaningful in practice. Fifth, we did not discuss how to further pinpoint these responsible ones after discovering the overall significance for a set of SNPs with the disease or trait. The step-down inference procedure introduced in [51] may be a promising strategy that can be employed to discriminate which specific SNPs likely drive the observed association signal. We reserve this as an interesting direction for future investigation.

## Supplementary Information

**Additional file 1.** Various gene-based association analysis methods.

**Additional file 2. Figure S1.** Estimated power for the seven SNP-set methods under the polygenic case (**A**) and the mixed case (**B**) with a significance level α of 10^−5^. Here, PVE = 0.3%, 0.5% or 1% at the right side, the number of causal SNPs (prop) = 0.05, 0.20 or 0.50 or the distribution of effect size including double, normal and *t* on the top, the number of the total analyzed SNPs = 50, 200 or 500 on the x-axis. The power was estimated across 10^3^ replications. **Figure S2.** Rank of power for the seven SNP-set methods under the sparse case (**A**), the polygenic case (**B**), and the mixed case (**C**) with a significance level α of 10^−5^. The number in each cell represents − log(P). normal: SNP effect sizes followed a standard normal distribution; double: SNP effect sizes followed a standard double exponential distribution; t: SNP effect sizes followed a standard t-distribution. **Figure S3.** Estimated power for the seven SNP-set methods in the case of rare variant association study under the polygenic case (**A**) and the mixed case (**B**) with a significance level α of 10^−5^. Here, PVE = 0.3%, 0.5% or 1% at the right side, the number of causal SNPs (prop) = 0.05, 0.20 or 0.50 or the distribution of effect size including double, normal and *t* on the top, the number of the total analyzed SNPs = 50, 200 or 500 on the x-axis. The power was estimated across 10^3^ replications. **Figure S4.** Upset plot to illustrate the number of identified genes shared across seven SNP-set methods for six psychiatric disorders. **Figure S5.** **A** Enrichment of differentially expressed pleiotropic genes associated with the six psychiatric disorders in terms of expression level across the 54 GTEx tissues. *P* values are shown in the y-axis with a scale of − log10. The bar in red represents significant enrichment after Bonferroni’s adjustment for multiple hypothesis tests; **B** Top 10 significant types of pathways in terms of the GO and KEGG enrichment analyses. *BP* Biological process, *CC* Cellular component, *MF* Molecular function. **Figure S6.** Bar plot of 531 unique genes associated with the six psychiatric disorders. The red color in the heatmap represents the rank of P values of SKAT and HMP; prop: the proportion of significant cis-SNPs (*P* < 5 × 10^−8^) within each associated gene. **Figure S7.** **A** Enrichment of differentially expressed pleiotropic genes related to the four plasma lipid traits in terms of expression level across the 54 GTEx tissues. *P* values are shown in the y-axis with a scale of − log10. The bar in red represents significant enrichment after Bonferroni’s adjustment for multiple hypothesis tests; **B** Top 10 significant types of pathways in terms of the GO and KEGG enrichment analyses. *BP* Biological process, *CC* Cellular component, *MF* Molecular function. **Figure S8.** **A** Enrichment of differentially expressed pleiotropic genes associated with the nine immune-related diseases in terms of expression level across the 54 GTEx tissues. *P* values are shown in the y-axis with a scale of − log10. The bar in red represents significant enrichment after Bonferroni’s adjustment for multiple hypothesis tests; **B** Top 10 significant types of pathways in terms of the GO and KEGG enrichment analyses. *BP* Biological process, *CC* Cellular component, *MF* Molecular function. **Table S1.** Summary information of the six psychiatric disorders, four plasma lipid traits and nine immune-related diseases. **Table S2.** Ratio between the empirical type I error and the given significance level estimated over 10^5^ simulations under rare variants. **Table S3.** Estimated power over 10^3^ simulations with common variants. **Table S4.** Total running time of 10^3^ simulations for the seven SNP-set methods under various simulation settings. **Table S5.** Identified genes associated with the six psychiatric disorders. **Table S6.** Identified genes associated with the four plasma lipid traits. **Table S7.** Identified genes associated with the nine immune-related diseases.

## Data Availability

All data generated or analyzed during this study are included in this article and its Additional files 1 and 2.

## Abbreviations

SNPs: Single nucleotide polymorphisms
eQTL: Expression quantitative trait loci
GWASs: Genome-wide association studies
PGC: The Psychiatric Genomics consortium
GLGC: The Global Lipids Genetics consortium
TWAS: Transcriptome-wide association study
LD: Linkage disequilibrium
SKAT: Sequence kernel association test
SKATO: Optimal SKAT
ACAT: Aggregated Cauchy association test
GATES: Gene-based association test that uses extended Simes procedure
MLP: Multiple linear regression
FLM: Functional multiple linear regression model
MAF: Minor allele frequency
PVE: The phenotypic variance explained
ADHD: Attention-deficit/hyperactivity disorder
ASD: Autism spectrum disorder
BIP: Bipolar disorder
CU: Cannabis use
MDD: Major depression disorder
SCZ: Schizophrenia
HDL: High-density-lipoprotein cholesterol
LDL: Low-density-lipoprotein cholesterol
TC: Total cholesterol
TG: Triglyceride
IBD: Inflammatory bowel disease
UC: Ulcerative colitis
CD: Crohn’s disease
SLE: Systemic lupus erythematosus
PBC: Primary biliary cirrhosis
PSC: Primary sclerosing cholangitis
RA: Rheumatoid arthritis
MS: Multiple sclerosis
OST: Osteoarthritis
