# Supplementary material for: A comprehensive comparison of multilocus association methods with summary statistics in genome-wide association studies
Source: BMC Bioinformatics. 2022 Aug 30;23:359. doi: 10.1186/s12859-022-04897-3 (PMC9429742; doi:10.1186/s12859-022-04897-3)
Supplement: Supplementary file 1 — Additional file 1. Various gene-based association analysis methods. [file 12859_2022_4897_MOESM1_ESM.docx]

**Supplementary Note**

## Notation definition

Consider a local genetic region (a gene or a pathway) with summary statistics of cis-SNPs available from large-scale genome-wide studies (GWAS) of a phenotype under investigation. Let ***Z***=(*Z*_1_, …, *Z_M_*)*^T^* be a vector of marginal *Z*-scores of SNPs located within that region, ***β***=(*β*_1_, …, *β_M_*)*^T^* be a vector of marginal effect sizes, ***P***=(*P*_1_, …, *P _M_*)*^T^* be a vector of corresponding *P* values. In addition, let *n* be the sample size of the original GWAS, **R** be the linkage disequilibrium (LD) matrix of these SNPs, which is often calculated with genotypes of ancestry-matched individuals in a reference panel such as the 1000 Genomes Project [[1](#_ENREF_1)], **1***_M_*=(1, …, 1)*^T^* be a vector with all the elements of one, **V**=diag(1/se(*β*)) with se(*β*) the standard error of the marginal effect size, ***P****_x_*=(*P*_(1)_, …, *P*(*_M_*))*^T^* the ascending (or ordered) *P* values, ***w***=(*w*_1_, …, *w_M_*)*^T^* the non-negative weights for test statistics of individual SNPs with $\sum_{i = 1}^{M} w_{k}=1$, and **W**=diag(***w***) the weight matrix.

## Various gene-based association analysis methods

### Multiple linear regression

By treating these genotype influences as fixed effects, the test statistic for multiple linear regression (MLR) for examining *H*_0_: ***β***=0 is defined as

$$F^{\text{MLR}} = \frac{\mathbf{U}^{2}/M}{(1 - \mathbf{U}^{2})/(n - M - 1)}$$

where $\mathbf{U}^{2} = \mathbf{Z}^{T}\text{(}WRW\text{)}^{-1}\mathbf{Z}/n$. The *P* value is obtained based on an *F* distribution with *M* and *n*-*M*-1 as its degrees of freedom [[2](#_ENREF_2), [3](#_ENREF_3)].

### Functional multiple linear regression model

Unlike MLR which includes all analyzed SNPs when testing the joint effect of these genetic variants collectively, the functional multiple linear regression model (FLM) [[4](#_ENREF_4)] attempts to reduce the number of SNPs in the model by generating an orthogonal basis set of *h*<*M* elements. For FLM, these elements are the *h* basis functions, through which the genotypes and their effects are presented as continuous functions. For an incomplete regression model, *U*^2^ is calculated as

$$\mathbf{U}^{2} = \frac{\mathbf{Z}^{T}VWC(C^{\top}WVRVWC)^{\text{-1}}C^{T}WV\mathbf{Z}}{n}$$

where *C* is an *M*×*h* matrix. For FLM, each element *c_ij_* of *C* represents the *j*^th^ basis association analysis by using the same *F* statistic constructed for MLR.

### Higher criticism test

The basic idea of higher criticism test (HC) was first proposed by Tukey, it was further developed by [Donoho and Jin](#_ENREF_7) [[5](#_ENREF_5)] for summary statistics in the setting where under the alternative the marginal test statistics come from a mixture of normal random variables, as well as by [Arias-Castro et al](#_ENREF_1) [[6](#_ENREF_6)] in the regression setting. Because the ***Z*** scores are correlated, [Hall and Jin](#_ENREF_13) [[7](#_ENREF_7)] designed a higher criticism test based on the transformed ***Z***^∗^. To do so, let

$$S^{*}(t) = \sum_{j = 1}^{M} \mathbf{1}_{\{Z_{(j)}^{*} \geq t\}}$$

Under *H*_0_, , where $2\bar{\Phi}(t) = 1 - \Phi(t)$ is the survival function of the normal distribution. The higher criticism test statistic is thus defined as

$$\text{HC} = \sup_{t \geq t_{0}}\left\{\frac{S^{\text{*}}(t) - 2M\bar{\Phi}(t)}{\sqrt{2M\bar{\Phi}(t)\left[1 - 2M\bar{\Phi}(t)\right]}}\right\}$$

### Generalized higher criticism test

Following $S^{\text{*}}(t)$ in , the generalized higher criticism test (GHC) statistic is defined as [[8](#_ENREF_8)]

$$\text{GHC} = \sup_{t \geq t_{0}} \left\{\frac{S^{*}(t) - 2M\bar{\Phi}(t)}{\sqrt{\widehat{\mathrm{var}}(S^{*}(t))}}\right\}.$$

### Berk-Jones test

The Berk-Jones (BJ) test first defines the significance thresholding function

$$S(t) = \sum_{j = 1}^{M} \mathbf{I}(Z_{(i)} \geq t)$$

where *Z*(*_i_*) is the *i*^th^ smallest *Z* statistics, *t* is the given threshold. Under the null, for a fixed *t* value *S*(*t*) has the binomial distribution Binomial(*d*, *π*) with $\pi=2\bar{\Phi}(t)$. This observation motivates the Berk-Jones statistic [[9](#_ENREF_9)], which is written as

$$\begin{matrix} \text{BJ}_{M} = \max_{1\leq j \leq M/2} \log\left[\frac{\Pr\{S(Z_{(M - j + 1)}) = j|E(Z) = \hat{\mu}_{j, M}\cdot\mathbf{1}_{M}\}}{\Pr\{S(Z_{(M - j + 1)}) = j|E(Z) = 0\cdot\mathbf{1}_{M}\}}\right] \\ \times\mathbf{I}\left\{2\bar{\Phi}(Z_{(M - j + 1)}) < \frac{j}{M}\right\} \end{matrix}$$

where $\hat{\mu}_{j, M} > 0$ solves the following equation

$$j/M = 1 - \{\Phi(Z_{(M - j + 1)} - \hat{\mu}_{j, M}) - \Phi(-Z_{(M - j + 1)} - \hat{\mu}_{j, M})\}$$

### Generalized Berk-Jones test

When SNPs in a gene are in LD (which is the often case), *S*(*t*) in no longer has a binomial distribution, and the Berk-Jones statistic can lose much of its power in finite samples. The generalized Berk-Jones (GBJ) incorporates the additional correlation information by explicitly conditioning on **R** [[10](#_ENREF_10)]

$$\begin{matrix} \text{GBJ}_{M} = \max_{1\leq j \leq M/2} \log\left[\frac{\Pr\{S(Z_{(M - j + 1)}) = j|E(\mathbf{Z}) = \hat{\mu}_{j, M}\cdot\mathbf{1}_{M}, \mathrm{cov}(Z) = \mathbf{R}\}}{\Pr\{S(Z_{(M - j + 1)}) = j|E(\mathbf{Z}) = 0\cdot\mathbf{1}_{M}, \mathrm{cov}(Z) = \mathbf{R}\}}\right] \\ \times\mathbf{I}\left\{2\bar{\Phi}(Z_{(M - j +1)}) < \frac{j}{M}\right\} \end{matrix}$$

GBJ is still the maximum of a set of likelihood-ratio typed tests, but gains notable power over BJ by accounting for the correlation among SNPs.

### Decorrelation by orthogonal transformation

Like SKAT/QT, the decorrelation by orthogonal transformation (DOT) method uses a symmetric orthogonal transformation of the vector of statistics ***Y*** to a new vector ***X*** [[11](#_ENREF_11)], with the new statistic based on the sum of elements of ***X***, $\text{DOT}=\sum_{i=1}^{M} X_{i}^{2}$. The orthogonal transformation is defined as follows. Let $\mathbf{D}=\left(\frac{1}{\sqrt{\text{λ}}}\mathbf{I}\right)$ and define ***X***=**H*Y*** with **H**=**EDE***^T^*. The squared values, $X_{i}^{2}$, are one degree of freedom independent chi-square variable; thus, DOT=***X****^T^****X*** is a chi-square random variable with *M* degrees of freedom and non-centrality value of

$$\text{γ}_{c} = \sum_{i = 1}^{M} \text{γ}_{i} = \mu_{Y}^{T}\mathbf{R}^{-1}\mu_{Y} = (\mathbf{H}\mu_{Y})^{T}(\mathbf{H}\mu_{Y})$$

The cumulative distribution of the new test statistic compared with the observed value *t*_0_ is thus

$$\Pr(X^{T}X > t_{0}) = \Pr(\text{χ}_{M, \text{γ}_{c}}^{2} > t_{0})$$

### Burden test

Under the null hypothesis of no association between those SNPs and the phenotype and within the context of the polygenic architecture of complex phenotype, we have

$$\left\{\begin{matrix} Z \text{\textasciitilde} \text{MVN}(0, R) \\ Z\mathbf{1}_{M} \text{\textasciitilde} N(0, \mathbf{1}_{M}R\mathbf{1}_{M}^{T}) \end{matrix}\right.$$

where MVN(***μ***, **Σ**) denotes the multivariate normal distribution with mean vector ***μ*** and variance-covariance matrix **Σ**; *N*(*μ*, σ^2^) denotes the univariate normal distribution with mean *μ* and variance σ^2^. The relationship shown in is a direct application of the regression with summary statistics (RSS) derived in [[12](#_ENREF_12)]. With these in hand, the statistic of the burden test (BT) is defined as

$$Q^{\text{BT}} = \text{(}\mathbf{Z}1_{M}\text{)}^{2}\text{(}1_{M}R1_{M}^{T}\text{)}^{-1}$$

where *Q*^BT^ asymptotically follows χ2 1, with χ2 1 the chi-squared distribution having one degree of freedom. It is easy to see that the burden test is built by the summation of the *Z*-score statistics and is thus also referred to as the linear sum test [[13](#_ENREF_13), [14](#_ENREF_14)].

### Optimal sequence kernel association test

Following the similar spirit of the optimal sequence kernel association test (SKAT-O) which attempts to combine the strength of burden test and SKAT in rare variant association analysis [[15-17](#_ENREF_15)], we build the following SKAT-O by integrating *Q*^BT^ and *Q*^SKAT^

$$Q^{\text{SKAT-O}} = \min_{\rho\in[0, 1]} P(Q^{\rho})$$

where *ρ* is the unknown weight and *Q^ρ^*=(1-*ρ*)*Q*^SKAT^+*ρQ*^BT^. Then, like *Q*^SKAT^, *Q*^SKAT-O^ also follows a weighted mixture of χ2 1, with the weight λ the eigenvalue of **R** [[14](#_ENREF_14), [15](#_ENREF_15), [18](#_ENREF_18)].

### Sequence kernel association test

Besides the linear form in the burden test shown above, a quadratic statistic (like QT) based on *Z* scores can be similarly constructed

$$Q^{\text{SKAT}} = (\mathbf{Z}1_{M})^{T}(\mathbf{Z}1_{M})$$

where *Q*^SKAT^ follows a weighted sum of χ2 1 distributions with one degree of freedom, with the weight λ being the eigenvalue of $\mathbf{VRV}$ [[19-22](#_ENREF_19)]. *Q*^SKAT^ shares the similar principle of the famous **s**equence kernel association test (SKAT), which was proposed under the context of variance component mixed-effect model for rare variant association testing in sequencing studies [[14](#_ENREF_14), [20-22](#_ENREF_20)]. Therefore, here we refer it as SKAT, which also enjoys the similar principle of QT (see above) [[11](#_ENREF_11)] and is sometimes also referred to as the squared sum test (S2T).

### Simes’s test

Based on the ordered P values, in the Simes’s method, we have [[23](#_ENREF_23)]

$$\Pr\left\{\bigcup_{i}^{M} (P_{(i)} \leq i\alpha)/M\right\} \leq\alpha$$

This suggests a test by rejecting the global null-hypothesis, $H_{0} = \{H_{01}, \ldots, H_{0M}\}$, if *P*(*_i_*)≤*iα*/*M* for at least one *i*. Equivalently, the global *P* value for the SNP-set based association in the Simes’s method is given by $\text{min}\left\{MP_{(i)}/i\right\}$.

### Fisher combination probability

By ignoring the LD among SNPs, the classical Fisher combination probability (FCP) is also used for SNP-based association analysis and can be expressed as [[24](#_ENREF_24)]

$$t = \text{-}2\sum_{i = 1}^{M} \ln P_{i} = \text{-}2\ln\left(\prod_{i = 1}^{M} P_{i}\right)$$

where the significance is compared with its exact χ^2^ distribution with 2*M* degrees of freedom.

### Truncated product method

The test statistic of the truncated product method (TPM) is defined as the product of all the *P* values that do not exceed a given cutoff value (say τ) [[25](#_ENREF_25)]

$$T_{\tau} = \prod_{i = 1}^{M} P_{i}^{I(P_{i} \leq\tau)}$$

where *I*() denotes an indicator function. The distribution of *T* is evaluated by conditioning on the number (i.e., *k*) of *P_i_* which is less than τ

$$\begin{matrix} \Pr(T_{\tau} \leq t_{0}) = \sum_{k = 1}^{M} \Pr(T_{\tau} \leq t_{0}|k)\Pr(k) \\ = \sum_{k = 1}^{M} \left(\begin{matrix} M \\ k \end{matrix}\right)(1 - \tau)^{M - k} \\ \times\left(t_{0}\sum_{s = 0}^{k - 1} \frac{(k\ln\tau- \ln t_{0})^{s}}{s!}I(t_{0} \leq\tau^{k}) + \tau^{k}I(t_{0} > \tau^{k})\right) \end{matrix}$$

where *t*_0_ is the observed value of *T_τ_*.

### *Rank truncated product test*

In the rank truncated product test (RTP) [[26](#_ENREF_26)] follows the similar rationale of TPM; however, the difference is that the number of *P* values to be combined, *k*, is fixed in RTP, rather than changed with the *P* value threshold as done in TPM [[25](#_ENREF_25)]. The test statistic of RTP is given as

$$U_{k} = \text{-}\sum_{i = 1}^{k} \ln P_{(i)}$$

with *k*<*M*. The resulting *P* value can be available from the cumulative distribution probability (CDF) of the product

$$P_{\text{RTP}}(k) = \Pr\left\{U_{k} \leq u_{0}\right\} = 1 \text{-} \int_{0}^{1} G_{k}\left\{\ln\left(\frac{[B_{k + 1}^{\text{-}1}(u)]^{k}}{u_{0}}\right)\right\}du$$

where $B_{k + 1}^{-1}()$ is the inverse CDF of Beta(*k*+1, *M*-*k*), *G_k_*() is the CDF of gamma(*k*, 1). Compared with TPM, RTP leads to an appealing extension, where *k* can be chosen adaptively, to maximize the statistical power.

### Augmented rank truncation

Following RTP , the augmented rank truncation (ART) method exploits the independence of the gamma-distributed summation and *P*(*_k_*) by transforming *P*(*_k_*) to a gamma random variable and adding the result to the sum [[27](#_ENREF_27)]

$$A_{k} = -\ln\{U_{k - 1}\} + (k - 1)\ln\{P_{(k)}\} + G_{\text{λ}}^{-1}\{1 - B_{k}(P_{(k)})\}$$

where $G_{k}^{-1}(\cdot)$ is the inverse CDF of gamma(*k*, 1) and

$$\text{λ} = (k - 1)E\{-\ln(P_{(k)})\} = (k - 1)\left[\Gamma'(M + 1)\Gamma(M + 1) - \Gamma'(k)/\Gamma(k)\right]$$

Г' is the first derivative of a gamma function, and *B_k_*(*x*) is the CDF of Beta(*k*, *M*-*k*+1) distribution evaluated at *x*. Given the observed value *a_k_*, the *P* value is

$$P_{ART} = \Pr(A_{k} \leq a_{k}) = 1- G_{k + \text{λ} - 1}(a_{k})$$

### Adaptive augmented rank truncation

A new and easily implemented version of the theoretical distribution for ART, called adaptive augmented rank truncation (ART-A), was recently proposed [[27](#_ENREF_27)]. ART-A exploits the fact that these ordered *P* values can be represented as functions of the same number of independent uniform random variables. ART-A first approximates the gamma distribution with a large shape parameter by the normal distribution, and then uses the fact that the joint distribution of the partial normal sums follows a multivariate normal distribution

$$U_{i} = \left(\frac{1 - P_{(i)}}{1 - P_{(i-1)}}\right)^{M - i + 1}$$

With *Z*_1_=(1-*P*_(1)_)*^M^*. The *P* value is obtained as

$$P_{\text{ART-A}} = 1 - G_{\sum_{i \text{=} 1}^{k} \text{λ}_{i}}\left(\sum_{i \text{=} 1}^{k} G_{\text{λ}_{i}}^{-1}(1 - U_{i})\right)$$

with λ given in . When λ*_i_* is large, the gamma CDF approaches the standard normal CDF, which motivates the inverse normal transformation. The quantile will be calculated by using λ*_i_*Φ^-1^(1-*U_i_*), as an approximation to $G_{\lambda_{i}}^{-1}(1-U_{i})$ for large *k*. The inverse normal method is useful as the joint distribution of the partial sums can be derived in a standard way to evaluate the *P* value of ART-A. For ART-A, the partial sum is defined as

$$S_{k} = \sum_{i \text{=} 1}^{k} \text{λ}_{i}\Phi^{-1}(1 - U_{i})$$

where Φ^-1^() is the inverse CDF of the standard normal distribution. Then, under the null hypothesis, ***S***=(*S*_1_, *S*_2_, …, *S_k_*)*^T^* follows a multivariate normal distribution MVN(0, Σ) with Σ = **FWF***^T^* and

$$\mathbf{F} = \left[\begin{matrix} 1 & 0 & \cdots& 0 & 0 \\ 1 & 1 & \cdots& 0 & 0 \\ \vdots& \vdots& \ddots& \vdots& \vdots\\ 1 & 1 & \cdots& 1 & 0 \\ 1 & 1 & \cdots& 1 & 1 \end{matrix}\right],\text{ }\mathbf{W} = \left[\begin{matrix} w_{1}^{2} & & & \\ & w_{2}^{2} & & \\ & & \ddots& \\ & & & w_{k}^{2} \end{matrix}\right]$$

The vector ***S*** can be standardized as *T_i_*=*S_i_*/*σ_i_*, where *σ_i_* is the diagonal element of Σ; then ***T***∼MVN(0, **D**), $D_{ij}=\frac{\Sigma_{ij}}{\sqrt{\Sigma_{ii}\Sigma_{jj}}}$. The null distribution of ***T*** is used to evaluate ART-A by using Pr(*S_i_*=*σ_i_*>*s_i_*) and to obtain quantiles (significance thresholds) using commonly available MVN distribution function.

### Gamma method

To combine multiple *P* values, Zaykin et al. [[28](#_ENREF_28)] exploits the feature of the gamma distribution that the summation of gamma-distributed random variables with the same rate still has a gamma distribution. As a result, the gamma method (GM) is defined by

$$U = \sum_{i = 1}^{M} G_{a, 1}^{-1}(1 \text{-} P_{i}), P_{GM} = 1 \text{-} G_{Ma, 1}(U)$$

where $G_{a, 1}^{-1}(1 \text{-} x)$ is the inverse of the cumulative distribution function of gamma(*a*, 1).

### SimpleM

The simpleM method computes a gene-based *P* value by taking the smallest *P* value of SNPs within a gene while correcting for the effective number of independent tests through the Bonferroni correction procedure [[29](#_ENREF_29), [30](#_ENREF_30)]

$$P = 1 \text{-} (1 \text{-} \min\{P_{(1)}, P_{(2)}, \cdots, P_{(M)}\})^{k}$$

where *k* is the effective number of independent tests estimated from the correlation matrix of SNPs using a principal component analysis approach. Generally, simpleM pre-assigns *k* so that the corresponding eigenvalues together explain 99.5% of the variation.

### Gene-based association test that uses extended Simes procedure

The gene-based association test that uses extended Simes procedure (GATES) combines the *P* values of those SNPs into an overall gene-based *P* value by

$$P_{\text{GATES}} = \text{min}_{1\leq i\leq M}(N_{e}P_{(i)}/N_{e(i)})$$

where N_e_ is the effective number of independent *P* values among the *M* genetic variants, P_(i)_ is the *i*^th^ smallest *P* value and N_e(i)_ is the effective number of independent *P* values among the top *i* SNPs. Specifically, N_e_ is estimated by

$$N_{e} = M - \sum_{j=1}^{M} (\text{λ}_{j} - 1)I(\text{λ}_{j} - 1)$$

where λ*_j_* is the *j*^th^ eigenvalue of the *P* value correlation matrix. The computation of N_e(i)_ is similar to that of N_e_; but the eigenvalues are computed for the *P* value correlation matrix of the top *i* SNPs [[30](#_ENREF_30), [31](#_ENREF_31)].

### Harmonic mean *P* values combination

For the harmonic mean *P* values (HMP) combination method [[32](#_ENREF_32)], we have

$$P_{\text{HMP}} = \int_{1/T_{\text{HMP}}}^{\infty} f_{x}(x|\log M + c, \frac{\pi}{2})dx, T_{\text{HMP}} = (\sum_{i = 1}^{M} w_{i})/(\sum_{i = 1}^{M} w_{i}/P_{i})$$

where *f_x_* denotes the Landau distribution probability density function with *c* = 0.874. It has been shown that, under some regular conditions, that the power of our test is asymptotically optimal in a strong sparsity setting, the combined *P* value for HMP is robust against the number of tests, selected weights (e.g., unequal prior weights), and positive dependency between *P* values.

### Aggregated Cauchy association test

For the aggregated Cauchy association test (ACAT) [[33](#_ENREF_33), [34](#_ENREF_34)], we have

$$\begin{matrix} P_{\text{ACAT}} = \frac{1}{2} - \text{arc}\tan\left\{T_{\text{ACAT}}/(\sum_{i = 1}^{M} w_{i})\right\}/\pi\\ T_{\text{ACAT}} = \sum_{i = 1}^{M} w_{i}\tan\left\{(\frac{1}{2} - P_{i})\pi\right\} \end{matrix}$$

## References

1. The 1000 Genomes Project Consortium: **A global reference for human genetic variation**. *Nature* 2015, **526**(7571):68-74.

2. Svishcheva GR, Belonogova NM, Zorkoltseva IV, Kirichenko AV, Axenovich TI: **Gene-based association tests using GWAS summary statistics**. *Bioinformatics* 2019, **35**(19):3701-3708.

3. Chow GC: **Tests of Equality Between Sets of Coefficients in Two Linear Regressions**. *Econometrica* 1960, **28**(3):591-605.

4. Belonogova NM, Svishcheva GR, Wilson JF, Campbell H, Axenovich TI: **Weighted functional linear regression models for gene-based association analysis**. *PLoS One* 2018, **13**(1):e0190486.

5. Donoho D, Jin J: **Higher criticism for detecting sparse heterogeneous mixtures**. *The Annals of Statistics* 2004, **32**(3):962-994, 933.

6. Arias-Castro E, Candès EJ, Plan Y: **Global testing under sparse alternatives: ANOVA, multiple comparisons and the higher criticism**. *The Annals of Statistics* 2011, **39**(5):2533-2556, 2524.

7. Hall P, Jin J: **Innovated higher criticism for detecting sparse signals in correlated noise**. *The Annals of Statistics* 2010, **38**(3):1686-1732, 1647.

8. Barnett I, Mukherjee R, Lin X: **The Generalized Higher Criticism for Testing SNP-Set Effects in Genetic Association Studies**. *Journal of the American Statistical Association* 2017, **112**(517):64-76.

9. Berk RH, Jones DH: **Goodness-of-fit test statistics that dominate the Kolmogorov statistics**. *Zeitschrift für Wahrscheinlichkeitstheorie und Verwandte Gebiete* 1979, **47**(1):47-59.

10. Sun R, Hui S, Bader GD, Lin X, Kraft P: **Powerful gene set analysis in GWAS with the Generalized Berk-Jones statistic**. *PLoS Genet* 2019, **15**(3):e1007530-e1007530.

11. Vsevolozhskaya OA, Shi M, Hu F, Zaykin DV: **DOT: Gene-set analysis by combining decorrelated association statistics**. *PLoS Computational Biology* 2020, **16**(4):e1007819.

12. Zhu X, Stephens M: **Bayesian large-scale multiple regression with summary statistics from genome-wide association studies**. *Annals of Applied Statistics* 2017, **11**(3):1561-1592.

13. Madsen BE, Browning SR: **A groupwise association test for rare mutations using a weighted sum statistic**. *PLoS Genet* 2009, **5**(2):e1000384.

14. Guo B, Wu B: **Statistical methods to detect novel genetic variants using publicly available GWAS summary data**. *Comput Biol Chem* 2018, **74**:76-79.

15. Ionita-Laza I, Lee S, Makarov V, Buxbaum Joseph D, Lin X: **Sequence Kernel Association Tests for the Combined Effect of Rare and Common Variants**. *The American Journal of Human Genetics* 2013, **92**(6):841-853.

16. Lee S, Wu MC, Lin X: **Optimal tests for rare variant effects in sequencing association studies**. *Biostatistics* 2012, **13**(4):762-775.

17. Lee S, Emond MJ, Bamshad MJ, Barnes KC, Rieder MJ, Nickerson DA, Christiani David C, Wurfel Mark M, Lin X: **Optimal Unified Approach for Rare-Variant Association Testing with Application to Small-Sample Case-Control Whole-Exome Sequencing Studies**. *The American Journal of Human Genetics* 2012, **91**(2):224-237.

18. Lee S, Wu MC, Lin X: **Optimal tests for rare variant effects in sequencing association studies**. *Biostatistics* 2012, **13**(4):762-775.

19. Wu MC, Kraft P, Epstein MP, Taylor DM, Chanock SJ, Hunter DJ, Lin X: **Powerful SNP-set analysis for case-control genome-wide association studies**. *Am J Hum Genet* 2010, **86**(6):929-942.

20. Wu MC, Lee S, Cai T, Li Y, Boehnke M, Lin X: **Rare-Variant Association Testing for Sequencing Data with the Sequence Kernel Association Test**. *The American Journal of Human Genetics* 2011, **89**(1):82-93.

21. Zeng P, Zhao Y, Liu J, Liu L, Zhang L, Wang T, Huang S, Chen F: **Likelihood Ratio Tests in Rare Variant Detection for Continuous Phenotypes**. *Annals of Human Genetics* 2014, **78**(5):320-332.

22. Zeng P, Zhao Y, Li H, Wang T, Chen F: **Permutation-based variance component test in generalized linear mixed model with application to multilocus genetic association study**. *BMC Medical Research Methodology* 2015, **15**:37.

23. Simes J: **An Improved Bonferroni Procedure for Multiple Tests of Significance**. *Biometrika* 1986, **73**:751-754.

24. Fisher RA: **Statistical Methods for Research Workers**. In: *Breakthroughs in Statistics: Methodology and Distribution.* Edited by Kotz S, Johnson NL. New York, NY: Springer New York; 1992: 66-70.

25. Zaykin DV, Zhivotovsky LA, Westfall PH, Weir BS: **Truncated product method for combining P-values**. *Genet Epidemiol* 2002, **22**(2):170-185.

26. Dudbridge F, Koeleman BPC: **Rank truncated product of P-values, with application to genomewide association scans**. *Genet Epidemiol* 2003, **25**(4):360-366.

27. Vsevolozhskaya OA, Hu F, Zaykin DV: **Detecting Weak Signals by Combining Small P-Values in Genetic Association Studies**. *Frontiers in Genetics* 2019, **10**(1051).

28. Zaykin DV, Zhivotovsky LA, Czika W, Shao S, Wolfinger RD: **Combining p-values in large-scale genomics experiments**. *Pharmaceutical Statistics* 2007, **6**(3):217-226.

29. Gao X, Starmer J, Martin ER: **A multiple testing correction method for genetic association studies using correlated single nucleotide polymorphisms**. *Genet Epidemiol* 2008, **32**(4):361-369.

30. Wang M, Huang J, Liu Y, Ma L, Potash JB, Han S: **COMBAT: A Combined Association Test for Genes Using Summary Statistics**. *Genetics* 2017, **207**(3):883.

31. Li M, Gui H, Kwan J, Sham P: **GATES: a rapid and powerful gene-based association test using extended Simes procedure**. *American journal of human genetics* 2011, **88**(3):283-293.

32. Wilson D: **The harmonic mean p-value for combining dependent tests**. *Proceedings of the National Academy of Sciences of the United States of America* 2019, **116**(4):1195-1200.

33. Liu Y, Chen S, Li Z, Morrison AC, Boerwinkle E, Lin X: **ACAT: A Fast and Powerful p Value Combination Method for Rare-Variant Analysis in Sequencing Studies**. *The American Journal of Human Genetics* 2019, **104**(3):410-421.

34. Li Z, Li X, Liu Y, Shen J, Chen H, Zhou H, Morrison AC, Boerwinkle E, Lin X: **Dynamic Scan Procedure for Detecting Rare-Variant Association Regions in Whole-Genome Sequencing Studies**. *The American Journal of Human Genetics* 2019, **104**(5):802-814.
