# Supplementary material for: A comprehensive comparison of multilocus association methods with summary statistics in genome-wide association studies
Source: BMC Bioinformatics. 2022 Aug 30;23:359. doi: 10.1186/s12859-022-04897-3 (PMC9429742; doi:10.1186/s12859-022-04897-3)
Supplement: Supplementary file 2 — Additional file 2. Figure S1. Estimated power for the seven SNP-set methods under the polygenic case (A) and the mixed case (B) with a significance level α of 10−5. Here, PVE = 0.3%, 0.5% or 1% at the right side, the number of causal SNPs (prop) = 0.05, 0.20 or 0.50 or the distribution of effect size including double, normal and t on the top, the number of the total analyzed SNPs = 50, 200 or 500 on the x-axis. The power was estimated across 103 replications. Figure S2. Rank of power for the seven SNP-set methods under the sparse case (A), the polygenic case (B), and the mixed case (C) with a significance level α of 10−5. The number in each cell represents − log(P). normal: SNP effect sizes followed a standard normal distribution; double: SNP effect sizes followed a standard double exponential distribution; t: SNP effect sizes followed a standard t-distribution. Figure S3. Estimated power for the seven SNP-set methods in the case of rare variant association study under the polygenic case (A) and the mixed case (B) with a significance level α of 10−5. Here, PVE = 0.3%, 0.5% or 1% at the right side, the number of causal SNPs (prop) = 0.05, 0.20 or 0.50 or the distribution of effect size including double, normal and t on the top, the number of the total analyzed SNPs = 50, 200 or 500 on the x-axis. The power was estimated across 103 replications. Figure S4. Upset plot to illustrate the number of identified genes shared across seven SNP-set methods for six psychiatric disorders. Figure S5. A Enrichment of differentially expressed pleiotropic genes associated with the six psychiatric disorders in terms of expression level across the 54 GTEx tissues. P values are shown in the y-axis with a scale of − log10. The bar in red represents significant enrichment after Bonferroni’s adjustment for multiple hypothesis tests; B Top 10 significant types of pathways in terms of the GO and KEGG enrichment analyses. BP Biological process, CC Cellular component, MF Molecular function. Fi [file 12859_2022_4897_MOESM2_ESM.docx]

# Supplementary Results


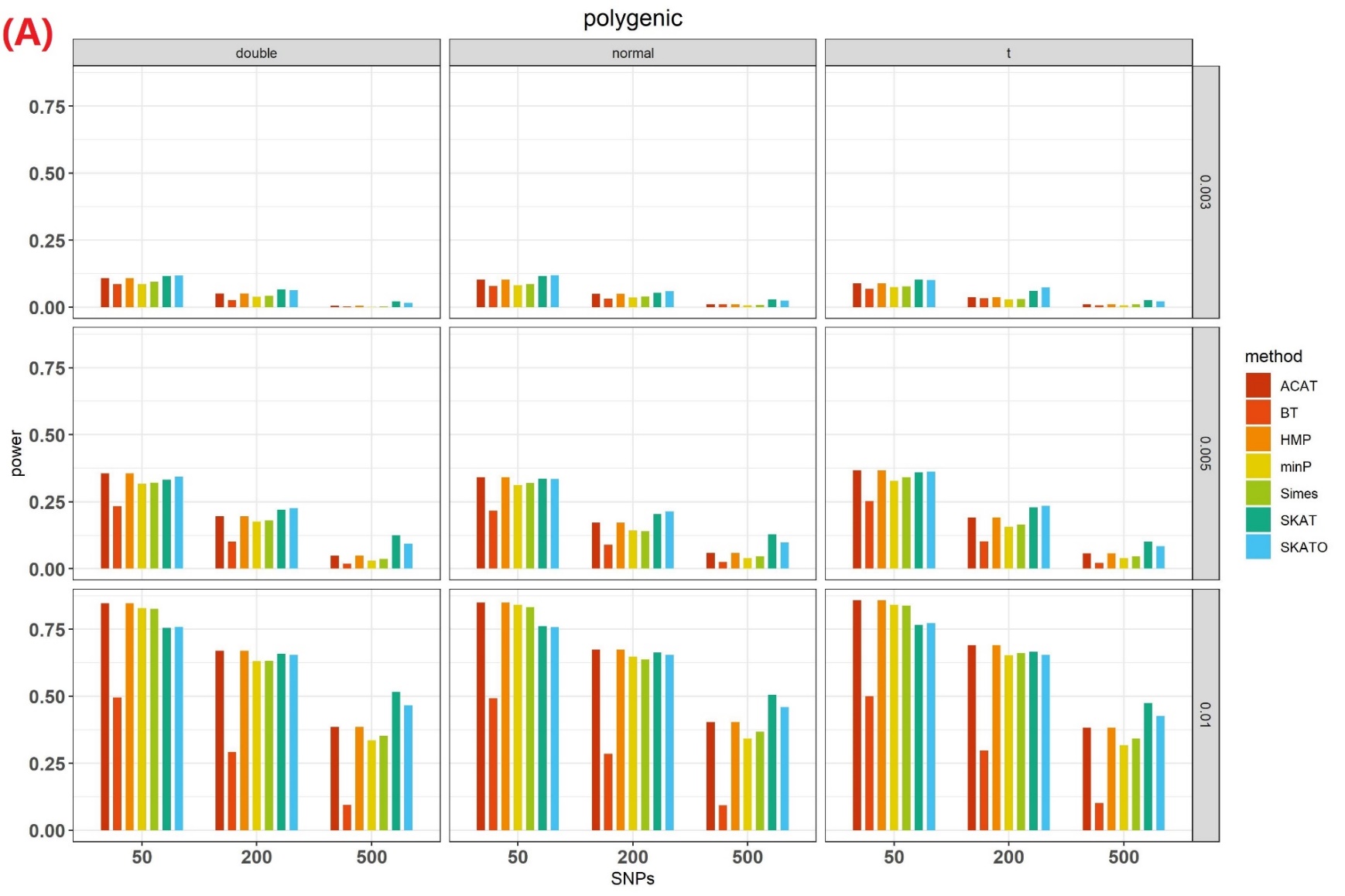

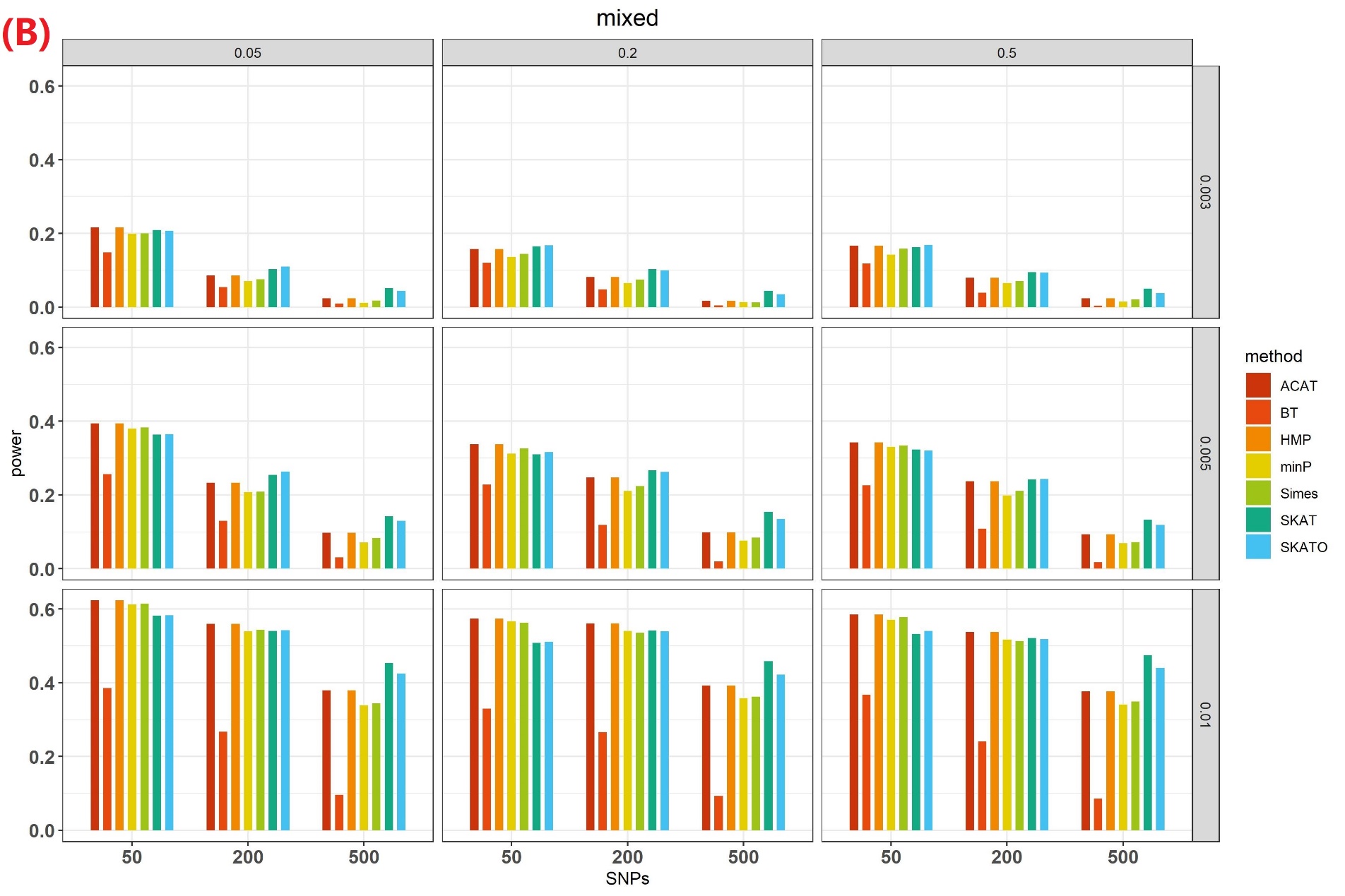


## Figure S1. Estimated power for the seven SNP-set methods under the polygenic case (A) and the mixed case (B) with a significance level α of 10^-5^. Here, PVE=0.3%, 0.5% or 1% at the right side, the number of causal SNPs (prop)=0.05, 0.20 or 0.50 or the distribution of effect size including double, normal and *t* on the top, the number of the total analyzed SNPs=50, 200 or 500 on the x-axis. The power was estimated across 10^3^ replications.


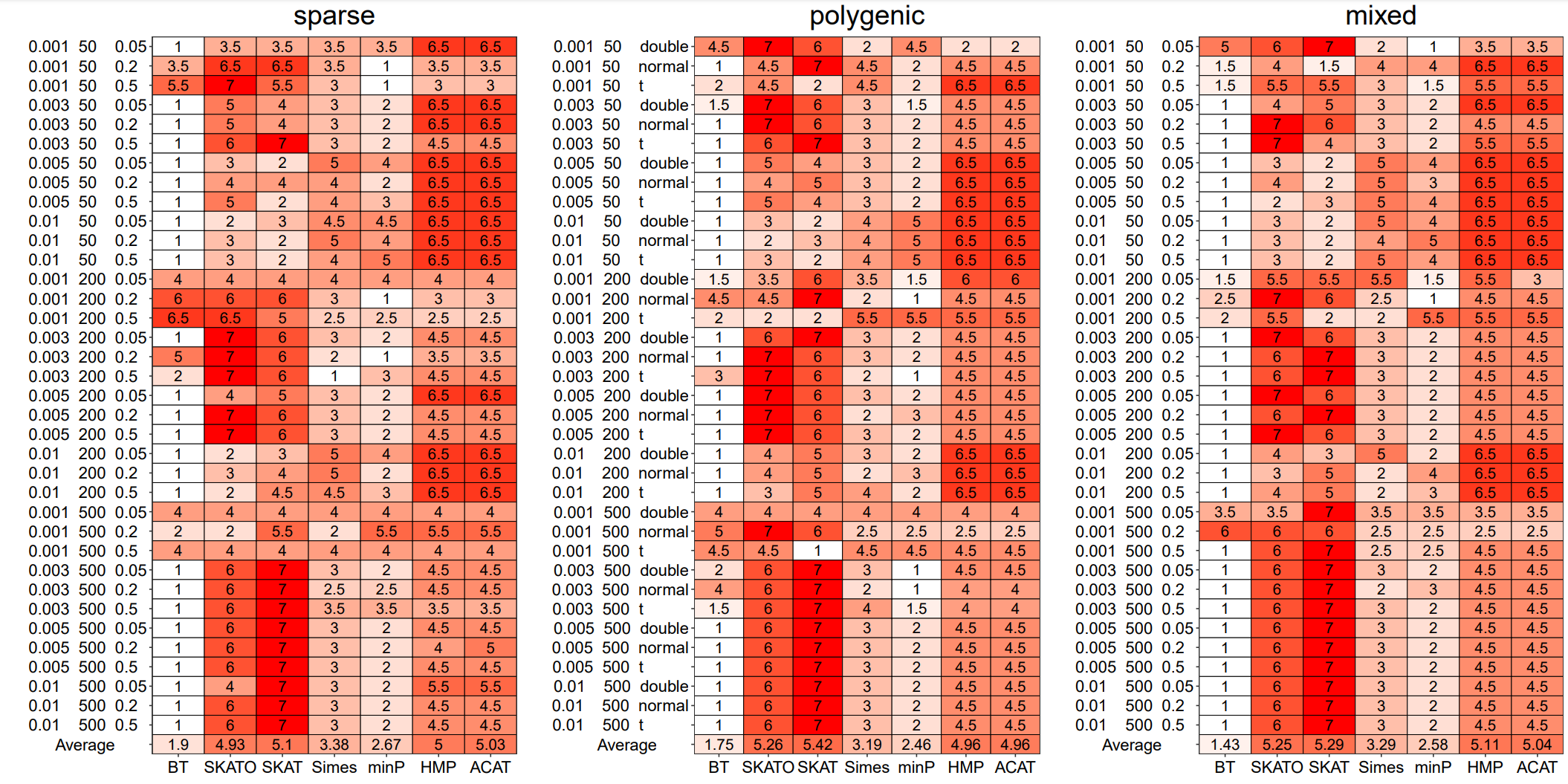


## Figure S2. Rank of power for the seven SNP-set methods under the sparse case (A), the polygenic case (B), and the mixed case (C) with a significance level α of 10^-5^. The number in each cell represents -log(P). normal: SNP effect sizes followed a standard normal distribution; double: SNP effect sizes followed a standard double exponential distribution; t: SNP effect sizes followed a standard t-distribution.


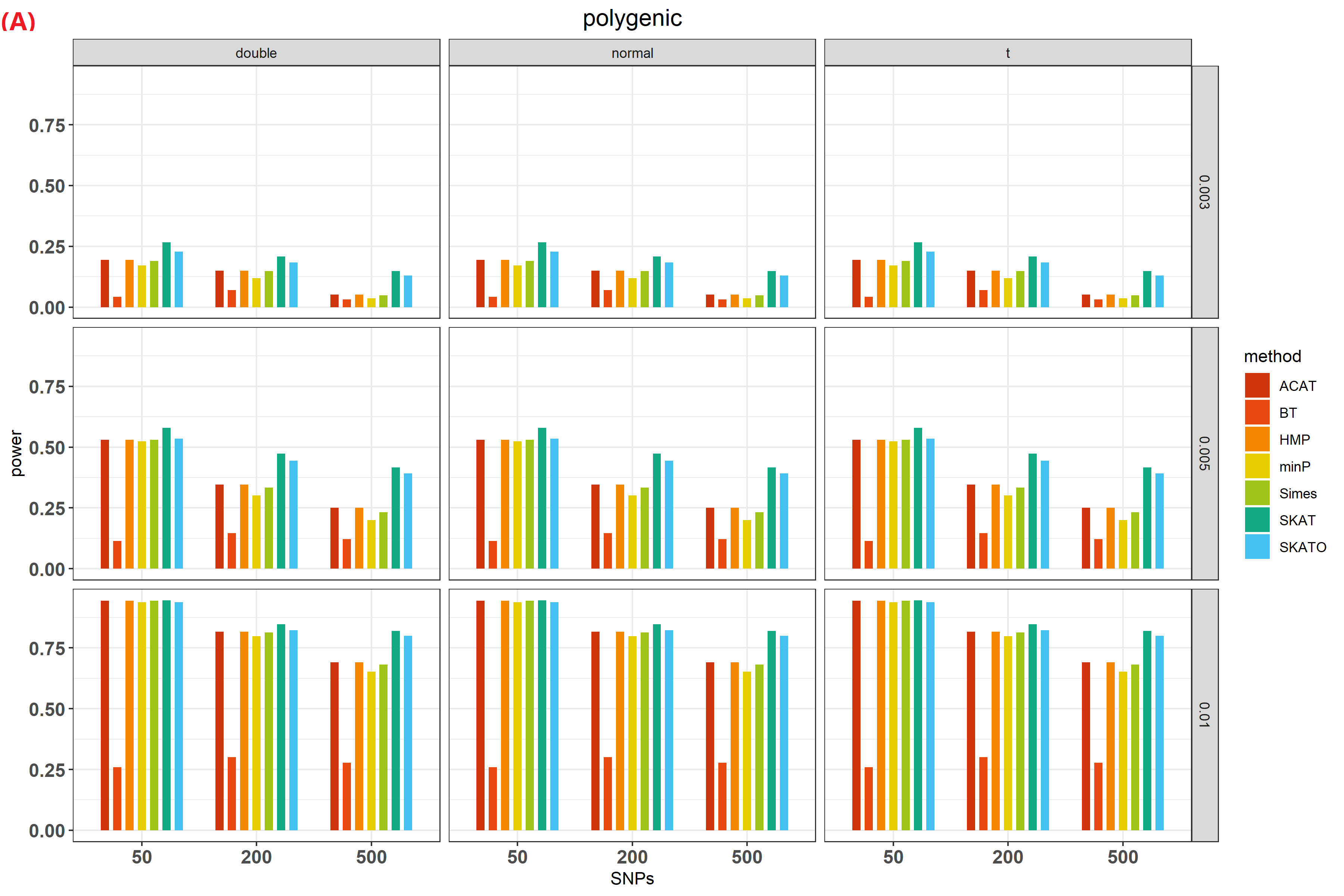

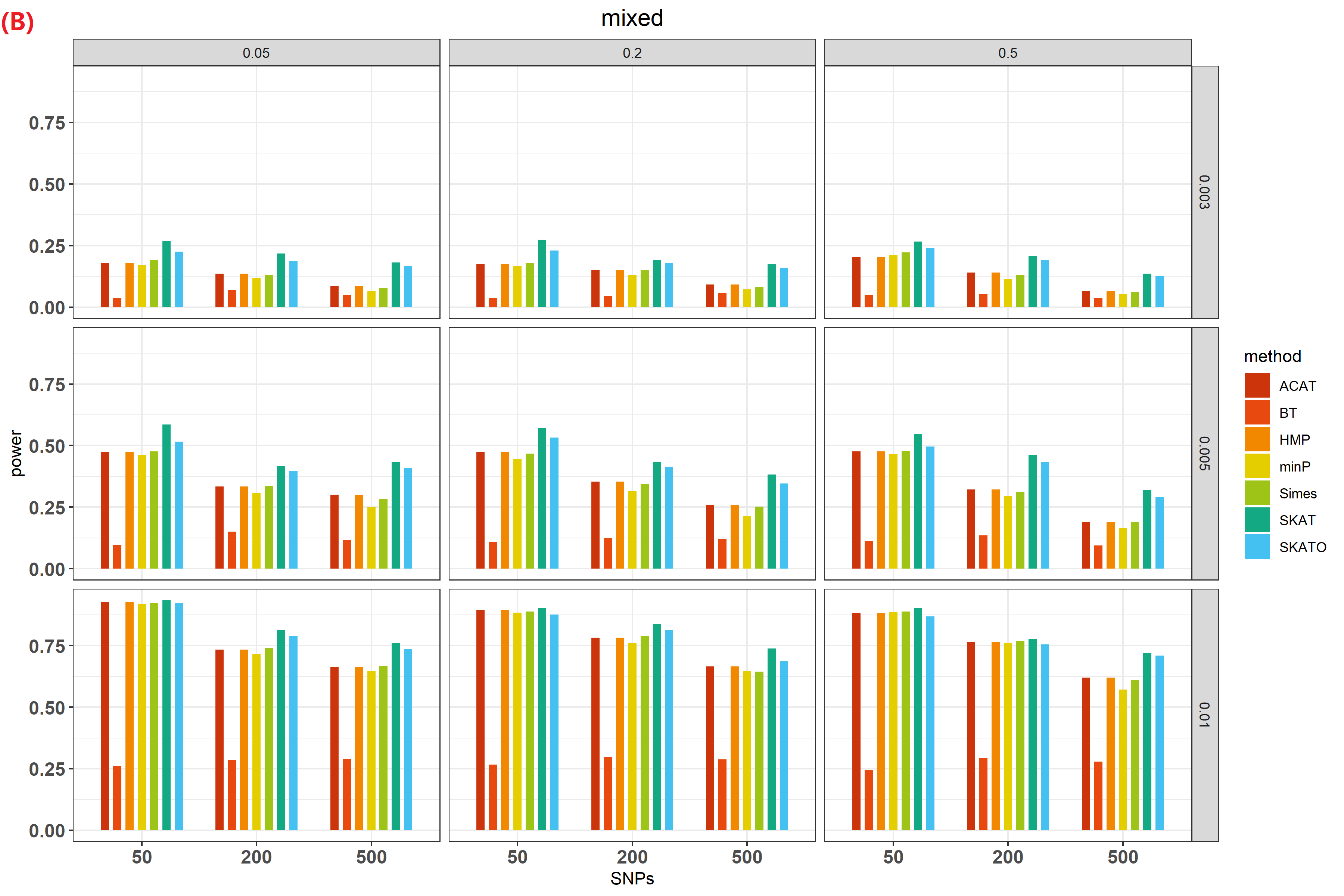


## Figure S3. Estimated power for the seven SNP-set methods in the case of rare variant association study under the polygenic case (A) and the mixed case (B) with a significance level α of 10^-5^. Here, PVE=0.3%, 0.5% or 1% at the right side, the number of causal SNPs (prop)=0.05, 0.20 or 0.50 or the distribution of effect size including double, normal and *t* on the top, the number of the total analyzed SNPs=50, 200 or 500 on the x-axis. The power was estimated across 10^3^ replications.


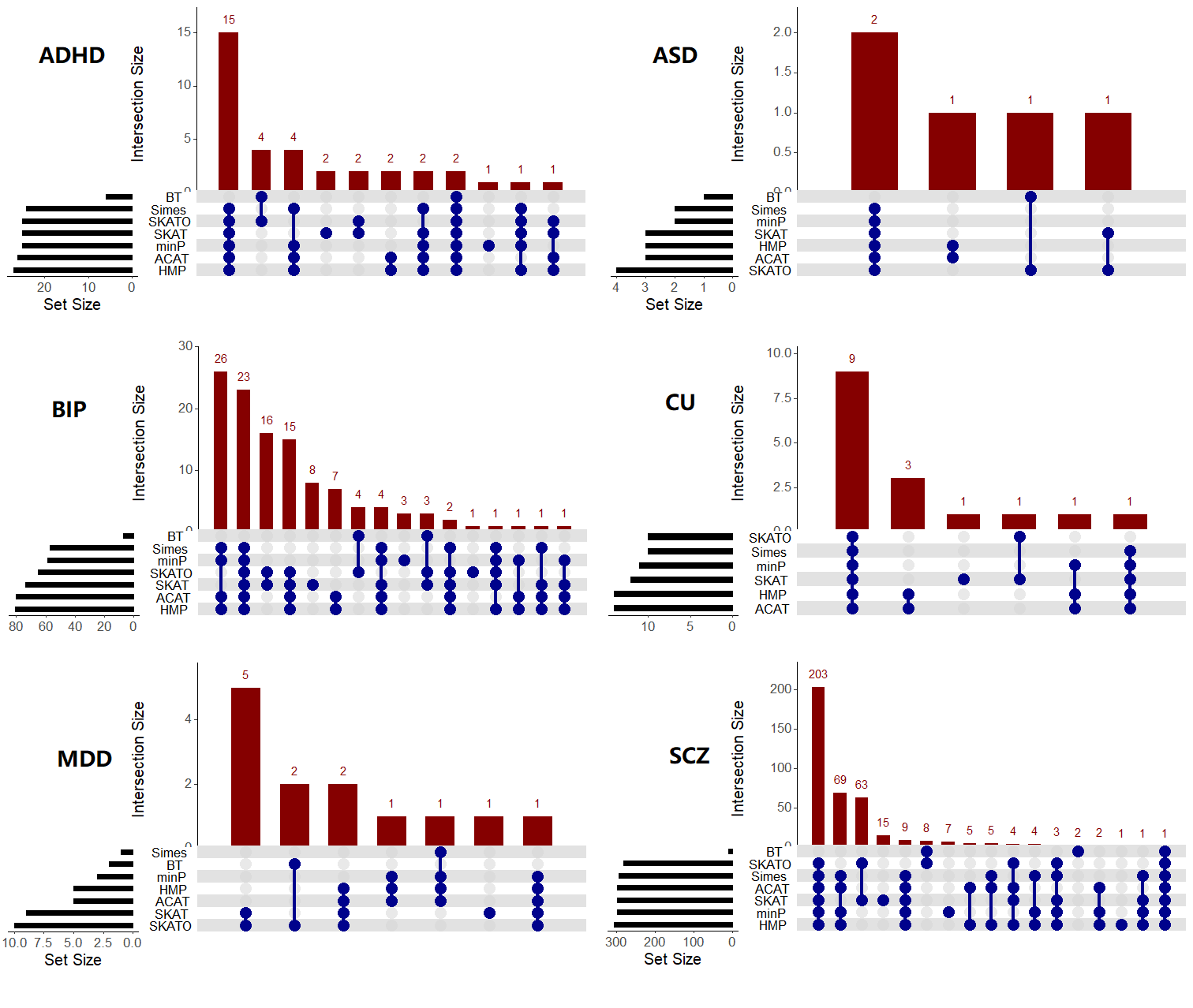


## Figure S4. Upset plot to illustrate the number of identified genes shared across seven SNP-set methods for six psychiatric disorders.


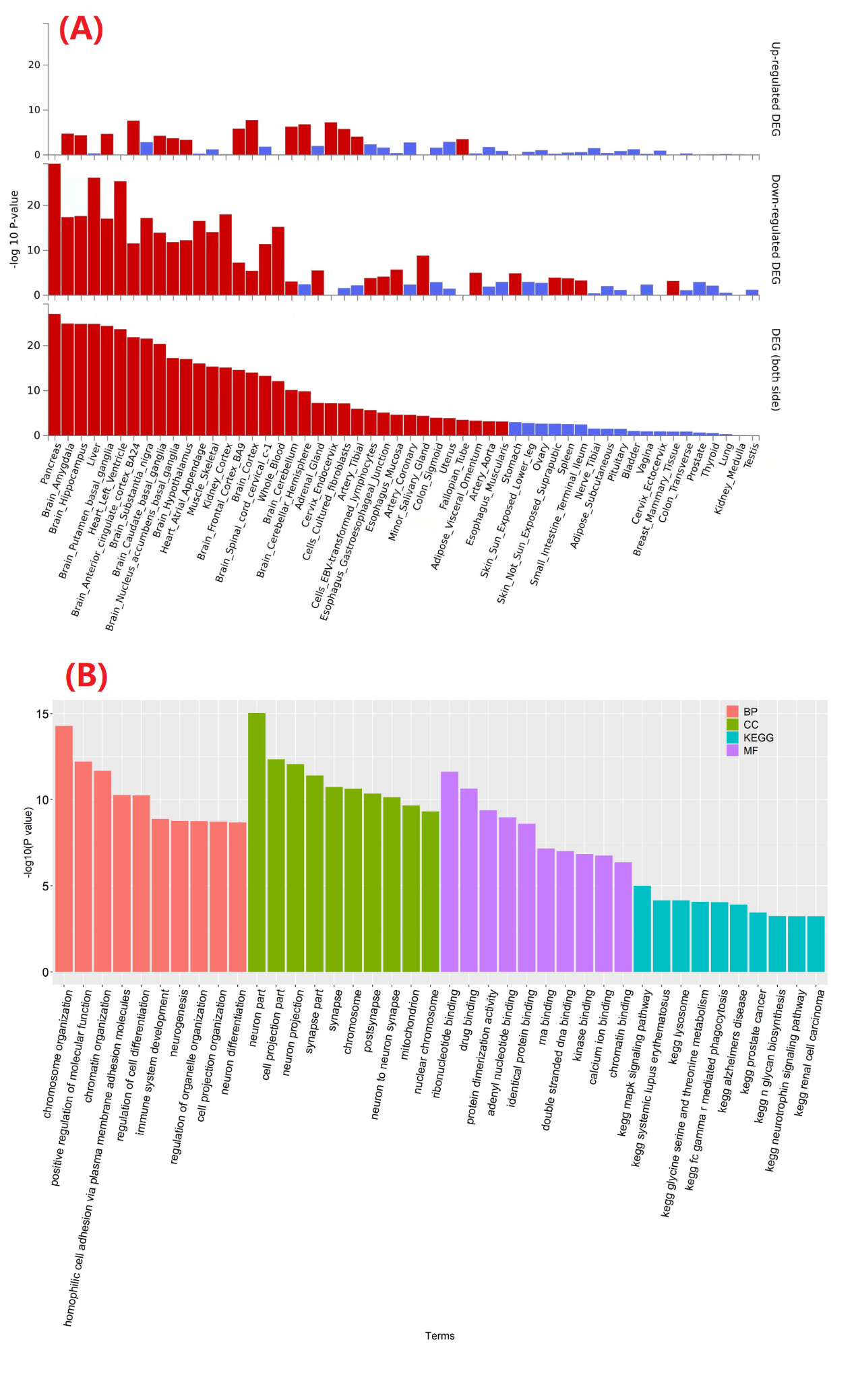


## Figure S5. (A) Enrichment of differentially expressed pleiotropic genes associated with the six psychiatric disorders in terms of expression level across the 54 GTEx tissues. *P* values are shown in the y-axis with a scale of -log10. The bar in red represents significant enrichment after Bonferroni’s adjustment for multiple hypothesis tests; (B) Top 10 significant types of pathways in terms of the GO and KEGG enrichment analyses. BP: biological process; CC: cellular component; MF: molecular function.


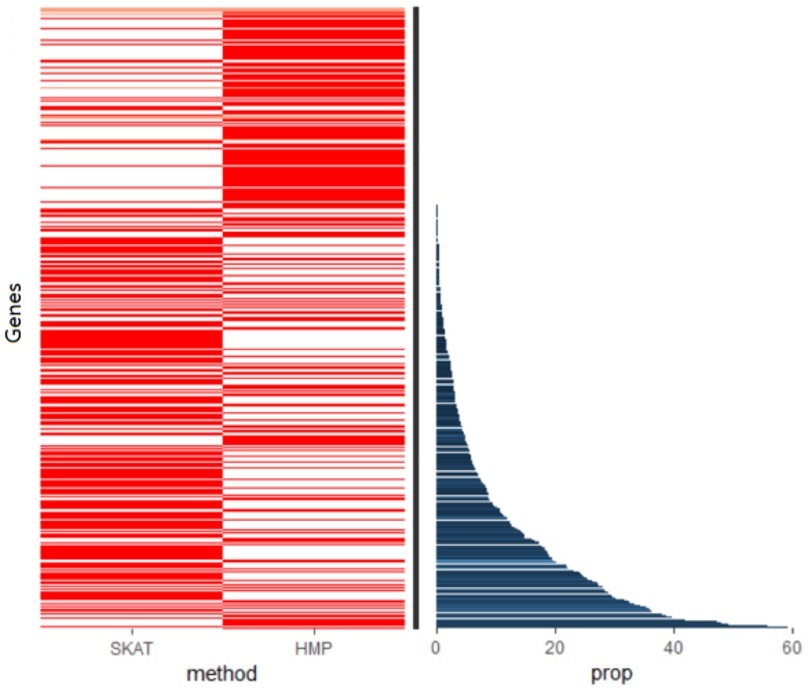


## Figure S6. Bar plot of 531 unique genes associated with the six psychiatric disorders. The red color in the heatmap represents the rank of *P* values of SKAT and HMP; prop: the proportion of significant cis-SNPs (*P*<5×10^-8^) within each associated gene.


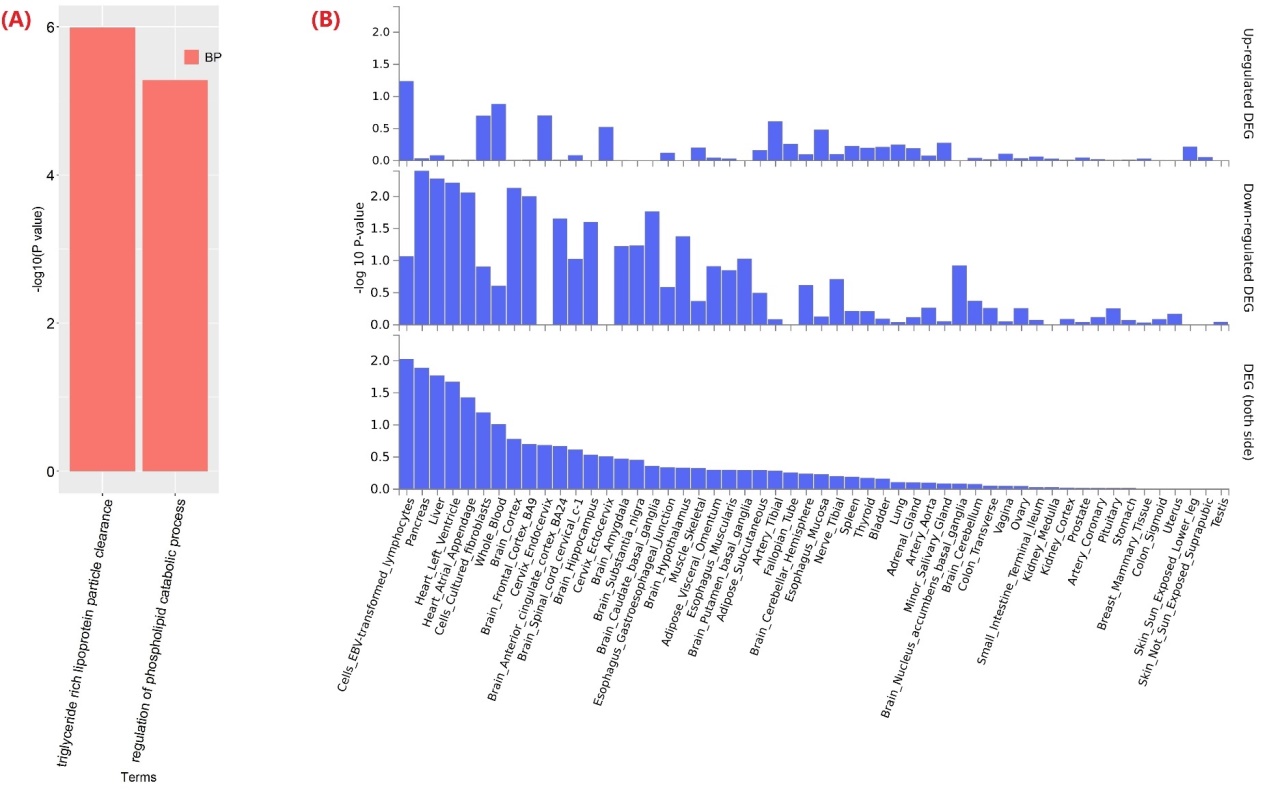


## Figure S7. (A) Enrichment of differentially expressed pleiotropic genes related to the four plasma lipid traits in terms of expression level across the 54 GTEx tissues. *P* values are shown in the y-axis with a scale of -log10. The bar in red represents significant enrichment after Bonferroni’s adjustment for multiple hypothesis tests; (B) Top 10 significant types of pathways in terms of the GO and KEGG enrichment analyses. BP: biological process; CC: cellular component; MF: molecular function.


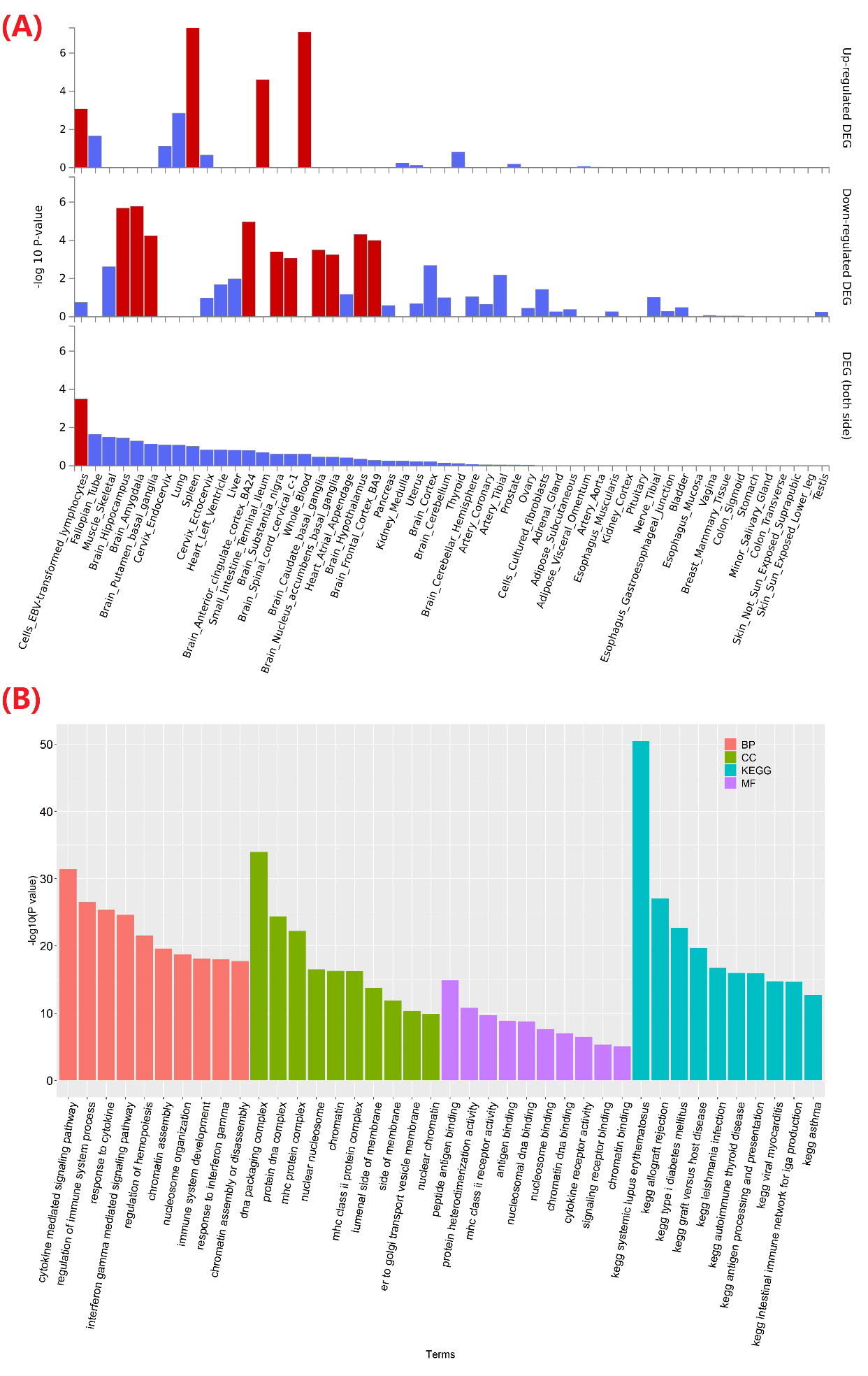


## Figure S8. (A) Enrichment of differentially expressed pleiotropic genes associated with the nine immune-related diseases in terms of expression level across the 54 GTEx tissues. *P* values are shown in the y-axis with a scale of -log10. The bar in red represents significant enrichment after Bonferroni’s adjustment for multiple hypothesis tests; (B) Top 10 significant types of pathways in terms of the GO and KEGG enrichment analyses. BP: biological process; CC: cellular component; MF: molecular function.

## Table S1. Summary information of the six psychiatric disorders, four plasma lipid traits and nine immune-related diseases

| phenotype | *N* | *M* | Ref |
| --- | --- | --- | --- |
| six psychiatric disorders | | | |
| ADHD | 53,293 | 6,414,003 | [[1](#_ENREF_1)] |
| ASD | 46,350 | 7,076,650 | [[2](#_ENREF_2)] |
| BIP | 51,710 | 7,479,414 | [[3](#_ENREF_3)] |
| CU | 184,765 | 6,908,164 | [[4](#_ENREF_4)] |
| MDD | 480,359 | 5,372,902 | [[5](#_ENREF_5)] |
| SCZ | 77,096 | 7,684,282 | [[6](#_ENREF_6)] |
| four plasma lipid traits | | | |
| HDL | 282,937 | 242,276 | [[7](#_ENREF_7)] |
| LDL | 264,019 | 242,246 | [[7](#_ENREF_7)] |
| TC | 285,961 | 242,279 | [[7](#_ENREF_7)] |
| TG | 273,014 | 242,267 | [[7](#_ENREF_7)] |
| nine immune-related diseases | | | |
| IBD | 34,652 | 9,277,320 | [[8](#_ENREF_8)] |
| UC | 27,432 | 9,132,943 | [[8](#_ENREF_8)] |
| CD | 20,883 | 9,116,333 | [[8](#_ENREF_8)] |
| SLE | 23,210 | 7,737,498 | [[9](#_ENREF_9)] |
| PBC | 13,239 | 1,134,225 | [[10](#_ENREF_10)] |
| PSC | 24,751 | 6,614,820 | [[11](#_ENREF_11)] |
| RA | 37,681 | 5,646,048 | [[12](#_ENREF_12)] |
| MS | 68,379 | 7,930,010 | [[13](#_ENREF_13)] |
| OST | 63,608 | 16,499,239 | [[14](#_ENREF_14)] |

**Note:** *N* is the sample size of original GWASs; *M* is the total number of SNPs across the whole genome; ADHD: attention-deficit/hyperactivity disorder; ASD: autism spectrum disorder; BIP: bipolar disorder; CU: cannabis use; MDD: major depression disorder; SCZ: schizophrenia; HDL: high-density-lipoprotein cholesterol; LDL: low-density-lipoprotein cholesterol; TG: triglycerides; TC: total cholesterol; IBD: inflammatory bowel disease; UC: ulcerative colitis; CD: Crohn’s disease; SLE: systemic lupus erythematosus; PBC: primary biliary cirrhosis; PSC: primary sclerosing cholangitis; RA: rheumatoid arthritis; MS: multiple sclerosis; and OST: osteoarthritis.

## Table S2. Ratio between the empirical type I error and the given significance level estimated over 10^5^ simulations under rare variants.

| **method** | **significance level α** | | | |  | **performance of type I error control** | | |
| --- | --- | --- | --- | --- | --- | --- | --- | --- |
|  | **0.05** | **0.01** | **0.001** | **average** |  | **inflated** | **well-controlled** | **conservative** |
| MLR | 0.00 | 0.00 | 0.00 | 0.00 |  |  |  | √ |
| FLM | 0.00 | 0.00 | 0.00 | 0.00 |  |  |  | √ |
| HC | 1.55 | 1.78 | 1.20 | 1.51 |  | √ |  |  |
| GHC | 1.49 | 1.72 | 1.20 | 1.47 |  | √ |  |  |
| BJ | 2.02 | 3.78 | 11.00 | 5.60 |  | √ |  |  |
| GBJ | 1.76 | 2.62 | 3.80 | 2.73 |  | √ |  |  |
| DOT | 0.40 | 0.37 | 0.41 | 0.40 |  |  |  | √ |
| BT | 1.08 | 1.02 | 1.00 | 1.03 |  |  | √ |  |
| SKAT-O | 0.94 | 1.06 | 1.00 | 1.00 |  |  | √ |  |
| SKAT | 0.90 | 1.00 | 0.60 | 0.83 |  |  | √ |  |
| Simes | 0.93 | 0.98 | 1.20 | 1.04 |  |  | √ |  |
| FCP | 2.46 | 6.02 | 24.20 | 10.89 |  | √ |  |  |
| TPM | 1.89 | 5.12 | 25.40 | 10.80 |  | √ |  |  |
| RTP | 2.22 | 5.50 | 22.40 | 10.04 |  | √ |  |  |
| minP | 0.98 | 1.00 | 1.40 | 1.13 |  |  | √ |  |
| ART | 2.32 | 5.78 | 23.40 | 10.50 |  | √ |  |  |
| ART-A | 1.58 | 2.91 | 7.77 | 4.09 |  | √ |  |  |
| GM | 1.46 | 3.20 | 12.80 | 5.82 |  | √ |  |  |
| SimpleM | 0.40 | 0.41 | 0.41 | 0.41 |  |  |  | √ |
| GATES | 0.49 | 0.48 | 0.20 | 0.39 |  | √ |  |  |
| HMP | 0.95 | 1.06 | 1.20 | 1.07 |  |  | √ |  |
| ACAT | 0.94 | 0.96 | 1.20 | 1.03 |  |  | √ |  |

Note: determine whether a SNP-set method was inflated, well-controlled or conservative according to the average ratio between the empirical type I error and the given significance level over 10^5^ simulations. inflated: ratio>1.2; well-controlled: 0.8≤ratio≤1.2; conservative: ratio<0.8.

## Table S3. Estimated power over 10^3^ simulations with common variants

| PVE | prop/effect size | *m* | BT | SKATO | SKAT | Simes | minP | HMP | ACAT |
| --- | --- | --- | --- | --- | --- | --- | --- | --- | --- |
| 0.003 | 0.05 | 50 | 0.107 | 0.155 | 0.154 | 0.150 | 0.141 | 0.171 | 0.171 |
|  |  | 200 | 0.036 | 0.065 | 0.059 | 0.044 | 0.037 | 0.054 | 0.054 |
|  |  | 500 | 0.003 | 0.020 | 0.033 | 0.012 | 0.011 | 0.016 | 0.016 |
|  | 0.2 | 50 | 0.083 | 0.115 | 0.113 | 0.107 | 0.101 | 0.118 | 0.118 |
|  |  | 200 | 0.039 | 0.058 | 0.053 | 0.033 | 0.027 | 0.038 | 0.038 |
|  |  | 500 | 0.005 | 0.025 | 0.031 | 0.010 | 0.010 | 0.015 | 0.015 |
|  | 0.5 | 50 | 0.094 | 0.140 | 0.142 | 0.111 | 0.098 | 0.127 | 0.127 |
|  |  | 200 | 0.031 | 0.066 | 0.058 | 0.030 | 0.032 | 0.038 | 0.038 |
|  |  | 500 | 0.003 | 0.010 | 0.023 | 0.005 | 0.005 | 0.005 | 0.005 |
| 0.005 | 0.05 | 50 | 0.292 | 0.420 | 0.418 | 0.461 | 0.443 | 0.493 | 0.493 |
|  |  | 200 | 0.094 | 0.208 | 0.220 | 0.187 | 0.178 | 0.222 | 0.222 |
|  |  | 500 | 0.015 | 0.111 | 0.125 | 0.065 | 0.053 | 0.083 | 0.083 |
|  | 0.2 | 50 | 0.240 | 0.355 | 0.355 | 0.355 | 0.333 | 0.387 | 0.387 |
|  |  | 200 | 0.098 | 0.225 | 0.220 | 0.166 | 0.154 | 0.203 | 0.203 |
|  |  | 500 | 0.022 | 0.099 | 0.118 | 0.055 | 0.052 | 0.063 | 0.064 |
|  | 0.5 | 50 | 0.255 | 0.373 | 0.358 | 0.369 | 0.360 | 0.390 | 0.390 |
|  |  | 200 | 0.103 | 0.221 | 0.218 | 0.165 | 0.146 | 0.192 | 0.192 |
|  |  | 500 | 0.010 | 0.087 | 0.105 | 0.037 | 0.028 | 0.043 | 0.043 |
| 0.01 | 0.05 | 50 | 0.559 | 0.804 | 0.808 | 0.936 | 0.936 | 0.946 | 0.946 |
|  |  | 200 | 0.301 | 0.642 | 0.652 | 0.712 | 0.708 | 0.751 | 0.751 |
|  |  | 500 | 0.092 | 0.445 | 0.493 | 0.433 | 0.419 | 0.463 | 0.463 |
|  | 0.2 | 50 | 0.521 | 0.778 | 0.768 | 0.872 | 0.864 | 0.884 | 0.884 |
|  |  | 200 | 0.296 | 0.650 | 0.662 | 0.667 | 0.646 | 0.697 | 0.697 |
|  |  | 500 | 0.085 | 0.435 | 0.480 | 0.335 | 0.324 | 0.377 | 0.377 |
|  | 0.5 | 50 | 0.492 | 0.756 | 0.747 | 0.839 | 0.840 | 0.848 | 0.848 |
|  |  | 200 | 0.301 | 0.651 | 0.661 | 0.661 | 0.654 | 0.694 | 0.694 |
|  |  | 500 | 0.080 | 0.432 | 0.485 | 0.331 | 0.324 | 0.368 | 0.368 |
| 0.003 | double | 50 | 0.086 | 0.118 | 0.116 | 0.094 | 0.086 | 0.108 | 0.108 |
|  |  | 200 | 0.026 | 0.064 | 0.067 | 0.043 | 0.039 | 0.051 | 0.051 |
|  |  | 500 | 0.002 | 0.016 | 0.022 | 0.003 | 0.001 | 0.005 | 0.005 |
|  | normal | 50 | 0.079 | 0.119 | 0.116 | 0.086 | 0.081 | 0.103 | 0.103 |
|  |  | 200 | 0.032 | 0.059 | 0.054 | 0.040 | 0.036 | 0.050 | 0.050 |
|  |  | 500 | 0.011 | 0.025 | 0.029 | 0.008 | 0.006 | 0.011 | 0.011 |
|  | t | 50 | 0.068 | 0.101 | 0.102 | 0.078 | 0.075 | 0.089 | 0.089 |
|  |  | 200 | 0.033 | 0.073 | 0.061 | 0.030 | 0.029 | 0.037 | 0.037 |
|  |  | 500 | 0.006 | 0.022 | 0.026 | 0.010 | 0.006 | 0.010 | 0.010 |
| 0.005 | double | 50 | 0.233 | 0.343 | 0.332 | 0.321 | 0.316 | 0.355 | 0.355 |
|  |  | 200 | 0.100 | 0.226 | 0.220 | 0.180 | 0.176 | 0.196 | 0.196 |
|  |  | 500 | 0.018 | 0.094 | 0.125 | 0.036 | 0.030 | 0.049 | 0.049 |
|  | normal | 50 | 0.216 | 0.334 | 0.336 | 0.319 | 0.313 | 0.342 | 0.342 |
|  |  | 200 | 0.089 | 0.213 | 0.203 | 0.140 | 0.143 | 0.171 | 0.171 |
|  |  | 500 | 0.026 | 0.098 | 0.128 | 0.047 | 0.040 | 0.059 | 0.059 |
|  | t | 50 | 0.252 | 0.363 | 0.359 | 0.341 | 0.328 | 0.366 | 0.366 |
|  |  | 200 | 0.102 | 0.235 | 0.229 | 0.165 | 0.157 | 0.191 | 0.191 |
|  |  | 500 | 0.021 | 0.084 | 0.101 | 0.046 | 0.040 | 0.058 | 0.058 |
| 0.01 | double | 50 | 0.495 | 0.759 | 0.754 | 0.825 | 0.829 | 0.846 | 0.846 |
|  |  | 200 | 0.292 | 0.655 | 0.658 | 0.632 | 0.631 | 0.669 | 0.669 |
|  |  | 500 | 0.095 | 0.466 | 0.516 | 0.352 | 0.336 | 0.385 | 0.385 |
|  | normal | 50 | 0.493 | 0.757 | 0.762 | 0.833 | 0.841 | 0.849 | 0.849 |
|  |  | 200 | 0.286 | 0.654 | 0.662 | 0.637 | 0.647 | 0.674 | 0.674 |
|  |  | 500 | 0.093 | 0.460 | 0.505 | 0.367 | 0.342 | 0.404 | 0.404 |
|  | t | 50 | 0.500 | 0.773 | 0.766 | 0.838 | 0.841 | 0.858 | 0.858 |
|  |  | 200 | 0.298 | 0.655 | 0.665 | 0.661 | 0.653 | 0.690 | 0.690 |
|  |  | 500 | 0.101 | 0.426 | 0.475 | 0.343 | 0.318 | 0.383 | 0.383 |
| 0.003 | 0.05 | 50 | 0.149 | 0.207 | 0.209 | 0.200 | 0.199 | 0.216 | 0.216 |
|  |  | 200 | 0.054 | 0.110 | 0.103 | 0.076 | 0.071 | 0.086 | 0.086 |
|  |  | 500 | 0.010 | 0.044 | 0.051 | 0.018 | 0.012 | 0.024 | 0.024 |
|  | 0.2 | 50 | 0.120 | 0.168 | 0.165 | 0.144 | 0.136 | 0.158 | 0.158 |
|  |  | 200 | 0.048 | 0.100 | 0.103 | 0.075 | 0.065 | 0.082 | 0.082 |
|  |  | 500 | 0.005 | 0.035 | 0.044 | 0.013 | 0.014 | 0.017 | 0.017 |
|  | 0.5 | 50 | 0.118 | 0.169 | 0.163 | 0.159 | 0.142 | 0.167 | 0.167 |
|  |  | 200 | 0.039 | 0.094 | 0.095 | 0.071 | 0.065 | 0.080 | 0.080 |
|  |  | 500 | 0.004 | 0.038 | 0.050 | 0.021 | 0.015 | 0.024 | 0.024 |
| 0.005 | 0.05 | 50 | 0.257 | 0.365 | 0.364 | 0.383 | 0.380 | 0.394 | 0.394 |
|  |  | 200 | 0.130 | 0.264 | 0.254 | 0.209 | 0.208 | 0.233 | 0.233 |
|  |  | 500 | 0.031 | 0.130 | 0.142 | 0.083 | 0.071 | 0.097 | 0.097 |
|  | 0.2 | 50 | 0.228 | 0.316 | 0.310 | 0.326 | 0.312 | 0.338 | 0.338 |
|  |  | 200 | 0.119 | 0.263 | 0.267 | 0.224 | 0.211 | 0.247 | 0.247 |
|  |  | 500 | 0.020 | 0.135 | 0.154 | 0.084 | 0.076 | 0.099 | 0.099 |
|  | 0.5 | 50 | 0.226 | 0.320 | 0.323 | 0.334 | 0.330 | 0.343 | 0.343 |
|  |  | 200 | 0.109 | 0.243 | 0.242 | 0.211 | 0.199 | 0.237 | 0.237 |
|  |  | 500 | 0.018 | 0.119 | 0.133 | 0.072 | 0.069 | 0.093 | 0.093 |
| 0.01 | 0.05 | 50 | 0.386 | 0.583 | 0.582 | 0.615 | 0.613 | 0.624 | 0.624 |
|  |  | 200 | 0.267 | 0.543 | 0.541 | 0.544 | 0.540 | 0.560 | 0.560 |
|  |  | 500 | 0.096 | 0.425 | 0.454 | 0.344 | 0.339 | 0.380 | 0.380 |
|  | 0.2 | 50 | 0.330 | 0.511 | 0.508 | 0.563 | 0.567 | 0.574 | 0.574 |
|  |  | 200 | 0.266 | 0.540 | 0.542 | 0.536 | 0.541 | 0.561 | 0.561 |
|  |  | 500 | 0.093 | 0.422 | 0.459 | 0.362 | 0.358 | 0.393 | 0.393 |
|  | 0.5 | 50 | 0.368 | 0.541 | 0.533 | 0.578 | 0.571 | 0.585 | 0.585 |
|  |  | 200 | 0.241 | 0.518 | 0.521 | 0.513 | 0.517 | 0.538 | 0.538 |
|  |  | 500 | 0.086 | 0.440 | 0.475 | 0.349 | 0.341 | 0.377 | 0.377 |

**Note:** PVE: the phenotypic variance explained by SNPs; prop: the proportion of SNPs which had substantial impacts on genotype under the sparse and mixed cases; effect size: the distribution where the effect size of SNPs was assumed to follow under the polygenic case; normal: standard normal distribution; double: standard double exponential distribution; *t*: standard *t*-distribution.

## Table S4. Total running time of 10^3^ simulations for the seven SNP-set methods under various simulation settings

| case | PVE | SNPs | prop/effect size | BT | SKATO | SKAT | Simes | minP | HMP | ACAT |
| --- | --- | --- | --- | --- | --- | --- | --- | --- | --- | --- |
| sparse | 0.001 | 50 | 0.05 | 3.98 | 120.13 | 4.60 | 4.39 | 4.97 | 4.14 | 3.89 |
|  |  |  | 0.2 | 3.91 | 129.75 | 4.52 | 3.83 | 4.75 | 4.01 | 3.98 |
|  |  |  | 0.5 | 4.28 | 130.11 | 4.97 | 4.11 | 4.52 | 4.01 | 3.82 |
|  |  | 200 | 0.05 | 56.50 | 318.72 | 73.64 | 56.53 | 61.77 | 57.25 | 56.80 |
|  |  |  | 0.2 | 57.00 | 322.38 | 71.86 | 56.39 | 64.83 | 56.58 | 57.92 |
|  |  |  | 0.5 | 63.47 | 298.79 | 73.34 | 63.47 | 67.71 | 58.72 | 57.64 |
|  |  | 500 | 0.05 | 260.01 | 1572.88 | 678.86 | 281.71 | 314.47 | 293.13 | 291.43 |
|  |  |  | 0.2 | 253.37 | 1211.10 | 499.75 | 281.34 | 309.05 | 295.23 | 241.62 |
|  |  |  | 0.5 | 304.51 | 1271.23 | 461.77 | 298.68 | 309.96 | 292.06 | 312.52 |
|  | 0.003 | 50 | 0.05 | 4.03 | 344.55 | 5.04 | 3.99 | 5.28 | 4.13 | 3.98 |
|  |  |  | 0.2 | 3.87 | 395.79 | 5.22 | 3.96 | 4.84 | 4.14 | 3.86 |
|  |  |  | 0.5 | 4.09 | 256.46 | 5.14 | 4.22 | 4.86 | 3.89 | 4.00 |
|  |  | 200 | 0.05 | 58.98 | 671.15 | 67.11 | 54.05 | 61.76 | 59.08 | 56.12 |
|  |  |  | 0.2 | 56.61 | 533.19 | 76.30 | 55.62 | 61.08 | 40.46 | 61.85 |
|  |  |  | 0.5 | 57.78 | 560.91 | 76.46 | 57.05 | 64.88 | 60.26 | 61.10 |
|  |  | 500 | 0.05 | 316.56 | 1318.73 | 505.69 | 303.00 | 328.98 | 315.71 | 362.67 |
|  |  |  | 0.2 | 319.79 | 1316.45 | 458.21 | 317.46 | 306.43 | 316.77 | 301.26 |
|  |  |  | 0.5 | 308.76 | 1407.00 | 525.20 | 290.71 | 316.09 | 305.06 | 280.83 |
|  | 0.005 | 50 | 0.05 | 3.84 | 659.52 | 7.49 | 4.07 | 4.73 | 4.21 | 3.89 |
|  |  |  | 0.2 | 4.10 | 483.83 | 7.16 | 3.83 | 4.48 | 4.12 | 3.98 |
|  |  |  | 0.5 | 4.87 | 623.52 | 7.01 | 4.07 | 4.46 | 4.09 | 4.17 |
|  |  | 200 | 0.05 | 59.80 | 1572.17 | 76.22 | 56.23 | 60.60 | 57.64 | 60.49 |
|  |  |  | 0.2 | 58.59 | 1234.77 | 70.84 | 58.35 | 60.55 | 58.62 | 52.71 |
|  |  |  | 0.5 | 39.91 | 1035.90 | 72.90 | 61.30 | 60.78 | 58.61 | 62.98 |
|  |  | 500 | 0.05 | 305.31 | 1398.20 | 479.43 | 272.25 | 285.03 | 301.52 | 291.94 |
|  |  |  | 0.2 | 297.73 | 1561.44 | 524.48 | 296.46 | 312.76 | 295.70 | 279.46 |
|  |  |  | 0.5 | 307.09 | 1367.46 | 465.87 | 310.03 | 305.33 | 305.55 | 296.08 |
|  | 0.01 | 50 | 0.05 | 4.14 | 2912.40 | 17.86 | 3.83 | 4.32 | 4.28 | 3.79 |
|  |  |  | 0.2 | 4.01 | 2329.63 | 14.92 | 3.87 | 4.59 | 3.85 | 4.17 |
|  |  |  | 0.5 | 3.83 | 2388.61 | 15.79 | 4.34 | 4.85 | 3.92 | 3.98 |
|  |  | 200 | 0.05 | 39.95 | 7114.75 | 76.37 | 47.77 | 61.05 | 60.51 | 59.35 |
|  |  |  | 0.2 | 64.63 | 7264.82 | 89.37 | 66.33 | 71.17 | 59.95 | 58.70 |
|  |  |  | 0.5 | 57.11 | 8951.24 | 71.29 | 66.67 | 71.73 | 66.75 | 69.40 |
|  |  | 500 | 0.05 | 276.99 | 4303.71 | 506.32 | 289.64 | 283.86 | 402.44 | 411.38 |
|  |  |  | 0.2 | 320.97 | 4469.48 | 450.42 | 276.29 | 308.69 | 317.12 | 315.34 |
|  |  |  | 0.5 | 274.22 | 3596.66 | 501.36 | 321.89 | 338.75 | 321.30 | 270.18 |
| polygenic | 0.001 | 50 | double | 3.87 | 128.73 | 4.38 | 3.76 | 4.55 | 3.89 | 3.87 |
|  |  |  | normal | 4.05 | 133.14 | 4.59 | 4.65 | 5.43 | 3.93 | 3.98 |
|  |  |  | *t* | 4.06 | 121.30 | 4.55 | 4.14 | 4.51 | 3.87 | 3.96 |
|  |  | 200 | double | 56.44 | 291.77 | 71.20 | 57.83 | 59.16 | 54.21 | 71.62 |
|  |  |  | normal | 60.95 | 275.46 | 76.35 | 59.53 | 59.36 | 60.19 | 49.75 |
|  |  |  | *t* | 60.10 | 326.13 | 77.92 | 58.85 | 60.46 | 58.19 | 55.74 |
|  |  | 500 | double | 293.53 | 1167.42 | 476.06 | 281.88 | 313.09 | 295.61 | 275.23 |
|  |  |  | normal | 301.16 | 1280.57 | 516.17 | 345.79 | 289.78 | 349.44 | 301.90 |
|  |  |  | *t* | 278.42 | 1214.13 | 524.50 | 305.20 | 316.62 | 285.23 | 293.29 |
|  | 0.003 | 50 | double | 3.93 | 271.28 | 4.94 | 3.79 | 4.60 | 4.28 | 3.70 |
|  |  |  | normal | 3.92 | 298.17 | 5.09 | 4.00 | 5.01 | 3.98 | 3.81 |
|  |  |  | *t* | 3.97 | 270.03 | 4.66 | 3.70 | 5.02 | 4.13 | 4.03 |
|  |  | 200 | double | 56.94 | 510.16 | 79.76 | 57.82 | 62.96 | 53.07 | 57.08 |
|  |  |  | normal | 54.90 | 522.69 | 80.49 | 60.12 | 84.58 | 76.81 | 55.16 |
|  |  |  | *t* | 39.78 | 485.94 | 80.20 | 62.82 | 64.13 | 55.33 | 73.42 |
|  |  | 500 | double | 301.37 | 1405.67 | 522.04 | 304.56 | 290.50 | 278.38 | 306.83 |
|  |  |  | normal | 298.61 | 1231.72 | 489.36 | 312.97 | 332.25 | 232.73 | 311.67 |
|  |  |  | *t* | 314.51 | 1308.70 | 489.21 | 286.11 | 289.78 | 265.06 | 294.14 |
|  | 0.005 | 50 | double | 3.90 | 502.57 | 6.70 | 4.04 | 4.94 | 4.22 | 3.92 |
|  |  | 50 | normal | 4.03 | 496.50 | 7.23 | 4.01 | 4.68 | 3.97 | 3.84 |
|  |  | 50 | *t* | 4.03 | 497.58 | 6.58 | 3.88 | 4.38 | 3.97 | 3.92 |
|  |  | 200 | double | 61.70 | 1198.61 | 69.42 | 57.95 | 67.50 | 47.31 | 51.97 |
|  |  | 200 | normal | 61.55 | 920.00 | 78.79 | 61.89 | 63.65 | 59.48 | 57.39 |
|  |  | 200 | *t* | 65.38 | 835.62 | 74.59 | 39.46 | 69.74 | 66.80 | 57.07 |
|  |  | 500 | double | 321.49 | 1457.07 | 521.19 | 285.34 | 301.11 | 272.73 | 327.24 |
|  |  | 500 | normal | 321.12 | 1758.60 | 460.32 | 313.06 | 332.22 | 305.56 | 292.82 |
|  |  | 500 | *t* | 318.07 | 1493.26 | 530.62 | 284.05 | 295.08 | 298.19 | 302.35 |
|  | 0.01 | 50 | double | 4.14 | 2162.43 | 15.01 | 3.74 | 4.61 | 4.04 | 3.80 |
|  |  | 50 | normal | 3.92 | 2628.63 | 14.96 | 4.08 | 4.42 | 4.22 | 3.84 |
|  |  | 50 | *t* | 3.91 | 2354.91 | 14.59 | 3.92 | 4.54 | 3.89 | 4.06 |
|  |  | 200 | double | 60.82 | 9232.44 | 76.66 | 57.60 | 58.25 | 59.42 | 58.93 |
|  |  | 200 | normal | 64.67 | 7602.39 | 79.70 | 54.73 | 50.39 | 62.83 | 60.34 |
|  |  | 200 | *t* | 57.36 | 8168.42 | 83.18 | 56.60 | 62.64 | 60.07 | 56.95 |
|  |  | 500 | double | 404.01 | 4613.48 | 504.33 | 274.87 | 291.95 | 297.70 | 301.09 |
|  |  | 500 | normal | 292.15 | 4822.55 | 511.33 | 290.45 | 315.20 | 301.26 | 410.21 |
|  |  | 500 | *t* | 304.52 | 3946.75 | 467.32 | 284.67 | 329.33 | 284.55 | 279.97 |
| mixed | 0.001 | 50 | 0.05 | 4.52 | 129.77 | 4.25 | 3.84 | 5.13 | 4.26 | 4.16 |
|  |  | 50 | 0.2 | 4.78 | 152.62 | 6.54 | 4.54 | 5.14 | 3.98 | 4.28 |
|  |  | 50 | 0.5 | 4.17 | 129.84 | 4.87 | 4.32 | 5.87 | 4.43 | 4.39 |
|  |  | 200 | 0.05 | 70.03 | 346.24 | 89.99 | 70.45 | 75.62 | 70.04 | 68.89 |
|  |  | 200 | 0.2 | 67.77 | 315.81 | 93.07 | 74.61 | 79.18 | 74.82 | 66.64 |
|  |  | 200 | 0.5 | 74.97 | 319.64 | 81.95 | 74.92 | 79.15 | 74.67 | 63.50 |
|  |  | 500 | 0.05 | 314.96 | 1348.93 | 460.39 | 282.87 | 301.08 | 310.25 | 285.22 |
|  |  | 500 | 0.2 | 326.91 | 1358.42 | 549.73 | 308.69 | 328.20 | 325.87 | 295.92 |
|  |  | 500 | 0.5 | 306.99 | 1316.85 | 520.50 | 311.84 | 327.02 | 309.95 | 308.56 |
|  | 0.003 | 50 | 0.05 | 4.36 | 427.10 | 6.52 | 3.87 | 4.82 | 4.12 | 4.11 |
|  |  | 50 | 0.2 | 4.53 | 291.02 | 5.63 | 3.86 | 4.75 | 4.19 | 4.01 |
|  |  | 50 | 0.5 | 4.04 | 280.35 | 5.36 | 4.27 | 4.90 | 3.97 | 3.89 |
|  |  | 200 | 0.05 | 60.94 | 758.54 | 90.73 | 60.68 | 65.69 | 58.48 | 61.27 |
|  |  | 200 | 0.2 | 64.51 | 1373.54 | 81.37 | 63.17 | 67.55 | 70.54 | 62.49 |
|  |  | 200 | 0.5 | 67.99 | 566.72 | 81.45 | 66.06 | 70.75 | 65.72 | 66.32 |
|  |  | 500 | 0.05 | 304.53 | 1409.53 | 537.92 | 305.33 | 294.24 | 278.73 | 280.90 |
|  |  | 500 | 0.2 | 301.00 | 1354.07 | 460.32 | 310.43 | 326.84 | 306.93 | 296.04 |
|  |  | 500 | 0.5 | 310.08 | 1420.35 | 483.57 | 282.02 | 318.55 | 282.84 | 312.77 |
|  | 0.005 | 50 | 0.05 | 4.11 | 1118.34 | 13.99 | 5.20 | 6.07 | 4.21 | 3.91 |
|  |  | 50 | 0.2 | 4.03 | 763.47 | 8.20 | 3.98 | 4.88 | 3.99 | 4.02 |
|  |  | 50 | 0.5 | 4.11 | 832.07 | 8.16 | 4.03 | 4.79 | 4.47 | 4.05 |
|  |  | 200 | 0.05 | 60.23 | 2114.33 | 70.37 | 61.72 | 66.90 | 59.65 | 50.04 |
|  |  | 200 | 0.2 | 57.94 | 1967.27 | 79.06 | 63.08 | 51.55 | 65.46 | 60.21 |
|  |  | 200 | 0.5 | 57.71 | 5181.29 | 78.88 | 61.31 | 59.63 | 57.74 | 59.06 |
|  |  | 500 | 0.05 | 294.48 | 1775.66 | 512.68 | 297.80 | 321.09 | 290.51 | 307.81 |
|  |  | 500 | 0.2 | 304.85 | 1773.52 | 488.75 | 274.40 | 291.59 | 305.43 | 306.90 |
|  |  | 500 | 0.5 | 405.03 | 2040.52 | 504.78 | 316.02 | 333.30 | 306.45 | 280.18 |
|  | 0.01 | 50 | 0.05 | 4.24 | 2882.92 | 21.95 | 3.78 | 4.63 | 3.92 | 3.84 |
|  |  | 50 | 0.2 | 4.29 | 2533.07 | 19.80 | 3.90 | 4.58 | 3.95 | 4.00 |
|  |  | 50 | 0.5 | 3.87 | 2440.22 | 19.66 | 3.97 | 4.88 | 3.97 | 3.92 |
|  |  | 200 | 0.05 | 58.44 | 7372.57 | 85.09 | 62.77 | 59.19 | 60.02 | 57.50 |
|  |  | 200 | 0.2 | 59.14 | 7738.16 | 89.89 | 65.13 | 67.82 | 58.83 | 61.18 |
|  |  | 200 | 0.5 | 64.44 | 7976.52 | 80.90 | 59.21 | 51.76 | 62.73 | 55.22 |
|  |  | 500 | 0.05 | 275.22 | 4091.25 | 488.99 | 239.97 | 305.25 | 274.18 | 283.38 |
|  |  | 500 | 0.2 | 281.50 | 6056.93 | 475.40 | 286.11 | 301.55 | 285.14 | 282.81 |
|  |  | 500 | 0.5 | 276.66 | 4463.22 | 601.42 | 282.14 | 298.35 | 277.97 | 271.77 |

**Note:** The time unit is second; PVE: the phenotypic variance explained by SNPs; prop: the proportion of SNPs which had substantial impacts on genotype under the sparse and mixed cases; effect size: the distribution where the effect size of SNPs was assumed to follow under the polygenic case; normal: standard normal distribution; double: standard double exponential distribution; *t*: standard *t*-distribution.

## Table S5. Identified genes associated with the six psychiatric disorders

| disorder | Gene | *m* | BT | SKATO | SKAT | Simes | minP | HMP | ACAT | Best SNP | *P*_GWAS_ | novel |
| --- | --- | --- | --- | --- | --- | --- | --- | --- | --- | --- | --- | --- |
| ADHD | *ARTN* | 73 | 1.00E+00 | 1.89E-02 | 1.18E-02 | 7.16E-05 | 6.33E-05 | 7.11E-05 | 7.11E-05 | rs3791101 | 5.87E-11 |  |
| ADHD | *ATP6V0B* | 91 | 1.00E+00 | 6.39E-02 | 3.56E-02 | 8.93E-05 | 8.17E-05 | 8.91E-05 | 8.91E-05 | rs3791101 | 5.87E-11 |  |
| ADHD | *B4GALT2* | 90 | 1.00E+00 | 7.89E-02 | 4.40E-02 | 8.83E-05 | 8.13E-05 | 8.82E-05 | 8.82E-05 | rs3791101 | 5.87E-11 |  |
| ADHD | *C1orf210* | 78 | 1.00E+00 | 3.64E-03 | 2.13E-03 | 1.01E-02 | 2.66E-02 | 6.19E-03 | 6.19E-03 | rs2275180 | 3.36E-08 |  |
| ADHD | *CACNG1* | 75 | 7.23E-09 | 2.03E-08 | 1.00E+00 | 1.00E+00 | 1.00E+00 | 1.00E+00 | 1.00E+00 | rs11079678 | 4.45E-05 | √ |
| ADHD | *CCDC24* | 88 | 1.00E+00 | 1.44E-01 | 8.25E-02 | 8.63E-05 | 7.97E-05 | 8.63E-05 | 8.63E-05 | rs3791101 | 5.87E-11 |  |
| ADHD | *CD160* | 45 | 1.08E-02 | 1.86E-02 | 1.00E+00 | 1.00E+00 | 1.00E+00 | 1.00E+00 | 1.00E+00 | rs35880546 | 1.76E-05 | √ |
| ADHD | *CDC20* | 71 | 1.00E+00 | 4.99E-04 | 4.51E-04 | 4.60E-03 | 1.45E-02 | 2.58E-03 | 2.58E-03 | rs2275180 | 3.36E-08 |  |
| ADHD | *CDH8* | 172 | 1.00E+00 | 2.87E-02 | 1.38E-02 | 9.24E-01 | 7.84E-01 | 4.32E-01 | 1.00E+00 | rs8058677 | 5.73E-07 | √ |
| ADHD | *DPH2* | 89 | 1.00E+00 | 4.45E-02 | 2.51E-02 | 8.73E-05 | 7.96E-05 | 8.71E-05 | 8.71E-05 | rs3791101 | 5.87E-11 |  |
| ADHD | *DUSP6* | 73 | 1.00E+00 | 1.01E-04 | 2.70E-05 | 1.05E-03 | 1.44E-03 | 5.83E-04 | 5.83E-04 | rs704061 | 1.30E-09 |  |
| ADHD | *EIF5B* | 86 | 2.91E-02 | 4.93E-02 | 1.00E+00 | 1.00E+00 | 1.00E+00 | 1.00E+00 | 1.00E+00 | rs113073870 | 6.59E-05 | √ |
| ADHD | *ELOVL1* | 71 | 1.00E+00 | 4.99E-04 | 4.51E-04 | 4.60E-03 | 1.45E-02 | 2.58E-03 | 2.58E-03 | rs2842198 | 6.41E-09 |  |
| ADHD | *FEZF1* | 71 | 1.00E+00 | 3.93E-02 | 1.21E-02 | 2.30E-01 | 1.56E-01 | 1.51E-01 | 1.51E-01 | rs1348442 | 1.93E-07 | √ |
| ADHD | *FOXP2* | 127 | 1.00E+00 | 7.93E-02 | 6.65E-02 | 9.16E-02 | 6.62E-02 | 4.17E-02 | 4.17E-02 | rs10231382 | 4.05E-08 |  |
| ADHD | *HELZ* | 94 | 1.34E-03 | 2.53E-03 | 1.00E+00 | 1.00E+00 | 1.00E+00 | 1.00E+00 | 1.00E+00 | rs11079678 | 4.45E-05 | √ |
| ADHD | *HYI* | 69 | 1.00E+00 | 3.61E-05 | 3.24E-05 | 4.46E-04 | 4.15E-04 | 2.97E-04 | 2.97E-04 | rs2819336 | 2.76E-10 |  |
| ADHD | *IPO13* | 89 | 1.00E+00 | 2.37E-02 | 1.39E-02 | 8.73E-05 | 7.87E-05 | 8.70E-05 | 8.70E-05 | rs3791101 | 5.87E-11 |  |
| ADHD | *KDM4A* | 102 | 2.07E-08 | 1.25E-08 | 1.29E-06 | 1.82E-06 | 1.78E-06 | 1.01E-06 | 1.01E-06 | rs17531412 | 1.07E-12 |  |
| ADHD | *MANBA* | 97 | 1.00E+00 | 4.35E-02 | 3.42E-02 | 5.17E-02 | 8.82E-02 | 4.25E-02 | 4.25E-02 | rs227378 | 6.76E-08 | √ |
| ADHD | *MED8* | 66 | 1.00E+00 | 3.25E-04 | 3.03E-04 | 4.01E-03 | 1.28E-02 | 2.23E-03 | 2.23E-03 | rs2842198 | 6.41E-09 |  |
| ADHD | *MEF2C* | 102 | 1.00E+00 | 1.27E-02 | 9.05E-03 | 3.08E-02 | 3.11E-02 | 1.80E-02 | 1.80E-02 | rs304132 | 3.01E-08 |  |
| ADHD | *MPL* | 78 | 1.00E+00 | 5.99E-04 | 4.47E-04 | 5.05E-03 | 1.80E-02 | 2.93E-03 | 2.93E-03 | rs2275180 | 3.36E-08 |  |
| ADHD | *NFKB1* | 104 | 1.00E+00 | 2.38E-01 | 2.08E-01 | 5.55E-02 | 1.05E-01 | 4.58E-02 | 4.58E-02 | rs227378 | 6.76E-08 | √ |
| ADHD | *POC1B* | 98 | 1.00E+00 | 1.67E-01 | 8.50E-02 | 1.41E-03 | 1.62E-03 | 7.82E-04 | 7.82E-04 | rs704061 | 1.30E-09 |  |
| ADHD | *PTPRF* | 113 | 2.12E-03 | 2.20E-08 | 9.83E-08 | 2.02E-06 | 1.98E-06 | 1.71E-06 | 1.71E-06 | rs17531412 | 1.07E-12 |  |
| ADHD | *SEMA6D* | 350 | 1.00E+00 | 3.96E-02 | 2.08E-02 | 1.82E-02 | 1.27E-02 | 1.77E-02 | 1.00E+00 | rs8039398 | 3.11E-09 |  |
| ADHD | *SLC6A9* | 104 | 1.00E+00 | 1.41E-01 | 7.92E-02 | 1.02E-04 | 9.49E-05 | 1.02E-04 | 1.02E-04 | rs3791101 | 5.87E-11 |  |
| ADHD | *SORCS3* | 390 | 1.00E+00 | 6.30E-02 | 2.94E-02 | 1.15E-01 | 5.97E-02 | 5.67E-02 | 1.00E+00 | rs11591402 | 1.76E-08 |  |
| ADHD | *ST3GAL3* | 149 | 1.00E+00 | 6.08E-06 | 2.82E-06 | 2.66E-06 | 2.60E-06 | 1.56E-06 | 1.56E-06 | rs17531412 | 1.07E-12 |  |
| ADHD | *SZT2* | 88 | 1.00E+00 | 3.47E-05 | 3.11E-05 | 5.69E-04 | 5.04E-04 | 3.65E-04 | 3.65E-04 | rs2819336 | 2.76E-10 |  |
| ADHD | *TALDO1* | 77 | 1.00E+00 | 1.47E-01 | 4.19E-02 | 4.26E-01 | 3.21E-01 | 1.80E-01 | 1.80E-01 | rs28633403 | 4.44E-07 | √ |
| ADHD | *TIE1* | 87 | 1.00E+00 | 4.76E-04 | 3.81E-04 | 6.48E-03 | 2.60E-02 | 4.02E-03 | 4.02E-03 | rs2275180 | 3.36E-08 |  |
| ADHD | *TMEM125* | 82 | 1.00E+00 | 6.44E-03 | 3.11E-03 | 1.33E-02 | 2.89E-02 | 8.07E-03 | 8.07E-03 | rs2275180 | 3.36E-08 |  |
| ADHD | *UBE2D3* | 46 | 1.00E+00 | 5.27E-01 | 4.84E-01 | 1.06E-01 | 3.35E-02 | 8.48E-02 | 8.48E-02 | rs227378 | 6.76E-08 | √ |
| ADHD | *WDR65* | 97 | 1.00E+00 | 1.13E-01 | 5.59E-02 | 1.88E-02 | 3.30E-02 | 1.10E-02 | 1.10E-02 | rs2275180 | 3.36E-08 |  |
| ASD | *NKX2-2* | 57 | 1.00E+00 | 5.98E-01 | 4.28E-01 | 5.88E-02 | 7.45E-02 | 4.70E-02 | 4.70E-02 | rs809220 | 1.85E-08 |  |
| ASD | *NKX2-4* | 66 | 1.00E+00 | 2.55E-04 | 1.83E-04 | 2.57E-03 | 7.85E-03 | 1.57E-03 | 1.57E-03 | rs73128965 | 1.51E-08 |  |
| ASD | *RP1L1* | 121 | 1.00E+00 | 2.32E-02 | 4.98E-02 | 9.88E-02 | 8.09E-02 | 9.15E-02 | 9.15E-02 | rs4841432 | 4.87E-08 |  |
| ASD | *SOX7* | 114 | 2.24E-04 | 3.05E-04 | 1.01E-01 | 9.31E-02 | 5.36E-02 | 8.36E-02 | 8.36E-02 | rs4841432 | 4.87E-08 |  |
| ASD | *XRN2* | 87 | 1.00E+00 | 5.73E-05 | 4.02E-05 | 3.08E-03 | 1.68E-03 | 1.05E-03 | 1.05E-03 | rs910805 | 2.22E-09 |  |
| BIP | *ADD3* | 68 | 1.00E+00 | 3.38E-03 | 3.20E-03 | 6.73E-02 | 8.32E-02 | 2.66E-02 | 2.66E-02 | rs4595478 | 1.85E-07 | √ |
| BIP | *ANKRD23* | 28 | 1.00E+00 | 1.00E+00 | 1.00E+00 | 3.05E-03 | 2.17E-03 | 2.21E-03 | 2.21E-03 | rs2314398 | 5.21E-09 |  |
| BIP | *AP1M2* | 63 | 1.00E+00 | 4.35E-03 | 3.43E-03 | 5.64E-02 | 9.29E-02 | 2.96E-02 | 2.96E-02 | rs3843751 | 1.46E-07 | √ |
| BIP | *ATG4D* | 60 | 1.00E+00 | 1.81E-02 | 1.18E-02 | 9.27E-02 | 1.08E-01 | 4.89E-02 | 4.89E-02 | rs3843751 | 1.46E-07 | √ |
| BIP | *ATP13A1* | 86 | 1.00E+00 | 1.42E-01 | 1.02E-01 | 5.24E-02 | 3.87E-02 | 4.48E-02 | 4.48E-02 | rs1036215 | 3.62E-08 |  |
| BIP | *CACNA1C* | 381 | 1.00E+00 | 1.98E-03 | 2.84E-04 | 1.11E-02 | 1.45E-02 | 5.37E-03 | 5.37E-03 | rs10744560 | 2.80E-09 |  |
| BIP | *CD47* | 74 | 1.00E+00 | 8.52E-02 | 3.32E-02 | 1.57E-01 | 9.22E-02 | 1.11E-01 | 1.11E-01 | rs3804640 | 1.00E-07 | √ |
| BIP | *CDKN2D* | 61 | 1.00E+00 | 6.72E-03 | 5.04E-03 | 7.80E-02 | 1.00E-01 | 3.85E-02 | 3.85E-02 | rs3843751 | 1.46E-07 | √ |
| BIP | *CILP2* | 58 | 1.00E+00 | 9.73E-02 | 6.92E-02 | 6.72E-03 | 1.75E-02 | 4.71E-03 | 4.71E-03 | rs8110171 | 1.70E-08 |  |
| BIP | *CNNM3* | 30 | 1.00E+00 | 1.00E+00 | 1.00E+00 | 3.27E-03 | 2.29E-03 | 2.33E-03 | 2.33E-03 | rs2314398 | 5.21E-09 |  |
| BIP | *CNNM4* | 35 | 1.00E+00 | 1.00E+00 | 1.00E+00 | 3.81E-03 | 2.65E-03 | 2.72E-03 | 2.72E-03 | rs2314398 | 5.21E-09 |  |
| BIP | *COX8A* | 42 | 1.00E+00 | 3.34E-02 | 3.17E-02 | 1.43E-01 | 3.14E-01 | 9.04E-02 | 9.04E-02 | rs11231677 | 4.78E-07 | √ |
| BIP | *CSF3* | 74 | 1.00E+00 | 8.51E-02 | 2.50E-02 | 7.15E-01 | 7.54E-01 | 3.21E-01 | 3.20E-01 | rs8067817 | 4.93E-07 | √ |
| BIP | *DAGLA* | 77 | 1.00E+00 | 2.32E-02 | 8.63E-03 | 7.28E-02 | 1.16E-01 | 4.37E-02 | 4.37E-02 | rs174578 | 8.47E-08 | √ |
| BIP | *DCLK3* | 111 | 1.00E+00 | 4.74E-04 | 9.89E-05 | 8.94E-08 | 8.95E-08 | 8.91E-08 | 8.92E-08 | rs9834970 | 4.79E-14 |  |
| BIP | *DFNA5* | 94 | 1.00E+00 | 4.02E-01 | 1.56E-01 | 2.12E-02 | 3.28E-02 | 1.39E-02 | 1.39E-02 | rs12672003 | 2.70E-08 |  |
| BIP | *DNAJB7* | 21 | 1.00E+00 | 4.55E-01 | 4.37E-01 | 8.39E-03 | 3.72E-03 | 7.94E-03 | 7.94E-03 | rs138321 | 4.25E-09 |  |
| BIP | *DNM2* | 84 | 1.00E+00 | 6.85E-02 | 5.38E-02 | 6.77E-02 | 1.03E-01 | 3.46E-02 | 3.46E-02 | rs3843751 | 1.46E-07 | √ |
| BIP | *DOCK2* | 396 | 1.00E+00 | 1.05E-01 | 3.54E-02 | 1.00E+00 | 1.00E+00 | 1.00E+00 | 1.00E+00 | rs7708829 | 1.65E-06 | √ |
| BIP | *EIF5B* | 86 | 7.71E-03 | 1.30E-02 | 1.00E+00 | 1.00E+00 | 1.00E+00 | 1.00E+00 | 1.00E+00 | rs10198056 | 1.33E-05 | √ |
| BIP | *EPM2AIP1* | 24 | 1.00E+00 | 1.43E-01 | 4.33E-02 | 7.63E-03 | 6.52E-03 | 6.95E-03 | 6.95E-03 | rs56149992 | 3.63E-09 |  |
| BIP | *FADS1* | 77 | 1.00E+00 | 1.27E-02 | 8.09E-03 | 7.28E-02 | 1.19E-01 | 4.36E-02 | 4.36E-02 | rs174592 | 4.03E-08 |  |
| BIP | *FADS2* | 79 | 1.00E+00 | 1.22E-02 | 8.63E-03 | 7.47E-02 | 1.24E-01 | 4.47E-02 | 4.47E-02 | rs174592 | 4.03E-08 |  |
| BIP | *FADS3* | 57 | 1.00E+00 | 6.33E-02 | 5.23E-02 | 5.39E-02 | 8.90E-02 | 3.23E-02 | 3.23E-02 | rs174592 | 4.03E-08 |  |
| BIP | *FAM196B* | 216 | 1.00E+00 | 1.35E-02 | 4.61E-03 | 1.00E+00 | 1.00E+00 | 1.00E+00 | 1.00E+00 | rs7708829 | 1.65E-06 | √ |
| BIP | *FEN1* | 75 | 1.00E+00 | 9.35E-03 | 5.77E-03 | 7.09E-02 | 1.15E-01 | 4.25E-02 | 4.25E-02 | rs174592 | 4.03E-08 |  |
| BIP | *GATAD2A* | 74 | 1.00E+00 | 2.22E-01 | 1.77E-01 | 9.41E-04 | 7.09E-04 | 5.45E-04 | 5.45E-04 | rs2011503 | 1.04E-09 |  |
| BIP | *GLT8D1* | 82 | 1.00E+00 | 5.16E-03 | 5.02E-03 | 8.09E-03 | 2.55E-03 | 3.26E-03 | 3.26E-03 | rs2302417 | 5.51E-09 |  |
| BIP | *GMIP* | 76 | 1.00E+00 | 1.07E-02 | 6.57E-03 | 1.47E-02 | 3.15E-02 | 9.72E-03 | 9.72E-03 | rs77427798 | 2.41E-08 |  |
| BIP | *GNL3* | 81 | 1.00E+00 | 5.70E-03 | 5.55E-03 | 1.19E-02 | 2.44E-03 | 4.28E-03 | 4.28E-03 | rs2302417 | 5.51E-09 |  |
| BIP | *GSDMA* | 82 | 3.75E-02 | 7.34E-04 | 1.05E-02 | 7.92E-01 | 6.87E-01 | 3.43E-01 | 3.43E-01 | rs8067817 | 4.93E-07 | √ |
| BIP | *GSDMB* | 63 | 1.15E-05 | 6.88E-06 | 9.38E-02 | 1.00E+00 | 1.00E+00 | 6.77E-01 | 6.76E-01 | rs8067817 | 4.93E-07 | √ |
| BIP | *HAPLN4* | 71 | 1.00E+00 | 7.24E-02 | 4.66E-02 | 6.23E-04 | 8.41E-04 | 3.16E-04 | 3.16E-04 | rs111444407 | 2.34E-10 |  |
| BIP | *HAUS2* | 42 | 1.00E+00 | 1.00E+00 | 1.00E+00 | 7.52E-02 | 3.66E-02 | 5.47E-02 | 5.47E-02 | rs11632169 | 1.06E-07 | √ |
| BIP | *HLF* | 111 | 1.00E+00 | 1.52E-02 | 3.61E-03 | 3.56E-02 | 2.95E-02 | 3.04E-02 | 1.00E+00 | rs4239193 | 6.52E-09 |  |
| BIP | *IFT57* | 65 | 1.00E+00 | 5.30E-02 | 3.88E-02 | 1.38E-01 | 6.14E-02 | 1.26E-01 | 1.26E-01 | rs3804640 | 1.00E-07 | √ |
| BIP | *ILF3* | 73 | 1.00E+00 | 1.58E-02 | 1.31E-02 | 5.89E-02 | 9.26E-02 | 3.01E-02 | 3.01E-02 | rs3843751 | 1.46E-07 | √ |
| BIP | *ITIH1* | 88 | 1.00E+00 | 5.80E-04 | 5.19E-04 | 5.36E-03 | 4.54E-03 | 2.30E-03 | 2.30E-03 | rs2302417 | 5.51E-09 |  |
| BIP | *ITIH3* | 84 | 1.00E+00 | 4.30E-04 | 3.74E-04 | 5.11E-03 | 4.83E-03 | 2.30E-03 | 2.30E-03 | rs2302417 | 5.51E-09 |  |
| BIP | *ITIH4* | 76 | 1.00E+00 | 3.09E-04 | 2.54E-04 | 4.63E-03 | 5.06E-03 | 2.12E-03 | 2.12E-03 | rs2302417 | 5.51E-09 |  |
| BIP | *KCNS1* | 70 | 1.00E+00 | 7.48E-04 | 3.93E-04 | 3.85E-01 | 2.82E-01 | 1.71E-01 | 1.71E-01 | rs6130764 | 5.52E-07 | √ |
| BIP | *KIAA1109* | 78 | 1.25E-01 | 1.91E-02 | 1.00E+00 | 9.58E-01 | 6.29E-01 | 6.46E-01 | 6.45E-01 | rs45605540 | 8.67E-07 | √ |
| BIP | *KLC2* | 71 | 1.00E+00 | 8.73E-02 | 7.10E-02 | 7.24E-02 | 1.71E-01 | 4.85E-02 | 4.85E-02 | rs10896090 | 2.16E-07 | √ |
| BIP | *KRI1* | 62 | 1.00E+00 | 8.82E-03 | 6.39E-03 | 9.25E-02 | 1.04E-01 | 4.24E-02 | 4.24E-02 | rs3843751 | 1.46E-07 | √ |
| BIP | *LMAN2L* | 33 | 1.00E+00 | 4.70E-01 | 3.06E-01 | 3.59E-03 | 2.72E-03 | 2.56E-03 | 2.56E-03 | rs2314398 | 5.21E-09 |  |
| BIP | *LPAR2* | 69 | 1.00E+00 | 7.79E-03 | 4.92E-03 | 1.33E-02 | 2.85E-02 | 8.83E-03 | 8.83E-03 | rs77427798 | 2.41E-08 |  |
| BIP | *LRRC3C* | 72 | 9.26E-04 | 1.84E-04 | 1.79E-02 | 6.96E-01 | 5.95E-01 | 3.15E-01 | 3.15E-01 | rs8067817 | 4.93E-07 | √ |
| BIP | *LRRC57* | 39 | 1.00E+00 | 1.00E+00 | 1.00E+00 | 6.99E-02 | 3.36E-02 | 5.11E-02 | 5.11E-02 | rs11632169 | 1.06E-07 | √ |
| BIP | *MAPK1* | 96 | 1.00E+00 | 2.16E-02 | 1.93E-02 | 1.84E-01 | 4.49E-01 | 1.07E-01 | 1.07E-01 | rs5754941 | 2.35E-07 | √ |
| BIP | *MAU2* | 58 | 1.00E+00 | 1.04E-01 | 7.89E-02 | 5.09E-04 | 5.73E-04 | 2.53E-04 | 2.53E-04 | rs111444407 | 2.34E-10 |  |
| BIP | *MED24* | 76 | 1.00E+00 | 9.89E-02 | 3.71E-02 | 7.34E-01 | 7.90E-01 | 3.35E-01 | 3.35E-01 | rs8067817 | 4.93E-07 | √ |
| BIP | *MEF2B* | 82 | 1.00E+00 | 1.00E+00 | 1.00E+00 | 1.31E-03 | 1.09E-03 | 8.54E-04 | 8.54E-04 | rs111444407 | 2.34E-10 |  |
| BIP | *MEF2BNB* | 83 | 1.00E+00 | 3.93E-01 | 1.62E-01 | 1.33E-03 | 1.12E-03 | 7.95E-04 | 7.95E-04 | rs111444407 | 2.34E-10 |  |
| BIP | *MEF2BNB-MEF2B* | 92 | 1.00E+00 | 1.00E+00 | 1.00E+00 | 1.47E-03 | 1.20E-03 | 8.81E-04 | 8.81E-04 | rs111444407 | 2.34E-10 |  |
| BIP | *MLH1* | 23 | 1.00E+00 | 3.00E-02 | 1.51E-02 | 7.31E-03 | 6.67E-03 | 6.66E-03 | 6.66E-03 | rs9867455 | 1.02E-08 |  |
| BIP | *MMD* | 91 | 1.00E+00 | 1.00E+00 | 5.04E-01 | 2.92E-02 | 2.32E-02 | 2.68E-02 | 2.68E-02 | rs4239193 | 6.52E-09 |  |
| BIP | *MPP6* | 101 | 1.00E+00 | 3.90E-01 | 2.28E-01 | 1.90E-02 | 3.35E-02 | 1.25E-02 | 1.25E-02 | rs12672003 | 2.70E-08 |  |
| BIP | *MRPS33* | 59 | 1.00E+00 | 1.01E-02 | 5.77E-03 | 7.64E-02 | 7.99E-02 | 3.54E-02 | 3.54E-02 | rs12703284 | 1.08E-07 | √ |
| BIP | *MUSTN1* | 73 | 1.00E+00 | 2.65E-04 | 2.12E-04 | 4.44E-03 | 5.30E-03 | 2.07E-03 | 2.07E-03 | rs2302417 | 5.51E-09 |  |
| BIP | *NAA40* | 47 | 1.00E+00 | 3.65E-02 | 3.49E-02 | 1.22E-01 | 3.00E-01 | 8.26E-02 | 8.26E-02 | rs11231677 | 4.78E-07 | √ |
| BIP | *NCAN* | 87 | 1.00E+00 | 5.04E-02 | 2.90E-02 | 7.63E-04 | 1.10E-03 | 3.87E-04 | 3.87E-04 | rs111444407 | 2.34E-10 |  |
| BIP | *NDUFA13* | 54 | 1.00E+00 | 1.79E-01 | 1.36E-01 | 5.68E-03 | 1.52E-02 | 3.99E-03 | 3.99E-03 | rs8110171 | 1.70E-08 |  |
| BIP | *NEK4* | 103 | 1.00E+00 | 1.16E-03 | 1.07E-03 | 6.27E-03 | 4.01E-03 | 2.54E-03 | 2.54E-03 | rs2302417 | 5.51E-09 |  |
| BIP | *NISCH* | 84 | 1.00E+00 | 1.07E-01 | 4.68E-02 | 6.75E-02 | 3.37E-02 | 2.84E-02 | 2.84E-02 | rs12629699 | 3.22E-08 |  |
| BIP | *NMB* | 67 | 1.00E+00 | 5.67E-03 | 3.69E-03 | 2.42E-02 | 4.61E-02 | 1.32E-02 | 1.32E-02 | rs71395455 | 1.88E-08 |  |
| BIP | *NR2C2AP* | 76 | 1.00E+00 | 1.77E-01 | 6.51E-02 | 1.22E-03 | 1.04E-03 | 7.28E-04 | 7.28E-04 | rs111444407 | 2.34E-10 |  |
| BIP | *NT5DC2* | 80 | 1.00E+00 | 1.77E-02 | 1.05E-02 | 3.33E-02 | 2.32E-02 | 1.37E-02 | 1.37E-02 | rs10433615 | 3.03E-08 |  |
| BIP | *ORMDL3* | 69 | 5.97E-05 | 2.63E-05 | 2.53E-02 | 7.62E-01 | 5.76E-01 | 3.40E-01 | 3.40E-01 | rs8067817 | 4.93E-07 | √ |
| BIP | *OSBPL3* | 180 | 1.00E+00 | 1.00E+00 | 4.43E-01 | 5.07E-02 | 8.14E-02 | 3.49E-02 | 3.49E-02 | rs17150022 | 2.87E-08 |  |
| BIP | *OTUB1* | 53 | 1.00E+00 | 2.03E-02 | 1.89E-02 | 1.81E-01 | 4.62E-01 | 1.14E-01 | 1.14E-01 | rs11231677 | 4.78E-07 | √ |
| BIP | *OTUD7B* | 51 | 1.00E+00 | 3.57E-01 | 2.71E-01 | 7.42E-02 | 2.18E-01 | 4.99E-02 | 4.99E-02 | rs12063329 | 4.82E-07 | √ |
| BIP | *PACS1* | 126 | 1.00E+00 | 1.48E-02 | 1.20E-02 | 8.68E-02 | 2.60E-01 | 4.99E-02 | 4.99E-02 | rs10896090 | 2.16E-07 | √ |
| BIP | *PBRM1* | 128 | 1.00E+00 | 6.62E-03 | 6.30E-03 | 1.88E-02 | 4.20E-03 | 6.25E-03 | 6.25E-03 | rs2302417 | 5.51E-09 |  |
| BIP | *PBX4* | 82 | 1.00E+00 | 1.48E-02 | 9.51E-03 | 1.05E-02 | 2.90E-02 | 7.17E-03 | 7.17E-03 | rs77427798 | 2.41E-08 |  |
| BIP | *PI3* | 70 | 1.00E+00 | 1.53E-02 | 1.32E-02 | 3.85E-01 | 2.42E-01 | 1.69E-01 | 1.69E-01 | rs6130764 | 5.52E-07 | √ |
| BIP | *PIGU* | 108 | 1.00E+00 | 6.14E-02 | 3.97E-02 | 3.72E-01 | 1.00E+00 | 2.48E-01 | 2.48E-01 | rs6060023 | 9.30E-07 | √ |
| BIP | *PLEKHO1* | 45 | 1.00E+00 | 2.66E-02 | 2.25E-02 | 5.64E-02 | 1.88E-01 | 3.72E-02 | 3.72E-02 | rs7544145 | 5.11E-07 | √ |
| BIP | *PSMD3* | 80 | 1.00E+00 | 3.21E-03 | 9.94E-03 | 7.73E-01 | 6.94E-01 | 3.36E-01 | 3.36E-01 | rs8067817 | 4.93E-07 | √ |
| BIP | *QTRT1* | 60 | 1.00E+00 | 3.42E-02 | 2.95E-02 | 4.84E-02 | 7.10E-02 | 2.47E-02 | 2.47E-02 | rs3843751 | 1.46E-07 | √ |
| BIP | *RAB1B* | 67 | 1.00E+00 | 1.04E-01 | 8.52E-02 | 6.83E-02 | 1.64E-01 | 4.98E-02 | 4.98E-02 | rs10896090 | 2.16E-07 | √ |
| BIP | *RAB3IL1* | 68 | 1.00E+00 | 2.45E-01 | 1.86E-01 | 6.43E-02 | 1.07E-01 | 3.97E-02 | 3.97E-02 | rs174592 | 4.03E-08 |  |
| BIP | *RBKS* | 46 | 1.00E+00 | 1.00E+00 | 1.00E+00 | 4.60E-02 | 1.63E-02 | 4.10E-02 | 4.10E-02 | rs2305929 | 5.95E-08 | √ |
| BIP | *RCOR2* | 51 | 1.00E+00 | 4.10E-02 | 3.99E-02 | 1.32E-01 | 3.20E-01 | 8.82E-02 | 8.82E-02 | rs4980532 | 8.73E-07 | √ |
| BIP | *RFXANK* | 81 | 1.00E+00 | 2.28E-01 | 8.66E-02 | 1.30E-03 | 1.10E-03 | 7.75E-04 | 7.75E-04 | rs111444407 | 2.34E-10 |  |
| BIP | *RHEBL1* | 41 | 1.00E+00 | 2.55E-02 | 2.20E-02 | 1.27E-01 | 1.14E-01 | 7.08E-02 | 7.08E-02 | rs7969091 | 2.96E-07 | √ |
| BIP | *RPS6KA2* | 416 | 1.00E+00 | 4.05E-02 | 9.29E-03 | 2.33E-01 | 2.41E-01 | 1.93E-01 | 1.00E+00 | rs10455979 | 4.38E-08 |  |
| BIP | *SAR1B* | 52 | 1.00E+00 | 1.00E+00 | 1.00E+00 | 1.25E-02 | 1.04E-02 | 1.25E-02 | 1.25E-02 | rs329319 | 1.43E-08 |  |
| BIP | *SEC11A* | 86 | 1.00E+00 | 1.45E-02 | 6.44E-03 | 2.86E-02 | 5.42E-02 | 1.47E-02 | 1.47E-02 | rs71395455 | 1.88E-08 |  |
| BIP | *SEC24A* | 40 | 1.00E+00 | 1.00E+00 | 1.00E+00 | 9.65E-03 | 7.72E-03 | 9.65E-03 | 9.65E-03 | rs329319 | 1.43E-08 |  |
| BIP | *SF3B2* | 71 | 1.00E+00 | 6.84E-02 | 2.76E-02 | 8.31E-02 | 2.75E-01 | 6.02E-02 | 6.02E-02 | rs7124165 | 3.53E-07 | √ |
| BIP | *SFMBT1* | 111 | 1.00E+00 | 6.65E-03 | 3.86E-03 | 9.07E-03 | 8.46E-03 | 5.00E-03 | 5.00E-03 | rs2071044 | 8.63E-09 |  |
| BIP | *SLC25A17* | 51 | 1.00E+00 | 3.28E-01 | 1.23E-01 | 2.04E-02 | 9.06E-03 | 1.93E-02 | 1.93E-02 | rs138321 | 4.25E-09 |  |
| BIP | *SLC44A2* | 72 | 1.00E+00 | 9.12E-03 | 7.14E-03 | 5.81E-02 | 9.49E-02 | 2.97E-02 | 2.97E-02 | rs3843751 | 1.46E-07 | √ |
| BIP | *SNAP23* | 43 | 1.00E+00 | 1.00E+00 | 1.00E+00 | 7.70E-02 | 3.76E-02 | 5.80E-02 | 5.80E-02 | rs11632169 | 1.06E-07 | √ |
| BIP | *SPCS1* | 80 | 1.00E+00 | 5.05E-03 | 4.91E-03 | 7.89E-03 | 2.55E-03 | 3.20E-03 | 3.20E-03 | rs2302417 | 5.51E-09 |  |
| BIP | *SSBP2* | 146 | 1.00E+00 | 7.31E-03 | 4.35E-03 | 1.44E-01 | 2.73E-01 | 8.35E-02 | 8.35E-02 | rs10035291 | 1.16E-07 | √ |
| BIP | *ST13* | 33 | 1.00E+00 | 3.20E-01 | 2.04E-01 | 1.32E-02 | 6.41E-03 | 1.25E-02 | 1.25E-02 | rs138321 | 4.25E-09 |  |
| BIP | *STAB1* | 85 | 1.00E+00 | 2.40E-02 | 1.09E-02 | 3.54E-02 | 2.62E-02 | 1.52E-02 | 1.52E-02 | rs10433615 | 3.03E-08 |  |
| BIP | *STARD9* | 87 | 1.00E+00 | 3.31E-02 | 2.74E-02 | 1.56E-01 | 6.40E-02 | 1.03E-01 | 1.03E-01 | rs11632169 | 1.06E-07 | √ |
| BIP | *STK4* | 113 | 1.00E+00 | 2.56E-03 | 1.33E-03 | 6.21E-01 | 5.09E-01 | 2.77E-01 | 2.77E-01 | rs6130764 | 5.52E-07 | √ |
| BIP | *SUGP1* | 70 | 1.00E+00 | 9.48E-02 | 6.93E-02 | 6.14E-04 | 7.81E-04 | 3.09E-04 | 3.09E-04 | rs111444407 | 2.34E-10 |  |
| BIP | *TERF2* | 74 | 1.00E+00 | 5.06E-02 | 4.19E-02 | 1.00E+00 | 1.00E+00 | 7.20E-01 | 7.20E-01 | rs7191614 | 2.93E-06 | √ |
| BIP | *TFAP2B* | 73 | 2.17E-03 | 2.90E-03 | 5.59E-01 | 2.31E-01 | 3.37E-01 | 1.42E-01 | 1.42E-01 | rs55648125 | 5.14E-08 | √ |
| BIP | *TM6SF2* | 67 | 1.00E+00 | 6.05E-02 | 3.93E-02 | 5.87E-04 | 7.82E-04 | 2.97E-04 | 2.97E-04 | rs111444407 | 2.34E-10 |  |
| BIP | *TMEM110* | 88 | 1.00E+00 | 3.74E-04 | 2.96E-04 | 5.36E-03 | 6.34E-03 | 2.50E-03 | 2.50E-03 | rs2302417 | 5.51E-09 |  |
| BIP | *TMEM110-MUSTN1* | 89 | 1.00E+00 | 3.65E-04 | 2.90E-04 | 5.42E-03 | 6.34E-03 | 2.52E-03 | 2.52E-03 | rs2302417 | 5.51E-09 |  |
| BIP | *TRANK1* | 89 | 1.00E+00 | 1.97E-06 | 4.01E-07 | 7.17E-08 | 7.17E-08 | 7.15E-08 | 7.15E-08 | rs9834970 | 4.79E-14 |  |
| BIP | *TSSK6* | 51 | 1.00E+00 | 2.24E-01 | 1.75E-01 | 5.37E-03 | 1.40E-02 | 3.77E-03 | 3.77E-03 | rs8110171 | 1.70E-08 |  |
| BIP | *VPS45* | 59 | 1.00E+00 | 3.14E-02 | 2.83E-02 | 5.75E-02 | 1.85E-01 | 3.68E-02 | 3.68E-02 | rs12063329 | 4.82E-07 | √ |
| BIP | *WDR73* | 68 | 1.00E+00 | 4.80E-03 | 3.09E-03 | 2.54E-02 | 4.79E-02 | 1.45E-02 | 1.45E-02 | rs71395455 | 1.88E-08 |  |
| BIP | *WFDC12* | 68 | 1.00E+00 | 2.22E-03 | 1.47E-03 | 3.74E-01 | 2.50E-01 | 1.65E-01 | 1.65E-01 | rs6130764 | 5.52E-07 | √ |
| BIP | *WFDC5* | 69 | 1.00E+00 | 1.70E-03 | 1.02E-03 | 3.79E-01 | 2.66E-01 | 1.68E-01 | 1.68E-01 | rs6130764 | 5.52E-07 | √ |
| BIP | *XPNPEP3* | 28 | 1.00E+00 | 4.58E-01 | 4.39E-01 | 1.12E-02 | 5.69E-03 | 1.06E-02 | 1.06E-02 | rs138321 | 4.25E-09 |  |
| BIP | *YJEFN3* | 57 | 1.00E+00 | 9.79E-02 | 6.82E-02 | 6.00E-03 | 1.66E-02 | 4.21E-03 | 4.21E-03 | rs8110171 | 1.70E-08 |  |
| BIP | *ZNF592* | 117 | 1.00E+00 | 8.72E-02 | 3.36E-02 | 4.39E-02 | 1.03E-01 | 2.58E-02 | 2.58E-02 | rs35630683 | 3.73E-08 |  |
| BIP | *ZPBP2* | 49 | 5.76E-06 | 7.42E-06 | 4.80E-01 | 1.00E+00 | 1.00E+00 | 1.00E+00 | 1.00E+00 | rs4794820 | 7.72E-07 | √ |
| CU | *ATP2A1* | 51 | 1.00E+00 | 4.97E-04 | 4.71E-04 | 5.83E-04 | 4.85E-04 | 1.91E-04 | 1.91E-04 | rs10499 | 9.66E-10 |  |
| CU | *ATXN2L* | 37 | 1.00E+00 | 1.54E-04 | 1.38E-04 | 4.23E-04 | 3.26E-04 | 1.39E-04 | 1.39E-04 | rs10499 | 9.66E-10 |  |
| CU | *CADM2* | 329 | 1.00E+00 | 9.93E-11 | 3.68E-11 | 1.26E-10 | 1.23E-09 | 5.87E-11 | 6.16E-11 | rs1368750 | 9.61E-17 |  |
| CU | *CD19* | 50 | 1.00E+00 | 5.63E-04 | 5.30E-04 | 6.01E-04 | 5.36E-04 | 2.07E-04 | 2.07E-04 | rs10499 | 9.66E-10 |  |
| CU | *LAT* | 32 | 1.00E+00 | 8.88E-02 | 3.58E-02 | 4.28E-04 | 4.37E-04 | 2.44E-04 | 2.44E-04 | rs10499 | 9.66E-10 |  |
| CU | *LRRTM4* | 355 | 1.00E+00 | 4.29E-02 | 2.22E-02 | 1.00E+00 | 1.00E+00 | 1.00E+00 | 1.00E+00 | rs7586062 | 1.44E-06 | √ |
| CU | *NCAM1* | 193 | 1.00E+00 | 5.08E-02 | 1.59E-02 | 9.22E-02 | 1.08E-01 | 5.73E-02 | 1.00E+00 | rs10891480 | 7.29E-10 |  |
| CU | *NFATC2IP* | 42 | 1.00E+00 | 1.12E-03 | 1.04E-03 | 5.62E-04 | 5.08E-04 | 2.09E-04 | 2.09E-04 | rs10499 | 9.66E-10 |  |
| CU | *RABEP2* | 54 | 1.00E+00 | 5.15E-04 | 4.89E-04 | 6.17E-04 | 5.25E-04 | 2.02E-04 | 2.02E-04 | rs10499 | 9.66E-10 |  |
| CU | *SGSM2* | 93 | 1.00E+00 | 1.00E+00 | 1.00E+00 | 6.58E-02 | 5.70E-02 | 3.64E-02 | 3.64E-02 | rs12451844 | 5.44E-08 | √ |
| CU | *SH2B1* | 44 | 1.00E+00 | 2.88E-04 | 2.75E-04 | 5.03E-04 | 4.05E-04 | 1.65E-04 | 1.65E-04 | rs10499 | 9.66E-10 |  |
| CU | *SMG6* | 175 | 1.00E+00 | 1.00E+00 | 1.00E+00 | 5.72E-02 | 3.35E-02 | 2.93E-02 | 2.93E-02 | rs17761723 | 1.94E-08 |  |
| CU | *SPNS1* | 34 | 1.00E+00 | 1.21E-02 | 7.48E-03 | 4.55E-04 | 4.55E-04 | 2.25E-04 | 2.25E-04 | rs10499 | 9.66E-10 |  |
| CU | *SRR* | 94 | 1.00E+00 | 1.00E+00 | 1.00E+00 | 6.66E-02 | 5.31E-02 | 3.23E-02 | 3.23E-02 | rs34264719 | 2.34E-08 |  |
| CU | *TSR1* | 88 | 1.00E+00 | 1.00E+00 | 1.00E+00 | 6.23E-02 | 5.17E-02 | 3.39E-02 | 3.39E-02 | rs112801087 | 3.17E-08 |  |
| CU | *TUFM* | 38 | 1.00E+00 | 1.67E-04 | 1.50E-04 | 4.34E-04 | 3.38E-04 | 1.42E-04 | 1.42E-04 | rs10499 | 9.66E-10 |  |
| MDD | *APOPT1* | 64 | 1.00E+00 | 3.67E-02 | 2.89E-02 | 5.71E-01 | 1.00E+00 | 3.38E-01 | 3.38E-01 | rs2756119 | 5.73E-07 | √ |
| MDD | *BAG5* | 58 | 1.00E+00 | 2.07E-02 | 1.64E-02 | 5.18E-01 | 1.00E+00 | 3.11E-01 | 3.10E-01 | rs2756119 | 5.73E-07 | √ |
| MDD | *BEND4* | 92 | 1.00E+00 | 1.47E-02 | 1.24E-02 | 7.01E-02 | 8.57E-02 | 3.29E-02 | 3.29E-02 | rs6832890 | 6.28E-08 | √ |
| MDD | *CKB* | 60 | 1.00E+00 | 3.23E-02 | 2.07E-02 | 5.95E-01 | 1.00E+00 | 3.36E-01 | 3.36E-01 | rs2756119 | 5.73E-07 | √ |
| MDD | *DCAF4L1* | 68 | 1.00E+00 | 2.84E-02 | 2.53E-02 | 8.88E-02 | 4.86E-02 | 3.26E-02 | 3.26E-02 | rs34215985 | 1.14E-07 | √ |
| MDD | *LRFN5* | 125 | 1.00E+00 | 1.92E-01 | 7.38E-02 | 3.93E-02 | 2.55E-02 | 1.70E-02 | 1.70E-02 | rs1950831 | 2.37E-08 |  |
| MDD | *NEGR1* | 259 | 1.00E+00 | 2.11E-01 | 8.91E-02 | 5.81E-02 | 3.80E-02 | 2.80E-02 | 2.80E-02 | rs7531118 | 1.86E-08 |  |
| MDD | *SHMT1* | 55 | 1.01E-05 | 1.50E-05 | 1.00E+00 | 1.00E+00 | 1.00E+00 | 1.00E+00 | 1.00E+00 | rs8068602 | 0.000965 | √ |
| MDD | *SLC30A9* | 100 | 1.00E+00 | 1.45E-02 | 1.28E-02 | 7.61E-02 | 6.68E-02 | 3.07E-02 | 3.07E-02 | rs6832890 | 6.28E-08 | √ |
| MDD | *SMCR8* | 59 | 1.17E-05 | 1.49E-05 | 1.00E+00 | 1.00E+00 | 1.00E+00 | 1.00E+00 | 1.00E+00 | rs8068602 | 0.000965 | √ |
| MDD | *TMEM33* | 67 | 1.00E+00 | 4.45E-02 | 3.76E-02 | 1.13E-01 | 1.43E-01 | 5.19E-02 | 5.19E-02 | rs34215985 | 1.14E-07 | √ |
| MDD | *TMX2-CTNND1* | 54 | 1.00E+00 | 7.78E-02 | 4.65E-02 | 5.58E-01 | 4.07E-01 | 2.04E-01 | 2.04E-01 | rs2509805 | 5.09E-07 | √ |
| MDD | *TRMT61A* | 62 | 1.00E+00 | 1.66E-02 | 1.13E-02 | 5.53E-01 | 1.00E+00 | 3.21E-01 | 3.21E-01 | rs2756119 | 5.73E-07 | √ |
| SCZ | *3-Sep* | 63 | 1.00E+00 | 1.19E-04 | 6.27E-05 | 1.67E-02 | 3.13E-02 | 9.30E-03 | 9.30E-03 | rs77441740 | 2.65E-08 |  |
| SCZ | *ABCB9* | 43 | 1.00E+00 | 2.79E-03 | 1.36E-03 | 5.01E-04 | 6.50E-04 | 2.37E-04 | 2.37E-04 | rs11608811 | 1.23E-09 |  |
| SCZ | *ACTR1A* | 79 | 1.00E+00 | 1.00E+00 | 1.00E+00 | 1.30E-02 | 1.04E-02 | 9.37E-03 | 9.37E-03 | rs10883735 | 1.59E-08 |  |
| SCZ | *ACTR5* | 98 | 1.00E+00 | 2.28E-06 | 5.71E-07 | 1.57E-04 | 1.42E-04 | 1.47E-04 | 1.47E-04 | rs6065094 | 9.53E-11 |  |
| SCZ | *ADAMTSL3* | 183 | 1.00E+00 | 1.69E-02 | 5.45E-03 | 2.99E-04 | 3.97E-04 | 1.71E-04 | 1.71E-04 | rs950169 | 1.41E-10 |  |
| SCZ | *AKT3* | 183 | 1.00E+00 | 1.63E-04 | 7.83E-05 | 8.02E-02 | 1.03E-01 | 3.37E-02 | 3.37E-02 | rs12093576 | 1.51E-08 |  |
| SCZ | *ALAS1* | 66 | 1.00E+00 | 1.00E+00 | 4.75E-01 | 3.62E-02 | 3.80E-02 | 1.98E-02 | 1.98E-02 | rs7622851 | 4.21E-08 |  |
| SCZ | *ALDOA* | 69 | 1.00E+00 | 6.07E-04 | 5.53E-04 | 2.26E-04 | 7.00E-04 | 1.44E-04 | 1.44E-04 | rs3814883 | 9.30E-11 |  |
| SCZ | *ALG12* | 53 | 1.00E+00 | 2.44E-02 | 2.14E-02 | 1.32E-01 | 1.72E-01 | 6.19E-02 | 6.19E-02 | rs5770755 | 4.91E-07 | √ |
| SCZ | *ALPK3* | 150 | 1.00E+00 | 1.00E+00 | 1.00E+00 | 1.65E-02 | 2.70E-02 | 1.10E-02 | 1.00E+00 | rs35828350 | 2.54E-09 |  |
| SCZ | *AMBRA1* | 67 | 1.00E+00 | 2.68E-05 | 2.25E-05 | 5.93E-05 | 1.20E-04 | 3.39E-05 | 3.39E-05 | rs7951870 | 1.67E-10 |  |
| SCZ | *ANKRD44* | 196 | 1.00E+00 | 5.80E-03 | 2.41E-03 | 8.58E-05 | 1.26E-04 | 6.25E-05 | 6.25E-05 | rs4685 | 4.12E-11 |  |
| SCZ | *ANKRD63* | 71 | 1.00E+00 | 8.48E-02 | 4.46E-02 | 8.97E-01 | 7.42E-01 | 5.08E-01 | 5.08E-01 | rs11857340 | 2.76E-07 | √ |
| SCZ | *ANKS1B* | 486 | 1.00E+00 | 5.30E-02 | 2.48E-02 | 1.00E+00 | 1.00E+00 | 7.07E-01 | 7.06E-01 | rs10735362 | 1.25E-06 | √ |
| SCZ | *ANP32E* | 59 | 1.00E+00 | 8.48E-04 | 3.79E-04 | 1.44E-03 | 2.71E-03 | 7.38E-04 | 7.38E-04 | rs1824850 | 1.20E-09 |  |
| SCZ | *AP3B2* | 82 | 1.00E+00 | 1.29E-03 | 8.46E-04 | 3.14E-02 | 2.35E-02 | 1.28E-02 | 1.28E-02 | rs783540 | 2.65E-08 |  |
| SCZ | *APH1A* | 57 | 1.00E+00 | 5.21E-02 | 3.41E-02 | 2.00E-03 | 2.70E-03 | 1.56E-03 | 1.56E-03 | rs78676616 | 3.25E-09 |  |
| SCZ | *APOPT1* | 64 | 1.00E+00 | 3.08E-04 | 2.44E-04 | 3.86E-07 | 7.16E-07 | 2.47E-07 | 2.47E-07 | rs35229468 | 5.95E-13 |  |
| SCZ | *ARHGAP1* | 25 | 1.00E+00 | 9.42E-05 | 6.85E-05 | 2.91E-04 | 2.46E-04 | 1.66E-04 | 1.66E-04 | rs7108770 | 2.47E-10 |  |
| SCZ | *ARHGAP40* | 104 | 1.00E+00 | 3.47E-02 | 1.87E-02 | 1.21E-02 | 1.12E-02 | 7.31E-03 | 7.31E-03 | rs6128857 | 6.34E-09 |  |
| SCZ | *ARL3* | 85 | 1.00E+00 | 5.09E-02 | 3.28E-02 | 1.45E-03 | 2.54E-03 | 6.91E-04 | 6.91E-04 | rs10883761 | 2.39E-09 |  |
| SCZ | *ARL5B* | 92 | 1.00E+00 | 1.00E+00 | 1.00E+00 | 5.14E-02 | 3.28E-02 | 5.10E-02 | 5.10E-02 | rs11511084 | 1.37E-08 |  |
| SCZ | *ARL6IP4* | 27 | 1.00E+00 | 3.19E-05 | 2.50E-05 | 3.14E-04 | 4.46E-04 | 1.49E-04 | 1.49E-04 | rs11608811 | 1.23E-09 |  |
| SCZ | *ARNT* | 67 | 1.00E+00 | 1.00E+00 | 1.00E+00 | 1.80E-01 | 4.42E-02 | 1.80E-01 | 1.80E-01 | rs116398299 | 8.08E-08 | √ |
| SCZ | *ARPC3* | 36 | 1.00E+00 | 4.13E-02 | 2.89E-02 | 8.37E-02 | 1.36E-01 | 6.22E-02 | 6.22E-02 | rs34840178 | 4.33E-07 | √ |
| SCZ | *AS3MT* | 83 | 1.00E+00 | 6.31E-10 | 3.24E-10 | 1.79E-11 | 3.10E-10 | 1.06E-11 | 1.12E-11 | rs7085104 | 1.30E-17 |  |
| SCZ | *ASPHD1* | 63 | 6.66E-01 | 1.07E-04 | 8.83E-05 | 1.94E-04 | 6.80E-04 | 1.06E-04 | 1.06E-04 | rs3814883 | 9.30E-11 |  |
| SCZ | *ATF4* | 41 | 1.00E+00 | 1.00E+00 | 1.00E+00 | 1.51E-02 | 9.81E-03 | 1.50E-02 | 1.50E-02 | rs5757717 | 7.76E-09 |  |
| SCZ | *ATG13* | 32 | 1.00E+00 | 3.15E-05 | 2.71E-05 | 8.55E-05 | 2.25E-04 | 6.02E-05 | 6.02E-05 | rs7108770 | 2.47E-10 |  |
| SCZ | *ATPAF2* | 36 | 1.00E+00 | 1.07E-03 | 9.94E-04 | 7.72E-03 | 9.31E-03 | 3.77E-03 | 3.77E-03 | rs4072738 | 2.21E-08 |  |
| SCZ | *ATXN7* | 102 | 1.00E+00 | 2.85E-04 | 1.15E-04 | 1.25E-03 | 1.06E-03 | 7.36E-04 | 7.36E-04 | rs832187 | 7.26E-10 |  |
| SCZ | *BAG5* | 58 | 1.00E+00 | 2.86E-04 | 2.30E-04 | 3.49E-07 | 6.50E-07 | 2.24E-07 | 2.24E-07 | rs35229468 | 5.95E-13 |  |
| SCZ | *BAP1* | 70 | 1.00E+00 | 1.00E+00 | 1.00E+00 | 8.79E-02 | 3.35E-02 | 5.04E-02 | 5.04E-02 | rs9311474 | 7.47E-08 | √ |
| SCZ | *BCL2L12* | 51 | 1.00E+00 | 7.14E-03 | 6.07E-03 | 5.60E-01 | 4.16E-01 | 2.40E-01 | 2.39E-01 | rs6509439 | 6.52E-07 | √ |
| SCZ | *BLM* | 82 | 1.00E+00 | 5.57E-02 | 2.35E-02 | 7.91E-06 | 7.82E-06 | 7.80E-06 | 7.80E-06 | rs4702 | 5.73E-12 |  |
| SCZ | *BOLL* | 78 | 1.00E+00 | 5.97E-04 | 3.74E-04 | 3.27E-03 | 8.69E-03 | 1.72E-03 | 1.72E-03 | rs9678520 | 1.25E-08 |  |
| SCZ | *BRD1* | 98 | 1.00E+00 | 4.21E-02 | 3.66E-02 | 2.43E-01 | 3.76E-01 | 1.13E-01 | 1.13E-01 | rs5770755 | 4.91E-07 | √ |
| SCZ | *BRE* | 175 | 1.00E+00 | 6.97E-02 | 4.76E-02 | 1.13E-01 | 8.96E-02 | 5.23E-02 | 5.23E-02 | rs12474906 | 7.70E-09 |  |
| SCZ | *C10orf32* | 78 | 1.00E+00 | 7.88E-11 | 4.94E-11 | 1.68E-11 | 2.91E-10 | 1.00E-11 | 9.34E-12 | rs7085104 | 1.30E-17 |  |
| SCZ | *C10orf95* | 69 | 1.00E+00 | 1.00E+00 | 1.00E+00 | 1.14E-02 | 1.07E-02 | 1.02E-02 | 1.02E-02 | rs10883735 | 1.59E-08 |  |
| SCZ | *C11orf49* | 42 | 3.05E-03 | 4.59E-03 | 8.54E-02 | 2.88E-01 | 3.49E-01 | 1.60E-01 | 1.60E-01 | rs10838634 | 6.96E-08 | √ |
| SCZ | *C11orf87* | 51 | 1.00E+00 | 1.99E-01 | 1.60E-01 | 2.48E-02 | 1.41E-02 | 2.28E-02 | 2.28E-02 | rs12421382 | 2.31E-08 |  |
| SCZ | *C12orf42* | 198 | 1.00E+00 | 1.96E-01 | 7.79E-02 | 3.33E-02 | 6.56E-02 | 2.64E-02 | 2.64E-02 | rs2139556 | 2.79E-08 |  |
| SCZ | *C12orf65* | 65 | 1.00E+00 | 1.70E-05 | 1.55E-05 | 1.98E-06 | 2.59E-06 | 1.15E-06 | 1.15E-06 | rs1615350 | 2.56E-12 |  |
| SCZ | *C14orf2* | 63 | 1.00E+00 | 9.07E-03 | 7.17E-03 | 3.04E-06 | 1.07E-05 | 2.54E-06 | 2.54E-06 | rs6576008 | 6.46E-12 |  |
| SCZ | *C16orf92* | 65 | 1.00E+00 | 3.85E-04 | 3.66E-04 | 1.88E-04 | 6.32E-04 | 1.17E-04 | 1.17E-04 | rs3814883 | 9.30E-11 |  |
| SCZ | *C1orf54* | 56 | 1.00E+00 | 2.45E-01 | 1.76E-01 | 1.64E-02 | 1.96E-02 | 8.37E-03 | 8.37E-03 | rs72694961 | 4.49E-09 |  |
| SCZ | *C20orf195* | 66 | 1.00E+00 | 2.88E-02 | 1.27E-02 | 1.00E+00 | 1.00E+00 | 6.19E-01 | 6.19E-01 | rs200050069 | 9.17E-08 | √ |
| SCZ | *C2orf47* | 57 | 1.00E+00 | 1.16E-05 | 4.71E-06 | 3.27E-08 | 1.72E-07 | 2.26E-08 | 2.26E-08 | rs281785 | 1.54E-13 |  |
| SCZ | *C2orf69* | 68 | 1.00E+00 | 9.31E-06 | 4.48E-06 | 3.90E-08 | 2.05E-07 | 2.70E-08 | 2.70E-08 | rs2949006 | 3.17E-14 |  |
| SCZ | *C2orf82* | 87 | 3.22E-01 | 1.38E-05 | 8.09E-06 | 7.02E-06 | 1.54E-05 | 3.49E-06 | 3.49E-06 | rs62193339 | 6.55E-12 |  |
| SCZ | *C3orf49* | 45 | 1.00E+00 | 6.27E-05 | 1.92E-05 | 5.49E-04 | 4.89E-04 | 3.48E-04 | 3.48E-04 | rs832187 | 7.26E-10 |  |
| SCZ | *C4orf27* | 59 | 1.00E+00 | 2.22E-01 | 1.12E-01 | 4.20E-02 | 1.02E-01 | 2.98E-02 | 2.98E-02 | rs10520163 | 1.62E-07 | √ |
| SCZ | *CA14* | 57 | 1.00E+00 | 2.61E-02 | 1.69E-02 | 2.00E-03 | 2.74E-03 | 1.56E-03 | 1.56E-03 | rs1824850 | 1.20E-09 |  |
| SCZ | *CACNA1C* | 386 | 1.00E+00 | 1.11E-13 | 1.39E-14 | 1.33E-11 | 1.44E-09 | 8.35E-12 | 9.34E-12 | rs1024582 | 3.02E-18 |  |
| SCZ | *CACNA1I* | 56 | 1.00E+00 | 1.00E+00 | 1.00E+00 | 2.06E-02 | 1.63E-02 | 2.05E-02 | 2.05E-02 | rs5757717 | 7.76E-09 |  |
| SCZ | *CACNB2* | 329 | 1.00E+00 | 4.52E-04 | 8.10E-05 | 4.36E-04 | 4.18E-04 | 3.40E-04 | 3.40E-04 | rs7893279 | 7.88E-11 |  |
| SCZ | *CDC25C* | 67 | 1.00E+00 | 1.00E+00 | 1.00E+00 | 4.37E-02 | 6.33E-02 | 3.35E-02 | 3.35E-02 | rs10043984 | 1.04E-07 | √ |
| SCZ | *CDIPT* | 44 | 1.00E+00 | 3.56E-03 | 2.56E-03 | 2.43E-04 | 5.28E-04 | 1.33E-04 | 1.33E-04 | rs4788197 | 8.38E-10 |  |
| SCZ | *CDK2AP1* | 57 | 1.00E+00 | 1.78E-05 | 1.58E-05 | 2.31E-06 | 2.31E-06 | 1.25E-06 | 1.25E-06 | rs1615350 | 2.56E-12 |  |
| SCZ | *CENPM* | 61 | 1.00E+00 | 2.56E-04 | 1.17E-04 | 1.62E-02 | 3.33E-02 | 1.17E-02 | 1.17E-02 | rs77441740 | 2.65E-08 |  |
| SCZ | *CEP170* | 56 | 1.00E+00 | 1.70E-02 | 1.43E-02 | 8.64E-02 | 7.90E-02 | 3.88E-02 | 3.88E-02 | rs3943093 | 1.85E-07 | √ |
| SCZ | *CHADL* | 50 | 1.00E+00 | 6.06E-02 | 1.97E-02 | 1.48E-03 | 3.11E-03 | 1.01E-03 | 1.01E-03 | rs9607782 | 2.86E-12 |  |
| SCZ | *CILP2* | 58 | 1.00E+00 | 2.30E-04 | 1.12E-04 | 2.51E-03 | 7.00E-03 | 1.63E-03 | 1.63E-03 | rs7245983 | 1.03E-08 |  |
| SCZ | *CKAP5* | 37 | 1.00E+00 | 1.77E-03 | 1.52E-03 | 8.69E-04 | 5.92E-04 | 8.51E-04 | 8.51E-04 | rs7109698 | 1.40E-09 |  |
| SCZ | *CKB* | 60 | 1.00E+00 | 1.93E-04 | 1.23E-04 | 3.62E-07 | 6.71E-07 | 2.32E-07 | 2.32E-07 | rs35229468 | 5.95E-13 |  |
| SCZ | *CLCN3* | 81 | 1.00E+00 | 2.04E-01 | 1.20E-01 | 5.13E-02 | 1.26E-01 | 3.22E-02 | 3.22E-02 | rs7696555 | 1.54E-07 | √ |
| SCZ | *CLP1* | 53 | 1.00E+00 | 7.76E-02 | 2.28E-02 | 2.39E-01 | 2.51E-01 | 1.32E-01 | 1.32E-01 | rs9420 | 5.23E-07 | √ |
| SCZ | *CNNM2* | 128 | 1.00E+00 | 1.18E-09 | 5.00E-10 | 2.76E-11 | 4.78E-10 | 1.45E-11 | 1.49E-11 | rs7085104 | 1.30E-17 |  |
| SCZ | *CNTN2* | 105 | 1.00E+00 | 1.78E-02 | 7.18E-03 | 1.00E+00 | 1.00E+00 | 1.00E+00 | 1.00E+00 | rs11240341 | 1.02E-06 | √ |
| SCZ | *CNTN4* | 791 | 1.00E+00 | 6.33E-01 | 1.87E-01 | 1.99E-04 | 1.96E-04 | 1.13E-04 | 1.00E+00 | rs17194490 | 1.49E-11 |  |
| SCZ | *COQ10B* | 45 | 1.00E+00 | 4.96E-06 | 1.62E-06 | 1.18E-05 | 2.60E-05 | 6.62E-06 | 6.62E-06 | rs4685 | 4.12E-11 |  |
| SCZ | *CPEB1* | 64 | 1.00E+00 | 1.06E-03 | 9.53E-04 | 2.45E-02 | 1.57E-02 | 9.35E-03 | 9.35E-03 | rs783540 | 2.65E-08 |  |
| SCZ | *CREB3L1* | 49 | 1.00E+00 | 7.89E-05 | 3.59E-05 | 1.38E-04 | 1.29E-04 | 6.75E-05 | 6.75E-05 | rs7951870 | 1.67E-10 |  |
| SCZ | *CRELD2* | 49 | 1.00E+00 | 2.35E-02 | 2.07E-02 | 1.22E-01 | 1.67E-01 | 6.12E-02 | 6.12E-02 | rs5770755 | 4.91E-07 | √ |
| SCZ | *CTSK* | 61 | 1.00E+00 | 1.00E+00 | 1.00E+00 | 1.64E-01 | 4.28E-02 | 1.64E-01 | 1.63E-01 | rs116398299 | 8.08E-08 | √ |
| SCZ | *CTSS* | 73 | 1.00E+00 | 1.00E+00 | 1.00E+00 | 1.96E-01 | 4.66E-02 | 1.96E-01 | 1.96E-01 | rs114845445 | 1.29E-07 | √ |
| SCZ | *CUEDC2* | 68 | 1.00E+00 | 1.00E+00 | 1.00E+00 | 2.24E-02 | 1.38E-02 | 2.23E-02 | 2.23E-02 | rs17114641 | 1.96E-08 |  |
| SCZ | *CUL3* | 127 | 1.00E+00 | 1.00E+00 | 1.00E+00 | 3.23E-02 | 3.04E-02 | 2.24E-02 | 2.24E-02 | rs11685299 | 2.61E-08 |  |
| SCZ | *CXXC4* | 60 | 3.90E-03 | 5.91E-03 | 1.00E+00 | 1.00E+00 | 1.00E+00 | 1.00E+00 | 1.00E+00 | rs2905627 | 6.27E-07 | √ |
| SCZ | *CYP17A1* | 81 | 1.00E+00 | 4.93E-10 | 3.58E-10 | 1.75E-11 | 3.03E-10 | 1.04E-11 | 1.12E-11 | rs7085104 | 1.30E-17 |  |
| SCZ | *CYP27C1* | 110 | 1.25E-04 | 7.44E-04 | 1.00E+00 | 1.00E+00 | 1.00E+00 | 1.00E+00 | 1.00E+00 | rs6740232 | 0.001359 | √ |
| SCZ | *CYP2D6* | 52 | 1.00E+00 | 6.41E-04 | 5.57E-04 | 1.31E-02 | 1.17E-02 | 5.98E-03 | 5.98E-03 | rs6002655 | 2.31E-10 |  |
| SCZ | *DCLK3* | 111 | 7.28E-01 | 2.56E-03 | 8.76E-04 | 1.08E-05 | 3.02E-05 | 7.05E-06 | 7.05E-06 | rs9876421 | 7.82E-12 |  |
| SCZ | *DDX56* | 46 | 1.00E+00 | 4.69E-02 | 3.66E-02 | 1.00E+00 | 1.00E+00 | 6.96E-01 | 6.96E-01 | rs217384 | 8.40E-06 | √ |
| SCZ | *DGKI* | 194 | 1.00E+00 | 2.79E-03 | 3.85E-04 | 1.19E-02 | 4.72E-02 | 7.68E-03 | 7.68E-03 | rs10954580 | 1.78E-08 |  |
| SCZ | *DGKZ* | 46 | 1.00E+00 | 4.26E-05 | 2.49E-05 | 7.33E-05 | 1.09E-04 | 3.80E-05 | 3.80E-05 | rs7951870 | 1.67E-10 |  |
| SCZ | *DNAH1* | 94 | 1.00E+00 | 1.00E+00 | 1.00E+00 | 5.16E-02 | 4.01E-02 | 2.73E-02 | 2.73E-02 | rs7622851 | 4.21E-08 |  |
| SCZ | *DNAJC19* | 45 | 1.00E+00 | 1.22E-04 | 1.13E-04 | 2.49E-04 | 8.72E-04 | 1.54E-04 | 1.54E-04 | rs13071962 | 1.64E-09 |  |
| SCZ | *DOC2A* | 73 | 1.00E+00 | 1.85E-04 | 1.80E-04 | 2.11E-04 | 7.06E-04 | 1.17E-04 | 1.17E-04 | rs3814883 | 9.30E-11 |  |
| SCZ | *DOPEY2* | 134 | 1.00E+00 | 1.66E-02 | 4.93E-03 | 1.00E+00 | 1.00E+00 | 1.00E+00 | 1.00E+00 | rs2298450 | 4.25E-06 | √ |
| SCZ | *DPP4* | 75 | 1.00E+00 | 4.33E-01 | 1.38E-01 | 6.04E-02 | 3.59E-02 | 4.02E-02 | 4.02E-02 | rs4340536 | 3.90E-08 |  |
| SCZ | *DPYD* | 461 | 1.00E+00 | 4.21E-09 | 5.26E-10 | 3.60E-08 | 7.22E-08 | 1.75E-08 | 1.00E+00 | rs2391902 | 9.31E-15 |  |
| SCZ | *DRD2* | 128 | 1.00E+00 | 2.58E-05 | 6.81E-06 | 1.15E-03 | 9.97E-04 | 1.10E-03 | 1.10E-03 | rs12420205 | 1.22E-10 |  |
| SCZ | *DRG2* | 58 | 1.00E+00 | 2.59E-02 | 1.56E-02 | 2.49E-02 | 2.64E-02 | 1.59E-02 | 1.59E-02 | rs9899355 | 4.84E-08 |  |
| SCZ | *EEF1A2* | 73 | 1.00E+00 | 3.38E-02 | 1.53E-02 | 1.00E+00 | 1.00E+00 | 6.85E-01 | 6.85E-01 | rs200050069 | 9.17E-08 | √ |
| SCZ | *EFHD1* | 105 | 1.00E+00 | 4.13E-03 | 9.41E-04 | 4.69E-06 | 6.98E-06 | 2.65E-06 | 2.65E-06 | rs4144795 | 3.29E-12 |  |
| SCZ | *EGR1* | 57 | 1.00E+00 | 3.85E-03 | 1.09E-03 | 9.61E-03 | 8.15E-03 | 5.70E-03 | 5.70E-03 | rs3849046 | 1.22E-08 |  |
| SCZ | *ELFN1* | 76 | 1.00E+00 | 9.18E-01 | 4.74E-01 | 5.60E-02 | 1.39E-01 | 4.12E-02 | 1.00E+00 | rs10950399 | 3.44E-08 |  |
| SCZ | *EP300* | 69 | 1.00E+00 | 1.35E-02 | 1.03E-02 | 1.36E-03 | 2.68E-03 | 8.49E-04 | 8.49E-04 | rs9607782 | 2.86E-12 |  |
| SCZ | *EPB41* | 133 | 1.00E+00 | 8.42E-01 | 5.24E-01 | 8.85E-02 | 4.22E-02 | 8.53E-02 | 8.53E-02 | rs533123 | 3.96E-08 |  |
| SCZ | *ESAM* | 70 | 1.00E+00 | 1.91E-05 | 5.06E-06 | 2.29E-02 | 1.89E-02 | 9.32E-03 | 9.32E-03 | rs55661361 | 2.11E-11 |  |
| SCZ | *ESRP2* | 60 | 1.00E+00 | 1.08E-01 | 8.43E-02 | 2.90E-02 | 4.18E-02 | 1.75E-02 | 1.75E-02 | rs10852439 | 7.45E-08 | √ |
| SCZ | *ETF1* | 61 | 1.87E-05 | 7.60E-08 | 1.48E-04 | 1.03E-02 | 9.16E-03 | 6.70E-03 | 6.70E-03 | rs3849046 | 1.22E-08 |  |
| SCZ | *F2* | 29 | 1.00E+00 | 2.30E-04 | 1.69E-04 | 3.38E-04 | 2.73E-04 | 2.11E-04 | 2.11E-04 | rs7108770 | 2.47E-10 |  |
| SCZ | *FAM109B* | 50 | 1.00E+00 | 2.02E-03 | 1.56E-03 | 2.22E-02 | 3.04E-02 | 1.11E-02 | 1.11E-02 | rs760648 | 5.02E-09 |  |
| SCZ | *FAM124B* | 119 | 1.00E+00 | 1.00E+00 | 1.00E+00 | 4.75E-02 | 3.34E-02 | 3.46E-02 | 3.46E-02 | rs4674916 | 2.75E-08 |  |
| SCZ | *FAM216A* | 35 | 1.00E+00 | 2.79E-02 | 1.81E-02 | 8.14E-02 | 1.37E-01 | 5.81E-02 | 5.81E-02 | rs34840178 | 4.33E-07 | √ |
| SCZ | *FAM53C* | 65 | 1.00E+00 | 3.65E-01 | 2.29E-01 | 4.24E-02 | 6.14E-02 | 3.24E-02 | 3.24E-02 | rs10043984 | 1.04E-07 | √ |
| SCZ | *FAM57B* | 66 | 1.00E+00 | 3.88E-04 | 3.71E-04 | 1.91E-04 | 6.45E-04 | 1.19E-04 | 1.19E-04 | rs3814883 | 9.30E-11 |  |
| SCZ | *FAM83D* | 72 | 1.00E+00 | 1.00E+00 | 1.00E+00 | 7.07E-03 | 3.91E-03 | 6.59E-03 | 6.59E-03 | rs34431148 | 4.23E-09 |  |
| SCZ | *FANCL* | 81 | 1.00E+00 | 3.34E-02 | 2.09E-02 | 1.73E-01 | 1.04E-01 | 1.42E-01 | 1.42E-01 | rs12713372 | 4.31E-08 |  |
| SCZ | *FCGRT* | 64 | 1.00E+00 | 4.73E-02 | 3.57E-02 | 7.02E-01 | 4.21E-01 | 3.20E-01 | 3.20E-01 | rs6509439 | 6.52E-07 | √ |
| SCZ | *FES* | 70 | 1.00E+00 | 1.23E-03 | 1.89E-04 | 6.75E-06 | 6.65E-06 | 6.66E-06 | 6.66E-06 | rs4702 | 5.73E-12 |  |
| SCZ | *FOXP1* | 220 | 1.00E+00 | 1.18E-03 | 1.62E-04 | 7.21E-01 | 5.57E-01 | 4.32E-01 | 4.32E-01 | rs62244881 | 1.17E-08 |  |
| SCZ | *FTSJ2* | 71 | 1.00E+00 | 1.99E-02 | 6.31E-03 | 1.37E-02 | 2.26E-02 | 9.97E-03 | 9.97E-03 | rs3778996 | 1.97E-08 |  |
| SCZ | *FURIN* | 76 | 1.00E+00 | 6.76E-03 | 1.39E-03 | 7.33E-06 | 7.22E-06 | 7.23E-06 | 7.23E-06 | rs4702 | 5.73E-12 |  |
| SCZ | *FUT9* | 153 | 1.00E+00 | 1.00E+00 | 1.00E+00 | 3.70E-02 | 7.16E-02 | 3.41E-02 | 3.41E-02 | rs910025 | 1.25E-08 |  |
| SCZ | *FXR1* | 61 | 1.00E+00 | 4.84E-05 | 4.21E-05 | 3.04E-04 | 1.23E-03 | 1.92E-04 | 1.92E-04 | rs13071962 | 1.64E-09 |  |
| SCZ | *GALNT10* | 224 | 1.00E+00 | 3.85E-02 | 2.02E-02 | 1.02E-01 | 2.38E-01 | 5.68E-02 | 5.68E-02 | rs514699 | 8.29E-08 | √ |
| SCZ | *GATAD2A* | 74 | 1.00E+00 | 6.73E-04 | 4.18E-04 | 2.78E-03 | 6.46E-03 | 1.40E-03 | 1.40E-03 | rs7245983 | 1.03E-08 |  |
| SCZ | *GDPD3* | 48 | 1.00E+00 | 1.64E-02 | 1.46E-02 | 2.17E-03 | 1.48E-03 | 1.44E-03 | 1.44E-03 | rs3814880 | 2.76E-09 |  |
| SCZ | *GIGYF2* | 129 | 1.00E+00 | 2.88E-06 | 1.84E-06 | 5.20E-06 | 8.47E-06 | 2.10E-06 | 2.10E-06 | rs4144795 | 3.29E-12 |  |
| SCZ | *GLT8D1* | 82 | 1.00E+00 | 6.59E-03 | 6.41E-03 | 1.43E-04 | 1.38E-04 | 1.19E-04 | 1.19E-04 | rs4481150 | 2.31E-10 |  |
| SCZ | *GLYCTK* | 71 | 1.00E+00 | 1.00E+00 | 1.00E+00 | 3.90E-02 | 3.18E-02 | 2.07E-02 | 2.07E-02 | rs7622851 | 4.21E-08 |  |
| SCZ | *GMEB1* | 46 | 1.00E+00 | 1.00E+00 | 1.00E+00 | 3.06E-02 | 1.37E-02 | 3.02E-02 | 3.02E-02 | rs533123 | 3.96E-08 |  |
| SCZ | *GMIP* | 76 | 1.00E+00 | 1.31E-02 | 8.17E-03 | 8.74E-03 | 1.23E-02 | 6.54E-03 | 6.54E-03 | rs7245983 | 1.03E-08 |  |
| SCZ | *GNL3* | 81 | 1.00E+00 | 8.41E-03 | 8.18E-03 | 2.34E-04 | 1.61E-04 | 1.93E-04 | 1.93E-04 | rs2710323 | 2.93E-10 |  |
| SCZ | *GPN3* | 35 | 1.00E+00 | 2.39E-02 | 1.62E-02 | 8.14E-02 | 1.38E-01 | 5.81E-02 | 5.81E-02 | rs34840178 | 4.33E-07 | √ |
| SCZ | *GRM3* | 118 | 1.00E+00 | 1.00E+00 | 8.99E-01 | 1.80E-02 | 1.73E-02 | 1.23E-02 | 1.23E-02 | rs12704290 | 3.65E-10 |  |
| SCZ | *GSDMB* | 63 | 4.38E-03 | 1.07E-02 | 1.00E+00 | 1.00E+00 | 1.00E+00 | 1.00E+00 | 1.00E+00 | rs72832972 | 0.000863 | √ |
| SCZ | *HAPLN4* | 71 | 1.00E+00 | 1.36E-02 | 8.01E-03 | 1.47E-02 | 2.98E-02 | 8.87E-03 | 8.87E-03 | rs67720221 | 4.19E-08 |  |
| SCZ | *HARBI1* | 26 | 1.00E+00 | 2.90E-05 | 2.71E-05 | 6.95E-05 | 1.75E-04 | 4.89E-05 | 4.89E-05 | rs7108770 | 2.47E-10 |  |
| SCZ | *HARS* | 72 | 1.00E+00 | 5.52E-02 | 4.98E-02 | 2.53E-01 | 3.43E-01 | 1.11E-01 | 1.11E-01 | rs62384243 | 7.96E-07 | √ |
| SCZ | *HARS2* | 76 | 1.00E+00 | 4.91E-02 | 4.45E-02 | 2.08E-01 | 3.39E-01 | 1.03E-01 | 1.03E-01 | rs62384243 | 7.96E-07 | √ |
| SCZ | *HDDC3* | 59 | 1.00E+00 | 1.12E-02 | 3.33E-03 | 5.69E-06 | 5.61E-06 | 5.61E-06 | 5.61E-06 | rs4702 | 5.73E-12 |  |
| SCZ | *HIRIP3* | 73 | 1.00E+00 | 1.62E-04 | 1.57E-04 | 2.11E-04 | 7.15E-04 | 1.17E-04 | 1.17E-04 | rs3814883 | 9.30E-11 |  |
| SCZ | *HIST1H1A* | 36 | 1.00E+00 | 2.05E-03 | 1.93E-03 | 1.26E-14 | 1.34E-10 | 1.26E-14 | 1.26E-14 | rs35050608 | 5.08E-21 |  |
| SCZ | *HIST1H1C* | 19 | 1.00E+00 | 4.52E-06 | 1.83E-06 | 6.64E-15 | 7.10E-11 | 6.64E-15 | 6.64E-15 | rs35050608 | 5.08E-21 |  |
| SCZ | *HIST1H2AA* | 86 | 1.00E+00 | 1.85E-05 | 4.06E-06 | 8.77E-12 | 3.21E-10 | 7.08E-12 | 7.47E-12 | rs13201341 | 1.21E-19 |  |
| SCZ | *HIST1H2AB* | 28 | 1.00E+00 | 8.69E-03 | 8.21E-03 | 9.78E-15 | 1.05E-10 | 9.78E-15 | 9.78E-15 | rs35050608 | 5.08E-21 |  |
| SCZ | *HIST1H2BA* | 86 | 1.00E+00 | 1.85E-05 | 4.06E-06 | 8.77E-12 | 3.21E-10 | 7.08E-12 | 7.47E-12 | rs13201341 | 1.21E-19 |  |
| SCZ | *HIST1H2BB* | 23 | 1.00E+00 | 3.07E-04 | 1.71E-04 | 8.04E-15 | 8.59E-11 | 8.03E-15 | 8.04E-15 | rs35050608 | 5.08E-21 |  |
| SCZ | *HIST1H3A* | 33 | 1.00E+00 | 4.26E-04 | 3.88E-04 | 1.15E-14 | 1.23E-10 | 1.15E-14 | 1.15E-14 | rs35050608 | 5.08E-21 |  |
| SCZ | *HIST1H3B* | 29 | 1.00E+00 | 1.07E-03 | 9.99E-04 | 1.01E-14 | 1.08E-10 | 1.01E-14 | 1.01E-14 | rs35050608 | 5.08E-21 |  |
| SCZ | *HIST1H3C* | 21 | 1.00E+00 | 7.61E-05 | 6.48E-05 | 7.34E-15 | 7.84E-11 | 7.34E-15 | 7.34E-15 | rs35050608 | 5.08E-21 |  |
| SCZ | *HIST1H4A* | 33 | 1.00E+00 | 4.26E-04 | 3.88E-04 | 1.15E-14 | 1.23E-10 | 1.15E-14 | 1.15E-14 | rs35050608 | 5.08E-21 |  |
| SCZ | *HIST1H4B* | 30 | 1.00E+00 | 1.01E-03 | 9.37E-04 | 1.05E-14 | 1.12E-10 | 1.05E-14 | 1.05E-14 | rs35050608 | 5.08E-21 |  |
| SCZ | *HSPA9* | 49 | 6.13E-01 | 3.41E-04 | 3.16E-04 | 8.26E-03 | 7.63E-03 | 5.44E-03 | 5.44E-03 | rs3849046 | 1.22E-08 |  |
| SCZ | *HSPD1* | 43 | 1.00E+00 | 8.08E-06 | 2.67E-06 | 1.13E-05 | 2.48E-05 | 6.34E-06 | 6.34E-06 | rs4685 | 4.12E-11 |  |
| SCZ | *HSPE1* | 43 | 1.00E+00 | 5.54E-06 | 1.96E-06 | 1.41E-05 | 4.17E-05 | 7.88E-06 | 7.88E-06 | rs6434928 | 6.93E-11 |  |
| SCZ | *IGSF9B* | 124 | 1.00E+00 | 9.09E-05 | 4.91E-05 | 8.52E-05 | 8.20E-05 | 3.98E-05 | 3.98E-05 | rs3802924 | 4.11E-11 |  |
| SCZ | *IMMP2L* | 295 | 1.00E+00 | 4.45E-04 | 1.56E-04 | 1.11E-05 | 2.00E-05 | 4.65E-06 | 1.00E+00 | rs12532143 | 2.13E-12 |  |
| SCZ | *INA* | 40 | 1.00E+00 | 3.26E-02 | 3.13E-02 | 4.67E-10 | 4.48E-10 | 4.27E-10 | 4.05E-10 | rs112913898 | 3.13E-16 |  |
| SCZ | *INO80E* | 73 | 1.00E+00 | 1.76E-04 | 1.71E-04 | 2.11E-04 | 7.10E-04 | 1.17E-04 | 1.17E-04 | rs3814883 | 9.30E-11 |  |
| SCZ | *IREB2* | 83 | 1.00E+00 | 9.18E-03 | 6.49E-03 | 1.56E-05 | 1.67E-05 | 8.66E-06 | 8.66E-06 | rs1700006 | 1.21E-12 |  |
| SCZ | *IRF3* | 46 | 1.00E+00 | 6.39E-03 | 5.53E-03 | 5.05E-01 | 3.65E-01 | 2.16E-01 | 2.16E-01 | rs6509439 | 6.52E-07 | √ |
| SCZ | *ITIH1* | 88 | 1.00E+00 | 2.73E-04 | 2.43E-04 | 1.53E-04 | 2.09E-04 | 9.61E-05 | 9.61E-05 | rs4481150 | 2.31E-10 |  |
| SCZ | *ITIH3* | 84 | 1.00E+00 | 1.46E-04 | 1.26E-04 | 1.46E-04 | 2.13E-04 | 9.25E-05 | 9.25E-05 | rs4481150 | 2.31E-10 |  |
| SCZ | *ITIH4* | 76 | 1.00E+00 | 7.38E-05 | 5.90E-05 | 1.32E-04 | 2.10E-04 | 8.37E-05 | 8.37E-05 | rs4481150 | 2.31E-10 |  |
| SCZ | *JAM3* | 116 | 1.00E+00 | 1.22E-02 | 9.68E-03 | 1.33E-04 | 1.73E-04 | 8.72E-05 | 8.72E-05 | rs4936216 | 4.68E-11 |  |
| SCZ | *KCNB1* | 144 | 1.00E+00 | 5.98E-03 | 1.07E-03 | 1.74E-01 | 1.69E-01 | 8.09E-02 | 8.09E-02 | rs7267348 | 8.34E-08 | √ |
| SCZ | *KCNJ13* | 76 | 1.00E+00 | 2.15E-04 | 1.69E-04 | 3.39E-06 | 4.93E-06 | 1.53E-06 | 1.53E-06 | rs4144795 | 3.29E-12 |  |
| SCZ | *KCNQ2* | 83 | 1.00E+00 | 1.46E-02 | 5.01E-03 | 1.00E+00 | 1.00E+00 | 7.76E-01 | 7.76E-01 | rs200050069 | 9.17E-08 | √ |
| SCZ | *KCTD13* | 77 | 1.00E+00 | 1.21E-04 | 1.15E-04 | 2.23E-04 | 7.95E-04 | 1.23E-04 | 1.23E-04 | rs3814883 | 9.30E-11 |  |
| SCZ | *KDM3B* | 85 | 1.00E+00 | 1.00E-02 | 4.04E-03 | 1.43E-02 | 1.09E-02 | 7.72E-03 | 7.72E-03 | rs3849046 | 1.22E-08 |  |
| SCZ | *KDM4A* | 102 | 1.00E+00 | 6.39E-01 | 3.81E-01 | 7.90E-03 | 4.57E-03 | 5.68E-03 | 5.68E-03 | rs11210892 | 4.60E-09 |  |
| SCZ | *KIAA1324L* | 131 | 1.00E+00 | 1.00E+00 | 1.00E+00 | 3.99E-02 | 2.15E-02 | 2.44E-02 | 2.44E-02 | rs12704290 | 3.65E-10 |  |
| SCZ | *KLC1* | 87 | 1.00E+00 | 1.76E-07 | 7.91E-08 | 5.24E-07 | 9.68E-07 | 3.15E-07 | 3.15E-07 | rs35229468 | 5.95E-13 |  |
| SCZ | *L3MBTL2* | 54 | 1.00E+00 | 5.62E-02 | 4.16E-02 | 1.60E-03 | 3.03E-03 | 1.09E-03 | 1.09E-03 | rs9607782 | 2.86E-12 |  |
| SCZ | *LHFPL3* | 324 | 1.00E+00 | 1.00E+00 | 1.00E+00 | 2.27E-02 | 1.78E-02 | 2.25E-02 | 1.00E+00 | rs2428162 | 2.33E-09 |  |
| SCZ | *LPAR2* | 69 | 1.00E+00 | 7.76E-03 | 4.91E-03 | 7.93E-03 | 1.11E-02 | 5.94E-03 | 5.94E-03 | rs7245983 | 1.03E-08 |  |
| SCZ | *LRP1* | 86 | 1.00E+00 | 1.00E+00 | 1.00E+00 | 4.78E-02 | 2.84E-02 | 3.83E-02 | 3.83E-02 | rs61937595 | 2.39E-12 |  |
| SCZ | *LRP4* | 23 | 1.00E+00 | 2.24E-02 | 2.02E-02 | 9.38E-02 | 1.42E-01 | 5.16E-02 | 5.16E-02 | rs10838634 | 6.96E-08 | √ |
| SCZ | *LRRC16A* | 313 | 1.00E+00 | 3.11E-01 | 1.18E-01 | 3.95E-10 | 1.17E-09 | 3.23E-10 | 3.55E-10 | rs34493019 | 1.42E-19 |  |
| SCZ | *LRRC3C* | 72 | 1.99E-02 | 5.49E-02 | 1.00E+00 | 1.00E+00 | 1.00E+00 | 1.00E+00 | 1.00E+00 | rs72832972 | 0.000863 | √ |
| SCZ | *LRRC48* | 29 | 1.00E+00 | 1.01E-03 | 9.27E-04 | 6.22E-03 | 7.85E-03 | 3.20E-03 | 3.20E-03 | rs4072738 | 2.21E-08 |  |
| SCZ | *LRRN3* | 63 | 1.00E+00 | 3.84E-01 | 3.05E-01 | 5.16E-06 | 8.37E-06 | 3.41E-06 | 3.41E-06 | rs214467 | 8.13E-12 |  |
| SCZ | *MAD1L1* | 199 | 1.00E+00 | 3.23E-05 | 4.04E-06 | 1.76E-06 | 1.75E-06 | 1.16E-06 | 1.16E-06 | rs58120505 | 9.28E-14 |  |
| SCZ | *MAN2A2* | 70 | 1.00E+00 | 2.14E-03 | 3.25E-04 | 6.75E-06 | 6.66E-06 | 6.66E-06 | 6.66E-06 | rs4702 | 5.73E-12 |  |
| SCZ | *MAP3K9* | 153 | 1.00E+00 | 1.00E+00 | 1.00E+00 | 1.14E-02 | 1.40E-02 | 9.04E-03 | 9.04E-03 | rs2189807 | 7.13E-09 |  |
| SCZ | *MAPK3* | 41 | 1.00E+00 | 3.69E-02 | 3.30E-02 | 1.08E-01 | 1.71E-01 | 8.50E-02 | 8.50E-02 | rs11642740 | 4.76E-07 | √ |
| SCZ | *MARK3* | 101 | 1.00E+00 | 2.17E-04 | 5.77E-05 | 6.09E-07 | 1.13E-06 | 3.91E-07 | 3.91E-07 | rs35229468 | 5.95E-13 |  |
| SCZ | *MARS2* | 65 | 1.00E+00 | 3.96E-04 | 2.36E-04 | 4.19E-04 | 6.16E-04 | 2.79E-04 | 2.79E-04 | rs55751750 | 8.28E-10 |  |
| SCZ | *MAU2* | 58 | 1.00E+00 | 1.92E-03 | 1.22E-03 | 5.42E-03 | 6.73E-03 | 2.46E-03 | 2.46E-03 | rs2916068 | 1.08E-08 |  |
| SCZ | *MDK* | 36 | 1.00E+00 | 6.52E-05 | 4.10E-05 | 5.74E-05 | 8.19E-05 | 2.97E-05 | 2.97E-05 | rs7951870 | 1.67E-10 |  |
| SCZ | *ME1* | 123 | 1.00E+00 | 3.49E-02 | 3.07E-02 | 3.81E-01 | 5.73E-01 | 2.65E-01 | 2.65E-01 | rs577806 | 7.90E-08 | √ |
| SCZ | *MGAT3* | 44 | 1.00E+00 | 1.00E+00 | 1.00E+00 | 1.62E-02 | 9.72E-03 | 1.62E-02 | 1.62E-02 | rs5757717 | 7.76E-09 |  |
| SCZ | *MKL1* | 116 | 1.00E+00 | 1.14E-01 | 9.17E-02 | 3.60E-03 | 2.18E-03 | 3.59E-03 | 3.59E-03 | rs133047 | 1.85E-09 |  |
| SCZ | *MMP16* | 157 | 1.00E+00 | 5.79E-02 | 3.94E-02 | 1.84E-01 | 3.71E-01 | 1.19E-01 | 1.19E-01 | rs4246111 | 1.52E-07 | √ |
| SCZ | *MNT* | 85 | 1.00E+00 | 7.20E-01 | 2.32E-01 | 1.04E-02 | 1.34E-02 | 5.79E-03 | 5.79E-03 | rs4523957 | 1.19E-08 |  |
| SCZ | *MOB4* | 59 | 1.00E+00 | 9.29E-06 | 3.51E-06 | 3.02E-05 | 5.68E-05 | 1.44E-05 | 1.44E-05 | rs6434928 | 6.93E-11 |  |
| SCZ | *MPHOSPH9* | 74 | 1.00E+00 | 9.13E-06 | 8.27E-06 | 2.25E-06 | 2.96E-06 | 1.30E-06 | 1.30E-06 | rs1615350 | 2.56E-12 |  |
| SCZ | *MRPL33* | 27 | 1.00E+00 | 8.07E-03 | 7.80E-03 | 2.17E-02 | 2.53E-02 | 1.15E-02 | 1.15E-02 | rs12474906 | 7.70E-09 |  |
| SCZ | *MRPS21* | 58 | 1.00E+00 | 6.63E-01 | 5.03E-01 | 2.56E-02 | 2.96E-02 | 1.19E-02 | 1.19E-02 | rs143299593 | 3.20E-08 |  |
| SCZ | *MSANTD2* | 94 | 1.00E+00 | 7.64E-05 | 1.47E-05 | 3.07E-02 | 2.52E-02 | 1.25E-02 | 1.25E-02 | rs55661361 | 2.11E-11 |  |
| SCZ | *MSL2* | 58 | 1.00E+00 | 3.82E-03 | 2.23E-03 | 1.22E-03 | 1.42E-03 | 7.73E-04 | 7.73E-04 | rs1279424 | 1.67E-09 |  |
| SCZ | *MSRA* | 333 | 1.00E+00 | 2.52E-03 | 1.30E-03 | 2.28E-02 | 2.60E-02 | 1.76E-02 | 1.76E-02 | rs73191547 | 4.84E-09 |  |
| SCZ | *MTMR11* | 37 | 1.00E+00 | 1.00E+00 | 1.00E+00 | 1.64E-03 | 1.34E-03 | 1.05E-03 | 1.05E-03 | rs28366567 | 1.19E-09 |  |
| SCZ | *MTMR4* | 63 | 4.18E-02 | 8.30E-02 | 1.00E+00 | 1.00E+00 | 1.00E+00 | 1.00E+00 | 1.00E+00 | rs149327800 | 5.30E-06 | √ |
| SCZ | *MUSTN1* | 73 | 1.00E+00 | 3.40E-05 | 2.60E-05 | 1.27E-04 | 2.12E-04 | 7.48E-05 | 7.48E-05 | rs4481150 | 2.31E-10 |  |
| SCZ | *MVP* | 41 | 1.00E+00 | 9.06E-02 | 5.65E-02 | 6.70E-04 | 9.31E-04 | 3.73E-04 | 3.73E-04 | rs4788190 | 1.18E-09 |  |
| SCZ | *NAB2* | 65 | 1.00E+00 | 1.00E+00 | 1.00E+00 | 3.62E-02 | 1.71E-02 | 2.90E-02 | 2.90E-02 | rs12814239 | 3.31E-08 |  |
| SCZ | *NAGA* | 51 | 1.00E+00 | 1.18E-03 | 9.04E-04 | 2.27E-02 | 3.19E-02 | 1.13E-02 | 1.13E-02 | rs2854743 | 1.27E-08 |  |
| SCZ | *NCAM1* | 195 | 1.00E+00 | 7.54E-02 | 2.46E-02 | 3.37E-01 | 2.18E-01 | 3.28E-01 | 3.28E-01 | rs4987094 | 1.03E-07 | √ |
| SCZ | *NCAN* | 87 | 1.00E+00 | 3.56E-02 | 2.01E-02 | 2.51E-02 | 4.10E-02 | 1.50E-02 | 1.50E-02 | rs67720221 | 4.19E-08 |  |
| SCZ | *NCK1* | 98 | 1.00E+00 | 4.61E-01 | 4.08E-01 | 1.66E-02 | 6.93E-03 | 1.65E-02 | 1.65E-02 | rs1280624 | 9.03E-09 |  |
| SCZ | *NDUFA13* | 54 | 1.00E+00 | 3.72E-04 | 2.10E-04 | 2.17E-03 | 6.11E-03 | 1.37E-03 | 1.37E-03 | rs7245983 | 1.03E-08 |  |
| SCZ | *NDUFA4L2* | 50 | 1.00E+00 | 1.00E+00 | 1.00E+00 | 2.78E-02 | 1.62E-02 | 2.78E-02 | 2.78E-02 | rs61937595 | 2.39E-12 |  |
| SCZ | *NDUFA6* | 53 | 1.00E+00 | 2.41E-03 | 1.84E-03 | 2.36E-02 | 3.22E-02 | 1.18E-02 | 1.18E-02 | rs760648 | 5.02E-09 |  |
| SCZ | *NDUFAF2* | 42 | 1.00E+00 | 1.00E+00 | 1.00E+00 | 8.72E-05 | 7.15E-05 | 6.17E-05 | 6.17E-05 | rs177114 | 2.16E-11 |  |
| SCZ | *NEK1* | 119 | 1.00E+00 | 1.27E-01 | 7.78E-02 | 7.09E-02 | 1.35E-01 | 3.86E-02 | 3.86E-02 | rs7696555 | 1.54E-07 | √ |
| SCZ | *NEK4* | 103 | 1.00E+00 | 7.71E-04 | 7.13E-04 | 1.79E-04 | 2.02E-04 | 1.11E-04 | 1.11E-04 | rs4481150 | 2.31E-10 |  |
| SCZ | *NEU2* | 73 | 1.00E+00 | 1.00E+00 | 1.00E+00 | 3.58E-04 | 5.10E-04 | 3.10E-04 | 3.10E-04 | rs778350 | 3.58E-10 |  |
| SCZ | *NFAM1* | 97 | 1.00E+00 | 1.00E+00 | 1.00E+00 | 5.74E-02 | 4.39E-02 | 5.08E-02 | 5.08E-02 | rs134906 | 2.52E-08 |  |
| SCZ | *NFATC3* | 87 | 1.00E+00 | 4.77E-01 | 4.31E-01 | 4.20E-02 | 3.87E-02 | 2.51E-02 | 2.51E-02 | rs10852439 | 7.45E-08 | √ |
| SCZ | *NGEF* | 128 | 1.00E+00 | 1.73E-05 | 9.97E-06 | 1.33E-05 | 2.28E-05 | 5.87E-06 | 5.87E-06 | rs62193339 | 6.55E-12 |  |
| SCZ | *NISCH* | 76 | 1.00E+00 | 2.70E-02 | 1.11E-02 | 7.35E-03 | 3.98E-03 | 6.41E-03 | 6.41E-03 | rs2336147 | 5.42E-09 |  |
| SCZ | *NMB* | 67 | 1.00E+00 | 1.16E-04 | 7.29E-05 | 2.52E-04 | 2.07E-04 | 1.16E-04 | 1.16E-04 | rs17531523 | 8.97E-11 |  |
| SCZ | *NOS1* | 168 | 1.00E+00 | 6.54E-02 | 3.28E-02 | 1.00E+00 | 1.00E+00 | 1.00E+00 | 1.00E+00 | rs28607014 | 9.03E-07 | √ |
| SCZ | *NOSIP* | 53 | 1.00E+00 | 3.46E-03 | 2.38E-03 | 5.82E-01 | 3.69E-01 | 2.43E-01 | 2.43E-01 | rs6509439 | 6.52E-07 | √ |
| SCZ | *NPC1L1* | 52 | 1.00E+00 | 5.05E-02 | 3.42E-02 | 1.00E+00 | 1.00E+00 | 7.85E-01 | 7.85E-01 | rs217384 | 8.40E-06 | √ |
| SCZ | *NPTX1* | 89 | 1.00E+00 | 6.15E-02 | 2.18E-02 | 1.00E+00 | 1.00E+00 | 1.00E+00 | 1.00E+00 | rs11658698 | 6.27E-06 | √ |
| SCZ | *NRGN* | 60 | 1.00E+00 | 1.17E-05 | 3.23E-06 | 1.96E-02 | 1.62E-02 | 7.99E-03 | 7.99E-03 | rs55661361 | 2.11E-11 |  |
| SCZ | *NSUN6* | 137 | 1.00E+00 | 4.14E-03 | 1.18E-03 | 1.82E-04 | 1.65E-04 | 1.58E-04 | 1.58E-04 | rs7893279 | 7.88E-11 |  |
| SCZ | *NT5C2* | 92 | 1.00E+00 | 1.06E-08 | 5.61E-09 | 1.60E-10 | 6.87E-10 | 8.16E-11 | 8.22E-11 | rs11191582 | 7.33E-17 |  |
| SCZ | *NT5DC2* | 72 | 1.00E+00 | 6.87E-03 | 6.26E-03 | 4.17E-04 | 2.71E-04 | 3.56E-04 | 3.56E-04 | rs2577831 | 3.44E-10 |  |
| SCZ | *NUDT1* | 71 | 1.00E+00 | 2.95E-02 | 9.43E-03 | 2.05E-02 | 2.26E-02 | 1.32E-02 | 1.32E-02 | rs3779003 | 2.68E-08 |  |
| SCZ | *NXPH4* | 55 | 1.00E+00 | 1.00E+00 | 1.00E+00 | 3.06E-02 | 1.87E-02 | 3.06E-02 | 3.05E-02 | rs61937595 | 2.39E-12 |  |
| SCZ | *OGFOD2* | 30 | 1.00E+00 | 3.50E-05 | 2.69E-05 | 3.49E-04 | 5.01E-04 | 1.65E-04 | 1.65E-04 | rs11608811 | 1.23E-09 |  |
| SCZ | *OPRD1* | 72 | 1.00E+00 | 1.00E+00 | 1.00E+00 | 4.79E-02 | 2.98E-02 | 4.64E-02 | 4.64E-02 | rs533123 | 3.96E-08 |  |
| SCZ | *ORMDL3* | 69 | 3.58E-03 | 7.93E-03 | 1.00E+00 | 1.00E+00 | 1.00E+00 | 1.00E+00 | 1.00E+00 | rs72832972 | 0.000863 | √ |
| SCZ | *OTUD7B* | 51 | 1.00E+00 | 5.15E-03 | 3.42E-03 | 1.05E-03 | 1.61E-03 | 4.95E-04 | 4.95E-04 | rs2319280 | 9.64E-10 |  |
| SCZ | *PACSIN3* | 27 | 1.14E-02 | 1.62E-02 | 1.00E+00 | 1.00E+00 | 8.95E-01 | 8.10E-01 | 8.10E-01 | rs1352307 | 2.81E-06 | √ |
| SCZ | *PBRM1* | 120 | 1.00E+00 | 8.79E-03 | 8.44E-03 | 3.47E-04 | 2.47E-04 | 2.85E-04 | 2.85E-04 | rs2710323 | 2.93E-10 |  |
| SCZ | *PBX4* | 82 | 1.00E+00 | 8.04E-04 | 4.26E-04 | 4.62E-03 | 1.15E-02 | 2.74E-03 | 2.74E-03 | rs7245983 | 1.03E-08 |  |
| SCZ | *PCCB* | 76 | 1.00E+00 | 1.25E-04 | 6.11E-05 | 8.31E-04 | 1.03E-03 | 4.01E-04 | 4.01E-04 | rs696520 | 1.06E-09 |  |
| SCZ | *PCDHA1* | 172 | 1.00E+00 | 1.55E-02 | 1.43E-02 | 2.42E-01 | 7.04E-01 | 1.25E-01 | 1.25E-01 | rs62384243 | 7.96E-07 | √ |
| SCZ | *PCDHA10* | 150 | 1.00E+00 | 1.99E-02 | 1.76E-02 | 2.21E-01 | 6.33E-01 | 1.23E-01 | 1.23E-01 | rs2563265 | 9.69E-07 | √ |
| SCZ | *PCDHA11* | 148 | 1.00E+00 | 2.16E-02 | 1.88E-02 | 2.32E-01 | 1.00E+00 | 1.34E-01 | 1.34E-01 | rs7733403 | 1.34E-06 | √ |
| SCZ | *PCDHA12* | 146 | 1.00E+00 | 2.35E-02 | 2.00E-02 | 2.45E-01 | 1.00E+00 | 1.41E-01 | 1.41E-01 | rs2014738 | 1.40E-06 | √ |
| SCZ | *PCDHA13* | 143 | 1.00E+00 | 2.64E-02 | 2.17E-02 | 2.67E-01 | 1.00E+00 | 1.49E-01 | 1.49E-01 | rs13157397 | 1.94E-06 | √ |
| SCZ | *PCDHA2* | 166 | 1.00E+00 | 1.53E-02 | 1.42E-02 | 2.33E-01 | 6.79E-01 | 1.22E-01 | 1.22E-01 | rs62384243 | 7.96E-07 | √ |
| SCZ | *PCDHA3* | 164 | 1.00E+00 | 1.57E-02 | 1.45E-02 | 2.30E-01 | 6.79E-01 | 1.22E-01 | 1.22E-01 | rs62384243 | 7.96E-07 | √ |
| SCZ | *PCDHA4* | 161 | 1.00E+00 | 1.66E-02 | 1.52E-02 | 2.26E-01 | 6.71E-01 | 1.21E-01 | 1.21E-01 | rs62384243 | 7.96E-07 | √ |
| SCZ | *PCDHA5* | 159 | 1.00E+00 | 1.70E-02 | 1.54E-02 | 2.23E-01 | 6.68E-01 | 1.20E-01 | 1.20E-01 | rs2563252 | 9.17E-07 | √ |
| SCZ | *PCDHA6* | 157 | 1.00E+00 | 1.82E-02 | 1.64E-02 | 2.27E-01 | 6.58E-01 | 1.24E-01 | 1.24E-01 | rs2563265 | 9.69E-07 | √ |
| SCZ | *PCDHA7* | 156 | 1.00E+00 | 1.80E-02 | 1.62E-02 | 2.26E-01 | 6.51E-01 | 1.23E-01 | 1.23E-01 | rs2563265 | 9.69E-07 | √ |
| SCZ | *PCDHA8* | 154 | 1.00E+00 | 1.87E-02 | 1.68E-02 | 2.27E-01 | 6.47E-01 | 1.24E-01 | 1.24E-01 | rs2563265 | 9.69E-07 | √ |
| SCZ | *PCDHA9* | 151 | 1.00E+00 | 1.95E-02 | 1.73E-02 | 2.22E-01 | 6.40E-01 | 1.24E-01 | 1.24E-01 | rs2563265 | 9.69E-07 | √ |
| SCZ | *PCNX* | 158 | 1.00E+00 | 9.21E-04 | 6.06E-04 | 4.03E-03 | 8.33E-03 | 2.41E-03 | 2.41E-03 | rs2810117 | 2.44E-09 |  |
| SCZ | *PIM3* | 54 | 1.00E+00 | 1.16E-02 | 9.15E-03 | 1.48E-01 | 2.17E-01 | 8.60E-02 | 1.00E+00 | rs5770755 | 4.91E-07 | √ |
| SCZ | *PITPNM2* | 66 | 1.00E+00 | 1.15E-05 | 1.05E-05 | 2.01E-06 | 2.66E-06 | 1.40E-06 | 1.40E-06 | rs1615350 | 2.56E-12 |  |
| SCZ | *PLA2G15* | 71 | 1.00E+00 | 1.32E-01 | 9.25E-02 | 3.43E-02 | 4.89E-02 | 2.07E-02 | 2.07E-02 | rs10852439 | 7.45E-08 | √ |
| SCZ | *PLCL1* | 163 | 1.00E+00 | 6.40E-04 | 4.09E-04 | 7.50E-03 | 8.64E-03 | 3.03E-03 | 3.03E-03 | rs35657897 | 5.87E-09 |  |
| SCZ | *PLEKHO1* | 45 | 1.00E+00 | 1.64E-04 | 1.38E-04 | 6.72E-04 | 1.68E-03 | 3.46E-04 | 3.46E-04 | rs1824850 | 1.20E-09 |  |
| SCZ | *PPDPF* | 69 | 1.00E+00 | 3.21E-02 | 1.34E-02 | 1.00E+00 | 1.00E+00 | 6.47E-01 | 6.47E-01 | rs200050069 | 9.17E-08 | √ |
| SCZ | *PPM1M* | 69 | 1.00E+00 | 1.00E+00 | 1.00E+00 | 3.79E-02 | 3.55E-02 | 2.04E-02 | 2.04E-02 | rs7622851 | 4.21E-08 |  |
| SCZ | *PPP1R13B* | 107 | 1.00E+00 | 8.31E-07 | 7.59E-07 | 3.23E-06 | 1.74E-05 | 2.26E-06 | 2.26E-06 | rs6576008 | 6.46E-12 |  |
| SCZ | *PPP1R16B* | 120 | 1.00E+00 | 2.72E-04 | 1.01E-04 | 1.92E-04 | 1.65E-04 | 1.80E-04 | 1.80E-04 | rs6065094 | 9.53E-11 |  |
| SCZ | *PPP2R3A* | 93 | 1.00E+00 | 5.65E-02 | 4.45E-02 | 2.44E-03 | 4.51E-03 | 2.07E-03 | 2.07E-03 | rs7372313 | 1.97E-09 |  |
| SCZ | *PPP4C* | 65 | 1.00E+00 | 1.42E-03 | 1.31E-03 | 3.56E-04 | 1.30E-03 | 2.75E-04 | 2.75E-04 | rs3814883 | 9.30E-11 |  |
| SCZ | *PRC1* | 75 | 1.00E+00 | 1.13E-01 | 3.81E-02 | 7.23E-06 | 7.14E-06 | 7.14E-06 | 7.14E-06 | rs4702 | 5.73E-12 |  |
| SCZ | *PRICKLE2* | 175 | 1.00E+00 | 1.21E-02 | 3.35E-03 | 5.25E-02 | 9.84E-02 | 3.45E-02 | 3.45E-02 | rs9879045 | 3.06E-08 |  |
| SCZ | *PRKCD* | 101 | 1.00E+00 | 7.59E-02 | 3.75E-02 | 1.07E-03 | 8.99E-04 | 7.57E-04 | 7.57E-04 | rs6799185 | 6.31E-10 |  |
| SCZ | *PRKD1* | 165 | 1.00E+00 | 2.74E-03 | 1.95E-03 | 1.88E-01 | 1.55E-01 | 1.00E-01 | 1.00E-01 | rs10149407 | 2.06E-08 |  |
| SCZ | *PRMT1* | 52 | 1.00E+00 | 7.79E-03 | 6.36E-03 | 5.71E-01 | 4.26E-01 | 2.44E-01 | 2.44E-01 | rs6509439 | 6.52E-07 | √ |
| SCZ | *PRMT7* | 98 | 1.00E+00 | 9.96E-01 | 7.78E-01 | 6.32E-02 | 6.92E-02 | 4.01E-02 | 4.01E-02 | rs10852439 | 7.45E-08 | √ |
| SCZ | *PRPF3* | 57 | 1.00E+00 | 4.79E-01 | 3.35E-01 | 2.51E-02 | 2.97E-02 | 1.51E-02 | 1.51E-02 | rs72696880 | 2.67E-08 |  |
| SCZ | *PRR12* | 55 | 1.00E+00 | 2.33E-03 | 1.91E-03 | 6.04E-01 | 4.15E-01 | 2.53E-01 | 2.53E-01 | rs6509439 | 6.52E-07 | √ |
| SCZ | *PRRG2* | 48 | 1.00E+00 | 2.77E-03 | 2.02E-03 | 5.27E-01 | 3.35E-01 | 2.20E-01 | 2.20E-01 | rs6509439 | 6.52E-07 | √ |
| SCZ | *PRRT2* | 32 | 1.00E+00 | 1.00E+00 | 1.00E+00 | 1.70E-02 | 9.49E-03 | 1.70E-02 | 1.70E-02 | rs8059619 | 4.59E-09 |  |
| SCZ | *PRSS35* | 71 | 1.00E+00 | 1.23E-03 | 9.71E-04 | 2.94E-03 | 4.37E-03 | 1.88E-03 | 1.88E-03 | rs3798869 | 2.33E-09 |  |
| SCZ | *PSD3* | 429 | 1.00E+00 | 2.83E-02 | 6.79E-03 | 1.00E+00 | 1.00E+00 | 1.00E+00 | 1.00E+00 | rs876983 | 7.22E-08 | √ |
| SCZ | *PSMA4* | 60 | 1.00E+00 | 1.08E-02 | 8.33E-03 | 1.13E-05 | 1.18E-05 | 5.12E-06 | 5.12E-06 | rs28681284 | 2.12E-13 |  |
| SCZ | *PSMD6* | 87 | 1.00E+00 | 1.88E-03 | 7.51E-04 | 6.74E-03 | 8.40E-03 | 3.45E-03 | 3.45E-03 | rs1828944 | 4.53E-09 |  |
| SCZ | *PTGIS* | 134 | 1.00E+00 | 8.23E-03 | 2.50E-03 | 1.62E-01 | 1.51E-01 | 7.55E-02 | 7.55E-02 | rs7267348 | 8.34E-08 | √ |
| SCZ | *PTK6* | 68 | 1.00E+00 | 3.43E-02 | 1.44E-02 | 1.00E+00 | 1.00E+00 | 6.38E-01 | 6.38E-01 | rs200050069 | 9.17E-08 | √ |
| SCZ | *PTN* | 136 | 1.00E+00 | 3.03E-02 | 7.46E-03 | 8.35E-03 | 3.28E-02 | 5.38E-03 | 5.38E-03 | rs10954580 | 1.78E-08 |  |
| SCZ | *PTPRF* | 113 | 1.00E+00 | 1.24E-01 | 5.31E-02 | 8.75E-03 | 5.30E-03 | 6.29E-03 | 6.29E-03 | rs11210892 | 4.60E-09 |  |
| SCZ | *PUS7* | 70 | 1.00E+00 | 6.74E-01 | 5.04E-01 | 1.39E-02 | 2.20E-02 | 1.20E-02 | 1.20E-02 | rs12705306 | 1.36E-08 |  |
| SCZ | *R3HDM2* | 51 | 1.00E+00 | 1.00E+00 | 1.00E+00 | 2.84E-02 | 1.36E-02 | 2.83E-02 | 2.83E-02 | rs61937595 | 2.39E-12 |  |
| SCZ | *RAD9B* | 31 | 1.00E+00 | 5.52E-02 | 3.63E-02 | 1.20E-01 | 1.83E-01 | 8.29E-02 | 8.28E-02 | rs28764794 | 4.76E-07 | √ |
| SCZ | *RANGAP1* | 62 | 1.00E+00 | 1.49E-01 | 6.40E-02 | 2.31E-03 | 3.87E-03 | 1.61E-03 | 1.61E-03 | rs9607782 | 2.86E-12 |  |
| SCZ | *RBBP5* | 94 | 1.00E+00 | 7.82E-03 | 5.44E-03 | 1.00E+00 | 1.00E+00 | 1.00E+00 | 1.00E+00 | rs11240341 | 1.02E-06 | √ |
| SCZ | *RBFOX1* | 1498 | 1.00E+00 | 1.88E-02 | 2.37E-03 | 1.00E+00 | 1.00E+00 | 1.00E+00 | 1.00E+00 | rs8056990 | 6.37E-06 | √ |
| SCZ | *RBKS* | 46 | 1.00E+00 | 1.04E-02 | 1.01E-02 | 3.70E-02 | 3.08E-02 | 1.79E-02 | 1.79E-02 | rs12474906 | 7.70E-09 |  |
| SCZ | *RBMS3* | 457 | 1.00E+00 | 1.63E-03 | 4.45E-04 | 1.00E+00 | 1.00E+00 | 9.75E-01 | 1.00E+00 | rs138565042 | 1.17E-07 | √ |
| SCZ | *RBX1* | 22 | 1.00E+00 | 2.84E-02 | 1.30E-02 | 1.13E-03 | 1.39E-03 | 7.98E-04 | 7.98E-04 | rs4821998 | 3.60E-09 |  |
| SCZ | *RCCD1* | 70 | 1.00E+00 | 2.04E-02 | 5.65E-03 | 6.75E-06 | 6.66E-06 | 6.66E-06 | 6.66E-06 | rs4702 | 5.73E-12 |  |
| SCZ | *RCN3* | 60 | 1.00E+00 | 3.48E-02 | 2.71E-02 | 6.58E-01 | 3.85E-01 | 3.00E-01 | 3.00E-01 | rs6509439 | 6.52E-07 | √ |
| SCZ | *REEP2* | 61 | 1.00E+00 | 4.27E-03 | 1.77E-03 | 1.03E-02 | 8.14E-03 | 5.83E-03 | 5.83E-03 | rs3849046 | 1.22E-08 |  |
| SCZ | *RERE* | 144 | 1.00E+00 | 2.98E-01 | 2.39E-01 | 9.95E-03 | 2.30E-02 | 7.01E-03 | 7.01E-03 | rs301798 | 1.84E-08 |  |
| SCZ | *RFT1* | 84 | 1.00E+00 | 5.83E-02 | 3.24E-02 | 8.92E-04 | 7.38E-04 | 6.28E-04 | 6.28E-04 | rs6799185 | 6.31E-10 |  |
| SCZ | *RFTN2* | 91 | 1.00E+00 | 4.30E-05 | 2.30E-05 | 5.84E-05 | 1.72E-04 | 3.58E-05 | 3.58E-05 | rs7605813 | 1.17E-10 |  |
| SCZ | *RGS6* | 495 | 1.00E+00 | 1.00E+00 | 1.00E+00 | 3.51E-02 | 2.53E-02 | 2.88E-02 | 1.00E+00 | rs2332700 | 4.38E-10 |  |
| SCZ | *RILPL2* | 52 | 1.00E+00 | 2.33E-02 | 1.46E-02 | 4.68E-02 | 2.85E-02 | 3.13E-02 | 3.13E-02 | rs28768122 | 1.97E-09 |  |
| SCZ | *RPRD2* | 84 | 1.00E+00 | 1.00E+00 | 6.92E-01 | 3.70E-02 | 3.70E-02 | 2.08E-02 | 2.08E-02 | rs141935877 | 1.69E-08 |  |
| SCZ | *RPS19BP1* | 42 | 1.00E+00 | 1.00E+00 | 1.00E+00 | 1.54E-02 | 1.06E-02 | 1.54E-02 | 1.54E-02 | rs5757717 | 7.76E-09 |  |
| SCZ | *RRAS* | 45 | 1.00E+00 | 4.46E-03 | 3.75E-03 | 4.94E-01 | 3.39E-01 | 2.11E-01 | 2.11E-01 | rs6509439 | 6.52E-07 | √ |
| SCZ | *SATB2* | 77 | 1.00E+00 | 7.04E-03 | 3.91E-03 | 2.08E-01 | 2.67E-01 | 1.00E-01 | 1.00E-01 | rs6704641 | 4.45E-07 | √ |
| SCZ | *SBNO1* | 64 | 1.00E+00 | 7.21E-05 | 5.88E-05 | 5.19E-06 | 4.84E-06 | 2.90E-06 | 2.90E-06 | rs2102949 | 4.56E-12 |  |
| SCZ | *SCAF1* | 47 | 1.00E+00 | 5.74E-03 | 4.98E-03 | 5.16E-01 | 3.69E-01 | 2.21E-01 | 2.21E-01 | rs6509439 | 6.52E-07 | √ |
| SCZ | *SCGN* | 125 | 1.00E+00 | 1.80E-01 | 7.99E-02 | 1.91E-11 | 4.67E-10 | 1.66E-11 | 1.68E-11 | rs34493019 | 1.42E-19 |  |
| SCZ | *SDCCAG8* | 133 | 1.00E+00 | 2.79E-06 | 5.41E-07 | 9.14E-02 | 2.15E-01 | 4.17E-02 | 4.17E-02 | rs12093576 | 1.51E-08 |  |
| SCZ | *SEC11A* | 86 | 1.00E+00 | 4.82E-04 | 1.82E-04 | 3.23E-04 | 2.56E-04 | 1.48E-04 | 1.48E-04 | rs17531523 | 8.97E-11 |  |
| SCZ | *SETD8* | 51 | 1.00E+00 | 7.18E-03 | 3.62E-03 | 2.29E-02 | 2.34E-02 | 1.76E-02 | 1.76E-02 | rs28768122 | 1.97E-09 |  |
| SCZ | *SEZ6L2* | 56 | 1.00E+00 | 1.54E-04 | 1.35E-04 | 1.97E-04 | 6.25E-04 | 1.02E-04 | 1.02E-04 | rs3814883 | 9.30E-11 |  |
| SCZ | *SF3B1* | 53 | 1.00E+00 | 1.12E-05 | 6.18E-06 | 1.39E-05 | 3.08E-05 | 8.79E-06 | 8.79E-06 | rs4685 | 4.12E-11 |  |
| SCZ | *SFMBT1* | 111 | 1.00E+00 | 1.47E-04 | 8.02E-05 | 2.95E-04 | 3.30E-04 | 1.45E-04 | 1.45E-04 | rs4481150 | 2.31E-10 |  |
| SCZ | *SFXN2* | 86 | 1.00E+00 | 3.65E-05 | 1.33E-05 | 2.33E-06 | 2.30E-06 | 1.24E-06 | 1.24E-06 | rs17115100 | 1.61E-12 |  |
| SCZ | *SGSM2* | 93 | 1.00E+00 | 4.66E-02 | 1.53E-02 | 6.66E-03 | 1.28E-02 | 3.83E-03 | 3.83E-03 | rs4523957 | 1.19E-08 |  |
| SCZ | *SH3RF1* | 122 | 1.00E+00 | 2.56E-01 | 1.59E-01 | 3.60E-02 | 4.54E-02 | 3.10E-02 | 3.10E-02 | rs7683893 | 3.01E-08 |  |
| SCZ | *SHISA6* | 288 | 1.00E+00 | 5.17E-02 | 1.08E-02 | 1.00E+00 | 1.00E+00 | 1.00E+00 | 1.00E+00 | rs1546560 | 2.19E-06 | √ |
| SCZ | *SHISA8* | 64 | 1.00E+00 | 3.80E-04 | 1.62E-04 | 2.00E-02 | 3.60E-02 | 1.40E-02 | 1.40E-02 | rs77441740 | 2.65E-08 |  |
| SCZ | *SHMT2* | 52 | 1.00E+00 | 1.00E+00 | 1.00E+00 | 2.89E-02 | 1.75E-02 | 2.89E-02 | 2.89E-02 | rs61937595 | 2.39E-12 |  |
| SCZ | *SIAE* | 81 | 1.00E+00 | 2.20E-07 | 2.74E-08 | 2.65E-02 | 2.24E-02 | 1.12E-02 | 1.12E-02 | rs55661361 | 2.11E-11 |  |
| SCZ | *SLC17A1* | 106 | 1.00E+00 | 2.47E-10 | 3.60E-11 | 4.53E-14 | 3.96E-10 | 4.51E-14 | 4.51E-14 | rs34043431 | 1.91E-20 |  |
| SCZ | *SLC17A2* | 92 | 1.00E+00 | 1.27E-06 | 6.53E-07 | 1.97E-14 | 3.44E-10 | 1.77E-14 | 1.77E-14 | rs13191445 | 9.41E-21 |  |
| SCZ | *SLC17A3* | 105 | 1.00E+00 | 2.51E-08 | 6.49E-09 | 4.49E-14 | 3.92E-10 | 4.46E-14 | 4.46E-14 | rs34043431 | 1.91E-20 |  |
| SCZ | *SLC17A4* | 91 | 9.72E-01 | 1.52E-09 | 2.98E-10 | 3.89E-14 | 3.40E-10 | 3.87E-14 | 3.87E-14 | rs34043431 | 1.91E-20 |  |
| SCZ | *SLC32A1* | 100 | 1.00E+00 | 8.35E-06 | 2.49E-06 | 1.60E-04 | 1.45E-04 | 1.53E-04 | 1.53E-04 | rs6065094 | 9.53E-11 |  |
| SCZ | *SLC35G2* | 62 | 1.00E+00 | 1.00E+00 | 1.00E+00 | 2.77E-03 | 1.92E-03 | 1.98E-03 | 1.98E-03 | rs9830653 | 4.03E-09 |  |
| SCZ | *SLC39A8* | 108 | 1.00E+00 | 1.00E+00 | 1.00E+00 | 7.07E-07 | 7.04E-07 | 7.07E-07 | 7.07E-07 | rs13107325 | 3.89E-13 |  |
| SCZ | *SLC45A1* | 60 | 1.00E+00 | 8.15E-04 | 1.35E-04 | 4.15E-03 | 1.31E-02 | 2.92E-03 | 2.92E-03 | rs301798 | 1.84E-08 |  |
| SCZ | *SLC7A6* | 87 | 1.00E+00 | 2.61E-01 | 1.93E-01 | 5.61E-02 | 6.04E-02 | 3.53E-02 | 3.53E-02 | rs10852439 | 7.45E-08 | √ |
| SCZ | *SLC7A6OS* | 84 | 1.00E+00 | 3.56E-01 | 2.73E-01 | 5.41E-02 | 6.19E-02 | 3.43E-02 | 3.43E-02 | rs10852439 | 7.45E-08 | √ |
| SCZ | *SMG6* | 176 | 1.00E+00 | 2.46E-03 | 2.08E-03 | 1.16E-02 | 2.14E-02 | 6.33E-03 | 6.33E-03 | rs4523957 | 1.19E-08 |  |
| SCZ | *SNAP91* | 114 | 1.00E+00 | 3.86E-05 | 3.07E-05 | 7.48E-04 | 2.03E-03 | 3.76E-04 | 3.76E-04 | rs217289 | 1.58E-09 |  |
| SCZ | *SNX19* | 136 | 1.00E+00 | 1.29E-06 | 4.50E-07 | 2.25E-06 | 3.88E-06 | 1.74E-06 | 1.74E-06 | rs10791097 | 1.73E-12 |  |
| SCZ | *SNX8* | 98 | 1.00E+00 | 5.28E-03 | 1.55E-03 | 2.96E-01 | 2.64E-01 | 2.08E-01 | 2.08E-01 | rs2058430 | 5.80E-08 | √ |
| SCZ | *SPA17* | 62 | 1.00E+00 | 1.67E-07 | 2.29E-08 | 2.03E-02 | 1.79E-02 | 8.24E-03 | 8.24E-03 | rs55661361 | 2.11E-11 |  |
| SCZ | *SPATA19* | 100 | 1.00E+00 | 5.18E-02 | 3.01E-02 | 8.89E-01 | 7.63E-01 | 5.62E-01 | 5.62E-01 | rs73034263 | 1.57E-09 |  |
| SCZ | *SPATS2L* | 194 | 1.00E+00 | 6.71E-04 | 2.30E-04 | 2.53E-06 | 4.58E-06 | 2.09E-06 | 2.09E-06 | rs11687313 | 1.42E-12 |  |
| SCZ | *SPCS1* | 80 | 1.00E+00 | 6.51E-03 | 6.33E-03 | 1.39E-04 | 1.37E-04 | 1.16E-04 | 1.16E-04 | rs4481150 | 2.31E-10 |  |
| SCZ | *SREBF2* | 85 | 1.00E+00 | 5.91E-03 | 2.74E-03 | 2.65E-02 | 4.17E-02 | 1.85E-02 | 1.85E-02 | rs77441740 | 2.65E-08 |  |
| SCZ | *SRMS* | 68 | 1.00E+00 | 3.15E-02 | 1.34E-02 | 1.00E+00 | 1.00E+00 | 6.38E-01 | 6.38E-01 | rs200050069 | 9.17E-08 | √ |
| SCZ | *SRPK2* | 63 | 1.00E+00 | 1.87E-04 | 1.76E-04 | 4.26E-04 | 1.53E-03 | 2.39E-04 | 2.39E-04 | rs7789569 | 2.88E-09 |  |
| SCZ | *SRR* | 94 | 1.00E+00 | 9.78E-03 | 7.12E-03 | 6.21E-03 | 1.21E-02 | 3.45E-03 | 3.45E-03 | rs4523957 | 1.19E-08 |  |
| SCZ | *ST3GAL3* | 151 | 1.00E+00 | 8.17E-01 | 6.13E-01 | 1.17E-02 | 6.88E-03 | 1.16E-02 | 1.16E-02 | rs11210892 | 4.60E-09 |  |
| SCZ | *STAB1* | 77 | 1.00E+00 | 6.15E-03 | 4.80E-03 | 4.46E-04 | 3.03E-04 | 3.81E-04 | 3.81E-04 | rs2577831 | 3.44E-10 |  |
| SCZ | *STAC3* | 46 | 1.00E+00 | 1.00E+00 | 1.00E+00 | 2.56E-02 | 1.39E-02 | 2.55E-02 | 2.55E-02 | rs61937595 | 2.39E-12 |  |
| SCZ | *STAG1* | 163 | 1.00E+00 | 6.86E-04 | 2.90E-04 | 1.02E-04 | 3.07E-04 | 7.08E-05 | 7.08E-05 | rs12163529 | 1.47E-10 |  |
| SCZ | *STAT6* | 69 | 1.00E+00 | 1.00E+00 | 1.00E+00 | 3.84E-02 | 1.90E-02 | 3.07E-02 | 3.07E-02 | rs12814239 | 3.31E-08 |  |
| SCZ | *SUFU* | 119 | 1.00E+00 | 1.00E+00 | 1.00E+00 | 3.14E-03 | 2.88E-03 | 1.26E-03 | 1.26E-03 | rs10883761 | 2.39E-09 |  |
| SCZ | *SUGP1* | 70 | 1.00E+00 | 4.66E-03 | 2.99E-03 | 9.00E-03 | 9.72E-03 | 3.92E-03 | 3.92E-03 | rs2916068 | 1.08E-08 |  |
| SCZ | *TAOK2* | 74 | 1.00E+00 | 1.69E-04 | 1.65E-04 | 2.14E-04 | 7.27E-04 | 1.18E-04 | 1.18E-04 | rs3814883 | 9.30E-11 |  |
| SCZ | *TBRG1* | 73 | 1.00E+00 | 3.37E-02 | 9.70E-03 | 1.33E-01 | 1.07E-01 | 7.02E-02 | 7.02E-02 | rs585454 | 1.08E-07 | √ |
| SCZ | *TBX6* | 59 | 1.00E+00 | 3.15E-03 | 2.95E-03 | 5.42E-04 | 1.68E-03 | 4.40E-04 | 4.40E-04 | rs3814881 | 2.76E-09 |  |
| SCZ | *TCF20* | 92 | 1.00E+00 | 6.08E-04 | 4.13E-04 | 1.09E-02 | 1.96E-02 | 5.56E-03 | 5.56E-03 | rs6002655 | 2.31E-10 |  |
| SCZ | *TCF4* | 193 | 1.00E+00 | 8.42E-02 | 5.23E-02 | 4.80E-07 | 4.78E-07 | 4.80E-07 | 4.80E-07 | rs9636107 | 1.48E-13 |  |
| SCZ | *TDRD9* | 82 | 1.00E+00 | 1.00E+00 | 1.00E+00 | 4.95E-06 | 1.38E-05 | 4.04E-06 | 4.04E-06 | rs6576008 | 6.46E-12 |  |
| SCZ | *THOC7* | 49 | 1.00E+00 | 5.72E-05 | 1.94E-05 | 5.98E-04 | 5.29E-04 | 3.79E-04 | 3.79E-04 | rs832187 | 7.26E-10 |  |
| SCZ | *TKT* | 88 | 6.88E-01 | 2.01E-01 | 4.94E-01 | 2.62E-02 | 2.51E-02 | 2.27E-02 | 2.27E-02 | rs11713763 | 2.73E-08 |  |
| SCZ | *TLR9* | 67 | 1.00E+00 | 1.00E+00 | 8.54E-01 | 3.68E-02 | 3.79E-02 | 1.98E-02 | 1.98E-02 | rs7622851 | 4.21E-08 |  |
| SCZ | *TM6SF2* | 67 | 1.00E+00 | 1.05E-02 | 6.16E-03 | 1.38E-02 | 2.74E-02 | 8.37E-03 | 8.37E-03 | rs2905430 | 3.52E-08 |  |
| SCZ | *TMEM110* | 88 | 1.00E+00 | 2.61E-05 | 1.95E-05 | 1.53E-04 | 2.54E-04 | 8.54E-05 | 8.54E-05 | rs4481150 | 2.31E-10 |  |
| SCZ | *TMEM110-MUSTN1* | 89 | 1.00E+00 | 2.81E-05 | 2.11E-05 | 1.55E-04 | 2.55E-04 | 8.63E-05 | 8.63E-05 | rs4481150 | 2.31E-10 |  |
| SCZ | *TMEM180* | 73 | 1.00E+00 | 1.00E+00 | 1.00E+00 | 1.20E-02 | 1.09E-02 | 1.07E-02 | 1.07E-02 | rs10883735 | 1.59E-08 |  |
| SCZ | *TMEM194A* | 68 | 1.00E+00 | 1.00E+00 | 1.00E+00 | 3.78E-02 | 1.80E-02 | 3.03E-02 | 3.03E-02 | rs12814239 | 3.31E-08 |  |
| SCZ | *TMEM219* | 72 | 1.00E+00 | 2.09E-04 | 2.04E-04 | 2.09E-04 | 7.20E-04 | 1.15E-04 | 1.15E-04 | rs3814883 | 9.30E-11 |  |
| SCZ | *TMEM81* | 85 | 1.00E+00 | 4.91E-03 | 2.47E-03 | 1.00E+00 | 1.00E+00 | 1.00E+00 | 1.00E+00 | rs11240341 | 1.02E-06 | √ |
| SCZ | *TNFRSF13C* | 63 | 1.00E+00 | 2.29E-04 | 9.15E-05 | 1.97E-02 | 3.42E-02 | 1.37E-02 | 1.37E-02 | rs77441740 | 2.65E-08 |  |
| SCZ | *TOM1L2* | 24 | 1.00E+00 | 8.74E-04 | 7.74E-04 | 5.14E-03 | 7.56E-03 | 2.89E-03 | 2.89E-03 | rs4072738 | 2.21E-08 |  |
| SCZ | *TRANK1* | 89 | 4.76E-01 | 3.43E-06 | 7.59E-07 | 8.66E-06 | 2.38E-05 | 4.96E-06 | 4.96E-06 | rs9876421 | 7.82E-12 |  |
| SCZ | *TRIM38* | 77 | 1.00E+00 | 5.71E-05 | 4.72E-05 | 1.65E-14 | 2.88E-10 | 1.48E-14 | 1.48E-14 | rs35050608 | 5.08E-21 |  |
| SCZ | *TRIM8* | 71 | 1.00E+00 | 5.75E-01 | 4.63E-01 | 1.87E-03 | 1.89E-03 | 7.76E-04 | 7.76E-04 | rs10883761 | 2.39E-09 |  |
| SCZ | *TRMT61A* | 62 | 1.00E+00 | 1.86E-04 | 1.22E-04 | 3.74E-07 | 6.94E-07 | 2.40E-07 | 2.40E-07 | rs35229468 | 5.95E-13 |  |
| SCZ | *TSNARE1* | 137 | 1.00E+00 | 9.15E-03 | 4.74E-03 | 1.79E-06 | 1.78E-06 | 1.53E-06 | 1.53E-06 | rs4129585 | 7.77E-13 |  |
| SCZ | *TSR1* | 88 | 1.00E+00 | 1.67E-02 | 9.32E-03 | 6.30E-03 | 1.17E-02 | 3.47E-03 | 3.47E-03 | rs4523957 | 1.19E-08 |  |
| SCZ | *TSSK6* | 51 | 1.00E+00 | 6.00E-04 | 3.63E-04 | 2.05E-03 | 5.63E-03 | 1.29E-03 | 1.29E-03 | rs7245983 | 1.03E-08 |  |
| SCZ | *TWF2* | 70 | 1.00E+00 | 1.00E+00 | 1.00E+00 | 3.84E-02 | 3.88E-02 | 2.07E-02 | 2.07E-02 | rs7622851 | 4.21E-08 |  |
| SCZ | *TYW5* | 69 | 1.00E+00 | 1.63E-05 | 8.13E-06 | 3.95E-08 | 2.09E-07 | 2.74E-08 | 2.74E-08 | rs2949006 | 3.17E-14 |  |
| SCZ | *UBLCP1* | 77 | 1.48E-04 | 9.22E-04 | 1.00E+00 | 1.00E+00 | 1.00E+00 | 1.00E+00 | 1.00E+00 | rs2546892 | 4.11E-06 | √ |
| SCZ | *UNC45A* | 70 | 1.00E+00 | 1.57E-02 | 4.39E-03 | 6.75E-06 | 6.67E-06 | 6.66E-06 | 6.66E-06 | rs4702 | 5.73E-12 |  |
| SCZ | *VPS29* | 29 | 1.00E+00 | 4.19E-02 | 2.70E-02 | 8.43E-02 | 1.26E-01 | 6.01E-02 | 6.01E-02 | rs34840178 | 4.33E-07 | √ |
| SCZ | *VPS33A* | 59 | 1.00E+00 | 1.47E-02 | 6.01E-03 | 1.00E+00 | 1.00E+00 | 1.00E+00 | 1.00E+00 | rs7952868 | 5.55E-05 | √ |
| SCZ | *VPS37B* | 54 | 1.00E+00 | 1.00E+00 | 1.00E+00 | 1.76E-03 | 2.16E-03 | 1.14E-03 | 1.14E-03 | rs883562 | 2.99E-09 |  |
| SCZ | *VPS45* | 59 | 1.00E+00 | 2.07E-04 | 1.86E-04 | 7.05E-04 | 1.50E-03 | 3.28E-04 | 3.28E-04 | rs2319280 | 9.64E-10 |  |
| SCZ | *VRK2* | 136 | 1.00E+00 | 2.32E-04 | 5.49E-05 | 5.94E-03 | 1.25E-02 | 3.27E-03 | 3.27E-03 | rs2678915 | 3.75E-09 |  |
| SCZ | *VSIG2* | 70 | 1.00E+00 | 1.89E-05 | 4.78E-06 | 2.29E-02 | 1.89E-02 | 9.32E-03 | 9.32E-03 | rs55661361 | 2.11E-11 |  |
| SCZ | *WBP2NL* | 64 | 1.00E+00 | 1.20E-04 | 9.18E-05 | 1.46E-02 | 2.60E-02 | 7.23E-03 | 7.23E-03 | rs77441740 | 2.65E-08 |  |
| SCZ | *WDR73* | 68 | 1.00E+00 | 7.29E-05 | 4.50E-05 | 2.56E-04 | 2.12E-04 | 1.07E-04 | 1.07E-04 | rs17531523 | 8.97E-11 |  |
| SCZ | *WDR82* | 75 | 1.00E+00 | 1.00E+00 | 1.00E+00 | 4.12E-02 | 3.54E-02 | 2.19E-02 | 2.19E-02 | rs7622851 | 4.21E-08 |  |
| SCZ | *XPNPEP3* | 28 | 1.00E+00 | 1.00E+00 | 1.00E+00 | 1.97E-03 | 1.18E-03 | 1.97E-03 | 1.97E-03 | rs926914 | 4.18E-09 |  |
| SCZ | *XRCC3* | 71 | 1.00E+00 | 1.17E-06 | 9.63E-07 | 5.04E-06 | 1.29E-05 | 3.12E-06 | 3.12E-06 | rs4900592 | 7.19E-12 |  |
| SCZ | *YJEFN3* | 57 | 1.00E+00 | 3.25E-04 | 1.57E-04 | 2.47E-03 | 6.66E-03 | 1.60E-03 | 1.60E-03 | rs7245983 | 1.03E-08 |  |
| SCZ | *YPEL3* | 55 | 1.00E+00 | 6.38E-03 | 5.97E-03 | 9.03E-04 | 1.63E-03 | 7.44E-04 | 7.44E-04 | rs3814880 | 2.76E-09 |  |
| SCZ | *YPEL4* | 60 | 1.00E+00 | 9.14E-02 | 3.18E-02 | 2.71E-01 | 2.71E-01 | 1.49E-01 | 1.49E-01 | rs9420 | 5.23E-07 | √ |
| SCZ | *YTHDF2* | 65 | 1.00E+00 | 1.00E+00 | 1.00E+00 | 4.33E-02 | 2.23E-02 | 4.20E-02 | 4.20E-02 | rs533123 | 3.96E-08 |  |
| SCZ | *ZBED4* | 68 | 1.00E+00 | 3.31E-02 | 2.84E-02 | 1.69E-01 | 2.15E-01 | 7.68E-02 | 7.68E-02 | rs5770755 | 4.91E-07 | √ |
| SCZ | *ZC3H7B* | 65 | 1.00E+00 | 8.62E-01 | 7.77E-01 | 3.86E-03 | 5.29E-03 | 3.01E-03 | 3.01E-03 | rs136402 | 7.18E-09 |  |
| SCZ | *ZDHHC5* | 53 | 1.00E+00 | 8.75E-02 | 4.00E-02 | 1.99E-01 | 2.27E-01 | 1.14E-01 | 1.14E-01 | rs9420 | 5.23E-07 | √ |
| SCZ | *ZFYVE21* | 74 | 1.00E+00 | 9.83E-07 | 9.01E-07 | 4.20E-06 | 1.32E-05 | 2.71E-06 | 2.71E-06 | rs4900592 | 7.19E-12 |  |
| SCZ | *ZMAT2* | 81 | 1.00E+00 | 4.04E-02 | 3.66E-02 | 1.53E-01 | 3.12E-01 | 8.65E-02 | 8.65E-02 | rs62384243 | 7.96E-07 | √ |
| SCZ | *ZNF365* | 216 | 1.00E+00 | 4.17E-04 | 8.42E-05 | 1.00E+00 | 1.00E+00 | 1.00E+00 | 1.00E+00 | rs72829007 | 6.19E-09 |  |
| SCZ | *ZNF408* | 25 | 1.00E+00 | 9.42E-05 | 6.85E-05 | 2.91E-04 | 2.46E-04 | 1.66E-04 | 1.66E-04 | rs7108770 | 2.47E-10 |  |
| SCZ | *ZNF536* | 137 | 1.00E+00 | 1.56E-02 | 4.24E-03 | 1.08E-01 | 9.60E-02 | 6.33E-02 | 6.33E-02 | rs2053079 | 1.48E-08 |  |
| SCZ | *ZNF592* | 117 | 1.00E+00 | 6.07E-02 | 2.29E-02 | 2.87E-03 | 2.89E-03 | 1.41E-03 | 1.00E+00 | rs17598603 | 1.97E-09 |  |
| SCZ | *ZNF804A* | 116 | 1.00E+00 | 8.61E-02 | 6.41E-02 | 7.28E-05 | 6.71E-05 | 6.02E-05 | 6.02E-05 | rs4666990 | 9.95E-12 |  |
| SCZ | *ZPBP2* | 49 | 5.34E-04 | 7.35E-04 | 1.00E+00 | 1.00E+00 | 1.00E+00 | 1.00E+00 | 1.00E+00 | rs17676191 | 0.000708 | √ |
| SCZ | *ZSWIM6* | 133 | 1.00E+00 | 2.16E-06 | 1.24E-06 | 1.08E-06 | 1.51E-06 | 5.56E-07 | 1.00E+00 | rs4391122 | 5.93E-13 |  |

Note: novel: defined by whether the gene contained GWAS significant SNPs (*P*_GWAS_ < 5×10^-8^).

## Table S6. Identified genes associated with the four plasma lipid traits

| traits | Gene | *m* | BT | SKATO | SKAT | Simes | minP | HMP | ACAT | Best SNP | *P*_GWAS_ | novel |
| --- | --- | --- | --- | --- | --- | --- | --- | --- | --- | --- | --- | --- |
| HDL | *ABCD2* | 9 | 1.00E+00 | 4.12E-06 | 1.19E-06 | 1.00E+00 | 1.00E+00 | 1.00E+00 | 1.00E+00 | rs201804365 | 9.62E-04 | √ |
| HDL | *ABHD2* | 9 | 1.00E+00 | 1.57E-08 | 1.96E-09 | 3.86E-03 | 4.51E-03 | 7.52E-24 | 7.50E-24 | rs139271800 | 1.17E-04 | √ |
| HDL | *ABHD8* | 22 | 8.53E-01 | 2.81E-65 | 1.03E-65 | 8.00E-03 | 2.04E-02 | 2.97E-135 | 2.96E-135 | rs200795418 | 2.04E-04 | √ |
| HDL | *AC010536.1* | 23 | 1.00E+00 | 3.82E-09 | 4.78E-10 | 1.67E-02 | 4.93E-03 | 4.48E-15 | 4.47E-15 | rs138881765 | 6.03E-03 | √ |
| HDL | *AC010536.2* | 22 | 1.00E+00 | 7.02E-07 | 8.77E-08 | 1.76E-03 | 1.72E-02 | 4.34E-15 | 4.33E-15 | rs138881765 | 6.03E-03 | √ |
| HDL | *AC063977.1* | 17 | 1.54E-01 | 1.51E-65 | 1.89E-66 | 3.08E-03 | 2.25E-03 | 2.24E-135 | 2.23E-135 | rs114957460 | 3.56E-03 | √ |
| HDL | *AC090616.2* | 12 | 1.00E+00 | 1.31E-35 | 1.64E-36 | 1.29E-02 | 1.16E-02 | 3.85E-100 | 3.84E-100 | rs118103228 | 6.30E-02 | √ |
| HDL | *AC118344.1* | 16 | 1.74E-01 | 1.28E-65 | 1.60E-66 | 2.33E-02 | 4.23E-03 | 2.14E-135 | 2.14E-135 | rs200667255 | 1.38E-03 | √ |
| HDL | *AC133485.1* | 13 | 1.00E+00 | 3.20E-76 | 4.00E-77 | 1.21E-02 | 2.39E-02 | 0.00E+00 | 1.61E-01 | rs118056264 | 3.96E-01 | √ |
| HDL | *ACTC1* | 9 | 1.00E+00 | 1.57E-08 | 1.96E-09 | 2.69E-02 | 1.64E-03 | 7.52E-24 | 7.50E-24 | rs200022337 | 3.30E-03 | √ |
| HDL | *ACY1* | 9 | 1.00E+00 | 2.18E-02 | 9.20E-03 | 1.00E+00 | 1.00E+00 | 1.00E+00 | 1.00E+00 | rs419752 | 6.13E-11 |  |
| HDL | *ADRM1* | 5 | 1.00E+00 | 8.50E-18 | 1.06E-18 | 2.70E-02 | 2.08E-02 | 3.84E-71 | 3.83E-71 | rs140927746 | 9.48E-04 | √ |
| HDL | *AL035696.1* | 102 | 1.00E+00 | 1.00E+00 | 1.00E+00 | 5.43E-02 | 6.10E-02 | 4.46E-02 | 4.45E-02 | rs202216214 | 1.72E-02 | √ |
| HDL | *ANGPTL4* | 16 | 1.74E-01 | 1.28E-65 | 1.60E-66 | 2.65E-02 | 1.76E-02 | 2.14E-135 | 2.14E-135 | rs116843064 | 4.79E-146 |  |
| HDL | *ANKS1A* | 97 | 1.00E+00 | 1.00E+00 | 1.00E+00 | 5.96E-02 | 6.67E-02 | 4.22E-02 | 4.21E-02 | rs41270076 | 6.37E-04 | √ |
| HDL | *ANKS3* | 7 | 1.00E+00 | 1.19E-72 | 1.48E-73 | 2.40E-02 | 1.15E-02 | 0.00E+00 | 1.61E-01 | rs78074706 | 1.00E-09 |  |
| HDL | *ANO8* | 22 | 8.53E-01 | 2.81E-65 | 1.03E-65 | 7.50E-03 | 5.04E-03 | 2.97E-135 | 2.96E-135 | rs200795418 | 2.04E-04 | √ |
| HDL | *ANXA2* | 9 | 1.00E+00 | 1.57E-08 | 1.96E-09 | 2.16E-02 | 5.52E-03 | 7.52E-24 | 7.50E-24 | rs138557547 | 3.93E-02 | √ |
| HDL | *ARID3B* | 7 | 1.00E+00 | 7.41E-09 | 9.26E-10 | 2.46E-02 | 4.87E-03 | 5.98E-24 | 5.97E-24 | rs61747605 | 9.42E-03 | √ |
| HDL | *ARL8B* | 4 | 1.00E+00 | 3.77E-02 | 9.96E-03 | 3.90E-01 | 3.82E-01 | 3.77E-01 | 3.76E-01 | rs151099815 | 3.27E-02 | √ |
| HDL | *ARNTL2* | 14 | 4.71E-02 | 7.18E-07 | 1.10E-06 | 1.00E+00 | 1.00E+00 | 1.00E+00 | 1.00E+00 | rs138825924 | 1.42E-03 | √ |
| HDL | *ASB16* | 7 | 1.00E+00 | 3.37E-47 | 4.21E-48 | 3.13E-02 | 1.28E-02 | 2.27E-100 | 2.27E-100 | rs72836561 | 8.12E-111 |  |
| HDL | *ATF7* | 3 | 1.52E-01 | 1.01E-02 | 2.19E-02 | 1.00E+00 | 1.00E+00 | 1.00E+00 | 1.00E+00 | rs34652380 | 6.09E-03 | √ |
| HDL | *ATP5G1* | 14 | 1.00E+00 | 5.80E-35 | 7.25E-36 | 2.28E-02 | 1.95E-02 | 4.47E-100 | 4.46E-100 | rs201757928 | 2.19E-03 | √ |
| HDL | *ATP6V0D1* | 19 | 1.00E+00 | 3.87E-08 | 4.83E-09 | 2.76E-02 | 1.59E-02 | 4.52E-18 | 4.51E-18 | rs4986970 | 7.15E-29 |  |
| HDL | *ATP8B1* | 2 | 2.47E-37 | 9.37E-79 | 1.82E-79 | 1.82E-02 | 1.68E-02 | 1.05E-88 | 1.05E-88 | rs141813907 | 3.50E-02 | √ |
| HDL | *AXL* | 16 | 1.74E-01 | 1.28E-65 | 1.60E-66 | 6.33E-03 | 1.36E-02 | 2.14E-135 | 2.14E-135 | rs141929169 | 3.14E-05 | √ |
| HDL | *B3GALT4* | 101 | 1.00E+00 | 1.00E+00 | 1.00E+00 | 5.44E-02 | 6.38E-02 | 4.43E-02 | 4.42E-02 | rs471942 | 1.91E-04 | √ |
| HDL | *BTN3A2* | 103 | 1.00E+00 | 1.00E+00 | 1.00E+00 | 7.07E-02 | 5.73E-02 | 4.56E-02 | 4.55E-02 | rs142951857 | 6.27E-04 | √ |
| HDL | *BUB1B* | 8 | 1.00E+00 | 2.42E-08 | 3.03E-09 | 2.23E-02 | 1.38E-02 | 6.85E-24 | 6.83E-24 | rs149315160 | 2.42E-03 | √ |
| HDL | *BVES* | 95 | 1.00E+00 | 1.00E+00 | 1.00E+00 | 5.72E-02 | 6.36E-02 | 4.38E-02 | 4.37E-02 | rs35952696 | 5.69E-03 | √ |
| HDL | *C16orf54* | 3 | 1.00E+00 | 2.00E-07 | 6.20E-08 | 6.97E-02 | 4.40E-02 | 4.29E-02 | 4.28E-02 | rs139384013 | 5.77E-03 | √ |
| HDL | *C16orf70* | 9 | 1.00E+00 | 2.50E-74 | 3.13E-75 | 1.60E-02 | 1.95E-02 | 0.00E+00 | 1.61E-01 | rs150369207 | 5.91E-26 |  |
| HDL | *C17orf103* | 12 | 1.00E+00 | 1.31E-35 | 1.64E-36 | 8.93E-03 | 7.98E-05 | 3.85E-100 | 3.84E-100 | rs138394714 | 1.22E-02 | √ |
| HDL | *C19orf29-AS1* | 18 | 2.68E-02 | 1.80E-54 | 2.25E-55 | 2.84E-02 | 9.67E-04 | 2.39E-135 | 2.38E-135 | rs145723843 | 1.97E-05 | √ |
| HDL | *C3orf14* | 9 | 1.00E+00 | 2.18E-02 | 9.20E-03 | 1.00E+00 | 1.00E+00 | 1.00E+00 | 1.00E+00 | rs140777402 | 1.89E-02 | √ |
| HDL | *C3orf64* | 9 | 1.00E+00 | 2.18E-02 | 9.20E-03 | 1.00E+00 | 1.00E+00 | 1.00E+00 | 1.00E+00 | rs201345267 | 9.56E-03 | √ |
| HDL | *C6orf170* | 99 | 1.00E+00 | 1.00E+00 | 1.00E+00 | 5.01E-02 | 6.74E-02 | 4.79E-02 | 4.78E-02 | rs112983358 | 3.61E-02 | √ |
| HDL | *C6orf203* | 98 | 1.00E+00 | 1.00E+00 | 1.00E+00 | 7.20E-02 | 4.99E-02 | 4.46E-02 | 4.45E-02 | rs139190201 | 3.89E-03 | √ |
| HDL | *CACNA1H* | 18 | 1.00E+00 | 1.14E-09 | 1.43E-10 | 1.26E-02 | 1.30E-02 | 3.40E-15 | 3.40E-15 | rs11642118 | 6.32E-04 | √ |
| HDL | *CAMK1* | 2 | 7.66E-03 | 7.87E-03 | 5.36E-01 | 1.00E+00 | 1.00E+00 | 1.00E+00 | 1.00E+00 | rs28942092 | 6.06E-03 | √ |
| HDL | *CBX5* | 7 | 1.00E+00 | 3.48E-07 | 5.00E-08 | 1.00E+00 | 1.00E+00 | 1.00E+00 | 1.00E+00 | rs34652380 | 6.09E-03 | √ |
| HDL | *CCDC101* | 19 | 1.00E+00 | 3.87E-08 | 4.83E-09 | 1.77E-02 | 7.89E-03 | 4.52E-18 | 4.51E-18 | rs117987062 | 9.36E-03 | √ |
| HDL | *CCDC159* | 16 | 1.74E-01 | 1.28E-65 | 1.60E-66 | 3.90E-03 | 3.60E-03 | 2.14E-135 | 2.14E-135 | rs145464906 | 5.04E-18 |  |
| HDL | *CCL25* | 28 | 3.39E-01 | 6.92E-41 | 8.64E-42 | 3.01E-02 | 3.64E-03 | 3.81E-135 | 3.81E-135 | rs116843064 | 4.79E-146 |  |
| HDL | *CCNDBP1* | 12 | 1.00E+00 | 5.88E-02 | 2.45E-02 | 1.82E-02 | 2.11E-02 | 1.01E-23 | 1.01E-23 | rs55707100 | 2.26E-34 |  |
| HDL | *CDH13* | 3 | 1.00E+00 | 2.00E-07 | 6.20E-08 | 4.40E-02 | 6.72E-02 | 4.29E-02 | 4.28E-02 | rs142821762 | 4.68E-03 | √ |
| HDL | *CDK2AP1* | 8 | 3.72E-01 | 3.53E-05 | 8.53E-06 | 1.00E+00 | 1.00E+00 | 1.00E+00 | 1.00E+00 | rs75175797 | 2.36E-10 |  |
| HDL | *CDT1* | 12 | 1.00E+00 | 2.94E-04 | 2.65E-04 | 5.68E-04 | 1.48E-02 | 2.97E-18 | 2.96E-18 | rs147221198 | 5.10E-04 | √ |
| HDL | *CENPQ* | 102 | 1.00E+00 | 1.00E+00 | 1.00E+00 | 6.12E-02 | 5.85E-02 | 4.46E-02 | 4.45E-02 | rs145011872 | 1.99E-02 | √ |
| HDL | *CES4A* | 10 | 1.00E+00 | 2.58E-08 | 3.22E-09 | 5.41E-03 | 1.64E-03 | 6.46E-08 | 6.45E-08 | rs75193761 | 1.51E-18 |  |
| HDL | *CES5A* | 8 | 1.00E+00 | 3.02E-83 | 3.77E-84 | 1.78E-02 | 8.34E-03 | 0.00E+00 | 1.61E-01 | rs117887370 | 1.08E-02 | √ |
| HDL | *CHD9* | 15 | 1.00E+00 | 4.02E-08 | 5.02E-09 | 2.62E-02 | 9.15E-03 | 9.94E-08 | 9.92E-08 | rs139067427 | 8.86E-03 | √ |
| HDL | *CLEC18B* | 5 | 1.00E+00 | 3.05E-11 | 3.81E-12 | 6.23E-03 | 2.18E-02 | 1.26E-18 | 1.25E-18 | rs200545714 | 2.12E-02 | √ |
| HDL | *CLEC4M* | 22 | 8.53E-01 | 2.81E-65 | 1.03E-65 | 1.78E-02 | 2.11E-02 | 2.97E-135 | 2.96E-135 | rs140448414 | 1.87E-03 | √ |
| HDL | *COMMD4* | 7 | 1.00E+00 | 7.41E-09 | 9.26E-10 | 5.20E-03 | 3.62E-03 | 5.98E-24 | 5.97E-24 | rs148486660 | 5.56E-04 | √ |
| HDL | *CPNE7* | 12 | 1.00E+00 | 2.94E-04 | 2.65E-04 | 8.23E-03 | 1.03E-02 | 2.97E-18 | 2.96E-18 | rs2270416 | 6.46E-03 | √ |
| HDL | *CREBL2* | 9 | 1.00E+00 | 4.12E-06 | 1.19E-06 | 1.00E+00 | 1.00E+00 | 1.00E+00 | 1.00E+00 | rs140182370 | 5.42E-04 | √ |
| HDL | *CSNK1G1* | 8 | 1.00E+00 | 2.42E-08 | 3.03E-09 | 2.45E-02 | 1.03E-03 | 6.85E-24 | 6.83E-24 | rs1802376 | 2.36E-03 | √ |
| HDL | *CSNK2B* | 101 | 1.00E+00 | 1.00E+00 | 1.00E+00 | 6.83E-02 | 6.50E-02 | 4.45E-02 | 4.45E-02 | rs41273264 | 2.72E-13 |  |
| HDL | *CTAGE1* | 4 | 1.60E-24 | 6.48E-85 | 2.93E-85 | 2.11E-02 | 2.44E-02 | 1.86E-88 | 1.85E-88 | rs151176879 | 3.16E-02 | √ |
| HDL | *CTD-2258A20.4* | 3 | 1.09E-10 | 2.47E-74 | 2.47E-74 | 2.49E-02 | 8.08E-03 | 0.00E+00 | 1.61E-01 | rs34621310 | 3.85E-04 | √ |
| HDL | *CYB5B* | 8 | 1.00E+00 | 8.80E-73 | 1.10E-73 | 2.61E-02 | 1.96E-02 | 0.00E+00 | 1.61E-01 | rs76116020 | 7.19E-09 |  |
| HDL | *CYLD* | 22 | 1.00E+00 | 7.02E-07 | 8.77E-08 | 2.31E-03 | 6.06E-03 | 4.34E-15 | 4.33E-15 | rs104895452 | 2.32E-03 | √ |
| HDL | *DBP* | 19 | 1.33E-01 | 1.43E-69 | 3.63E-70 | 5.88E-03 | 1.81E-02 | 2.57E-135 | 2.57E-135 | rs200190654 | 3.97E-03 | √ |
| HDL | *DDR1* | 100 | 1.00E+00 | 1.00E+00 | 1.00E+00 | 5.01E-02 | 6.59E-02 | 4.40E-02 | 4.40E-02 | rs150846158 | 3.70E-04 | √ |
| HDL | *DDX19A* | 22 | 1.00E+00 | 7.02E-07 | 8.77E-08 | 6.76E-04 | 2.48E-02 | 4.34E-15 | 4.33E-15 | rs147433916 | 4.70E-03 | √ |
| HDL | *DECR2* | 9 | 1.00E+00 | 2.50E-74 | 3.13E-75 | 1.46E-02 | 6.76E-04 | 0.00E+00 | 1.61E-01 | rs2308312 | 5.20E-06 | √ |
| HDL | *DHX33* | 12 | 1.00E+00 | 1.31E-35 | 1.64E-36 | 1.69E-02 | 2.41E-02 | 3.85E-100 | 3.84E-100 | rs145046442 | 8.49E-04 | √ |
| HDL | *DNAAF1* | 22 | 1.00E+00 | 7.02E-07 | 8.77E-08 | 1.16E-02 | 3.82E-03 | 4.34E-15 | 4.33E-15 | rs142821762 | 4.68E-03 | √ |
| HDL | *DOC2A* | 13 | 1.00E+00 | 2.33E-03 | 2.14E-03 | 4.96E-03 | 1.72E-02 | 3.31E-18 | 3.30E-18 | rs139384013 | 5.77E-03 | √ |
| HDL | *DUSP22* | 102 | 1.00E+00 | 1.00E+00 | 1.00E+00 | 5.49E-02 | 5.64E-02 | 4.45E-02 | 4.45E-02 | rs202216214 | 1.72E-02 | √ |
| HDL | *EEF2K* | 2 | 1.41E-06 | 8.89E-07 | 5.46E-06 | 3.32E-02 | 2.63E-02 | 4.20E-03 | 4.20E-03 | rs140364490 | 1.34E-03 | √ |
| HDL | *EID2* | 22 | 8.53E-01 | 2.81E-65 | 1.03E-65 | 2.46E-02 | 6.45E-03 | 2.97E-135 | 2.96E-135 | rs144304735 | 2.70E-03 | √ |
| HDL | *ELL3* | 7 | 1.00E+00 | 7.41E-09 | 9.26E-10 | 1.39E-03 | 4.25E-03 | 5.98E-24 | 5.97E-24 | rs55707100 | 2.26E-34 |  |
| HDL | *ELP2* | 3 | 8.70E-31 | 7.79E-92 | 9.73E-93 | 3.00E-02 | 1.47E-02 | 1.32E-88 | 1.32E-88 | rs188061556 | 5.01E-02 | √ |
| HDL | *EMP2* | 12 | 1.00E+00 | 4.53E-80 | 5.66E-81 | 5.37E-03 | 1.56E-02 | 0.00E+00 | 1.61E-01 | rs141751547 | 1.11E-02 | √ |
| HDL | *EMR2* | 22 | 8.53E-01 | 2.81E-65 | 1.03E-65 | 1.04E-03 | 1.34E-02 | 2.97E-135 | 2.96E-135 | rs16979636 | 2.35E-04 | √ |
| HDL | *ENY2* | 3 | 7.98E-218 | 1.31E-235 | 3.73E-224 | 3.08E-02 | 7.71E-03 | 5.33E-144 | 5.32E-144 | rs138118364 | 7.30E-03 | √ |
| HDL | *ERCC4* | 11 | 1.00E+00 | 2.22E-04 | 2.02E-04 | 1.53E-02 | 9.43E-03 | 2.73E-18 | 2.72E-18 | rs145037209 | 1.55E-02 | √ |
| HDL | *FAHD1* | 13 | 1.00E+00 | 3.20E-76 | 4.00E-77 | 1.59E-02 | 1.76E-03 | 0.00E+00 | 1.61E-01 | rs45517419 | 4.11E-04 | √ |
| HDL | *FAM101B* | 14 | 1.00E+00 | 5.80E-35 | 7.25E-36 | 1.09E-02 | 1.98E-02 | 4.47E-100 | 4.46E-100 | rs191778127 | 1.46E-03 | √ |
| HDL | *FAM173A* | 23 | 1.00E+00 | 3.82E-09 | 4.78E-10 | 1.65E-02 | 9.50E-03 | 4.48E-15 | 4.47E-15 | rs145644880 | 9.40E-05 | √ |
| HDL | *FAM86A* | 14 | 1.00E+00 | 1.08E-05 | 4.27E-06 | 1.23E-02 | 1.87E-03 | 9.47E-08 | 9.45E-08 | rs78074706 | 1.00E-09 |  |
| HDL | *FBXL19* | 22 | 1.00E+00 | 7.02E-07 | 8.77E-08 | 2.80E-04 | 1.67E-02 | 4.34E-15 | 4.33E-15 | rs35376811 | 7.04E-04 | √ |
| HDL | *FBXO30* | 97 | 1.00E+00 | 1.00E+00 | 1.00E+00 | 4.45E-02 | 4.31E-02 | 4.22E-02 | 4.21E-02 | rs142581081 | 5.12E-02 | √ |
| HDL | *FHOD1* | 13 | 1.00E+00 | 1.89E-13 | 2.37E-14 | 2.85E-02 | 1.59E-02 | 8.81E-08 | 8.80E-08 | rs150369207 | 5.91E-26 |  |
| HDL | *FICD* | 9 | 1.00E+00 | 4.12E-06 | 1.19E-06 | 1.00E+00 | 1.00E+00 | 1.00E+00 | 1.00E+00 | rs35664142 | 5.60E-04 | √ |
| HDL | *FOPNL* | 13 | 1.00E+00 | 2.33E-03 | 2.14E-03 | 2.61E-02 | 1.31E-02 | 3.31E-18 | 3.30E-18 | noRS | 2.32E-03 | √ |
| HDL | *FOXD4L6* | 4 | 1.00E+00 | 9.68E-01 | 3.47E-01 | 5.87E-04 | 1.29E-02 | 3.13E-14 | 3.12E-14 | rs200937603 | 6.76E-01 | √ |
| HDL | *FSD2* | 11 | 1.00E+00 | 2.71E-04 | 3.39E-05 | 1.35E-02 | 3.27E-03 | 9.34E-24 | 9.33E-24 | rs188892600 | 1.27E-02 | √ |
| HDL | *FUK* | 19 | 1.00E+00 | 3.87E-08 | 4.83E-09 | 8.58E-03 | 2.42E-02 | 4.52E-18 | 4.51E-18 | rs147433916 | 4.70E-03 | √ |
| HDL | *GALNS* | 11 | 1.00E+00 | 2.94E-79 | 3.67E-80 | 7.15E-03 | 1.59E-02 | 0.00E+00 | 1.61E-01 | rs147221198 | 5.10E-04 | √ |
| HDL | *GAS2L3* | 11 | 1.00E+00 | 8.46E-03 | 1.09E-03 | 1.00E+00 | 1.00E+00 | 1.00E+00 | 1.00E+00 | rs116925463 | 1.11E-02 | √ |
| HDL | *GNL1* | 103 | 1.00E+00 | 1.00E+00 | 1.00E+00 | 6.62E-02 | 6.84E-02 | 4.49E-02 | 4.49E-02 | noRS | 1.15E-03 | √ |
| HDL | *GNMT* | 100 | 1.00E+00 | 1.00E+00 | 1.00E+00 | 4.84E-02 | 6.35E-02 | 4.36E-02 | 4.36E-02 | rs146188256 | 1.53E-04 | √ |
| HDL | *GNPTG* | 19 | 1.00E+00 | 3.87E-08 | 4.83E-09 | 1.96E-02 | 1.24E-03 | 4.52E-18 | 4.51E-18 | rs11642118 | 6.32E-04 | √ |
| HDL | *GPT2* | 11 | 1.00E+00 | 3.89E-05 | 4.86E-06 | 9.04E-03 | 1.78E-02 | 2.92E-18 | 2.92E-18 | rs200288545 | 2.26E-02 | √ |
| HDL | *GTPBP2* | 97 | 1.00E+00 | 1.00E+00 | 1.00E+00 | 5.12E-02 | 6.31E-02 | 4.44E-02 | 4.44E-02 | rs200525225 | 2.37E-03 | √ |
| HDL | *HIGD1A* | 9 | 1.00E+00 | 2.18E-02 | 9.20E-03 | 1.00E+00 | 1.00E+00 | 1.00E+00 | 1.00E+00 | rs199854093 | 6.18E-03 | √ |
| HDL | *HINT3* | 102 | 1.00E+00 | 1.00E+00 | 1.00E+00 | 6.94E-02 | 6.92E-02 | 4.46E-02 | 4.45E-02 | rs116306619 | 1.19E-01 | √ |
| HDL | *HIST1H2AJ* | 100 | 1.00E+00 | 1.00E+00 | 1.00E+00 | 4.50E-02 | 5.37E-02 | 4.40E-02 | 4.39E-02 | rs41269281 | 2.83E-03 | √ |
| HDL | *HIST1H2BG* | 100 | 1.00E+00 | 1.00E+00 | 1.00E+00 | 6.98E-02 | 5.88E-02 | 4.89E-02 | 4.88E-02 | rs142951857 | 6.27E-04 | √ |
| HDL | *HIST1H3G* | 101 | 1.00E+00 | 1.00E+00 | 1.00E+00 | 6.55E-02 | 6.26E-02 | 4.48E-02 | 4.47E-02 | rs142951857 | 6.27E-04 | √ |
| HDL | *HLA-DPB1* | 101 | 1.00E+00 | 1.00E+00 | 1.00E+00 | 6.05E-02 | 6.36E-02 | 4.81E-02 | 4.81E-02 | rs142107957 | 1.40E-03 | √ |
| HDL | *HLF* | 12 | 1.00E+00 | 1.31E-35 | 1.64E-36 | 1.78E-02 | 1.53E-03 | 3.85E-100 | 3.84E-100 | rs115766743 | 4.03E-02 | √ |
| HDL | *HMHA1* | 22 | 8.53E-01 | 2.81E-65 | 1.03E-65 | 2.06E-02 | 6.33E-03 | 2.97E-135 | 2.96E-135 | rs138363826 | 1.31E-03 | √ |
| HDL | *HOXC5* | 11 | 1.00E+00 | 1.34E-04 | 1.70E-05 | 1.00E+00 | 1.00E+00 | 1.00E+00 | 1.00E+00 | rs34652380 | 6.09E-03 | √ |
| HDL | *HSPB9* | 15 | 1.00E+00 | 5.92E-38 | 1.07E-38 | 1.75E-02 | 1.34E-02 | 4.78E-100 | 4.78E-100 | rs145447726 | 3.37E-04 | √ |
| HDL | *IGDCC4* | 9 | 1.00E+00 | 1.57E-08 | 1.96E-09 | 1.51E-02 | 7.26E-04 | 7.52E-24 | 7.50E-24 | rs202245263 | 1.04E-02 | √ |
| HDL | *IGFALS* | 7 | 1.00E+00 | 1.19E-72 | 1.48E-73 | 2.87E-03 | 1.04E-02 | 0.00E+00 | 1.61E-01 | rs45517419 | 4.11E-04 | √ |
| HDL | *IMP4* | 7 | 1.00E+00 | 3.68E-03 | 4.60E-04 | 2.28E-02 | 5.13E-03 | 4.42E-27 | 4.41E-27 | rs150073700 | 5.20E-03 | √ |
| HDL | *IP6K1* | 9 | 1.00E+00 | 2.18E-02 | 9.20E-03 | 1.00E+00 | 1.00E+00 | 1.00E+00 | 1.00E+00 | rs61731329 | 1.15E-03 | √ |
| HDL | *IQCH* | 8 | 1.00E+00 | 2.42E-08 | 3.03E-09 | 1.90E-02 | 2.24E-02 | 6.85E-24 | 6.83E-24 | rs36096355 | 2.38E-04 | √ |
| HDL | *ITGAX* | 12 | 2.03E-02 | 2.48E-07 | 8.38E-07 | 1.00E+00 | 1.00E+00 | 1.00E+00 | 1.00E+00 | rs35376811 | 7.04E-04 | √ |
| HDL | *ITGB1BP1* | 7 | 1.00E+00 | 3.68E-03 | 4.60E-04 | 2.57E-02 | 1.12E-02 | 4.42E-27 | 4.41E-27 | rs61754178 | 1.03E-02 | √ |
| HDL | *ITPA* | 5 | 1.00E+00 | 8.50E-18 | 1.06E-18 | 2.05E-03 | 9.35E-03 | 3.84E-71 | 3.83E-71 | rs141472746 | 2.59E-04 | √ |
| HDL | *JAK2* | 5 | 1.00E+00 | 1.00E+00 | 7.99E-01 | 1.45E-02 | 1.73E-02 | 3.53E-14 | 3.53E-14 | noRS | 8.52E-06 | √ |
| HDL | *KARS* | 9 | 1.00E+00 | 2.50E-74 | 3.13E-75 | 3.76E-05 | 6.46E-03 | 0.00E+00 | 1.61E-01 | rs62619985 | 4.71E-04 | √ |
| HDL | *KATNA1* | 98 | 1.00E+00 | 1.00E+00 | 1.00E+00 | 7.44E-02 | 6.68E-02 | 4.46E-02 | 4.45E-02 | rs184775357 | 7.95E-03 | √ |
| HDL | *KDM2B* | 12 | 1.00E+00 | 1.56E-06 | 2.19E-07 | 1.00E+00 | 1.00E+00 | 1.00E+00 | 1.00E+00 | rs184565788 | 6.27E-04 | √ |
| HDL | *KIF22* | 8 | 1.00E+00 | 3.02E-83 | 3.77E-84 | 2.25E-02 | 2.35E-02 | 0.00E+00 | 1.61E-01 | rs139384013 | 5.77E-03 | √ |
| HDL | *KREMEN2* | 10 | 1.00E+00 | 1.53E-77 | 1.91E-78 | 1.65E-02 | 6.69E-03 | 0.00E+00 | 1.61E-01 | rs150076785 | 4.36E-04 | √ |
| HDL | *KRT18* | 7 | 1.00E+00 | 3.48E-07 | 5.00E-08 | 1.00E+00 | 1.00E+00 | 1.00E+00 | 1.00E+00 | rs139954107 | 6.36E-03 | √ |
| HDL | *LAMA2* | 102 | 1.00E+00 | 1.00E+00 | 1.00E+00 | 7.65E-02 | 6.94E-02 | 4.53E-02 | 4.53E-02 | rs147185142 | 9.75E-03 | √ |
| HDL | *LEMD2* | 100 | 1.00E+00 | 1.00E+00 | 1.00E+00 | 5.38E-02 | 5.74E-02 | 4.36E-02 | 4.36E-02 | rs471942 | 1.91E-04 | √ |
| HDL | *LLPH* | 7 | 1.00E+00 | 6.83E-03 | 2.13E-03 | 1.00E+00 | 1.00E+00 | 1.00E+00 | 1.00E+00 | rs141160603 | 9.13E-04 | √ |
| HDL | *LMF1* | 5 | 1.00E+00 | 3.05E-11 | 3.81E-12 | 1.01E-02 | 1.55E-02 | 1.26E-18 | 1.25E-18 | rs145644880 | 9.40E-05 | √ |
| HDL | *LPAR1* | 5 | 1.00E+00 | 1.00E+00 | 6.34E-01 | 4.53E-03 | 8.44E-03 | 4.02E-14 | 4.02E-14 | rs41278435 | 1.25E-03 | √ |
| HDL | *LPCAT4* | 9 | 1.00E+00 | 1.57E-08 | 1.96E-09 | 1.64E-02 | 2.11E-02 | 7.52E-24 | 7.50E-24 | rs202088512 | 1.96E-02 | √ |
| HDL | *LRRC16A* | 104 | 1.00E+00 | 1.00E+00 | 1.00E+00 | 7.84E-02 | 5.08E-02 | 4.79E-02 | 4.78E-02 | rs146860658 | 7.25E-03 | √ |
| HDL | *LRRIQ1* | 3 | 1.52E-01 | 1.01E-02 | 2.19E-02 | 1.00E+00 | 1.00E+00 | 1.00E+00 | 1.00E+00 | rs201810554 | 4.78E-03 | √ |
| HDL | *LRRK2* | 3 | 1.52E-01 | 1.01E-02 | 2.19E-02 | 1.00E+00 | 1.00E+00 | 1.00E+00 | 1.00E+00 | rs35507033 | 1.50E-03 | √ |
| HDL | *LYRM1* | 11 | 1.00E+00 | 2.94E-79 | 3.67E-80 | 3.04E-02 | 2.10E-02 | 0.00E+00 | 1.61E-01 | rs141137569 | 1.27E-03 | √ |
| HDL | *LYSMD2* | 8 | 1.00E+00 | 2.42E-08 | 3.03E-09 | 7.28E-04 | 8.10E-03 | 6.85E-24 | 6.83E-24 | noRS | 2.24E-02 | √ |
| HDL | *MAN1A1* | 100 | 1.00E+00 | 1.00E+00 | 1.00E+00 | 6.52E-02 | 4.65E-02 | 4.40E-02 | 4.40E-02 | rs34213490 | 2.83E-02 | √ |
| HDL | *MAP3K2* | 7 | 1.00E+00 | 3.68E-03 | 4.60E-04 | 9.56E-03 | 1.62E-02 | 4.42E-27 | 4.41E-27 | rs201694464 | 1.51E-02 | √ |
| HDL | *MAPK6* | 7 | 1.00E+00 | 7.41E-09 | 9.26E-10 | 2.59E-02 | 1.14E-02 | 5.98E-24 | 5.97E-24 | rs61731219 | 5.72E-04 | √ |
| HDL | *MARVELD3* | 5 | 1.00E+00 | 3.05E-11 | 3.81E-12 | 1.85E-02 | 2.49E-02 | 1.26E-18 | 1.25E-18 | rs139187106 | 6.77E-03 | √ |
| HDL | *MB21D1* | 102 | 1.00E+00 | 1.00E+00 | 1.00E+00 | 4.86E-02 | 5.89E-02 | 4.45E-02 | 4.45E-02 | rs113690012 | 1.46E-04 | √ |
| HDL | *MED24* | 6 | 1.00E+00 | 9.91E-47 | 2.58E-47 | 1.42E-02 | 2.84E-03 | 1.98E-100 | 1.97E-100 | rs11556624 | 2.45E-06 | √ |
| HDL | *MEF2A* | 7 | 1.00E+00 | 7.41E-09 | 9.26E-10 | 2.81E-02 | 6.49E-05 | 5.98E-24 | 5.97E-24 | rs151128482 | 3.06E-03 | √ |
| HDL | *MESP1* | 9 | 1.00E+00 | 1.57E-08 | 1.96E-09 | 6.88E-03 | 1.46E-02 | 7.52E-24 | 7.50E-24 | rs139271800 | 1.17E-04 | √ |
| HDL | *MGST1* | 10 | 1.00E+00 | 4.93E-06 | 8.37E-07 | 1.00E+00 | 1.00E+00 | 1.00E+00 | 1.00E+00 | rs111967344 | 1.13E-02 | √ |
| HDL | *MLL2* | 7 | 1.00E+00 | 3.48E-07 | 5.00E-08 | 1.00E+00 | 1.00E+00 | 1.00E+00 | 1.00E+00 | rs1126930 | 6.98E-07 | √ |
| HDL | *MLLT4-AS1* | 107 | 1.00E+00 | 1.00E+00 | 1.00E+00 | 6.65E-02 | 6.50E-02 | 4.70E-02 | 4.69E-02 | rs138998311 | 1.08E-28 |  |
| HDL | *MON2* | 3 | 1.52E-01 | 1.01E-02 | 2.19E-02 | 1.00E+00 | 1.00E+00 | 1.00E+00 | 1.00E+00 | rs139869747 | 3.07E-02 | √ |
| HDL | *MPV17L* | 14 | 1.00E+00 | 1.08E-05 | 4.27E-06 | 2.97E-02 | 1.77E-02 | 9.47E-08 | 9.45E-08 | noRS | 2.32E-03 | √ |
| HDL | *MRPL12* | 12 | 1.00E+00 | 1.31E-35 | 1.64E-36 | 2.78E-02 | 1.60E-02 | 3.85E-100 | 3.84E-100 | rs139309995 | 6.85E-04 | √ |
| HDL | *MRPS34* | 22 | 1.00E+00 | 7.02E-07 | 8.77E-08 | 1.77E-02 | 2.12E-02 | 4.34E-15 | 4.33E-15 | rs45517419 | 4.11E-04 | √ |
| HDL | *MSL1* | 6 | 1.00E+00 | 9.91E-47 | 2.58E-47 | 5.93E-03 | 9.28E-03 | 1.98E-100 | 1.97E-100 | rs11556624 | 2.45E-06 | √ |
| HDL | *MT3* | 19 | 1.00E+00 | 3.87E-08 | 4.83E-09 | 2.47E-02 | 1.37E-02 | 4.52E-18 | 4.51E-18 | rs5880 | 0.00E+00 |  |
| HDL | *MTRNR2L4* | 2 | 1.41E-06 | 8.89E-07 | 5.46E-06 | 1.25E-02 | 1.25E-02 | 4.20E-03 | 4.20E-03 | rs147461021 | 2.39E-03 | √ |
| HDL | *MYH11* | 14 | 1.00E+00 | 1.08E-05 | 4.27E-06 | 1.09E-02 | 1.96E-02 | 9.47E-08 | 9.45E-08 | noRS | 2.32E-03 | √ |
| HDL | *MYLK4* | 101 | 1.00E+00 | 1.00E+00 | 1.00E+00 | 4.82E-02 | 6.55E-02 | 4.45E-02 | 4.45E-02 | rs143394328 | 2.83E-04 | √ |
| HDL | *NCOA6* | 5 | 1.00E+00 | 3.58E-18 | 4.48E-19 | 8.92E-03 | 1.55E-03 | 3.64E-71 | 3.63E-71 | rs146138949 | 3.81E-03 | √ |
| HDL | *NOMO3* | 5 | 1.00E+00 | 3.05E-11 | 3.81E-12 | 1.49E-02 | 9.17E-03 | 1.26E-18 | 1.25E-18 | rs114349489 | 5.78E-03 | √ |
| HDL | *NPEPL1* | 6 | 1.00E+00 | 3.94E-18 | 4.92E-19 | 8.81E-03 | 2.51E-02 | 4.12E-71 | 4.11E-71 | rs201438798 | 9.43E-03 | √ |
| HDL | *NPIPL1* | 9 | 1.00E+00 | 3.52E-83 | 4.40E-84 | 2.72E-02 | 2.33E-02 | 0.00E+00 | 1.61E-01 | rs117987062 | 9.36E-03 | √ |
| HDL | *NRM* | 102 | 1.00E+00 | 1.00E+00 | 1.00E+00 | 4.53E-02 | 5.21E-02 | 4.48E-02 | 4.47E-02 | rs150846158 | 3.70E-04 | √ |
| HDL | *ODF3L1* | 11 | 1.00E+00 | 2.71E-04 | 3.39E-05 | 2.34E-02 | 2.00E-02 | 9.34E-24 | 9.33E-24 | rs148486660 | 5.56E-04 | √ |
| HDL | *ORAI3* | 11 | 1.00E+00 | 2.22E-04 | 2.02E-04 | 9.28E-03 | 2.29E-02 | 2.73E-18 | 2.72E-18 | rs35376811 | 7.04E-04 | √ |
| HDL | *ORMDL3* | 8 | 1.00E+00 | 5.04E-46 | 6.30E-47 | 6.26E-04 | 3.94E-03 | 2.40E-100 | 2.40E-100 | rs11556624 | 2.45E-06 | √ |
| HDL | *PAPD5* | 13 | 1.00E+00 | 3.20E-76 | 4.00E-77 | 5.10E-03 | 1.66E-02 | 0.00E+00 | 1.61E-01 | rs104895452 | 2.32E-03 | √ |
| HDL | *PAQR4* | 7 | 1.00E+00 | 1.19E-72 | 1.48E-73 | 3.12E-02 | 1.80E-02 | 0.00E+00 | 1.61E-01 | rs150076785 | 4.36E-04 | √ |
| HDL | *PDK2* | 15 | 1.00E+00 | 8.30E-28 | 1.04E-28 | 2.45E-02 | 1.29E-02 | 4.70E-100 | 4.69E-100 | rs150024578 | 7.35E-03 | √ |
| HDL | *PDPK1* | 3 | 1.00E+00 | 2.00E-07 | 6.20E-08 | 6.14E-02 | 4.66E-02 | 4.29E-02 | 4.28E-02 | rs45517419 | 4.11E-04 | √ |
| HDL | *PDPR* | 6 | 1.00E+00 | 5.83E-08 | 7.28E-09 | 1.03E-01 | 9.86E-02 | 8.63E-02 | 8.61E-02 | rs75820607 | 2.12E-02 | √ |
| HDL | *PGP* | 4 | 1.00E+00 | 2.24E-11 | 2.80E-12 | 2.19E-02 | 2.61E-03 | 1.08E-18 | 1.08E-18 | rs45517419 | 4.11E-04 | √ |
| HDL | *PHIP* | 101 | 1.00E+00 | 1.00E+00 | 1.00E+00 | 7.02E-02 | 6.15E-02 | 4.48E-02 | 4.47E-02 | rs35338066 | 4.58E-03 | √ |
| HDL | *PHLPP2* | 13 | 1.00E+00 | 1.89E-13 | 2.37E-14 | 1.45E-02 | 9.18E-03 | 8.81E-08 | 8.80E-08 | rs139187106 | 6.77E-03 | √ |
| HDL | *PKIB* | 101 | 1.00E+00 | 1.00E+00 | 1.00E+00 | 6.45E-02 | 5.53E-02 | 4.48E-02 | 4.47E-02 | rs150118372 | 1.10E-02 | √ |
| HDL | *PLA2G15* | 24 | 1.00E+00 | 1.80E-17 | 2.25E-18 | 1.79E-03 | 2.01E-02 | 5.96E-18 | 5.95E-18 | rs4986970 | 7.15E-29 |  |
| HDL | *PLCB2* | 7 | 1.00E+00 | 7.41E-09 | 9.26E-10 | 1.01E-02 | 1.07E-02 | 5.98E-24 | 5.97E-24 | rs149315160 | 2.42E-03 | √ |
| HDL | *PLEKHG1* | 101 | 1.00E+00 | 1.00E+00 | 1.00E+00 | 5.70E-02 | 4.51E-02 | 4.45E-02 | 4.45E-02 | rs144848429 | 4.21E-02 | √ |
| HDL | *PLLP* | 21 | 1.00E+00 | 4.52E-08 | 5.65E-09 | 2.37E-02 | 1.92E-02 | 5.09E-18 | 5.08E-18 | rs5880 | 0.00E+00 |  |
| HDL | *PLTP* | 7 | 1.28E-09 | 3.97E-23 | 9.90E-24 | 5.97E-03 | 1.80E-02 | 3.78E-71 | 3.77E-71 | rs144016582 | 3.97E-03 | √ |
| HDL | *PMFBP1* | 10 | 1.00E+00 | 3.97E-83 | 4.96E-84 | 2.35E-02 | 4.65E-03 | 0.00E+00 | 1.61E-01 | rs201202896 | 1.02E-02 | √ |
| HDL | *PODXL2* | 9 | 1.00E+00 | 2.18E-02 | 9.20E-03 | 1.00E+00 | 1.00E+00 | 1.00E+00 | 1.00E+00 | rs199793381 | 1.51E-02 | √ |
| HDL | *POLR3K* | 13 | 1.00E+00 | 2.33E-03 | 2.14E-03 | 1.12E-02 | 1.82E-03 | 3.31E-18 | 3.30E-18 | rs2308312 | 5.20E-06 | √ |
| HDL | *PPARD* | 105 | 1.00E+00 | 1.00E+00 | 1.00E+00 | 6.00E-02 | 6.29E-02 | 4.63E-02 | 4.62E-02 | rs41270076 | 6.37E-04 | √ |
| HDL | *PPP1R15A* | 22 | 8.53E-01 | 2.81E-65 | 1.03E-65 | 7.58E-03 | 7.71E-04 | 2.97E-135 | 2.96E-135 | rs200190654 | 3.97E-03 | √ |
| HDL | *PPP1R3G* | 98 | 1.00E+00 | 1.00E+00 | 1.00E+00 | 6.42E-02 | 5.12E-02 | 4.46E-02 | 4.45E-02 | rs22662 | 6.49E-03 | √ |
| HDL | *PRCD* | 12 | 1.00E+00 | 1.31E-35 | 1.64E-36 | 2.06E-02 | 8.78E-03 | 3.85E-100 | 3.84E-100 | rs62621822 | 6.45E-04 | √ |
| HDL | *PRDM13* | 104 | 1.00E+00 | 1.00E+00 | 1.00E+00 | 5.83E-02 | 6.20E-02 | 4.54E-02 | 4.54E-02 | rs62434070 | 1.39E-02 | √ |
| HDL | *PRICKLE1* | 10 | 1.00E+00 | 1.20E-04 | 1.50E-05 | 1.00E+00 | 1.00E+00 | 1.00E+00 | 1.00E+00 | rs138452760 | 1.29E-02 | √ |
| HDL | *PRRC2A* | 106 | 1.00E+00 | 1.00E+00 | 1.00E+00 | 7.65E-02 | 4.99E-02 | 4.61E-02 | 4.60E-02 | rs41273264 | 2.72E-13 |  |
| HDL | *PRSS21* | 23 | 1.00E+00 | 3.82E-09 | 4.78E-10 | 9.91E-03 | 2.20E-02 | 4.48E-15 | 4.47E-15 | rs150076785 | 4.36E-04 | √ |
| HDL | *PRSS50* | 9 | 1.00E+00 | 2.18E-02 | 9.20E-03 | 1.00E+00 | 1.00E+00 | 1.00E+00 | 1.00E+00 | rs17079425 | 3.36E-04 | √ |
| HDL | *PTCRA* | 102 | 1.00E+00 | 1.00E+00 | 1.00E+00 | 6.82E-02 | 4.88E-02 | 4.48E-02 | 4.47E-02 | rs146188256 | 1.53E-04 | √ |
| HDL | *PTK7* | 101 | 1.00E+00 | 1.00E+00 | 1.00E+00 | 4.80E-02 | 6.03E-02 | 4.45E-02 | 4.45E-02 | rs146188256 | 1.53E-04 | √ |
| HDL | *PTPN1* | 5 | 1.00E+00 | 8.50E-18 | 1.06E-18 | 3.01E-02 | 2.01E-02 | 3.84E-71 | 3.83E-71 | rs141643081 | 3.96E-05 | √ |
| HDL | *RAB7A* | 2 | 1.00E+00 | 7.61E-03 | 2.92E-03 | 2.10E-01 | 2.01E-01 | 1.88E-01 | 1.87E-01 | rs142476058 | 8.01E-03 | √ |
| HDL | *RAP1B* | 9 | 1.00E+00 | 9.38E-06 | 1.35E-06 | 1.00E+00 | 1.00E+00 | 1.00E+00 | 1.00E+00 | rs146132026 | 3.46E-02 | √ |
| HDL | *RBL2* | 4 | 1.00E+00 | 3.44E-07 | 7.56E-08 | 7.79E-02 | 7.61E-02 | 5.37E-02 | 5.36E-02 | rs139067427 | 8.86E-03 | √ |
| HDL | *RDBP* | 101 | 1.00E+00 | 1.00E+00 | 1.00E+00 | 4.57E-02 | 5.65E-02 | 4.48E-02 | 4.47E-02 | rs41273264 | 2.72E-13 |  |
| HDL | *RHBDF1* | 10 | 1.00E+00 | 8.91E-06 | 6.73E-06 | 1.62E-02 | 1.45E-02 | 2.56E-18 | 2.55E-18 | rs2308312 | 5.20E-06 | √ |
| HDL | *RNASET2* | 103 | 1.00E+00 | 1.00E+00 | 1.00E+00 | 5.78E-02 | 7.07E-02 | 4.57E-02 | 4.56E-02 | rs200677090 | 1.45E-02 | √ |
| HDL | *RNF183* | 4 | 1.00E+00 | 9.68E-01 | 3.47E-01 | 1.45E-02 | 3.01E-03 | 3.13E-14 | 3.12E-14 | rs145999003 | 5.80E-04 | √ |
| HDL | *RNPS1* | 22 | 1.00E+00 | 7.02E-07 | 8.77E-08 | 1.64E-02 | 5.52E-03 | 4.34E-15 | 4.33E-15 | rs45517419 | 4.11E-04 | √ |
| HDL | *ROCK1* | 3 | 8.70E-31 | 7.79E-92 | 9.73E-93 | 2.28E-02 | 1.64E-02 | 1.32E-88 | 1.32E-88 | rs184926533 | 1.13E-01 | √ |
| HDL | *ROMO1* | 5 | 1.00E+00 | 8.50E-18 | 1.06E-18 | 1.98E-02 | 9.51E-03 | 3.84E-71 | 3.83E-71 | rs200450712 | 6.65E-04 | √ |
| HDL | *RP1-180E22.3* | 97 | 1.00E+00 | 1.00E+00 | 1.00E+00 | 6.83E-02 | 6.17E-02 | 4.59E-02 | 4.58E-02 | rs11543266 | 1.28E-03 | √ |
| HDL | *RP11-105C19.1* | 11 | 1.00E+00 | 1.20E-14 | 1.50E-15 | 2.22E-02 | 1.75E-02 | 2.85E-18 | 2.84E-18 | rs137985480 | 3.99E-03 | √ |
| HDL | *RP11-1166P10.9* | 5 | 1.00E+00 | 3.05E-11 | 3.81E-12 | 1.81E-02 | 1.60E-02 | 1.26E-18 | 1.25E-18 | rs118056264 | 3.96E-01 | √ |
| HDL | *RP11-166B2.1* | 11 | 1.00E+00 | 8.27E-03 | 4.59E-03 | 1.00E+00 | 1.00E+00 | 1.00E+00 | 1.00E+00 | rs201653834 | 1.01E-02 | √ |
| HDL | *RP11-234B24.2* | 15 | 1.00E+00 | 1.28E-01 | 4.15E-02 | 1.00E+00 | 1.00E+00 | 1.00E+00 | 1.00E+00 | rs148232276 | 1.39E-03 | √ |
| HDL | *RP11-234B24.6* | 3 | 1.52E-01 | 1.01E-02 | 2.19E-02 | 1.00E+00 | 1.00E+00 | 1.00E+00 | 1.00E+00 | rs148232276 | 1.39E-03 | √ |
| HDL | *RP11-266L9.5* | 11 | 1.00E+00 | 2.22E-04 | 2.02E-04 | 1.56E-02 | 3.38E-03 | 2.73E-18 | 2.72E-18 | rs61742943 | 5.01E-03 | √ |
| HDL | *RP11-276H1.3* | 11 | 1.00E+00 | 2.22E-04 | 2.02E-04 | 1.67E-02 | 1.06E-02 | 2.73E-18 | 2.72E-18 | rs145376245 | 2.97E-02 | √ |
| HDL | *RP11-285A1.1* | 8 | 1.00E+00 | 2.42E-08 | 3.03E-09 | 1.25E-02 | 5.51E-03 | 6.85E-24 | 6.83E-24 | rs56095004 | 3.08E-03 | √ |
| HDL | *RP11-343C2.3* | 17 | 1.00E+00 | 4.50E-12 | 5.63E-13 | 5.58E-03 | 1.90E-02 | 3.16E-15 | 3.15E-15 | rs76116020 | 7.19E-09 |  |
| HDL | *RP11-357N13.7* | 14 | 1.00E+00 | 1.08E-05 | 4.27E-06 | 2.99E-02 | 1.39E-02 | 9.47E-08 | 9.45E-08 | rs139067427 | 8.86E-03 | √ |
| HDL | *RP11-388M20.9* | 23 | 1.00E+00 | 3.82E-09 | 4.78E-10 | 6.43E-03 | 1.59E-02 | 4.48E-15 | 4.47E-15 | rs35376811 | 7.04E-04 | √ |
| HDL | *RP11-426C22.5* | 16 | 1.00E+00 | 3.50E-10 | 4.38E-11 | 3.00E-03 | 1.23E-02 | 3.04E-15 | 3.03E-15 | rs117987062 | 9.36E-03 | √ |
| HDL | *RP11-457M11.5* | 100 | 1.00E+00 | 1.00E+00 | 1.00E+00 | 4.59E-02 | 6.26E-02 | 4.36E-02 | 4.36E-02 | rs142951857 | 6.27E-04 | √ |
| HDL | *RP11-481J2.2* | 10 | 1.00E+00 | 3.97E-83 | 4.96E-84 | 2.59E-02 | 1.09E-02 | 0.00E+00 | 1.61E-01 | rs150122367 | 2.88E-03 | √ |
| HDL | *RP11-545I5.3* | 94 | 1.00E+00 | 1.00E+00 | 1.00E+00 | 6.10E-02 | 4.98E-02 | 4.39E-02 | 4.39E-02 | rs142581081 | 5.12E-02 | √ |
| HDL | *RP11-57A19.2* | 16 | 1.00E+00 | 3.27E-04 | 2.49E-04 | 6.65E-03 | 3.86E-03 | 4.01E-18 | 4.00E-18 | rs113061700 | 2.77E-02 | √ |
| HDL | *RP11-686F15.2* | 7 | 1.00E+00 | 6.83E-03 | 2.13E-03 | 1.00E+00 | 1.00E+00 | 1.00E+00 | 1.00E+00 | rs34652380 | 6.09E-03 | √ |
| HDL | *RP11-817O13.8* | 11 | 1.00E+00 | 2.71E-04 | 3.39E-05 | 2.68E-02 | 1.50E-02 | 9.34E-24 | 9.33E-24 | rs148486660 | 5.56E-04 | √ |
| HDL | *RP11-875O11.2* | 6 | 7.01E-108 | 5.05E-205 | 2.52E-203 | 1.85E-02 | 3.72E-03 | 9.79E-144 | 9.77E-144 | rs201537177 | 1.04E-02 | √ |
| HDL | *RPL27* | 14 | 1.00E+00 | 5.80E-35 | 7.25E-36 | 2.69E-02 | 1.56E-02 | 4.47E-100 | 4.46E-100 | rs56116165 | 1.54E-04 | √ |
| HDL | *RPP25* | 9 | 1.00E+00 | 1.57E-08 | 1.96E-09 | 2.06E-02 | 1.43E-02 | 7.52E-24 | 7.50E-24 | rs148486660 | 5.56E-04 | √ |
| HDL | *RPSAP58* | 20 | 3.15E-03 | 2.92E-44 | 3.65E-45 | 1.26E-02 | 1.58E-02 | 2.60E-135 | 2.59E-135 | rs200272849 | 7.57E-02 | √ |
| HDL | *SAMD14* | 14 | 1.00E+00 | 1.46E-37 | 3.51E-38 | 3.30E-04 | 4.29E-03 | 4.43E-100 | 4.42E-100 | rs150024578 | 7.35E-03 | √ |
| HDL | *SATB1* | 9 | 1.00E+00 | 2.18E-02 | 9.20E-03 | 1.00E+00 | 1.00E+00 | 1.00E+00 | 1.00E+00 | rs148337599 | 2.23E-01 | √ |
| HDL | *SCAND3* | 96 | 1.00E+00 | 1.00E+00 | 1.00E+00 | 7.03E-02 | 5.10E-02 | 4.38E-02 | 4.37E-02 | rs41269281 | 2.83E-03 | √ |
| HDL | *SCML4* | 103 | 1.00E+00 | 1.00E+00 | 1.00E+00 | 6.35E-02 | 6.16E-02 | 4.49E-02 | 4.48E-02 | rs9480830 | 3.63E-02 | √ |
| HDL | *SCNN1B* | 2 | 1.41E-06 | 8.89E-07 | 5.46E-06 | 1.85E-02 | 1.91E-02 | 4.20E-03 | 4.20E-03 | rs112597134 | 5.37E-03 | √ |
| HDL | *SEC11A* | 7 | 1.00E+00 | 7.41E-09 | 9.26E-10 | 2.57E-02 | 2.37E-02 | 5.98E-24 | 5.97E-24 | rs147924097 | 1.52E-03 | √ |
| HDL | *SLC2A3* | 11 | 8.32E-01 | 1.17E-05 | 2.82E-06 | 1.00E+00 | 1.00E+00 | 1.00E+00 | 1.00E+00 | rs74810750 | 1.03E-02 | √ |
| HDL | *SLC35B2* | 100 | 1.00E+00 | 1.00E+00 | 1.00E+00 | 6.02E-02 | 6.27E-02 | 4.40E-02 | 4.40E-02 | rs146833594 | 1.59E-03 | √ |
| HDL | *SLFN12L* | 14 | 1.00E+00 | 5.80E-35 | 7.25E-36 | 2.85E-03 | 2.50E-02 | 4.47E-100 | 4.46E-100 | rs201598708 | 1.03E-02 | √ |
| HDL | *SNHG5* | 102 | 1.00E+00 | 1.00E+00 | 1.00E+00 | 5.74E-02 | 6.11E-02 | 4.46E-02 | 4.45E-02 | rs41271629 | 5.33E-03 | √ |
| HDL | *SNN* | 10 | 1.00E+00 | 8.91E-06 | 6.73E-06 | 2.11E-02 | 8.99E-03 | 2.56E-18 | 2.55E-18 | rs201653834 | 1.01E-02 | √ |
| HDL | *SNRPB2* | 5 | 8.79E-03 | 1.55E-20 | 1.94E-21 | 6.74E-03 | 1.16E-02 | 3.75E-71 | 3.74E-71 | rs115426082 | 3.76E-02 | √ |
| HDL | *SNRPF* | 8 | 1.00E+00 | 5.25E-07 | 6.73E-08 | 1.00E+00 | 1.00E+00 | 1.00E+00 | 1.00E+00 | rs141724326 | 2.92E-03 | √ |
| HDL | *SPHK1* | 14 | 1.00E+00 | 1.46E-37 | 3.51E-38 | 2.59E-02 | 1.37E-02 | 4.43E-100 | 4.42E-100 | rs150994435 | 7.44E-05 | √ |
| HDL | *SPIRE2* | 7 | 1.00E+00 | 1.19E-72 | 1.48E-73 | 1.89E-02 | 7.10E-03 | 0.00E+00 | 1.61E-01 | rs1110400 | 9.80E-03 | √ |
| HDL | *SUPT6H* | 6 | 1.00E+00 | 9.91E-47 | 2.58E-47 | 2.67E-02 | 1.51E-02 | 1.98E-100 | 1.97E-100 | rs200744136 | 1.05E-03 | √ |
| HDL | *SYNJ2* | 99 | 1.00E+00 | 1.00E+00 | 1.00E+00 | 5.12E-02 | 6.17E-02 | 4.75E-02 | 4.75E-02 | rs201799213 | 1.72E-02 | √ |
| HDL | *TCF19* | 103 | 1.00E+00 | 1.00E+00 | 1.00E+00 | 5.75E-02 | 6.27E-02 | 4.54E-02 | 4.53E-02 | rs41273264 | 2.72E-13 |  |
| HDL | *TCFL5* | 7 | 1.28E-09 | 3.97E-23 | 9.90E-24 | 8.95E-03 | 1.61E-02 | 3.78E-71 | 3.77E-71 | rs34304654 | 3.60E-04 | √ |
| HDL | *THBS2* | 99 | 1.00E+00 | 1.00E+00 | 1.00E+00 | 6.29E-02 | 6.44E-02 | 4.51E-02 | 4.50E-02 | rs140852957 | 4.38E-03 | √ |
| HDL | *THEMIS* | 97 | 1.00E+00 | 1.00E+00 | 1.00E+00 | 4.46E-02 | 5.00E-02 | 4.44E-02 | 4.44E-02 | rs201266835 | 2.68E-02 | √ |
| HDL | *THUMPD1* | 22 | 1.00E+00 | 7.02E-07 | 8.77E-08 | 2.89E-03 | 3.35E-03 | 4.34E-15 | 4.33E-15 | rs141137569 | 1.27E-03 | √ |
| HDL | *TIAM2* | 101 | 1.00E+00 | 1.00E+00 | 1.00E+00 | 6.12E-02 | 6.97E-02 | 4.45E-02 | 4.44E-02 | rs148543891 | 1.50E-02 | √ |
| HDL | *TMC5* | 12 | 1.00E+00 | 2.94E-04 | 2.65E-04 | 2.77E-02 | 1.48E-02 | 2.97E-18 | 2.96E-18 | rs150938236 | 1.29E-03 | √ |
| HDL | *TMCO7* | 13 | 1.00E+00 | 2.33E-03 | 2.14E-03 | 1.79E-02 | 4.26E-03 | 3.31E-18 | 3.30E-18 | rs76116020 | 7.19E-09 |  |
| HDL | *TMEM204* | 11 | 1.00E+00 | 2.94E-79 | 3.67E-80 | 8.98E-03 | 2.61E-03 | 0.00E+00 | 1.61E-01 | rs11642118 | 6.32E-04 | √ |
| HDL | *TMEM219* | 19 | 1.00E+00 | 3.87E-08 | 4.83E-09 | 4.47E-03 | 4.03E-03 | 4.52E-18 | 4.51E-18 | rs139384013 | 5.77E-03 | √ |
| HDL | *TP53I3* | 7 | 1.00E+00 | 3.68E-03 | 4.60E-04 | 1.72E-02 | 2.10E-02 | 4.42E-27 | 4.41E-27 | rs3208747 | 4.98E-03 | √ |
| HDL | *TRAK1* | 9 | 1.00E+00 | 2.18E-02 | 9.20E-03 | 1.00E+00 | 1.00E+00 | 1.00E+00 | 1.00E+00 | rs199854093 | 6.18E-03 | √ |
| HDL | *TRAPPC2L* | 6 | 1.00E+00 | 3.07E-72 | 3.84E-73 | 1.78E-03 | 2.24E-03 | 0.00E+00 | 1.61E-01 | rs147221198 | 5.10E-04 | √ |
| HDL | *TTLL6* | 8 | 1.00E+00 | 2.42E-46 | 4.09E-47 | 1.03E-02 | 1.25E-02 | 2.63E-100 | 2.63E-100 | rs201757928 | 2.19E-03 | √ |
| HDL | *TUFM* | 22 | 1.00E+00 | 7.02E-07 | 8.77E-08 | 2.27E-02 | 1.24E-02 | 4.34E-15 | 4.33E-15 | rs117987062 | 9.36E-03 | √ |
| HDL | *TXNL4B* | 22 | 1.00E+00 | 7.02E-07 | 8.77E-08 | 1.34E-02 | 1.95E-02 | 4.34E-15 | 4.33E-15 | rs201202896 | 1.02E-02 | √ |
| HDL | *UBR1* | 9 | 1.00E+00 | 1.57E-08 | 1.96E-09 | 1.43E-02 | 2.61E-03 | 7.52E-24 | 7.50E-24 | rs55707100 | 2.26E-34 |  |
| HDL | *UBR5* | 3 | 7.98E-218 | 1.31E-235 | 3.73E-224 | 5.34E-03 | 1.05E-02 | 5.33E-144 | 5.32E-144 | rs28566564 | 2.16E-02 | √ |
| HDL | *UBXN7* | 9 | 1.00E+00 | 2.18E-02 | 9.20E-03 | 1.00E+00 | 1.00E+00 | 1.00E+00 | 1.00E+00 | rs143348369 | 4.90E-04 | √ |
| HDL | *USP6* | 12 | 1.00E+00 | 1.31E-35 | 1.64E-36 | 2.17E-02 | 2.39E-02 | 3.85E-100 | 3.84E-100 | rs145046442 | 8.49E-04 | √ |
| HDL | *VSIG10L* | 22 | 8.53E-01 | 2.81E-65 | 1.03E-65 | 2.86E-02 | 2.42E-03 | 2.97E-135 | 2.96E-135 | rs114957460 | 3.56E-03 | √ |
| HDL | *WTAP* | 102 | 1.00E+00 | 1.00E+00 | 1.00E+00 | 5.63E-02 | 4.70E-02 | 4.46E-02 | 4.45E-02 | rs144100305 | 3.74E-04 | √ |
| HDL | *WWTR1* | 9 | 1.00E+00 | 2.18E-02 | 9.20E-03 | 1.00E+00 | 1.00E+00 | 1.00E+00 | 1.00E+00 | rs146475504 | 7.25E-03 | √ |
| HDL | *ZC3H3* | 5 | 2.44E-108 | 1.51E-157 | 1.88E-158 | 2.61E-02 | 4.65E-03 | 8.51E-144 | 8.49E-144 | rs199941693 | 3.11E-04 | √ |
| HDL | *ZDHHC1* | 22 | 1.00E+00 | 7.02E-07 | 8.77E-08 | 7.74E-03 | 1.05E-02 | 4.34E-15 | 4.33E-15 | rs150369207 | 5.91E-26 |  |
| HDL | *ZDHHC7* | 7 | 1.00E+00 | 1.19E-72 | 1.48E-73 | 9.36E-03 | 1.43E-02 | 0.00E+00 | 1.61E-01 | rs9934891 | 1.96E-02 | √ |
| HDL | *ZNF184* | 87 | 1.00E+00 | 1.00E+00 | 1.00E+00 | 5.20E-02 | 6.11E-02 | 4.05E-02 | 4.05E-02 | rs200353027 | 5.90E-03 | √ |
| HDL | *ZNF19* | 20 | 1.00E+00 | 2.44E-07 | 3.05E-08 | 1.24E-02 | 9.36E-03 | 3.94E-15 | 3.93E-15 | rs139187106 | 6.77E-03 | √ |
| HDL | *ZNF233* | 16 | 1.74E-01 | 1.28E-65 | 1.60E-66 | 4.75E-03 | 9.38E-03 | 2.14E-135 | 2.14E-135 | noRS | 3.42E-09 |  |
| HDL | *ZNF333* | 22 | 8.53E-01 | 2.81E-65 | 1.03E-65 | 2.57E-02 | 2.03E-02 | 2.97E-135 | 2.96E-135 | rs16979636 | 2.35E-04 | √ |
| HDL | *ZNF343* | 6 | 1.00E+00 | 3.94E-18 | 4.92E-19 | 1.98E-02 | 3.01E-03 | 4.12E-71 | 4.11E-71 | rs141669275 | 6.27E-04 | √ |
| HDL | *ZNF512B* | 6 | 2.59E-01 | 5.60E-17 | 7.00E-18 | 1.22E-02 | 6.27E-04 | 4.69E-71 | 4.68E-71 | rs201732460 | 5.57E-04 | √ |
| HDL | *ZNF558* | 19 | 8.72E-06 | 3.12E-72 | 2.41E-72 | 2.36E-02 | 2.46E-02 | 2.77E-135 | 2.76E-135 | rs116843064 | 4.79E-146 |  |
| HDL | *ZNF581* | 22 | 8.53E-01 | 2.81E-65 | 1.03E-65 | 1.50E-02 | 1.23E-02 | 2.97E-135 | 2.96E-135 | rs188779067 | 1.89E-03 | √ |
| HDL | *ZNF599* | 28 | 3.39E-01 | 6.92E-41 | 8.64E-42 | 8.08E-03 | 2.80E-03 | 3.81E-135 | 3.81E-135 | rs138113607 | 6.90E-03 | √ |
| HDL | *ZNF699* | 16 | 1.74E-01 | 1.28E-65 | 1.60E-66 | 2.51E-02 | 2.28E-02 | 2.14E-135 | 2.14E-135 | rs75774843 | 3.64E-04 | √ |
| HDL | *ZNF720* | 11 | 1.00E+00 | 2.22E-04 | 2.02E-04 | 1.48E-02 | 1.79E-02 | 2.73E-18 | 2.72E-18 | rs138066479 | 2.66E-03 | √ |
| HDL | *ZNF771* | 21 | 1.00E+00 | 4.52E-08 | 5.65E-09 | 1.08E-03 | 1.62E-02 | 5.09E-18 | 5.08E-18 | rs141977752 | 5.53E-03 | √ |
| HDL | *ZNF780A* | 16 | 1.37E-04 | 1.46E-44 | 1.83E-45 | 2.90E-02 | 9.44E-03 | 2.08E-135 | 2.08E-135 | rs144304735 | 2.70E-03 | √ |
| LDL | *ABCA9* | 7 | 6.34E-08 | 3.86E-29 | 1.12E-29 | 2.24E-02 | 2.44E-02 | 1.80E-42 | 1.80E-42 | rs77542162 | 4.83E-50 |  |
| LDL | *AC051649.16* | 4 | 1.00E+00 | 2.24E-04 | 7.66E-05 | 3.80E-02 | 2.63E-02 | 1.43E-02 | 1.43E-02 | rs141482597 | 2.59E-03 | √ |
| LDL | *ADAR* | 19 | 1.13E-10 | 4.10E-40 | 3.06E-40 | 1.50E-02 | 1.43E-02 | 6.51E-42 | 6.51E-42 | rs141845046 | 2.62E-03 | √ |
| LDL | *ADCK4* | 15 | 1.00E+00 | 3.18E-04 | 4.41E-05 | 8.16E-02 | 6.16E-02 | 5.21E-02 | 5.21E-02 | rs200888669 | 2.54E-03 | √ |
| LDL | *ADRM1* | 5 | 1.00E+00 | 1.00E+00 | 1.00E+00 | 1.71E-02 | 1.08E-02 | 3.96E-05 | 3.95E-05 | rs148515817 | 9.22E-06 | √ |
| LDL | *AGPAT1* | 18 | 1.00E+00 | 1.00E+00 | 1.00E+00 | 1.60E-02 | 1.11E-02 | 5.71E-04 | 5.70E-04 | rs8192573 | 1.31E-06 | √ |
| LDL | *AIF1* | 19 | 1.00E+00 | 1.00E+00 | 1.00E+00 | 2.31E-02 | 2.52E-02 | 6.10E-04 | 6.09E-04 | rs3911893 | 1.69E-06 | √ |
| LDL | *AL161915.1* | 5 | 5.88E-01 | 3.35E-02 | 3.74E-02 | 1.00E+00 | 1.00E+00 | 1.00E+00 | 1.00E+00 | rs61738954 | 2.82E-02 | √ |
| LDL | *ALG14* | 17 | 5.42E-06 | 9.21E-60 | 3.87E-60 | 5.79E-03 | 1.36E-02 | 5.63E-42 | 5.63E-42 | rs150211570 | 6.71E-03 | √ |
| LDL | *AMZ2* | 7 | 6.34E-08 | 3.86E-29 | 1.12E-29 | 4.19E-03 | 3.46E-03 | 1.80E-42 | 1.80E-42 | rs79076582 | 5.17E-03 | √ |
| LDL | *APOC1* | 12 | 1.77E-34 | 1.66E-137 | 1.15E-137 | 1.43E-02 | 1.23E-02 | 4.72E-224 | 4.72E-224 | rs28399654 | 7.47E-232 |  |
| LDL | *APOC2* | 12 | 2.16E-34 | 2.26E-120 | 2.82E-121 | 2.13E-02 | 1.55E-02 | 4.54E-224 | 4.53E-224 | rs28399654 | 7.47E-232 |  |
| LDL | *APOE* | 14 | 8.23E-25 | 4.88E-137 | 7.18E-137 | 1.97E-02 | 1.01E-02 | 5.38E-224 | 5.37E-224 | rs28399654 | 7.47E-232 |  |
| LDL | *ARL1* | 2 | 2.45E-01 | 1.03E-02 | 5.08E-03 | 1.00E+00 | 1.00E+00 | 1.00E+00 | 1.00E+00 | rs201873038 | 4.74E-03 | √ |
| LDL | *ATG4D* | 15 | 1.00E+00 | 1.17E-04 | 1.52E-05 | 5.42E-02 | 7.71E-02 | 5.23E-02 | 5.22E-02 | rs45518133 | 6.62E-10 |  |
| LDL | *ATP8B1* | 2 | 1.00E+00 | 7.80E-05 | 2.85E-05 | 3.27E-03 | 1.15E-02 | 1.42E-03 | 1.42E-03 | rs121909100 | 1.33E-02 | √ |
| LDL | *BCL3* | 14 | 1.00E-29 | 9.26E-133 | 5.93E-132 | 5.38E-03 | 2.33E-02 | 5.43E-224 | 5.43E-224 | rs28399654 | 7.47E-232 |  |
| LDL | *BEST1* | 4 | 1.00E+00 | 2.24E-04 | 7.66E-05 | 1.63E-02 | 3.95E-02 | 1.43E-02 | 1.43E-02 | rs3815045 | 1.13E-04 | √ |
| LDL | *BTBD7* | 6 | 1.00E+00 | 1.84E-05 | 3.45E-06 | 1.19E-02 | 3.42E-03 | 1.46E-06 | 1.46E-06 | rs150981613 | 3.39E-03 | √ |
| LDL | *BTN3A3* | 18 | 1.00E+00 | 1.00E+00 | 1.00E+00 | 1.90E-02 | 1.16E-03 | 5.71E-04 | 5.70E-04 | rs1800562 | 2.61E-12 |  |
| LDL | *BTNL2* | 16 | 1.00E+00 | 1.00E+00 | 1.00E+00 | 2.72E-02 | 1.61E-02 | 4.95E-04 | 4.95E-04 | rs8192573 | 1.31E-06 | √ |
| LDL | *C11orf63* | 4 | 1.00E+00 | 2.24E-04 | 7.66E-05 | 4.14E-02 | 3.05E-02 | 1.43E-02 | 1.43E-02 | rs145386058 | 1.29E-02 | √ |
| LDL | *C14orf102* | 6 | 1.00E+00 | 1.84E-05 | 3.45E-06 | 3.14E-02 | 1.56E-02 | 1.46E-06 | 1.46E-06 | rs143888178 | 1.15E-02 | √ |
| LDL | *C14orf93* | 6 | 1.00E+00 | 1.84E-05 | 3.45E-06 | 1.99E-02 | 1.54E-02 | 1.46E-06 | 1.46E-06 | rs138326972 | 7.71E-03 | √ |
| LDL | *C19orf12* | 13 | 5.22E-32 | 3.66E-144 | 4.57E-145 | 2.13E-02 | 5.64E-05 | 4.94E-224 | 4.94E-224 | rs151025990 | 3.85E-02 | √ |
| LDL | *C19orf57* | 15 | 6.00E-20 | 9.77E-109 | 1.22E-109 | 1.88E-02 | 2.20E-02 | 6.30E-224 | 6.30E-224 | rs143203128 | 4.22E-03 | √ |
| LDL | *C1orf74* | 17 | 2.49E-11 | 2.05E-41 | 7.16E-41 | 2.66E-02 | 1.47E-02 | 5.86E-42 | 5.86E-42 | rs144356617 | 7.07E-03 | √ |
| LDL | *C6orf211* | 19 | 1.00E+00 | 1.00E+00 | 1.00E+00 | 2.34E-02 | 1.63E-02 | 6.10E-04 | 6.09E-04 | rs114607043 | 4.13E-03 | √ |
| LDL | *C6orf70* | 19 | 1.00E+00 | 1.00E+00 | 1.00E+00 | 1.54E-02 | 1.02E-02 | 6.10E-04 | 6.09E-04 | rs112432653 | 1.52E-03 | √ |
| LDL | *CCDC97* | 7 | 1.00E+00 | 6.18E-13 | 7.73E-14 | 5.12E-02 | 4.98E-02 | 2.48E-02 | 2.48E-02 | rs201283333 | 1.12E-02 | √ |
| LDL | *CD164* | 19 | 1.00E+00 | 1.00E+00 | 1.00E+00 | 2.88E-02 | 2.04E-02 | 6.10E-04 | 6.09E-04 | rs150654249 | 1.64E-02 | √ |
| LDL | *CDK2* | 2 | 2.45E-01 | 1.03E-02 | 5.08E-03 | 1.00E+00 | 1.00E+00 | 1.00E+00 | 1.00E+00 | rs143838622 | 1.43E-03 | √ |
| LDL | *CDKAL1* | 18 | 1.00E+00 | 1.00E+00 | 1.00E+00 | 6.42E-03 | 1.87E-02 | 5.71E-04 | 5.70E-04 | rs61737148 | 3.87E-02 | √ |
| LDL | *CLSPN* | 5 | 5.88E-01 | 3.35E-02 | 3.74E-02 | 1.00E+00 | 1.00E+00 | 1.00E+00 | 1.00E+00 | rs146817855 | 6.86E-03 | √ |
| LDL | *CNFN* | 15 | 1.00E+00 | 3.18E-04 | 4.41E-05 | 6.81E-02 | 6.41E-02 | 5.21E-02 | 5.21E-02 | rs201596848 | 4.91E-12 |  |
| LDL | *CNKSR1* | 15 | 8.88E-12 | 8.21E-40 | 5.39E-39 | 9.07E-03 | 8.97E-03 | 5.44E-42 | 5.44E-42 | rs34939368 | 2.72E-03 | √ |
| LDL | *COX7A2* | 19 | 1.00E+00 | 1.00E+00 | 1.00E+00 | 4.24E-03 | 1.13E-02 | 6.10E-04 | 6.09E-04 | rs147152005 | 1.10E-02 | √ |
| LDL | *CRIP1* | 6 | 1.00E+00 | 1.84E-05 | 3.45E-06 | 2.32E-02 | 1.10E-03 | 1.46E-06 | 1.46E-06 | rs76619093 | 2.25E-03 | √ |
| LDL | *CRY1* | 2 | 2.45E-01 | 1.03E-02 | 5.08E-03 | 1.00E+00 | 1.00E+00 | 1.00E+00 | 1.00E+00 | rs147063979 | 1.33E-02 | √ |
| LDL | *CTAGE1* | 4 | 1.00E+00 | 2.39E-05 | 4.66E-06 | 2.75E-02 | 1.74E-02 | 2.51E-03 | 2.51E-03 | rs8087963 | 1.21E-01 | √ |
| LDL | *CTC-435M10.3* | 13 | 1.56E-31 | 4.23E-120 | 5.29E-121 | 7.47E-03 | 9.20E-03 | 5.06E-224 | 5.06E-224 | rs143477104 | 4.43E-03 | √ |
| LDL | *CYGB* | 7 | 6.34E-08 | 3.86E-29 | 1.12E-29 | 3.27E-03 | 1.81E-03 | 1.80E-42 | 1.80E-42 | rs139765369 | 5.34E-03 | √ |
| LDL | *CYP4F2* | 14 | 1.54E-28 | 7.02E-129 | 8.77E-130 | 2.25E-02 | 1.06E-02 | 5.59E-224 | 5.59E-224 | rs117610912 | 3.26E-03 | √ |
| LDL | *DCDC2B* | 17 | 5.42E-06 | 9.21E-60 | 3.87E-60 | 2.53E-02 | 4.78E-04 | 5.63E-42 | 5.63E-42 | rs141270394 | 2.34E-03 | √ |
| LDL | *DENND1C* | 16 | 1.00E+00 | 2.00E-02 | 7.44E-03 | 6.12E-02 | 8.15E-02 | 5.71E-02 | 5.71E-02 | rs150449347 | 1.78E-03 | √ |
| LDL | *DENND2C* | 20 | 1.03E-11 | 3.51E-148 | 4.39E-149 | 4.20E-03 | 6.40E-03 | 0.00E+00 | 1.26E-01 | rs115508967 | 9.81E-04 | √ |
| LDL | *DHCR24* | 16 | 4.41E-11 | 3.32E-41 | 2.10E-40 | 2.87E-02 | 2.18E-02 | 5.63E-42 | 5.63E-42 | rs11591147 | 0.00E+00 |  |
| LDL | *DLK2* | 69 | 1.00E+00 | 1.10E-02 | 2.06E-03 | 1.00E+00 | 1.00E+00 | 1.00E+00 | 1.00E+00 | rs145669581 | 8.74E-05 | √ |
| LDL | *DNM2* | 14 | 1.00E+00 | 7.02E-11 | 8.78E-12 | 7.95E-02 | 5.07E-02 | 4.82E-02 | 4.82E-02 | rs139043155 | 1.53E-14 |  |
| LDL | *DYNLT1* | 19 | 1.00E+00 | 1.00E+00 | 1.00E+00 | 4.45E-03 | 4.31E-03 | 6.10E-04 | 6.09E-04 | rs12204826 | 1.16E-03 | √ |
| LDL | *EIF3G* | 16 | 1.00E+00 | 2.00E-02 | 7.44E-03 | 8.43E-02 | 6.10E-02 | 5.71E-02 | 5.71E-02 | rs45518133 | 6.62E-10 |  |
| LDL | *ELP2* | 3 | 1.00E+00 | 8.04E-07 | 2.82E-07 | 1.85E-02 | 6.57E-03 | 1.79E-03 | 1.79E-03 | rs199596507 | 5.72E-03 | √ |
| LDL | *EPB41* | 17 | 2.49E-11 | 2.05E-41 | 7.16E-41 | 1.94E-02 | 2.07E-02 | 5.86E-42 | 5.86E-42 | rs111642750 | 5.89E-03 | √ |
| LDL | *EPS8L1* | 13 | 5.22E-32 | 3.66E-144 | 4.57E-145 | 6.28E-03 | 1.37E-02 | 4.94E-224 | 4.94E-224 | rs61734210 | 7.56E-03 | √ |
| LDL | *FAM129A* | 5 | 5.88E-01 | 3.35E-02 | 3.74E-02 | 1.00E+00 | 1.00E+00 | 1.00E+00 | 1.00E+00 | rs151291324 | 2.18E-02 | √ |
| LDL | *FAM213B* | 5 | 3.63E-02 | 2.10E-02 | 7.98E-02 | 1.00E+00 | 1.00E+00 | 1.00E+00 | 1.00E+00 | rs202216018 | 5.53E-03 | √ |
| LDL | *FANCE* | 19 | 1.00E+00 | 1.00E+00 | 1.00E+00 | 1.83E-02 | 5.57E-03 | 6.10E-04 | 6.09E-04 | rs33959228 | 3.06E-04 | √ |
| LDL | *FBXW9* | 12 | 2.16E-34 | 2.26E-120 | 2.82E-121 | 1.60E-02 | 1.90E-02 | 4.54E-224 | 4.53E-224 | rs45564645 | 1.49E-03 | √ |
| LDL | *FCGBP* | 14 | 8.23E-25 | 4.88E-137 | 7.18E-137 | 7.81E-03 | 9.71E-03 | 5.38E-224 | 5.37E-224 | rs140296932 | 5.38E-04 | √ |
| LDL | *FUCA2* | 19 | 1.00E+00 | 1.00E+00 | 1.00E+00 | 9.64E-03 | 9.71E-04 | 6.10E-04 | 6.09E-04 | rs35263016 | 1.17E-02 | √ |
| LDL | *GABBR1* | 18 | 1.00E+00 | 1.00E+00 | 1.00E+00 | 1.22E-02 | 9.76E-03 | 5.81E-04 | 5.81E-04 | rs36210739 | 4.01E-03 | √ |
| LDL | *GLO1* | 10 | 1.00E+00 | 5.20E-05 | 8.53E-06 | 1.00E+00 | 1.00E+00 | 1.00E+00 | 1.00E+00 | rs61758417 | 4.50E-04 | √ |
| LDL | *GMPR2* | 7 | 1.00E+00 | 4.63E-05 | 6.02E-06 | 1.30E-02 | 1.17E-02 | 1.72E-06 | 1.72E-06 | rs138574046 | 4.62E-03 | √ |
| LDL | *GPANK1* | 19 | 1.00E+00 | 1.00E+00 | 1.00E+00 | 2.93E-02 | 7.66E-03 | 6.10E-04 | 6.09E-04 | rs3911893 | 1.69E-06 | √ |
| LDL | *HECTD3* | 5 | 5.88E-01 | 3.35E-02 | 3.74E-02 | 1.00E+00 | 1.00E+00 | 1.00E+00 | 1.00E+00 | rs41306591 | 5.16E-05 | √ |
| LDL | *HES5* | 16 | 4.41E-11 | 3.32E-41 | 2.10E-40 | 8.45E-03 | 2.67E-03 | 5.63E-42 | 5.63E-42 | rs202216018 | 5.53E-03 | √ |
| LDL | *HIST1H1B* | 19 | 1.00E+00 | 1.00E+00 | 1.00E+00 | 1.54E-02 | 2.11E-02 | 6.10E-04 | 6.09E-04 | rs201148465 | 2.49E-08 |  |
| LDL | *HIST1H2BH* | 15 | 1.00E+00 | 1.00E+00 | 1.00E+00 | 3.16E-02 | 5.81E-03 | 4.63E-04 | 4.63E-04 | rs1800562 | 2.61E-12 |  |
| LDL | *HIST1H2BK* | 79 | 1.00E+00 | 5.00E-02 | 1.86E-02 | 1.00E+00 | 1.00E+00 | 1.00E+00 | 1.00E+00 | rs114674760 | 3.70E-04 | √ |
| LDL | *HIST1H3H* | 19 | 1.00E+00 | 1.00E+00 | 1.00E+00 | 2.44E-02 | 5.84E-03 | 6.10E-04 | 6.09E-04 | rs201148465 | 2.49E-08 |  |
| LDL | *HIST1H4H* | 19 | 1.00E+00 | 1.00E+00 | 1.00E+00 | 2.33E-02 | 9.31E-03 | 6.10E-04 | 6.09E-04 | rs1800562 | 2.61E-12 |  |
| LDL | *HLA-E* | 10 | 1.00E+00 | 5.20E-05 | 8.53E-06 | 1.00E+00 | 1.00E+00 | 1.00E+00 | 1.00E+00 | rs150846286 | 3.55E-04 | √ |
| LDL | *HPCA* | 20 | 6.75E-13 | 1.11E-150 | 1.39E-151 | 2.90E-02 | 2.27E-02 | 0.00E+00 | 1.26E-01 | rs200910193 | 1.51E-02 | √ |
| LDL | *HSD17B8* | 19 | 1.00E+00 | 1.00E+00 | 1.00E+00 | 6.93E-03 | 2.36E-02 | 6.10E-04 | 6.09E-04 | rs3198005 | 2.08E-03 | √ |
| LDL | *HSP90AB1* | 77 | 1.00E+00 | 1.14E-01 | 4.76E-02 | 1.00E+00 | 1.00E+00 | 1.00E+00 | 1.00E+00 | rs138499828 | 7.37E-03 | √ |
| LDL | *IFT81* | 2 | 2.45E-01 | 1.03E-02 | 5.08E-03 | 1.00E+00 | 1.00E+00 | 1.00E+00 | 1.00E+00 | rs146368046 | 1.93E-02 | √ |
| LDL | *IMP4* | 7 | 1.00E+00 | 3.35E-43 | 4.18E-44 | 1.08E-03 | 1.03E-02 | 2.51E-69 | 2.51E-69 | rs74409947 | 6.81E-03 | √ |
| LDL | *IPCEF1* | 19 | 1.00E+00 | 1.00E+00 | 1.00E+00 | 8.17E-03 | 1.32E-02 | 6.10E-04 | 6.09E-04 | rs79910351 | 1.07E-02 | √ |
| LDL | *ITGB1BP1* | 7 | 1.00E+00 | 3.35E-43 | 4.18E-44 | 4.44E-03 | 1.60E-02 | 2.51E-69 | 2.51E-69 | rs201291876 | 3.12E-02 | √ |
| LDL | *ITPA* | 5 | 1.00E+00 | 1.00E+00 | 1.00E+00 | 8.96E-03 | 2.06E-03 | 3.96E-05 | 3.95E-05 | rs2422857 | 1.75E-02 | √ |
| LDL | *JTB* | 19 | 1.13E-10 | 4.10E-40 | 3.06E-40 | 2.88E-02 | 1.76E-02 | 6.51E-42 | 6.51E-42 | rs144796295 | 1.25E-03 | √ |
| LDL | *KCNC3* | 16 | 1.00E+00 | 2.00E-02 | 7.44E-03 | 8.46E-02 | 6.48E-02 | 5.71E-02 | 5.71E-02 | rs146344351 | 8.08E-52 |  |
| LDL | *KDM1B* | 19 | 1.00E+00 | 1.00E+00 | 1.00E+00 | 2.50E-02 | 1.56E-02 | 6.10E-04 | 6.09E-04 | rs1142345 | 6.30E-03 | √ |
| LDL | *KIAA0090* | 15 | 7.85E-09 | 4.73E-40 | 5.34E-40 | 1.89E-02 | 1.07E-02 | 5.40E-42 | 5.39E-42 | rs143351261 | 7.53E-03 | √ |
| LDL | *KLHDC9* | 19 | 1.44E-09 | 1.50E-42 | 8.24E-43 | 2.09E-02 | 9.51E-03 | 6.45E-42 | 6.44E-42 | rs139629845 | 1.95E-03 | √ |
| LDL | *KLHL21* | 21 | 1.13E-07 | 2.48E-42 | 3.69E-41 | 1.18E-02 | 2.25E-02 | 7.22E-42 | 7.21E-42 | rs200409038 | 6.72E-04 | √ |
| LDL | *KLHL32* | 19 | 1.00E+00 | 1.00E+00 | 1.00E+00 | 9.20E-03 | 1.32E-03 | 6.10E-04 | 6.09E-04 | rs145638531 | 8.03E-04 | √ |
| LDL | *KRI1* | 15 | 1.00E+00 | 1.17E-04 | 1.52E-05 | 5.98E-02 | 6.36E-02 | 5.23E-02 | 5.22E-02 | rs45518133 | 6.62E-10 |  |
| LDL | *LDLR* | 9 | 1.00E+00 | 2.26E-09 | 2.82E-10 | 5.34E-02 | 5.43E-02 | 3.39E-02 | 3.39E-02 | rs139043155 | 1.53E-14 |  |
| LDL | *LENG1* | 12 | 6.88E-36 | 1.17E-143 | 1.46E-144 | 1.57E-02 | 2.46E-02 | 4.58E-224 | 4.58E-224 | rs139593424 | 7.60E-03 | √ |
| LDL | *LIPE* | 13 | 5.22E-32 | 3.66E-144 | 4.57E-145 | 6.05E-03 | 4.04E-05 | 4.94E-224 | 4.94E-224 | rs201596848 | 4.91E-12 |  |
| LDL | *MAP3K10* | 16 | 1.00E+00 | 2.00E-02 | 7.44E-03 | 7.40E-02 | 6.32E-02 | 5.71E-02 | 5.71E-02 | rs140296932 | 5.38E-04 | √ |
| LDL | *MAP3K2* | 7 | 1.00E+00 | 3.35E-43 | 4.18E-44 | 2.13E-02 | 1.36E-02 | 2.51E-69 | 2.51E-69 | rs61743282 | 5.78E-03 | √ |
| LDL | *MAU2* | 12 | 2.16E-34 | 2.26E-120 | 2.82E-121 | 2.45E-02 | 1.86E-02 | 4.54E-224 | 4.53E-224 | rs138995642 | 2.35E-03 | √ |
| LDL | *MDN1* | 18 | 1.00E+00 | 1.00E+00 | 1.00E+00 | 9.66E-03 | 2.45E-03 | 5.71E-04 | 5.70E-04 | rs190202337 | 8.66E-03 | √ |
| LDL | *MED8* | 14 | 8.14E-12 | 9.56E-40 | 4.43E-39 | 2.91E-02 | 4.92E-03 | 5.13E-42 | 5.13E-42 | rs144275350 | 1.76E-03 | √ |
| LDL | *MEF2BNB* | 13 | 1.00E+00 | 2.76E-07 | 3.64E-08 | 5.46E-02 | 5.95E-02 | 4.38E-02 | 4.37E-02 | rs138995642 | 2.35E-03 | √ |
| LDL | *MGST3* | 19 | 2.61E-10 | 4.46E-42 | 4.01E-42 | 2.75E-02 | 7.62E-03 | 6.46E-42 | 6.45E-42 | rs202095448 | 3.51E-02 | √ |
| LDL | *MIXL1* | 17 | 2.49E-11 | 2.05E-41 | 7.16E-41 | 4.15E-05 | 6.38E-03 | 5.86E-42 | 5.86E-42 | rs75188792 | 1.97E-02 | √ |
| LDL | *MLLT4-AS1* | 107 | 1.00E+00 | 8.78E-02 | 4.41E-02 | 1.00E+00 | 1.00E+00 | 1.00E+00 | 1.00E+00 | rs138998311 | 2.33E-12 |  |
| LDL | *MRPL11* | 4 | 1.00E+00 | 2.24E-04 | 7.66E-05 | 1.88E-02 | 1.69E-02 | 1.43E-02 | 1.43E-02 | noRS | 8.60E-04 | √ |
| LDL | *MRPL4* | 15 | 1.00E+00 | 3.18E-04 | 4.41E-05 | 6.09E-02 | 5.96E-02 | 5.21E-02 | 5.21E-02 | rs45518133 | 6.62E-10 |  |
| LDL | *MTO1* | 18 | 1.00E+00 | 1.00E+00 | 1.00E+00 | 2.54E-02 | 1.71E-02 | 5.71E-04 | 5.70E-04 | rs145043138 | 2.36E-02 | √ |
| LDL | *NBPF11* | 19 | 1.44E-09 | 1.50E-42 | 8.24E-43 | 2.87E-02 | 1.64E-02 | 6.45E-42 | 6.44E-42 | rs114731263 | 1.09E-02 | √ |
| LDL | *NCOA6* | 5 | 1.00E+00 | 1.00E+00 | 1.00E+00 | 2.46E-02 | 1.70E-02 | 3.75E-05 | 3.75E-05 | rs138765880 | 2.92E-03 | √ |
| LDL | *NDUFA11* | 8 | 1.00E+00 | 3.41E-13 | 4.26E-14 | 3.71E-02 | 4.63E-02 | 2.73E-02 | 2.73E-02 | rs113345491 | 6.67E-03 | √ |
| LDL | *NDUFS2* | 19 | 2.65E-14 | 3.40E-189 | 4.25E-190 | 2.30E-02 | 7.42E-04 | 0.00E+00 | 1.26E-01 | rs139629845 | 1.95E-03 | √ |
| LDL | *NFKBIE* | 14 | 1.00E+00 | 5.09E-04 | 7.10E-05 | 1.00E+00 | 1.00E+00 | 1.00E+00 | 1.00E+00 | rs138499828 | 7.37E-03 | √ |
| LDL | *NPAS1* | 15 | 1.00E+00 | 3.18E-04 | 4.41E-05 | 7.73E-02 | 5.24E-02 | 5.21E-02 | 5.21E-02 | rs201572880 | 5.09E-04 | √ |
| LDL | *NPEPL1* | 6 | 1.00E+00 | 1.00E+00 | 1.00E+00 | 5.75E-03 | 4.53E-03 | 4.24E-05 | 4.24E-05 | rs201383880 | 1.91E-02 | √ |
| LDL | *NT5E* | 18 | 1.00E+00 | 1.00E+00 | 1.00E+00 | 2.64E-02 | 4.88E-03 | 5.71E-04 | 5.70E-04 | rs200250022 | 2.65E-02 | √ |
| LDL | *NUMBL* | 17 | 1.84E-13 | 2.69E-88 | 3.37E-89 | 5.97E-03 | 2.37E-02 | 7.24E-224 | 7.24E-224 | rs200888669 | 2.54E-03 | √ |
| LDL | *OAZ1* | 16 | 1.00E+00 | 2.00E-02 | 7.44E-03 | 7.29E-02 | 6.13E-02 | 5.71E-02 | 5.71E-02 | rs201250275 | 4.81E-04 | √ |
| LDL | *OCEL1* | 15 | 1.00E+00 | 1.17E-04 | 1.52E-05 | 7.97E-02 | 7.74E-02 | 5.23E-02 | 5.22E-02 | rs199848268 | 1.24E-03 | √ |
| LDL | *PEA15* | 17 | 2.49E-11 | 2.05E-41 | 7.16E-41 | 2.69E-02 | 1.34E-02 | 5.86E-42 | 5.86E-42 | rs199842829 | 6.21E-04 | √ |
| LDL | *PFDN2* | 18 | 3.83E-14 | 4.21E-163 | 5.26E-164 | 5.19E-03 | 2.68E-03 | 0.00E+00 | 1.26E-01 | rs139629845 | 1.95E-03 | √ |
| LDL | *PLTP* | 7 | 1.00E+00 | 3.86E-02 | 2.50E-02 | 3.03E-02 | 3.76E-03 | 3.90E-05 | 3.89E-05 | rs6065903 | 4.25E-03 | √ |
| LDL | *PPAN* | 16 | 1.00E+00 | 2.00E-02 | 7.44E-03 | 6.41E-02 | 7.26E-02 | 5.71E-02 | 5.71E-02 | rs45518133 | 6.62E-10 |  |
| LDL | *PPP6R1* | 12 | 1.77E-34 | 1.66E-137 | 1.15E-137 | 2.51E-02 | 2.32E-02 | 4.72E-224 | 4.72E-224 | rs61734210 | 7.56E-03 | √ |
| LDL | *PRCC* | 17 | 2.49E-11 | 2.05E-41 | 7.16E-41 | 3.46E-03 | 1.34E-02 | 5.86E-42 | 5.86E-42 | rs149267462 | 6.96E-03 | √ |
| LDL | *PRKCH* | 5 | 1.00E+00 | 6.51E-06 | 8.65E-07 | 2.87E-02 | 1.80E-02 | 1.22E-06 | 1.22E-06 | rs144630332 | 1.08E-02 | √ |
| LDL | *PSENEN* | 14 | 1.00E-29 | 9.26E-133 | 5.93E-132 | 3.84E-03 | 1.04E-02 | 5.43E-224 | 5.43E-224 | rs61747965 | 4.39E-04 | √ |
| LDL | *PSMB1* | 19 | 1.00E+00 | 1.00E+00 | 1.00E+00 | 5.68E-03 | 1.05E-02 | 6.10E-04 | 6.09E-04 | rs112432653 | 1.52E-03 | √ |
| LDL | *PTPN1* | 5 | 1.00E+00 | 1.00E+00 | 1.00E+00 | 7.39E-03 | 1.14E-02 | 3.96E-05 | 3.95E-05 | rs202129855 | 1.54E-03 | √ |
| LDL | *RAB39A* | 4 | 1.00E+00 | 2.24E-04 | 7.66E-05 | 3.25E-02 | 2.59E-02 | 1.43E-02 | 1.43E-02 | rs189775078 | 2.85E-03 | √ |
| LDL | *RAB8A* | 15 | 1.00E+00 | 3.18E-04 | 4.41E-05 | 7.90E-02 | 5.72E-02 | 5.21E-02 | 5.21E-02 | rs117610912 | 3.26E-03 | √ |
| LDL | *RASSF2* | 6 | 4.55E-03 | 1.22E-02 | 1.00E+00 | 1.00E+00 | 1.00E+00 | 1.00E+00 | 1.00E+00 | rs141901582 | 3.58E-02 | √ |
| LDL | *RBMXL2* | 4 | 1.00E+00 | 2.24E-04 | 7.66E-05 | 2.43E-02 | 1.97E-02 | 1.43E-02 | 1.43E-02 | rs115534913 | 4.47E-04 | √ |
| LDL | *RIT1* | 20 | 1.03E-11 | 3.51E-148 | 4.39E-149 | 1.34E-02 | 2.34E-02 | 0.00E+00 | 1.26E-01 | rs145545651 | 8.19E-04 | √ |
| LDL | *ROCK1* | 3 | 1.00E+00 | 8.04E-07 | 2.82E-07 | 2.68E-02 | 5.54E-03 | 1.79E-03 | 1.79E-03 | rs185578147 | 4.67E-03 | √ |
| LDL | *ROMO1* | 5 | 1.00E+00 | 1.00E+00 | 1.00E+00 | 2.75E-02 | 2.19E-02 | 3.96E-05 | 3.95E-05 | rs138765880 | 2.92E-03 | √ |
| LDL | *RP1-153P14.5* | 19 | 1.00E+00 | 1.00E+00 | 1.00E+00 | 2.35E-03 | 7.48E-03 | 6.10E-04 | 6.09E-04 | rs141593198 | 3.35E-02 | √ |
| LDL | *RP11-131H24.4* | 6 | 1.00E+00 | 1.84E-05 | 3.45E-06 | 2.39E-02 | 3.86E-03 | 1.46E-06 | 1.46E-06 | rs28929474 | 4.30E-14 |  |
| LDL | *RP11-176H8.1* | 6 | 1.00E+00 | 1.84E-05 | 3.45E-06 | 1.06E-02 | 6.03E-03 | 1.46E-06 | 1.46E-06 | rs201915249 | 2.42E-04 | √ |
| LDL | *RP11-206L10.7* | 19 | 3.19E-11 | 3.22E-148 | 4.02E-149 | 7.88E-03 | 2.15E-02 | 0.00E+00 | 1.26E-01 | rs145442390 | 8.08E-03 | √ |
| LDL | *RP11-276E17.2* | 14 | 8.14E-12 | 9.56E-40 | 4.43E-39 | 2.83E-02 | 4.91E-03 | 5.13E-42 | 5.13E-42 | rs138713518 | 6.89E-02 | √ |
| LDL | *RP11-469N6.1* | 4 | 1.00E+00 | 2.24E-04 | 7.66E-05 | 2.04E-02 | 2.10E-02 | 1.43E-02 | 1.43E-02 | rs148540880 | 6.52E-04 | √ |
| LDL | *RP11-536C5.7* | 17 | 2.49E-11 | 2.05E-41 | 7.16E-41 | 1.36E-02 | 2.07E-02 | 5.86E-42 | 5.86E-42 | rs199842829 | 6.21E-04 | √ |
| LDL | *RP5-1073O3.7* | 20 | 1.03E-11 | 3.51E-148 | 4.39E-149 | 1.20E-02 | 1.81E-02 | 0.00E+00 | 1.26E-01 | rs115508967 | 9.81E-04 | √ |
| LDL | *RPLP2* | 4 | 1.00E+00 | 2.24E-04 | 7.66E-05 | 2.16E-02 | 1.91E-02 | 1.43E-02 | 1.43E-02 | rs192744525 | 2.64E-05 | √ |
| LDL | *RTN4IP1* | 19 | 1.00E+00 | 1.00E+00 | 1.00E+00 | 1.99E-02 | 1.41E-02 | 6.10E-04 | 6.09E-04 | rs61741860 | 2.22E-02 | √ |
| LDL | *SCAF11* | 2 | 2.45E-01 | 1.03E-02 | 5.08E-03 | 1.00E+00 | 1.00E+00 | 1.00E+00 | 1.00E+00 | rs140285438 | 4.18E-03 | √ |
| LDL | *SEC63* | 12 | 2.64E-01 | 3.97E-03 | 2.52E-03 | 1.00E+00 | 1.00E+00 | 1.00E+00 | 1.00E+00 | rs201176284 | 2.94E-02 | √ |
| LDL | *SLC35C1* | 4 | 1.00E+00 | 2.24E-04 | 7.66E-05 | 3.14E-02 | 2.58E-02 | 1.43E-02 | 1.43E-02 | rs141577566 | 1.05E-02 | √ |
| LDL | *SNRNP48* | 19 | 1.00E+00 | 1.00E+00 | 1.00E+00 | 2.24E-03 | 1.76E-02 | 6.10E-04 | 6.09E-04 | rs149129914 | 1.21E-03 | √ |
| LDL | *SNRPB2* | 5 | 1.00E+00 | 2.48E-01 | 1.72E-01 | 1.53E-02 | 6.80E-03 | 3.87E-05 | 3.86E-05 | rs147795685 | 4.34E-04 | √ |
| LDL | *SNX9* | 11 | 1.68E-01 | 3.40E-03 | 2.17E-03 | 1.00E+00 | 1.00E+00 | 1.00E+00 | 1.00E+00 | rs61756209 | 3.65E-02 | √ |
| LDL | *SPAG5* | 7 | 6.34E-08 | 3.86E-29 | 1.12E-29 | 2.10E-03 | 1.78E-02 | 1.80E-42 | 1.80E-42 | rs61732688 | 1.28E-03 | √ |
| LDL | *SRGAP2* | 19 | 4.78E-09 | 6.08E-41 | 2.94E-41 | 6.75E-03 | 1.45E-02 | 6.64E-42 | 6.64E-42 | rs141643308 | 1.43E-02 | √ |
| LDL | *SRSF12* | 19 | 1.00E+00 | 1.00E+00 | 1.00E+00 | 3.70E-03 | 1.86E-02 | 6.10E-04 | 6.09E-04 | rs190202337 | 8.66E-03 | √ |
| LDL | *SSR2* | 19 | 1.44E-09 | 1.50E-42 | 8.24E-43 | 2.12E-02 | 1.69E-02 | 6.45E-42 | 6.44E-42 | rs111741196 | 1.16E-03 | √ |
| LDL | *ST6GALNAC2* | 3 | 3.78E-05 | 2.24E-09 | 2.19E-09 | 4.12E-03 | 2.36E-02 | 4.23E-30 | 4.23E-30 | rs139765369 | 5.34E-03 | √ |
| LDL | *STARD3* | 5 | 2.18E-07 | 1.27E-27 | 1.58E-28 | 3.10E-02 | 9.30E-03 | 1.26E-42 | 1.26E-42 | rs150365108 | 2.43E-03 | √ |
| LDL | *STIM1* | 4 | 1.00E+00 | 2.24E-04 | 7.66E-05 | 2.80E-02 | 3.65E-02 | 1.43E-02 | 1.43E-02 | rs143555602 | 3.38E-02 | √ |
| LDL | *TBCB* | 20 | 1.71E-10 | 3.79E-132 | 6.71E-132 | 1.67E-02 | 2.41E-02 | 8.56E-224 | 8.56E-224 | rs61747965 | 4.39E-04 | √ |
| LDL | *TCFL5* | 7 | 1.00E+00 | 3.86E-02 | 2.50E-02 | 7.88E-04 | 2.32E-03 | 3.90E-05 | 3.89E-05 | rs139953956 | 5.97E-05 | √ |
| LDL | *TEX2* | 7 | 5.53E-09 | 4.72E-28 | 5.90E-29 | 1.29E-02 | 2.43E-02 | 1.69E-42 | 1.69E-42 | rs201148948 | 1.33E-03 | √ |
| LDL | *TMC6* | 4 | 3.65E-05 | 1.79E-09 | 1.52E-09 | 2.16E-02 | 2.37E-02 | 5.91E-30 | 5.91E-30 | rs111563325 | 2.19E-03 | √ |
| LDL | *TMEM125* | 17 | 2.49E-11 | 2.05E-41 | 7.16E-41 | 2.58E-02 | 2.08E-02 | 5.86E-42 | 5.86E-42 | rs144275350 | 1.76E-03 | √ |
| LDL | *TMEM147* | 8 | 1.00E+00 | 3.41E-13 | 4.26E-14 | 4.80E-02 | 4.65E-02 | 2.73E-02 | 2.73E-02 | rs61747965 | 4.39E-04 | √ |
| LDL | *TMEM170B* | 19 | 1.00E+00 | 1.00E+00 | 1.00E+00 | 8.14E-03 | 2.46E-02 | 6.10E-04 | 6.09E-04 | rs41272545 | 5.61E-03 | √ |
| LDL | *TMEM50A* | 5 | 5.88E-01 | 3.35E-02 | 3.74E-02 | 1.00E+00 | 1.00E+00 | 1.00E+00 | 1.00E+00 | rs34484514 | 4.35E-04 | √ |
| LDL | *TNXB* | 19 | 1.00E+00 | 1.00E+00 | 1.00E+00 | 8.77E-04 | 1.14E-02 | 6.10E-04 | 6.09E-04 | rs8192573 | 1.31E-06 | √ |
| LDL | *TP53I3* | 7 | 1.00E+00 | 3.35E-43 | 4.18E-44 | 3.99E-03 | 1.38E-02 | 2.51E-69 | 2.51E-69 | rs137910641 | 4.14E-03 | √ |
| LDL | *TRAF6* | 4 | 1.00E+00 | 2.24E-04 | 7.66E-05 | 3.68E-02 | 1.73E-02 | 1.43E-02 | 1.43E-02 | rs148380512 | 4.66E-03 | √ |
| LDL | *TRAPPC2P1* | 14 | 1.00E+00 | 6.59E-06 | 8.23E-07 | 6.17E-02 | 5.86E-02 | 4.82E-02 | 4.81E-02 | noRS | 6.23E-03 | √ |
| LDL | *TRIM38* | 79 | 1.00E+00 | 5.00E-02 | 1.86E-02 | 1.00E+00 | 1.00E+00 | 1.00E+00 | 1.00E+00 | rs1800562 | 2.61E-12 |  |
| LDL | *TSSK4* | 6 | 1.00E+00 | 1.84E-05 | 3.45E-06 | 2.23E-02 | 9.86E-03 | 1.46E-06 | 1.46E-06 | rs138574046 | 4.62E-03 | √ |
| LDL | *TULP2* | 15 | 1.00E+00 | 3.18E-04 | 4.41E-05 | 6.05E-02 | 7.20E-02 | 5.21E-02 | 5.21E-02 | rs201149176 | 2.44E-03 | √ |
| LDL | *U2AF1L4* | 15 | 1.56E-19 | 6.59E-91 | 3.84E-91 | 1.18E-02 | 2.02E-02 | 5.92E-224 | 5.91E-224 | rs61747965 | 4.39E-04 | √ |
| LDL | *UBE2J1* | 11 | 1.00E+00 | 1.20E-01 | 4.03E-02 | 2.82E-02 | 2.51E-02 | 4.08E-04 | 4.07E-04 | rs190202337 | 8.66E-03 | √ |
| LDL | *UHRF1BP1* | 19 | 1.00E+00 | 1.00E+00 | 1.00E+00 | 2.13E-02 | 2.13E-02 | 6.10E-04 | 6.09E-04 | rs33959228 | 3.06E-04 | √ |
| LDL | *VNN2* | 19 | 1.00E+00 | 1.00E+00 | 1.00E+00 | 3.94E-03 | 1.38E-02 | 6.10E-04 | 6.09E-04 | rs146517500 | 5.56E-03 | √ |
| LDL | *WDR77* | 16 | 1.92E-06 | 6.24E-61 | 1.59E-61 | 3.63E-03 | 4.93E-03 | 5.25E-42 | 5.25E-42 | rs202224198 | 5.23E-03 | √ |
| LDL | *WTIP* | 12 | 1.77E-34 | 1.66E-137 | 1.15E-137 | 2.13E-02 | 9.53E-04 | 4.72E-224 | 4.72E-224 | rs79233678 | 2.24E-02 | √ |
| LDL | *XAB2* | 16 | 1.00E+00 | 4.56E-06 | 5.71E-07 | 6.96E-02 | 7.35E-02 | 5.67E-02 | 5.67E-02 | rs141168886 | 2.83E-03 | √ |
| LDL | *XXbac-BPG181M17.5* | 19 | 1.00E+00 | 1.00E+00 | 1.00E+00 | 2.46E-02 | 3.95E-03 | 6.10E-04 | 6.09E-04 | rs115817940 | 4.87E-04 | √ |
| LDL | *YPEL5* | 4 | 1.00E+00 | 1.36E-04 | 3.74E-05 | 2.34E-02 | 4.61E-03 | 1.10E-06 | 1.10E-06 | rs149145987 | 5.17E-02 | √ |
| LDL | *ZBTB32* | 7 | 1.00E+00 | 6.18E-13 | 7.73E-14 | 4.55E-02 | 2.80E-02 | 2.48E-02 | 2.48E-02 | rs61747965 | 4.39E-04 | √ |
| LDL | *ZBTB48* | 19 | 1.13E-10 | 4.10E-40 | 3.06E-40 | 8.44E-03 | 2.34E-02 | 6.51E-42 | 6.51E-42 | rs200409038 | 6.72E-04 | √ |
| LDL | *ZCCHC11* | 18 | 2.49E-16 | 1.10E-154 | 1.38E-155 | 5.95E-03 | 1.19E-02 | 0.00E+00 | 1.26E-01 | rs141460821 | 4.28E-03 | √ |
| LDL | *ZDHHC14* | 19 | 1.00E+00 | 1.00E+00 | 1.00E+00 | 8.47E-03 | 1.61E-02 | 6.10E-04 | 6.09E-04 | rs61756209 | 3.65E-02 | √ |
| LDL | *ZFP36* | 15 | 1.00E+00 | 3.18E-04 | 4.41E-05 | 8.05E-02 | 7.32E-02 | 5.21E-02 | 5.21E-02 | rs143818753 | 2.72E-03 | √ |
| LDL | *ZNF134* | 18 | 6.86E-13 | 4.96E-132 | 3.95E-132 | 1.41E-02 | 1.43E-02 | 7.56E-224 | 7.55E-224 | noRS | 6.23E-03 | √ |
| LDL | *ZNF343* | 6 | 1.00E+00 | 1.00E+00 | 1.00E+00 | 3.02E-02 | 2.00E-02 | 4.24E-05 | 4.24E-05 | rs141680917 | 6.81E-03 | √ |
| LDL | *ZNF410* | 4 | 1.41E-01 | 1.38E-05 | 2.91E-06 | 1.33E-02 | 6.67E-03 | 1.07E-06 | 1.07E-06 | rs61752569 | 9.45E-04 | √ |
| LDL | *ZNF444* | 15 | 1.00E+00 | 3.18E-04 | 4.41E-05 | 6.92E-02 | 7.10E-02 | 5.21E-02 | 5.21E-02 | rs146186875 | 4.06E-03 | √ |
| LDL | *ZNF512B* | 6 | 1.00E+00 | 1.00E+00 | 1.00E+00 | 1.97E-02 | 2.06E-02 | 4.84E-05 | 4.83E-05 | rs141423196 | 1.25E-04 | √ |
| LDL | *ZNF544* | 14 | 8.23E-25 | 4.88E-137 | 7.18E-137 | 2.75E-02 | 1.99E-02 | 5.38E-224 | 5.37E-224 | rs145443903 | 3.39E-03 | √ |
| LDL | *ZNF551* | 16 | 1.00E+00 | 3.47E-60 | 4.34E-61 | 2.33E-02 | 1.48E-02 | 2.89E-223 | 2.89E-223 | noRS | 6.23E-03 | √ |
| LDL | *ZNF573* | 15 | 1.00E+00 | 3.18E-04 | 4.41E-05 | 5.93E-02 | 7.64E-02 | 5.21E-02 | 5.21E-02 | rs144294637 | 2.57E-03 | √ |
| LDL | *ZNF611* | 15 | 1.00E+00 | 3.18E-04 | 4.41E-05 | 8.27E-02 | 5.48E-02 | 5.21E-02 | 5.21E-02 | rs143289804 | 2.06E-03 | √ |
| LDL | *ZNF682* | 15 | 1.00E+00 | 3.18E-04 | 4.41E-05 | 5.76E-02 | 6.04E-02 | 5.21E-02 | 5.21E-02 | rs10411195 | 1.68E-05 | √ |
| LDL | *ZNF77* | 7 | 1.00E+00 | 6.18E-13 | 7.73E-14 | 4.31E-02 | 4.02E-02 | 2.48E-02 | 2.48E-02 | rs201250275 | 4.81E-04 | √ |
| LDL | *ZNF772* | 15 | 1.00E+00 | 3.18E-04 | 4.41E-05 | 6.26E-02 | 6.04E-02 | 5.21E-02 | 5.21E-02 | noRS | 6.23E-03 | √ |
| LDL | *ZNF812* | 16 | 1.00E+00 | 5.42E-04 | 7.08E-05 | 6.54E-02 | 5.63E-02 | 5.63E-02 | 5.62E-02 | rs34658893 | 5.73E-07 | √ |
| LDL | *ZSCAN18* | 14 | 1.00E+00 | 6.59E-06 | 8.23E-07 | 7.62E-02 | 6.03E-02 | 4.82E-02 | 4.81E-02 | rs145443903 | 3.39E-03 | √ |
| TC | *ABCA9* | 7 | 3.18E-02 | 8.26E-16 | 1.03E-16 | 1.63E-03 | 1.92E-02 | 3.17E-24 | 3.17E-24 | rs77542162 | 8.49E-32 |  |
| TC | *AC133485.1* | 13 | 1.00E+00 | 1.00E+00 | 6.18E-01 | 2.62E-02 | 1.00E-03 | 3.12E-11 | 3.12E-11 | rs79859029 | 4.89E-02 | √ |
| TC | *ADAR* | 19 | 5.43E-07 | 2.09E-33 | 1.60E-33 | 6.19E-03 | 1.35E-02 | 9.59E-31 | 9.59E-31 | rs201905030 | 2.78E-03 | √ |
| TC | *ADCK4* | 15 | 1.00E+00 | 7.07E-03 | 1.28E-03 | 1.00E+00 | 1.00E+00 | 1.00E+00 | 1.00E+00 | rs55793080 | 1.85E-03 | √ |
| TC | *ADRM1* | 5 | 1.00E+00 | 6.23E-07 | 7.79E-08 | 1.06E-02 | 6.98E-03 | 3.55E-29 | 3.55E-29 | rs148515817 | 1.07E-03 | √ |
| TC | *AL035696.1* | 102 | 3.98E-05 | 6.93E-07 | 3.14E-07 | 1.01E-02 | 4.70E-03 | 2.04E-03 | 2.04E-03 | rs202216214 | 9.73E-03 | √ |
| TC | *AL161915.1* | 5 | 1.13E-02 | 2.30E-03 | 6.70E-03 | 1.00E+00 | 1.00E+00 | 1.00E+00 | 1.00E+00 | rs2304305 | 1.77E-02 | √ |
| TC | *ALG14* | 17 | 3.80E-04 | 5.78E-48 | 1.57E-48 | 2.57E-02 | 1.15E-02 | 8.30E-31 | 8.30E-31 | rs34364382 | 5.88E-03 | √ |
| TC | *AMZ2* | 7 | 3.18E-02 | 8.26E-16 | 1.03E-16 | 2.52E-02 | 2.14E-02 | 3.17E-24 | 3.17E-24 | rs79076582 | 3.12E-03 | √ |
| TC | *ANKS1A* | 97 | 4.10E-03 | 2.12E-05 | 1.38E-05 | 2.15E-02 | 1.43E-02 | 3.39E-03 | 3.39E-03 | rs33959228 | 1.16E-06 | √ |
| TC | *ANKS3* | 7 | 1.00E+00 | 1.64E-01 | 8.74E-02 | 2.07E-02 | 1.59E-02 | 1.71E-11 | 1.71E-11 | rs147502184 | 4.60E-03 | √ |
| TC | *APOC1* | 12 | 1.70E-13 | 5.10E-43 | 8.50E-44 | 6.72E-03 | 4.08E-03 | 2.20E-96 | 2.20E-96 | rs28399653 | 2.55E-104 |  |
| TC | *APOC2* | 12 | 2.34E-16 | 7.98E-59 | 1.37E-58 | 8.38E-03 | 2.42E-02 | 2.11E-96 | 2.11E-96 | rs28399653 | 2.55E-104 |  |
| TC | *APOE* | 14 | 1.28E-09 | 2.62E-57 | 1.42E-57 | 9.06E-04 | 1.69E-02 | 2.50E-96 | 2.50E-96 | rs28399653 | 2.55E-104 |  |
| TC | *ASCC3* | 76 | 1.00E+00 | 6.86E-01 | 3.40E-01 | 2.39E-02 | 2.88E-02 | 4.10E-03 | 4.10E-03 | rs144432672 | 4.86E-02 | √ |
| TC | *ATG4D* | 15 | 1.00E+00 | 1.10E-02 | 2.59E-03 | 1.00E+00 | 1.00E+00 | 1.00E+00 | 1.00E+00 | rs200547722 | 4.43E-12 |  |
| TC | *ATP8B1* | 2 | 4.98E-17 | 2.74E-40 | 3.83E-41 | 1.03E-02 | 1.44E-02 | 2.40E-44 | 2.40E-44 | rs146269992 | 1.07E-02 | √ |
| TC | *B3GALT4* | 101 | 6.73E-05 | 1.38E-06 | 6.39E-07 | 7.15E-03 | 9.94E-03 | 2.02E-03 | 2.02E-03 | rs1057149 | 6.67E-07 | √ |
| TC | *BCL3* | 14 | 7.53E-11 | 3.10E-48 | 3.87E-49 | 1.48E-02 | 1.42E-02 | 2.53E-96 | 2.53E-96 | rs28399653 | 2.55E-104 |  |
| TC | *BTBD7* | 6 | 1.00E+00 | 2.94E-05 | 4.58E-06 | 4.37E-03 | 2.41E-02 | 1.87E-06 | 1.87E-06 | rs139526735 | 1.86E-02 | √ |
| TC | *BTBD9* | 82 | 1.51E-03 | 3.19E-05 | 3.56E-05 | 2.24E-02 | 5.52E-03 | 2.83E-03 | 2.83E-03 | rs61758417 | 1.09E-03 | √ |
| TC | *BTN3A2* | 103 | 3.67E-07 | 7.17E-10 | 1.53E-10 | 3.03E-02 | 1.83E-02 | 2.08E-03 | 2.08E-03 | rs1800562 | 6.25E-10 |  |
| TC | *BVES* | 95 | 4.98E-04 | 1.19E-05 | 4.84E-06 | 2.90E-03 | 2.48E-02 | 2.04E-03 | 2.04E-03 | rs200229134 | 1.07E-02 | √ |
| TC | *C14orf102* | 6 | 1.00E+00 | 2.94E-05 | 4.58E-06 | 1.28E-02 | 7.89E-03 | 1.87E-06 | 1.87E-06 | noRS | 6.98E-03 | √ |
| TC | *C14orf93* | 6 | 1.00E+00 | 2.94E-05 | 4.58E-06 | 2.41E-02 | 1.37E-02 | 1.87E-06 | 1.87E-06 | rs142130715 | 4.82E-03 | √ |
| TC | *C16orf70* | 9 | 1.00E+00 | 4.49E-01 | 1.82E-01 | 2.72E-02 | 2.45E-02 | 2.20E-11 | 2.20E-11 | rs150369207 | 1.22E-04 | √ |
| TC | *C19orf12* | 13 | 5.79E-15 | 4.00E-56 | 5.00E-57 | 2.66E-02 | 6.07E-03 | 2.30E-96 | 2.30E-96 | rs151025990 | 6.10E-03 | √ |
| TC | *C19orf57* | 15 | 2.29E-07 | 7.17E-54 | 2.81E-54 | 3.14E-02 | 1.29E-02 | 2.94E-96 | 2.94E-96 | rs142208784 | 5.82E-04 | √ |
| TC | *C1orf74* | 17 | 1.96E-08 | 1.41E-34 | 1.18E-33 | 1.84E-02 | 9.21E-03 | 8.63E-31 | 8.63E-31 | rs144356617 | 8.13E-03 | √ |
| TC | *C6orf170* | 99 | 3.33E-03 | 4.04E-06 | 5.05E-07 | 2.30E-02 | 5.39E-03 | 2.52E-03 | 2.52E-03 | rs145215218 | 4.33E-02 | √ |
| TC | *C6orf203* | 98 | 4.89E-05 | 5.04E-07 | 1.14E-07 | 7.38E-03 | 1.25E-02 | 1.40E-03 | 1.40E-03 | rs139061896 | 1.09E-02 | √ |
| TC | *CAMK1* | 2 | 1.69E-05 | 1.74E-05 | 1.84E-03 | 1.00E+00 | 1.00E+00 | 1.00E+00 | 1.00E+00 | rs148267685 | 5.43E-03 | √ |
| TC | *CCDC97* | 7 | 1.00E+00 | 1.08E-07 | 1.35E-08 | 1.00E+00 | 1.00E+00 | 1.00E+00 | 1.00E+00 | rs201283333 | 3.13E-03 | √ |
| TC | *CENPQ* | 102 | 3.98E-05 | 6.93E-07 | 3.14E-07 | 3.01E-02 | 2.24E-02 | 2.04E-03 | 2.04E-03 | rs200286174 | 1.39E-02 | √ |
| TC | *CES5A* | 8 | 1.00E+00 | 2.16E-01 | 7.45E-02 | 2.22E-02 | 6.96E-03 | 2.05E-11 | 2.05E-11 | rs142090669 | 7.00E-02 | √ |
| TC | *CFB* | 75 | 1.00E+00 | 6.27E-01 | 3.01E-01 | 1.79E-02 | 7.11E-03 | 4.07E-03 | 4.07E-03 | rs36221133 | 1.01E-11 |  |
| TC | *CLSPN* | 5 | 1.13E-02 | 2.30E-03 | 6.70E-03 | 1.00E+00 | 1.00E+00 | 1.00E+00 | 1.00E+00 | rs146817855 | 3.68E-03 | √ |
| TC | *CNFN* | 15 | 1.00E+00 | 7.07E-03 | 1.28E-03 | 1.00E+00 | 1.00E+00 | 1.00E+00 | 1.00E+00 | rs201596848 | 5.06E-06 | √ |
| TC | *CNKSR1* | 15 | 8.82E-08 | 4.16E-32 | 1.95E-32 | 1.87E-02 | 1.87E-02 | 8.02E-31 | 8.02E-31 | rs141991719 | 5.30E-04 | √ |
| TC | *COX6A1P2* | 83 | 1.82E-03 | 1.75E-05 | 1.51E-05 | 2.70E-02 | 8.87E-03 | 2.85E-03 | 2.85E-03 | rs34617818 | 3.25E-04 | √ |
| TC | *CRIP1* | 6 | 1.00E+00 | 2.94E-05 | 4.58E-06 | 8.49E-03 | 1.83E-02 | 1.87E-06 | 1.87E-06 | noRS | 4.37E-03 | √ |
| TC | *CSNK2B* | 101 | 6.22E-07 | 1.29E-09 | 3.20E-10 | 2.79E-02 | 2.25E-02 | 1.45E-03 | 1.45E-03 | rs41560824 | 9.15E-12 |  |
| TC | *CTAGE1* | 4 | 1.29E-12 | 3.06E-41 | 3.82E-42 | 2.94E-02 | 2.33E-02 | 4.23E-44 | 4.23E-44 | rs201319761 | 1.35E-01 | √ |
| TC | *CTC-435M10.3* | 13 | 7.82E-15 | 2.69E-58 | 3.01E-58 | 1.98E-02 | 1.20E-02 | 2.36E-96 | 2.36E-96 | rs201283333 | 3.13E-03 | √ |
| TC | *CTD-2258A20.4* | 3 | 1.00E+00 | 6.32E-02 | 5.96E-02 | 6.17E-04 | 6.51E-03 | 6.31E-12 | 6.31E-12 | rs34621310 | 1.79E-02 | √ |
| TC | *CXCR6* | 5 | 1.00E+00 | 2.65E-04 | 1.28E-04 | 1.00E+00 | 1.00E+00 | 1.00E+00 | 1.00E+00 | rs200491743 | 1.81E-03 | √ |
| TC | *CYB5B* | 8 | 1.00E+00 | 2.98E-01 | 1.35E-01 | 2.02E-02 | 2.07E-02 | 1.97E-11 | 1.97E-11 | rs76116020 | 1.96E-04 | √ |
| TC | *CYGB* | 7 | 3.18E-02 | 8.26E-16 | 1.03E-16 | 2.48E-02 | 1.27E-02 | 3.17E-24 | 3.17E-24 | rs142734519 | 2.33E-03 | √ |
| TC | *CYP4F2* | 14 | 1.40E-12 | 2.33E-35 | 2.91E-36 | 6.41E-03 | 3.20E-03 | 2.60E-96 | 2.60E-96 | rs117610912 | 7.73E-04 | √ |
| TC | *DCDC2B* | 17 | 3.80E-04 | 5.78E-48 | 1.57E-48 | 2.65E-02 | 2.25E-02 | 8.30E-31 | 8.30E-31 | rs144152354 | 7.13E-03 | √ |
| TC | *DDR1* | 100 | 7.00E-06 | 2.59E-07 | 1.97E-07 | 6.20E-03 | 1.16E-02 | 1.44E-03 | 1.44E-03 | rs1634703 | 9.53E-12 |  |
| TC | *DECR2* | 9 | 1.00E+00 | 4.49E-01 | 1.82E-01 | 2.48E-02 | 5.93E-03 | 2.20E-11 | 2.20E-11 | rs201341279 | 2.28E-04 | √ |
| TC | *DENND1C* | 16 | 1.00E+00 | 1.62E-02 | 3.26E-03 | 1.00E+00 | 1.00E+00 | 1.00E+00 | 1.00E+00 | rs201305066 | 3.21E-03 | √ |
| TC | *DENND2C* | 20 | 9.34E-11 | 1.50E-160 | 1.87E-161 | 2.65E-02 | 2.01E-04 | 1.10E-300 | 1.10E-300 | rs115508967 | 6.40E-03 | √ |
| TC | *DHCR24* | 16 | 3.07E-08 | 4.69E-34 | 2.48E-33 | 2.42E-02 | 1.08E-02 | 8.29E-31 | 8.29E-31 | rs11591147 | 0.00E+00 |  |
| TC | *DHX16* | 82 | 1.51E-03 | 3.19E-05 | 3.56E-05 | 3.24E-02 | 2.49E-02 | 2.83E-03 | 2.83E-03 | rs2517490 | 1.26E-06 | √ |
| TC | *DLK2* | 69 | 1.87E-04 | 2.82E-07 | 3.41E-07 | 2.77E-02 | 2.60E-02 | 3.01E-03 | 3.01E-03 | rs145669581 | 4.26E-04 | √ |
| TC | *DNM2* | 14 | 1.00E+00 | 2.44E-05 | 3.05E-06 | 1.00E+00 | 1.00E+00 | 1.00E+00 | 1.00E+00 | rs139043155 | 4.41E-12 |  |
| TC | *DUSP22* | 102 | 4.73E-05 | 9.61E-07 | 2.70E-07 | 4.40E-03 | 2.28E-02 | 3.58E-03 | 3.58E-03 | rs202216214 | 9.73E-03 | √ |
| TC | *EIF3G* | 16 | 1.00E+00 | 1.62E-02 | 3.26E-03 | 1.00E+00 | 1.00E+00 | 1.00E+00 | 1.00E+00 | rs200547722 | 4.43E-12 |  |
| TC | *ELOVL4* | 76 | 1.74E-05 | 7.16E-10 | 5.38E-10 | 5.95E-03 | 1.91E-02 | 2.67E-03 | 2.67E-03 | rs189898207 | 6.45E-04 | √ |
| TC | *ELP2* | 3 | 3.85E-16 | 1.95E-45 | 2.44E-46 | 7.23E-03 | 7.13E-03 | 3.02E-44 | 3.02E-44 | rs145455632 | 2.57E-02 | √ |
| TC | *EMP2* | 12 | 1.00E+00 | 6.07E-01 | 1.95E-01 | 2.76E-02 | 1.63E-02 | 2.80E-11 | 2.80E-11 | rs201966261 | 1.51E-03 | √ |
| TC | *EPB41* | 17 | 1.96E-08 | 1.41E-34 | 1.18E-33 | 1.76E-02 | 1.32E-02 | 8.63E-31 | 8.63E-31 | rs111642750 | 2.43E-03 | √ |
| TC | *EPS8L1* | 13 | 5.79E-15 | 4.00E-56 | 5.00E-57 | 1.74E-02 | 2.50E-02 | 2.30E-96 | 2.30E-96 | rs4252572 | 7.89E-03 | √ |
| TC | *FAHD1* | 13 | 1.00E+00 | 1.00E+00 | 6.18E-01 | 2.01E-02 | 2.45E-03 | 3.12E-11 | 3.12E-11 | rs35816944 | 1.02E-03 | √ |
| TC | *FAM129A* | 5 | 1.13E-02 | 2.30E-03 | 6.70E-03 | 1.00E+00 | 1.00E+00 | 1.00E+00 | 1.00E+00 | rs202216870 | 2.16E-02 | √ |
| TC | *FAM213B* | 5 | 1.29E-04 | 2.55E-04 | 8.77E-03 | 1.00E+00 | 1.00E+00 | 1.00E+00 | 1.00E+00 | rs202216018 | 3.90E-03 | √ |
| TC | *FAM50B* | 89 | 4.25E-02 | 6.34E-04 | 5.62E-04 | 3.40E-03 | 8.56E-03 | 3.08E-03 | 3.08E-03 | rs78486783 | 4.62E-04 | √ |
| TC | *FBXO30* | 97 | 4.10E-03 | 2.12E-05 | 1.38E-05 | 3.45E-02 | 8.92E-03 | 3.39E-03 | 3.39E-03 | rs3811103 | 2.73E-04 | √ |
| TC | *FBXW9* | 12 | 2.34E-16 | 7.98E-59 | 1.37E-58 | 2.65E-02 | 6.76E-03 | 2.11E-96 | 2.11E-96 | rs121434369 | 2.17E-03 | √ |
| TC | *FCGBP* | 14 | 1.28E-09 | 2.62E-57 | 1.42E-57 | 3.00E-02 | 1.07E-02 | 2.50E-96 | 2.50E-96 | rs140296932 | 2.19E-03 | √ |
| TC | *GALNS* | 11 | 1.00E+00 | 4.73E-01 | 1.76E-01 | 2.42E-02 | 1.24E-02 | 2.77E-11 | 2.77E-11 | rs147032017 | 4.99E-07 | √ |
| TC | *GBP5* | 8 | 1.00E+00 | 1.00E+00 | 1.00E+00 | 4.52E-02 | 2.80E-02 | 2.72E-02 | 2.72E-02 | rs149274895 | 5.10E-03 | √ |
| TC | *GLO1* | 10 | 1.00E+00 | 4.52E-02 | 1.27E-02 | 1.00E+00 | 1.00E+00 | 1.00E+00 | 1.00E+00 | rs61758417 | 1.09E-03 | √ |
| TC | *GMPR2* | 7 | 1.00E+00 | 6.12E-05 | 8.69E-06 | 1.46E-03 | 1.40E-03 | 2.21E-06 | 2.21E-06 | rs76057705 | 2.33E-03 | √ |
| TC | *GNL1* | 103 | 1.11E-05 | 2.52E-07 | 1.43E-07 | 3.36E-02 | 5.23E-03 | 3.61E-03 | 3.61E-03 | rs7757648 | 4.03E-06 | √ |
| TC | *GNMT* | 100 | 5.44E-05 | 1.37E-06 | 7.23E-07 | 5.51E-03 | 8.80E-03 | 3.51E-03 | 3.51E-03 | rs145669581 | 4.26E-04 | √ |
| TC | *GTPBP2* | 97 | 1.51E-04 | 4.29E-06 | 3.51E-06 | 2.41E-03 | 1.27E-02 | 1.42E-03 | 1.42E-03 | rs145669581 | 4.26E-04 | √ |
| TC | *HECTD3* | 5 | 1.13E-02 | 2.30E-03 | 6.70E-03 | 1.00E+00 | 1.00E+00 | 1.00E+00 | 1.00E+00 | rs41306591 | 7.11E-04 | √ |
| TC | *HES5* | 16 | 3.07E-08 | 4.69E-34 | 2.48E-33 | 7.33E-04 | 1.50E-02 | 8.29E-31 | 8.29E-31 | rs202216018 | 3.90E-03 | √ |
| TC | *HINT3* | 102 | 3.98E-05 | 6.93E-07 | 3.14E-07 | 2.66E-02 | 2.00E-02 | 2.04E-03 | 2.04E-03 | rs139662554 | 5.43E-04 | √ |
| TC | *HIST1H2AJ* | 100 | 6.67E-04 | 1.78E-06 | 5.15E-07 | 3.81E-03 | 3.60E-03 | 3.53E-03 | 3.53E-03 | rs201148465 | 2.20E-07 | √ |
| TC | *HIST1H2BG* | 100 | 1.65E-02 | 7.27E-05 | 1.40E-05 | 3.02E-02 | 2.77E-02 | 2.57E-03 | 2.57E-03 | rs1800562 | 6.25E-10 |  |
| TC | *HIST1H2BK* | 79 | 1.80E-04 | 2.76E-07 | 8.57E-08 | 1.71E-02 | 1.83E-02 | 2.76E-03 | 2.76E-03 | rs114674760 | 1.19E-03 | √ |
| TC | *HIST1H3G* | 101 | 8.68E-06 | 4.28E-07 | 4.42E-07 | 5.59E-03 | 1.56E-02 | 2.05E-03 | 2.05E-03 | rs1800562 | 6.25E-10 |  |
| TC | *HLA-DOA* | 76 | 1.74E-05 | 7.16E-10 | 5.38E-10 | 1.72E-02 | 2.48E-02 | 2.67E-03 | 2.67E-03 | rs1057149 | 6.67E-07 | √ |
| TC | *HLA-DPB1* | 101 | 5.45E-03 | 2.96E-05 | 4.36E-06 | 1.44E-02 | 1.09E-02 | 2.53E-03 | 2.53E-03 | rs1057149 | 6.67E-07 | √ |
| TC | *HLA-E* | 10 | 1.00E+00 | 4.52E-02 | 1.27E-02 | 1.00E+00 | 1.00E+00 | 1.00E+00 | 1.00E+00 | rs7757648 | 4.03E-06 | √ |
| TC | *HPCA* | 20 | 1.50E-12 | 7.34E-157 | 9.17E-158 | 2.47E-02 | 1.82E-02 | 1.13E-300 | 1.13E-300 | rs144152354 | 7.13E-03 | √ |
| TC | *HSP90AB1* | 77 | 1.10E-04 | 2.03E-07 | 9.94E-08 | 2.47E-02 | 2.34E-02 | 2.71E-03 | 2.71E-03 | rs200526960 | 3.74E-03 | √ |
| TC | *ICMT* | 8 | 1.00E+00 | 1.00E+00 | 1.00E+00 | 5.83E-02 | 3.59E-02 | 2.72E-02 | 2.72E-02 | rs200409038 | 5.72E-04 | √ |
| TC | *IGFALS* | 7 | 1.00E+00 | 1.64E-01 | 8.74E-02 | 1.37E-02 | 2.51E-02 | 1.71E-11 | 1.71E-11 | rs35816944 | 1.02E-03 | √ |
| TC | *IMP4* | 7 | 1.00E+00 | 4.36E-32 | 5.45E-33 | 2.35E-03 | 1.62E-03 | 1.48E-60 | 1.48E-60 | noRS | 1.32E-02 | √ |
| TC | *ITGB1BP1* | 7 | 1.00E+00 | 4.36E-32 | 5.45E-33 | 1.25E-02 | 1.17E-02 | 1.48E-60 | 1.48E-60 | rs145511943 | 4.90E-02 | √ |
| TC | *ITPA* | 5 | 1.00E+00 | 6.23E-07 | 7.79E-08 | 1.59E-02 | 2.27E-02 | 3.55E-29 | 3.55E-29 | rs201878603 | 1.27E-03 | √ |
| TC | *JTB* | 19 | 5.43E-07 | 2.09E-33 | 1.60E-33 | 1.04E-03 | 1.10E-02 | 9.59E-31 | 9.59E-31 | rs144796295 | 5.80E-03 | √ |
| TC | *KARS* | 9 | 1.00E+00 | 4.49E-01 | 1.82E-01 | 6.03E-03 | 5.82E-03 | 2.20E-11 | 2.20E-11 | rs149900444 | 1.13E-04 | √ |
| TC | *KATNA1* | 98 | 4.89E-05 | 5.04E-07 | 1.14E-07 | 2.02E-02 | 2.24E-02 | 1.40E-03 | 1.40E-03 | rs147326739 | 1.01E-02 | √ |
| TC | *KCNC3* | 16 | 1.00E+00 | 1.62E-02 | 3.26E-03 | 1.00E+00 | 1.00E+00 | 1.00E+00 | 1.00E+00 | rs146344351 | 1.13E-61 |  |
| TC | *KIAA0090* | 15 | 5.96E-06 | 8.48E-33 | 1.08E-32 | 2.21E-02 | 8.12E-03 | 7.95E-31 | 7.95E-31 | rs146219234 | 3.67E-03 | √ |
| TC | *KIF22* | 8 | 1.00E+00 | 2.16E-01 | 7.45E-02 | 3.72E-03 | 7.86E-03 | 2.05E-11 | 2.05E-11 | rs148682647 | 5.58E-03 | √ |
| TC | *KLHDC9* | 19 | 3.12E-06 | 9.48E-35 | 4.78E-35 | 1.98E-02 | 2.04E-02 | 9.49E-31 | 9.49E-31 | rs143582871 | 2.48E-03 | √ |
| TC | *KLHL21* | 21 | 5.44E-05 | 2.56E-33 | 6.13E-34 | 2.28E-02 | 2.53E-03 | 1.06E-30 | 1.06E-30 | rs200409038 | 5.72E-04 | √ |
| TC | *KREMEN2* | 10 | 1.00E+00 | 7.09E-01 | 2.78E-01 | 2.99E-02 | 1.53E-02 | 2.44E-11 | 2.44E-11 | rs11466045 | 2.76E-03 | √ |
| TC | *KRI1* | 15 | 1.00E+00 | 1.10E-02 | 2.59E-03 | 1.00E+00 | 1.00E+00 | 1.00E+00 | 1.00E+00 | rs200547722 | 4.43E-12 |  |
| TC | *LAMA2* | 102 | 3.72E-06 | 1.82E-07 | 1.82E-07 | 3.33E-02 | 2.18E-02 | 2.07E-03 | 2.07E-03 | rs56035053 | 1.14E-02 | √ |
| TC | *LDLR* | 9 | 1.00E+00 | 1.48E-05 | 1.85E-06 | 1.00E+00 | 1.00E+00 | 1.00E+00 | 1.00E+00 | rs139043155 | 4.41E-12 |  |
| TC | *LEMD2* | 100 | 5.44E-05 | 1.37E-06 | 7.23E-07 | 2.91E-02 | 7.00E-03 | 3.51E-03 | 3.51E-03 | rs149067310 | 4.73E-03 | √ |
| TC | *LENG1* | 12 | 5.63E-18 | 1.13E-54 | 1.41E-55 | 3.05E-02 | 2.34E-02 | 2.14E-96 | 2.14E-96 | rs150437143 | 1.14E-03 | √ |
| TC | *LIPE* | 13 | 5.79E-15 | 4.00E-56 | 5.00E-57 | 2.18E-02 | 2.32E-02 | 2.30E-96 | 2.30E-96 | rs201596848 | 5.06E-06 | √ |
| TC | *LRRC16A* | 104 | 1.43E-04 | 2.59E-06 | 1.65E-06 | 1.35E-02 | 4.77E-03 | 1.53E-03 | 1.53E-03 | rs1800562 | 6.25E-10 |  |
| TC | *LYRM1* | 11 | 1.00E+00 | 4.73E-01 | 1.76E-01 | 7.13E-03 | 2.12E-02 | 2.77E-11 | 2.77E-11 | rs201001472 | 1.15E-03 | √ |
| TC | *MAN1A1* | 100 | 1.01E-05 | 1.66E-07 | 8.12E-08 | 2.33E-02 | 7.35E-03 | 1.44E-03 | 1.44E-03 | rs75607191 | 3.18E-02 | √ |
| TC | *MAP3K10* | 16 | 1.00E+00 | 1.62E-02 | 3.26E-03 | 1.00E+00 | 1.00E+00 | 1.00E+00 | 1.00E+00 | rs55793080 | 1.85E-03 | √ |
| TC | *MAP3K2* | 7 | 1.00E+00 | 4.36E-32 | 5.45E-33 | 2.59E-03 | 2.96E-03 | 1.48E-60 | 1.48E-60 | rs115176021 | 1.22E-02 | √ |
| TC | *MAU2* | 12 | 2.34E-16 | 7.98E-59 | 1.37E-58 | 1.72E-02 | 1.16E-02 | 2.11E-96 | 2.11E-96 | rs4808209 | 4.80E-04 | √ |
| TC | *MB21D1* | 102 | 4.73E-05 | 9.61E-07 | 2.70E-07 | 1.73E-02 | 1.97E-02 | 3.58E-03 | 3.58E-03 | rs142498397 | 4.29E-04 | √ |
| TC | *MED8* | 14 | 2.19E-08 | 1.84E-32 | 6.83E-32 | 2.00E-02 | 2.01E-02 | 7.56E-31 | 7.56E-31 | noRS | 2.18E-03 | √ |
| TC | *MEF2BNB* | 13 | 1.00E+00 | 3.72E-04 | 4.85E-05 | 1.00E+00 | 1.00E+00 | 1.00E+00 | 1.00E+00 | rs4808209 | 4.80E-04 | √ |
| TC | *MGST3* | 19 | 4.27E-07 | 7.37E-34 | 1.54E-34 | 7.27E-05 | 1.09E-02 | 9.51E-31 | 9.51E-31 | rs200583099 | 1.13E-02 | √ |
| TC | *MIXL1* | 17 | 1.96E-08 | 1.41E-34 | 1.18E-33 | 6.75E-03 | 1.26E-02 | 8.63E-31 | 8.63E-31 | rs143544007 | 2.66E-02 | √ |
| TC | *MLLT4-AS1* | 107 | 2.00E-06 | 3.07E-08 | 8.90E-09 | 2.51E-02 | 1.73E-02 | 2.14E-03 | 2.14E-03 | rs138998311 | 6.17E-21 |  |
| TC | *MRPL4* | 15 | 1.00E+00 | 7.07E-03 | 1.28E-03 | 1.00E+00 | 1.00E+00 | 1.00E+00 | 1.00E+00 | rs200547722 | 4.43E-12 |  |
| TC | *MUC22* | 76 | 1.74E-05 | 7.16E-10 | 5.38E-10 | 2.60E-02 | 6.01E-03 | 2.67E-03 | 2.67E-03 | rs41560824 | 9.15E-12 |  |
| TC | *MYLK4* | 101 | 6.22E-07 | 1.29E-09 | 3.20E-10 | 2.36E-02 | 1.35E-02 | 1.45E-03 | 1.45E-03 | rs144293488 | 5.30E-03 | √ |
| TC | *NBPF11* | 19 | 3.12E-06 | 9.48E-35 | 4.78E-35 | 4.45E-03 | 2.86E-03 | 9.49E-31 | 9.49E-31 | rs114731263 | 2.25E-03 | √ |
| TC | *NCOA6* | 5 | 1.00E+00 | 5.93E-07 | 7.61E-08 | 6.29E-04 | 5.23E-03 | 3.37E-29 | 3.37E-29 | rs150846093 | 1.83E-03 | √ |
| TC | *NDUFA11* | 8 | 1.00E+00 | 1.68E-07 | 2.10E-08 | 1.00E+00 | 1.00E+00 | 1.00E+00 | 1.00E+00 | rs113345491 | 7.43E-03 | √ |
| TC | *NDUFS2* | 19 | 2.86E-11 | 3.10E-155 | 3.87E-156 | 2.45E-02 | 1.87E-02 | 1.06E-300 | 1.06E-300 | rs143582871 | 2.48E-03 | √ |
| TC | *NFKBIE* | 14 | 1.00E+00 | 1.04E-01 | 2.81E-02 | 1.00E+00 | 1.00E+00 | 1.00E+00 | 1.00E+00 | rs200526960 | 3.74E-03 | √ |
| TC | *NPAS1* | 15 | 1.00E+00 | 7.07E-03 | 1.28E-03 | 1.00E+00 | 1.00E+00 | 1.00E+00 | 1.00E+00 | noRS | 1.18E-04 | √ |
| TC | *NPEPL1* | 6 | 1.00E+00 | 4.33E-07 | 5.41E-08 | 2.50E-03 | 1.17E-02 | 3.81E-29 | 3.81E-29 | rs201383880 | 7.25E-03 | √ |
| TC | *NPIPL1* | 9 | 1.00E+00 | 1.61E-01 | 5.10E-02 | 3.08E-02 | 1.80E-02 | 2.26E-11 | 2.26E-11 | rs147994015 | 3.83E-03 | √ |
| TC | *NRM* | 102 | 4.13E-06 | 2.32E-08 | 5.11E-09 | 5.30E-03 | 2.56E-02 | 1.46E-03 | 1.46E-03 | rs2517490 | 1.26E-06 | √ |
| TC | *NUMBL* | 17 | 5.76E-02 | 9.54E-47 | 1.19E-47 | 3.79E-03 | 5.15E-03 | 3.37E-96 | 3.37E-96 | rs55793080 | 1.85E-03 | √ |
| TC | *OAZ1* | 16 | 1.00E+00 | 1.62E-02 | 3.26E-03 | 1.00E+00 | 1.00E+00 | 1.00E+00 | 1.00E+00 | rs202084637 | 4.59E-03 | √ |
| TC | *OCEL1* | 15 | 1.00E+00 | 1.10E-02 | 2.59E-03 | 1.00E+00 | 1.00E+00 | 1.00E+00 | 1.00E+00 | rs201583022 | 2.32E-03 | √ |
| TC | *PAPD5* | 13 | 1.00E+00 | 1.00E+00 | 6.18E-01 | 2.87E-02 | 1.36E-02 | 3.12E-11 | 3.12E-11 | rs1131716 | 1.33E-02 | √ |
| TC | *PAQR4* | 7 | 1.00E+00 | 1.64E-01 | 8.74E-02 | 1.28E-03 | 3.33E-04 | 1.71E-11 | 1.71E-11 | rs11466045 | 2.76E-03 | √ |
| TC | *PEA15* | 17 | 1.96E-08 | 1.41E-34 | 1.18E-33 | 2.37E-02 | 2.13E-03 | 8.63E-31 | 8.63E-31 | rs199842829 | 7.39E-04 | √ |
| TC | *PFDN2* | 18 | 9.98E-13 | 1.51E-179 | 1.89E-180 | 2.78E-02 | 1.60E-02 | 1.00E-300 | 1.00E-300 | rs143582871 | 2.48E-03 | √ |
| TC | *PHIP* | 101 | 8.68E-06 | 4.28E-07 | 4.42E-07 | 1.97E-02 | 1.55E-02 | 2.05E-03 | 2.05E-03 | rs61729435 | 3.25E-02 | √ |
| TC | *PKIB* | 101 | 8.68E-06 | 4.28E-07 | 4.42E-07 | 1.76E-02 | 1.19E-02 | 2.05E-03 | 2.05E-03 | rs143986149 | 3.66E-02 | √ |
| TC | *PKN2* | 7 | 1.00E+00 | 1.00E+00 | 1.00E+00 | 4.75E-02 | 4.58E-02 | 2.21E-02 | 2.21E-02 | rs149274895 | 5.10E-03 | √ |
| TC | *PLEKHG1* | 101 | 6.22E-07 | 1.29E-09 | 3.20E-10 | 1.35E-02 | 1.04E-02 | 1.45E-03 | 1.45E-03 | rs75380096 | 1.23E-02 | √ |
| TC | *PLTP* | 7 | 3.80E-05 | 4.92E-12 | 1.37E-12 | 1.00E-02 | 1.06E-02 | 3.50E-29 | 3.50E-29 | rs142436120 | 3.46E-03 | √ |
| TC | *PMFBP1* | 10 | 1.00E+00 | 1.62E-01 | 5.13E-02 | 3.14E-02 | 8.04E-03 | 2.45E-11 | 2.45E-11 | rs35781168 | 3.32E-06 | √ |
| TC | *PNISR* | 74 | 1.00E+00 | 1.16E-03 | 2.03E-04 | 1.00E+00 | 1.00E+00 | 1.00E+00 | 1.00E+00 | rs147222511 | 2.97E-03 | √ |
| TC | *PPAN* | 16 | 1.00E+00 | 1.62E-02 | 3.26E-03 | 1.00E+00 | 1.00E+00 | 1.00E+00 | 1.00E+00 | rs200547722 | 4.43E-12 |  |
| TC | *PPARD* | 105 | 6.44E-06 | 3.99E-07 | 3.69E-07 | 2.75E-02 | 2.46E-02 | 2.11E-03 | 2.11E-03 | rs33959228 | 1.16E-06 | √ |
| TC | *PPP1R3G* | 98 | 4.89E-05 | 5.04E-07 | 1.14E-07 | 1.73E-02 | 9.80E-03 | 1.40E-03 | 1.40E-03 | rs34382405 | 1.96E-02 | √ |
| TC | *PPP6R1* | 12 | 1.70E-13 | 5.10E-43 | 8.50E-44 | 1.03E-02 | 2.23E-02 | 2.20E-96 | 2.20E-96 | rs115450119 | 1.79E-03 | √ |
| TC | *PRCC* | 17 | 1.96E-08 | 1.41E-34 | 1.18E-33 | 5.47E-03 | 2.46E-02 | 8.63E-31 | 8.63E-31 | rs142253945 | 1.27E-03 | √ |
| TC | *PRDM13* | 104 | 1.67E-05 | 2.13E-07 | 8.65E-08 | 2.64E-02 | 1.09E-02 | 1.48E-03 | 1.48E-03 | rs147222511 | 2.97E-03 | √ |
| TC | *PRKCH* | 5 | 1.00E+00 | 8.31E-06 | 1.19E-06 | 1.45E-02 | 1.65E-02 | 1.57E-06 | 1.57E-06 | rs144630332 | 2.30E-02 | √ |
| TC | *PRRC2A* | 106 | 1.08E-05 | 1.71E-07 | 5.47E-08 | 2.19E-03 | 2.14E-02 | 1.50E-03 | 1.50E-03 | rs41560824 | 9.15E-12 |  |
| TC | *PSENEN* | 14 | 7.53E-11 | 3.10E-48 | 3.87E-49 | 6.35E-03 | 8.74E-03 | 2.53E-96 | 2.53E-96 | rs61747965 | 9.38E-05 | √ |
| TC | *PTCRA* | 102 | 4.13E-06 | 2.32E-08 | 5.11E-09 | 1.88E-02 | 1.76E-02 | 1.46E-03 | 1.46E-03 | rs145669581 | 4.26E-04 | √ |
| TC | *PTK7* | 101 | 6.22E-07 | 1.29E-09 | 3.20E-10 | 1.53E-02 | 1.63E-02 | 1.45E-03 | 1.45E-03 | rs145669581 | 4.26E-04 | √ |
| TC | *PTPN1* | 5 | 1.00E+00 | 6.23E-07 | 7.79E-08 | 2.06E-02 | 1.86E-02 | 3.55E-29 | 3.55E-29 | rs150635098 | 7.09E-04 | √ |
| TC | *RAB8A* | 15 | 1.00E+00 | 7.07E-03 | 1.28E-03 | 1.00E+00 | 1.00E+00 | 1.00E+00 | 1.00E+00 | rs117610912 | 7.73E-04 | √ |
| TC | *RDBP* | 101 | 8.68E-06 | 4.28E-07 | 4.42E-07 | 1.44E-02 | 8.12E-03 | 2.05E-03 | 2.05E-03 | rs36221133 | 1.01E-11 |  |
| TC | *RHOA* | 4 | 1.00E+00 | 8.84E-04 | 4.85E-04 | 1.00E+00 | 1.00E+00 | 1.00E+00 | 1.00E+00 | rs146471142 | 2.90E-04 | √ |
| TC | *RHOH* | 11 | 4.04E-01 | 4.33E-02 | 3.49E-02 | 1.00E+00 | 1.00E+00 | 1.00E+00 | 1.00E+00 | rs202029831 | 2.39E-02 | √ |
| TC | *RIT1* | 20 | 9.34E-11 | 1.50E-160 | 1.87E-161 | 3.10E-02 | 1.71E-03 | 1.10E-300 | 1.10E-300 | rs142253945 | 1.27E-03 | √ |
| TC | *RNASET2* | 103 | 7.77E-06 | 2.60E-07 | 1.65E-07 | 8.06E-03 | 2.42E-02 | 2.09E-03 | 2.09E-03 | rs41269593 | 5.67E-03 | √ |
| TC | *RNF146* | 90 | 1.47E-02 | 7.14E-05 | 5.69E-05 | 2.28E-02 | 7.28E-03 | 3.11E-03 | 3.11E-03 | rs79339979 | 4.94E-02 | √ |
| TC | *ROCK1* | 3 | 3.85E-16 | 1.95E-45 | 2.44E-46 | 2.40E-02 | 1.41E-02 | 3.02E-44 | 3.02E-44 | rs113763420 | 8.98E-03 | √ |
| TC | *ROMO1* | 5 | 1.00E+00 | 6.23E-07 | 7.79E-08 | 1.43E-02 | 2.44E-02 | 3.55E-29 | 3.55E-29 | rs145409038 | 5.02E-03 | √ |
| TC | *RP1-180E22.3* | 97 | 2.93E-03 | 1.09E-05 | 1.50E-06 | 5.68E-03 | 7.47E-03 | 2.42E-03 | 2.42E-03 | rs11543266 | 3.05E-03 | √ |
| TC | *RP1-238O23.4* | 76 | 1.00E+00 | 6.86E-01 | 3.40E-01 | 2.45E-02 | 1.47E-02 | 4.10E-03 | 4.10E-03 | rs117069761 | 2.01E-03 | √ |
| TC | *RP1-80B9.2* | 76 | 1.74E-05 | 7.16E-10 | 5.38E-10 | 3.00E-02 | 2.56E-02 | 2.67E-03 | 2.67E-03 | rs139921713 | 2.00E-02 | √ |
| TC | *RP11-131H24.4* | 6 | 1.00E+00 | 2.94E-05 | 4.58E-06 | 2.64E-02 | 2.48E-02 | 1.87E-06 | 1.87E-06 | rs28929474 | 5.53E-14 |  |
| TC | *RP11-176H8.1* | 6 | 1.00E+00 | 2.94E-05 | 4.58E-06 | 1.01E-02 | 2.06E-02 | 1.87E-06 | 1.87E-06 | rs35867418 | 9.64E-05 | √ |
| TC | *RP11-206L10.7* | 19 | 7.98E-11 | 1.27E-160 | 1.59E-161 | 3.11E-03 | 3.44E-03 | 1.05E-300 | 1.05E-300 | rs138690517 | 3.25E-03 | √ |
| TC | *RP11-276E17.2* | 14 | 2.19E-08 | 1.84E-32 | 6.83E-32 | 5.19E-03 | 3.21E-03 | 7.56E-31 | 7.56E-31 | rs138713518 | 1.49E-02 | √ |
| TC | *RP11-457M11.5* | 100 | 5.44E-05 | 1.37E-06 | 7.23E-07 | 2.85E-02 | 1.48E-02 | 3.51E-03 | 3.51E-03 | rs114224033 | 1.25E-03 | √ |
| TC | *RP11-481J2.2* | 10 | 1.00E+00 | 1.62E-01 | 5.13E-02 | 1.26E-02 | 1.12E-02 | 2.45E-11 | 2.45E-11 | rs146685901 | 8.44E-03 | √ |
| TC | *RP11-536C5.7* | 17 | 1.96E-08 | 1.41E-34 | 1.18E-33 | 6.37E-03 | 9.01E-03 | 8.63E-31 | 8.63E-31 | rs199842829 | 7.39E-04 | √ |
| TC | *RP11-545I5.3* | 94 | 1.59E-03 | 4.20E-05 | 2.59E-05 | 1.83E-02 | 2.62E-02 | 2.05E-03 | 2.05E-03 | rs3811103 | 2.73E-04 | √ |
| TC | *RP5-1073O3.7* | 20 | 9.34E-11 | 1.50E-160 | 1.87E-161 | 1.45E-02 | 1.47E-02 | 1.10E-300 | 1.10E-300 | rs115508967 | 6.40E-03 | √ |
| TC | *RSPO1* | 2 | 2.12E-02 | 2.50E-02 | 1.00E+00 | 1.00E+00 | 1.00E+00 | 1.00E+00 | 1.00E+00 | rs200020734 | 4.06E-05 | √ |
| TC | *SCAND3* | 96 | 6.26E-04 | 1.09E-05 | 8.25E-06 | 2.25E-02 | 3.63E-03 | 1.40E-03 | 1.40E-03 | rs115051243 | 1.62E-03 | √ |
| TC | *SCML4* | 103 | 1.38E-06 | 7.68E-09 | 1.21E-09 | 2.02E-02 | 1.88E-02 | 1.46E-03 | 1.46E-03 | rs141211769 | 3.89E-02 | √ |
| TC | *SLC22A1* | 83 | 1.00E+00 | 1.38E-02 | 4.12E-03 | 2.18E-02 | 1.76E-02 | 2.17E-03 | 2.17E-03 | rs3798220 | 1.33E-26 |  |
| TC | *SLC35B2* | 100 | 7.00E-06 | 2.59E-07 | 1.97E-07 | 5.20E-03 | 1.05E-02 | 1.44E-03 | 1.44E-03 | rs200526960 | 3.74E-03 | √ |
| TC | *SNHG5* | 102 | 3.98E-05 | 6.93E-07 | 3.14E-07 | 1.02E-02 | 1.70E-02 | 2.04E-03 | 2.04E-03 | rs41271629 | 1.02E-02 | √ |
| TC | *SNRPB2* | 5 | 7.63E-03 | 8.61E-09 | 1.08E-09 | 1.84E-02 | 2.14E-02 | 3.47E-29 | 3.47E-29 | rs147795685 | 2.11E-03 | √ |
| TC | *SNX14* | 72 | 7.69E-02 | 1.21E-04 | 4.01E-05 | 7.81E-02 | 8.04E-02 | 5.54E-02 | 5.54E-02 | rs41271629 | 1.02E-02 | √ |
| TC | *SPAG5* | 7 | 3.18E-02 | 8.26E-16 | 1.03E-16 | 6.62E-03 | 2.28E-02 | 3.17E-24 | 3.17E-24 | rs61732688 | 2.47E-03 | √ |
| TC | *SPIRE2* | 7 | 1.00E+00 | 1.64E-01 | 8.74E-02 | 1.77E-02 | 3.19E-03 | 1.71E-11 | 1.71E-11 | rs145621381 | 1.35E-03 | √ |
| TC | *SRGAP2* | 19 | 1.27E-05 | 9.83E-33 | 2.00E-33 | 1.12E-02 | 1.08E-02 | 9.78E-31 | 9.78E-31 | rs34144261 | 2.15E-03 | √ |
| TC | *SRPK1* | 76 | 1.00E+00 | 6.86E-01 | 3.40E-01 | 4.65E-03 | 1.21E-02 | 4.10E-03 | 4.10E-03 | rs2766597 | 4.85E-05 | √ |
| TC | *SS18L2* | 4 | 1.00E+00 | 8.84E-04 | 4.85E-04 | 1.00E+00 | 1.00E+00 | 1.00E+00 | 1.00E+00 | rs146404227 | 6.38E-04 | √ |
| TC | *SSR2* | 19 | 3.12E-06 | 9.48E-35 | 4.78E-35 | 2.67E-02 | 1.65E-02 | 9.49E-31 | 9.49E-31 | rs142253945 | 1.27E-03 | √ |
| TC | *ST6GALNAC2* | 3 | 1.00E+00 | 6.17E-03 | 5.74E-03 | 1.74E-02 | 7.88E-03 | 2.88E-11 | 2.88E-11 | rs142734519 | 2.33E-03 | √ |
| TC | *STARD3* | 5 | 4.82E-03 | 2.50E-16 | 3.12E-17 | 5.66E-03 | 2.29E-03 | 2.21E-24 | 2.21E-24 | rs150365108 | 1.42E-04 | √ |
| TC | *SYNJ2* | 99 | 2.06E-03 | 9.09E-06 | 2.26E-06 | 1.95E-02 | 5.32E-03 | 2.50E-03 | 2.50E-03 | rs146885905 | 8.41E-03 | √ |
| TC | *TAP1* | 81 | 1.00E+00 | 3.31E-02 | 1.32E-02 | 2.67E-02 | 8.46E-03 | 2.12E-03 | 2.12E-03 | rs1057149 | 6.67E-07 | √ |
| TC | *TBCB* | 20 | 4.34E-02 | 6.27E-52 | 7.83E-53 | 1.38E-02 | 9.03E-04 | 3.99E-96 | 3.99E-96 | rs61747965 | 9.38E-05 | √ |
| TC | *TCEANC2* | 2 | 2.12E-02 | 2.50E-02 | 1.00E+00 | 1.00E+00 | 1.00E+00 | 1.00E+00 | 1.00E+00 | rs148513524 | 1.67E-03 | √ |
| TC | *TCF19* | 103 | 1.68E-06 | 2.17E-08 | 9.14E-09 | 1.28E-02 | 2.71E-02 | 2.07E-03 | 2.07E-03 | rs41560824 | 9.15E-12 |  |
| TC | *TCFL5* | 7 | 3.80E-05 | 4.92E-12 | 1.37E-12 | 2.15E-02 | 2.08E-03 | 3.50E-29 | 3.50E-29 | rs139953956 | 2.29E-04 | √ |
| TC | *TDP2* | 80 | 1.00E+00 | 2.92E-01 | 1.16E-01 | 1.96E-02 | 1.12E-02 | 4.32E-03 | 4.32E-03 | rs139103468 | 6.53E-03 | √ |
| TC | *TEX2* | 7 | 7.55E-05 | 2.17E-16 | 3.28E-17 | 2.44E-03 | 2.45E-02 | 2.97E-24 | 2.97E-24 | rs201148948 | 6.33E-04 | √ |
| TC | *THBS2* | 99 | 4.41E-04 | 1.70E-05 | 1.56E-05 | 2.83E-02 | 1.88E-02 | 1.41E-03 | 1.41E-03 | rs111656694 | 1.70E-02 | √ |
| TC | *THEMIS* | 97 | 1.51E-04 | 4.29E-06 | 3.51E-06 | 1.06E-02 | 2.64E-02 | 1.42E-03 | 1.42E-03 | rs145087914 | 9.08E-03 | √ |
| TC | *TIAM2* | 101 | 4.93E-06 | 9.02E-08 | 4.21E-08 | 1.85E-02 | 3.39E-03 | 1.45E-03 | 1.45E-03 | rs139039219 | 5.77E-04 | √ |
| TC | *TMC6* | 4 | 3.26E-02 | 1.09E-03 | 1.41E-03 | 2.00E-02 | 9.26E-03 | 4.03E-11 | 4.03E-11 | rs149892338 | 2.31E-04 | √ |
| TC | *TMEM110-MUSTN1* | 4 | 1.00E+00 | 8.84E-04 | 4.85E-04 | 1.00E+00 | 1.00E+00 | 1.00E+00 | 1.00E+00 | rs148806125 | 3.60E-04 | √ |
| TC | *TMEM125* | 17 | 1.96E-08 | 1.41E-34 | 1.18E-33 | 2.21E-02 | 1.72E-02 | 8.63E-31 | 8.63E-31 | noRS | 2.18E-03 | √ |
| TC | *TMEM147* | 8 | 1.00E+00 | 1.68E-07 | 2.10E-08 | 1.00E+00 | 1.00E+00 | 1.00E+00 | 1.00E+00 | rs61747965 | 9.38E-05 | √ |
| TC | *TMEM204* | 11 | 1.00E+00 | 4.73E-01 | 1.76E-01 | 2.07E-02 | 1.02E-02 | 2.77E-11 | 2.77E-11 | rs35816944 | 1.02E-03 | √ |
| TC | *TMEM242* | 67 | 3.35E-03 | 3.58E-07 | 6.83E-08 | 2.62E-02 | 1.44E-02 | 2.90E-03 | 2.90E-03 | rs73571886 | 8.63E-02 | √ |
| TC | *TMEM50A* | 5 | 1.13E-02 | 2.30E-03 | 6.70E-03 | 1.00E+00 | 1.00E+00 | 1.00E+00 | 1.00E+00 | rs34484514 | 2.73E-05 | √ |
| TC | *TP53I3* | 7 | 1.00E+00 | 4.36E-32 | 5.45E-33 | 1.73E-02 | 1.13E-02 | 1.48E-60 | 1.48E-60 | rs3208747 | 3.29E-03 | √ |
| TC | *TRAPPC2L* | 6 | 1.00E+00 | 2.09E-01 | 1.16E-01 | 6.97E-03 | 1.95E-03 | 1.50E-11 | 1.50E-11 | rs147032017 | 4.99E-07 | √ |
| TC | *TRAPPC2P1* | 14 | 1.00E+00 | 2.02E-03 | 3.06E-04 | 1.00E+00 | 1.00E+00 | 1.00E+00 | 1.00E+00 | rs144605370 | 8.20E-03 | √ |
| TC | *TRIM38* | 79 | 1.80E-04 | 2.76E-07 | 8.57E-08 | 3.25E-02 | 2.26E-02 | 2.76E-03 | 2.76E-03 | rs1800562 | 6.25E-10 |  |
| TC | *TSSK4* | 6 | 1.00E+00 | 2.94E-05 | 4.58E-06 | 3.12E-02 | 2.13E-02 | 1.87E-06 | 1.87E-06 | rs76057705 | 2.33E-03 | √ |
| TC | *TULP2* | 15 | 1.00E+00 | 7.07E-03 | 1.28E-03 | 1.00E+00 | 1.00E+00 | 1.00E+00 | 1.00E+00 | rs75479391 | 3.54E-04 | √ |
| TC | *U2AF1L4* | 15 | 9.76E-10 | 4.48E-58 | 1.16E-58 | 1.68E-02 | 2.45E-03 | 2.76E-96 | 2.76E-96 | rs61747965 | 9.38E-05 | √ |
| TC | *URB2* | 6 | 2.19E-02 | 4.97E-03 | 1.78E-02 | 1.00E+00 | 1.00E+00 | 1.00E+00 | 1.00E+00 | rs41304137 | 1.33E-02 | √ |
| TC | *VARS2* | 82 | 1.00E+00 | 1.15E-02 | 3.47E-03 | 3.73E-03 | 6.36E-03 | 2.15E-03 | 2.15E-03 | rs41560824 | 9.15E-12 |  |
| TC | *WDR77* | 16 | 2.35E-05 | 2.48E-48 | 6.59E-49 | 1.44E-03 | 5.51E-03 | 7.73E-31 | 7.73E-31 | rs202224198 | 1.44E-02 | √ |
| TC | *WTAP* | 102 | 3.98E-05 | 6.93E-07 | 3.14E-07 | 2.65E-02 | 1.56E-02 | 2.04E-03 | 2.04E-03 | rs2282143 | 1.71E-08 |  |
| TC | *WTIP* | 12 | 1.70E-13 | 5.10E-43 | 8.50E-44 | 4.27E-03 | 4.51E-03 | 2.20E-96 | 2.20E-96 | rs200278064 | 1.21E-02 | √ |
| TC | *XAB2* | 16 | 1.00E+00 | 1.56E-02 | 3.30E-03 | 1.00E+00 | 1.00E+00 | 1.00E+00 | 1.00E+00 | rs147423999 | 3.51E-03 | √ |
| TC | *XCR1* | 3 | 5.69E-02 | 1.30E-03 | 1.33E-03 | 1.00E+00 | 1.00E+00 | 1.00E+00 | 1.00E+00 | rs200491743 | 1.81E-03 | √ |
| TC | *YPEL5* | 4 | 1.00E+00 | 1.58E-04 | 5.83E-05 | 1.04E-02 | 2.48E-03 | 2.98E-04 | 2.98E-04 | rs149145987 | 8.43E-02 | √ |
| TC | *ZBTB32* | 7 | 1.00E+00 | 1.08E-07 | 1.35E-08 | 1.00E+00 | 1.00E+00 | 1.00E+00 | 1.00E+00 | rs61747965 | 9.38E-05 | √ |
| TC | *ZBTB48* | 19 | 5.43E-07 | 2.09E-33 | 1.60E-33 | 1.70E-02 | 1.91E-02 | 9.59E-31 | 9.59E-31 | rs200409038 | 5.72E-04 | √ |
| TC | *ZC2HC1B* | 87 | 5.05E-01 | 6.08E-03 | 1.74E-03 | 7.05E-03 | 2.03E-02 | 2.28E-03 | 2.28E-03 | rs45574234 | 7.52E-03 | √ |
| TC | *ZCCHC11* | 18 | 7.00E-14 | 2.63E-167 | 3.29E-168 | 2.46E-02 | 1.50E-02 | 1.01E-300 | 1.01E-300 | rs149073048 | 3.68E-03 | √ |
| TC | *ZDHHC7* | 7 | 1.00E+00 | 1.64E-01 | 8.74E-02 | 2.25E-02 | 2.12E-02 | 1.71E-11 | 1.71E-11 | rs113746582 | 1.53E-03 | √ |
| TC | *ZFP36* | 15 | 1.00E+00 | 7.07E-03 | 1.28E-03 | 1.00E+00 | 1.00E+00 | 1.00E+00 | 1.00E+00 | rs200026990 | 2.17E-03 | √ |
| TC | *ZNF134* | 18 | 2.30E-02 | 5.75E-54 | 7.19E-55 | 1.57E-02 | 2.28E-02 | 3.52E-96 | 3.52E-96 | rs144605370 | 8.20E-03 | √ |
| TC | *ZNF184* | 87 | 8.26E-04 | 9.35E-06 | 3.58E-06 | 1.63E-02 | 1.00E-02 | 1.89E-03 | 1.89E-03 | rs201148465 | 2.20E-07 | √ |
| TC | *ZNF343* | 6 | 1.00E+00 | 4.33E-07 | 5.41E-08 | 3.09E-02 | 5.64E-03 | 3.81E-29 | 3.81E-29 | rs61729228 | 7.16E-03 | √ |
| TC | *ZNF410* | 4 | 1.40E-01 | 1.44E-05 | 3.50E-06 | 7.10E-03 | 2.96E-03 | 1.37E-06 | 1.37E-06 | rs140227220 | 2.39E-03 | √ |
| TC | *ZNF444* | 15 | 1.00E+00 | 7.07E-03 | 1.28E-03 | 1.00E+00 | 1.00E+00 | 1.00E+00 | 1.00E+00 | rs115450119 | 1.79E-03 | √ |
| TC | *ZNF512B* | 6 | 1.00E+00 | 5.63E-07 | 7.04E-08 | 1.10E-02 | 2.38E-02 | 4.34E-29 | 4.34E-29 | rs201131002 | 1.60E-03 | √ |
| TC | *ZNF544* | 14 | 1.28E-09 | 2.62E-57 | 1.42E-57 | 2.40E-02 | 2.43E-02 | 2.50E-96 | 2.50E-96 | rs45580533 | 3.05E-04 | √ |
| TC | *ZNF551* | 16 | 1.00E+00 | 1.74E-30 | 2.18E-31 | 5.85E-04 | 2.25E-02 | 3.25E-96 | 3.25E-96 | rs140944722 | 7.35E-03 | √ |
| TC | *ZNF573* | 15 | 1.00E+00 | 7.07E-03 | 1.28E-03 | 1.00E+00 | 1.00E+00 | 1.00E+00 | 1.00E+00 | rs144294637 | 1.10E-03 | √ |
| TC | *ZNF611* | 15 | 1.00E+00 | 7.07E-03 | 1.28E-03 | 1.00E+00 | 1.00E+00 | 1.00E+00 | 1.00E+00 | rs143289804 | 6.72E-04 | √ |
| TC | *ZNF682* | 15 | 1.00E+00 | 7.07E-03 | 1.28E-03 | 1.00E+00 | 1.00E+00 | 1.00E+00 | 1.00E+00 | rs10411195 | 6.21E-08 | √ |
| TC | *ZNF77* | 7 | 1.00E+00 | 1.08E-07 | 1.35E-08 | 1.00E+00 | 1.00E+00 | 1.00E+00 | 1.00E+00 | rs138699938 | 6.09E-03 | √ |
| TC | *ZNF772* | 15 | 1.00E+00 | 7.07E-03 | 1.28E-03 | 1.00E+00 | 1.00E+00 | 1.00E+00 | 1.00E+00 | rs144605370 | 8.20E-03 | √ |
| TC | *ZNF812* | 16 | 1.00E+00 | 1.35E-02 | 2.59E-03 | 1.00E+00 | 1.00E+00 | 1.00E+00 | 1.00E+00 | rs34658893 | 2.08E-07 | √ |
| TC | *ZSCAN18* | 14 | 1.00E+00 | 2.02E-03 | 3.06E-04 | 1.00E+00 | 1.00E+00 | 1.00E+00 | 1.00E+00 | rs45580533 | 3.05E-04 | √ |
| TG | *ABHD8* | 22 | 1.10E-04 | 1.15E-57 | 1.44E-58 | 2.31E-02 | 7.76E-03 | 5.07E-167 | 5.07E-167 | rs142856821 | 2.50E-03 | √ |
| TG | *AC016745.1* | 7 | 3.28E-03 | 1.21E-03 | 3.53E-03 | 1.00E+00 | 1.00E+00 | 1.00E+00 | 1.00E+00 | rs144991866 | 2.41E-02 | √ |
| TG | *AC017002.2* | 8 | 9.22E-06 | 1.14E-05 | 1.80E-03 | 7.81E-01 | 7.62E-01 | 7.58E-01 | 7.58E-01 | rs142179264 | 3.33E-02 | √ |
| TG | *AC063977.1* | 17 | 5.39E-06 | 1.54E-79 | 1.92E-80 | 2.64E-02 | 1.36E-02 | 3.83E-167 | 3.83E-167 | rs78874436 | 6.77E-03 | √ |
| TG | *AC090616.2* | 12 | 1.00E+00 | 1.48E-18 | 1.85E-19 | 2.54E-03 | 1.81E-02 | 5.50E-56 | 5.50E-56 | rs199728576 | 5.20E-02 | √ |
| TG | *AC118344.1* | 16 | 1.44E-05 | 4.24E-85 | 5.30E-86 | 2.57E-02 | 1.63E-02 | 3.66E-167 | 3.66E-167 | rs199990200 | 4.94E-03 | √ |
| TG | *AC123768.5* | 6 | 1.00E+00 | 4.92E-02 | 1.65E-02 | 6.19E-03 | 8.60E-03 | 2.00E-06 | 2.00E-06 | rs199901818 | 2.03E-02 | √ |
| TG | *AL035696.1* | 102 | 1.00E+00 | 2.38E-06 | 6.98E-07 | 1.19E-03 | 1.51E-03 | 8.53E-15 | 8.53E-15 | rs148581187 | 2.06E-02 | √ |
| TG | *ALMS1* | 7 | 3.28E-03 | 1.21E-03 | 3.53E-03 | 1.00E+00 | 1.00E+00 | 1.00E+00 | 1.00E+00 | rs144558717 | 2.56E-03 | √ |
| TG | *ANGPTL4* | 16 | 1.44E-05 | 4.24E-85 | 5.30E-86 | 5.93E-03 | 1.84E-02 | 3.66E-167 | 3.66E-167 | rs116843064 | 4.19E-175 |  |
| TG | *ANKS1A* | 97 | 6.11E-01 | 1.82E-05 | 1.24E-05 | 2.71E-02 | 3.60E-03 | 8.06E-15 | 8.06E-15 | rs61734579 | 2.15E-04 | √ |
| TG | *ANO8* | 22 | 1.10E-04 | 1.15E-57 | 1.44E-58 | 1.26E-02 | 2.44E-02 | 5.07E-167 | 5.07E-167 | rs142856821 | 2.50E-03 | √ |
| TG | *APOC1* | 12 | 1.00E+00 | 9.02E-07 | 1.19E-07 | 1.87E-02 | 1.08E-03 | 1.86E-11 | 1.86E-11 | rs769455 | 1.14E-22 |  |
| TG | *APOC2* | 12 | 1.00E+00 | 2.20E-07 | 3.15E-08 | 1.43E-03 | 7.63E-03 | 1.79E-11 | 1.79E-11 | rs769455 | 1.14E-22 |  |
| TG | *APOE* | 14 | 1.00E+00 | 3.62E-06 | 4.93E-07 | 1.04E-02 | 1.43E-02 | 2.12E-11 | 2.12E-11 | rs769455 | 1.14E-22 |  |
| TG | *ARID3B* | 7 | 1.00E+00 | 1.39E-20 | 1.73E-21 | 3.86E-03 | 1.81E-02 | 4.46E-46 | 4.45E-46 | rs115331762 | 3.33E-03 | √ |
| TG | *ASB16* | 7 | 1.00E+00 | 5.75E-27 | 7.19E-28 | 1.74E-02 | 2.30E-02 | 3.25E-56 | 3.25E-56 | rs72836561 | 5.93E-64 |  |
| TG | *ATP5G1* | 14 | 1.00E+00 | 3.25E-18 | 4.06E-19 | 1.53E-02 | 8.72E-03 | 6.38E-56 | 6.38E-56 | rs149641326 | 1.18E-04 | √ |
| TG | *AXL* | 16 | 1.44E-05 | 4.24E-85 | 5.30E-86 | 2.04E-02 | 2.91E-03 | 3.66E-167 | 3.66E-167 | rs138799075 | 3.35E-03 | √ |
| TG | *B3GALT4* | 101 | 1.00E+00 | 2.18E-05 | 1.18E-05 | 2.23E-02 | 1.33E-02 | 8.47E-15 | 8.47E-15 | rs138097363 | 2.09E-03 | √ |
| TG | *BCL3* | 14 | 9.38E-01 | 2.48E-04 | 9.97E-05 | 2.17E-02 | 7.27E-03 | 2.14E-11 | 2.14E-11 | rs769455 | 1.14E-22 |  |
| TG | *BTBD9* | 82 | 9.40E-02 | 9.85E-04 | 5.42E-04 | 6.60E-03 | 1.78E-03 | 1.10E-14 | 1.10E-14 | rs117061525 | 3.37E-03 | √ |
| TG | *BTN3A2* | 103 | 1.01E-01 | 9.04E-07 | 3.90E-07 | 5.17E-06 | 1.61E-02 | 8.71E-15 | 8.71E-15 | rs114760306 | 3.43E-03 | √ |
| TG | *BVES* | 95 | 1.00E+00 | 1.14E-04 | 4.13E-05 | 1.03E-02 | 3.86E-03 | 2.07E-14 | 2.07E-14 | rs11961225 | 8.43E-02 | √ |
| TG | *C17orf103* | 12 | 1.00E+00 | 1.48E-18 | 1.85E-19 | 3.92E-04 | 6.55E-04 | 5.50E-56 | 5.50E-56 | rs375519519 | 4.39E-02 | √ |
| TG | *C19orf12* | 13 | 1.00E+00 | 3.14E-08 | 3.93E-09 | 3.10E-03 | 1.85E-03 | 1.95E-11 | 1.95E-11 | rs150514783 | 2.64E-02 | √ |
| TG | *C19orf29-AS1* | 18 | 6.70E-06 | 2.82E-79 | 3.52E-80 | 2.50E-02 | 2.05E-02 | 4.08E-167 | 4.08E-167 | rs200504647 | 1.49E-02 | √ |
| TG | *C19orf57* | 15 | 1.00E+00 | 2.79E-05 | 4.55E-06 | 1.98E-02 | 2.31E-03 | 2.48E-11 | 2.48E-11 | rs141666721 | 2.79E-04 | √ |
| TG | *C1orf200* | 3 | 1.69E-04 | 7.06E-05 | 9.42E-05 | 1.00E+00 | 1.00E+00 | 1.00E+00 | 1.00E+00 | rs61760196 | 1.17E-04 | √ |
| TG | *C2orf56* | 8 | 9.22E-06 | 1.14E-05 | 1.80E-03 | 7.61E-01 | 7.61E-01 | 7.58E-01 | 7.58E-01 | rs200868969 | 5.48E-04 | √ |
| TG | *C2orf84* | 10 | 4.19E-04 | 1.47E-04 | 3.39E-04 | 9.54E-01 | 9.36E-01 | 9.33E-01 | 9.33E-01 | rs3208747 | 1.68E-06 | √ |
| TG | *C6orf170* | 99 | 1.00E+00 | 7.82E-06 | 9.78E-07 | 2.46E-02 | 1.71E-02 | 2.26E-14 | 2.26E-14 | rs200303171 | 4.01E-02 | √ |
| TG | *C6orf203* | 98 | 1.00E+00 | 2.38E-05 | 1.07E-05 | 2.90E-02 | 3.94E-03 | 2.10E-14 | 2.10E-14 | rs139061896 | 3.12E-03 | √ |
| TG | *CCDC159* | 16 | 1.44E-05 | 4.24E-85 | 5.30E-86 | 2.57E-02 | 1.43E-02 | 3.66E-167 | 3.66E-167 | rs145464906 | 2.76E-12 |  |
| TG | *CCL25* | 28 | 3.11E-02 | 9.88E-75 | 1.23E-75 | 1.79E-02 | 2.11E-02 | 6.52E-167 | 6.52E-167 | rs116843064 | 4.19E-175 |  |
| TG | *CDCA5* | 8 | 1.00E+00 | 2.13E-22 | 1.24E-22 | 3.98E-03 | 1.87E-02 | 2.50E-43 | 2.50E-43 | rs607969 | 1.15E-05 | √ |
| TG | *CENPQ* | 102 | 1.00E+00 | 2.38E-06 | 6.98E-07 | 1.78E-02 | 1.78E-02 | 8.53E-15 | 8.53E-15 | rs150896181 | 1.01E-02 | √ |
| TG | *CEP164* | 5 | 9.61E-02 | 6.75E-28 | 8.44E-29 | 1.53E-02 | 1.15E-02 | 1.56E-43 | 1.56E-43 | rs138326449 | 3.57E-138 |  |
| TG | *CLEC4M* | 22 | 1.10E-04 | 1.15E-57 | 1.44E-58 | 2.17E-02 | 2.05E-02 | 5.07E-167 | 5.07E-167 | rs180746700 | 4.22E-03 | √ |
| TG | *COA5* | 9 | 8.82E-05 | 1.06E-04 | 2.49E-03 | 8.89E-01 | 8.85E-01 | 8.72E-01 | 8.72E-01 | rs200128112 | 1.20E-03 | √ |
| TG | *COL9A2* | 3 | 1.69E-04 | 7.06E-05 | 9.42E-05 | 1.00E+00 | 1.00E+00 | 1.00E+00 | 1.00E+00 | rs41301076 | 2.22E-03 | √ |
| TG | *COX6A1P2* | 83 | 1.77E-01 | 7.95E-04 | 3.24E-04 | 2.39E-03 | 1.42E-02 | 1.11E-14 | 1.11E-14 | rs148679597 | 9.05E-03 | √ |
| TG | *CSNK1G1* | 8 | 1.00E+00 | 6.00E-20 | 7.83E-21 | 7.52E-03 | 1.73E-03 | 5.10E-46 | 5.10E-46 | rs2228512 | 6.80E-03 | √ |
| TG | *CSNK2B* | 101 | 1.00E+00 | 3.64E-06 | 1.31E-06 | 2.89E-02 | 2.04E-03 | 8.51E-15 | 8.51E-15 | rs61995676 | 2.70E-23 |  |
| TG | *CTC-435M10.3* | 13 | 1.00E+00 | 1.04E-06 | 1.30E-07 | 4.03E-03 | 8.97E-03 | 1.99E-11 | 1.99E-11 | rs138799075 | 3.35E-03 | √ |
| TG | *CYP4F2* | 14 | 1.00E+00 | 2.43E-06 | 3.04E-07 | 1.02E-02 | 1.65E-02 | 2.20E-11 | 2.20E-11 | rs141337818 | 4.05E-03 | √ |
| TG | *DBP* | 19 | 2.59E-06 | 8.66E-61 | 1.08E-61 | 1.19E-02 | 1.39E-02 | 4.40E-167 | 4.40E-167 | rs34282921 | 4.27E-03 | √ |
| TG | *DDR1* | 100 | 1.00E+00 | 1.89E-07 | 4.40E-08 | 2.95E-02 | 9.45E-03 | 8.42E-15 | 8.42E-15 | rs2524229 | 1.31E-05 | √ |
| TG | *DHX16* | 82 | 9.40E-02 | 9.85E-04 | 5.42E-04 | 2.76E-03 | 6.52E-03 | 1.10E-14 | 1.10E-14 | rs1264446 | 2.59E-04 | √ |
| TG | *DHX33* | 12 | 1.00E+00 | 1.48E-18 | 1.85E-19 | 2.66E-02 | 1.73E-02 | 5.50E-56 | 5.50E-56 | rs142046798 | 1.17E-02 | √ |
| TG | *DKK3* | 8 | 1.00E+00 | 2.13E-22 | 1.24E-22 | 2.44E-02 | 2.35E-02 | 2.50E-43 | 2.50E-43 | rs61729668 | 8.20E-03 | √ |
| TG | *DLK2* | 69 | 1.49E-03 | 1.13E-06 | 1.36E-06 | 3.07E-02 | 1.80E-02 | 9.48E-15 | 9.48E-15 | rs145145776 | 1.21E-04 | √ |
| TG | *DUSP22* | 102 | 9.53E-02 | 1.93E-05 | 1.52E-05 | 1.86E-02 | 2.39E-02 | 8.51E-15 | 8.51E-15 | rs148581187 | 2.06E-02 | √ |
| TG | *EID2* | 22 | 1.10E-04 | 1.15E-57 | 1.44E-58 | 1.47E-02 | 1.82E-03 | 5.07E-167 | 5.07E-167 | rs74400185 | 5.18E-04 | √ |
| TG | *ELL3* | 7 | 1.00E+00 | 1.39E-20 | 1.73E-21 | 6.17E-05 | 8.22E-03 | 4.46E-46 | 4.45E-46 | rs55707100 | 8.60E-54 |  |
| TG | *ELOVL4* | 76 | 5.49E-04 | 1.18E-06 | 6.70E-07 | 9.94E-04 | 1.43E-02 | 1.04E-14 | 1.04E-14 | rs141417041 | 2.19E-02 | √ |
| TG | *EMR2* | 22 | 1.10E-04 | 1.15E-57 | 1.44E-58 | 1.18E-02 | 6.21E-03 | 5.07E-167 | 5.07E-167 | rs143659792 | 1.09E-03 | √ |
| TG | *ENY2* | 3 | 2.00E-168 | 1.16E-183 | 2.56E-175 | 1.85E-02 | 1.19E-02 | 1.57E-118 | 1.57E-118 | rs9774677 | 2.76E-02 | √ |
| TG | *EPS8L1* | 13 | 1.00E+00 | 3.14E-08 | 3.93E-09 | 1.89E-02 | 1.12E-02 | 1.95E-11 | 1.95E-11 | rs148304098 | 2.78E-03 | √ |
| TG | *FAM101B* | 14 | 1.00E+00 | 3.25E-18 | 4.06E-19 | 1.35E-02 | 2.22E-02 | 6.38E-56 | 6.38E-56 | rs191778127 | 1.60E-04 | √ |
| TG | *FAM124B* | 10 | 4.19E-04 | 1.47E-04 | 3.39E-04 | 9.51E-01 | 9.49E-01 | 9.33E-01 | 9.33E-01 | rs200577403 | 7.85E-03 | √ |
| TG | *FAM50B* | 89 | 5.40E-01 | 6.46E-04 | 4.89E-04 | 2.81E-02 | 1.80E-02 | 7.58E-15 | 7.58E-15 | rs144214404 | 2.32E-02 | √ |
| TG | *FBXO30* | 97 | 6.11E-01 | 1.82E-05 | 1.24E-05 | 2.33E-03 | 1.82E-02 | 8.06E-15 | 8.06E-15 | rs185072292 | 1.14E-02 | √ |
| TG | *FBXW9* | 12 | 1.00E+00 | 2.20E-07 | 3.15E-08 | 1.45E-02 | 5.65E-03 | 1.79E-11 | 1.79E-11 | rs183982301 | 4.61E-03 | √ |
| TG | *FCGBP* | 14 | 1.00E+00 | 3.62E-06 | 4.93E-07 | 3.05E-02 | 2.18E-02 | 2.12E-11 | 2.12E-11 | rs74400185 | 5.18E-04 | √ |
| TG | *FOSL1* | 8 | 1.00E+00 | 2.13E-22 | 1.24E-22 | 2.96E-02 | 1.19E-02 | 2.50E-43 | 2.50E-43 | noRS | 1.70E-03 | √ |
| TG | *GAL3ST2* | 9 | 8.82E-05 | 1.06E-04 | 2.49E-03 | 8.84E-01 | 8.85E-01 | 8.72E-01 | 8.72E-01 | rs189711563 | 2.72E-03 | √ |
| TG | *GNL1* | 103 | 1.12E-01 | 3.57E-06 | 2.16E-06 | 2.46E-02 | 1.29E-02 | 8.59E-15 | 8.59E-15 | rs1264446 | 2.59E-04 | √ |
| TG | *GNMT* | 100 | 1.64E-01 | 1.37E-05 | 8.90E-06 | 2.82E-02 | 9.94E-03 | 8.34E-15 | 8.34E-15 | rs145145776 | 1.21E-04 | √ |
| TG | *GORASP2* | 10 | 4.19E-04 | 1.47E-04 | 3.39E-04 | 9.52E-01 | 9.50E-01 | 9.33E-01 | 9.33E-01 | rs34546065 | 5.62E-03 | √ |
| TG | *GTPBP2* | 97 | 1.00E+00 | 6.72E-06 | 2.22E-06 | 1.81E-02 | 2.12E-02 | 2.10E-14 | 2.10E-14 | rs61743561 | 9.53E-03 | √ |
| TG | *HDAC4* | 9 | 1.45E-04 | 9.04E-06 | 6.43E-06 | 8.93E-01 | 8.81E-01 | 8.78E-01 | 8.77E-01 | rs143274079 | 9.56E-04 | √ |
| TG | *HEXA* | 7 | 1.00E+00 | 6.77E-02 | 2.75E-02 | 1.00E-02 | 5.23E-03 | 2.34E-06 | 2.34E-06 | rs138058578 | 2.65E-03 | √ |
| TG | *HINT3* | 102 | 1.00E+00 | 2.38E-06 | 6.98E-07 | 7.68E-03 | 1.36E-02 | 8.53E-15 | 8.53E-15 | rs145594277 | 2.05E-02 | √ |
| TG | *HIST1H2AJ* | 100 | 3.35E-01 | 1.66E-05 | 1.18E-05 | 2.79E-02 | 6.47E-03 | 8.41E-15 | 8.41E-15 | rs147161729 | 1.07E-02 | √ |
| TG | *HIST1H2BG* | 100 | 1.00E+00 | 1.90E-04 | 5.51E-05 | 2.51E-02 | 1.95E-02 | 2.31E-14 | 2.31E-14 | rs114760306 | 3.43E-03 | √ |
| TG | *HIST1H2BK* | 79 | 5.92E-03 | 1.12E-05 | 4.65E-06 | 2.75E-02 | 1.95E-02 | 1.07E-14 | 1.07E-14 | rs200393466 | 1.83E-02 | √ |
| TG | *HIST1H3G* | 101 | 1.00E+00 | 8.32E-06 | 4.77E-06 | 6.30E-03 | 4.41E-03 | 8.57E-15 | 8.57E-15 | rs114760306 | 3.43E-03 | √ |
| TG | *HLA-DOA* | 76 | 5.49E-04 | 1.18E-06 | 6.70E-07 | 9.17E-03 | 1.08E-02 | 1.04E-14 | 1.04E-14 | rs138097363 | 2.09E-03 | √ |
| TG | *HLA-DPB1* | 101 | 1.00E+00 | 6.09E-06 | 7.61E-07 | 1.22E-02 | 1.00E-02 | 2.27E-14 | 2.27E-14 | rs138097363 | 2.09E-03 | √ |
| TG | *HLF* | 12 | 1.00E+00 | 1.48E-18 | 1.85E-19 | 1.35E-02 | 1.58E-02 | 5.50E-56 | 5.50E-56 | rs201714381 | 3.19E-02 | √ |
| TG | *HMHA1* | 22 | 1.10E-04 | 1.15E-57 | 1.44E-58 | 2.02E-02 | 7.29E-03 | 5.07E-167 | 5.07E-167 | rs139251906 | 2.36E-04 | √ |
| TG | *HSP90AB1* | 77 | 1.09E-04 | 1.84E-08 | 2.19E-08 | 2.46E-03 | 1.43E-02 | 1.05E-14 | 1.05E-14 | rs115815965 | 2.89E-03 | √ |
| TG | *HSPB9* | 15 | 1.00E+00 | 2.65E-18 | 3.31E-19 | 1.21E-04 | 1.21E-02 | 6.84E-56 | 6.84E-56 | rs34016093 | 4.00E-03 | √ |
| TG | *IDH1* | 10 | 4.19E-04 | 1.47E-04 | 3.39E-04 | 9.46E-01 | 9.38E-01 | 9.33E-01 | 9.33E-01 | rs146158348 | 7.64E-03 | √ |
| TG | *IMP4* | 7 | 1.54E-03 | 6.82E-08 | 2.40E-08 | 1.64E-02 | 1.67E-02 | 6.66E-40 | 6.66E-40 | rs138837389 | 5.29E-02 | √ |
| TG | *IQCH* | 8 | 1.00E+00 | 6.00E-20 | 7.83E-21 | 1.23E-02 | 1.06E-02 | 5.10E-46 | 5.10E-46 | rs200477258 | 2.54E-02 | √ |
| TG | *ITGB1BP1* | 7 | 1.54E-03 | 6.82E-08 | 2.40E-08 | 1.66E-03 | 9.14E-03 | 6.66E-40 | 6.66E-40 | rs200225020 | 6.10E-04 | √ |
| TG | *KATNA1* | 98 | 1.00E+00 | 2.38E-05 | 1.07E-05 | 1.84E-02 | 1.45E-02 | 2.10E-14 | 2.10E-14 | rs201128649 | 7.19E-03 | √ |
| TG | *KYNU* | 10 | 4.19E-04 | 1.47E-04 | 3.39E-04 | 9.45E-01 | 9.52E-01 | 9.33E-01 | 9.33E-01 | rs77631323 | 8.31E-02 | √ |
| TG | *LAMA2* | 102 | 6.60E-01 | 5.63E-06 | 2.69E-06 | 2.33E-02 | 1.39E-02 | 8.67E-15 | 8.67E-15 | rs149305108 | 8.44E-03 | √ |
| TG | *LEMD2* | 100 | 1.64E-01 | 1.37E-05 | 8.90E-06 | 3.12E-03 | 7.57E-03 | 8.34E-15 | 8.34E-15 | rs149206085 | 3.69E-03 | √ |
| TG | *LENG1* | 12 | 1.00E+00 | 5.34E-08 | 6.67E-09 | 1.87E-02 | 5.13E-03 | 1.81E-11 | 1.81E-11 | rs118056835 | 2.27E-04 | √ |
| TG | *LIPE* | 13 | 1.00E+00 | 3.14E-08 | 3.93E-09 | 2.30E-02 | 8.70E-03 | 1.95E-11 | 1.95E-11 | rs147133204 | 3.18E-03 | √ |
| TG | *LRRC16A* | 104 | 1.00E+00 | 5.98E-06 | 1.33E-06 | 2.47E-02 | 1.72E-02 | 2.26E-14 | 2.26E-14 | rs200533860 | 1.48E-02 | √ |
| TG | *LTBP1* | 10 | 4.19E-04 | 1.47E-04 | 3.39E-04 | 9.39E-01 | 9.46E-01 | 9.33E-01 | 9.33E-01 | rs201116141 | 8.13E-03 | √ |
| TG | *MAN1A1* | 100 | 1.00E+00 | 1.18E-06 | 2.75E-07 | 2.74E-03 | 1.45E-02 | 8.42E-15 | 8.42E-15 | rs150485975 | 2.44E-03 | √ |
| TG | *MAP3K2* | 7 | 1.54E-03 | 6.82E-08 | 2.40E-08 | 1.23E-03 | 1.65E-02 | 6.66E-40 | 6.66E-40 | rs34785867 | 1.96E-02 | √ |
| TG | *MAT2A* | 8 | 9.22E-06 | 1.14E-05 | 1.80E-03 | 7.76E-01 | 7.76E-01 | 7.58E-01 | 7.58E-01 | rs41290043 | 1.94E-03 | √ |
| TG | *MAU2* | 12 | 1.00E+00 | 2.20E-07 | 3.15E-08 | 1.56E-02 | 8.71E-03 | 1.79E-11 | 1.79E-11 | rs4808209 | 1.59E-06 | √ |
| TG | *MB21D1* | 102 | 9.53E-02 | 1.93E-05 | 1.52E-05 | 2.86E-02 | 5.67E-03 | 8.51E-15 | 8.51E-15 | rs80338794 | 1.09E-02 | √ |
| TG | *MED24* | 6 | 1.00E+00 | 4.74E-27 | 5.93E-28 | 1.12E-02 | 2.28E-02 | 2.82E-56 | 2.82E-56 | rs144470339 | 1.12E-02 | √ |
| TG | *MEN1* | 8 | 1.00E+00 | 2.13E-22 | 1.24E-22 | 3.61E-04 | 1.23E-02 | 2.50E-43 | 2.50E-43 | rs607969 | 1.15E-05 | √ |
| TG | *MLLT4-AS1* | 107 | 9.28E-02 | 3.61E-07 | 1.41E-07 | 4.71E-03 | 1.31E-02 | 8.98E-15 | 8.97E-15 | rs149243529 | 1.83E-02 | √ |
| TG | *MORN2* | 9 | 8.82E-05 | 1.06E-04 | 2.49E-03 | 8.95E-01 | 8.92E-01 | 8.72E-01 | 8.72E-01 | rs147872027 | 7.32E-04 | √ |
| TG | *MRPL12* | 12 | 1.00E+00 | 1.48E-18 | 1.85E-19 | 6.24E-04 | 1.26E-02 | 5.50E-56 | 5.50E-56 | rs200938354 | 2.38E-04 | √ |
| TG | *MRPL20* | 3 | 1.69E-04 | 7.06E-05 | 9.42E-05 | 1.00E+00 | 1.00E+00 | 1.00E+00 | 1.00E+00 | rs116266251 | 5.23E-03 | √ |
| TG | *MRPL21* | 8 | 1.00E+00 | 2.13E-22 | 1.24E-22 | 1.33E-03 | 1.12E-02 | 2.50E-43 | 2.50E-43 | rs115294924 | 3.33E-04 | √ |
| TG | *MRPL44* | 6 | 8.34E-02 | 7.71E-03 | 9.39E-03 | 1.00E+00 | 1.00E+00 | 1.00E+00 | 1.00E+00 | rs142594795 | 6.51E-03 | √ |
| TG | *MSL1* | 6 | 1.00E+00 | 4.74E-27 | 5.93E-28 | 4.64E-03 | 2.12E-02 | 2.82E-56 | 2.82E-56 | rs144470339 | 1.12E-02 | √ |
| TG | *MUC22* | 76 | 5.49E-04 | 1.18E-06 | 6.70E-07 | 3.80E-03 | 1.47E-02 | 1.04E-14 | 1.04E-14 | rs78957773 | 1.21E-07 | √ |
| TG | *MYLK4* | 101 | 1.00E+00 | 3.64E-06 | 1.31E-06 | 9.31E-03 | 4.16E-03 | 8.51E-15 | 8.51E-15 | rs144293488 | 1.62E-03 | √ |
| TG | *NOL10* | 10 | 4.19E-04 | 1.47E-04 | 3.39E-04 | 9.53E-01 | 9.56E-01 | 9.33E-01 | 9.33E-01 | rs142020517 | 1.87E-02 | √ |
| TG | *NR3C2* | 10 | 1.00E+00 | 4.66E-02 | 2.14E-02 | 1.00E+00 | 1.00E+00 | 1.00E+00 | 1.00E+00 | rs117523998 | 1.17E-02 | √ |
| TG | *NRM* | 102 | 1.00E+00 | 8.56E-06 | 5.38E-06 | 6.10E-04 | 2.41E-02 | 8.57E-15 | 8.57E-15 | rs1264446 | 2.59E-04 | √ |
| TG | *NUMBL* | 17 | 1.00E+00 | 2.13E-03 | 4.88E-04 | 2.71E-02 | 1.82E-05 | 2.85E-11 | 2.85E-11 | rs199990200 | 4.94E-03 | √ |
| TG | *ORMDL3* | 8 | 1.00E+00 | 3.02E-24 | 3.78E-25 | 2.43E-02 | 1.84E-02 | 3.44E-56 | 3.44E-56 | rs144470339 | 1.12E-02 | √ |
| TG | *PAAF1* | 7 | 1.00E+00 | 1.36E-23 | 2.90E-24 | 2.51E-02 | 2.35E-02 | 2.32E-43 | 2.32E-43 | rs144949543 | 2.92E-03 | √ |
| TG | *PATL2* | 20 | 1.00E+00 | 1.00E+00 | 1.00E+00 | 2.61E-02 | 1.44E-03 | 5.93E-06 | 5.93E-06 | rs147945181 | 1.37E-03 | √ |
| TG | *PCBP1* | 9 | 8.82E-05 | 1.06E-04 | 2.49E-03 | 8.91E-01 | 8.87E-01 | 8.72E-01 | 8.72E-01 | rs138771201 | 2.42E-02 | √ |
| TG | *PDK2* | 15 | 1.00E+00 | 1.81E-17 | 2.26E-18 | 1.72E-02 | 1.08E-02 | 6.72E-56 | 6.72E-56 | rs78434961 | 9.12E-04 | √ |
| TG | *PGLYRP4* | 3 | 1.69E-04 | 7.06E-05 | 9.42E-05 | 1.00E+00 | 1.00E+00 | 1.00E+00 | 1.00E+00 | rs2233868 | 8.33E-03 | √ |
| TG | *PHIP* | 101 | 1.00E+00 | 8.32E-06 | 4.77E-06 | 2.57E-02 | 2.06E-02 | 8.57E-15 | 8.57E-15 | rs145854631 | 4.54E-02 | √ |
| TG | *PKIB* | 101 | 1.00E+00 | 8.32E-06 | 4.77E-06 | 9.21E-03 | 2.88E-03 | 8.57E-15 | 8.57E-15 | rs146353927 | 5.27E-03 | √ |
| TG | *PLCB2* | 7 | 1.00E+00 | 1.39E-20 | 1.73E-21 | 1.44E-03 | 2.32E-02 | 4.46E-46 | 4.45E-46 | rs7169142 | 2.03E-03 | √ |
| TG | *PLEKHG1* | 101 | 1.00E+00 | 3.64E-06 | 1.31E-06 | 6.57E-03 | 4.86E-03 | 8.51E-15 | 8.51E-15 | rs75380096 | 2.31E-03 | √ |
| TG | *PNISR* | 74 | 1.00E+00 | 5.75E-03 | 9.45E-04 | 2.16E-02 | 2.41E-03 | 9.99E-15 | 9.99E-15 | rs138873880 | 9.98E-03 | √ |
| TG | *POMC* | 9 | 8.82E-05 | 1.06E-04 | 2.49E-03 | 8.89E-01 | 8.79E-01 | 8.72E-01 | 8.72E-01 | rs151168784 | 1.05E-02 | √ |
| TG | *PPARD* | 105 | 5.65E-01 | 3.34E-05 | 2.71E-05 | 2.32E-02 | 1.39E-02 | 8.84E-15 | 8.84E-15 | rs61734579 | 2.15E-04 | √ |
| TG | *PPM1B* | 10 | 4.19E-04 | 1.47E-04 | 3.39E-04 | 9.44E-01 | 9.41E-01 | 9.33E-01 | 9.33E-01 | rs144732922 | 2.21E-03 | √ |
| TG | *PPP1R15A* | 22 | 1.10E-04 | 1.15E-57 | 1.44E-58 | 1.25E-02 | 2.46E-02 | 5.07E-167 | 5.07E-167 | rs147854826 | 7.08E-04 | √ |
| TG | *PPP1R3G* | 98 | 1.00E+00 | 2.38E-05 | 1.07E-05 | 1.10E-03 | 2.88E-03 | 2.10E-14 | 2.10E-14 | rs22662 | 6.76E-02 | √ |
| TG | *PPP6R1* | 12 | 1.00E+00 | 9.02E-07 | 1.19E-07 | 9.47E-04 | 2.36E-02 | 1.86E-11 | 1.86E-11 | rs148304098 | 2.78E-03 | √ |
| TG | *PRCD* | 12 | 1.00E+00 | 1.48E-18 | 1.85E-19 | 1.58E-02 | 1.42E-02 | 5.50E-56 | 5.50E-56 | rs141700513 | 3.36E-03 | √ |
| TG | *PRDM13* | 104 | 1.00E+00 | 1.06E-05 | 6.04E-06 | 1.36E-02 | 1.39E-02 | 8.69E-15 | 8.68E-15 | rs138873880 | 9.98E-03 | √ |
| TG | *PRKCDBP* | 7 | 1.00E+00 | 1.36E-23 | 2.90E-24 | 2.82E-02 | 1.37E-02 | 2.32E-43 | 2.32E-43 | rs114115159 | 1.78E-04 | √ |
| TG | *PRRC2A* | 106 | 7.27E-01 | 4.00E-07 | 1.05E-07 | 2.23E-02 | 2.08E-02 | 8.81E-15 | 8.81E-15 | rs61995676 | 2.70E-23 |  |
| TG | *PSENEN* | 14 | 9.38E-01 | 2.48E-04 | 9.97E-05 | 6.51E-03 | 9.21E-03 | 2.14E-11 | 2.14E-11 | rs201138169 | 1.09E-03 | √ |
| TG | *PTCRA* | 102 | 1.00E+00 | 8.56E-06 | 5.38E-06 | 1.76E-02 | 2.03E-02 | 8.57E-15 | 8.57E-15 | rs145145776 | 1.21E-04 | √ |
| TG | *PTK7* | 101 | 1.00E+00 | 3.64E-06 | 1.31E-06 | 2.31E-02 | 4.86E-03 | 8.51E-15 | 8.51E-15 | rs145145776 | 1.21E-04 | √ |
| TG | *RDBP* | 101 | 1.00E+00 | 8.32E-06 | 4.77E-06 | 1.18E-02 | 6.00E-04 | 8.57E-15 | 8.57E-15 | rs61995676 | 2.70E-23 |  |
| TG | *RHOH* | 11 | 1.00E+00 | 1.18E-02 | 3.66E-03 | 1.00E+00 | 1.00E+00 | 1.00E+00 | 1.00E+00 | rs142570098 | 7.74E-03 | √ |
| TG | *RNASET2* | 103 | 7.16E-01 | 1.07E-05 | 5.17E-06 | 2.78E-02 | 6.60E-03 | 8.73E-15 | 8.73E-15 | rs17856382 | 2.90E-03 | √ |
| TG | *RNF146* | 90 | 8.24E-01 | 7.23E-04 | 5.47E-04 | 1.34E-02 | 4.66E-03 | 7.64E-15 | 7.64E-15 | rs139745911 | 5.39E-03 | √ |
| TG | *RNF181* | 9 | 1.45E-04 | 9.04E-06 | 6.43E-06 | 9.03E-01 | 8.84E-01 | 8.78E-01 | 8.77E-01 | rs41290043 | 1.94E-03 | √ |
| TG | *RP1-180E22.3* | 97 | 1.00E+00 | 1.03E-05 | 1.41E-06 | 1.56E-02 | 1.23E-02 | 2.17E-14 | 2.17E-14 | rs11543266 | 3.26E-04 | √ |
| TG | *RP1-80B9.2* | 76 | 5.49E-04 | 1.18E-06 | 6.70E-07 | 2.66E-02 | 1.43E-02 | 1.04E-14 | 1.04E-14 | rs139362604 | 4.25E-02 | √ |
| TG | *RP11-356J5.12* | 6 | 4.25E-05 | 1.24E-25 | 7.54E-26 | 1.38E-02 | 4.32E-03 | 1.76E-43 | 1.76E-43 | rs78952668 | 3.82E-03 | √ |
| TG | *RP11-442N24__B.1* | 3 | 1.69E-04 | 7.06E-05 | 9.42E-05 | 1.00E+00 | 1.00E+00 | 1.00E+00 | 1.00E+00 | rs201460078 | 7.94E-03 | √ |
| TG | *RP11-457M11.5* | 100 | 1.64E-01 | 1.37E-05 | 8.90E-06 | 2.86E-02 | 2.35E-02 | 8.34E-15 | 8.34E-15 | rs114760306 | 3.43E-03 | √ |
| TG | *RP11-488L18.10* | 3 | 1.69E-04 | 7.06E-05 | 9.42E-05 | 1.00E+00 | 1.00E+00 | 1.00E+00 | 1.00E+00 | rs55762230 | 9.64E-04 | √ |
| TG | *RP11-545I5.3* | 94 | 1.00E+00 | 7.15E-04 | 3.19E-04 | 6.53E-03 | 3.19E-03 | 2.07E-14 | 2.07E-14 | rs185072292 | 1.14E-02 | √ |
| TG | *RP11-711M9.1* | 10 | 4.19E-04 | 1.47E-04 | 3.39E-04 | 9.61E-01 | 9.50E-01 | 9.33E-01 | 9.33E-01 | rs13420401 | 4.19E-03 | √ |
| TG | *RP11-817O13.8* | 11 | 1.00E+00 | 9.54E-17 | 1.19E-17 | 2.50E-02 | 2.09E-02 | 6.96E-46 | 6.96E-46 | rs143478022 | 1.05E-03 | √ |
| TG | *RP11-875O11.2* | 6 | 1.03E-85 | 1.65E-161 | 7.68E-159 | 2.58E-02 | 1.49E-02 | 2.87E-118 | 2.87E-118 | rs141021862 | 1.12E-04 | √ |
| TG | *RPL27* | 14 | 1.00E+00 | 3.25E-18 | 4.06E-19 | 2.46E-02 | 6.28E-03 | 6.38E-56 | 6.38E-56 | rs201961848 | 5.83E-07 | √ |
| TG | *RPL37A* | 9 | 8.82E-05 | 1.06E-04 | 2.49E-03 | 8.79E-01 | 8.80E-01 | 8.72E-01 | 8.72E-01 | rs192418356 | 2.85E-03 | √ |
| TG | *RPS27A* | 9 | 8.82E-05 | 1.06E-04 | 2.49E-03 | 8.99E-01 | 8.88E-01 | 8.72E-01 | 8.72E-01 | rs34891804 | 6.13E-03 | √ |
| TG | *RPSAP58* | 20 | 2.20E-06 | 7.12E-68 | 8.91E-69 | 2.17E-02 | 2.30E-02 | 4.44E-167 | 4.44E-167 | rs139015019 | 3.61E-02 | √ |
| TG | *SAMD14* | 14 | 1.00E+00 | 3.62E-21 | 4.80E-22 | 2.32E-02 | 8.79E-03 | 6.33E-56 | 6.33E-56 | rs78434961 | 9.12E-04 | √ |
| TG | *SCAND3* | 96 | 1.00E+00 | 1.18E-05 | 3.86E-06 | 2.96E-02 | 6.00E-03 | 2.07E-14 | 2.06E-14 | rs147161729 | 1.07E-02 | √ |
| TG | *SCML4* | 103 | 1.00E+00 | 4.66E-07 | 1.58E-07 | 3.02E-02 | 1.87E-02 | 8.59E-15 | 8.59E-15 | rs35555197 | 5.20E-02 | √ |
| TG | *SFT2D3* | 9 | 8.82E-05 | 1.06E-04 | 2.49E-03 | 9.02E-01 | 8.95E-01 | 8.72E-01 | 8.72E-01 | rs34785867 | 1.96E-02 | √ |
| TG | *SH3YL1* | 10 | 4.19E-04 | 1.47E-04 | 3.39E-04 | 9.52E-01 | 9.42E-01 | 9.33E-01 | 9.33E-01 | rs144604911 | 1.76E-01 | √ |
| TG | *SLC35B2* | 100 | 1.00E+00 | 1.89E-07 | 4.40E-08 | 1.38E-02 | 9.71E-03 | 8.42E-15 | 8.42E-15 | rs115815965 | 2.89E-03 | √ |
| TG | *SLFN12L* | 14 | 1.00E+00 | 3.25E-18 | 4.06E-19 | 2.88E-02 | 3.32E-05 | 6.38E-56 | 6.38E-56 | rs200841311 | 6.00E-03 | √ |
| TG | *SNHG5* | 102 | 1.00E+00 | 2.38E-06 | 6.98E-07 | 4.29E-03 | 1.05E-02 | 8.53E-15 | 8.53E-15 | rs138968407 | 1.49E-02 | √ |
| TG | *SNX14* | 72 | 1.00E+00 | 3.53E-03 | 5.69E-04 | 2.63E-02 | 1.84E-02 | 9.78E-15 | 9.78E-15 | rs138968407 | 1.49E-02 | √ |
| TG | *SNX32* | 7 | 1.00E+00 | 1.36E-23 | 2.90E-24 | 2.88E-02 | 5.47E-04 | 2.32E-43 | 2.32E-43 | noRS | 1.70E-03 | √ |
| TG | *SPHK1* | 14 | 1.00E+00 | 3.62E-21 | 4.80E-22 | 1.36E-02 | 6.53E-03 | 6.33E-56 | 6.33E-56 | rs141700513 | 3.36E-03 | √ |
| TG | *SPTBN5* | 13 | 1.00E+00 | 8.73E-01 | 3.49E-01 | 4.34E-03 | 1.38E-02 | 3.89E-06 | 3.89E-06 | rs2412710 | 4.84E-14 |  |
| TG | *SSFA2* | 7 | 3.28E-03 | 1.21E-03 | 3.53E-03 | 1.00E+00 | 1.00E+00 | 1.00E+00 | 1.00E+00 | rs137921064 | 4.96E-03 | √ |
| TG | *STOML1* | 5 | 1.00E+00 | 5.36E-02 | 2.28E-02 | 2.81E-03 | 1.33E-02 | 1.74E-06 | 1.74E-06 | rs115331762 | 3.33E-03 | √ |
| TG | *SUPT6H* | 6 | 1.00E+00 | 4.74E-27 | 5.93E-28 | 2.76E-02 | 6.02E-03 | 2.82E-56 | 2.82E-56 | rs200744136 | 1.19E-04 | √ |
| TG | *SYNJ2* | 99 | 1.00E+00 | 6.32E-05 | 9.28E-06 | 2.97E-02 | 1.38E-02 | 2.24E-14 | 2.24E-14 | rs61748681 | 5.32E-03 | √ |
| TG | *TBCB* | 20 | 1.00E+00 | 2.38E-03 | 4.73E-04 | 2.41E-02 | 2.22E-03 | 3.37E-11 | 3.37E-11 | rs201138169 | 1.09E-03 | √ |
| TG | *TCF19* | 103 | 7.31E-01 | 5.23E-06 | 2.90E-06 | 6.97E-03 | 2.32E-02 | 8.68E-15 | 8.67E-15 | rs41273264 | 4.66E-23 |  |
| TG | *THBS2* | 99 | 1.00E+00 | 7.51E-05 | 3.35E-05 | 2.65E-02 | 1.23E-02 | 2.13E-14 | 2.13E-14 | rs141680830 | 2.81E-02 | √ |
| TG | *THEMIS* | 97 | 1.00E+00 | 6.72E-06 | 2.22E-06 | 2.71E-02 | 2.04E-02 | 2.10E-14 | 2.10E-14 | rs139745911 | 5.39E-03 | √ |
| TG | *TIAM2* | 101 | 1.00E+00 | 1.07E-06 | 2.26E-07 | 2.86E-02 | 2.19E-03 | 8.51E-15 | 8.51E-15 | rs143540866 | 1.83E-02 | √ |
| TG | *TMED3* | 6 | 1.00E+00 | 4.92E-02 | 1.65E-02 | 1.25E-02 | 1.52E-02 | 2.00E-06 | 2.00E-06 | rs140240186 | 5.38E-02 | √ |
| TG | *TMEM182* | 13 | 2.30E-03 | 1.21E-04 | 6.66E-05 | 1.00E+00 | 1.00E+00 | 1.00E+00 | 1.00E+00 | rs143081976 | 2.69E-02 | √ |
| TG | *TMEM242* | 67 | 4.76E-05 | 6.33E-06 | 3.39E-05 | 1.65E-02 | 3.34E-03 | 9.12E-15 | 9.12E-15 | rs145635490 | 4.75E-02 | √ |
| TG | *TP53I3* | 7 | 1.54E-03 | 6.82E-08 | 2.40E-08 | 2.51E-02 | 2.30E-02 | 6.66E-40 | 6.66E-40 | rs3208747 | 1.68E-06 | √ |
| TG | *TPRKB* | 7 | 3.28E-03 | 1.21E-03 | 3.53E-03 | 1.00E+00 | 1.00E+00 | 1.00E+00 | 1.00E+00 | rs200915592 | 8.84E-03 | √ |
| TG | *TRIM38* | 79 | 5.92E-03 | 1.12E-05 | 4.65E-06 | 1.83E-03 | 1.62E-02 | 1.07E-14 | 1.07E-14 | rs114760306 | 3.43E-03 | √ |
| TG | *TRMT61B* | 10 | 4.19E-04 | 1.47E-04 | 3.39E-04 | 9.57E-01 | 9.50E-01 | 9.33E-01 | 9.33E-01 | rs185951007 | 3.59E-03 | √ |
| TG | *TTC27* | 9 | 8.82E-05 | 1.06E-04 | 2.49E-03 | 9.02E-01 | 8.89E-01 | 8.72E-01 | 8.72E-01 | rs201116141 | 8.13E-03 | √ |
| TG | *TTLL6* | 8 | 1.00E+00 | 1.63E-25 | 2.04E-26 | 7.65E-03 | 1.10E-02 | 3.77E-56 | 3.77E-56 | rs149641326 | 1.18E-04 | √ |
| TG | *TUBA3D* | 6 | 8.34E-02 | 7.71E-03 | 9.39E-03 | 1.00E+00 | 1.00E+00 | 1.00E+00 | 1.00E+00 | rs202134743 | 2.14E-02 | √ |
| TG | *U2AF1L4* | 15 | 1.00E+00 | 5.03E-06 | 6.83E-07 | 1.45E-02 | 1.65E-02 | 2.33E-11 | 2.33E-11 | rs201138169 | 1.09E-03 | √ |
| TG | *UBR5* | 3 | 2.00E-168 | 1.16E-183 | 2.56E-175 | 3.64E-04 | 9.52E-03 | 1.57E-118 | 1.57E-118 | rs138624614 | 2.14E-02 | √ |
| TG | *USP6* | 12 | 1.00E+00 | 1.48E-18 | 1.85E-19 | 5.08E-03 | 2.37E-02 | 5.50E-56 | 5.50E-56 | rs200424489 | 2.59E-03 | √ |
| TG | *VSIG10L* | 22 | 1.10E-04 | 1.15E-57 | 1.44E-58 | 2.61E-02 | 1.19E-02 | 5.07E-167 | 5.07E-167 | rs78874436 | 6.77E-03 | √ |
| TG | *WTAP* | 102 | 1.00E+00 | 2.38E-06 | 6.98E-07 | 9.43E-03 | 1.95E-02 | 8.53E-15 | 8.53E-15 | rs34130495 | 6.26E-05 | √ |
| TG | *WTIP* | 12 | 1.00E+00 | 9.02E-07 | 1.19E-07 | 2.88E-03 | 2.46E-03 | 1.86E-11 | 1.86E-11 | rs200278064 | 3.00E-03 | √ |
| TG | *ZC3H3* | 5 | 1.20E-84 | 3.38E-123 | 4.23E-124 | 2.92E-02 | 6.46E-03 | 2.50E-118 | 2.50E-118 | rs199941693 | 9.08E-05 | √ |
| TG | *ZNF134* | 18 | 1.00E+00 | 2.24E-03 | 5.24E-04 | 2.17E-02 | 1.10E-02 | 2.98E-11 | 2.98E-11 | rs149525702 | 4.99E-03 | √ |
| TG | *ZNF184* | 87 | 1.00E+00 | 2.64E-05 | 6.94E-06 | 2.17E-02 | 1.64E-02 | 1.91E-14 | 1.91E-14 | rs200393466 | 1.83E-02 | √ |
| TG | *ZNF233* | 16 | 1.44E-05 | 4.24E-85 | 5.30E-86 | 1.42E-03 | 2.46E-02 | 3.66E-167 | 3.66E-167 | rs28399653 | 3.02E-19 |  |
| TG | *ZNF333* | 22 | 1.10E-04 | 1.15E-57 | 1.44E-58 | 4.02E-03 | 1.90E-05 | 5.07E-167 | 5.07E-167 | rs143659792 | 1.09E-03 | √ |
| TG | *ZNF544* | 14 | 1.00E+00 | 3.62E-06 | 4.93E-07 | 1.69E-02 | 6.51E-03 | 2.12E-11 | 2.12E-11 | rs144974711 | 3.06E-04 | √ |
| TG | *ZNF551* | 16 | 1.00E+00 | 1.00E+00 | 5.07E-01 | 9.86E-03 | 1.81E-02 | 3.76E-11 | 3.76E-11 | rs149525702 | 4.99E-03 | √ |
| TG | *ZNF558* | 19 | 2.51E-04 | 2.17E-68 | 2.71E-69 | 1.98E-02 | 1.46E-02 | 4.74E-167 | 4.74E-167 | rs116843064 | 4.19E-175 |  |
| TG | *ZNF581* | 22 | 1.10E-04 | 1.15E-57 | 1.44E-58 | 1.86E-02 | 2.00E-02 | 5.07E-167 | 5.07E-167 | rs11880748 | 8.20E-03 | √ |
| TG | *ZNF599* | 28 | 3.11E-02 | 9.88E-75 | 1.23E-75 | 3.19E-03 | 6.73E-03 | 6.52E-167 | 6.52E-167 | rs138079924 | 1.48E-03 | √ |
| TG | *ZNF699* | 16 | 1.44E-05 | 4.24E-85 | 5.30E-86 | 1.06E-02 | 1.02E-02 | 3.66E-167 | 3.66E-167 | rs201401770 | 3.07E-03 | √ |
| TG | *ZNF780A* | 16 | 3.79E-10 | 1.84E-69 | 2.29E-70 | 1.74E-02 | 1.15E-02 | 3.56E-167 | 3.56E-167 | rs74400185 | 5.18E-04 | √ |

**Note:** novel: defined by whether the gene contained GWAS significant SNPs (*P*_GWAS_ < 5×10^-8^).

## Table S7. Identified genes associated with the nine immune-related diseases

| diseases | Gene | *m* | BT | SKATO | SKAT | HMP | ACAT | Best SNP | *P*GWAS | novel |
| --- | --- | --- | --- | --- | --- | --- | --- | --- | --- | --- |
| IBD | *AAGAB* | 107 | 3.38E-01 | 1.00E+00 | 1.00E+00 | 4.60E-08 | 6.91E-08 | rs141910407 | 6.70E-103 |  |
| IBD | *ABHD16A* | 107 | 7.42E-01 | 5.51E-03 | 1.00E+00 | 2.83E-30 | 4.25E-30 | rs7653276 | 1.51E-05 | √ |
| IBD | *AC008697.1* | 260 | 4.83E-02 | 1.09E-09 | 5.71E-05 | 8.76E-25 | 1.31E-24 | rs45527431 | 5.68E-32 |  |
| IBD | *AC079630.2* | 368 | 4.74E-01 | 1.00E+00 | 1.00E+00 | 2.23E-03 | 3.36E-03 | rs1143679 | 5.03E-48 |  |
| IBD | *ADO* | 368 | 1.93E-01 | 3.96E-05 | 1.00E+00 | 2.15E-04 | 3.26E-04 | rs2736332 | 4.83E-18 |  |
| IBD | *AGER* | 368 | 6.65E-01 | 1.00E+00 | 1.00E+00 | 3.35E-47 | 5.03E-47 | rs141910407 | 6.70E-103 |  |
| IBD | *AGPAT1* | 368 | 4.36E-01 | 1.00E+00 | 1.00E+00 | 4.02E-50 | 6.03E-50 | rs141910407 | 6.70E-103 |  |
| IBD | *AIF1* | 368 | 4.12E-01 | 1.21E-05 | 1.00E+00 | 2.01E-31 | 3.02E-31 | rs141910407 | 6.70E-103 |  |
| IBD | *AIRE* | 368 | 3.32E-01 | 2.41E-06 | 7.57E-06 | 3.09E-11 | 4.63E-11 | rs283478 | 4.12E-03 | √ |
| IBD | *AMIGO3* | 368 | 7.58E-02 | 4.10E-08 | 2.78E-07 | 4.24E-23 | 6.36E-23 | rs58688157 | 2.97E-11 |  |
| IBD | *AMT* | 368 | 1.42E-18 | 7.17E-11 | 1.10E-07 | 5.80E-19 | 8.71E-19 | rs8177429 | 1.32E-06 | √ |
| IBD | *AP001055.6* | 1039 | 2.27E-03 | 1.00E+00 | 1.00E+00 | 1.29E-11 | 1.93E-11 | rs141910407 | 6.70E-103 |  |
| IBD | *AP001056.1* | 12 | 9.03E-03 | 3.43E-06 | 3.90E-06 | 2.92E-11 | 4.38E-11 | rs1143679 | 5.03E-48 |  |
| IBD | *AP001058.3* | 12 | 2.33E-02 | 1.32E-08 | 1.32E-05 | 3.63E-11 | 5.45E-11 | rs17849501 | 1.81E-59 |  |
| IBD | *APEH* | 12 | 8.46E-01 | 4.37E-12 | 5.81E-08 | 3.28E-23 | 4.91E-23 | rs141910407 | 6.70E-103 |  |
| IBD | *APOBR* | 12 | 8.82E-01 | 1.99E-09 | 9.82E-10 | 2.14E-04 | 3.20E-04 | rs35000415 | 1.86E-45 |  |
| IBD | *APOM* | 12 | 4.12E-01 | 1.43E-04 | 4.73E-01 | 3.27E-30 | 4.90E-30 | rs115484360 | 2.93E-102 |  |
| IBD | *ARFRP1* | 14 | 3.29E-02 | 7.53E-01 | 1.00E+00 | 1.03E-11 | 1.55E-11 | rs115484360 | 2.93E-102 |  |
| IBD | *ATF6B* | 8 | 3.24E-01 | 1.00E+00 | 1.00E+00 | 6.45E-51 | 9.67E-51 | rs114720630 | 6.72E-72 |  |
| IBD | *ATG16L1* | 8 | 5.60E-02 | 1.00E+00 | 1.00E+00 | 5.83E-15 | 8.75E-15 | rs141910407 | 6.70E-103 |  |
| IBD | *ATP6V0A1* | 16 | 6.66E-04 | 1.00E+00 | 1.00E+00 | 1.77E-04 | 2.82E-04 | rs2736332 | 4.83E-18 |  |
| IBD | *ATP6V1G2* | 10 | 1.32E-01 | 1.00E+00 | 1.00E+00 | 4.43E-06 | 6.65E-06 | rs9273327 | 1.76E-100 |  |
| IBD | *AZI1* | 7 | 1.04E-24 | 1.00E+00 | 1.00E+00 | 1.00E+00 | 1.00E+00 | rs45527431 | 5.68E-32 |  |
| IBD | *BAG6* | 7 | 1.68E-01 | 1.34E-02 | 1.00E+00 | 1.01E-30 | 1.52E-30 | rs45527431 | 5.68E-32 |  |
| IBD | *BRD7* | 52 | 4.39E-01 | 1.00E+00 | 1.00E+00 | 1.01E-04 | 1.50E-04 | rs45527431 | 5.68E-32 |  |
| IBD | *BRWD1* | 17 | 4.45E-02 | 1.00E+00 | 1.00E+00 | 3.40E-15 | 5.10E-15 | rs45527431 | 5.68E-32 |  |
| IBD | *BSN* | 10 | 3.73E-04 | 5.64E-13 | 1.09E-03 | 5.77E-23 | 8.65E-23 | rs45527431 | 5.68E-32 |  |
| IBD | *BTNL2* | 191 | 9.36E-01 | 1.00E+00 | 1.00E+00 | 1.46E-07 | 2.19E-07 | rs45527431 | 5.68E-32 |  |
| IBD | *C11orf30* | 140 | 9.55E-02 | 1.00E+00 | 1.00E+00 | 2.09E-18 | 3.13E-18 | rs141910407 | 6.70E-103 |  |
| IBD | *C1orf106* | 245 | 6.13E-03 | 1.00E+00 | 1.00E+00 | 5.16E-07 | 7.73E-07 | rs58688157 | 2.97E-11 |  |
| IBD | *C2* | 332 | 9.09E-01 | 1.19E-01 | 1.00E+00 | 3.49E-43 | 5.23E-43 | rs141910407 | 6.70E-103 |  |
| IBD | *C20orf195* | 8 | 7.46E-02 | 1.00E+00 | 1.00E+00 | 6.37E-06 | 9.56E-06 | rs9852014 | 2.26E-36 |  |
| IBD | *C21orf33* | 8 | 7.38E-03 | 5.64E-05 | 3.14E-05 | 5.07E-11 | 7.61E-11 | rs9852014 | 2.26E-36 |  |
| IBD | *C3orf62* | 73 | 1.99E-02 | 6.11E-04 | 2.64E-06 | 1.61E-17 | 2.42E-17 | rs141910407 | 6.70E-103 |  |
| IBD | *C4A* | 19 | 1.26E-01 | 1.00E+00 | 1.00E+00 | 1.86E-42 | 2.78E-42 | rs141910407 | 6.70E-103 |  |
| IBD | *C4B* | 136 | 7.86E-01 | 1.00E+00 | 1.00E+00 | 1.78E-42 | 2.67E-42 | rs76623030 | 7.87E-05 | √ |
| IBD | *C5orf56* | 136 | 2.73E-01 | 7.32E-05 | 1.00E+00 | 2.72E-13 | 4.09E-13 | rs141910407 | 6.70E-103 |  |
| IBD | *C5orf62* | 277 | 4.24E-01 | 1.00E+00 | 1.00E+00 | 9.60E-04 | 1.47E-03 | rs141910407 | 6.70E-103 |  |
| IBD | *C6orf47* | 277 | 1.41E-01 | 3.24E-02 | 9.66E-01 | 4.54E-30 | 6.82E-30 | rs3747093 | 2.88E-14 |  |
| IBD | *C6orf48* | 277 | 1.76E-01 | 1.46E-03 | 4.82E-05 | 1.00E+00 | 1.00E+00 | rs6889239 | 2.19E-18 |  |
| IBD | *C9orf46* | 277 | 7.90E-01 | 1.00E+00 | 1.00E+00 | 1.18E-04 | 1.76E-04 | rs147027375 | 1.55E-96 |  |
| IBD | *CAMSAP2* | 277 | 7.57E-02 | 1.00E+00 | 1.00E+00 | 9.63E-09 | 1.44E-08 | rs353608 | 2.93E-11 |  |
| IBD | *CARD6* | 277 | 4.42E-07 | 1.00E+00 | 1.00E+00 | 1.00E+00 | 1.00E+00 | rs35251378 | 3.61E-13 |  |
| IBD | *CBLL1* | 277 | 9.13E-01 | 1.00E+00 | 1.00E+00 | 2.22E-03 | 3.39E-03 | rs58688157 | 2.97E-11 |  |
| IBD | *CCDC101* | 277 | 2.32E-03 | 4.01E-05 | 1.10E-04 | 4.89E-03 | 7.37E-03 | rs4787482 | 1.22E-03 | √ |
| IBD | *CCDC116* | 151 | 9.58E-01 | 1.00E+00 | 1.00E+00 | 1.85E-02 | 2.79E-02 | rs141910407 | 6.70E-103 |  |
| IBD | *CCHCR1* | 151 | 2.18E-02 | 2.39E-01 | 1.11E-01 | 9.51E-04 | 1.43E-03 | rs3809822 | 1.29E-07 | √ |
| IBD | *CCNY* | 151 | 5.23E-01 | 9.76E-03 | 1.26E-04 | 1.00E+00 | 1.00E+00 | rs141910407 | 6.70E-103 |  |
| IBD | *CCR6* | 151 | 9.50E-01 | 1.00E+00 | 1.00E+00 | 2.99E-04 | 4.45E-04 | rs9273327 | 1.76E-100 |  |
| IBD | *CELSR3* | 151 | 8.44E-01 | 9.30E-01 | 1.00E+00 | 1.27E-02 | 1.90E-02 | rs9852014 | 2.26E-36 |  |
| IBD | *CFB* | 151 | 8.53E-01 | 8.69E-02 | 1.00E+00 | 3.08E-42 | 4.62E-42 | rs141910407 | 6.70E-103 |  |
| IBD | *CLIC1* | 151 | 1.25E-02 | 1.00E+00 | 1.00E+00 | 8.15E-31 | 1.22E-30 | rs45527431 | 5.68E-32 |  |
| IBD | *CLK2* | 151 | 5.58E-01 | 8.33E-03 | 1.13E-01 | 1.00E+00 | 1.00E+00 | rs8177429 | 1.32E-06 | √ |
| IBD | *CLN3* | 556 | 3.97E-01 | 1.99E-09 | 2.89E-09 | 2.23E-04 | 3.39E-04 | rs71603662 | 3.64E-07 | √ |
| IBD | *COASY* | 57 | 1.35E-04 | 2.12E-03 | 3.55E-04 | 1.00E+00 | 1.00E+00 | rs116298963 | 5.55E-54 |  |
| IBD | *CSF2* | 57 | 1.29E-01 | 2.96E-08 | 2.93E-02 | 2.58E-06 | 3.87E-06 | rs141910407 | 6.70E-103 |  |
| IBD | *CSF3* | 57 | 5.87E-02 | 5.30E-11 | 2.13E-07 | 3.63E-11 | 5.45E-11 | rs114828403 | 6.82E-72 |  |
| IBD | *CSNK2B* | 57 | 1.53E-01 | 1.22E-02 | 3.28E-01 | 2.51E-30 | 3.76E-30 | rs141910407 | 6.70E-103 |  |
| IBD | *CTB-134H23.1* | 57 | 5.03E-02 | 4.89E-06 | 4.54E-06 | 1.73E-02 | 2.59E-02 | rs115484360 | 2.93E-102 |  |
| IBD | *CTD-2330K9.3* | 57 | 3.39E-02 | 4.46E-08 | 1.47E-01 | 2.14E-03 | 3.20E-03 | rs2573219 | 1.13E-42 |  |
| IBD | *CYLD* | 57 | 2.19E-02 | 1.00E+00 | 1.00E+00 | 3.17E-29 | 4.75E-29 | rs141910407 | 6.70E-103 |  |
| IBD | *CYP21A2* | 167 | 9.86E-01 | 1.00E+00 | 1.00E+00 | 6.48E-43 | 9.73E-43 | rs147027375 | 1.55E-96 |  |
| IBD | *DAG1* | 167 | 3.89E-12 | 7.65E-12 | 1.53E-09 | 4.27E-20 | 6.41E-20 | rs141910407 | 6.70E-103 |  |
| IBD | *DCTN4* | 167 | 2.88E-01 | 1.00E+00 | 1.00E+00 | 1.43E-02 | 2.20E-02 | rs141910407 | 6.70E-103 |  |
| IBD | *DDAH2* | 8 | 4.13E-03 | 1.00E+00 | 1.00E+00 | 1.63E-29 | 2.45E-29 | rs62074179 | 1.46E-04 | √ |
| IBD | *DDX39B* | 7 | 2.13E-01 | 1.00E+00 | 1.00E+00 | 1.23E-07 | 1.85E-07 | rs2736332 | 4.83E-18 |  |
| IBD | *DGKD* | 85 | 4.46E-03 | 5.43E-02 | 1.00E+00 | 1.03E-15 | 1.54E-15 | rs6671847 | 6.64E-12 |  |
| IBD | *DHX58* | 52 | 9.54E-01 | 4.32E-03 | 5.03E-03 | 1.00E+00 | 1.00E+00 | rs35251378 | 3.61E-13 |  |
| IBD | *DOM3Z* | 36 | 6.87E-01 | 1.00E+00 | 1.00E+00 | 2.08E-41 | 3.12E-41 | rs141910407 | 6.70E-103 |  |
| IBD | *DYRK3* | 33 | 4.78E-01 | 1.00E+00 | 1.00E+00 | 3.91E-18 | 5.86E-18 | rs35000415 | 1.86E-45 |  |
| IBD | *EGFL8* | 33 | 5.68E-01 | 1.00E+00 | 1.00E+00 | 7.57E-50 | 1.13E-49 | rs1143679 | 5.03E-48 |  |
| IBD | *EGR2* | 33 | 8.42E-01 | 1.00E+00 | 1.00E+00 | 2.02E-02 | 3.08E-02 | rs73934025 | 5.39E-06 | √ |
| IBD | *EHMT2* | 33 | 9.80E-01 | 1.04E-06 | 1.36E-04 | 4.87E-43 | 7.30E-43 | rs115236446 | 1.15E-47 |  |
| IBD | *EIF3C* | 253 | 5.82E-07 | 6.62E-05 | 1.15E-03 | 2.61E-01 | 3.91E-01 | rs36010116 | 9.48E-05 | √ |
| IBD | *EIF3CL* | 253 | 1.86E-01 | 5.06E-10 | 1.00E+00 | 2.92E-04 | 4.39E-04 | rs141910407 | 6.70E-103 |  |
| IBD | *ENTPD7* | 253 | 4.95E-01 | 1.00E+00 | 1.00E+00 | 3.91E-10 | 5.86E-10 | rs141910407 | 6.70E-103 |  |
| IBD | *ERBB2* | 253 | 4.84E-01 | 1.46E-09 | 1.53E-08 | 1.31E-15 | 1.96E-15 | rs6869426 | 1.17E-03 | √ |
| IBD | *ERRFI1* | 474 | 3.10E-01 | 1.00E+00 | 1.00E+00 | 3.77E-03 | 5.68E-03 | rs112679482 | 8.29E-06 | √ |
| IBD | *FAIM3* | 129 | 1.38E-01 | 1.00E+00 | 1.00E+00 | 1.46E-06 | 2.19E-06 | rs9852014 | 2.26E-36 |  |
| IBD | *FAM189B* | 448 | 4.36E-01 | 1.52E-03 | 1.00E+00 | 1.00E+00 | 1.00E+00 | rs45527431 | 5.68E-32 |  |
| IBD | *FAM212A* | 118 | 1.22E-01 | 2.02E-08 | 1.51E-07 | 8.63E-04 | 1.30E-03 | rs147027375 | 1.55E-96 |  |
| IBD | *FAM98B* | 8 | 3.80E-01 | 1.46E-07 | 1.00E+00 | 1.00E+00 | 1.00E+00 | rs115619714 | 1.26E-98 |  |
| IBD | *FCGR2A* | 8 | 4.35E-01 | 1.60E-05 | 2.00E-05 | 5.09E-08 | 7.63E-08 | rs35400317 | 5.89E-32 |  |
| IBD | *FCGR2B* | 8 | 7.11E-01 | 1.00E+00 | 1.00E+00 | 2.41E-07 | 3.61E-07 | rs13197176 | 1.05E-34 |  |
| IBD | *FDPS* | 4 | 2.28E-01 | 1.20E-02 | 1.53E-01 | 1.00E+00 | 1.00E+00 | rs13195401 | 3.45E-31 |  |
| IBD | *FGFR1OP* | 4 | 4.41E-01 | 2.31E-05 | 1.00E+00 | 9.82E-05 | 1.50E-04 | rs45527431 | 5.68E-32 |  |
| IBD | *FKBPL* | 4 | 1.48E-01 | 1.00E+00 | 1.00E+00 | 2.11E-50 | 3.16E-50 | rs45527431 | 5.68E-32 |  |
| IBD | *GBA* | 4 | 1.78E-01 | 7.23E-03 | 8.49E-02 | 1.00E+00 | 1.00E+00 | rs13195401 | 3.45E-31 |  |
| IBD | *GHDC* | 4 | 1.31E-02 | 2.55E-03 | 4.79E-03 | 1.00E+00 | 1.00E+00 | rs45527431 | 5.68E-32 |  |
| IBD | *GMEB2* | 4 | 6.67E-07 | 1.00E+00 | 1.00E+00 | 1.11E-11 | 1.66E-11 | rs45527431 | 5.68E-32 |  |
| IBD | *GMPPB* | 292 | 1.28E-01 | 4.03E-09 | 1.06E-07 | 1.10E-22 | 1.64E-22 | rs140365013 | 8.94E-33 |  |
| IBD | *GOT1* | 292 | 1.01E-01 | 1.00E+00 | 1.00E+00 | 1.68E-19 | 2.52E-19 | rs140365013 | 8.94E-33 |  |
| IBD | *GPANK1* | 292 | 1.39E-02 | 1.00E+00 | 1.00E+00 | 1.40E-29 | 2.11E-29 | rs13197176 | 1.05E-34 |  |
| IBD | *GPR25* | 292 | 3.25E-02 | 1.00E+00 | 1.00E+00 | 4.12E-08 | 6.18E-08 | rs13197176 | 1.05E-34 |  |
| IBD | *GPSM3* | 57 | 7.46E-01 | 1.00E+00 | 1.00E+00 | 6.27E-51 | 9.41E-51 | rs13197176 | 1.05E-34 |  |
| IBD | *GPX1* | 44 | 3.85E-08 | 2.00E-06 | 1.10E-05 | 1.46E-18 | 2.18E-18 | rs13197176 | 1.05E-34 |  |
| IBD | *GRB7* | 44 | 6.44E-01 | 2.64E-10 | 3.36E-09 | 2.72E-15 | 4.08E-15 | rs13197176 | 1.05E-34 |  |
| IBD | *GS1-410F4.2* | 44 | 3.53E-01 | 1.00E+00 | 1.00E+00 | 1.54E-11 | 2.30E-11 | rs13195401 | 3.45E-31 |  |
| IBD | *GSDMB* | 50 | 8.67E-01 | 4.25E-08 | 1.12E-05 | 1.24E-17 | 1.86E-17 | rs45527431 | 5.68E-32 |  |
| IBD | *HCG22* | 50 | 5.24E-02 | 1.00E+00 | 1.00E+00 | 4.01E-03 | 6.27E-03 | rs45527431 | 5.68E-32 |  |
| IBD | *HCG27* | 50 | 1.99E-01 | 1.00E+00 | 1.00E+00 | 2.00E-03 | 3.08E-03 | rs45527431 | 5.68E-32 |  |
| IBD | *HCN3* | 50 | 4.42E-01 | 1.96E-03 | 2.93E-03 | 1.00E+00 | 1.00E+00 | rs45527431 | 5.68E-32 |  |
| IBD | *HIC2* | 50 | 8.07E-12 | 1.00E+00 | 1.00E+00 | 1.00E+00 | 1.00E+00 | rs45527431 | 5.68E-32 |  |
| IBD | *HLA-B* | 50 | 7.52E-01 | 1.00E+00 | 1.00E+00 | 2.74E-05 | 4.39E-05 | rs45527431 | 5.68E-32 |  |
| IBD | *HLA-C* | 50 | 6.07E-01 | 1.00E+00 | 1.00E+00 | 3.49E-05 | 5.02E-05 | rs45527431 | 5.68E-32 |  |
| IBD | *HLA-DOB* | 50 | 6.03E-03 | 7.94E-02 | 1.00E+00 | 5.80E-04 | 8.59E-04 | rs140365013 | 8.94E-33 |  |
| IBD | *HLA-DQA1* | 96 | 1.43E-04 | 5.14E-28 | 4.88E-21 | 4.88E-37 | 7.32E-37 | rs140365013 | 8.94E-33 |  |
| IBD | *HLA-DQA2* | 96 | 2.31E-05 | 3.82E-07 | 1.00E+00 | 2.00E-09 | 2.99E-09 | rs13197176 | 1.05E-34 |  |
| IBD | *HLA-DQB1* | 96 | 2.93E-05 | 3.60E-16 | 1.93E-10 | 8.78E-37 | 1.32E-36 | rs13197176 | 1.05E-34 |  |
| IBD | *HLA-DQB2* | 96 | 3.91E-04 | 2.23E-07 | 1.16E-03 | 2.47E-07 | 3.70E-07 | rs13197176 | 1.05E-34 |  |
| IBD | *HLA-DRA* | 88 | 3.57E-01 | 1.00E+00 | 1.00E+00 | 4.52E-07 | 6.78E-07 | rs13197176 | 1.05E-34 |  |
| IBD | *HLA-DRB1* | 76 | 1.50E-06 | 2.02E-22 | 1.17E-14 | 3.07E-37 | 4.60E-37 | rs13195401 | 3.45E-31 |  |
| IBD | *HLA-DRB5* | 129 | 1.08E-05 | 1.33E-24 | 2.59E-15 | 2.52E-37 | 3.79E-37 | rs13195401 | 3.45E-31 |  |
| IBD | *HSPA1B* | 45 | 2.39E-01 | 8.00E-02 | 1.14E-02 | 1.00E+00 | 1.00E+00 | rs13195401 | 3.45E-31 |  |
| IBD | *HSPA6* | 354 | 5.71E-01 | 8.32E-09 | 1.95E-05 | 3.73E-08 | 5.59E-08 | rs45527431 | 5.68E-32 |  |
| IBD | *HSPB9* | 107 | 4.13E-01 | 4.61E-03 | 4.91E-03 | 1.00E+00 | 1.00E+00 | rs45527431 | 5.68E-32 |  |
| IBD | *ICOSLG* | 138 | 1.36E-01 | 6.39E-07 | 3.94E-06 | 4.34E-11 | 6.51E-11 | rs45527431 | 5.68E-32 |  |
| IBD | *IFNG* | 44 | 1.27E-01 | 1.00E+00 | 1.00E+00 | 1.97E-12 | 2.96E-12 | rs45527431 | 5.68E-32 |  |
| IBD | *IKZF3* | 165 | 5.46E-01 | 4.36E-18 | 1.19E-14 | 6.45E-17 | 9.68E-17 | rs13197176 | 1.05E-34 |  |
| IBD | *IL10* | 102 | 4.91E-03 | 9.96E-03 | 1.00E+00 | 5.97E-18 | 8.95E-18 | rs13197176 | 1.05E-34 |  |
| IBD | *IL12B* | 219 | 1.12E-02 | 9.32E-08 | 1.59E-06 | 5.95E-24 | 8.93E-24 | rs13197176 | 1.05E-34 |  |
| IBD | *IL12RB2* | 241 | 3.13E-05 | 2.08E-50 | 1.07E-04 | 6.28E-98 | 9.42E-98 | rs13195401 | 3.45E-31 |  |
| IBD | *IL19* | 155 | 6.52E-03 | 8.05E-02 | 1.00E+00 | 2.48E-17 | 3.72E-17 | rs13195401 | 3.45E-31 |  |
| IBD | *IL23R* | 155 | 1.36E-01 | 4.65E-47 | 1.23E-37 | 8.78E-97 | 1.32E-96 | rs45527431 | 5.68E-32 |  |
| IBD | *IL24* | 155 | 8.91E-02 | 5.64E-07 | 1.64E-06 | 1.63E-06 | 2.44E-06 | rs45527431 | 5.68E-32 |  |
| IBD | *IL26* | 155 | 2.89E-01 | 1.00E+00 | 1.00E+00 | 4.56E-12 | 6.85E-12 | rs45527431 | 5.68E-32 |  |
| IBD | *IL27* | 155 | 8.10E-03 | 1.10E-04 | 1.89E-04 | 1.36E-02 | 2.06E-02 | rs45527431 | 5.68E-32 |  |
| IBD | *INPP5D* | 155 | 4.91E-01 | 1.00E+00 | 1.00E+00 | 7.30E-15 | 1.10E-14 | rs140365013 | 8.94E-33 |  |
| IBD | *IP6K1* | 155 | 1.89E-01 | 5.16E-16 | 8.78E-12 | 4.03E-23 | 6.04E-23 | rs13197176 | 1.05E-34 |  |
| IBD | *IP6K2* | 155 | 1.42E-01 | 4.03E-05 | 1.00E+00 | 9.39E-03 | 1.41E-02 | rs13197176 | 1.05E-34 |  |
| IBD | *IQCH* | 398 | 9.10E-01 | 1.00E+00 | 1.00E+00 | 1.86E-06 | 2.79E-06 | rs116186974 | 1.59E-51 |  |
| IBD | *IRF1* | 398 | 5.55E-01 | 1.24E-08 | 1.28E-04 | 6.09E-14 | 9.13E-14 | rs116099232 | 2.94E-100 |  |
| IBD | *IRGM* | 348 | 8.44E-01 | 1.00E+00 | 1.00E+00 | 1.42E-02 | 2.15E-02 | rs3131383 | 6.60E-99 |  |
| IBD | *JAK2* | 463 | 8.14E-01 | 3.97E-14 | 8.13E-13 | 6.85E-16 | 1.03E-15 | rs9273327 | 1.76E-100 |  |
| IBD | *KAT2A* | 98 | 5.34E-01 | 3.15E-03 | 4.65E-03 | 1.00E+00 | 1.00E+00 | rs9273327 | 1.76E-100 |  |
| IBD | *KCNH4* | 302 | 1.16E-02 | 4.65E-04 | 2.46E-03 | 1.00E+00 | 1.00E+00 | rs9273327 | 1.76E-100 |  |
| IBD | *KIAA1841* | 27 | 3.17E-01 | 5.77E-01 | 1.00E+00 | 8.98E-15 | 1.35E-14 | rs9273327 | 1.76E-100 |  |
| IBD | *KIF21B* | 245 | 4.23E-01 | 1.00E+00 | 1.00E+00 | 1.88E-08 | 2.81E-08 | rs9273327 | 1.76E-100 |  |
| IBD | *KRTCAP2* | 273 | 1.40E-01 | 1.69E-02 | 1.00E+00 | 1.00E+00 | 1.00E+00 | rs9273327 | 1.76E-100 |  |
| IBD | *LIF* | 273 | 1.31E-01 | 2.71E-02 | 1.00E+00 | 1.00E+00 | 1.00E+00 | rs1269852 | 6.99E-101 |  |
| IBD | *LIME1* | 21 | 2.10E-01 | 1.00E+00 | 1.00E+00 | 1.52E-11 | 2.28E-11 | rs9273327 | 1.76E-100 |  |
| IBD | *LINC00481* | 21 | 2.51E-01 | 1.00E+00 | 1.00E+00 | 8.26E-04 | 1.24E-03 | rs116300399 | 7.35E-101 |  |
| IBD | *LRP3* | 21 | 1.69E-01 | 1.00E+00 | 1.00E+00 | 8.49E-04 | 1.27E-03 | rs9273327 | 1.76E-100 |  |
| IBD | *LRRC32* | 21 | 1.29E-01 | 3.80E-03 | 7.45E-02 | 3.81E-18 | 5.71E-18 | rs141910407 | 6.70E-103 |  |
| IBD | *LRRK2* | 61 | 3.64E-01 | 2.11E-08 | 2.73E-08 | 8.66E-05 | 1.32E-04 | rs1150757 | 2.08E-101 |  |
| IBD | *LSM2* | 61 | 4.31E-02 | 1.66E-04 | 1.85E-04 | 5.18E-30 | 7.78E-30 | rs141910407 | 6.70E-103 |  |
| IBD | *LST1* | 61 | 1.69E-01 | 8.61E-01 | 1.00E+00 | 7.69E-26 | 1.15E-25 | rs115387234 | 3.50E-49 |  |
| IBD | *LTA* | 61 | 1.89E-01 | 5.97E-02 | 1.00E+00 | 5.66E-25 | 8.48E-25 | rs114416651 | 1.34E-49 |  |
| IBD | *LTB* | 61 | 3.63E-01 | 7.22E-03 | 1.00E+00 | 6.84E-27 | 1.03E-26 | rs45527431 | 5.68E-32 |  |
| IBD | *LY6G5B* | 61 | 7.38E-02 | 1.05E-02 | 1.00E+00 | 7.18E-31 | 1.08E-30 | rs58688157 | 2.97E-11 |  |
| IBD | *LY6G5C* | 61 | 5.91E-01 | 4.44E-02 | 1.00E+00 | 7.57E-31 | 1.14E-30 | rs115575857 | 2.42E-98 |  |
| IBD | *MAPKAPK2* | 58 | 1.21E-02 | 1.00E+00 | 1.00E+00 | 3.04E-18 | 4.56E-18 | rs141910407 | 6.70E-103 |  |
| IBD | *MED24* | 58 | 7.60E-01 | 8.91E-07 | 1.65E-03 | 1.88E-10 | 2.82E-10 | rs141910407 | 6.70E-103 |  |
| IBD | *MICA* | 58 | 6.89E-01 | 1.00E+00 | 1.00E+00 | 8.05E-05 | 1.19E-04 | rs141910407 | 6.70E-103 |  |
| IBD | *MICB* | 58 | 9.24E-02 | 5.14E-03 | 1.00E+00 | 6.56E-05 | 1.00E-04 | rs6671847 | 6.64E-12 |  |
| IBD | *MIEN1* | 58 | 6.93E-01 | 3.91E-04 | 1.00E+00 | 2.12E-15 | 3.18E-15 | rs35251378 | 3.61E-13 |  |
| IBD | *MIER1* | 58 | 7.89E-01 | 1.68E-08 | 1.00E+00 | 1.31E-24 | 1.97E-24 | rs35251378 | 3.61E-13 |  |
| IBD | *MLX* | 147 | 3.00E-03 | 8.61E-01 | 9.38E-04 | 1.00E+00 | 1.00E+00 | rs35251378 | 3.61E-13 |  |
| IBD | *MON1A* | 167 | 1.29E-01 | 3.36E-08 | 1.00E+00 | 5.04E-03 | 7.55E-03 | rs35251378 | 3.61E-13 |  |
| IBD | *MSH5* | 208 | 1.28E-02 | 4.95E-01 | 1.00E+00 | 7.91E-32 | 1.19E-31 | rs9852014 | 2.26E-36 |  |
| IBD | *MSH5-SAPCD1* | 589 | 2.09E-02 | 1.00E+00 | 1.00E+00 | 3.14E-31 | 4.71E-31 | rs112679482 | 8.29E-06 | √ |
| IBD | *MST1* | 184 | 3.47E-01 | 8.46E-08 | 1.12E-01 | 2.60E-23 | 3.89E-23 | rs276819 | 5.81E-05 | √ |
| IBD | *MST1R* | 289 | 3.42E-06 | 3.02E-07 | 1.00E+00 | 1.17E-03 | 1.76E-03 | rs35000415 | 1.86E-45 |  |
| IBD | *MTX1* | 289 | 7.31E-01 | 6.19E-04 | 1.00E+00 | 1.00E+00 | 1.00E+00 | rs58688157 | 2.97E-11 |  |
| IBD | *MUC1* | 8 | 4.04E-01 | 2.84E-03 | 1.00E+00 | 1.00E+00 | 1.00E+00 | rs13332649 | 5.43E-17 |  |
| IBD | *MUC19* | 8 | 4.05E-01 | 1.00E+00 | 1.00E+00 | 2.18E-04 | 3.70E-04 | rs1143679 | 5.03E-48 |  |
| IBD | *MX2* | 8 | 8.37E-01 | 1.00E+00 | 1.00E+00 | 3.89E-08 | 5.84E-08 | rs1143679 | 5.03E-48 |  |
| IBD | *NCKIPSD* | 8 | 6.81E-02 | 8.25E-10 | 9.08E-02 | 1.12E-02 | 1.68E-02 | rs1143679 | 5.03E-48 |  |
| IBD | *NCR3* | 8 | 3.28E-01 | 1.00E+00 | 1.00E+00 | 2.72E-26 | 4.08E-26 | rs11264305 | 1.96E-04 | √ |
| IBD | *NEU1* | 39 | 3.16E-01 | 2.51E-02 | 1.00E+00 | 1.51E-42 | 2.27E-42 | rs4787482 | 1.22E-03 | √ |
| IBD | *NFKBIL1* | 223 | 1.04E-01 | 4.10E-02 | 1.00E+00 | 5.46E-05 | 8.15E-05 | rs115591082 | 7.33E-55 |  |
| IBD | *NICN1* | 108 | 1.54E-16 | 1.81E-10 | 8.25E-08 | 8.66E-19 | 1.30E-18 | rs17849501 | 1.81E-59 |  |
| IBD | *NKD1* | 682 | 4.61E-10 | 1.07E-14 | 5.26E-05 | 4.88E-33 | 7.32E-33 | rs17849501 | 1.81E-59 |  |
| IBD | *NKIRAS2* | 682 | 4.97E-01 | 6.38E-04 | 4.06E-04 | 1.00E+00 | 1.00E+00 | rs13191227 | 1.41E-32 |  |
| IBD | *NOD2* | 682 | 9.78E-06 | 1.00E+00 | 1.00E+00 | 7.44E-31 | 1.12E-30 | rs115619714 | 1.26E-98 |  |
| IBD | *NOTCH4* | 682 | 9.65E-01 | 1.00E+00 | 1.00E+00 | 1.51E-50 | 2.27E-50 | rs41266779 | 1.54E-21 |  |
| IBD | *NPIPL1* | 682 | 3.36E-01 | 2.81E-09 | 8.07E-09 | 2.51E-04 | 3.76E-04 | rs58688157 | 2.97E-11 |  |
| IBD | *ORMDL3* | 682 | 6.99E-01 | 3.08E-20 | 3.35E-13 | 1.88E-17 | 2.81E-17 | rs141910407 | 6.70E-103 |  |
| IBD | *OTUD3* | 682 | 7.92E-01 | 3.55E-17 | 2.07E-11 | 1.44E-07 | 2.17E-07 | rs141910407 | 6.70E-103 |  |
| IBD | *P4HA2* | 682 | 5.67E-01 | 1.14E-02 | 5.11E-01 | 4.13E-13 | 6.20E-13 | rs141910407 | 6.70E-103 |  |
| IBD | *PBX2* | 75 | 8.28E-01 | 1.00E+00 | 1.00E+00 | 2.19E-50 | 3.29E-50 | rs141910407 | 6.70E-103 |  |
| IBD | *PDGFB* | 75 | 3.20E-01 | 1.00E+00 | 1.00E+00 | 2.10E-11 | 3.14E-11 | rs141910407 | 6.70E-103 |  |
| IBD | *PDLIM4* | 75 | 4.12E-01 | 2.62E-03 | 3.19E-01 | 2.92E-12 | 4.38E-12 | rs141910407 | 6.70E-103 |  |
| IBD | *PEX13* | 47 | 4.12E-01 | 1.76E-12 | 3.29E-11 | 2.53E-15 | 3.79E-15 | rs9852014 | 2.26E-36 |  |
| IBD | *PFKL* | 30 | 5.80E-02 | 1.00E+00 | 1.00E+00 | 1.09E-07 | 1.64E-07 | rs141597299 | 1.44E-100 |  |
| IBD | *PGAP3* | 30 | 3.50E-02 | 5.95E-03 | 1.00E+00 | 3.63E-15 | 5.44E-15 | rs115484360 | 2.93E-102 |  |
| IBD | *PKLR* | 131 | 8.41E-01 | 4.01E-02 | 3.72E-01 | 1.00E+00 | 1.00E+00 | rs58688157 | 2.97E-11 |  |
| IBD | *POU5F1* | 131 | 5.48E-02 | 1.00E+00 | 1.00E+00 | 2.24E-04 | 3.39E-04 | rs35251378 | 3.61E-13 |  |
| IBD | *PPIF* | 121 | 7.67E-01 | 1.00E+00 | 2.39E-03 | 1.00E+00 | 1.00E+00 | rs141910407 | 6.70E-103 |  |
| IBD | *PPIL2* | 316 | 9.67E-01 | 1.00E+00 | 1.00E+00 | 1.45E-02 | 2.20E-02 | rs141910407 | 6.70E-103 |  |
| IBD | *PPT2* | 316 | 8.21E-01 | 1.00E+00 | 1.00E+00 | 8.60E-50 | 1.29E-49 | rs147027375 | 1.55E-96 |  |
| IBD | *PRRC2A* | 316 | 5.88E-02 | 3.70E-03 | 1.00E+00 | 1.11E-30 | 1.66E-30 | rs4274624 | 9.73E-66 |  |
| IBD | *PRRT1* | 316 | 8.96E-02 | 1.00E+00 | 1.00E+00 | 6.20E-49 | 9.29E-49 | rs17849501 | 1.81E-59 |  |
| IBD | *PSMB8* | 316 | 1.66E-02 | 6.72E-02 | 1.00E+00 | 3.26E-04 | 4.83E-04 | rs141910407 | 6.70E-103 |  |
| IBD | *PSMB9* | 316 | 2.45E-03 | 2.01E-08 | 1.00E+00 | 3.05E-05 | 4.39E-05 | rs141910407 | 6.70E-103 |  |
| IBD | *PSMD3* | 119 | 5.03E-01 | 8.67E-19 | 9.04E-22 | 4.88E-18 | 7.32E-18 | rs115484360 | 2.93E-102 |  |
| IBD | *PSMG1* | 119 | 4.34E-03 | 1.00E+00 | 1.00E+00 | 9.25E-14 | 1.39E-13 | rs116725083 | 1.78E-36 |  |
| IBD | *PSORS1C1* | 623 | 4.53E-02 | 1.00E+00 | 1.00E+00 | 1.33E-02 | 2.08E-02 | rs17849501 | 1.81E-59 |  |
| IBD | *PTPN2* | 623 | 3.32E-01 | 1.48E-06 | 5.29E-02 | 1.03E-01 | 1.56E-01 | rs141910407 | 6.70E-103 |  |
| IBD | *PTRF* | 623 | 3.22E-03 | 1.00E+00 | 1.00E+00 | 8.12E-05 | 1.32E-04 | rs67981811 | 9.96E-35 |  |
| IBD | *PUS10* | 33 | 2.80E-01 | 2.44E-13 | 1.35E-10 | 1.70E-15 | 2.54E-15 | rs115263146 | 1.49E-02 | √ |
| IBD | *PWP2* | 33 | 6.66E-03 | 9.07E-07 | 3.19E-06 | 2.63E-11 | 3.95E-11 | rs112679482 | 8.29E-06 | √ |
| IBD | *RAB5C* | 33 | 3.54E-01 | 1.68E-03 | 4.37E-03 | 1.00E+00 | 1.00E+00 | rs141910407 | 6.70E-103 |  |
| IBD | *RAD50* | 33 | 5.24E-01 | 1.43E-01 | 1.00E+00 | 9.66E-13 | 1.45E-12 | rs6927768 | 1.09E-04 | √ |
| IBD | *RAPGEF6* | 41 | 1.23E-02 | 1.00E+00 | 1.00E+00 | 4.51E-02 | 6.77E-02 | rs35251378 | 3.61E-13 |  |
| IBD | *RBM22* | 41 | 7.88E-01 | 1.00E+00 | 1.00E+00 | 3.83E-02 | 5.75E-02 | rs1078324 | 7.11E-20 |  |
| IBD | *RBM6* | 153 | 1.08E-01 | 4.55E-04 | 6.36E-03 | 5.36E-02 | 8.04E-02 | rs114720630 | 6.72E-72 |  |
| IBD | *RDBP* | 153 | 2.73E-01 | 1.00E+00 | 1.00E+00 | 6.84E-42 | 1.03E-41 | rs115387042 | 1.72E-37 |  |
| IBD | *REL* | 212 | 5.39E-01 | 9.84E-11 | 5.28E-07 | 1.89E-15 | 2.84E-15 | rs116298963 | 5.55E-54 |  |
| IBD | *RHOA* | 212 | 1.53E-14 | 3.59E-08 | 1.01E-04 | 8.97E-19 | 1.35E-18 | rs58688157 | 2.97E-11 |  |
| IBD | *RIMBP3B* | 154 | 1.18E-12 | 1.00E+00 | 1.00E+00 | 1.00E+00 | 1.00E+00 | rs58688157 | 2.97E-11 |  |
| IBD | *RIMBP3C* | 154 | 1.21E-01 | 1.00E+00 | 1.00E+00 | 3.45E-03 | 5.20E-03 | rs13202295 | 1.12E-34 |  |
| IBD | *RLN1* | 270 | 2.04E-01 | 4.49E-02 | 1.00E+00 | 4.06E-07 | 6.08E-07 | rs114629349 | 1.70E-98 |  |
| IBD | *RLN2* | 270 | 2.19E-01 | 1.00E+00 | 1.00E+00 | 3.63E-05 | 5.64E-05 | rs11956160 | 6.84E-04 | √ |
| IBD | *RNASET2* | 204 | 8.24E-01 | 4.05E-01 | 1.00E+00 | 2.48E-04 | 3.70E-04 | rs3747093 | 2.88E-14 |  |
| IBD | *RNF123* | 204 | 1.19E-02 | 2.40E-13 | 1.14E-11 | 6.38E-23 | 9.57E-23 | rs141910407 | 6.70E-103 |  |
| IBD | *RNF5* | 146 | 9.85E-01 | 1.00E+00 | 1.00E+00 | 3.09E-50 | 4.64E-50 | rs10912578 | 1.65E-15 |  |
| IBD | *RP11-1348G14.2* | 146 | 2.02E-19 | 3.39E-05 | 1.20E-04 | 2.93E-01 | 4.41E-01 | rs141910407 | 6.70E-103 |  |
| IBD | *RP11-168O16.1* | 146 | 3.49E-01 | 1.00E+00 | 1.00E+00 | 1.70E-05 | 2.51E-05 | rs141910407 | 6.70E-103 |  |
| IBD | *RP11-29H23.1* | 146 | 6.50E-09 | 1.00E+00 | 1.00E+00 | 1.00E+00 | 1.00E+00 | rs13217620 | 2.21E-34 |  |
| IBD | *RP11-401P9.4* | 146 | 1.83E-09 | 3.13E-11 | 6.84E-02 | 3.90E-33 | 5.84E-33 | rs276819 | 5.81E-05 | √ |
| IBD | *RP11-476D10.1* | 129 | 9.21E-01 | 1.00E+00 | 1.00E+00 | 5.75E-03 | 8.62E-03 | rs9273327 | 1.76E-100 |  |
| IBD | *RP11-514O12.4* | 64 | 9.56E-01 | 1.00E+00 | 1.00E+00 | 2.84E-04 | 4.26E-04 | rs9273327 | 1.76E-100 |  |
| IBD | *RP4-697K14.7* | 32 | 1.76E-05 | 6.53E-12 | 4.94E-11 | 1.28E-06 | 1.92E-06 | rs112679482 | 8.29E-06 | √ |
| IBD | *RPL3* | 482 | 3.19E-01 | 1.00E+00 | 1.00E+00 | 2.56E-11 | 3.84E-11 | rs147027375 | 1.55E-96 |  |
| IBD | *RPL37* | 482 | 3.12E-11 | 1.00E+00 | 1.00E+00 | 1.00E+00 | 1.00E+00 | rs58688157 | 2.97E-11 |  |
| IBD | *RPS6KA2* | 482 | 9.63E-01 | 1.00E+00 | 1.00E+00 | 1.21E-02 | 1.82E-02 | rs71603662 | 3.64E-07 | √ |
| IBD | *RTEL1* | 81 | 3.10E-02 | 1.17E-02 | 1.00E+00 | 1.77E-11 | 2.65E-11 | rs1143679 | 5.03E-48 |  |
| IBD | *RTEL1* | 206 | 3.10E-02 | 1.17E-02 | 1.00E+00 | 1.77E-11 | 2.65E-11 | rs58688157 | 2.97E-11 |  |
| IBD | *RTEL1* | 284 | 1.54E-02 | 1.07E-01 | 1.00E+00 | 4.94E-12 | 7.41E-12 | rs35251378 | 3.61E-13 |  |
| IBD | *RTEL1* | 230 | 1.54E-02 | 1.07E-01 | 1.00E+00 | 4.94E-12 | 7.41E-12 | rs141910407 | 6.70E-103 |  |
| IBD | *SAPCD1* | 62 | 1.82E-01 | 1.00E+00 | 1.00E+00 | 1.95E-30 | 2.93E-30 | rs114720630 | 6.72E-72 |  |
| IBD | *SCAMP3* | 89 | 9.74E-01 | 2.47E-02 | 4.43E-01 | 1.00E+00 | 1.00E+00 | rs3747093 | 2.88E-14 |  |
| IBD | *SDF2L1* | 89 | 8.78E-01 | 1.00E+00 | 1.00E+00 | 2.92E-02 | 4.40E-02 | rs115575857 | 2.42E-98 |  |
| IBD | *SERBP1* | 113 | 3.40E-01 | 1.00E+00 | 1.00E+00 | 1.79E-03 | 2.75E-03 | rs141910407 | 6.70E-103 |  |
| IBD | *SKIV2L* | 453 | 8.49E-01 | 4.55E-01 | 1.00E+00 | 1.15E-42 | 1.73E-42 | rs58688157 | 2.97E-11 |  |
| IBD | *SLC22A4* | 453 | 8.54E-01 | 1.08E-05 | 1.00E+00 | 4.47E-13 | 6.70E-13 | rs67981811 | 9.96E-35 |  |
| IBD | *SLC22A5* | 453 | 4.33E-02 | 3.06E-15 | 2.08E-11 | 4.15E-14 | 6.23E-14 | rs2736332 | 4.83E-18 |  |
| IBD | *SLC25A28* | 453 | 3.26E-01 | 1.00E+00 | 1.00E+00 | 2.82E-21 | 4.23E-21 | rs58721818 | 3.38E-18 |  |
| IBD | *SLC26A6* | 453 | 8.10E-01 | 1.00E+00 | 1.00E+00 | 2.70E-02 | 4.05E-02 | rs12575883 | 7.01E-04 | √ |
| IBD | *SLC2A13* | 453 | 9.52E-01 | 3.36E-01 | 1.00E+00 | 3.97E-02 | 5.95E-02 | rs58721818 | 3.38E-18 |  |
| IBD | *SLC2A4RG* | 453 | 3.28E-01 | 1.00E+00 | 1.00E+00 | 7.57E-12 | 1.13E-11 | rs58721818 | 3.38E-18 |  |
| IBD | *SLC35D1* | 453 | 5.14E-02 | 4.42E-35 | 3.12E-33 | 4.05E-25 | 6.08E-25 | rs58721818 | 3.38E-18 |  |
| IBD | *SMAD3* | 19 | 9.91E-01 | 1.00E+00 | 1.00E+00 | 3.59E-06 | 5.39E-06 | rs1143679 | 5.03E-48 |  |
| IBD | *SNX20* | 131 | 7.43E-10 | 9.14E-13 | 5.67E-07 | 2.80E-33 | 4.20E-33 | rs1143679 | 5.03E-48 |  |
| IBD | *SRMS* | 131 | 5.80E-02 | 1.70E-06 | 1.00E+00 | 1.77E-06 | 2.65E-06 | rs45527431 | 5.68E-32 |  |
| IBD | *STARD3* | 131 | 2.66E-01 | 1.00E+00 | 1.00E+00 | 2.01E-13 | 3.01E-13 | rs17849501 | 1.81E-59 |  |
| IBD | *STAT3* | 41 | 4.60E-02 | 2.94E-02 | 1.00E+00 | 3.99E-05 | 6.27E-05 | rs115387042 | 1.72E-37 |  |
| IBD | *STAT5A* | 73 | 3.16E-01 | 1.00E+00 | 1.00E+00 | 1.01E-04 | 1.57E-04 | rs114720630 | 6.72E-72 |  |
| IBD | *STAT5B* | 127 | 2.62E-01 | 2.54E-08 | 4.55E-02 | 1.36E-04 | 2.07E-04 | rs115575857 | 2.42E-98 |  |
| IBD | *STK19* | 35 | 6.47E-01 | 1.42E-05 | 2.33E-04 | 2.90E-42 | 4.36E-42 | rs141910407 | 6.70E-103 |  |
| IBD | *STMN3* | 35 | 6.03E-04 | 1.48E-13 | 4.10E-13 | 1.58E-12 | 2.38E-12 | rs115387042 | 1.72E-37 |  |
| IBD | *SULT1A1* | 35 | 1.25E-09 | 4.36E-05 | 9.59E-05 | 6.28E-02 | 9.44E-02 | rs3747093 | 2.88E-14 |  |
| IBD | *SULT1A2* | 35 | 2.94E-06 | 4.26E-05 | 1.11E-04 | 3.36E-02 | 5.05E-02 | rs58688157 | 2.97E-11 |  |
| IBD | *SYNGR1* | 8 | 7.90E-01 | 1.00E+00 | 1.00E+00 | 1.50E-11 | 2.26E-11 | rs141910407 | 6.70E-103 |  |
| IBD | *TAB1* | 8 | 7.05E-01 | 1.00E+00 | 1.00E+00 | 1.71E-07 | 2.56E-07 | rs2736332 | 4.83E-18 |  |
| IBD | *TAP1* | 276 | 1.56E-02 | 1.00E+00 | 1.00E+00 | 2.31E-03 | 3.45E-03 | rs115575857 | 2.42E-98 |  |
| IBD | *TAP2* | 276 | 2.73E-02 | 1.00E+00 | 1.00E+00 | 3.92E-03 | 5.84E-03 | rs12729943 | 5.35E-04 | √ |
| IBD | *TCAP* | 276 | 9.11E-02 | 7.74E-03 | 1.00E+00 | 5.41E-14 | 8.11E-14 | rs28694840 | 5.07E-05 | √ |
| IBD | *TCF19* | 276 | 1.34E-01 | 1.00E+00 | 1.00E+00 | 4.38E-05 | 6.27E-05 | rs17849501 | 1.81E-59 |  |
| IBD | *TCP10L2* | 276 | 7.37E-01 | 4.30E-08 | 5.61E-08 | 7.83E-04 | 1.17E-03 | rs35000415 | 1.86E-45 |  |
| IBD | *TCTA* | 276 | 4.78E-26 | 1.39E-07 | 2.02E-03 | 3.98E-19 | 5.97E-19 | rs36010116 | 9.48E-05 | √ |
| IBD | *THBS3* | 276 | 4.44E-01 | 3.19E-03 | 1.00E+00 | 1.00E+00 | 1.00E+00 | rs13017709 | 2.95E-03 | √ |
| IBD | *TMCO4* | 276 | 7.46E-01 | 7.00E-09 | 1.71E-08 | 1.21E-06 | 1.82E-06 | rs141910407 | 6.70E-103 |  |
| IBD | *TMEM191C* | 274 | 1.23E-03 | 4.07E-08 | 1.56E-08 | 4.00E-04 | 6.02E-04 | rs116298963 | 5.55E-54 |  |
| IBD | *TMEM9* | 274 | 9.54E-01 | 1.00E+00 | 1.00E+00 | 2.79E-05 | 4.39E-05 | rs9273327 | 1.76E-100 |  |
| IBD | *TMPRSS3* | 274 | 9.67E-01 | 1.00E+00 | 1.00E+00 | 1.50E-11 | 2.25E-11 | rs9273327 | 1.76E-100 |  |
| IBD | *TNF* | 274 | 2.25E-01 | 3.63E-02 | 1.00E+00 | 3.85E-25 | 5.78E-25 | rs114720630 | 6.72E-72 |  |
| IBD | *TNFSF15* | 274 | 4.37E-02 | 8.38E-21 | 3.34E-20 | 2.84E-16 | 4.27E-16 | rs11579128 | 2.54E-05 | √ |
| IBD | *TNFSF8* | 274 | 4.68E-01 | 1.00E+00 | 1.00E+00 | 1.47E-11 | 2.21E-11 | rs114629349 | 1.70E-98 |  |
| IBD | *TNXB* | 274 | 6.88E-01 | 1.00E+00 | 1.00E+00 | 2.43E-49 | 3.64E-49 | rs3747093 | 2.88E-14 |  |
| IBD | *TRAIP* | 274 | 1.43E-01 | 4.06E-08 | 1.87E-07 | 1.18E-03 | 1.77E-03 | rs141910407 | 6.70E-103 |  |
| IBD | *TRAPPC10* | 21 | 8.80E-01 | 1.00E+00 | 1.00E+00 | 2.41E-10 | 3.62E-10 | rs58721818 | 3.38E-18 |  |
| IBD | *UBA7* | 60 | 2.32E-01 | 4.39E-07 | 3.74E-06 | 1.77E-03 | 2.65E-03 | rs10912578 | 1.65E-15 |  |
| IBD | *UBE2L3* | 60 | 9.67E-01 | 1.00E+00 | 1.00E+00 | 5.34E-03 | 8.03E-03 | rs6869426 | 1.17E-03 | √ |
| IBD | *UBLCP1* | 10 | 1.20E-01 | 1.00E+00 | 1.00E+00 | 1.72E-18 | 2.59E-18 | rs35000415 | 1.86E-45 |  |
| IBD | *USP4* | 10 | 9.92E-01 | 9.11E-05 | 4.32E-02 | 1.35E-18 | 2.03E-18 | rs141910407 | 6.70E-103 |  |
| IBD | *VARS* | 165 | 1.25E-01 | 6.26E-01 | 1.00E+00 | 1.87E-31 | 2.80E-31 | rs115466242 | 5.26E-41 |  |
| IBD | *VWA1* | 16 | 9.96E-08 | 1.00E+00 | 1.00E+00 | 1.00E+00 | 1.00E+00 | rs13195401 | 3.45E-31 |  |
| IBD | *VWA7* | 16 | 2.43E-02 | 1.00E+00 | 1.00E+00 | 3.94E-31 | 5.90E-31 | rs1143679 | 5.03E-48 |  |
| IBD | *WDR78* | 16 | 6.66E-02 | 1.00E+00 | 1.00E+00 | 1.82E-11 | 2.73E-11 | rs35000415 | 1.86E-45 |  |
| IBD | *WDR88* | 16 | 1.61E-01 | 1.23E-03 | 8.94E-03 | 8.48E-04 | 1.31E-03 | rs35251378 | 3.61E-13 |  |
| IBD | *XXbac-BPG246D15.9* | 16 | 1.81E-02 | 1.00E+00 | 1.00E+00 | 2.26E-04 | 3.39E-04 | rs3747093 | 2.88E-14 |  |
| IBD | *XXbac-BPG300A18.12* | 16 | 3.66E-01 | 1.00E+00 | 1.00E+00 | 8.86E-50 | 1.33E-49 | rs141910407 | 6.70E-103 |  |
| IBD | *XXbac-BPG32J3.22* | 16 | 4.82E-02 | 2.39E-03 | 1.00E+00 | 1.13E-30 | 1.70E-30 | rs114720630 | 6.72E-72 |  |
| IBD | *YDJC* | 16 | 9.07E-01 | 1.00E+00 | 1.00E+00 | 2.26E-02 | 3.41E-02 | rs141910407 | 6.70E-103 |  |
| IBD | *ZBTB12* | 144 | 6.65E-01 | 1.10E-04 | 1.00E+00 | 2.25E-43 | 3.38E-43 | rs114720630 | 6.72E-72 |  |
| IBD | *ZBTB40* | 144 | 1.74E-01 | 1.00E+00 | 1.00E+00 | 1.84E-02 | 2.76E-02 | rs9273327 | 1.76E-100 |  |
| IBD | *ZBTB46* | 144 | 7.36E-01 | 5.54E-01 | 1.00E+00 | 2.13E-11 | 3.19E-11 | rs9273327 | 1.76E-100 |  |
| IBD | *ZCCHC24* | 144 | 6.39E-01 | 2.88E-03 | 1.94E-03 | 1.00E+00 | 1.00E+00 | rs141910407 | 6.70E-103 |  |
| IBD | *ZGPAT* | 144 | 2.01E-01 | 1.00E+00 | 1.00E+00 | 2.41E-11 | 3.61E-11 | rs141910407 | 6.70E-103 |  |
| IBD | *ZNF300* | 144 | 7.48E-01 | 1.00E+00 | 1.00E+00 | 1.43E-02 | 2.19E-02 | rs3747093 | 2.88E-14 |  |
| IBD | *ZNF365* | 144 | 1.04E-02 | 1.00E+00 | 1.00E+00 | 7.31E-16 | 1.10E-15 | rs3747093 | 2.88E-14 |  |
| IBD | *ZNF385C* | 144 | 5.57E-01 | 1.36E-03 | 3.47E-03 | 1.00E+00 | 1.00E+00 | rs141910407 | 6.70E-103 |  |
| IBD | *ZPBP2* | 33 | 1.38E-01 | 8.11E-18 | 4.70E-06 | 1.87E-17 | 2.81E-17 | rs114720630 | 6.72E-72 |  |
| UC | *ABHD16A* | 24 | 7.40E-01 | 5.96E-05 | 1.20E-03 | 9.69E-34 | 1.45E-33 | rs6589115 | 4.27E-05 | √ |
| UC | *AC008697.1* | 145 | 1.40E-01 | 5.43E-06 | 8.08E-04 | 5.33E-12 | 7.99E-12 | rs35251378 | 3.61E-13 |  |
| UC | *AC016747.3* | 145 | 2.64E-01 | 3.55E-02 | 1.00E+00 | 1.00E+00 | 1.00E+00 | rs115387042 | 1.72E-37 |  |
| UC | *ADA* | 291 | 7.33E-01 | 1.58E-03 | 3.27E-03 | 1.00E+00 | 1.00E+00 | rs116725083 | 1.78E-36 |  |
| UC | *AGER* | 47 | 3.05E-01 | 2.86E-04 | 1.00E+00 | 4.76E-52 | 7.15E-52 | rs67981811 | 9.96E-35 |  |
| UC | *AGPAT1* | 47 | 6.28E-01 | 6.94E-02 | 1.00E+00 | 5.71E-55 | 8.57E-55 | rs13202295 | 1.12E-34 |  |
| UC | *AIF1* | 189 | 1.50E-01 | 3.39E-09 | 9.99E-04 | 6.89E-35 | 1.03E-34 | rs115891768 | 9.37E-35 |  |
| UC | *AIRE* | 136 | 4.55E-01 | 9.03E-03 | 1.86E-02 | 1.98E-03 | 2.97E-03 | rs116137698 | 1.46E-35 |  |
| UC | *AMIGO3* | 136 | 2.11E-01 | 4.46E-06 | 4.73E-05 | 1.39E-11 | 2.09E-11 | rs115350668 | 3.23E-41 |  |
| UC | *AMT* | 76 | 3.79E-10 | 2.78E-07 | 1.33E-05 | 1.15E-07 | 1.76E-07 | rs45527431 | 5.68E-32 |  |
| UC | *ANKMY1* | 76 | 1.87E-02 | 1.00E+00 | 1.00E+00 | 1.14E-06 | 1.71E-06 | rs115387042 | 1.72E-37 |  |
| UC | *AP001055.6* | 25 | 1.71E-02 | 1.00E+00 | 1.00E+00 | 1.50E-03 | 2.25E-03 | rs4803210 | 1.56E-05 | √ |
| UC | *AP001056.1* | 25 | 3.77E-02 | 2.54E-02 | 1.89E-02 | 1.75E-03 | 2.63E-03 | rs13202295 | 1.12E-34 |  |
| UC | *AP001058.3* | 25 | 5.48E-02 | 2.61E-03 | 6.35E-02 | 2.63E-03 | 3.95E-03 | rs4803210 | 1.56E-05 | √ |
| UC | *APEH* | 66 | 3.07E-01 | 5.97E-08 | 3.27E-05 | 9.65E-12 | 1.45E-11 | rs4803210 | 1.56E-05 | √ |
| UC | *APOBR* | 15 | 6.56E-01 | 5.49E-03 | 3.17E-03 | 1.00E+00 | 1.00E+00 | rs1143679 | 5.03E-48 |  |
| UC | *APOM* | 107 | 2.22E-01 | 1.66E-05 | 9.40E-03 | 1.12E-33 | 1.68E-33 | rs112679482 | 8.29E-06 | √ |
| UC | *ARFRP1* | 10 | 3.09E-01 | 1.00E+00 | 1.00E+00 | 6.56E-05 | 9.84E-05 | rs115387042 | 1.72E-37 |  |
| UC | *ATF6B* | 10 | 1.38E-01 | 4.05E-06 | 2.59E-04 | 9.16E-56 | 1.37E-55 | rs67981811 | 9.96E-35 |  |
| UC | *ATP6V1F* | 269 | 6.12E-01 | 2.30E-02 | 1.81E-02 | 1.00E+00 | 1.00E+00 | rs115387042 | 1.72E-37 |  |
| UC | *ATP6V1G2* | 510 | 7.69E-01 | 1.00E+00 | 1.00E+00 | 5.15E-09 | 7.73E-09 | rs3117572 | 1.71E-16 |  |
| UC | *ATP6V1G2-DDX39B* | 510 | 9.42E-01 | 1.10E-03 | 5.54E-02 | 9.48E-05 | 1.42E-04 | rs2293370 | 4.26E-15 |  |
| UC | *BAG6* | 510 | 6.86E-01 | 2.68E-08 | 5.73E-04 | 3.47E-34 | 5.20E-34 | rs701008 | 6.76E-05 | √ |
| UC | *BRD2* | 510 | 9.36E-03 | 1.15E-06 | 1.22E-06 | 6.30E-03 | 9.45E-03 | rs28421666 | 8.67E-47 |  |
| UC | *BRWD1* | 510 | 5.36E-02 | 1.00E+00 | 1.00E+00 | 4.23E-21 | 6.34E-21 | rs28421666 | 8.67E-47 |  |
| UC | *BSN* | 510 | 3.86E-03 | 4.54E-09 | 5.33E-03 | 1.61E-11 | 2.42E-11 | rs1259601 | 4.77E-03 | √ |
| UC | *BTNL2* | 510 | 2.23E-01 | 1.00E+00 | 1.00E+00 | 3.94E-19 | 5.92E-19 | rs3117572 | 1.71E-16 |  |
| UC | *C11orf30* | 12 | 3.77E-01 | 1.00E+00 | 1.00E+00 | 1.02E-04 | 1.53E-04 | rs2293370 | 4.26E-15 |  |
| UC | *C1orf106* | 28 | 8.47E-02 | 1.00E+00 | 1.00E+00 | 5.98E-04 | 8.99E-04 | rs11655935 | 2.55E-04 | √ |
| UC | *C2* | 196 | 4.70E-01 | 3.17E-07 | 1.84E-03 | 1.97E-49 | 2.95E-49 | rs9469099 | 1.35E-32 |  |
| UC | *C21orf33* | 196 | 4.20E-02 | 8.18E-02 | 3.64E-02 | 3.46E-03 | 5.19E-03 | rs10488631 | 5.08E-23 |  |
| UC | *C3orf62* | 10 | 5.99E-03 | 2.72E-05 | 2.25E-07 | 4.26E-06 | 6.39E-06 | rs1431402 | 6.21E-39 |  |
| UC | *C3orf71* | 36 | 7.12E-04 | 4.74E-02 | 5.98E-03 | 1.00E+00 | 1.00E+00 | rs3117572 | 1.71E-16 |  |
| UC | *C4A* | 36 | 4.28E-01 | 4.08E-03 | 4.68E-01 | 9.73E-49 | 1.46E-48 | rs4938573 | 1.15E-13 |  |
| UC | *C4B* | 36 | 2.41E-01 | 2.80E-04 | 5.13E-02 | 9.51E-49 | 1.43E-48 | rs7774434 | 2.37E-56 |  |
| UC | *C6orf47* | 52 | 9.56E-01 | 5.13E-01 | 1.00E+00 | 1.55E-33 | 2.33E-33 | rs510372 | 1.68E-09 |  |
| UC | *C6orf48* | 56 | 2.19E-02 | 1.59E-07 | 6.57E-08 | 8.03E-07 | 1.20E-06 | rs2459144 | 2.15E-04 | √ |
| UC | *CAMSAP2* | 85 | 8.62E-02 | 2.10E-01 | 1.00E+00 | 8.24E-06 | 1.26E-05 | rs9469099 | 1.35E-32 |  |
| UC | *CAPN10* | 632 | 1.94E-01 | 1.00E+00 | 1.00E+00 | 1.31E-04 | 1.96E-04 | rs9469099 | 1.35E-32 |  |
| UC | *CBLL1* | 301 | 6.42E-01 | 9.20E-01 | 1.00E+00 | 2.37E-11 | 3.55E-11 | rs9469099 | 1.35E-32 |  |
| UC | *CCDC26* | 70 | 2.84E-01 | 9.35E-02 | 1.95E-02 | 1.00E+00 | 1.00E+00 | rs3117572 | 1.71E-16 |  |
| UC | *CCDC71* | 25 | 3.91E-01 | 2.04E-02 | 2.11E-02 | 1.00E+00 | 1.00E+00 | rs11759575 | 1.92E-21 |  |
| UC | *CCHCR1* | 62 | 8.26E-02 | 6.54E-08 | 1.95E-07 | 4.54E-12 | 6.81E-12 | rs510372 | 1.68E-09 |  |
| UC | *CCR10* | 175 | 4.70E-01 | 5.32E-01 | 8.44E-01 | 9.58E-03 | 1.44E-02 | rs3763295 | 2.74E-15 |  |
| UC | *CELSR3* | 22 | 8.04E-01 | 2.04E-02 | 1.00E+00 | 1.14E-02 | 1.70E-02 | rs2293370 | 4.26E-15 |  |
| UC | *CFB* | 22 | 2.56E-01 | 7.71E-06 | 5.40E-03 | 1.66E-48 | 2.49E-48 | rs2304256 | 1.05E-10 |  |
| UC | *CLIC1* | 22 | 3.94E-02 | 5.83E-02 | 1.00E+00 | 2.79E-34 | 4.18E-34 | rs2297067 | 6.34E-19 |  |
| UC | *CLN3* | 158 | 9.79E-01 | 2.94E-03 | 1.62E-03 | 1.00E+00 | 1.00E+00 | rs701008 | 6.76E-05 | √ |
| UC | *CNTNAP1* | 100 | 6.49E-01 | 8.36E-01 | 1.00E+00 | 1.08E-02 | 1.62E-02 | rs9469099 | 1.35E-32 |  |
| UC | *COASY* | 235 | 1.71E-01 | 1.33E-05 | 4.66E-07 | 3.86E-03 | 5.79E-03 | rs12924729 | 2.39E-14 |  |
| UC | *CSF3* | 146 | 1.78E-01 | 1.93E-07 | 1.10E-06 | 5.35E-05 | 8.04E-05 | rs12924729 | 2.39E-14 |  |
| UC | *CSNK2B* | 73 | 9.51E-01 | 6.66E-03 | 3.16E-02 | 8.57E-34 | 1.29E-33 | rs3117572 | 1.71E-16 |  |
| UC | *CTD-2330K9.3* | 211 | 1.70E-01 | 6.80E-07 | 3.58E-02 | 2.34E-01 | 3.50E-01 | rs7774434 | 2.37E-56 |  |
| UC | *CYP21A2* | 211 | 9.66E-03 | 5.85E-05 | 7.46E-01 | 3.39E-49 | 5.09E-49 | rs3117572 | 1.71E-16 |  |
| UC | *DAG1* | 247 | 1.28E-08 | 4.13E-08 | 2.42E-06 | 7.57E-09 | 1.14E-08 | rs12924729 | 2.39E-14 |  |
| UC | *DDAH2* | 171 | 2.54E-02 | 1.36E-01 | 1.00E+00 | 5.59E-33 | 8.38E-33 | rs12924729 | 2.39E-14 |  |
| UC | *DDX39B* | 171 | 6.53E-01 | 6.91E-03 | 1.00E+00 | 1.43E-10 | 2.15E-10 | rs701008 | 6.76E-05 | √ |
| UC | *DLD* | 326 | 1.41E-02 | 1.00E+00 | 1.00E+00 | 1.48E-09 | 2.22E-09 | rs4779112 | 1.63E-07 | √ |
| UC | *DOM3Z* | 57 | 8.09E-01 | 2.10E-06 | 5.76E-04 | 1.17E-47 | 1.76E-47 | rs4938573 | 1.15E-13 |  |
| UC | *DUSP28* | 214 | 1.71E-02 | 1.00E+00 | 1.00E+00 | 1.75E-05 | 2.63E-05 | rs9469099 | 1.35E-32 |  |
| UC | *DYRK3* | 214 | 4.40E-01 | 1.00E+00 | 1.00E+00 | 1.35E-13 | 2.03E-13 | rs701008 | 6.76E-05 | √ |
| UC | *EGFL8* | 214 | 9.19E-02 | 1.34E-05 | 3.13E-05 | 1.07E-54 | 1.61E-54 | rs3850302 | 3.38E-04 | √ |
| UC | *EHMT2* | 214 | 3.76E-01 | 3.27E-08 | 4.91E-04 | 2.61E-49 | 3.91E-49 | rs7216806 | 1.25E-02 | √ |
| UC | *EIF3CL* | 214 | 4.38E-01 | 7.83E-04 | 1.00E+00 | 1.00E+00 | 1.00E+00 | rs3117572 | 1.71E-16 |  |
| UC | *ENTPD7* | 214 | 5.19E-01 | 1.00E+00 | 1.00E+00 | 8.70E-06 | 1.32E-05 | rs4938573 | 1.15E-13 |  |
| UC | *ERBB2* | 214 | 5.34E-01 | 4.46E-06 | 1.07E-05 | 1.54E-08 | 2.30E-08 | rs12924729 | 2.39E-14 |  |
| UC | *EZH1* | 214 | 7.66E-01 | 1.00E+00 | 1.00E+00 | 1.85E-02 | 2.77E-02 | rs9469099 | 1.35E-32 |  |
| UC | *FAIM3* | 34 | 2.52E-01 | 7.13E-01 | 1.00E+00 | 4.82E-05 | 7.23E-05 | rs7216806 | 1.25E-02 | √ |
| UC | *FAM134C* | 209 | 7.45E-01 | 9.27E-03 | 5.88E-03 | 6.72E-03 | 1.01E-02 | rs28421666 | 8.67E-47 |  |
| UC | *FAM212A* | 209 | 1.40E-01 | 1.05E-05 | 6.37E-05 | 3.25E-01 | 4.88E-01 | rs9469099 | 1.35E-32 |  |
| UC | *FAM213B* | 156 | 3.30E-04 | 1.33E-04 | 5.01E-04 | 3.39E-02 | 5.09E-02 | rs27772 | 4.42E-03 | √ |
| UC | *FAM98B* | 156 | 6.52E-01 | 2.01E-02 | 1.00E+00 | 1.00E+00 | 1.00E+00 | rs7224568 | 4.10E-03 | √ |
| UC | *FCGR2A* | 73 | 3.85E-01 | 4.46E-10 | 6.92E-10 | 4.39E-13 | 6.58E-13 | rs510372 | 1.68E-09 |  |
| UC | *FCGR2B* | 247 | 5.40E-01 | 1.00E+00 | 1.00E+00 | 6.24E-12 | 9.36E-12 | rs291089 | 3.13E-04 | √ |
| UC | *FKBPL* | 247 | 2.16E-01 | 3.08E-02 | 1.00E+00 | 3.00E-55 | 4.49E-55 | rs2459144 | 2.15E-04 | √ |
| UC | *FLNC* | 77 | 4.80E-01 | 8.86E-03 | 8.64E-03 | 1.00E+00 | 1.00E+00 | rs2304256 | 1.05E-10 |  |
| UC | *GABBR1* | 77 | 8.11E-01 | 1.00E+00 | 1.00E+00 | 7.47E-03 | 1.12E-02 | rs9469099 | 1.35E-32 |  |
| UC | *GMEB2* | 77 | 2.51E-03 | 1.00E+00 | 1.00E+00 | 7.02E-05 | 1.05E-04 | rs10488631 | 5.08E-23 |  |
| UC | *GMPPB* | 77 | 4.64E-01 | 4.64E-06 | 4.95E-05 | 3.70E-11 | 5.56E-11 | rs13202536 | 1.21E-04 | √ |
| UC | *GOT1* | 77 | 1.39E-01 | 1.00E+00 | 1.00E+00 | 5.93E-09 | 8.90E-09 | rs3117572 | 1.71E-16 |  |
| UC | *GPANK1* | 77 | 6.18E-01 | 3.66E-01 | 1.00E+00 | 4.80E-33 | 7.20E-33 | rs510372 | 1.68E-09 |  |
| UC | *GPR25* | 77 | 1.64E-01 | 1.00E+00 | 1.00E+00 | 4.26E-05 | 6.45E-05 | rs7216806 | 1.25E-02 | √ |
| UC | *GPR35* | 77 | 4.96E-01 | 1.00E+00 | 1.00E+00 | 5.23E-06 | 7.85E-06 | rs28421666 | 8.67E-47 |  |
| UC | *GPSM3* | 183 | 8.95E-01 | 1.04E-05 | 7.61E-04 | 8.91E-56 | 1.34E-55 | rs7224568 | 4.10E-03 | √ |
| UC | *GPX1* | 183 | 6.37E-07 | 3.53E-07 | 2.02E-07 | 3.86E-07 | 5.77E-07 | rs7224568 | 4.10E-03 | √ |
| UC | *GRB7* | 842 | 6.91E-01 | 5.19E-07 | 2.79E-06 | 3.15E-08 | 5.02E-08 | rs11832772 | 7.31E-07 | √ |
| UC | *GRK6* | 842 | 1.61E-01 | 2.50E-02 | 3.35E-02 | 1.00E+00 | 1.00E+00 | rs2233976 | 2.37E-14 |  |
| UC | *GS1-410F4.2* | 842 | 4.51E-01 | 1.00E+00 | 1.00E+00 | 1.63E-20 | 2.44E-20 | rs3763295 | 2.74E-15 |  |
| UC | *GSDMB* | 183 | 8.63E-01 | 7.46E-06 | 1.80E-04 | 7.66E-09 | 1.15E-08 | rs9967367 | 9.60E-03 | √ |
| UC | *HCG22* | 156 | 3.51E-01 | 2.27E-03 | 2.81E-01 | 1.94E-06 | 3.17E-06 | rs201004 | 1.02E-07 | √ |
| UC | *HCG27* | 54 | 2.63E-01 | 1.13E-04 | 1.00E+00 | 2.07E-11 | 3.11E-11 | rs12200985 | 1.41E-07 | √ |
| UC | *HES5* | 120 | 7.53E-03 | 6.64E-02 | 1.00E+00 | 4.88E-02 | 7.31E-02 | rs201004 | 1.02E-07 | √ |
| UC | *HLA-B* | 120 | 8.99E-01 | 1.00E+00 | 1.00E+00 | 1.61E-07 | 2.38E-07 | rs201004 | 1.02E-07 | √ |
| UC | *HLA-C* | 1167 | 4.35E-01 | 8.58E-01 | 1.00E+00 | 1.20E-11 | 1.80E-11 | rs201004 | 1.02E-07 | √ |
| UC | *HLA-DMA* | 1167 | 7.80E-03 | 1.17E-06 | 1.22E-06 | 6.42E-03 | 9.63E-03 | rs201004 | 1.02E-07 | √ |
| UC | *HLA-DMB* | 1167 | 2.27E-01 | 1.13E-06 | 1.22E-06 | 6.19E-03 | 9.28E-03 | rs201004 | 1.02E-07 | √ |
| UC | *HLA-DOA* | 1167 | 4.07E-02 | 1.45E-06 | 1.13E-06 | 7.32E-03 | 1.10E-02 | rs201004 | 1.02E-07 | √ |
| UC | *HLA-DOB* | 1167 | 1.71E-03 | 3.05E-04 | 5.86E-03 | 3.26E-07 | 4.89E-07 | rs201004 | 1.02E-07 | √ |
| UC | *HLA-DPA1* | 250 | 7.23E-02 | 1.48E-06 | 1.23E-06 | 7.72E-03 | 1.16E-02 | rs201004 | 1.02E-07 | √ |
| UC | *HLA-DPB1* | 147 | 5.15E-01 | 3.26E-06 | 2.40E-06 | 1.58E-02 | 2.37E-02 | rs201004 | 1.02E-07 | √ |
| UC | *HLA-DQA1* | 55 | 3.78E-05 | 9.70E-67 | 5.95E-54 | 3.36E-79 | 5.04E-79 | rs10484439 | 4.17E-07 | √ |
| UC | *HLA-DQA2* | 5 | 1.56E-06 | 1.77E-10 | 1.00E+00 | 7.38E-41 | 1.11E-40 | rs201004 | 1.02E-07 | √ |
| UC | *HLA-DQB1* | 704 | 3.30E-06 | 1.48E-38 | 5.96E-26 | 6.11E-79 | 9.16E-79 | rs201004 | 1.02E-07 | √ |
| UC | *HLA-DQB2* | 62 | 4.68E-05 | 9.20E-10 | 7.01E-06 | 1.51E-41 | 2.26E-41 | rs201004 | 1.02E-07 | √ |
| UC | *HLA-DRA* | 120 | 1.16E-02 | 1.00E+00 | 1.00E+00 | 1.22E-18 | 1.83E-18 | rs201004 | 1.02E-07 | √ |
| UC | *HLA-DRB1* | 17 | 5.48E-07 | 6.38E-52 | 5.86E-34 | 2.14E-79 | 3.21E-79 | rs201004 | 1.02E-07 | √ |
| UC | *HLA-DRB5* | 157 | 1.93E-06 | 5.24E-54 | 2.20E-33 | 1.76E-79 | 2.64E-79 | rs3117572 | 1.71E-16 |  |
| UC | *HLA-F* | 14 | 8.69E-01 | 1.00E+00 | 1.00E+00 | 9.80E-03 | 1.47E-02 | rs3763295 | 2.74E-15 |  |
| UC | *HSD17B1* | 14 | 7.34E-02 | 4.48E-03 | 1.50E-02 | 5.17E-03 | 7.76E-03 | rs7774434 | 2.37E-56 |  |
| UC | *HSPA1A* | 61 | 1.14E-01 | 6.85E-02 | 1.00E+00 | 5.58E-07 | 8.40E-07 | rs7774434 | 2.37E-56 |  |
| UC | *HSPA1B* | 61 | 2.34E-02 | 8.55E-04 | 1.74E-03 | 1.27E-07 | 1.88E-07 | rs7774434 | 2.37E-56 |  |
| UC | *HSPA1L* | 61 | 4.12E-01 | 1.36E-03 | 4.23E-02 | 1.75E-08 | 2.62E-08 | rs7774434 | 2.37E-56 |  |
| UC | *HSPA6* | 104 | 5.07E-01 | 6.76E-14 | 8.18E-10 | 1.32E-12 | 1.98E-12 | rs7774434 | 2.37E-56 |  |
| UC | *ICOSLG* | 104 | 1.93E-01 | 9.16E-03 | 1.56E-02 | 2.94E-03 | 4.40E-03 | rs7774434 | 2.37E-56 |  |
| UC | *IFNG* | 104 | 1.69E-01 | 2.28E-01 | 1.00E+00 | 1.78E-21 | 2.66E-21 | rs7774434 | 2.37E-56 |  |
| UC | *IKZF3* | 104 | 6.15E-01 | 9.26E-12 | 2.42E-11 | 2.88E-08 | 3.76E-08 | rs7774434 | 2.37E-56 |  |
| UC | *IL10* | 104 | 1.63E-02 | 4.37E-03 | 1.00E+00 | 2.30E-13 | 3.45E-13 | rs7774434 | 2.37E-56 |  |
| UC | *IL12B* | 104 | 4.61E-02 | 4.09E-04 | 8.70E-04 | 6.76E-11 | 1.01E-10 | rs7774434 | 2.37E-56 |  |
| UC | *IL12RB2* | 104 | 8.59E-03 | 2.09E-14 | 1.00E+00 | 3.63E-31 | 5.45E-31 | rs7774434 | 2.37E-56 |  |
| UC | *IL19* | 104 | 1.97E-02 | 2.29E-02 | 1.00E+00 | 9.45E-13 | 1.42E-12 | rs7774434 | 2.37E-56 |  |
| UC | *IL23R* | 3 | 8.42E-01 | 1.27E-05 | 3.16E-02 | 7.23E-30 | 1.08E-29 | rs7774434 | 2.37E-56 |  |
| UC | *IL24* | 3 | 1.53E-01 | 3.34E-06 | 1.04E-05 | 5.38E-05 | 8.06E-05 | rs3117572 | 1.71E-16 |  |
| UC | *IL26* | 57 | 3.55E-01 | 1.00E+00 | 1.00E+00 | 4.58E-21 | 6.87E-21 | rs3117572 | 1.71E-16 |  |
| UC | *IMPDH2* | 57 | 2.97E-03 | 7.19E-02 | 3.33E-02 | 1.00E+00 | 1.00E+00 | rs3117572 | 1.71E-16 |  |
| UC | *IP6K1* | 4 | 4.19E-01 | 3.90E-09 | 8.64E-07 | 1.35E-11 | 2.02E-11 | rs2304256 | 1.05E-10 |  |
| UC | *IP6K2* | 4 | 2.06E-01 | 1.07E-06 | 9.61E-04 | 8.67E-03 | 1.30E-02 | rs2304256 | 1.05E-10 |  |
| UC | *IRF5* | 16 | 8.96E-01 | 4.13E-02 | 1.46E-02 | 1.00E+00 | 1.00E+00 | rs2304256 | 1.05E-10 |  |
| UC | *JAK2* | 16 | 8.68E-01 | 6.22E-09 | 3.60E-08 | 5.55E-08 | 8.78E-08 | rs2304256 | 1.05E-10 |  |
| UC | *KIAA1841* | 16 | 5.25E-01 | 1.73E-02 | 1.00E+00 | 1.64E-12 | 2.46E-12 | rs9967367 | 9.60E-03 | √ |
| UC | *KIF1A* | 34 | 8.15E-01 | 1.00E+00 | 1.00E+00 | 2.59E-06 | 3.89E-06 | rs485499 | 1.43E-23 |  |
| UC | *KIF21B* | 34 | 5.19E-01 | 1.00E+00 | 1.00E+00 | 2.17E-05 | 3.23E-05 | rs7224568 | 4.10E-03 | √ |
| UC | *KLHDC8B* | 36 | 1.19E-02 | 2.13E-02 | 2.03E-02 | 1.00E+00 | 1.00E+00 | rs485499 | 1.43E-23 |  |
| UC | *LAMB1* | 36 | 3.23E-01 | 2.26E-01 | 7.41E-01 | 6.64E-10 | 9.96E-10 | rs6679356 | 7.49E-28 |  |
| UC | *LAMB2* | 36 | 1.39E-04 | 1.61E-02 | 2.49E-02 | 1.00E+00 | 1.00E+00 | rs6679356 | 7.49E-28 |  |
| UC | *LIF* | 66 | 2.80E-01 | 4.23E-03 | 1.00E+00 | 1.00E+00 | 1.00E+00 | rs485499 | 1.43E-23 |  |
| UC | *LIME1* | 108 | 6.85E-01 | 1.00E+00 | 1.00E+00 | 9.63E-05 | 1.44E-04 | rs10488631 | 5.08E-23 |  |
| UC | *LINC00481* | 108 | 4.40E-01 | 1.00E+00 | 1.00E+00 | 3.98E-12 | 5.98E-12 | rs2459144 | 2.15E-04 | √ |
| UC | *LRRC32* | 245 | 4.37E-01 | 2.73E-01 | 1.00E+00 | 2.04E-04 | 3.06E-04 | rs9967367 | 9.60E-03 | √ |
| UC | *LSM2* | 65 | 6.99E-02 | 6.93E-04 | 2.82E-03 | 1.77E-33 | 2.66E-33 | rs3745516 | 1.22E-20 |  |
| UC | *LST1* | 354 | 7.48E-01 | 2.51E-03 | 1.00E+00 | 7.38E-30 | 1.11E-29 | rs485499 | 1.43E-23 |  |
| UC | *LTA* | 155 | 4.90E-01 | 1.66E-02 | 1.00E+00 | 5.42E-29 | 8.14E-29 | rs12200985 | 1.41E-07 | √ |
| UC | *LTB* | 155 | 3.12E-01 | 5.53E-05 | 4.51E-01 | 6.56E-31 | 9.83E-31 | rs3763295 | 2.74E-15 |  |
| UC | *LY6G5B* | 155 | 7.95E-01 | 3.52E-03 | 4.57E-01 | 2.46E-34 | 3.68E-34 | rs7216806 | 1.25E-02 | √ |
| UC | *LY6G5C* | 155 | 6.81E-01 | 1.16E-03 | 1.00E+00 | 2.59E-34 | 3.89E-34 | rs2459144 | 2.15E-04 | √ |
| UC | *MAPKAPK2* | 10 | 1.48E-02 | 1.00E+00 | 1.00E+00 | 1.13E-13 | 1.69E-13 | rs3117572 | 1.71E-16 |  |
| UC | *MED24* | 10 | 8.12E-01 | 2.72E-06 | 1.58E-05 | 2.40E-04 | 3.60E-04 | rs3117572 | 1.71E-16 |  |
| UC | *MICA* | 378 | 7.15E-01 | 1.64E-02 | 5.51E-02 | 4.73E-07 | 7.15E-07 | rs3117572 | 1.71E-16 |  |
| UC | *MICB* | 378 | 8.91E-01 | 3.70E-08 | 7.29E-09 | 7.55E-08 | 1.13E-07 | rs3117572 | 1.71E-16 |  |
| UC | *MIEN1* | 165 | 7.32E-01 | 3.27E-03 | 1.00E+00 | 2.37E-08 | 3.55E-08 | rs701008 | 6.76E-05 | √ |
| UC | *MIER1* | 165 | 5.06E-01 | 3.34E-02 | 1.00E+00 | 3.29E-01 | 4.94E-01 | rs7224568 | 4.10E-03 | √ |
| UC | *MLX* | 165 | 6.71E-01 | 6.03E-03 | 2.18E-06 | 6.47E-03 | 9.70E-03 | rs701008 | 6.76E-05 | √ |
| UC | *MMEL1* | 165 | 2.27E-03 | 1.49E-04 | 4.45E-01 | 1.72E-02 | 2.58E-02 | rs701008 | 6.76E-05 | √ |
| UC | *MON1A* | 165 | 3.40E-01 | 4.33E-07 | 1.00E+00 | 3.26E-01 | 4.89E-01 | rs3117572 | 1.71E-16 |  |
| UC | *MSH5* | 165 | 5.54E-02 | 4.66E-05 | 1.00E+00 | 2.71E-35 | 4.06E-35 | rs7224568 | 4.10E-03 | √ |
| UC | *MSH5-SAPCD1* | 165 | 8.00E-02 | 5.13E-03 | 5.37E-01 | 1.07E-34 | 1.61E-34 | rs3117572 | 1.71E-16 |  |
| UC | *MST1* | 165 | 5.66E-01 | 9.62E-07 | 2.13E-03 | 8.78E-12 | 1.32E-11 | rs3117572 | 1.71E-16 |  |
| UC | *MST1R* | 57 | 7.28E-04 | 6.38E-05 | 1.00E+00 | 4.41E-01 | 6.61E-01 | rs2233976 | 2.37E-14 |  |
| UC | *MUC22* | 57 | 2.38E-02 | 1.00E+00 | 1.00E+00 | 4.07E-06 | 6.57E-06 | rs2459144 | 2.15E-04 | √ |
| UC | *NAGLU* | 142 | 4.55E-01 | 1.00E+00 | 1.00E+00 | 2.66E-02 | 3.99E-02 | rs11172113 | 3.60E-05 | √ |
| UC | *NCKIPSD* | 288 | 9.93E-02 | 2.24E-09 | 1.05E-03 | 1.00E-02 | 1.50E-02 | rs7673766 | 4.20E-03 | √ |
| UC | *NCR3* | 200 | 3.31E-01 | 7.02E-01 | 1.00E+00 | 2.61E-30 | 3.91E-30 | rs3745516 | 1.22E-20 |  |
| UC | *NEU1* | 200 | 4.36E-01 | 3.19E-06 | 1.77E-02 | 8.38E-49 | 1.26E-48 | rs570963 | 3.17E-22 |  |
| UC | *NFKBIL1* | 200 | 5.84E-01 | 2.39E-02 | 1.00E+00 | 6.34E-08 | 1.00E-07 | rs7676765 | 9.82E-07 | √ |
| UC | *NICN1* | 200 | 3.86E-10 | 9.56E-07 | 5.98E-06 | 1.54E-07 | 2.26E-07 | rs4475032 | 1.16E-02 | √ |
| UC | *NKIRAS2* | 200 | 4.62E-01 | 8.04E-02 | 4.63E-02 | 1.00E+00 | 1.00E+00 | rs7774434 | 2.37E-56 |  |
| UC | *NOTCH4* | 230 | 9.13E-01 | 1.70E-05 | 2.59E-04 | 2.15E-55 | 3.22E-55 | rs3745516 | 1.22E-20 |  |
| UC | *NPIPL1* | 249 | 2.97E-01 | 4.22E-03 | 8.41E-03 | 1.00E+00 | 1.00E+00 | rs9906813 | 4.68E-03 | √ |
| UC | *NR5A2* | 112 | 9.70E-01 | 1.00E+00 | 1.00E+00 | 8.01E-03 | 1.20E-02 | rs4475032 | 1.16E-02 | √ |
| UC | *NSD1* | 112 | 6.18E-01 | 4.82E-02 | 1.17E-01 | 1.00E+00 | 1.00E+00 | rs7224568 | 4.10E-03 | √ |
| UC | *ORMDL3* | 112 | 6.18E-01 | 9.94E-13 | 3.03E-09 | 8.92E-09 | 1.34E-08 | rs701008 | 6.76E-05 | √ |
| UC | *OTUD3* | 112 | 9.89E-01 | 1.16E-42 | 1.07E-26 | 8.76E-33 | 1.31E-32 | rs28421666 | 8.67E-47 |  |
| UC | *P4HTM* | 14 | 2.50E-02 | 1.61E-02 | 4.13E-04 | 1.00E+00 | 1.00E+00 | rs1431402 | 6.21E-39 |  |
| UC | *PANK4* | 468 | 3.20E-02 | 2.44E-05 | 8.38E-02 | 2.08E-02 | 3.13E-02 | rs7224568 | 4.10E-03 | √ |
| UC | *PAPOLG* | 203 | 5.55E-01 | 1.00E+00 | 1.00E+00 | 4.33E-04 | 6.50E-04 | rs201004 | 1.02E-07 | √ |
| UC | *PBX2* | 203 | 7.88E-01 | 1.48E-05 | 2.46E-02 | 3.12E-55 | 4.68E-55 | rs2293370 | 4.26E-15 |  |
| UC | *PEX13* | 22 | 5.27E-01 | 5.54E-11 | 1.34E-10 | 4.81E-13 | 7.22E-13 | rs510372 | 1.68E-09 |  |
| UC | *PGAP3* | 123 | 6.53E-02 | 1.10E-01 | 1.00E+00 | 3.86E-08 | 6.27E-08 | rs2293370 | 4.26E-15 |  |
| UC | *PKIG* | 123 | 9.55E-01 | 2.43E-01 | 1.00E+00 | 1.28E-04 | 2.18E-04 | rs3745516 | 1.22E-20 |  |
| UC | *PLCH2* | 123 | 1.01E-02 | 9.29E-05 | 1.38E-01 | 1.15E-02 | 1.72E-02 | rs12200985 | 1.41E-07 | √ |
| UC | *PLEKHH3* | 123 | 6.57E-01 | 1.00E+00 | 1.00E+00 | 1.05E-02 | 1.58E-02 | rs3763295 | 2.74E-15 |  |
| UC | *PNPLA2* | 123 | 2.14E-03 | 5.66E-04 | 1.00E+00 | 1.00E+00 | 1.00E+00 | rs28421666 | 8.67E-47 |  |
| UC | *POU5F1* | 123 | 4.78E-01 | 1.00E+00 | 1.00E+00 | 1.07E-12 | 1.61E-12 | rs9469099 | 1.35E-32 |  |
| UC | *PPT2* | 123 | 4.31E-02 | 3.19E-06 | 5.05E-04 | 1.22E-54 | 1.83E-54 | rs12200985 | 1.41E-07 | √ |
| UC | *PRKAR2A* | 123 | 1.97E-01 | 1.00E+00 | 1.00E+00 | 4.99E-02 | 7.49E-02 | rs8108848 | 8.69E-05 | √ |
| UC | *PRRC2A* | 1085 | 8.56E-01 | 3.88E-05 | 5.72E-02 | 3.79E-34 | 5.69E-34 | rs7774434 | 2.37E-56 |  |
| UC | *PRRT1* | 374 | 4.99E-01 | 4.14E-01 | 1.00E+00 | 8.80E-54 | 1.32E-53 | rs7774434 | 2.37E-56 |  |
| UC | *PSMB8* | 374 | 6.00E-03 | 3.75E-03 | 1.00E+00 | 5.28E-07 | 7.90E-07 | rs7224568 | 4.10E-03 | √ |
| UC | *PSMB9* | 374 | 4.42E-04 | 4.79E-08 | 1.00E+00 | 1.03E-05 | 1.55E-05 | rs2233976 | 2.37E-14 |  |
| UC | *PSMC3IP* | 374 | 7.05E-01 | 2.44E-01 | 1.00E+00 | 6.57E-03 | 9.85E-03 | rs7216806 | 1.25E-02 | √ |
| UC | *PSMD3* | 374 | 7.59E-01 | 1.69E-11 | 2.64E-13 | 3.68E-09 | 5.52E-09 | rs2304256 | 1.05E-10 |  |
| UC | *PSMG1* | 374 | 2.09E-03 | 1.00E+00 | 1.00E+00 | 1.01E-19 | 1.52E-19 | rs9469099 | 1.35E-32 |  |
| UC | *PSORS1C1* | 374 | 3.35E-01 | 3.72E-02 | 1.00E+00 | 6.80E-07 | 1.08E-06 | rs7216806 | 1.25E-02 | √ |
| UC | *PUS10* | 134 | 5.36E-01 | 1.99E-12 | 2.51E-10 | 3.28E-13 | 4.93E-13 | rs1431402 | 6.21E-39 |  |
| UC | *PWP2* | 134 | 7.76E-02 | 1.41E-02 | 1.50E-02 | 1.78E-03 | 2.67E-03 | rs7774434 | 2.37E-56 |  |
| UC | *QARS* | 18 | 8.05E-05 | 2.18E-02 | 2.21E-02 | 1.00E+00 | 1.00E+00 | rs12924729 | 2.39E-14 |  |
| UC | *QRICH1* | 18 | 1.06E-03 | 2.65E-02 | 2.20E-02 | 1.00E+00 | 1.00E+00 | rs28421666 | 8.67E-47 |  |
| UC | *RBM5* | 252 | 9.30E-03 | 7.90E-04 | 1.69E-03 | 1.00E+00 | 1.00E+00 | rs4465620 | 1.67E-05 | √ |
| UC | *RBM6* | 252 | 2.08E-01 | 4.58E-05 | 1.86E-04 | 7.40E-01 | 1.00E+00 | rs4465620 | 1.67E-05 | √ |
| UC | *RDBP* | 97 | 9.84E-01 | 3.63E-02 | 1.00E+00 | 3.62E-48 | 5.44E-48 | rs7575363 | 9.01E-05 | √ |
| UC | *REL* | 97 | 8.69E-01 | 1.26E-10 | 2.17E-07 | 3.72E-13 | 5.59E-13 | rs701008 | 6.76E-05 | √ |
| UC | *RHOA* | 94 | 2.95E-10 | 3.28E-07 | 2.06E-04 | 1.75E-07 | 2.63E-07 | rs701008 | 6.76E-05 | √ |
| UC | *RIMBP3B* | 93 | 1.85E-06 | 1.00E+00 | 1.00E+00 | 1.00E+00 | 1.00E+00 | rs8005065 | 1.46E-02 | √ |
| UC | *RLN1* | 93 | 2.93E-01 | 1.50E-01 | 1.00E+00 | 1.18E-02 | 1.77E-02 | rs1431402 | 6.21E-39 |  |
| UC | *RNF123* | 200 | 9.10E-02 | 7.09E-09 | 7.52E-08 | 1.89E-11 | 2.84E-11 | rs510372 | 1.68E-09 |  |
| UC | *RNF5* | 472 | 6.83E-01 | 1.15E-05 | 3.73E-04 | 4.39E-55 | 6.59E-55 | rs7774434 | 2.37E-56 |  |
| UC | *RNPEPL1* | 303 | 7.05E-02 | 1.00E+00 | 1.00E+00 | 8.22E-05 | 1.23E-04 | rs3117572 | 1.71E-16 |  |
| UC | *RP11-1348G14.2* | 30 | 1.69E-08 | 4.19E-01 | 5.54E-01 | 1.00E+00 | 1.00E+00 | rs485499 | 1.43E-23 |  |
| UC | *RP11-168O16.1* | 28 | 4.29E-01 | 1.00E+00 | 1.00E+00 | 5.99E-04 | 8.98E-04 | rs1954118 | 2.12E-16 |  |
| UC | *RP11-356I2.1* | 255 | 7.39E-01 | 1.00E+00 | 1.00E+00 | 1.51E-04 | 2.29E-04 | rs11065987 | 3.20E-08 |  |
| UC | *RP11-95M15.1* | 138 | 7.76E-01 | 1.00E+00 | 1.00E+00 | 8.48E-05 | 1.28E-04 | rs9469099 | 1.35E-32 |  |
| UC | *RP3-395M20.8* | 138 | 1.28E-02 | 2.57E-05 | 2.26E-02 | 1.12E-02 | 1.69E-02 | rs7774434 | 2.37E-56 |  |
| UC | *RP4-697K14.7* | 138 | 4.74E-03 | 1.04E-05 | 1.30E-05 | 4.04E-01 | 6.06E-01 | rs4465620 | 1.67E-05 | √ |
| UC | *RPLP2* | 138 | 2.53E-03 | 4.64E-02 | 1.00E+00 | 1.00E+00 | 1.00E+00 | rs485499 | 1.43E-23 |  |
| UC | *RTEL1* | 138 | 3.04E-01 | 1.00E+00 | 1.00E+00 | 1.12E-04 | 1.68E-04 | rs12924729 | 2.39E-14 |  |
| UC | *RTEL1* | 138 | 3.04E-01 | 1.00E+00 | 1.00E+00 | 1.12E-04 | 1.68E-04 | rs3745516 | 1.22E-20 |  |
| UC | *RTEL1* | 138 | 2.11E-01 | 1.00E+00 | 1.00E+00 | 3.13E-05 | 4.70E-05 | rs4475032 | 1.16E-02 | √ |
| UC | *RTEL1* | 138 | 2.11E-01 | 1.00E+00 | 1.00E+00 | 3.13E-05 | 4.70E-05 | rs9469099 | 1.35E-32 |  |
| UC | *SAPCD1* | 35 | 3.59E-01 | 2.54E-01 | 1.00E+00 | 6.68E-34 | 1.00E-33 | rs7557937 | 9.58E-05 | √ |
| UC | *SEMA3F* | 203 | 2.57E-01 | 1.71E-02 | 1.00E+00 | 1.00E+00 | 1.00E+00 | rs7216806 | 1.25E-02 | √ |
| UC | *1-Sep* | 632 | 5.08E-01 | 5.15E-03 | 2.41E-03 | 1.00E+00 | 1.00E+00 | rs7774434 | 2.37E-56 |  |
| UC | *SERINC3* | 122 | 7.49E-01 | 1.00E+00 | 1.00E+00 | 3.32E-05 | 5.63E-05 | rs7774434 | 2.37E-56 |  |
| UC | *SKIV2L* | 121 | 4.27E-01 | 3.56E-10 | 1.36E-08 | 6.03E-49 | 9.04E-49 | rs9674621 | 2.61E-03 | √ |
| UC | *SLC25A20* | 36 | 2.28E-03 | 8.10E-02 | 1.02E-02 | 1.00E+00 | 1.00E+00 | rs9674621 | 2.61E-03 | √ |
| UC | *SLC25A28* | 54 | 4.72E-01 | 4.69E-01 | 1.00E+00 | 5.59E-11 | 8.38E-11 | rs7224568 | 4.10E-03 | √ |
| UC | *SLC26A4* | 54 | 3.54E-01 | 1.00E+00 | 1.00E+00 | 2.74E-03 | 4.17E-03 | rs9967367 | 9.60E-03 | √ |
| UC | *SLC26A6* | 54 | 8.04E-01 | 1.00E+00 | 1.00E+00 | 2.79E-02 | 4.19E-02 | rs9967367 | 9.60E-03 | √ |
| UC | *SLC2A4RG* | 134 | 8.38E-01 | 1.00E+00 | 1.00E+00 | 4.80E-05 | 7.20E-05 | rs3763295 | 2.74E-15 |  |
| UC | *SLC35D1* | 53 | 2.14E-01 | 3.22E-05 | 5.30E-03 | 3.14E-02 | 4.71E-02 | rs2293370 | 4.26E-15 |  |
| UC | *SRMS* | 81 | 3.58E-01 | 1.39E-02 | 1.00E+00 | 4.16E-01 | 6.24E-01 | rs11172113 | 3.60E-05 | √ |
| UC | *STARD3* | 81 | 2.81E-01 | 1.00E+00 | 1.00E+00 | 3.61E-06 | 5.72E-06 | rs2293370 | 4.26E-15 |  |
| UC | *STAT5B* | 81 | 3.40E-01 | 6.45E-04 | 1.00E+00 | 1.00E+00 | 1.00E+00 | rs2297067 | 6.34E-19 |  |
| UC | *STK19* | 81 | 7.58E-01 | 1.22E-07 | 1.33E-03 | 1.53E-48 | 2.29E-48 | rs7020333 | 5.49E-03 | √ |
| UC | *STMN3* | 81 | 4.36E-02 | 7.77E-07 | 2.62E-07 | 1.00E-05 | 1.51E-05 | rs10488631 | 5.08E-23 |  |
| UC | *TAP1* | 81 | 7.57E-04 | 3.16E-03 | 5.10E-03 | 6.21E-06 | 9.12E-06 | rs9469099 | 1.35E-32 |  |
| UC | *TAP2* | 81 | 2.56E-03 | 1.00E+00 | 1.00E+00 | 1.56E-05 | 2.34E-05 | rs510372 | 1.68E-09 |  |
| UC | *TCAP* | 81 | 1.33E-01 | 4.02E-01 | 1.00E+00 | 2.11E-06 | 3.22E-06 | rs701008 | 6.76E-05 | √ |
| UC | *TCF19* | 32 | 4.15E-01 | 1.00E+00 | 1.00E+00 | 2.09E-13 | 3.14E-13 | rs701008 | 6.76E-05 | √ |
| UC | *TCTA* | 56 | 2.08E-15 | 3.74E-06 | 1.05E-03 | 8.09E-08 | 1.25E-07 | rs7557937 | 9.58E-05 | √ |
| UC | *TMCO4* | 56 | 7.82E-01 | 5.46E-24 | 2.76E-22 | 7.67E-32 | 1.15E-31 | rs2304256 | 1.05E-10 |  |
| UC | *TMEM9* | 375 | 9.60E-01 | 1.00E+00 | 1.00E+00 | 1.56E-03 | 2.31E-03 | rs17663721 | 2.27E-03 | √ |
| UC | *TMPRSS3* | 375 | 9.83E-01 | 1.00E+00 | 1.00E+00 | 1.69E-17 | 2.54E-17 | rs4938573 | 1.15E-13 |  |
| UC | *TNF* | 375 | 5.90E-01 | 3.23E-02 | 1.00E+00 | 3.70E-29 | 5.54E-29 | rs3117572 | 1.71E-16 |  |
| UC | *TNFRSF14* | 375 | 2.65E-03 | 1.13E-04 | 1.22E-01 | 3.29E-02 | 4.93E-02 | rs1431402 | 6.21E-39 |  |
| UC | *TNFSF15* | 375 | 4.50E-01 | 3.34E-10 | 8.29E-10 | 3.75E-06 | 5.63E-06 | rs3117572 | 1.71E-16 |  |
| UC | *TNFSF8* | 375 | 6.59E-01 | 1.00E+00 | 1.00E+00 | 4.57E-03 | 6.86E-03 | rs1431402 | 6.21E-39 |  |
| UC | *TNXB* | 375 | 4.85E-03 | 3.78E-05 | 2.89E-02 | 3.45E-54 | 5.17E-54 | rs10468514 | 2.06E-03 | √ |
| UC | *TOM1* | 375 | 6.76E-01 | 1.83E-02 | 9.80E-01 | 1.00E+00 | 1.00E+00 | rs7774434 | 2.37E-56 |  |
| UC | *TPPP* | 229 | 1.63E-04 | 1.50E-04 | 1.52E-03 | 1.00E+00 | 1.00E+00 | rs7774434 | 2.37E-56 |  |
| UC | *TRAIP* | 435 | 1.35E-01 | 7.15E-06 | 5.46E-05 | 3.80E-01 | 5.70E-01 | rs28421666 | 8.67E-47 |  |
| UC | *TRAPPC10* | 435 | 9.90E-01 | 1.00E+00 | 1.00E+00 | 1.97E-02 | 2.95E-02 | rs3117572 | 1.71E-16 |  |
| UC | *TTC34* | 232 | 1.21E-03 | 1.68E-05 | 1.96E-05 | 2.35E-02 | 3.53E-02 | rs11832772 | 7.31E-07 | √ |
| UC | *TTPAL* | 232 | 5.84E-01 | 1.00E+00 | 1.00E+00 | 2.36E-05 | 4.01E-05 | rs4952108 | 5.05E-08 | √ |
| UC | *TUBG1* | 232 | 3.19E-01 | 1.00E+00 | 1.00E+00 | 2.17E-02 | 3.25E-02 | rs9469099 | 1.35E-32 |  |
| UC | *TUBG2* | 112 | 6.18E-01 | 2.38E-01 | 2.21E-02 | 8.97E-03 | 1.35E-02 | rs2304256 | 1.05E-10 |  |
| UC | *UBA7* | 253 | 2.17E-01 | 1.01E-05 | 6.73E-05 | 5.96E-01 | 8.94E-01 | rs201004 | 1.02E-07 | √ |
| UC | *UBLCP1* | 36 | 3.83E-01 | 1.00E+00 | 1.00E+00 | 4.66E-10 | 6.98E-10 | rs201004 | 1.02E-07 | √ |
| UC | *USP19* | 36 | 5.38E-03 | 3.19E-02 | 2.49E-02 | 1.00E+00 | 1.00E+00 | rs9674621 | 2.61E-03 | √ |
| UC | *USP4* | 36 | 2.53E-01 | 1.32E-05 | 3.16E-04 | 4.36E-07 | 6.52E-07 | rs7224568 | 4.10E-03 | √ |
| UC | *VARS* | 76 | 1.26E-01 | 5.56E-04 | 1.00E+00 | 6.40E-35 | 9.60E-35 | rs209181 | 1.91E-07 | √ |
| UC | *VWA1* | 76 | 5.68E-09 | 1.00E+00 | 1.00E+00 | 1.00E+00 | 1.00E+00 | rs209181 | 1.91E-07 | √ |
| UC | *VWA7* | 154 | 3.21E-02 | 2.24E-01 | 1.00E+00 | 1.35E-34 | 2.02E-34 | rs3130612 | 8.23E-245 |  |
| UC | *WBSCR27* | 216 | 8.70E-01 | 2.87E-02 | 2.63E-02 | 1.00E+00 | 1.00E+00 | rs66462181 | 9.20E-81 |  |
| UC | *WDR88* | 216 | 3.42E-01 | 1.14E-02 | 3.19E-02 | 2.35E-01 | 3.52E-01 | rs4713534 | 6.58E-232 |  |
| UC | *XXbac-BPG181M17.5* | 322 | 8.55E-04 | 1.37E-06 | 1.30E-06 | 7.13E-03 | 1.07E-02 | rs4713534 | 6.58E-232 |  |
| UC | *XXbac-BPG246D15.9* | 322 | 3.75E-03 | 1.00E+00 | 1.00E+00 | 1.75E-05 | 2.61E-05 | rs3130612 | 8.23E-245 |  |
| UC | *XXbac-BPG300A18.12* | 322 | 8.62E-01 | 1.04E-06 | 1.58E-06 | 1.26E-54 | 1.89E-54 | rs9858213 | 2.43E-20 |  |
| UC | *XXbac-BPG32J3.22* | 322 | 5.17E-01 | 1.95E-06 | 3.02E-03 | 3.88E-34 | 5.82E-34 | rs9858213 | 2.43E-20 |  |
| UC | *ZBTB12* | 322 | 3.52E-01 | 4.71E-09 | 1.00E+00 | 1.18E-49 | 1.76E-49 | rs9858213 | 2.43E-20 |  |
| UC | *ZBTB40* | 30 | 1.80E-01 | 1.00E+00 | 1.00E+00 | 1.35E-05 | 2.04E-05 | rs3130612 | 8.23E-245 |  |
| UC | *ZBTB46* | 92 | 8.47E-01 | 1.00E+00 | 1.00E+00 | 1.35E-04 | 2.03E-04 | rs7630869 | 3.38E-17 |  |
| UC | *ZDHHC11* | 201 | 1.00E-01 | 9.23E-03 | 4.66E-02 | 1.00E+00 | 1.00E+00 | rs2239976 | 1.39E-02 | √ |
| UC | *ZDHHC11B* | 81 | 3.44E-02 | 3.37E-03 | 1.07E-02 | 1.00E+00 | 1.00E+00 | rs4713534 | 6.58E-232 |  |
| UC | *ZGPAT* | 182 | 6.68E-01 | 1.00E+00 | 1.00E+00 | 1.53E-04 | 2.29E-04 | rs3130612 | 8.23E-245 |  |
| UC | *ZPBP2* | 122 | 2.73E-01 | 6.14E-11 | 1.94E-03 | 8.91E-09 | 1.34E-08 | rs3130612 | 8.23E-245 |  |
| CD | *AAGAB* | 122 | 3.92E-01 | 1.00E+00 | 1.00E+00 | 8.84E-09 | 1.33E-08 | rs11197272 | 2.64E-04 | √ |
| CD | *ABHD16A* | 122 | 4.93E-01 | 2.17E-09 | 2.50E-03 | 9.42E-07 | 1.38E-06 | rs4947349 | 4.57E-171 |  |
| CD | *AC008697.1* | 8 | 1.21E-01 | 1.51E-06 | 4.96E-03 | 1.63E-17 | 2.45E-17 | rs7750271 | 3.06E-07 | √ |
| CD | *AC016586.1* | 21 | 3.72E-07 | 1.00E+00 | 1.00E+00 | 1.00E+00 | 1.00E+00 | rs3130612 | 8.23E-245 |  |
| CD | *AC079630.2* | 45 | 4.10E-01 | 7.78E-04 | 1.00E+00 | 2.71E-10 | 4.07E-10 | rs115991849 | 2.53E-41 |  |
| CD | *ADCY3* | 116 | 4.31E-01 | 2.58E-02 | 1.00E+00 | 1.12E-01 | 1.68E-01 | rs13119723 | 2.22E-10 |  |
| CD | *ADCY7* | 99 | 3.66E-01 | 1.00E+00 | 1.00E+00 | 1.29E-07 | 1.88E-07 | rs4713534 | 6.58E-232 |  |
| CD | *ADO* | 54 | 6.64E-02 | 3.27E-08 | 3.71E-01 | 6.28E-08 | 1.00E-07 | rs4817988 | 4.20E-15 |  |
| CD | *AGER* | 39 | 3.23E-01 | 9.05E-01 | 1.00E+00 | 1.86E-03 | 2.79E-03 | rs9858213 | 2.43E-20 |  |
| CD | *AGPAT1* | 186 | 3.60E-01 | 7.25E-01 | 1.00E+00 | 1.83E-03 | 2.75E-03 | rs45527431 | 1.12E-80 |  |
| CD | *AIF1* | 186 | 5.26E-03 | 4.07E-12 | 7.26E-07 | 1.09E-07 | 1.63E-07 | rs45527431 | 1.12E-80 |  |
| CD | *AIRE* | 10 | 4.83E-01 | 3.62E-06 | 5.58E-06 | 5.57E-09 | 8.36E-09 | rs45527431 | 1.12E-80 |  |
| CD | *AMIGO3* | 10 | 1.08E-01 | 3.76E-07 | 9.13E-07 | 2.64E-14 | 3.95E-14 | rs45527431 | 1.12E-80 |  |
| CD | *AMT* | 10 | 1.70E-13 | 5.52E-08 | 3.00E-05 | 3.69E-13 | 5.54E-13 | rs45527431 | 1.12E-80 |  |
| CD | *AP001055.6* | 10 | 3.78E-02 | 2.36E-02 | 1.00E+00 | 2.35E-09 | 3.52E-09 | rs45527431 | 1.12E-80 |  |
| CD | *AP001056.1* | 175 | 9.29E-02 | 8.72E-07 | 2.13E-06 | 5.77E-09 | 8.65E-09 | rs4713534 | 6.58E-232 |  |
| CD | *AP001058.3* | 175 | 2.11E-01 | 4.08E-08 | 1.14E-05 | 5.71E-09 | 8.56E-09 | rs117400681 | 1.44E-03 | √ |
| CD | *APEH* | 175 | 5.41E-01 | 1.33E-09 | 2.78E-07 | 1.52E-14 | 2.28E-14 | rs12363179 | 1.29E-04 | √ |
| CD | *APOBR* | 89 | 9.87E-01 | 2.14E-09 | 1.52E-09 | 1.03E-04 | 1.55E-04 | rs146484572 | 2.17E-03 | √ |
| CD | *APOM* | 31 | 9.71E-03 | 1.44E-09 | 3.79E-06 | 8.78E-07 | 1.28E-06 | rs4446955 | 5.33E-05 | √ |
| CD | *ARFRP1* | 31 | 4.41E-02 | 6.92E-02 | 6.56E-01 | 8.86E-03 | 1.33E-02 | rs3130612 | 8.23E-245 |  |
| CD | *ASH1L* | 16 | 5.48E-01 | 1.29E-02 | 4.02E-02 | 1.00E+00 | 1.00E+00 | rs9858213 | 2.43E-20 |  |
| CD | *ATF6B* | 54 | 3.01E-03 | 1.13E-05 | 5.14E-04 | 1.82E-04 | 2.73E-04 | rs148734725 | 4.98E-17 |  |
| CD | *ATG16L1* | 54 | 3.90E-03 | 1.00E+00 | 1.00E+00 | 3.62E-51 | 5.42E-51 | rs3130612 | 8.23E-245 |  |
| CD | *ATG4B* | 54 | 7.74E-02 | 4.67E-02 | 6.26E-01 | 1.00E+00 | 1.00E+00 | rs9267488 | 2.31E-236 |  |
| CD | *ATP2A1* | 54 | 1.45E-01 | 1.00E+00 | 1.00E+00 | 1.07E-02 | 1.61E-02 | rs3130612 | 8.23E-245 |  |
| CD | *ATP6V1G2* | 177 | 8.20E-03 | 4.67E-03 | 1.00E+00 | 4.22E-06 | 5.98E-06 | rs3130612 | 8.23E-245 |  |
| CD | *ATP6V1G2-DDX39B* | 274 | 4.96E-02 | 1.12E-05 | 1.00E+00 | 5.09E-07 | 7.15E-07 | rs932653 | 2.02E-05 | √ |
| CD | *ATXN2L* | 95 | 1.22E-01 | 1.00E+00 | 1.00E+00 | 7.77E-03 | 1.17E-02 | rs34762726 | 2.59E-20 |  |
| CD | *AZI1* | 129 | 2.38E-44 | 1.00E+00 | 1.00E+00 | 1.00E+00 | 1.00E+00 | rs3130612 | 8.23E-245 |  |
| CD | *BAG6* | 129 | 8.17E-03 | 1.40E-08 | 8.77E-01 | 4.95E-07 | 7.40E-07 | rs3130612 | 8.23E-245 |  |
| CD | *BRD7* | 129 | 3.33E-01 | 1.00E+00 | 1.00E+00 | 9.35E-41 | 1.40E-40 | rs3130612 | 8.23E-245 |  |
| CD | *BSN* | 160 | 5.67E-03 | 2.09E-08 | 1.05E-02 | 2.54E-14 | 3.81E-14 | rs68006638 | 4.49E-64 |  |
| CD | *C11orf30* | 160 | 1.08E-01 | 1.14E-02 | 1.00E+00 | 7.92E-16 | 1.19E-15 | rs9276348 | 9.17E-199 |  |
| CD | *C11orf9* | 117 | 4.84E-01 | 1.85E-03 | 3.19E-01 | 1.00E+00 | 1.00E+00 | rs111631632 | 9.63E-04 | √ |
| CD | *C2* | 193 | 3.55E-01 | 8.71E-04 | 2.97E-02 | 1.69E-09 | 2.54E-09 | rs12363179 | 1.29E-04 | √ |
| CD | *C21orf33* | 181 | 5.33E-02 | 8.29E-06 | 1.24E-05 | 8.37E-09 | 1.25E-08 | rs3130612 | 8.23E-245 |  |
| CD | *C3orf62* | 181 | 2.90E-01 | 5.75E-02 | 1.90E-02 | 5.05E-12 | 7.57E-12 | rs45527431 | 1.12E-80 |  |
| CD | *C4A* | 119 | 9.40E-04 | 7.39E-09 | 2.23E-08 | 1.17E-08 | 1.75E-08 | rs116747494 | 3.25E-63 |  |
| CD | *C4B* | 119 | 8.66E-02 | 5.97E-07 | 7.52E-07 | 1.04E-08 | 1.55E-08 | rs9267488 | 2.31E-236 |  |
| CD | *C5orf56* | 119 | 1.48E-01 | 6.55E-11 | 4.39E-05 | 1.04E-23 | 1.56E-23 | rs9858213 | 2.43E-20 |  |
| CD | *C5orf62* | 53 | 4.40E-01 | 1.78E-02 | 1.00E+00 | 1.26E-10 | 1.89E-10 | rs13062429 | 3.27E-17 |  |
| CD | *C6orf47* | 53 | 1.51E-02 | 4.02E-10 | 5.01E-09 | 1.23E-06 | 1.78E-06 | rs60045856 | 2.92E-97 |  |
| CD | *C6orf48* | 113 | 9.14E-01 | 4.42E-04 | 9.94E-03 | 3.36E-05 | 5.05E-05 | rs3130612 | 8.23E-245 |  |
| CD | *CA11* | 113 | 9.90E-01 | 5.44E-03 | 1.00E+00 | 1.00E+00 | 1.00E+00 | rs2596500 | 7.62E-244 |  |
| CD | *CARD6* | 113 | 8.87E-10 | 1.00E+00 | 1.00E+00 | 9.61E-01 | 1.00E+00 | rs3130612 | 8.23E-245 |  |
| CD | *CCDC101* | 166 | 5.84E-03 | 9.17E-07 | 2.20E-06 | 1.07E-03 | 1.60E-03 | rs12597418 | 1.55E-03 | √ |
| CD | *CCDC116* | 10 | 8.98E-01 | 1.00E+00 | 1.00E+00 | 7.61E-06 | 1.16E-05 | rs3130612 | 8.23E-245 |  |
| CD | *CCDC122* | 10 | 7.42E-01 | 2.13E-01 | 1.00E+00 | 3.85E-03 | 5.78E-03 | rs4713534 | 6.58E-232 |  |
| CD | *CCL1* | 10 | 5.06E-01 | 1.00E+00 | 1.00E+00 | 5.26E-07 | 7.90E-07 | rs3130612 | 8.23E-245 |  |
| CD | *CCNY* | 10 | 6.04E-01 | 3.80E-06 | 4.32E-09 | 2.40E-05 | 3.61E-05 | rs9858213 | 2.43E-20 |  |
| CD | *CCR6* | 10 | 9.42E-01 | 6.29E-02 | 1.00E+00 | 3.20E-11 | 4.80E-11 | rs6657596 | 2.70E-08 |  |
| CD | *CDC37* | 10 | 9.93E-01 | 1.00E+00 | 1.00E+00 | 3.91E-03 | 5.88E-03 | rs12363179 | 1.29E-04 | √ |
| CD | *CDC42SE2* | 10 | 5.11E-01 | 1.00E+00 | 1.00E+00 | 1.33E-07 | 2.01E-07 | rs8025237 | 2.63E-03 | √ |
| CD | *CEP76* | 10 | 3.94E-01 | 1.00E+00 | 1.00E+00 | 1.72E-05 | 2.98E-05 | rs4713534 | 6.58E-232 |  |
| CD | *CFB* | 269 | 3.22E-01 | 1.19E-07 | 4.12E-05 | 1.75E-08 | 2.62E-08 | rs2523978 | 9.69E-149 |  |
| CD | *CKB* | 206 | 7.41E-01 | 1.89E-02 | 1.04E-02 | 1.00E+00 | 1.00E+00 | rs816416 | 5.95E-05 | √ |
| CD | *CLIC1* | 206 | 3.64E-02 | 1.00E+00 | 1.00E+00 | 4.88E-07 | 7.27E-07 | rs7966763 | 1.21E-04 | √ |
| CD | *CLK2* | 206 | 4.26E-01 | 5.61E-05 | 5.72E-04 | 1.00E+00 | 1.00E+00 | rs7966763 | 1.21E-04 | √ |
| CD | *CLN3* | 206 | 3.31E-01 | 5.03E-09 | 1.71E-08 | 9.84E-05 | 1.48E-04 | rs9858213 | 2.43E-20 |  |
| CD | *CNEP1R1* | 188 | 5.74E-01 | 3.51E-02 | 1.00E+00 | 1.00E+00 | 1.00E+00 | rs3130612 | 8.23E-245 |  |
| CD | *CREM* | 256 | 4.79E-01 | 2.01E-06 | 1.67E-02 | 1.18E-05 | 1.78E-05 | rs4713534 | 6.58E-232 |  |
| CD | *CSF2* | 256 | 3.19E-02 | 1.74E-15 | 6.68E-07 | 3.95E-16 | 5.92E-16 | rs9858213 | 2.43E-20 |  |
| CD | *CSF3* | 69 | 9.04E-02 | 4.92E-08 | 3.85E-05 | 5.35E-06 | 8.08E-06 | rs2596500 | 7.62E-244 |  |
| CD | *CSNK2B* | 11 | 2.75E-02 | 1.42E-09 | 1.98E-08 | 8.50E-07 | 1.24E-06 | rs45527431 | 1.12E-80 |  |
| CD | *CTB-134H23.1* | 8 | 8.96E-02 | 3.63E-07 | 4.02E-07 | 1.60E-03 | 2.40E-03 | rs3130612 | 8.23E-245 |  |
| CD | *CTD-2330K9.3* | 227 | 3.94E-02 | 4.85E-06 | 1.01E-01 | 2.75E-03 | 4.13E-03 | rs3130612 | 8.23E-245 |  |
| CD | *CTD-3088G3.3* | 22 | 4.69E-01 | 9.38E-04 | 6.84E-04 | 1.00E+00 | 1.00E+00 | rs6657596 | 2.70E-08 |  |
| CD | *CUL2* | 33 | 6.77E-02 | 1.99E-07 | 1.04E-02 | 1.09E-05 | 1.63E-05 | rs35400317 | 3.94E-80 |  |
| CD | *CYLD* | 468 | 8.81E-06 | 1.00E+00 | 1.00E+00 | 1.24E-94 | 1.86E-94 | rs13197176 | 4.49E-100 |  |
| CD | *CYP21A2* | 468 | 4.15E-03 | 2.97E-03 | 1.00E+00 | 4.13E-09 | 6.19E-09 | rs13195402 | 1.46E-79 |  |
| CD | *DAG1* | 468 | 1.19E-07 | 2.24E-07 | 2.89E-06 | 1.67E-13 | 2.51E-13 | rs45527431 | 1.12E-80 |  |
| CD | *DAP3* | 468 | 6.71E-01 | 4.09E-02 | 1.00E+00 | 1.00E+00 | 1.00E+00 | rs45527431 | 1.12E-80 |  |
| CD | *DBP* | 468 | 6.84E-01 | 4.23E-03 | 1.25E-01 | 1.00E+00 | 1.00E+00 | rs13195402 | 1.46E-79 |  |
| CD | *DCTN4* | 348 | 2.78E-01 | 1.00E+00 | 1.00E+00 | 1.51E-09 | 2.27E-09 | rs45527431 | 1.12E-80 |  |
| CD | *DDAH2* | 271 | 1.05E-02 | 5.48E-01 | 1.00E+00 | 9.77E-06 | 1.47E-05 | rs45527431 | 1.12E-80 |  |
| CD | *DDX39B* | 384 | 1.66E-02 | 2.14E-04 | 1.00E+00 | 4.07E-06 | 5.77E-06 | rs34196306 | 6.12E-87 |  |
| CD | *DGKD* | 253 | 1.89E-04 | 1.62E-12 | 1.06E-03 | 4.30E-52 | 6.45E-52 | rs34196306 | 6.12E-87 |  |
| CD | *DHX58* | 253 | 7.99E-01 | 5.47E-03 | 5.26E-03 | 1.00E+00 | 1.00E+00 | rs13197176 | 4.49E-100 |  |
| CD | *DNAJC27-AS1* | 147 | 5.55E-01 | 2.34E-03 | 3.44E-01 | 3.19E-02 | 4.79E-02 | rs13197176 | 4.49E-100 |  |
| CD | *DNAJC28* | 62 | 8.83E-01 | 1.00E+00 | 1.00E+00 | 1.20E-04 | 1.79E-04 | rs13197176 | 4.49E-100 |  |
| CD | *DOM3Z* | 120 | 3.37E-01 | 6.71E-06 | 1.00E+00 | 1.01E-07 | 1.50E-07 | rs13197176 | 4.49E-100 |  |
| CD | *DTNB* | 120 | 5.40E-01 | 8.26E-03 | 2.83E-03 | 1.00E+00 | 1.00E+00 | rs35017208 | 3.88E-101 |  |
| CD | *DYRK3* | 56 | 7.77E-01 | 1.00E+00 | 1.00E+00 | 3.36E-04 | 5.04E-04 | rs13195402 | 1.46E-79 |  |
| CD | *EFNA3* | 100 | 7.08E-01 | 3.80E-03 | 3.38E-03 | 1.00E+00 | 1.00E+00 | rs45527431 | 1.12E-80 |  |
| CD | *EGFL8* | 206 | 2.90E-01 | 1.09E-02 | 1.00E+00 | 9.44E-04 | 1.42E-03 | rs45527431 | 1.12E-80 |  |
| CD | *EGR2* | 70 | 8.52E-01 | 1.00E+00 | 1.00E+00 | 5.31E-06 | 7.96E-06 | rs45527431 | 1.12E-80 |  |
| CD | *EHMT2* | 70 | 3.35E-01 | 1.46E-04 | 4.72E-03 | 2.83E-09 | 4.25E-09 | rs45527431 | 1.12E-80 |  |
| CD | *EIF2B4* | 70 | 9.75E-01 | 5.73E-02 | 7.17E-03 | 1.00E+00 | 1.00E+00 | rs45527431 | 1.12E-80 |  |
| CD | *EIF3C* | 70 | 3.68E-06 | 7.93E-07 | 6.63E-06 | 7.37E-03 | 1.11E-02 | rs45527431 | 1.12E-80 |  |
| CD | *EIF3CL* | 78 | 1.82E-01 | 1.20E-08 | 1.00E+00 | 1.08E-04 | 1.62E-04 | rs45527431 | 1.12E-80 |  |
| CD | *ENTPD7* | 78 | 6.32E-01 | 1.00E+00 | 1.00E+00 | 1.02E-05 | 1.53E-05 | rs34196306 | 6.12E-87 |  |
| CD | *ERAP2* | 78 | 9.53E-01 | 6.94E-01 | 1.00E+00 | 5.05E-04 | 7.61E-04 | rs34196306 | 6.12E-87 |  |
| CD | *ERBB2* | 78 | 5.75E-01 | 3.01E-06 | 3.62E-05 | 1.84E-07 | 3.13E-07 | rs13197176 | 4.49E-100 |  |
| CD | *FAM175B* | 103 | 6.58E-01 | 3.82E-02 | 3.82E-02 | 1.00E+00 | 1.00E+00 | rs13197176 | 4.49E-100 |  |
| CD | *FAM189B* | 43 | 4.19E-01 | 6.13E-06 | 8.82E-03 | 1.00E+00 | 1.00E+00 | rs13197176 | 4.49E-100 |  |
| CD | *FAM212A* | 497 | 2.71E-01 | 1.62E-07 | 4.60E-07 | 1.94E-03 | 2.92E-03 | rs35017208 | 3.88E-101 |  |
| CD | *FAM83E* | 76 | 8.14E-01 | 2.14E-03 | 3.59E-02 | 3.66E-01 | 5.50E-01 | rs13195402 | 1.46E-79 |  |
| CD | *FAM98B* | 79 | 4.58E-01 | 8.69E-04 | 1.00E+00 | 1.00E+00 | 1.00E+00 | rs13195402 | 1.46E-79 |  |
| CD | *FDPS* | 79 | 1.70E-01 | 1.32E-04 | 3.73E-03 | 1.00E+00 | 1.00E+00 | rs13195402 | 1.46E-79 |  |
| CD | *FDX1L* | 79 | 8.43E-01 | 1.00E+00 | 1.00E+00 | 4.54E-03 | 6.82E-03 | rs45527431 | 1.12E-80 |  |
| CD | *FGFR1OP* | 109 | 3.47E-01 | 3.16E-11 | 1.00E+00 | 6.82E-12 | 1.02E-11 | rs45527431 | 1.12E-80 |  |
| CD | *FKBPL* | 109 | 3.62E-04 | 1.30E-04 | 1.00E+00 | 2.07E-04 | 3.11E-04 | rs45527431 | 1.12E-80 |  |
| CD | *FNIP1* | 109 | 2.34E-02 | 1.00E+00 | 1.00E+00 | 1.04E-10 | 1.56E-10 | rs45527431 | 1.12E-80 |  |
| CD | *FUT11* | 109 | 1.47E-06 | 1.00E+00 | 1.00E+00 | 1.55E-01 | 2.33E-01 | rs13197176 | 4.49E-100 |  |
| CD | *GART* | 109 | 6.66E-01 | 1.00E+00 | 1.00E+00 | 6.96E-05 | 1.04E-04 | rs13197176 | 4.49E-100 |  |
| CD | *GBA* | 109 | 1.83E-01 | 6.54E-05 | 3.46E-04 | 1.00E+00 | 1.00E+00 | rs35017208 | 3.88E-101 |  |
| CD | *GHDC* | 109 | 7.20E-02 | 3.32E-03 | 5.05E-03 | 1.00E+00 | 1.00E+00 | rs13195402 | 1.46E-79 |  |
| CD | *GMEB2* | 109 | 1.74E-05 | 2.40E-01 | 1.00E+00 | 6.04E-03 | 9.06E-03 | rs13195402 | 1.46E-79 |  |
| CD | *GMPPB* | 200 | 9.63E-02 | 5.30E-08 | 3.80E-07 | 6.82E-14 | 1.02E-13 | rs45527431 | 1.12E-80 |  |
| CD | *GON4L* | 248 | 5.34E-01 | 9.63E-04 | 5.09E-02 | 1.00E+00 | 1.00E+00 | rs45527431 | 1.12E-80 |  |
| CD | *GOT1* | 8 | 2.98E-01 | 1.00E+00 | 1.00E+00 | 2.65E-13 | 3.98E-13 | rs45527431 | 1.12E-80 |  |
| CD | *GPANK1* | 8 | 5.84E-04 | 8.14E-07 | 2.50E-04 | 3.64E-06 | 5.28E-06 | rs45527431 | 1.12E-80 |  |
| CD | *GPSM3* | 68 | 4.56E-01 | 3.43E-03 | 1.00E+00 | 1.65E-03 | 2.47E-03 | rs34196306 | 6.12E-87 |  |
| CD | *GPX1* | 10 | 4.47E-04 | 2.18E-03 | 9.61E-02 | 4.35E-13 | 6.53E-13 | rs13197176 | 4.49E-100 |  |
| CD | *GPX3* | 139 | 7.21E-01 | 1.00E+00 | 1.00E+00 | 1.02E-04 | 1.53E-04 | rs13197176 | 4.49E-100 |  |
| CD | *GRB7* | 61 | 6.90E-01 | 1.93E-06 | 5.42E-06 | 3.39E-07 | 5.52E-07 | rs3130612 | 8.23E-245 |  |
| CD | *GSDMB* | 61 | 9.41E-01 | 2.51E-05 | 7.04E-04 | 5.91E-10 | 8.86E-10 | rs3130612 | 8.23E-245 |  |
| CD | *GTF3C2* | 61 | 7.78E-01 | 5.20E-01 | 5.05E-03 | 1.00E+00 | 1.00E+00 | rs4713534 | 6.58E-232 |  |
| CD | *HCG22* | 61 | 2.56E-02 | 2.02E-05 | 2.43E-05 | 7.72E-03 | 1.16E-02 | rs4713534 | 6.58E-232 |  |
| CD | *HCG27* | 61 | 3.90E-01 | 1.00E+00 | 1.00E+00 | 1.90E-02 | 2.85E-02 | rs9276348 | 9.17E-199 |  |
| CD | *HCN3* | 61 | 3.13E-01 | 3.66E-05 | 4.80E-05 | 1.00E+00 | 1.00E+00 | rs4713534 | 6.58E-232 |  |
| CD | *HEATR3* | 61 | 8.56E-01 | 1.97E-03 | 1.18E-02 | 8.87E-01 | 1.00E+00 | rs9276348 | 9.17E-199 |  |
| CD | *HIC2* | 61 | 4.26E-11 | 8.61E-02 | 8.49E-02 | 1.00E+00 | 1.00E+00 | rs9276348 | 9.17E-199 |  |
| CD | *HINT1* | 22 | 9.27E-03 | 3.11E-04 | 2.60E-01 | 2.36E-06 | 3.54E-06 | rs4713534 | 6.58E-232 |  |
| CD | *HLA-B* | 22 | 5.81E-01 | 4.15E-02 | 1.06E-01 | 1.20E-03 | 1.85E-03 | rs4713534 | 6.58E-232 |  |
| CD | *HLA-C* | 22 | 9.12E-01 | 6.75E-01 | 1.00E+00 | 8.95E-03 | 1.25E-02 | rs4713534 | 6.58E-232 |  |
| CD | *HLA-DOB* | 22 | 2.78E-01 | 2.28E-01 | 1.00E+00 | 3.79E-02 | 5.68E-02 | rs4713534 | 6.58E-232 |  |
| CD | *HLA-DQA2* | 107 | 9.08E-02 | 2.02E-04 | 1.91E-01 | 3.80E-02 | 5.69E-02 | rs4713534 | 6.58E-232 |  |
| CD | *HLA-DQB2* | 107 | 1.58E-01 | 6.91E-03 | 6.65E-01 | 1.30E-02 | 1.95E-02 | rs4713534 | 6.58E-232 |  |
| CD | *HSD17B1* | 216 | 1.83E-06 | 1.00E+00 | 1.00E+00 | 1.00E+00 | 1.00E+00 | rs4713534 | 6.58E-232 |  |
| CD | *HSPA1A* | 216 | 3.39E-01 | 1.00E+00 | 1.00E+00 | 2.05E-02 | 3.07E-02 | rs2517617 | 5.01E-152 |  |
| CD | *HSPA1B* | 216 | 6.90E-01 | 2.24E-04 | 1.27E-03 | 3.55E-05 | 5.34E-05 | rs2517617 | 5.01E-152 |  |
| CD | *HSPA1L* | 216 | 6.06E-01 | 4.28E-02 | 7.53E-01 | 2.03E-01 | 3.05E-01 | rs77952682 | 7.42E-04 | √ |
| CD | *HSPB9* | 268 | 6.96E-01 | 7.12E-03 | 5.47E-03 | 1.00E+00 | 1.00E+00 | rs45527431 | 1.12E-80 |  |
| CD | *ICAM1* | 19 | 8.19E-01 | 1.00E+00 | 1.00E+00 | 8.08E-03 | 1.21E-02 | rs9276348 | 9.17E-199 |  |
| CD | *ICAM3* | 19 | 9.31E-01 | 1.00E+00 | 1.00E+00 | 3.27E-03 | 4.93E-03 | rs3130612 | 8.23E-245 |  |
| CD | *ICAM4* | 256 | 6.99E-01 | 1.00E+00 | 1.00E+00 | 3.22E-03 | 4.84E-03 | rs3130612 | 8.23E-245 |  |
| CD | *ICAM5* | 256 | 4.44E-01 | 1.00E+00 | 1.00E+00 | 6.36E-03 | 9.56E-03 | rs3130612 | 8.23E-245 |  |
| CD | *ICOSLG* | 256 | 4.95E-01 | 5.46E-07 | 2.97E-06 | 6.19E-09 | 9.29E-09 | rs4147359 | 4.06E-13 |  |
| CD | *IFNAR1* | 256 | 9.91E-01 | 1.00E+00 | 1.00E+00 | 4.11E-03 | 6.16E-03 | rs4147359 | 4.06E-13 |  |
| CD | *IFNGR2* | 256 | 6.43E-01 | 1.00E+00 | 1.00E+00 | 3.19E-04 | 4.78E-04 | rs13062429 | 3.27E-17 |  |
| CD | *IKZF3* | 256 | 6.22E-01 | 7.60E-11 | 2.16E-08 | 3.00E-09 | 4.50E-09 | rs9858213 | 2.43E-20 |  |
| CD | *IL10* | 119 | 9.10E-02 | 1.00E+00 | 1.00E+00 | 9.08E-04 | 1.36E-03 | rs7621347 | 5.12E-08 | √ |
| CD | *IL10RB* | 184 | 6.51E-01 | 1.00E+00 | 1.00E+00 | 4.82E-02 | 7.24E-02 | rs73408005 | 1.29E-39 |  |
| CD | *IL12B* | 41 | 6.00E-02 | 7.95E-06 | 9.53E-05 | 1.04E-16 | 1.56E-16 | rs80204008 | 1.34E-03 | √ |
| CD | *IL12RB2* | 8 | 1.07E-04 | 2.38E-53 | 2.44E-08 | 2.60E-87 | 3.89E-87 | rs13119723 | 2.22E-10 |  |
| CD | *IL18R1* | 3 | 9.22E-01 | 1.00E+00 | 1.00E+00 | 2.19E-02 | 3.28E-02 | rs111631632 | 9.63E-04 | √ |
| CD | *IL18RAP* | 3 | 9.62E-02 | 1.00E+00 | 1.00E+00 | 3.75E-03 | 5.62E-03 | rs2901182 | 5.70E-06 | √ |
| CD | *IL19* | 19 | 9.96E-02 | 1.00E+00 | 1.00E+00 | 3.65E-03 | 5.48E-03 | rs10882105 | 1.18E-04 | √ |
| CD | *IL23R* | 19 | 3.00E-02 | 1.76E-73 | 2.01E-65 | 7.40E-86 | 1.11E-85 | rs56098412 | 9.98E-84 |  |
| CD | *IL24* | 19 | 3.53E-01 | 4.03E-03 | 3.65E-03 | 1.00E+00 | 1.00E+00 | rs34762726 | 2.59E-20 |  |
| CD | *IL27* | 19 | 2.09E-02 | 2.72E-06 | 4.24E-06 | 2.77E-03 | 4.15E-03 | rs7966763 | 1.21E-04 | √ |
| CD | *IL4* | 19 | 4.45E-01 | 1.59E-02 | 7.06E-01 | 1.00E+00 | 1.00E+00 | rs1873625 | 2.82E-19 |  |
| CD | *INPP5D* | 19 | 2.60E-01 | 1.00E+00 | 1.00E+00 | 5.38E-51 | 8.07E-51 | rs35283694 | 4.20E-30 |  |
| CD | *IP6K1* | 19 | 1.88E-01 | 8.79E-14 | 1.39E-10 | 2.05E-14 | 3.08E-14 | rs34196306 | 6.12E-87 |  |
| CD | *IQCH* | 19 | 9.99E-01 | 1.00E+00 | 1.00E+00 | 1.43E-06 | 2.14E-06 | rs3130612 | 8.23E-245 |  |
| CD | *IRF1* | 18 | 4.47E-01 | 2.21E-14 | 5.23E-09 | 1.62E-24 | 2.43E-24 | rs17526722 | 1.29E-64 |  |
| CD | *IRGM* | 18 | 8.21E-01 | 1.00E+00 | 1.00E+00 | 6.19E-10 | 9.28E-10 | rs3130612 | 8.23E-245 |  |
| CD | *JAK2* | 18 | 8.56E-01 | 2.98E-10 | 3.49E-09 | 1.47E-06 | 2.24E-06 | rs3130612 | 8.23E-245 |  |
| CD | *KAT2A* | 18 | 8.08E-01 | 6.22E-03 | 5.36E-03 | 1.00E+00 | 1.00E+00 | rs3130612 | 8.23E-245 |  |
| CD | *KCNH4* | 18 | 5.67E-02 | 2.23E-03 | 8.30E-03 | 1.00E+00 | 1.00E+00 | rs3130612 | 8.23E-245 |  |
| CD | *KEAP1* | 18 | 9.16E-01 | 1.00E+00 | 1.00E+00 | 4.99E-02 | 7.48E-02 | rs3130612 | 8.23E-245 |  |
| CD | *KIAA1841* | 18 | 3.70E-01 | 1.00E+00 | 1.00E+00 | 1.71E-03 | 2.56E-03 | rs3130612 | 8.23E-245 |  |
| CD | *KRTCAP2* | 18 | 2.28E-01 | 2.50E-03 | 1.00E+00 | 1.00E+00 | 1.00E+00 | rs12597418 | 1.55E-03 | √ |
| CD | *LACC1* | 40 | 5.33E-01 | 1.00E+00 | 1.00E+00 | 1.25E-02 | 1.87E-02 | rs3130612 | 8.23E-245 |  |
| CD | *LIME1* | 40 | 1.85E-01 | 1.00E+00 | 1.00E+00 | 3.38E-03 | 5.07E-03 | rs3130612 | 8.23E-245 |  |
| CD | *LINC00481* | 20 | 3.11E-01 | 1.00E+00 | 1.00E+00 | 1.51E-02 | 2.27E-02 | rs6657596 | 2.70E-08 |  |
| CD | *LNPEP* | 89 | 4.18E-01 | 1.34E-01 | 1.00E+00 | 7.28E-04 | 1.09E-03 | rs34434446 | 1.24E-38 |  |
| CD | *LOC678655* | 12 | 9.57E-01 | 4.69E-02 | 1.00E+00 | 1.00E+00 | 1.00E+00 | rs9858213 | 2.43E-20 |  |
| CD | *LRRC32* | 12 | 1.08E-01 | 2.27E-04 | 3.20E-03 | 1.50E-15 | 2.25E-15 | rs816416 | 5.95E-05 | √ |
| CD | *LRRK2* | 12 | 2.87E-01 | 1.60E-16 | 1.61E-16 | 9.04E-13 | 1.36E-12 | rs3130612 | 8.23E-245 |  |
| CD | *LSM2* | 221 | 1.51E-01 | 2.47E-05 | 6.84E-04 | 3.11E-06 | 4.66E-06 | rs3130612 | 8.23E-245 |  |
| CD | *LST1* | 221 | 1.41E-02 | 2.20E-07 | 3.91E-04 | 8.28E-06 | 1.18E-05 | rs9858213 | 2.43E-20 |  |
| CD | *LTA* | 41 | 5.58E-03 | 2.92E-09 | 1.00E+00 | 6.69E-06 | 9.49E-06 | rs9858213 | 2.43E-20 |  |
| CD | *LTB* | 32 | 1.35E-02 | 1.26E-07 | 6.94E-02 | 2.66E-06 | 3.77E-06 | rs920200 | 1.03E-03 | √ |
| CD | *LY6G5B* | 32 | 1.92E-03 | 8.89E-10 | 4.62E-07 | 3.72E-07 | 5.52E-07 | rs8067912 | 3.78E-05 | √ |
| CD | *LY6G5C* | 32 | 2.08E-01 | 1.78E-09 | 4.61E-05 | 3.81E-07 | 5.64E-07 | rs3130612 | 8.23E-245 |  |
| CD | *LYRM7* | 104 | 7.26E-03 | 5.10E-04 | 2.74E-03 | 7.73E-09 | 1.16E-08 | rs7621347 | 5.12E-08 | √ |
| CD | *MAPKAPK2* | 75 | 1.53E-01 | 1.00E+00 | 1.00E+00 | 4.73E-04 | 7.09E-04 | rs3130612 | 8.23E-245 |  |
| CD | *MARK3* | 8 | 2.69E-01 | 4.90E-02 | 2.29E-01 | 1.00E+00 | 1.00E+00 | rs13062429 | 3.27E-17 |  |
| CD | *MED24* | 8 | 7.59E-01 | 1.37E-04 | 1.36E-01 | 2.71E-05 | 4.09E-05 | rs148306918 | 1.16E-04 | √ |
| CD | *METTL10* | 8 | 4.88E-01 | 7.39E-02 | 4.32E-02 | 1.00E+00 | 1.00E+00 | rs3130612 | 8.23E-245 |  |
| CD | *MICA* | 8 | 3.99E-01 | 1.05E-04 | 5.74E-03 | 6.33E-05 | 9.84E-05 | rs3130612 | 8.23E-245 |  |
| CD | *MICB* | 8 | 1.36E-02 | 1.25E-07 | 2.58E-02 | 1.80E-06 | 2.55E-06 | rs9858213 | 2.43E-20 |  |
| CD | *MIEN1* | 8 | 7.19E-01 | 1.74E-02 | 1.00E+00 | 3.22E-07 | 5.52E-07 | rs1238262 | 2.37E-110 |  |
| CD | *MIER1* | 8 | 7.46E-01 | 2.10E-11 | 7.74E-01 | 1.57E-24 | 2.36E-24 | rs73437980 | 8.53E-05 | √ |
| CD | *MON1A* | 8 | 1.40E-01 | 1.03E-05 | 1.00E+00 | 4.93E-03 | 7.40E-03 | rs4713534 | 6.58E-232 |  |
| CD | *MSH5* | 8 | 2.32E-02 | 2.04E-01 | 1.00E+00 | 4.74E-08 | 7.52E-08 | rs35017208 | 3.88E-101 |  |
| CD | *MSH5-SAPCD1* | 7 | 3.30E-02 | 1.00E+00 | 1.00E+00 | 1.88E-07 | 2.88E-07 | rs7630869 | 3.38E-17 |  |
| CD | *MST1* | 7 | 6.49E-02 | 5.43E-06 | 1.11E-01 | 1.63E-14 | 2.45E-14 | rs6657596 | 2.70E-08 |  |
| CD | *MST1R* | 17 | 3.37E-05 | 1.49E-06 | 3.18E-01 | 1.69E-03 | 2.54E-03 | rs4713534 | 6.58E-232 |  |
| CD | *MTX1* | 17 | 8.94E-01 | 2.25E-04 | 1.82E-02 | 1.00E+00 | 1.00E+00 | rs2901182 | 5.70E-06 | √ |
| CD | *MUC1* | 8 | 5.66E-01 | 1.22E-03 | 7.74E-01 | 1.00E+00 | 1.00E+00 | rs7758736 | 5.32E-166 |  |
| CD | *MUC19* | 8 | 2.93E-01 | 1.66E-02 | 1.00E+00 | 1.32E-11 | 1.99E-11 | rs1238262 | 2.37E-110 |  |
| CD | *MUC22* | 8 | 5.63E-02 | 1.00E+00 | 1.00E+00 | 2.92E-02 | 4.38E-02 | rs56098412 | 9.98E-84 |  |
| CD | *MX2* | 8 | 9.07E-01 | 1.00E+00 | 1.00E+00 | 6.90E-06 | 1.04E-05 | rs6657596 | 2.70E-08 |  |
| CD | *NCKIPSD* | 8 | 2.48E-01 | 2.67E-04 | 1.00E+00 | 1.00E+00 | 1.00E+00 | rs13217620 | 3.58E-92 |  |
| CD | *NCR3* | 8 | 1.13E-02 | 2.71E-03 | 1.00E+00 | 6.84E-06 | 9.71E-06 | rs3130612 | 8.23E-245 |  |
| CD | *NDST2* | 8 | 8.82E-01 | 1.65E-03 | 1.00E+00 | 5.81E-02 | 8.71E-02 | rs4713534 | 6.58E-232 |  |
| CD | *NEU1* | 8 | 2.99E-01 | 9.45E-03 | 1.00E+00 | 7.85E-09 | 1.18E-08 | rs35446411 | 3.23E-09 |  |
| CD | *NFKBIL1* | 8 | 1.72E-03 | 3.05E-12 | 2.70E-09 | 3.42E-06 | 4.85E-06 | rs3130612 | 8.23E-245 |  |
| CD | *NICN1* | 8 | 7.53E-11 | 6.34E-07 | 1.02E-04 | 5.03E-13 | 7.55E-13 | rs4713534 | 6.58E-232 |  |
| CD | *NKD1* | 8 | 4.14E-21 | 2.83E-42 | 7.84E-20 | 2.04E-98 | 3.06E-98 | rs13217620 | 3.58E-92 |  |
| CD | *NKIRAS2* | 8 | 7.35E-01 | 3.05E-03 | 1.55E-03 | 1.00E+00 | 1.00E+00 | rs4713534 | 6.58E-232 |  |
| CD | *NOD2* | 8 | 2.34E-12 | 1.00E+00 | 1.00E+00 | 4.75E-96 | 7.13E-96 | rs4713534 | 6.58E-232 |  |
| CD | *NOTCH4* | 7 | 9.32E-01 | 2.55E-01 | 1.00E+00 | 2.51E-03 | 3.76E-03 | rs4817988 | 4.20E-15 |  |
| CD | *NPIPL1* | 7 | 5.09E-01 | 5.25E-09 | 9.90E-09 | 1.09E-04 | 1.64E-04 | rs3130612 | 8.23E-245 |  |
| CD | *ORMDL3* | 7 | 8.81E-01 | 9.33E-13 | 8.74E-09 | 9.12E-10 | 1.37E-09 | rs2901182 | 5.70E-06 | √ |
| CD | *P4HA2* | 8 | 6.30E-01 | 6.76E-07 | 1.89E-04 | 1.44E-23 | 2.16E-23 | rs13064576 | 5.94E-19 |  |
| CD | *PAPD5* | 8 | 7.97E-01 | 1.00E+00 | 1.00E+00 | 7.17E-09 | 1.08E-08 | rs35999162 | 7.48E-18 |  |
| CD | *PBX2* | 8 | 7.38E-01 | 1.15E-01 | 1.00E+00 | 3.12E-03 | 4.68E-03 | rs4147359 | 4.06E-13 |  |
| CD | *PDE4A* | 8 | 3.27E-01 | 1.00E+00 | 1.00E+00 | 1.26E-02 | 1.88E-02 | rs9858213 | 2.43E-20 |  |
| CD | *PDGFB* | 8 | 4.37E-01 | 1.00E+00 | 1.00E+00 | 6.91E-11 | 1.04E-10 | rs9858213 | 2.43E-20 |  |
| CD | *PDLIM4* | 8 | 4.13E-01 | 2.84E-07 | 1.31E-04 | 1.25E-22 | 1.87E-22 | rs3130612 | 8.23E-245 |  |
| CD | *PEX13* | 7 | 6.50E-01 | 4.84E-05 | 3.22E-04 | 5.91E-04 | 8.87E-04 | rs1054684 | 4.31E-152 |  |
| CD | *PFKL* | 7 | 1.32E-01 | 1.00E+00 | 1.00E+00 | 1.00E-06 | 1.50E-06 | rs9858213 | 2.43E-20 |  |
| CD | *PGAP3* | 17 | 1.36E-01 | 1.39E-02 | 1.00E+00 | 5.87E-07 | 1.03E-06 | rs9276348 | 9.17E-199 |  |
| CD | *PHTF1* | 17 | 8.90E-01 | 1.00E+00 | 1.00E+00 | 2.65E-03 | 4.05E-03 | rs9858213 | 2.43E-20 |  |
| CD | *PKLR* | 17 | 5.90E-01 | 3.43E-04 | 2.08E-03 | 1.00E+00 | 1.00E+00 | rs4713534 | 6.58E-232 |  |
| CD | *PLCB3* | 17 | 3.98E-01 | 4.49E-02 | 7.40E-02 | 1.00E+00 | 1.00E+00 | rs35017208 | 3.88E-101 |  |
| CD | *PLCL1* | 8 | 6.62E-01 | 1.90E-02 | 2.50E-02 | 1.00E+00 | 1.00E+00 | rs2239976 | 1.39E-02 | √ |
| CD | *PLEKHH2* | 8 | 9.48E-01 | 6.43E-04 | 2.33E-02 | 2.64E-04 | 3.97E-04 | rs816416 | 5.95E-05 | √ |
| CD | *POU5F1* | 8 | 1.56E-02 | 1.94E-02 | 2.53E-01 | 3.63E-02 | 5.45E-02 | rs4548024 | 9.63E-08 | √ |
| CD | *PPIF* | 8 | 6.94E-01 | 2.12E-05 | 7.99E-09 | 1.05E-04 | 1.57E-04 | rs143449347 | 1.12E-03 | √ |
| CD | *PPIL2* | 8 | 8.92E-01 | 1.00E+00 | 1.00E+00 | 6.84E-06 | 1.05E-05 | rs66462181 | 9.20E-81 |  |
| CD | *PPM1G* | 8 | 4.26E-01 | 1.15E-03 | 3.66E-03 | 2.03E-01 | 3.05E-01 | rs6657596 | 2.70E-08 |  |
| CD | *PPT2* | 7 | 6.19E-02 | 1.37E-04 | 2.55E-01 | 1.75E-04 | 2.62E-04 | rs2239976 | 1.39E-02 | √ |
| CD | *PRDM1* | 7 | 9.42E-01 | 1.00E+00 | 1.00E+00 | 5.27E-05 | 7.91E-05 | rs4947349 | 4.57E-171 |  |
| CD | *PRKAA1* | 8 | 5.63E-05 | 1.00E+00 | 1.00E+00 | 1.32E-04 | 1.98E-04 | rs9276348 | 9.17E-199 |  |
| CD | *PRRC2A* | 8 | 1.08E-03 | 2.37E-10 | 1.44E-08 | 5.47E-07 | 8.15E-07 | rs3130612 | 8.23E-245 |  |
| CD | *PRRT1* | 18 | 4.61E-04 | 1.00E+00 | 1.00E+00 | 3.70E-04 | 5.55E-04 | rs3117145 | 2.97E-112 |  |
| CD | *PSMB8* | 18 | 3.78E-01 | 9.16E-03 | 4.11E-01 | 2.34E-02 | 3.51E-02 | rs73437980 | 8.53E-05 | √ |
| CD | *PSMB9* | 16 | 2.27E-01 | 7.29E-07 | 7.25E-02 | 1.48E-03 | 2.21E-03 | rs3130612 | 8.23E-245 |  |
| CD | *PSMD3* | 16 | 4.32E-01 | 1.60E-12 | 8.39E-15 | 2.27E-10 | 3.40E-10 | rs148734725 | 4.98E-17 |  |
| CD | *PSMG2* | 8 | 8.52E-01 | 1.00E+00 | 1.00E+00 | 8.62E-07 | 1.29E-06 | rs7621347 | 5.12E-08 | √ |
| CD | *PSORS1C1* | 8 | 2.29E-02 | 2.08E-02 | 1.00E+00 | 9.41E-04 | 1.41E-03 | rs9276348 | 9.17E-199 |  |
| CD | *PTGER4* | 8 | 3.55E-03 | 1.00E+00 | 1.00E+00 | 4.50E-19 | 6.75E-19 | rs3130612 | 8.23E-245 |  |
| CD | *PTPN2* | 7 | 3.34E-01 | 1.47E-09 | 9.82E-05 | 7.68E-08 | 1.13E-07 | rs116747494 | 3.25E-63 |  |
| CD | *PTPN22* | 7 | 7.87E-01 | 7.41E-01 | 9.31E-01 | 3.50E-02 | 5.33E-02 | rs4713534 | 6.58E-232 |  |
| CD | *PUS10* | 7 | 3.66E-01 | 5.06E-05 | 8.17E-04 | 4.31E-04 | 6.47E-04 | rs4713534 | 6.58E-232 |  |
| CD | *PWP2* | 18 | 1.23E-02 | 3.93E-07 | 2.33E-06 | 4.98E-09 | 7.47E-09 | rs1054684 | 4.31E-152 |  |
| CD | *RAB5C* | 18 | 5.67E-01 | 6.06E-03 | 7.77E-03 | 1.00E+00 | 1.00E+00 | rs3130612 | 8.23E-245 |  |
| CD | *RABEP2* | 18 | 5.82E-01 | 1.00E+00 | 1.00E+00 | 4.32E-02 | 6.48E-02 | rs9858213 | 2.43E-20 |  |
| CD | *RAD50* | 8 | 4.62E-01 | 5.16E-04 | 7.03E-02 | 3.62E-23 | 5.43E-23 | rs10958810 | 4.04E-05 | √ |
| CD | *RAPGEF6* | 8 | 3.30E-02 | 2.15E-02 | 1.00E+00 | 4.42E-12 | 6.62E-12 | rs10982508 | 2.11E-04 | √ |
| CD | *RASIP1* | 8 | 5.86E-01 | 1.90E-02 | 1.13E-01 | 6.03E-01 | 9.04E-01 | rs3130612 | 8.23E-245 |  |
| CD | *RAVER1* | 7 | 5.83E-01 | 1.00E+00 | 1.00E+00 | 7.41E-03 | 1.12E-02 | rs6657596 | 2.70E-08 |  |
| CD | *RBM22* | 7 | 8.28E-01 | 1.00E+00 | 1.00E+00 | 6.32E-09 | 9.48E-09 | rs9267488 | 2.31E-236 |  |
| CD | *RBM6* | 7 | 2.47E-01 | 3.18E-02 | 4.09E-01 | 6.04E-03 | 9.06E-03 | rs9858213 | 2.43E-20 |  |
| CD | *RDBP* | 7 | 9.80E-02 | 7.54E-10 | 2.83E-09 | 4.13E-08 | 6.27E-08 | rs442694 | 3.90E-122 |  |
| CD | *REL* | 7 | 5.49E-01 | 1.82E-03 | 8.07E-02 | 5.06E-04 | 7.60E-04 | rs13195402 | 1.46E-79 |  |
| CD | *RHOA* | 7 | 1.12E-08 | 3.69E-05 | 5.57E-03 | 5.84E-13 | 8.76E-13 | rs6657596 | 2.70E-08 |  |
| CD | *RIMBP3B* | 7 | 5.62E-12 | 7.64E-02 | 8.14E-02 | 1.00E+00 | 1.00E+00 | rs9858213 | 2.43E-20 |  |
| CD | *RIMBP3C* | 16 | 1.76E-01 | 1.00E+00 | 1.00E+00 | 1.52E-06 | 2.34E-06 | rs7621347 | 5.12E-08 | √ |
| CD | *RIT1* | 16 | 5.08E-01 | 5.93E-04 | 4.75E-01 | 1.00E+00 | 1.00E+00 | rs9833611 | 3.12E-19 |  |
| CD | *RLN1* | 7 | 3.17E-01 | 4.01E-03 | 1.00E+00 | 4.38E-04 | 6.57E-04 | rs9858213 | 2.43E-20 |  |
| CD | *RLN2* | 7 | 4.40E-01 | 1.00E+00 | 1.00E+00 | 3.75E-02 | 5.62E-02 | rs3130612 | 8.23E-245 |  |
| CD | *RNASET2* | 8 | 6.42E-01 | 1.65E-05 | 1.00E+00 | 7.27E-12 | 1.09E-11 | rs2596500 | 7.62E-244 |  |
| CD | *RNF123* | 8 | 2.10E-02 | 2.63E-10 | 3.30E-09 | 3.14E-14 | 4.70E-14 | rs9276550 | 2.32E-183 |  |
| CD | *RNF5* | 7 | 4.90E-01 | 1.59E-01 | 1.00E+00 | 2.25E-03 | 3.38E-03 | rs3130612 | 8.23E-245 |  |
| CD | *RP11-1152H15.1* | 7 | 3.65E-01 | 1.00E+00 | 1.00E+00 | 1.04E-02 | 1.56E-02 | rs4947349 | 4.57E-171 |  |
| CD | *RP11-1348G14.2* | 8 | 1.07E-16 | 9.64E-07 | 3.25E-06 | 1.26E-02 | 1.89E-02 | rs7630869 | 3.38E-17 |  |
| CD | *RP11-1348G14.4* | 8 | 6.85E-02 | 2.19E-03 | 1.00E+00 | 8.90E-03 | 1.34E-02 | rs4713534 | 6.58E-232 |  |
| CD | *RP11-206L10.3* | 8 | 2.26E-06 | 1.00E+00 | 1.00E+00 | 1.00E+00 | 1.00E+00 | rs4713534 | 6.58E-232 |  |
| CD | *RP11-29H23.1* | 8 | 1.94E-10 | 1.00E+00 | 1.00E+00 | 1.00E+00 | 1.00E+00 | rs4713534 | 6.58E-232 |  |
| CD | *RP11-401P9.4* | 8 | 1.70E-19 | 2.74E-36 | 1.83E-15 | 1.26E-98 | 1.88E-98 | rs3130612 | 8.23E-245 |  |
| CD | *RP11-476D10.1* | 8 | 8.06E-01 | 7.30E-02 | 1.00E+00 | 2.17E-09 | 3.26E-09 | rs3130612 | 8.23E-245 |  |
| CD | *RP11-514O12.4* | 10 | 7.99E-01 | 1.11E-04 | 1.00E+00 | 4.45E-12 | 6.67E-12 | rs60045856 | 2.92E-97 |  |
| CD | *RP4-697K14.7* | 10 | 5.68E-04 | 9.20E-08 | 4.83E-07 | 2.19E-03 | 3.29E-03 | rs116747494 | 3.25E-63 |  |
| CD | *RP5-1073O3.5* | 10 | 2.46E-01 | 4.29E-01 | 8.58E-01 | 2.19E-02 | 3.34E-02 | rs1238262 | 2.37E-110 |  |
| CD | *RPL18* | 17 | 6.81E-01 | 2.46E-02 | 1.22E-01 | 1.00E+00 | 1.00E+00 | rs1238262 | 2.37E-110 |  |
| CD | *RPL3* | 17 | 4.08E-01 | 1.00E+00 | 1.00E+00 | 6.99E-11 | 1.05E-10 | rs35017208 | 3.88E-101 |  |
| CD | *RPL37* | 7 | 3.56E-14 | 1.00E+00 | 1.00E+00 | 1.00E+00 | 1.00E+00 | rs13199649 | 5.37E-97 |  |
| CD | *RPS6KA2* | 7 | 5.70E-01 | 1.00E+00 | 1.00E+00 | 1.50E-10 | 2.26E-10 | rs34724414 | 1.15E-101 |  |
| CD | *RSBN1* | 8 | 1.33E-01 | 5.92E-01 | 9.05E-01 | 4.29E-04 | 6.54E-04 | rs35243068 | 3.34E-105 |  |
| CD | *RSPH3* | 8 | 9.24E-01 | 1.23E-02 | 2.83E-02 | 3.57E-01 | 5.35E-01 | rs2746150 | 3.29E-123 |  |
| CD | *RTEL1* | 7 | 4.01E-02 | 4.12E-03 | 1.26E-01 | 1.71E-03 | 2.57E-03 | rs66462181 | 9.20E-81 |  |
| CD | *RTEL1* | 7 | 4.01E-02 | 4.12E-03 | 1.26E-01 | 1.71E-03 | 2.57E-03 | rs1238262 | 2.37E-110 |  |
| CD | *RTEL1* | 8 | 2.48E-02 | 1.34E-02 | 1.00E+00 | 2.63E-03 | 3.94E-03 | rs13199649 | 5.37E-97 |  |
| CD | *RTEL1* | 8 | 2.48E-02 | 1.34E-02 | 1.00E+00 | 2.63E-03 | 3.94E-03 | rs816416 | 5.95E-05 | √ |
| CD | *RUSC1* | 17 | 4.23E-01 | 1.05E-03 | 2.31E-03 | 1.00E+00 | 1.00E+00 | rs1238262 | 2.37E-110 |  |
| CD | *SAPCD1* | 17 | 1.54E-01 | 1.00E+00 | 1.00E+00 | 1.17E-06 | 1.76E-06 | rs35017208 | 3.88E-101 |  |
| CD | *SCAMP3* | 8 | 9.28E-01 | 1.58E-04 | 2.15E-03 | 1.00E+00 | 1.00E+00 | rs1238262 | 2.37E-110 |  |
| CD | *SDF2L1* | 8 | 9.89E-01 | 1.00E+00 | 1.00E+00 | 1.43E-05 | 2.21E-05 | rs9296009 | 1.00E-250 |  |
| CD | *SEC24C* | 8 | 2.06E-06 | 1.00E+00 | 1.00E+00 | 1.97E-01 | 2.96E-01 | rs7247290 | 1.40E-03 | √ |
| CD | *SEH1L* | 8 | 9.37E-01 | 1.00E+00 | 1.00E+00 | 1.37E-06 | 2.06E-06 | rs9296009 | 1.00E-250 |  |
| CD | *SERBP1* | 8 | 2.03E-01 | 1.00E+00 | 1.00E+00 | 1.65E-02 | 2.47E-02 | rs9296009 | 1.00E-250 |  |
| CD | *SH2B1* | 7 | 4.09E-01 | 1.00E+00 | 1.00E+00 | 1.47E-02 | 2.20E-02 | rs34562262 | 9.60E-215 |  |
| CD | *SKIV2L* | 7 | 6.32E-01 | 6.38E-06 | 2.25E-02 | 7.35E-09 | 1.10E-08 | rs58352886 | 2.00E-03 | √ |
| CD | *SLC22A4* | 7 | 7.16E-01 | 1.51E-11 | 5.04E-01 | 1.01E-23 | 1.51E-23 | rs35590487 | 4.20E-03 | √ |
| CD | *SLC22A5* | 155 | 2.05E-02 | 5.78E-21 | 9.14E-15 | 2.12E-24 | 3.18E-24 | rs9296009 | 1.00E-250 |  |
| CD | *SLC25A28* | 155 | 4.08E-01 | 1.00E+00 | 1.00E+00 | 4.75E-15 | 7.13E-15 | rs12764378 | 1.90E-13 |  |
| CD | *SLC2A13* | 155 | 9.04E-01 | 2.50E-05 | 1.17E-01 | 1.55E-06 | 2.28E-06 | rs58352886 | 2.00E-03 | √ |
| CD | *SLC2A4RG* | 105 | 2.60E-01 | 1.00E+00 | 1.00E+00 | 9.76E-03 | 1.46E-02 | rs9296009 | 1.00E-250 |  |
| CD | *SLC35D1* | 105 | 9.48E-02 | 9.18E-51 | 9.90E-53 | 1.86E-39 | 2.79E-39 | rs7488113 | 4.90E-04 | √ |
| CD | *SMAD3* | 105 | 7.90E-01 | 1.00E+00 | 1.00E+00 | 1.55E-06 | 2.33E-06 | rs12539741 | 1.20E-11 |  |
| CD | *SNX17* | 105 | 9.39E-01 | 3.07E-02 | 2.15E-03 | 1.00E+00 | 1.00E+00 | rs2844454 | 3.40E-205 |  |
| CD | *SNX20* | 105 | 1.22E-20 | 3.36E-37 | 1.04E-23 | 1.13E-98 | 1.70E-98 | rs2844454 | 3.40E-205 |  |
| CD | *SP110* | 105 | 6.69E-01 | 1.00E+00 | 1.00E+00 | 4.30E-02 | 6.45E-02 | rs12126142 | 4.50E-06 | √ |
| CD | *SPHK2* | 105 | 7.68E-01 | 9.65E-03 | 9.82E-03 | 1.00E+00 | 1.00E+00 | rs3819721 | 1.20E-242 |  |
| CD | *SRMS* | 105 | 6.63E-02 | 1.03E-04 | 1.99E-01 | 1.51E-02 | 2.27E-02 | rs73222264 | 1.80E-03 | √ |
| CD | *STARD3* | 142 | 4.45E-01 | 2.83E-01 | 1.00E+00 | 3.03E-06 | 5.34E-06 | rs9296009 | 1.00E-250 |  |
| CD | *STAT3* | 142 | 7.10E-02 | 2.28E-02 | 1.00E+00 | 3.79E-02 | 5.69E-02 | rs41270518 | 3.10E-115 |  |
| CD | *STAT5B* | 142 | 3.65E-01 | 9.85E-07 | 1.92E-02 | 1.02E-01 | 1.53E-01 | rs71363730 | 3.40E-04 | √ |
| CD | *STK19* | 142 | 3.25E-01 | 8.85E-08 | 9.33E-04 | 1.81E-08 | 2.71E-08 | rs62321692 | 3.20E-06 | √ |
| CD | *STMN3* | 142 | 3.14E-03 | 3.59E-09 | 2.59E-08 | 1.70E-03 | 2.55E-03 | rs10790268 | 3.30E-15 |  |
| CD | *SULT1A1* | 142 | 2.07E-08 | 9.59E-07 | 1.71E-06 | 7.18E-03 | 1.08E-02 | rs9269037 | 1.00E-250 |  |
| CD | *SULT1A2* | 142 | 1.51E-05 | 1.11E-06 | 2.83E-06 | 7.62E-03 | 1.14E-02 | rs35590487 | 4.20E-03 | √ |
| CD | *SYNGR1* | 142 | 8.40E-01 | 7.76E-02 | 1.00E+00 | 4.27E-11 | 6.41E-11 | rs9296009 | 1.00E-250 |  |
| CD | *SYT11* | 95 | 7.06E-01 | 1.72E-04 | 1.05E-02 | 1.00E+00 | 1.00E+00 | rs9296009 | 1.00E-250 |  |
| CD | *TAB1* | 95 | 8.25E-01 | 1.00E+00 | 1.00E+00 | 4.33E-08 | 6.27E-08 | rs9296009 | 1.00E-250 |  |
| CD | *TAGAP* | 95 | 5.15E-01 | 1.86E-04 | 5.94E-03 | 6.50E-02 | 9.75E-02 | rs9296009 | 1.00E-250 |  |
| CD | *TCAP* | 95 | 2.17E-01 | 3.86E-03 | 1.00E+00 | 7.88E-07 | 1.20E-06 | rs9296009 | 1.00E-250 |  |
| CD | *TCF19* | 95 | 7.76E-02 | 1.00E+00 | 1.00E+00 | 1.73E-02 | 2.59E-02 | rs35502919 | 4.60E-186 |  |
| CD | *TCP10L2* | 95 | 8.02E-01 | 9.64E-16 | 1.81E-15 | 5.75E-11 | 8.63E-11 | rs11574914 | 1.50E-13 |  |
| CD | *TCTA* | 74 | 3.58E-18 | 5.34E-05 | 8.01E-02 | 2.03E-13 | 3.05E-13 | rs1571878 | 4.90E-15 |  |
| CD | *THADA* | 74 | 7.70E-01 | 2.38E-03 | 4.20E-02 | 1.04E-01 | 1.56E-01 | rs3087243 | 9.20E-20 |  |
| CD | *THBS3* | 74 | 6.11E-01 | 1.24E-04 | 3.89E-03 | 1.00E+00 | 1.00E+00 | rs9296009 | 1.00E-250 |  |
| CD | *TMEM191C* | 74 | 1.27E-03 | 5.45E-11 | 2.22E-11 | 3.12E-07 | 4.76E-07 | rs9296009 | 1.00E-250 |  |
| CD | *TMEM50B* | 74 | 9.08E-01 | 1.00E+00 | 1.00E+00 | 8.68E-04 | 1.30E-03 | rs55722612 | 1.00E-250 |  |
| CD | *TNF* | 74 | 1.34E-02 | 4.30E-11 | 9.05E-08 | 4.90E-06 | 6.97E-06 | rs35590487 | 4.20E-03 | √ |
| CD | *TNFSF15* | 71 | 7.99E-03 | 8.60E-18 | 2.56E-17 | 2.59E-13 | 3.89E-13 | rs35590487 | 4.20E-03 | √ |
| CD | *TNFSF8* | 71 | 3.28E-01 | 1.00E+00 | 1.00E+00 | 9.80E-08 | 1.50E-07 | rs9296009 | 1.00E-250 |  |
| CD | *TNIP1* | 71 | 7.42E-01 | 1.00E+00 | 1.00E+00 | 6.79E-06 | 1.02E-05 | rs35599935 | 2.40E-180 |  |
| CD | *TNXB* | 71 | 1.99E-02 | 1.80E-07 | 5.84E-07 | 2.73E-09 | 4.10E-09 | rs10790268 | 3.30E-15 |  |
| CD | *TRAIP* | 71 | 3.17E-01 | 3.58E-07 | 5.94E-07 | 2.55E-03 | 3.82E-03 | rs9296009 | 1.00E-250 |  |
| CD | *TRAPPC10* | 71 | 8.25E-01 | 5.97E-01 | 9.39E-01 | 3.55E-08 | 5.02E-08 | rs3819721 | 1.20E-242 |  |
| CD | *TRIB1* | 48 | 5.30E-01 | 1.00E+00 | 1.00E+00 | 1.08E-05 | 1.63E-05 | rs9296009 | 1.00E-250 |  |
| CD | *TRIM46* | 48 | 3.33E-01 | 1.11E-02 | 1.00E+00 | 1.00E+00 | 1.00E+00 | rs2844454 | 3.40E-205 |  |
| CD | *TRIM54* | 48 | 8.90E-01 | 8.15E-01 | 1.07E-02 | 1.00E+00 | 1.00E+00 | rs10790268 | 3.30E-15 |  |
| CD | *TTC33* | 48 | 1.72E-01 | 1.00E+00 | 1.00E+00 | 1.75E-17 | 2.62E-17 | rs73222264 | 1.80E-03 | √ |
| CD | *TUFM* | 48 | 2.04E-01 | 1.00E+00 | 1.00E+00 | 5.20E-03 | 7.80E-03 | rs9296009 | 1.00E-250 |  |
| CD | *TYK2* | 48 | 8.47E-01 | 1.00E+00 | 1.00E+00 | 2.68E-03 | 4.02E-03 | rs9296009 | 1.00E-250 |  |
| CD | *UBA7* | 48 | 4.41E-01 | 1.82E-06 | 3.15E-06 | 2.48E-03 | 3.72E-03 | rs9296009 | 1.00E-250 |  |
| CD | *UBE2L3* | 48 | 8.53E-01 | 7.56E-01 | 1.00E+00 | 2.69E-06 | 4.15E-06 | rs7488113 | 4.90E-04 | √ |
| CD | *UBLCP1* | 80 | 1.20E-01 | 1.00E+00 | 1.00E+00 | 6.45E-10 | 9.67E-10 | rs12715125 | 2.40E-08 |  |
| CD | *USP4* | 80 | 2.18E-01 | 7.20E-03 | 1.00E+00 | 3.89E-13 | 5.84E-13 | rs12232497 | 3.60E-09 |  |
| CD | *VARS* | 80 | 3.41E-01 | 7.44E-02 | 1.00E+00 | 1.12E-07 | 1.63E-07 | rs8032939 | 2.40E-12 |  |
| CD | *VWA7* | 80 | 1.30E-01 | 6.50E-01 | 1.00E+00 | 2.36E-07 | 3.51E-07 | rs56656810 | 2.80E-06 | √ |
| CD | *WDR78* | 80 | 1.90E-01 | 1.00E+00 | 1.00E+00 | 1.56E-12 | 2.34E-12 | rs10985070 | 1.70E-08 |  |
| CD | *WDR88* | 80 | 2.23E-01 | 8.37E-04 | 2.17E-03 | 2.63E-02 | 3.94E-02 | rs1571878 | 4.90E-15 |  |
| CD | *XXbac-BPG246D15.9* | 105 | 4.93E-01 | 1.46E-03 | 6.97E-01 | 1.21E-02 | 1.81E-02 | rs9296009 | 1.00E-250 |  |
| CD | *XXbac-BPG300A18.12* | 105 | 8.99E-02 | 4.78E-04 | 1.00E+00 | 3.56E-04 | 5.34E-04 | rs12539741 | 1.20E-11 |  |
| CD | *XXbac-BPG32J3.22* | 105 | 1.29E-02 | 2.08E-10 | 1.52E-03 | 5.81E-07 | 8.65E-07 | rs9261444 | 6.80E-52 |  |
| CD | *YDJC* | 105 | 8.08E-01 | 1.00E+00 | 1.00E+00 | 7.47E-06 | 1.14E-05 | rs11574914 | 1.50E-13 |  |
| CD | *YPEL1* | 105 | 8.77E-01 | 1.00E+00 | 1.00E+00 | 4.31E-05 | 6.49E-05 | rs9296009 | 1.00E-250 |  |
| CD | *ZBTB12* | 105 | 7.93E-02 | 5.54E-07 | 4.40E-01 | 1.45E-09 | 2.17E-09 | rs9296009 | 1.00E-250 |  |
| CD | *ZBTB46* | 115 | 4.63E-01 | 1.54E-02 | 1.00E+00 | 5.36E-03 | 8.04E-03 | rs12232497 | 3.60E-09 |  |
| CD | *ZCCHC24* | 115 | 5.77E-01 | 1.76E-08 | 7.57E-09 | 8.25E-05 | 1.24E-04 | rs2523668 | 1.20E-134 |  |
| CD | *ZFP36L2* | 115 | 7.65E-01 | 1.01E-02 | 1.00E+00 | 1.00E+00 | 1.00E+00 | rs35502919 | 4.60E-186 |  |
| CD | *ZGLP1* | 115 | 5.43E-01 | 1.00E+00 | 1.00E+00 | 2.27E-03 | 3.42E-03 | rs11089637 | 5.60E-07 | √ |
| CD | *ZGPAT* | 115 | 1.76E-01 | 1.00E+00 | 1.00E+00 | 6.76E-03 | 1.01E-02 | rs9357094 | 4.50E-60 |  |
| CD | *ZMIZ1* | 115 | 9.92E-01 | 4.23E-05 | 1.52E-03 | 2.96E-04 | 4.45E-04 | rs805284 | 2.80E-189 |  |
| CD | *ZNF300* | 115 | 5.60E-01 | 1.00E+00 | 1.00E+00 | 1.11E-09 | 1.67E-09 | rs805284 | 2.80E-189 |  |
| CD | *ZNF365* | 16 | 1.17E-02 | 1.00E+00 | 1.00E+00 | 3.70E-18 | 5.56E-18 | rs9268763 | 1.00E-250 |  |
| CD | *ZNF385C* | 16 | 8.09E-01 | 3.68E-03 | 4.70E-03 | 1.00E+00 | 1.00E+00 | rs7753264 | 1.00E-250 |  |
| CD | *ZNF513* | 16 | 8.69E-01 | 6.50E-02 | 2.38E-03 | 1.00E+00 | 1.00E+00 | rs34331363 | 1.00E-250 |  |
| CD | *ZPBP2* | 16 | 2.25E-01 | 2.30E-11 | 4.71E-06 | 8.61E-10 | 1.29E-09 | rs547261 | 1.00E-250 |  |
| CD | *ZRANB1* | 16 | 8.67E-01 | 3.87E-02 | 4.40E-02 | 1.00E+00 | 1.00E+00 | rs34331363 | 1.00E-250 |  |
| SLE | *ABHD16A* | 16 | 1.76E-29 | 8.25E-34 | 2.15E-04 | 1.11E-88 | 1.66E-88 | rs34331363 | 1.00E-250 |  |
| SLE | *ABI3BP* | 16 | 2.55E-06 | 1.00E+00 | 1.00E+00 | 1.00E+00 | 1.00E+00 | rs7653276 | 1.51E-05 | √ |
| SLE | *ABT1* | 16 | 2.03E-01 | 1.16E-16 | 1.00E+00 | 3.41E-13 | 5.12E-13 | rs45527431 | 5.68E-32 |  |
| SLE | *AC106782.18* | 109 | 7.04E-02 | 4.47E-11 | 1.24E-11 | 2.34E-37 | 3.50E-37 | rs1143679 | 5.03E-48 |  |
| SLE | *AF131215.3* | 109 | 2.89E-02 | 1.01E-04 | 4.61E-03 | 1.00E+00 | 1.00E+00 | rs2736332 | 4.83E-18 |  |
| SLE | *AGER* | 109 | 5.89E-02 | 2.82E-45 | 7.23E-18 | 1.06E-89 | 1.58E-89 | rs141910407 | 6.70E-103 |  |
| SLE | *AGPAT1* | 109 | 3.81E-01 | 7.30E-17 | 1.00E+00 | 6.77E-90 | 1.02E-89 | rs141910407 | 6.70E-103 |  |
| SLE | *AIF1* | 109 | 9.15E-14 | 8.39E-33 | 1.46E-27 | 1.33E-84 | 2.00E-84 | rs141910407 | 6.70E-103 |  |
| SLE | *ALPP* | 109 | 2.86E-01 | 1.00E+00 | 1.00E+00 | 4.20E-27 | 6.30E-27 | rs283478 | 4.12E-03 | √ |
| SLE | *ANO9* | 109 | 1.79E-03 | 4.03E-02 | 1.93E-02 | 1.00E+00 | 1.00E+00 | rs58688157 | 2.97E-11 |  |
| SLE | *ANXA6* | 59 | 5.69E-01 | 1.00E+00 | 1.00E+00 | 1.14E-04 | 1.97E-04 | rs8177429 | 1.32E-06 | √ |
| SLE | *APOM* | 59 | 7.42E-19 | 6.61E-22 | 1.64E-10 | 5.37E-88 | 8.05E-88 | rs141910407 | 6.70E-103 |  |
| SLE | *ARMC5* | 59 | 4.79E-02 | 1.00E+00 | 1.00E+00 | 2.60E-22 | 3.90E-22 | rs1143679 | 5.03E-48 |  |
| SLE | *ARPC5* | 59 | 6.60E-16 | 6.89E-07 | 3.09E-10 | 5.66E-01 | 8.49E-01 | rs17849501 | 1.81E-59 |  |
| SLE | *ATF6B* | 59 | 2.61E-01 | 2.65E-61 | 1.64E-31 | 1.46E-90 | 2.19E-90 | rs141910407 | 6.70E-103 |  |
| SLE | *ATP6V1F* | 59 | 1.55E-02 | 1.40E-18 | 7.24E-15 | 2.21E-34 | 3.32E-34 | rs35000415 | 1.86E-45 |  |
| SLE | *ATP6V1G2* | 59 | 2.33E-01 | 1.32E-62 | 7.77E-13 | 1.22E-83 | 1.83E-83 | rs115484360 | 2.93E-102 |  |
| SLE | *ATP6V1G2-DDX39B* | 59 | 6.38E-01 | 2.37E-61 | 2.89E-13 | 5.44E-84 | 8.15E-84 | rs115484360 | 2.93E-102 |  |
| SLE | *B3GALT4* | 35 | 7.14E-01 | 1.00E+00 | 1.00E+00 | 2.53E-08 | 3.62E-08 | rs114720630 | 6.72E-72 |  |
| SLE | *BAG6* | 35 | 1.49E-16 | 2.16E-24 | 5.15E-01 | 6.58E-88 | 9.88E-88 | rs141910407 | 6.70E-103 |  |
| SLE | *BLK* | 35 | 2.30E-03 | 1.00E+00 | 1.00E+00 | 5.18E-06 | 7.76E-06 | rs2736332 | 4.83E-18 |  |
| SLE | *BRD2* | 35 | 8.93E-02 | 1.00E+00 | 1.00E+00 | 6.95E-40 | 1.04E-39 | rs9273327 | 1.76E-100 |  |
| SLE | *BTN1A1* | 35 | 2.06E-02 | 1.00E+00 | 1.00E+00 | 3.56E-15 | 5.34E-15 | rs45527431 | 5.68E-32 |  |
| SLE | *BTN2A1* | 18 | 6.82E-01 | 1.00E+00 | 1.00E+00 | 6.89E-15 | 1.03E-14 | rs45527431 | 5.68E-32 |  |
| SLE | *BTN2A2* | 18 | 8.69E-01 | 1.00E+00 | 1.00E+00 | 2.35E-16 | 3.52E-16 | rs45527431 | 5.68E-32 |  |
| SLE | *BTN3A1* | 18 | 8.57E-01 | 1.00E+00 | 1.00E+00 | 1.42E-15 | 2.14E-15 | rs45527431 | 5.68E-32 |  |
| SLE | *BTN3A2* | 18 | 7.56E-01 | 1.00E+00 | 1.00E+00 | 2.04E-15 | 3.06E-15 | rs45527431 | 5.68E-32 |  |
| SLE | *BTN3A3* | 18 | 9.76E-01 | 1.00E+00 | 1.00E+00 | 4.90E-15 | 7.34E-15 | rs45527431 | 5.68E-32 |  |
| SLE | *BTNL2* | 18 | 3.82E-01 | 4.88E-04 | 1.65E-02 | 1.22E-84 | 1.83E-84 | rs141910407 | 6.70E-103 |  |
| SLE | *C11orf35* | 18 | 7.39E-06 | 9.37E-03 | 9.04E-03 | 1.00E+00 | 1.00E+00 | rs58688157 | 2.97E-11 |  |
| SLE | *C2* | 18 | 2.30E-04 | 1.99E-33 | 4.12E-12 | 4.74E-90 | 7.11E-90 | rs141910407 | 6.70E-103 |  |
| SLE | *C3orf25* | 18 | 1.81E-01 | 1.00E+00 | 1.00E+00 | 6.18E-20 | 9.26E-20 | rs9852014 | 2.26E-36 |  |
| SLE | *C3orf37* | 18 | 6.62E-02 | 1.00E+00 | 1.00E+00 | 6.39E-26 | 9.58E-26 | rs9852014 | 2.26E-36 |  |
| SLE | *C4A* | 18 | 3.86E-01 | 4.54E-20 | 9.24E-03 | 4.98E-91 | 7.46E-91 | rs141910407 | 6.70E-103 |  |
| SLE | *C4B* | 18 | 6.49E-02 | 3.24E-56 | 1.57E-14 | 2.57E-91 | 3.86E-91 | rs141910407 | 6.70E-103 |  |
| SLE | *C5orf54* | 18 | 5.76E-01 | 9.92E-01 | 1.00E+00 | 2.07E-04 | 3.10E-04 | rs76623030 | 7.87E-05 | √ |
| SLE | *C6orf47* | 18 | 8.03E-21 | 4.36E-20 | 7.05E-08 | 1.33E-88 | 1.99E-88 | rs141910407 | 6.70E-103 |  |
| SLE | *C6orf48* | 29 | 7.71E-14 | 3.25E-20 | 4.96E-04 | 1.92E-89 | 2.88E-89 | rs141910407 | 6.70E-103 |  |
| SLE | *CCDC116* | 206 | 7.28E-01 | 1.00E+00 | 1.00E+00 | 4.20E-03 | 6.29E-03 | rs3747093 | 2.88E-14 |  |
| SLE | *CCDC69* | 206 | 9.95E-01 | 1.00E+00 | 1.00E+00 | 1.51E-03 | 2.26E-03 | rs6889239 | 2.19E-18 |  |
| SLE | *CCHCR1* | 206 | 3.38E-12 | 9.61E-16 | 4.87E-05 | 2.69E-77 | 4.03E-77 | rs147027375 | 1.55E-96 |  |
| SLE | *CD44* | 206 | 8.41E-05 | 2.87E-06 | 3.07E-04 | 6.26E-02 | 9.39E-02 | rs353608 | 2.93E-11 |  |
| SLE | *CDC37* | 206 | 2.50E-09 | 2.71E-03 | 1.00E+00 | 1.83E-03 | 2.75E-03 | rs35251378 | 3.61E-13 |  |
| SLE | *CDHR5* | 117 | 2.28E-09 | 1.17E-03 | 1.00E+00 | 1.00E+00 | 1.00E+00 | rs58688157 | 2.97E-11 |  |
| SLE | *CDIPT* | 117 | 4.93E-01 | 3.77E-02 | 1.00E+00 | 1.00E+00 | 1.00E+00 | rs4787482 | 1.22E-03 | √ |
| SLE | *CFB* | 117 | 5.33E-05 | 8.03E-29 | 6.47E-08 | 6.29E-90 | 9.44E-90 | rs141910407 | 6.70E-103 |  |
| SLE | *CLDN7* | 117 | 8.86E-01 | 9.70E-03 | 1.00E+00 | 1.00E+00 | 1.00E+00 | rs3809822 | 1.29E-07 | √ |
| SLE | *CLIC1* | 144 | 5.68E-19 | 4.57E-07 | 1.00E+00 | 1.88E-89 | 2.82E-89 | rs141910407 | 6.70E-103 |  |
| SLE | *COL11A2* | 9 | 9.92E-01 | 1.00E+00 | 1.00E+00 | 4.35E-13 | 6.52E-13 | rs9273327 | 1.76E-100 |  |
| SLE | *COPG1* | 22 | 1.41E-01 | 1.00E+00 | 1.00E+00 | 1.57E-23 | 2.35E-23 | rs9852014 | 2.26E-36 |  |
| SLE | *CSNK2B* | 22 | 4.30E-23 | 3.05E-23 | 4.63E-15 | 3.03E-88 | 4.55E-88 | rs141910407 | 6.70E-103 |  |
| SLE | *CTA-14H9.5* | 119 | 4.20E-02 | 1.00E+00 | 1.00E+00 | 6.35E-14 | 9.52E-14 | rs45527431 | 5.68E-32 |  |
| SLE | *CTB-17P3.4* | 35 | 8.45E-01 | 1.00E+00 | 1.00E+00 | 4.23E-05 | 7.09E-05 | rs8177429 | 1.32E-06 | √ |
| SLE | *CTC-231O11.1* | 35 | 1.90E-02 | 3.28E-05 | 1.00E+00 | 1.53E-04 | 2.30E-04 | rs71603662 | 3.64E-07 | √ |
| SLE | *CUTA* | 32 | 2.57E-01 | 1.00E+00 | 1.00E+00 | 8.15E-03 | 1.23E-02 | rs116298963 | 5.55E-54 |  |
| SLE | *CYP21A2* | 32 | 7.43E-01 | 4.26E-69 | 1.17E-17 | 1.11E-90 | 1.67E-90 | rs141910407 | 6.70E-103 |  |
| SLE | *DAXX* | 32 | 5.89E-01 | 1.00E+00 | 1.00E+00 | 1.15E-02 | 1.73E-02 | rs114828403 | 6.82E-72 |  |
| SLE | *DDAH2* | 32 | 5.14E-19 | 1.73E-14 | 1.26E-01 | 9.68E-90 | 1.45E-89 | rs141910407 | 6.70E-103 |  |
| SLE | *DDX39B* | 32 | 4.20E-01 | 1.12E-53 | 7.31E-03 | 2.91E-84 | 4.37E-84 | rs115484360 | 2.93E-102 |  |
| SLE | *DIS3L2* | 48 | 8.47E-01 | 1.00E+00 | 1.00E+00 | 2.33E-27 | 3.49E-27 | rs2573219 | 1.13E-42 |  |
| SLE | *DOM3Z* | 48 | 5.17E-01 | 2.00E-72 | 2.04E-40 | 3.79E-92 | 5.69E-92 | rs141910407 | 6.70E-103 |  |
| SLE | *DPCR1* | 48 | 1.93E-06 | 1.00E+00 | 1.00E+00 | 1.00E+00 | 1.00E+00 | rs147027375 | 1.55E-96 |  |
| SLE | *EGFL8* | 48 | 9.24E-02 | 5.02E-45 | 1.87E-29 | 1.03E-89 | 1.54E-89 | rs141910407 | 6.70E-103 |  |
| SLE | *EHMT2* | 48 | 6.22E-04 | 1.16E-25 | 1.00E+00 | 2.62E-90 | 3.93E-90 | rs141910407 | 6.70E-103 |  |
| SLE | *FAM106A* | 48 | 4.96E-13 | 1.00E+00 | 1.00E+00 | 1.00E+00 | 1.00E+00 | rs62074179 | 1.46E-04 | √ |
| SLE | *FAM167A* | 48 | 9.44E-07 | 3.19E-05 | 3.83E-04 | 1.32E-05 | 1.99E-05 | rs2736332 | 4.83E-18 |  |
| SLE | *FCGR2A* | 44 | 6.46E-01 | 1.76E-10 | 1.43E-03 | 1.00E+00 | 1.00E+00 | rs6671847 | 6.64E-12 |  |
| SLE | *FDX1L* | 44 | 1.29E-11 | 7.15E-07 | 1.18E-04 | 1.73E-03 | 2.59E-03 | rs35251378 | 3.61E-13 |  |
| SLE | *FKBPL* | 44 | 4.65E-01 | 1.21E-44 | 7.71E-11 | 4.27E-90 | 6.41E-90 | rs141910407 | 6.70E-103 |  |
| SLE | *FLNC* | 44 | 6.93E-03 | 6.05E-16 | 3.90E-15 | 4.14E-34 | 6.22E-34 | rs35000415 | 1.86E-45 |  |
| SLE | *FUS* | 44 | 5.33E-01 | 2.91E-14 | 1.41E-17 | 3.20E-37 | 4.80E-37 | rs1143679 | 5.03E-48 |  |
| SLE | *FUT1* | 44 | 6.55E-01 | 1.81E-02 | 1.00E+00 | 1.00E+00 | 1.00E+00 | rs73934025 | 5.39E-06 | √ |
| SLE | *GABBR1* | 44 | 1.70E-01 | 9.61E-06 | 5.21E-01 | 5.52E-05 | 8.26E-05 | rs115236446 | 1.15E-47 |  |
| SLE | *GLS* | 44 | 4.23E-01 | 1.64E-01 | 1.64E-01 | 1.50E-22 | 2.25E-22 | rs36010116 | 9.48E-05 | √ |
| SLE | *GPANK1* | 37 | 1.71E-23 | 8.34E-23 | 9.15E-16 | 6.91E-88 | 1.04E-87 | rs141910407 | 6.70E-103 |  |
| SLE | *GPSM3* | 37 | 6.24E-03 | 2.39E-45 | 1.97E-33 | 6.20E-90 | 9.30E-90 | rs141910407 | 6.70E-103 |  |
| SLE | *GPX3* | 37 | 9.65E-01 | 1.00E+00 | 1.00E+00 | 5.87E-04 | 9.10E-04 | rs6869426 | 1.17E-03 | √ |
| SLE | *GSDMB* | 37 | 2.55E-07 | 1.63E-01 | 1.00E+00 | 1.00E+00 | 1.00E+00 | rs112679482 | 8.29E-06 | √ |
| SLE | *H1FX* | 37 | 1.57E-01 | 1.00E+00 | 1.00E+00 | 9.24E-22 | 1.39E-21 | rs9852014 | 2.26E-36 |  |
| SLE | *HCG11* | 37 | 2.56E-02 | 1.00E+00 | 1.00E+00 | 1.16E-14 | 1.74E-14 | rs45527431 | 5.68E-32 |  |
| SLE | *HCG22* | 37 | 3.59E-08 | 1.48E-18 | 3.18E-11 | 1.19E-77 | 1.78E-77 | rs147027375 | 1.55E-96 |  |
| SLE | *HCG27* | 17 | 1.85E-02 | 1.62E-22 | 4.29E-18 | 5.73E-76 | 8.59E-76 | rs115619714 | 1.26E-98 |  |
| SLE | *HFE* | 17 | 7.32E-02 | 1.00E+00 | 1.00E+00 | 1.07E-10 | 1.60E-10 | rs35400317 | 5.89E-32 |  |
| SLE | *HIST1H1B* | 17 | 3.64E-10 | 3.53E-07 | 1.00E+00 | 2.51E-22 | 3.76E-22 | rs13197176 | 1.05E-34 |  |
| SLE | *HIST1H1C* | 8 | 2.70E-03 | 1.00E+00 | 1.00E+00 | 2.04E-11 | 3.05E-11 | rs13195401 | 3.45E-31 |  |
| SLE | *HIST1H1D* | 8 | 4.17E-01 | 1.00E+00 | 1.00E+00 | 1.08E-13 | 1.63E-13 | rs45527431 | 5.68E-32 |  |
| SLE | *HIST1H1E* | 35 | 7.90E-01 | 1.00E+00 | 1.00E+00 | 1.13E-12 | 1.70E-12 | rs45527431 | 5.68E-32 |  |
| SLE | *HIST1H2AB* | 35 | 1.54E-03 | 1.00E+00 | 1.00E+00 | 1.90E-11 | 2.85E-11 | rs13195401 | 3.45E-31 |  |
| SLE | *HIST1H2AC* | 35 | 5.45E-01 | 1.00E+00 | 1.00E+00 | 1.50E-12 | 2.26E-12 | rs45527431 | 5.68E-32 |  |
| SLE | *HIST1H2AE* | 20 | 6.56E-01 | 1.00E+00 | 1.00E+00 | 2.60E-12 | 3.91E-12 | rs45527431 | 5.68E-32 |  |
| SLE | *HIST1H2AG* | 20 | 1.46E-01 | 1.00E+00 | 1.00E+00 | 2.56E-19 | 3.85E-19 | rs140365013 | 8.94E-33 |  |
| SLE | *HIST1H2AH* | 20 | 2.00E-01 | 1.00E+00 | 1.00E+00 | 6.46E-19 | 9.69E-19 | rs140365013 | 8.94E-33 |  |
| SLE | *HIST1H2AI* | 32 | 5.41E-11 | 9.22E-08 | 4.04E-06 | 6.14E-23 | 9.21E-23 | rs13197176 | 1.05E-34 |  |
| SLE | *HIST1H2AJ* | 32 | 8.08E-11 | 3.82E-08 | 2.06E-06 | 1.34E-22 | 2.01E-22 | rs13197176 | 1.05E-34 |  |
| SLE | *HIST1H2AK* | 32 | 8.35E-11 | 2.36E-03 | 1.00E+00 | 2.76E-21 | 4.14E-21 | rs13197176 | 1.05E-34 |  |
| SLE | *HIST1H2AL* | 28 | 1.10E-10 | 1.54E-04 | 1.00E+00 | 4.49E-22 | 6.73E-22 | rs13197176 | 1.05E-34 |  |
| SLE | *HIST1H2AM* | 28 | 9.76E-11 | 8.29E-04 | 1.00E+00 | 1.16E-21 | 1.75E-21 | rs13197176 | 1.05E-34 |  |
| SLE | *HIST1H2BB* | 28 | 2.53E-03 | 5.51E-03 | 3.70E-05 | 9.98E-12 | 1.50E-11 | rs13195401 | 3.45E-31 |  |
| SLE | *HIST1H2BC* | 29 | 2.67E-01 | 1.00E+00 | 1.00E+00 | 3.07E-13 | 4.60E-13 | rs45527431 | 5.68E-32 |  |
| SLE | *HIST1H2BD* | 73 | 7.99E-01 | 1.00E+00 | 1.00E+00 | 3.01E-13 | 4.52E-13 | rs45527431 | 5.68E-32 |  |
| SLE | *HIST1H2BE* | 73 | 6.92E-01 | 1.00E+00 | 1.00E+00 | 1.69E-13 | 2.53E-13 | rs45527431 | 5.68E-32 |  |
| SLE | *HIST1H2BF* | 73 | 7.33E-01 | 1.00E+00 | 1.00E+00 | 4.36E-13 | 6.54E-13 | rs45527431 | 5.68E-32 |  |
| SLE | *HIST1H2BG* | 12 | 5.35E-01 | 1.00E+00 | 1.00E+00 | 2.96E-13 | 4.45E-13 | rs45527431 | 5.68E-32 |  |
| SLE | *HIST1H2BH* | 96 | 4.44E-01 | 1.00E+00 | 1.00E+00 | 2.83E-14 | 4.25E-14 | rs45527431 | 5.68E-32 |  |
| SLE | *HIST1H2BI* | 14 | 3.45E-01 | 1.00E+00 | 1.00E+00 | 2.08E-13 | 3.12E-13 | rs45527431 | 5.68E-32 |  |
| SLE | *HIST1H2BJ* | 14 | 1.13E-01 | 1.00E+00 | 1.00E+00 | 5.26E-19 | 7.89E-19 | rs140365013 | 8.94E-33 |  |
| SLE | *HIST1H2BK* | 111 | 1.76E-01 | 1.00E+00 | 1.00E+00 | 2.25E-19 | 3.38E-19 | rs140365013 | 8.94E-33 |  |
| SLE | *HIST1H2BL* | 928 | 3.01E-10 | 8.21E-07 | 5.04E-06 | 4.35E-23 | 6.52E-23 | rs13197176 | 1.05E-34 |  |
| SLE | *HIST1H2BM* | 389 | 6.32E-11 | 7.07E-09 | 3.87E-07 | 1.92E-23 | 2.88E-23 | rs13197176 | 1.05E-34 |  |
| SLE | *HIST1H2BN* | 87 | 1.30E-10 | 6.03E-01 | 1.00E+00 | 4.60E-20 | 6.90E-20 | rs13197176 | 1.05E-34 |  |
| SLE | *HIST1H2BO* | 254 | 8.89E-11 | 1.46E-05 | 1.00E+00 | 1.39E-21 | 2.08E-21 | rs13197176 | 1.05E-34 |  |
| SLE | *HIST1H3A* | 254 | 4.61E-04 | 1.00E+00 | 1.00E+00 | 7.78E-12 | 1.17E-11 | rs13195401 | 3.45E-31 |  |
| SLE | *HIST1H3B* | 254 | 1.02E-03 | 1.00E+00 | 1.00E+00 | 2.18E-11 | 3.28E-11 | rs13195401 | 3.45E-31 |  |
| SLE | *HIST1H3C* | 254 | 4.76E-03 | 1.00E+00 | 1.00E+00 | 1.25E-10 | 1.87E-10 | rs13195401 | 3.45E-31 |  |
| SLE | *HIST1H3D* | 254 | 9.64E-01 | 1.00E+00 | 1.00E+00 | 3.95E-12 | 5.92E-12 | rs45527431 | 5.68E-32 |  |
| SLE | *HIST1H3E* | 254 | 3.78E-01 | 1.00E+00 | 1.00E+00 | 1.21E-13 | 1.82E-13 | rs45527431 | 5.68E-32 |  |
| SLE | *HIST1H3F* | 23 | 5.77E-01 | 1.00E+00 | 1.00E+00 | 4.90E-14 | 7.36E-14 | rs45527431 | 5.68E-32 |  |
| SLE | *HIST1H3G* | 23 | 4.10E-01 | 1.00E+00 | 1.00E+00 | 3.02E-14 | 4.54E-14 | rs45527431 | 5.68E-32 |  |
| SLE | *HIST1H3H* | 23 | 8.08E-11 | 6.12E-09 | 1.44E-08 | 4.36E-23 | 6.53E-23 | rs13197176 | 1.05E-34 |  |
| SLE | *HIST1H3I* | 85 | 4.98E-11 | 3.06E-04 | 1.00E+00 | 1.14E-21 | 1.71E-21 | rs13197176 | 1.05E-34 |  |
| SLE | *HIST1H3J* | 263 | 2.13E-10 | 1.05E-01 | 1.00E+00 | 5.02E-21 | 7.53E-21 | rs13197176 | 1.05E-34 |  |
| SLE | *HIST1H4A* | 69 | 1.68E-03 | 1.00E+00 | 1.00E+00 | 1.55E-10 | 2.33E-10 | rs13195401 | 3.45E-31 |  |
| SLE | *HIST1H4B* | 21 | 9.10E-04 | 1.00E+00 | 1.00E+00 | 7.14E-11 | 1.07E-10 | rs13195401 | 3.45E-31 |  |
| SLE | *HIST1H4C* | 21 | 2.10E-01 | 1.00E+00 | 1.00E+00 | 5.92E-13 | 8.88E-13 | rs45527431 | 5.68E-32 |  |
| SLE | *HIST1H4D* | 21 | 6.94E-01 | 1.00E+00 | 1.00E+00 | 2.33E-13 | 3.49E-13 | rs45527431 | 5.68E-32 |  |
| SLE | *HIST1H4E* | 159 | 6.02E-01 | 1.00E+00 | 1.00E+00 | 4.38E-12 | 6.57E-12 | rs45527431 | 5.68E-32 |  |
| SLE | *HIST1H4H* | 159 | 1.85E-01 | 1.00E+00 | 1.00E+00 | 4.67E-14 | 7.00E-14 | rs45527431 | 5.68E-32 |  |
| SLE | *HIST1H4I* | 159 | 2.14E-01 | 1.00E+00 | 1.00E+00 | 3.83E-19 | 5.74E-19 | rs140365013 | 8.94E-33 |  |
| SLE | *HIST1H4J* | 159 | 9.82E-11 | 2.72E-04 | 1.00E+00 | 4.20E-22 | 6.30E-22 | rs13197176 | 1.05E-34 |  |
| SLE | *HIST1H4K* | 262 | 5.74E-11 | 1.47E-03 | 1.00E+00 | 7.07E-21 | 1.06E-20 | rs13197176 | 1.05E-34 |  |
| SLE | *HLA-A* | 262 | 6.97E-01 | 1.00E+00 | 1.00E+00 | 2.29E-12 | 3.43E-12 | rs116186974 | 1.59E-51 |  |
| SLE | *HLA-B* | 97 | 3.64E-01 | 3.64E-17 | 1.74E-11 | 9.25E-86 | 1.39E-85 | rs116099232 | 2.94E-100 |  |
| SLE | *HLA-C* | 67 | 2.53E-01 | 7.57E-18 | 1.08E-14 | 3.10E-86 | 4.65E-86 | rs3131383 | 6.60E-99 |  |
| SLE | *HLA-DMA* | 41 | 5.75E-02 | 1.00E+00 | 1.00E+00 | 2.85E-38 | 4.28E-38 | rs9273327 | 1.76E-100 |  |
| SLE | *HLA-DMB* | 41 | 9.52E-04 | 1.00E+00 | 1.00E+00 | 3.49E-45 | 5.24E-45 | rs9273327 | 1.76E-100 |  |
| SLE | *HLA-DOA* | 41 | 7.76E-03 | 1.00E+00 | 1.00E+00 | 1.55E-30 | 2.33E-30 | rs9273327 | 1.76E-100 |  |
| SLE | *HLA-DOB* | 102 | 1.40E-01 | 1.94E-08 | 1.00E+00 | 5.29E-46 | 7.93E-46 | rs9273327 | 1.76E-100 |  |
| SLE | *HLA-DPA1* | 52 | 8.27E-01 | 1.00E+00 | 1.00E+00 | 6.82E-11 | 1.02E-10 | rs9273327 | 1.76E-100 |  |
| SLE | *HLA-DPB1* | 223 | 8.77E-01 | 1.00E+00 | 1.00E+00 | 2.34E-13 | 3.51E-13 | rs9273327 | 1.76E-100 |  |
| SLE | *HLA-DQA1* | 223 | 1.68E-12 | 9.92E-20 | 1.98E-15 | 1.07E-27 | 1.61E-27 | rs1269852 | 6.99E-101 |  |
| SLE | *HLA-DQA2* | 223 | 1.04E-01 | 3.79E-12 | 1.00E+00 | 5.22E-46 | 7.83E-46 | rs9273327 | 1.76E-100 |  |
| SLE | *HLA-DQB1* | 223 | 4.51E-13 | 2.91E-21 | 3.98E-26 | 1.00E-27 | 1.50E-27 | rs116300399 | 7.35E-101 |  |
| SLE | *HLA-DQB2* | 87 | 2.28E-01 | 3.29E-11 | 1.37E-01 | 9.62E-48 | 1.44E-47 | rs9273327 | 1.76E-100 |  |
| SLE | *HLA-DRA* | 87 | 7.77E-03 | 1.25E-04 | 1.80E-02 | 1.00E+00 | 1.00E+00 | rs141910407 | 6.70E-103 |  |
| SLE | *HLA-DRB1* | 87 | 1.49E-12 | 6.95E-14 | 3.28E-08 | 3.17E-27 | 4.75E-27 | rs1150757 | 2.08E-101 |  |
| SLE | *HLA-DRB5* | 16 | 7.14E-09 | 3.01E-14 | 3.27E-10 | 4.32E-29 | 6.48E-29 | rs141910407 | 6.70E-103 |  |
| SLE | *HLA-F* | 16 | 1.16E-01 | 1.10E-06 | 1.00E+00 | 1.39E-05 | 2.17E-05 | rs115387234 | 3.50E-49 |  |
| SLE | *HLA-G* | 27 | 2.17E-01 | 4.48E-01 | 1.00E+00 | 6.95E-07 | 1.11E-06 | rs114416651 | 1.34E-49 |  |
| SLE | *HMGN4* | 27 | 3.07E-02 | 2.47E-01 | 1.00E+00 | 2.47E-14 | 3.70E-14 | rs45527431 | 5.68E-32 |  |
| SLE | *HRAS* | 89 | 1.27E-03 | 4.14E-03 | 4.17E-03 | 1.00E+00 | 1.00E+00 | rs58688157 | 2.97E-11 |  |
| SLE | *HSD17B8* | 89 | 8.50E-01 | 1.00E+00 | 1.00E+00 | 6.44E-12 | 9.66E-12 | rs115575857 | 2.42E-98 |  |
| SLE | *HSPA1A* | 27 | 3.21E-21 | 3.90E-07 | 1.00E+00 | 2.51E-89 | 3.76E-89 | rs141910407 | 6.70E-103 |  |
| SLE | *HSPA1B* | 131 | 2.58E-14 | 5.97E-17 | 5.93E-01 | 1.94E-89 | 2.91E-89 | rs141910407 | 6.70E-103 |  |
| SLE | *HSPA1L* | 130 | 6.60E-26 | 1.80E-14 | 1.00E+00 | 1.83E-89 | 2.75E-89 | rs141910407 | 6.70E-103 |  |
| SLE | *HSPA6* | 144 | 2.69E-01 | 7.67E-11 | 2.87E-01 | 1.00E+00 | 1.00E+00 | rs6671847 | 6.64E-12 |  |
| SLE | *ICAM1* | 144 | 5.70E-08 | 2.90E-03 | 1.00E+00 | 1.31E-03 | 1.97E-03 | rs35251378 | 3.61E-13 |  |
| SLE | *ICAM3* | 178 | 5.91E-11 | 3.15E-02 | 1.00E+00 | 1.96E-03 | 2.94E-03 | rs35251378 | 3.61E-13 |  |
| SLE | *ICAM4* | 384 | 3.39E-09 | 3.45E-01 | 1.00E+00 | 2.75E-03 | 4.13E-03 | rs35251378 | 3.61E-13 |  |
| SLE | *ICAM5* | 384 | 2.09E-08 | 1.00E+00 | 1.00E+00 | 5.86E-03 | 8.78E-03 | rs35251378 | 3.61E-13 |  |
| SLE | *IFT122* | 78 | 9.76E-01 | 1.00E+00 | 1.00E+00 | 7.17E-23 | 1.08E-22 | rs9852014 | 2.26E-36 |  |
| SLE | *IKZF3* | 78 | 1.87E-06 | 4.96E-01 | 1.00E+00 | 1.00E+00 | 1.00E+00 | rs112679482 | 8.29E-06 | √ |
| SLE | *IL1RN* | 78 | 6.92E-01 | 1.00E+00 | 1.00E+00 | 1.04E-69 | 1.56E-69 | rs276819 | 5.81E-05 | √ |
| SLE | *IRF5* | 78 | 1.40E-06 | 1.91E-13 | 1.52E-13 | 1.08E-33 | 1.62E-33 | rs35000415 | 1.86E-45 |  |
| SLE | *IRF7* | 302 | 1.26E-08 | 1.72E-04 | 2.62E-01 | 1.00E+00 | 1.00E+00 | rs58688157 | 2.97E-11 |  |
| SLE | *IRF8* | 302 | 1.98E-07 | 1.94E-09 | 1.16E-03 | 1.47E-06 | 2.27E-06 | rs13332649 | 5.43E-17 |  |
| SLE | *ITGAD* | 302 | 5.59E-08 | 1.88E-03 | 1.00E+00 | 3.58E-36 | 5.37E-36 | rs1143679 | 5.03E-48 |  |
| SLE | *ITGAM* | 301 | 5.72E-06 | 5.00E-09 | 1.96E-09 | 1.35E-37 | 2.03E-37 | rs1143679 | 5.03E-48 |  |
| SLE | *ITGAX* | 301 | 4.53E-07 | 3.07E-04 | 1.00E+00 | 4.50E-37 | 6.74E-37 | rs1143679 | 5.03E-48 |  |
| SLE | *KCNN3* | 215 | 4.83E-01 | 1.14E-02 | 1.00E+00 | 1.00E+00 | 1.00E+00 | rs11264305 | 1.96E-04 | √ |
| SLE | *KCTD13* | 120 | 6.69E-01 | 4.66E-02 | 1.00E+00 | 1.00E+00 | 1.00E+00 | rs4787482 | 1.22E-03 | √ |
| SLE | *KIFC1* | 120 | 3.03E-01 | 1.00E+00 | 1.00E+00 | 4.70E-02 | 7.06E-02 | rs115591082 | 7.33E-55 |  |
| SLE | *LAMC1* | 269 | 3.33E-05 | 2.44E-02 | 1.00E+00 | 1.00E+00 | 1.00E+00 | rs17849501 | 1.81E-59 |  |
| SLE | *LAMC2* | 269 | 2.17E-08 | 6.10E-04 | 1.00E+00 | 7.72E-14 | 1.16E-13 | rs17849501 | 1.81E-59 |  |
| SLE | *LINC00240* | 269 | 5.50E-02 | 1.00E+00 | 1.00E+00 | 1.12E-19 | 1.68E-19 | rs13191227 | 1.41E-32 |  |
| SLE | *LINC00481* | 269 | 3.95E-02 | 1.49E-05 | 2.67E-06 | 4.39E-77 | 6.59E-77 | rs115619714 | 1.26E-98 |  |
| SLE | *LRRC16A* | 294 | 3.76E-02 | 1.00E+00 | 1.00E+00 | 2.12E-07 | 3.14E-07 | rs41266779 | 1.54E-21 |  |
| SLE | *LRRC56* | 126 | 7.13E-06 | 1.39E-03 | 6.85E-04 | 1.00E+00 | 1.00E+00 | rs58688157 | 2.97E-11 |  |
| SLE | *LSM2* | 21 | 8.90E-21 | 8.92E-17 | 1.00E+00 | 2.06E-89 | 3.09E-89 | rs141910407 | 6.70E-103 |  |
| SLE | *LST1* | 21 | 1.66E-01 | 7.16E-58 | 4.74E-18 | 7.76E-85 | 1.16E-84 | rs141910407 | 6.70E-103 |  |
| SLE | *LTA* | 100 | 1.02E-01 | 9.83E-67 | 8.12E-16 | 2.00E-84 | 3.01E-84 | rs141910407 | 6.70E-103 |  |
| SLE | *LTB* | 167 | 1.54E-01 | 2.73E-43 | 6.26E-11 | 2.00E-84 | 3.00E-84 | rs141910407 | 6.70E-103 |  |
| SLE | *LY6G5B* | 174 | 3.43E-24 | 2.33E-22 | 5.34E-23 | 6.69E-88 | 1.00E-87 | rs141910407 | 6.70E-103 |  |
| SLE | *LY6G5C* | 122 | 4.26E-27 | 1.96E-32 | 5.99E-17 | 4.61E-88 | 6.91E-88 | rs141910407 | 6.70E-103 |  |
| SLE | *MBD4* | 122 | 9.87E-02 | 1.00E+00 | 1.00E+00 | 1.69E-23 | 2.53E-23 | rs9852014 | 2.26E-36 |  |
| SLE | *MICA* | 170 | 6.79E-04 | 1.01E-35 | 1.91E-08 | 1.45E-87 | 2.18E-87 | rs141597299 | 1.44E-100 |  |
| SLE | *MICB* | 170 | 3.64E-01 | 3.19E-55 | 8.50E-16 | 3.04E-84 | 4.56E-84 | rs115484360 | 2.93E-102 |  |
| SLE | *MIR210HG* | 170 | 1.97E-05 | 1.36E-02 | 1.26E-02 | 1.00E+00 | 1.00E+00 | rs58688157 | 2.97E-11 |  |
| SLE | *MRPL4* | 30 | 1.77E-06 | 1.00E+00 | 1.00E+00 | 8.45E-03 | 1.27E-02 | rs35251378 | 3.61E-13 |  |
| SLE | *MSH5* | 61 | 2.59E-19 | 4.72E-10 | 1.00E+00 | 1.16E-89 | 1.74E-89 | rs141910407 | 6.70E-103 |  |
| SLE | *MSH5-SAPCD1* | 61 | 1.35E-18 | 9.57E-09 | 1.00E+00 | 2.51E-89 | 3.76E-89 | rs141910407 | 6.70E-103 |  |
| SLE | *MUC22* | 106 | 3.61E-09 | 9.27E-25 | 2.97E-19 | 3.97E-77 | 5.95E-77 | rs147027375 | 1.55E-96 |  |
| SLE | *NAB1* | 54 | 7.27E-02 | 1.00E+00 | 1.00E+00 | 6.48E-09 | 9.71E-09 | rs4274624 | 9.73E-66 |  |
| SLE | *NCF2* | 70 | 1.17E-11 | 4.35E-12 | 1.71E-14 | 1.15E-01 | 1.72E-01 | rs17849501 | 1.81E-59 |  |
| SLE | *NCR3* | 40 | 2.19E-01 | 1.28E-42 | 1.43E-12 | 2.47E-84 | 3.70E-84 | rs141910407 | 6.70E-103 |  |
| SLE | *NEU1* | 40 | 1.13E-12 | 7.16E-19 | 1.00E+00 | 5.88E-90 | 8.82E-90 | rs141910407 | 6.70E-103 |  |
| SLE | *NFKBIL1* | 71 | 1.56E-01 | 3.17E-41 | 3.88E-09 | 2.63E-84 | 3.95E-84 | rs115484360 | 2.93E-102 |  |
| SLE | *NKAPL* | 95 | 5.52E-10 | 3.63E-01 | 1.00E+00 | 8.08E-25 | 1.21E-24 | rs116725083 | 1.78E-36 |  |
| SLE | *NMNAT2* | 288 | 9.97E-15 | 7.91E-03 | 5.91E-02 | 1.21E-10 | 1.82E-10 | rs17849501 | 1.81E-59 |  |
| SLE | *NOTCH4* | 75 | 1.68E-01 | 5.14E-37 | 4.03E-33 | 3.51E-90 | 5.27E-90 | rs141910407 | 6.70E-103 |  |
| SLE | *OR2B6* | 35 | 5.98E-11 | 2.13E-07 | 1.00E+00 | 1.98E-22 | 2.97E-22 | rs67981811 | 9.96E-35 |  |
| SLE | *OR2T3* | 860 | 5.42E-01 | 4.02E-08 | 1.00E+00 | 1.00E+00 | 1.00E+00 | rs115263146 | 1.49E-02 | √ |
| SLE | *ORMDL3* | 186 | 1.43E-08 | 9.19E-04 | 1.00E+00 | 1.00E+00 | 1.00E+00 | rs112679482 | 8.29E-06 | √ |
| SLE | *PBX2* | 73 | 2.38E-01 | 1.12E-36 | 5.99E-21 | 1.08E-89 | 1.62E-89 | rs141910407 | 6.70E-103 |  |
| SLE | *PDCD2* | 307 | 2.09E-01 | 6.44E-13 | 1.00E+00 | 1.00E+00 | 1.00E+00 | rs6927768 | 1.09E-04 | √ |
| SLE | *PDE4A* | 307 | 1.08E-07 | 1.00E+00 | 1.00E+00 | 1.16E-03 | 1.73E-03 | rs35251378 | 3.61E-13 |  |
| SLE | *PDE6A* | 307 | 1.10E-01 | 1.00E+00 | 1.00E+00 | 2.44E-08 | 3.62E-08 | rs1078324 | 7.11E-20 |  |
| SLE | *PFDN6* | 307 | 6.68E-01 | 1.00E+00 | 1.00E+00 | 1.73E-10 | 2.60E-10 | rs114720630 | 6.72E-72 |  |
| SLE | *PGBD1* | 307 | 7.01E-12 | 1.68E-04 | 2.11E-03 | 6.13E-25 | 9.19E-25 | rs115387042 | 1.72E-37 |  |
| SLE | *PHF1* | 50 | 4.49E-01 | 1.00E+00 | 1.00E+00 | 3.05E-02 | 4.59E-02 | rs116298963 | 5.55E-54 |  |
| SLE | *PHRF1* | 50 | 2.32E-07 | 8.58E-05 | 7.12E-02 | 1.00E+00 | 1.00E+00 | rs58688157 | 2.97E-11 |  |
| SLE | *PKP3* | 498 | 2.21E-02 | 1.90E-03 | 7.16E-04 | 1.00E+00 | 1.00E+00 | rs58688157 | 2.97E-11 |  |
| SLE | *POM121L2* | 418 | 4.08E-05 | 1.00E+00 | 1.00E+00 | 3.49E-21 | 5.24E-21 | rs13202295 | 1.12E-34 |  |
| SLE | *POU5F1* | 418 | 2.72E-08 | 1.19E-09 | 6.87E-01 | 6.29E-77 | 9.43E-77 | rs114629349 | 1.70E-98 |  |
| SLE | *PPARGC1B* | 139 | 3.28E-01 | 1.00E+00 | 1.00E+00 | 2.53E-07 | 3.74E-07 | rs11956160 | 6.84E-04 | √ |
| SLE | *PPIL2* | 54 | 6.06E-01 | 1.00E+00 | 1.00E+00 | 7.59E-03 | 1.14E-02 | rs3747093 | 2.88E-14 |  |
| SLE | *PPT2* | 54 | 3.68E-02 | 7.84E-61 | 5.59E-35 | 2.46E-90 | 3.69E-90 | rs141910407 | 6.70E-103 |  |
| SLE | *PRDX6* | 54 | 7.84E-05 | 1.61E-10 | 4.58E-08 | 3.28E-01 | 4.91E-01 | rs10912578 | 1.65E-15 |  |
| SLE | *PRRC2A* | 54 | 6.84E-13 | 1.50E-20 | 9.87E-22 | 1.00E-86 | 1.51E-86 | rs141910407 | 6.70E-103 |  |
| SLE | *PRRT1* | 12 | 8.25E-01 | 7.51E-37 | 4.69E-02 | 5.43E-90 | 8.15E-90 | rs141910407 | 6.70E-103 |  |
| SLE | *PRSS16* | 53 | 4.76E-02 | 7.17E-03 | 2.99E-01 | 5.84E-21 | 8.76E-21 | rs13217620 | 2.21E-34 |  |
| SLE | *PSD4* | 53 | 4.34E-01 | 1.00E+00 | 1.00E+00 | 6.86E-71 | 1.03E-70 | rs276819 | 5.81E-05 | √ |
| SLE | *PSMB8* | 54 | 5.94E-01 | 1.22E-08 | 1.00E+00 | 3.10E-48 | 4.65E-48 | rs9273327 | 1.76E-100 |  |
| SLE | *PSMB9* | 62 | 7.85E-01 | 5.48E-18 | 1.79E-08 | 1.18E-47 | 1.77E-47 | rs9273327 | 1.76E-100 |  |
| SLE | *PSMD3* | 75 | 3.07E-08 | 3.35E-07 | 1.00E+00 | 1.00E+00 | 1.00E+00 | rs112679482 | 8.29E-06 | √ |
| SLE | *PSORS1C1* | 75 | 5.00E-12 | 3.89E-20 | 1.01E-11 | 2.59E-77 | 3.88E-77 | rs147027375 | 1.55E-96 |  |
| SLE | *PTDSS2* | 46 | 1.16E-02 | 7.17E-03 | 6.50E-03 | 1.00E+00 | 1.00E+00 | rs58688157 | 2.97E-11 |  |
| SLE | *PTTG1* | 242 | 1.49E-03 | 1.96E-03 | 1.00E+00 | 5.66E-04 | 8.50E-04 | rs71603662 | 3.64E-07 | √ |
| SLE | *PYCARD* | 240 | 8.07E-02 | 6.17E-12 | 2.11E-11 | 2.00E-37 | 3.01E-37 | rs1143679 | 5.03E-48 |  |
| SLE | *RASSF7* | 240 | 1.61E-05 | 6.48E-04 | 3.10E-03 | 1.00E+00 | 1.00E+00 | rs58688157 | 2.97E-11 |  |
| SLE | *RAVER1* | 89 | 5.85E-11 | 2.51E-01 | 1.00E+00 | 2.02E-02 | 3.03E-02 | rs35251378 | 3.61E-13 |  |
| SLE | *RDBP* | 155 | 6.09E-02 | 4.01E-18 | 4.68E-05 | 1.04E-90 | 1.56E-90 | rs141910407 | 6.70E-103 |  |
| SLE | *RGL2* | 145 | 6.91E-01 | 1.00E+00 | 1.00E+00 | 2.07E-10 | 3.10E-10 | rs114720630 | 6.72E-72 |  |
| SLE | *RIMBP3C* | 145 | 2.19E-02 | 1.00E+00 | 1.00E+00 | 5.63E-04 | 8.44E-04 | rs3747093 | 2.88E-14 |  |
| SLE | *RING1* | 21 | 8.25E-01 | 1.00E+00 | 1.00E+00 | 2.77E-12 | 4.15E-12 | rs115575857 | 2.42E-98 |  |
| SLE | *RNF5* | 21 | 1.37E-01 | 1.68E-46 | 3.59E-36 | 5.00E-90 | 7.51E-90 | rs141910407 | 6.70E-103 |  |
| SLE | *RNH1* | 76 | 2.57E-03 | 1.39E-02 | 1.46E-02 | 1.00E+00 | 1.00E+00 | rs58688157 | 2.97E-11 |  |
| SLE | *RP1-265C24.8* | 76 | 2.37E-07 | 4.89E-04 | 1.00E+00 | 3.39E-25 | 5.09E-25 | rs67981811 | 9.96E-35 |  |
| SLE | *RP11-148O21.2* | 76 | 3.33E-05 | 2.11E-01 | 1.00E+00 | 3.64E-06 | 5.46E-06 | rs2736332 | 4.83E-18 |  |
| SLE | *RP11-240M16.1* | 76 | 2.42E-01 | 1.00E+00 | 1.00E+00 | 3.90E-07 | 5.91E-07 | rs58721818 | 3.38E-18 |  |
| SLE | *RP11-259P6.1* | 9 | 1.79E-03 | 2.80E-03 | 1.00E+00 | 1.00E+00 | 1.00E+00 | rs12575883 | 7.01E-04 | √ |
| SLE | *RP11-356I2.1* | 9 | 5.24E-01 | 1.00E+00 | 1.00E+00 | 3.78E-06 | 5.70E-06 | rs58721818 | 3.38E-18 |  |
| SLE | *RP11-356I2.2* | 9 | 1.80E-01 | 6.52E-03 | 1.59E-02 | 1.84E-07 | 2.78E-07 | rs58721818 | 3.38E-18 |  |
| SLE | *RP11-356I2.4* | 4 | 6.69E-01 | 3.73E-04 | 3.51E-04 | 2.78E-07 | 4.22E-07 | rs58721818 | 3.38E-18 |  |
| SLE | *RP11-388M20.9* | 4 | 8.72E-04 | 2.14E-12 | 8.07E-16 | 4.91E-38 | 7.36E-38 | rs1143679 | 5.03E-48 |  |
| SLE | *RP11-452L6.1* | 4 | 2.18E-03 | 1.00E+00 | 1.00E+00 | 6.18E-28 | 9.27E-28 | rs1143679 | 5.03E-48 |  |
| SLE | *RP11-457M11.5* | 4 | 4.50E-01 | 3.26E-06 | 1.00E+00 | 8.08E-19 | 1.21E-18 | rs45527431 | 5.68E-32 |  |
| SLE | *RP11-74N20.1* | 4 | 3.73E-16 | 5.15E-03 | 7.43E-02 | 9.10E-01 | 1.00E+00 | rs17849501 | 1.81E-59 |  |
| SLE | *RP5-1186N24.3* | 4 | 3.25E-01 | 8.69E-03 | 6.85E-04 | 1.00E+00 | 1.00E+00 | rs115387042 | 1.72E-37 |  |
| SLE | *RPS18* | 4 | 4.81E-01 | 1.00E+00 | 1.00E+00 | 8.04E-11 | 1.21E-10 | rs114720630 | 6.72E-72 |  |
| SLE | *RXRB* | 4 | 9.82E-01 | 1.00E+00 | 1.00E+00 | 4.60E-12 | 6.89E-12 | rs115575857 | 2.42E-98 |  |
| SLE | *SAPCD1* | 68 | 2.38E-19 | 4.80E-04 | 1.00E+00 | 4.11E-89 | 6.16E-89 | rs141910407 | 6.70E-103 |  |
| SLE | *SCAND3* | 126 | 1.68E-01 | 2.47E-04 | 1.37E-02 | 6.16E-26 | 9.24E-26 | rs115387042 | 1.72E-37 |  |
| SLE | *SDF2L1* | 94 | 7.78E-01 | 1.00E+00 | 1.00E+00 | 7.66E-03 | 1.15E-02 | rs3747093 | 2.88E-14 |  |
| SLE | *SIGIRR* | 49 | 9.26E-03 | 2.01E-03 | 5.81E-04 | 1.00E+00 | 1.00E+00 | rs58688157 | 2.97E-11 |  |
| SLE | *SKIV2L* | 32 | 3.53E-01 | 8.89E-72 | 2.23E-41 | 1.13E-90 | 1.69E-90 | rs141910407 | 6.70E-103 |  |
| SLE | *SLC35G5* | 207 | 4.06E-01 | 6.56E-03 | 1.00E+00 | 1.00E+00 | 1.00E+00 | rs2736332 | 4.83E-18 |  |
| SLE | *SLC39A7* | 207 | 9.89E-01 | 1.00E+00 | 1.00E+00 | 9.83E-12 | 1.47E-11 | rs115575857 | 2.42E-98 |  |
| SLE | *SLC45A3* | 48 | 4.22E-03 | 1.98E-02 | 1.00E+00 | 1.00E+00 | 1.00E+00 | rs12729943 | 5.35E-04 | √ |
| SLE | *SLU7* | 48 | 5.63E-01 | 1.00E+00 | 1.00E+00 | 3.36E-03 | 5.04E-03 | rs28694840 | 5.07E-05 | √ |
| SLE | *SMG7* | 48 | 1.73E-14 | 3.25E-01 | 1.00E+00 | 1.00E+00 | 1.00E+00 | rs17849501 | 1.81E-59 |  |
| SLE | *SMO* | 40 | 1.19E-01 | 1.00E+00 | 1.00E+00 | 8.74E-12 | 1.31E-11 | rs35000415 | 1.86E-45 |  |
| SLE | *STAT1* | 23 | 8.97E-03 | 1.72E-01 | 1.64E-01 | 1.27E-52 | 1.91E-52 | rs36010116 | 9.48E-05 | √ |
| SLE | *STAT4* | 34 | 1.48E-01 | 1.31E-01 | 1.64E-01 | 7.06E-53 | 1.06E-52 | rs13017709 | 2.95E-03 | √ |
| SLE | *STK19* | 298 | 4.11E-03 | 1.70E-70 | 2.37E-02 | 5.25E-91 | 7.87E-91 | rs141910407 | 6.70E-103 |  |
| SLE | *SYNGAP1* | 298 | 3.00E-01 | 1.00E+00 | 1.00E+00 | 4.66E-03 | 7.04E-03 | rs116298963 | 5.55E-54 |  |
| SLE | *TAP1* | 47 | 7.53E-01 | 9.29E-01 | 1.00E+00 | 1.84E-46 | 2.76E-46 | rs9273327 | 1.76E-100 |  |
| SLE | *TAP2* | 47 | 7.54E-01 | 1.90E-04 | 1.00E+00 | 3.77E-49 | 5.65E-49 | rs9273327 | 1.76E-100 |  |
| SLE | *TAPBP* | 47 | 6.79E-01 | 1.00E+00 | 1.00E+00 | 1.49E-02 | 2.23E-02 | rs114720630 | 6.72E-72 |  |
| SLE | *TBCE* | 47 | 3.74E-04 | 6.81E-03 | 1.00E+00 | 1.00E+00 | 1.00E+00 | rs11579128 | 2.54E-05 | √ |
| SLE | *TCF19* | 47 | 2.14E-14 | 3.82E-16 | 1.19E-09 | 5.70E-77 | 8.54E-77 | rs114629349 | 1.70E-98 |  |
| SLE | *TMEM191C* | 47 | 3.49E-04 | 6.05E-06 | 7.25E-06 | 2.49E-04 | 3.74E-04 | rs3747093 | 2.88E-14 |  |
| SLE | *TNF* | 47 | 1.75E-01 | 9.56E-48 | 1.43E-21 | 1.04E-84 | 1.56E-84 | rs141910407 | 6.70E-103 |  |
| SLE | *TNFAIP3* | 47 | 2.64E-01 | 1.00E+00 | 1.00E+00 | 1.81E-07 | 2.78E-07 | rs58721818 | 3.38E-18 |  |
| SLE | *TNFSF4* | 296 | 2.53E-01 | 4.50E-01 | 1.00E+00 | 6.17E-05 | 9.61E-05 | rs10912578 | 1.65E-15 |  |
| SLE | *TNIP1* | 296 | 6.82E-01 | 1.00E+00 | 1.00E+00 | 4.66E-04 | 7.82E-04 | rs6869426 | 1.17E-03 | √ |
| SLE | *TNPO3* | 296 | 2.62E-05 | 1.11E-01 | 1.00E+00 | 3.46E-33 | 5.19E-33 | rs35000415 | 1.86E-45 |  |
| SLE | *TNXB* | 296 | 9.48E-01 | 6.02E-56 | 2.60E-16 | 2.52E-91 | 3.77E-91 | rs141910407 | 6.70E-103 |  |
| SLE | *TRIM27* | 296 | 1.49E-03 | 2.71E-02 | 5.72E-02 | 5.31E-27 | 7.97E-27 | rs115466242 | 5.26E-41 |  |
| SLE | *TRIM38* | 296 | 1.89E-06 | 4.65E-04 | 6.88E-04 | 7.91E-12 | 1.19E-11 | rs13195401 | 3.45E-31 |  |
| SLE | *TRIM72* | 296 | 4.08E-02 | 1.14E-05 | 1.45E-01 | 2.74E-37 | 4.11E-37 | rs1143679 | 5.03E-48 |  |
| SLE | *TSPAN33* | 296 | 2.13E-02 | 1.00E+00 | 1.00E+00 | 5.08E-18 | 7.62E-18 | rs35000415 | 1.86E-45 |  |
| SLE | *TYK2* | 48 | 2.81E-10 | 1.00E+00 | 1.00E+00 | 1.47E-02 | 2.20E-02 | rs35251378 | 3.61E-13 |  |
| SLE | *UBE2L3* | 48 | 7.06E-01 | 1.00E+00 | 1.00E+00 | 2.53E-03 | 3.80E-03 | rs3747093 | 2.88E-14 |  |
| SLE | *VARS* | 48 | 1.27E-20 | 8.84E-13 | 1.00E+00 | 2.32E-89 | 3.49E-89 | rs141910407 | 6.70E-103 |  |
| SLE | *VPS52* | 48 | 6.50E-01 | 1.00E+00 | 1.00E+00 | 3.97E-11 | 5.96E-11 | rs114720630 | 6.72E-72 |  |
| SLE | *VWA7* | 48 | 4.05E-23 | 8.82E-09 | 1.00E+00 | 2.04E-89 | 3.06E-89 | rs141910407 | 6.70E-103 |  |
| SLE | *WDR46* | 48 | 5.42E-01 | 1.00E+00 | 1.00E+00 | 2.52E-10 | 3.77E-10 | rs114720630 | 6.72E-72 |  |
| SLE | *XXbac-BPG181M17.5* | 48 | 6.83E-02 | 1.00E+00 | 1.00E+00 | 9.87E-42 | 1.48E-41 | rs9273327 | 1.76E-100 |  |
| SLE | *XXbac-BPG246D15.9* | 32 | 2.67E-01 | 1.39E-19 | 1.51E-17 | 4.90E-48 | 7.35E-48 | rs9273327 | 1.76E-100 |  |
| SLE | *XXbac-BPG300A18.12* | 32 | 8.45E-02 | 2.26E-39 | 1.00E-20 | 2.78E-90 | 4.18E-90 | rs141910407 | 6.70E-103 |  |
| SLE | *XXbac-BPG32J3.22* | 32 | 5.30E-21 | 1.21E-34 | 1.35E-08 | 4.02E-88 | 6.04E-88 | rs141910407 | 6.70E-103 |  |
| SLE | *YDJC* | 32 | 7.74E-01 | 1.00E+00 | 1.00E+00 | 6.53E-03 | 9.80E-03 | rs3747093 | 2.88E-14 |  |
| SLE | *YPEL1* | 32 | 8.43E-01 | 1.00E+00 | 1.00E+00 | 9.51E-03 | 1.43E-02 | rs3747093 | 2.88E-14 |  |
| SLE | *ZBTB12* | 32 | 3.59E-04 | 5.04E-46 | 2.31E-21 | 2.29E-90 | 3.43E-90 | rs141910407 | 6.70E-103 |  |
| SLE | *ZBTB22* | 32 | 6.31E-01 | 1.00E+00 | 1.00E+00 | 3.72E-03 | 5.58E-03 | rs114720630 | 6.72E-72 |  |
| SLE | *ZC3H12C* | 27 | 1.77E-05 | 3.76E-02 | 1.00E+00 | 1.00E+00 | 1.00E+00 | rs6589115 | 4.27E-05 | √ |
| SLE | *ZGLP1* | 27 | 4.97E-11 | 2.47E-03 | 1.00E+00 | 3.42E-03 | 5.13E-03 | rs35251378 | 3.61E-13 |  |
| SLE | *ZKSCAN3* | 27 | 3.16E-12 | 2.19E-03 | 3.07E-02 | 4.23E-25 | 6.35E-25 | rs115387042 | 1.72E-37 |  |
| SLE | *ZKSCAN4* | 27 | 1.13E-10 | 8.01E-06 | 1.00E+00 | 1.35E-24 | 2.03E-24 | rs116725083 | 1.78E-36 |  |
| SLE | *ZNF165* | 27 | 3.77E-07 | 2.87E-05 | 1.00E+00 | 1.85E-25 | 2.77E-25 | rs67981811 | 9.96E-35 |  |
| SLE | *ZNF184* | 27 | 2.89E-09 | 1.00E+00 | 1.00E+00 | 3.59E-21 | 5.38E-21 | rs13202295 | 1.12E-34 |  |
| SLE | *ZNF192* | 27 | 1.11E-07 | 1.83E-02 | 1.00E+00 | 3.55E-25 | 5.32E-25 | rs115891768 | 9.37E-35 |  |
| SLE | *ZNF193* | 27 | 3.11E-10 | 6.65E-01 | 1.00E+00 | 4.50E-25 | 6.74E-25 | rs116137698 | 1.46E-35 |  |
| SLE | *ZNF311* | 78 | 1.30E-04 | 6.61E-05 | 2.67E-07 | 5.33E-27 | 7.99E-27 | rs115350668 | 3.23E-41 |  |
| SLE | *ZNF322* | 78 | 8.80E-01 | 4.26E-21 | 1.00E+00 | 1.31E-18 | 1.97E-18 | rs45527431 | 5.68E-32 |  |
| SLE | *ZNF323* | 78 | 2.86E-12 | 1.26E-02 | 1.00E+00 | 2.42E-24 | 3.63E-24 | rs115387042 | 1.72E-37 |  |
| SLE | *ZNF383* | 78 | 5.73E-07 | 1.00E+00 | 1.00E+00 | 1.00E+00 | 1.00E+00 | rs4803210 | 1.56E-05 | √ |
| SLE | *ZNF391* | 78 | 1.58E-08 | 1.00E+00 | 1.00E+00 | 1.94E-21 | 2.92E-21 | rs13202295 | 1.12E-34 |  |
| SLE | *ZNF420* | 78 | 1.03E-07 | 1.00E+00 | 1.00E+00 | 1.00E+00 | 1.00E+00 | rs4803210 | 1.56E-05 | √ |
| SLE | *ZNF568* | 78 | 1.49E-06 | 1.00E+00 | 1.00E+00 | 1.00E+00 | 1.00E+00 | rs4803210 | 1.56E-05 | √ |
| SLE | *ZNF843* | 78 | 6.71E-05 | 5.93E-02 | 1.00E+00 | 1.37E-28 | 2.06E-28 | rs1143679 | 5.03E-48 |  |
| SLE | *ZPBP2* | 43 | 1.41E-06 | 6.59E-03 | 1.00E+00 | 1.00E+00 | 1.00E+00 | rs112679482 | 8.29E-06 | √ |
| SLE | *ZSCAN12* | 57 | 2.34E-11 | 2.72E-03 | 1.00E+00 | 6.83E-25 | 1.02E-24 | rs115387042 | 1.72E-37 |  |
| SLE | *ZSCAN16* | 57 | 1.51E-07 | 9.85E-04 | 1.00E+00 | 2.50E-25 | 3.75E-25 | rs67981811 | 9.96E-35 |  |
| SLE | *ZSCAN23* | 57 | 8.01E-10 | 5.90E-05 | 1.55E-03 | 1.80E-25 | 2.70E-25 | rs115387042 | 1.72E-37 |  |
| PBC | *ABHD16A* | 20 | 5.12E-02 | 3.04E-02 | 1.00E+00 | 5.32E-07 | 7.45E-07 | rs3117572 | 1.71E-16 |  |
| PBC | *ADPRH* | 26 | 1.84E-04 | 1.00E+00 | 1.00E+00 | 8.08E-05 | 1.21E-04 | rs2293370 | 4.26E-15 |  |
| PBC | *AGAP2* | 26 | 2.19E-05 | 2.56E-03 | 6.12E-02 | 1.00E+00 | 1.00E+00 | rs701008 | 6.76E-05 | √ |
| PBC | *AGER* | 474 | 6.20E-19 | 1.14E-02 | 1.00E+00 | 1.94E-05 | 2.92E-05 | rs28421666 | 8.67E-47 |  |
| PBC | *AGPAT1* | 17 | 3.98E-13 | 1.00E+00 | 1.00E+00 | 1.25E-04 | 1.87E-04 | rs28421666 | 8.67E-47 |  |
| PBC | *ANXA7* | 51 | 5.25E-07 | 1.16E-01 | 9.10E-02 | 1.00E+00 | 1.00E+00 | rs1259601 | 4.77E-03 | √ |
| PBC | *APOM* | 130 | 9.67E-01 | 1.00E+00 | 1.00E+00 | 1.73E-06 | 2.45E-06 | rs3117572 | 1.71E-16 |  |
| PBC | *ARHGAP31* | 235 | 2.91E-04 | 3.20E-02 | 1.00E+00 | 3.15E-05 | 4.76E-05 | rs2293370 | 4.26E-15 |  |
| PBC | *ASPSCR1* | 81 | 7.19E-01 | 1.25E-08 | 1.00E+00 | 1.00E+00 | 1.00E+00 | rs11655935 | 2.55E-04 | √ |
| PBC | *ATF6B* | 581 | 3.00E-13 | 1.43E-02 | 1.00E+00 | 1.39E-05 | 2.09E-05 | rs9469099 | 1.35E-32 |  |
| PBC | *ATP6V1F* | 581 | 6.83E-01 | 6.17E-03 | 9.73E-03 | 3.71E-12 | 5.56E-12 | rs10488631 | 5.08E-23 |  |
| PBC | *B3GALT4* | 581 | 4.42E-11 | 2.74E-03 | 1.00E+00 | 4.71E-19 | 7.06E-19 | rs1431402 | 6.21E-39 |  |
| PBC | *BAG6* | 85 | 3.23E-01 | 4.74E-01 | 1.00E+00 | 2.28E-06 | 3.21E-06 | rs3117572 | 1.71E-16 |  |
| PBC | *BCL9L* | 85 | 4.34E-01 | 4.38E-04 | 1.00E+00 | 2.91E-04 | 4.37E-04 | rs4938573 | 1.15E-13 |  |
| PBC | *BRD2* | 100 | 2.02E-11 | 4.06E-08 | 6.22E-08 | 1.00E+00 | 1.00E+00 | rs7774434 | 2.37E-56 |  |
| PBC | *C11orf20* | 53 | 1.90E-02 | 4.57E-06 | 3.48E-04 | 1.00E+00 | 1.00E+00 | rs510372 | 1.68E-09 |  |
| PBC | *C19orf63* | 53 | 5.24E-01 | 1.00E+00 | 1.00E+00 | 7.01E-09 | 1.05E-08 | rs2459144 | 2.15E-04 | √ |
| PBC | *C2* | 775 | 1.02E-11 | 5.58E-07 | 1.00E+00 | 4.91E-07 | 7.57E-07 | rs9469099 | 1.35E-32 |  |
| PBC | *C4A* | 95 | 1.08E-02 | 1.00E+00 | 1.00E+00 | 2.15E-04 | 3.22E-04 | rs9469099 | 1.35E-32 |  |
| PBC | *C4B* | 95 | 6.48E-08 | 1.00E+00 | 1.00E+00 | 2.55E-05 | 3.82E-05 | rs9469099 | 1.35E-32 |  |
| PBC | *C6orf47* | 95 | 8.50E-01 | 1.00E+00 | 1.00E+00 | 2.36E-06 | 3.31E-06 | rs3117572 | 1.71E-16 |  |
| PBC | *C6orf48* | 95 | 1.86E-08 | 1.00E+00 | 1.00E+00 | 2.17E-06 | 3.23E-06 | rs11759575 | 1.92E-21 |  |
| PBC | *CCDC88B* | 95 | 1.53E-03 | 8.26E-06 | 1.00E+00 | 1.00E+00 | 1.00E+00 | rs510372 | 1.68E-09 |  |
| PBC | *CCHCR1* | 95 | 2.42E-01 | 4.27E-02 | 1.00E+00 | 2.87E-04 | 4.30E-04 | rs3763295 | 2.74E-15 |  |
| PBC | *CD80* | 95 | 4.44E-06 | 1.00E+00 | 1.00E+00 | 8.23E-04 | 1.24E-03 | rs2293370 | 4.26E-15 |  |
| PBC | *CDC37* | 95 | 5.43E-07 | 5.40E-01 | 1.00E+00 | 1.00E+00 | 1.00E+00 | rs2304256 | 1.05E-10 |  |
| PBC | *CDC42BPB* | 65 | 9.29E-01 | 1.00E+00 | 1.00E+00 | 2.33E-07 | 3.48E-07 | rs2297067 | 6.34E-19 |  |
| PBC | *CDK4* | 65 | 1.46E-05 | 1.22E-02 | 2.24E-01 | 1.00E+00 | 1.00E+00 | rs701008 | 6.76E-05 | √ |
| PBC | *CFB* | 65 | 2.69E-08 | 8.92E-07 | 1.00E+00 | 7.44E-05 | 1.12E-04 | rs9469099 | 1.35E-32 |  |
| PBC | *CIITA* | 65 | 3.82E-02 | 1.41E-09 | 5.75E-10 | 1.00E+00 | 1.00E+00 | rs12924729 | 2.39E-14 |  |
| PBC | *CLEC16A* | 65 | 7.49E-05 | 4.19E-15 | 5.86E-04 | 2.17E-02 | 3.25E-02 | rs12924729 | 2.39E-14 |  |
| PBC | *CLIC1* | 65 | 6.05E-05 | 1.00E+00 | 1.00E+00 | 6.96E-07 | 1.01E-06 | rs3117572 | 1.71E-16 |  |
| PBC | *COL11A2* | 65 | 3.32E-17 | 6.03E-10 | 6.07E-04 | 3.44E-19 | 5.16E-19 | rs7774434 | 2.37E-56 |  |
| PBC | *CSNK2B* | 25 | 7.94E-01 | 1.00E+00 | 1.00E+00 | 9.13E-07 | 1.29E-06 | rs3117572 | 1.71E-16 |  |
| PBC | *CTD-3088G3.2* | 25 | 1.21E-04 | 1.51E-02 | 6.46E-03 | 1.00E+00 | 1.00E+00 | rs12924729 | 2.39E-14 |  |
| PBC | *CTD-3088G3.3* | 25 | 1.75E-04 | 2.92E-05 | 6.26E-03 | 5.31E-01 | 7.97E-01 | rs12924729 | 2.39E-14 |  |
| PBC | *CTDSP2* | 25 | 7.16E-06 | 2.15E-02 | 1.00E+00 | 1.00E+00 | 1.00E+00 | rs701008 | 6.76E-05 | √ |
| PBC | *CTSH* | 338 | 1.13E-01 | 1.14E-02 | 9.34E-03 | 1.00E+00 | 1.00E+00 | rs4779112 | 1.63E-07 | √ |
| PBC | *CXCR5* | 338 | 1.59E-01 | 1.00E+00 | 1.00E+00 | 2.16E-03 | 3.23E-03 | rs4938573 | 1.15E-13 |  |
| PBC | *CYP21A2* | 338 | 3.70E-11 | 1.39E-01 | 1.00E+00 | 1.63E-05 | 2.44E-05 | rs9469099 | 1.35E-32 |  |
| PBC | *CYP27B1* | 13 | 1.34E-05 | 1.55E-02 | 3.18E-02 | 1.00E+00 | 1.00E+00 | rs701008 | 6.76E-05 | √ |
| PBC | *DAAM1* | 62 | 2.29E-01 | 1.72E-05 | 1.00E+00 | 1.00E+00 | 1.00E+00 | rs3850302 | 3.38E-04 | √ |
| PBC | *DCXR* | 62 | 9.59E-01 | 2.69E-08 | 1.00E+00 | 1.00E+00 | 1.00E+00 | rs7216806 | 1.25E-02 | √ |
| PBC | *DDAH2* | 150 | 7.45E-05 | 1.00E+00 | 1.00E+00 | 7.68E-07 | 1.12E-06 | rs3117572 | 1.71E-16 |  |
| PBC | *DDX6* | 269 | 4.06E-02 | 2.36E-07 | 1.00E+00 | 2.88E-04 | 4.32E-04 | rs4938573 | 1.15E-13 |  |
| PBC | *DEXI* | 269 | 1.22E-01 | 1.11E-03 | 1.00E+00 | 1.00E+00 | 1.00E+00 | rs12924729 | 2.39E-14 |  |
| PBC | *DOM3Z* | 269 | 5.00E-05 | 6.70E-10 | 2.70E-02 | 1.03E-04 | 1.54E-04 | rs9469099 | 1.35E-32 |  |
| PBC | *DUS1L* | 48 | 4.57E-01 | 1.14E-03 | 1.00E+00 | 1.00E+00 | 1.00E+00 | rs7216806 | 1.25E-02 | √ |
| PBC | *EGFL8* | 25 | 1.72E-13 | 1.00E+00 | 1.00E+00 | 9.69E-05 | 1.45E-04 | rs28421666 | 8.67E-47 |  |
| PBC | *EHMT2* | 25 | 9.24E-17 | 1.85E-25 | 1.87E-08 | 3.77E-07 | 5.83E-07 | rs9469099 | 1.35E-32 |  |
| PBC | *ERAP2* | 25 | 1.49E-02 | 1.57E-02 | 1.00E+00 | 1.00E+00 | 1.00E+00 | rs27772 | 4.42E-03 | √ |
| PBC | *ERBB2* | 72 | 1.44E-04 | 1.54E-03 | 8.52E-03 | 2.13E-02 | 3.20E-02 | rs7224568 | 4.10E-03 | √ |
| PBC | *ESRRA* | 72 | 4.59E-02 | 4.68E-04 | 1.38E-01 | 1.00E+00 | 1.00E+00 | rs510372 | 1.68E-09 |  |
| PBC | *FAIM3* | 72 | 2.92E-06 | 1.00E+00 | 1.00E+00 | 1.00E+00 | 1.00E+00 | rs291089 | 3.13E-04 | √ |
| PBC | *FAM71E1* | 72 | 7.91E-01 | 1.00E+00 | 1.00E+00 | 6.30E-09 | 9.45E-09 | rs2459144 | 2.15E-04 | √ |
| PBC | *FDX1L* | 116 | 8.58E-08 | 1.00E+00 | 1.00E+00 | 1.00E+00 | 1.00E+00 | rs2304256 | 1.05E-10 |  |
| PBC | *FKBPL* | 70 | 8.07E-14 | 1.00E+00 | 1.00E+00 | 2.34E-05 | 3.52E-05 | rs9469099 | 1.35E-32 |  |
| PBC | *FLNC* | 21 | 9.89E-01 | 5.63E-01 | 1.00E+00 | 1.41E-12 | 2.12E-12 | rs10488631 | 5.08E-23 |  |
| PBC | *GOPC* | 21 | 4.93E-01 | 1.46E-02 | 2.28E-02 | 1.00E+00 | 1.00E+00 | rs13202536 | 1.21E-04 | √ |
| PBC | *GPANK1* | 71 | 7.73E-01 | 1.00E+00 | 1.00E+00 | 1.54E-06 | 2.17E-06 | rs3117572 | 1.71E-16 |  |
| PBC | *GPR137* | 693 | 5.73E-05 | 1.57E-05 | 1.00E+00 | 1.00E+00 | 1.00E+00 | rs510372 | 1.68E-09 |  |
| PBC | *GPS1* | 693 | 5.75E-01 | 1.10E-06 | 1.00E+00 | 1.00E+00 | 1.00E+00 | rs7216806 | 1.25E-02 | √ |
| PBC | *GPSM3* | 693 | 7.81E-23 | 1.67E-03 | 1.00E+00 | 2.25E-05 | 3.38E-05 | rs28421666 | 8.67E-47 |  |
| PBC | *GRB7* | 693 | 5.44E-04 | 7.58E-03 | 1.81E-02 | 9.75E-03 | 1.46E-02 | rs7224568 | 4.10E-03 | √ |
| PBC | *GSDMB* | 693 | 6.72E-08 | 2.98E-02 | 1.00E+00 | 3.21E-02 | 4.81E-02 | rs7224568 | 4.10E-03 | √ |
| PBC | *GXYLT1* | 693 | 1.96E-06 | 1.87E-01 | 8.16E-01 | 1.00E+00 | 1.00E+00 | rs11832772 | 7.31E-07 | √ |
| PBC | *HCG22* | 693 | 7.55E-01 | 2.24E-01 | 1.00E+00 | 7.11E-04 | 1.07E-03 | rs2233976 | 2.37E-14 |  |
| PBC | *HCG27* | 693 | 9.47E-01 | 8.36E-01 | 1.00E+00 | 3.40E-03 | 5.10E-03 | rs3763295 | 2.74E-15 |  |
| PBC | *HDHD2* | 114 | 9.02E-01 | 3.87E-12 | 1.00E+00 | 1.00E+00 | 1.00E+00 | rs9967367 | 9.60E-03 | √ |
| PBC | *HIST1H1B* | 114 | 7.82E-12 | 1.00E+00 | 1.00E+00 | 1.00E+00 | 1.00E+00 | rs201004 | 1.02E-07 | √ |
| PBC | *HIST1H2AG* | 114 | 6.26E-05 | 2.15E-01 | 1.80E-02 | 1.00E+00 | 1.00E+00 | rs12200985 | 1.41E-07 | √ |
| PBC | *HIST1H2AI* | 114 | 9.76E-18 | 1.74E-03 | 1.00E+00 | 1.00E+00 | 1.00E+00 | rs201004 | 1.02E-07 | √ |
| PBC | *HIST1H2AJ* | 114 | 1.11E-17 | 8.25E-03 | 1.00E+00 | 1.00E+00 | 1.00E+00 | rs201004 | 1.02E-07 | √ |
| PBC | *HIST1H2AK* | 114 | 4.69E-10 | 1.00E+00 | 1.00E+00 | 1.00E+00 | 1.00E+00 | rs201004 | 1.02E-07 | √ |
| PBC | *HIST1H2AL* | 114 | 5.34E-14 | 1.00E+00 | 1.00E+00 | 1.00E+00 | 1.00E+00 | rs201004 | 1.02E-07 | √ |
| PBC | *HIST1H2AM* | 114 | 8.57E-14 | 1.00E+00 | 1.00E+00 | 1.00E+00 | 1.00E+00 | rs201004 | 1.02E-07 | √ |
| PBC | *HIST1H2BL* | 268 | 3.04E-13 | 1.00E+00 | 1.00E+00 | 1.00E+00 | 1.00E+00 | rs201004 | 1.02E-07 | √ |
| PBC | *HIST1H2BM* | 268 | 5.87E-16 | 4.16E-02 | 1.00E+00 | 1.00E+00 | 1.00E+00 | rs201004 | 1.02E-07 | √ |
| PBC | *HIST1H2BN* | 268 | 3.90E-11 | 1.00E+00 | 1.00E+00 | 1.00E+00 | 1.00E+00 | rs201004 | 1.02E-07 | √ |
| PBC | *HIST1H2BO* | 268 | 2.42E-15 | 1.00E+00 | 1.00E+00 | 1.00E+00 | 1.00E+00 | rs201004 | 1.02E-07 | √ |
| PBC | *HIST1H3A* | 239 | 5.70E-05 | 4.31E-04 | 6.80E-01 | 1.00E+00 | 1.00E+00 | rs10484439 | 4.17E-07 | √ |
| PBC | *HIST1H3H* | 239 | 4.59E-17 | 5.30E-06 | 4.62E-04 | 1.00E+00 | 1.00E+00 | rs201004 | 1.02E-07 | √ |
| PBC | *HIST1H3I* | 239 | 9.45E-15 | 1.00E+00 | 1.00E+00 | 1.00E+00 | 1.00E+00 | rs201004 | 1.02E-07 | √ |
| PBC | *HIST1H3J* | 239 | 4.96E-14 | 1.00E+00 | 1.00E+00 | 1.00E+00 | 1.00E+00 | rs201004 | 1.02E-07 | √ |
| PBC | *HIST1H4J* | 447 | 2.25E-12 | 1.00E+00 | 1.00E+00 | 1.00E+00 | 1.00E+00 | rs201004 | 1.02E-07 | √ |
| PBC | *HIST1H4K* | 86 | 1.81E-11 | 1.00E+00 | 1.00E+00 | 1.00E+00 | 1.00E+00 | rs201004 | 1.02E-07 | √ |
| PBC | *HLA-B* | 44 | 1.08E-09 | 1.11E-03 | 1.00E+00 | 1.00E+00 | 1.00E+00 | rs3117572 | 1.71E-16 |  |
| PBC | *HLA-C* | 78 | 1.65E-07 | 2.22E-02 | 1.00E+00 | 1.00E+00 | 1.00E+00 | rs3763295 | 2.74E-15 |  |
| PBC | *HLA-DMA* | 78 | 2.97E-11 | 5.72E-08 | 6.22E-08 | 1.00E+00 | 1.00E+00 | rs7774434 | 2.37E-56 |  |
| PBC | *HLA-DMB* | 104 | 4.59E-10 | 3.63E-08 | 6.21E-08 | 1.00E+00 | 1.00E+00 | rs7774434 | 2.37E-56 |  |
| PBC | *HLA-DOA* | 325 | 3.96E-11 | 1.88E-08 | 6.25E-08 | 1.00E+00 | 1.00E+00 | rs7774434 | 2.37E-56 |  |
| PBC | *HLA-DOB* | 325 | 6.82E-05 | 1.04E-05 | 2.02E-05 | 1.25E-06 | 1.84E-06 | rs7774434 | 2.37E-56 |  |
| PBC | *HLA-DPA1* | 516 | 7.35E-18 | 7.26E-08 | 6.62E-08 | 8.12E-14 | 1.22E-13 | rs7774434 | 2.37E-56 |  |
| PBC | *HLA-DPB1* | 516 | 1.97E-20 | 7.50E-08 | 9.51E-08 | 9.54E-15 | 1.43E-14 | rs7774434 | 2.37E-56 |  |
| PBC | *HLA-DQA1* | 14 | 3.22E-23 | 5.84E-19 | 4.65E-08 | 1.71E-04 | 2.56E-04 | rs7774434 | 2.37E-56 |  |
| PBC | *HLA-DQA2* | 14 | 9.39E-03 | 1.35E-05 | 2.06E-05 | 1.20E-06 | 1.75E-06 | rs7774434 | 2.37E-56 |  |
| PBC | *HLA-DQB1* | 14 | 8.98E-38 | 1.13E-48 | 9.46E-45 | 4.76E-05 | 7.14E-05 | rs7774434 | 2.37E-56 |  |
| PBC | *HLA-DQB2* | 14 | 8.41E-02 | 3.95E-05 | 8.74E-04 | 1.81E-07 | 2.61E-07 | rs7774434 | 2.37E-56 |  |
| PBC | *HLA-DRB1* | 14 | 5.42E-25 | 3.34E-47 | 9.30E-45 | 5.17E-05 | 7.76E-05 | rs7774434 | 2.37E-56 |  |
| PBC | *HLA-DRB5* | 14 | 1.18E-13 | 2.28E-32 | 2.15E-19 | 1.38E-04 | 2.07E-04 | rs7774434 | 2.37E-56 |  |
| PBC | *HSD17B8* | 14 | 6.58E-20 | 9.91E-18 | 1.83E-22 | 1.52E-19 | 2.28E-19 | rs7774434 | 2.37E-56 |  |
| PBC | *HSPA1A* | 150 | 3.33E-09 | 1.00E+00 | 1.00E+00 | 1.15E-06 | 1.71E-06 | rs3117572 | 1.71E-16 |  |
| PBC | *HSPA1B* | 150 | 7.74E-10 | 5.63E-10 | 1.00E+00 | 2.93E-07 | 4.47E-07 | rs3117572 | 1.71E-16 |  |
| PBC | *HSPA1L* | 130 | 2.86E-10 | 6.15E-11 | 1.00E+00 | 2.14E-07 | 3.10E-07 | rs3117572 | 1.71E-16 |  |
| PBC | *ICAM1* | 1347 | 8.84E-08 | 1.00E+00 | 1.00E+00 | 1.00E+00 | 1.00E+00 | rs2304256 | 1.05E-10 |  |
| PBC | *ICAM3* | 62 | 3.80E-08 | 1.00E+00 | 1.00E+00 | 1.00E+00 | 1.00E+00 | rs2304256 | 1.05E-10 |  |
| PBC | *ICAM4* | 274 | 2.97E-07 | 1.00E+00 | 1.00E+00 | 1.00E+00 | 1.00E+00 | rs2304256 | 1.05E-10 |  |
| PBC | *ICAM5* | 141 | 5.06E-09 | 2.11E-01 | 2.86E-01 | 1.00E+00 | 1.00E+00 | rs2304256 | 1.05E-10 |  |
| PBC | *IER3IP1* | 163 | 1.52E-01 | 1.12E-06 | 1.00E+00 | 1.00E+00 | 1.00E+00 | rs9967367 | 9.60E-03 | √ |
| PBC | *IFT80* | 32 | 1.68E-01 | 5.54E-03 | 3.26E-03 | 1.00E+00 | 1.00E+00 | rs485499 | 1.43E-23 |  |
| PBC | *IKZF3* | 24 | 4.65E-07 | 9.24E-02 | 1.00E+00 | 2.56E-02 | 3.84E-02 | rs7224568 | 4.10E-03 | √ |
| PBC | *IL12A* | 203 | 6.41E-13 | 6.54E-16 | 1.80E-06 | 2.36E-14 | 3.54E-14 | rs485499 | 1.43E-23 |  |
| PBC | *IL12RB2* | 176 | 8.76E-01 | 1.00E+00 | 1.00E+00 | 2.65E-14 | 3.97E-14 | rs6679356 | 7.49E-28 |  |
| PBC | *IL23R* | 176 | 1.86E-01 | 1.00E+00 | 1.00E+00 | 1.56E-15 | 2.34E-15 | rs6679356 | 7.49E-28 |  |
| PBC | *IQCJ-SCHIP1* | 176 | 9.08E-01 | 2.30E-07 | 2.17E-01 | 4.68E-05 | 8.08E-05 | rs485499 | 1.43E-23 |  |
| PBC | *IRF5* | 176 | 5.70E-08 | 6.51E-03 | 1.00E+00 | 8.73E-12 | 1.31E-11 | rs10488631 | 5.08E-23 |  |
| PBC | *JOSD2* | 78 | 8.65E-01 | 1.00E+00 | 1.00E+00 | 2.04E-09 | 3.06E-09 | rs2459144 | 2.15E-04 | √ |
| PBC | *KATNAL2* | 78 | 3.45E-01 | 4.94E-44 | 1.00E+00 | 1.00E+00 | 1.00E+00 | rs9967367 | 9.60E-03 | √ |
| PBC | *KCNC3* | 78 | 4.49E-01 | 1.00E+00 | 1.00E+00 | 2.19E-09 | 3.29E-09 | rs3745516 | 1.22E-20 |  |
| PBC | *KPNA4* | 78 | 3.51E-02 | 3.22E-15 | 2.29E-14 | 1.00E+00 | 1.00E+00 | rs485499 | 1.43E-23 |  |
| PBC | *LINC00240* | 78 | 1.03E-04 | 1.84E-02 | 6.06E-03 | 1.00E+00 | 1.00E+00 | rs12200985 | 1.41E-07 | √ |
| PBC | *LINC00481* | 78 | 6.32E-01 | 1.00E+00 | 1.00E+00 | 7.00E-04 | 1.05E-03 | rs3763295 | 2.74E-15 |  |
| PBC | *LRRC45* | 78 | 5.74E-01 | 1.78E-07 | 1.00E+00 | 1.00E+00 | 1.00E+00 | rs7216806 | 1.25E-02 | √ |
| PBC | *LRRC4B* | 86 | 6.76E-01 | 1.00E+00 | 1.00E+00 | 6.65E-10 | 9.97E-10 | rs2459144 | 2.15E-04 | √ |
| PBC | *LSM2* | 15 | 5.75E-08 | 7.20E-03 | 1.00E+00 | 5.50E-07 | 8.32E-07 | rs3117572 | 1.71E-16 |  |
| PBC | *LST1* | 70 | 6.32E-01 | 1.82E-03 | 1.00E+00 | 1.00E+00 | 1.00E+00 | rs3117572 | 1.71E-16 |  |
| PBC | *LY6G5B* | 81 | 2.50E-01 | 3.65E-04 | 1.00E+00 | 1.36E-06 | 1.91E-06 | rs3117572 | 1.71E-16 |  |
| PBC | *LY6G5C* | 256 | 1.49E-01 | 8.48E-01 | 1.00E+00 | 9.24E-07 | 1.30E-06 | rs3117572 | 1.71E-16 |  |
| PBC | *9-Mar* | 59 | 1.13E-05 | 1.22E-02 | 4.36E-02 | 1.00E+00 | 1.00E+00 | rs701008 | 6.76E-05 | √ |
| PBC | *MED1* | 59 | 3.34E-02 | 3.00E-02 | 1.92E-02 | 1.00E+00 | 1.00E+00 | rs7224568 | 4.10E-03 | √ |
| PBC | *METTL1* | 59 | 1.57E-05 | 1.97E-02 | 1.20E-01 | 1.00E+00 | 1.00E+00 | rs701008 | 6.76E-05 | √ |
| PBC | *METTL21B* | 59 | 7.84E-06 | 3.73E-02 | 1.59E-01 | 1.00E+00 | 1.00E+00 | rs701008 | 6.76E-05 | √ |
| PBC | *MICA* | 59 | 2.23E-07 | 5.14E-05 | 1.00E+00 | 1.00E+00 | 1.00E+00 | rs3117572 | 1.71E-16 |  |
| PBC | *MIEN1* | 59 | 2.64E-04 | 6.88E-03 | 3.82E-02 | 1.93E-02 | 2.90E-02 | rs7224568 | 4.10E-03 | √ |
| PBC | *MSH5* | 59 | 5.15E-03 | 1.00E+00 | 1.00E+00 | 9.25E-07 | 1.32E-06 | rs3117572 | 1.71E-16 |  |
| PBC | *MSH5-SAPCD1* | 59 | 1.69E-03 | 1.00E+00 | 1.00E+00 | 7.42E-07 | 1.07E-06 | rs3117572 | 1.71E-16 |  |
| PBC | *MUC22* | 245 | 4.98E-01 | 4.09E-18 | 1.95E-02 | 4.20E-03 | 6.30E-03 | rs2233976 | 2.37E-14 |  |
| PBC | *MYBPC2* | 71 | 8.62E-01 | 1.00E+00 | 1.00E+00 | 1.08E-08 | 1.61E-08 | rs2459144 | 2.15E-04 | √ |
| PBC | *MYO1A* | 71 | 1.53E-08 | 1.03E-05 | 1.00E+00 | 1.00E+00 | 1.00E+00 | rs11172113 | 3.60E-05 | √ |
| PBC | *NAAA* | 71 | 5.55E-09 | 1.00E+00 | 1.00E+00 | 1.00E+00 | 1.00E+00 | rs7673766 | 4.20E-03 | √ |
| PBC | *NAPSA* | 71 | 5.01E-01 | 1.00E+00 | 1.00E+00 | 3.62E-09 | 5.43E-09 | rs3745516 | 1.22E-20 |  |
| PBC | *NEU1* | 71 | 5.46E-09 | 8.03E-06 | 1.00E+00 | 9.59E-07 | 1.49E-06 | rs570963 | 3.17E-22 |  |
| PBC | *NFKB1* | 71 | 8.93E-01 | 2.15E-03 | 5.80E-01 | 1.00E+00 | 1.00E+00 | rs7676765 | 9.82E-07 | √ |
| PBC | *NISCH* | 71 | 2.72E-01 | 1.62E-09 | 1.00E+00 | 1.00E+00 | 1.00E+00 | rs4475032 | 1.16E-02 | √ |
| PBC | *NOTCH4* | 90 | 4.18E-21 | 9.13E-03 | 1.00E+00 | 4.19E-05 | 6.29E-05 | rs7774434 | 2.37E-56 |  |
| PBC | *NR1H2* | 90 | 8.88E-01 | 1.00E+00 | 1.00E+00 | 4.48E-09 | 6.71E-09 | rs3745516 | 1.22E-20 |  |
| PBC | *NSF* | 90 | 1.44E-07 | 1.00E+00 | 1.00E+00 | 1.00E+00 | 1.00E+00 | rs9906813 | 4.68E-03 | √ |
| PBC | *NT5DC2* | 90 | 9.40E-01 | 2.48E-04 | 1.00E+00 | 1.00E+00 | 1.00E+00 | rs4475032 | 1.16E-02 | √ |
| PBC | *ORMDL3* | 262 | 1.40E-09 | 1.56E-02 | 1.00E+00 | 1.69E-01 | 2.53E-01 | rs7224568 | 4.10E-03 | √ |
| PBC | *OS9* | 8 | 3.81E-05 | 2.31E-02 | 1.00E+00 | 1.00E+00 | 1.00E+00 | rs701008 | 6.76E-05 | √ |
| PBC | *PBX2* | 8 | 2.05E-20 | 2.20E-03 | 1.00E+00 | 3.21E-05 | 4.83E-05 | rs28421666 | 8.67E-47 |  |
| PBC | *PFDN6* | 8 | 4.75E-09 | 1.13E-18 | 2.37E-15 | 1.34E-19 | 2.01E-19 | rs1431402 | 6.21E-39 |  |
| PBC | *PGAP3* | 8 | 8.83E-06 | 3.80E-03 | 6.14E-02 | 2.45E-02 | 3.67E-02 | rs7224568 | 4.10E-03 | √ |
| PBC | *PGBD1* | 45 | 1.69E-02 | 1.83E-01 | 2.48E-02 | 1.00E+00 | 1.00E+00 | rs201004 | 1.02E-07 | √ |
| PBC | *PLA1A* | 45 | 4.45E-02 | 1.00E+00 | 1.00E+00 | 2.96E-03 | 4.43E-03 | rs2293370 | 4.26E-15 |  |
| PBC | *PLCB3* | 131 | 6.63E-02 | 6.93E-05 | 6.62E-03 | 1.00E+00 | 1.00E+00 | rs510372 | 1.68E-09 |  |
| PBC | *POGLUT1* | 131 | 1.99E-06 | 1.00E+00 | 1.00E+00 | 7.24E-03 | 1.09E-02 | rs2293370 | 4.26E-15 |  |
| PBC | *POLD1* | 131 | 2.42E-01 | 1.00E+00 | 1.00E+00 | 5.10E-09 | 7.65E-09 | rs3745516 | 1.22E-20 |  |
| PBC | *POM121L2* | 23 | 1.94E-05 | 5.40E-03 | 9.69E-05 | 1.00E+00 | 1.00E+00 | rs12200985 | 1.41E-07 | √ |
| PBC | *POU5F1* | 66 | 6.64E-01 | 1.00E+00 | 1.00E+00 | 2.22E-03 | 3.33E-03 | rs3763295 | 2.74E-15 |  |
| PBC | *PPT2* | 109 | 1.10E-19 | 2.81E-01 | 1.00E+00 | 2.79E-05 | 4.19E-05 | rs28421666 | 8.67E-47 |  |
| PBC | *PRRT1* | 109 | 8.51E-17 | 1.00E+00 | 1.00E+00 | 4.70E-05 | 7.06E-05 | rs9469099 | 1.35E-32 |  |
| PBC | *PRSS16* | 109 | 9.13E-08 | 7.94E-05 | 1.19E-04 | 1.00E+00 | 1.00E+00 | rs12200985 | 1.41E-07 | √ |
| PBC | *PSENEN* | 109 | 4.99E-07 | 1.00E+00 | 1.00E+00 | 1.00E+00 | 1.00E+00 | rs8108848 | 8.69E-05 | √ |
| PBC | *PSMB8* | 109 | 9.38E-03 | 1.24E-05 | 2.08E-05 | 2.29E-06 | 3.36E-06 | rs7774434 | 2.37E-56 |  |
| PBC | *PSMB9* | 109 | 1.48E-03 | 8.43E-06 | 1.38E-05 | 1.31E-06 | 1.91E-06 | rs7774434 | 2.37E-56 |  |
| PBC | *PSMD3* | 109 | 7.86E-03 | 5.52E-12 | 2.38E-01 | 4.21E-02 | 6.32E-02 | rs7224568 | 4.10E-03 | √ |
| PBC | *PSORS1C1* | 109 | 9.06E-01 | 1.30E-08 | 1.58E-01 | 1.17E-03 | 1.76E-03 | rs2233976 | 2.37E-14 |  |
| PBC | *RAC3* | 109 | 7.94E-01 | 2.22E-09 | 1.00E+00 | 1.00E+00 | 1.00E+00 | rs7216806 | 1.25E-02 | √ |
| PBC | *RAVER1* | 109 | 3.39E-08 | 1.00E+00 | 1.00E+00 | 1.00E+00 | 1.00E+00 | rs2304256 | 1.05E-10 |  |
| PBC | *RDBP* | 490 | 1.23E-03 | 1.00E+00 | 1.00E+00 | 4.28E-05 | 6.44E-05 | rs9469099 | 1.35E-32 |  |
| PBC | *RFNG* | 20 | 5.85E-01 | 1.60E-02 | 1.00E+00 | 1.00E+00 | 1.00E+00 | rs7216806 | 1.25E-02 | √ |
| PBC | *RGL2* | 20 | 1.54E-10 | 7.32E-24 | 7.04E-23 | 9.58E-20 | 1.44E-19 | rs1431402 | 6.21E-39 |  |
| PBC | *RING1* | 20 | 5.10E-16 | 1.00E+00 | 1.00E+00 | 8.68E-18 | 1.30E-17 | rs7774434 | 2.37E-56 |  |
| PBC | *RMI2* | 109 | 2.16E-07 | 3.68E-04 | 5.51E-03 | 1.00E+00 | 1.00E+00 | rs12924729 | 2.39E-14 |  |
| PBC | *RNF5* | 131 | 4.28E-17 | 1.72E-02 | 1.00E+00 | 2.83E-05 | 4.25E-05 | rs28421666 | 8.67E-47 |  |
| PBC | *RP11-347C12.1* | 70 | 2.34E-04 | 1.33E-02 | 1.00E+00 | 1.00E+00 | 1.00E+00 | rs4465620 | 1.67E-05 | √ |
| PBC | *RP11-347C12.3* | 328 | 5.65E-02 | 4.72E-02 | 1.00E+00 | 1.00E+00 | 1.00E+00 | rs4465620 | 1.67E-05 | √ |
| PBC | *RP11-509E16.1* | 328 | 3.83E-01 | 1.38E-02 | 1.55E-02 | 1.00E+00 | 1.00E+00 | rs7575363 | 9.01E-05 | √ |
| PBC | *RP11-571M6.15* | 254 | 7.12E-06 | 1.75E-02 | 7.85E-02 | 1.00E+00 | 1.00E+00 | rs701008 | 6.76E-05 | √ |
| PBC | *RP11-571M6.6* | 251 | 2.90E-05 | 6.91E-03 | 6.32E-02 | 1.00E+00 | 1.00E+00 | rs701008 | 6.76E-05 | √ |
| PBC | *RP11-736N17.2* | 7 | 6.99E-01 | 1.00E+00 | 1.00E+00 | 6.62E-09 | 9.92E-09 | rs8005065 | 1.46E-02 | √ |
| PBC | *RPS18* | 7 | 4.74E-11 | 1.00E+00 | 1.00E+00 | 1.04E-17 | 1.56E-17 | rs1431402 | 6.21E-39 |  |
| PBC | *RPS6KA4* | 8 | 1.89E-01 | 4.50E-04 | 1.00E+00 | 1.00E+00 | 1.00E+00 | rs510372 | 1.68E-09 |  |
| PBC | *RXRB* | 8 | 4.27E-18 | 2.08E-13 | 3.29E-13 | 1.81E-19 | 2.71E-19 | rs7774434 | 2.37E-56 |  |
| PBC | *SAPCD1* | 8 | 4.76E-05 | 3.72E-01 | 1.00E+00 | 4.10E-07 | 5.83E-07 | rs3117572 | 1.71E-16 |  |
| PBC | *SCHIP1* | 8 | 1.49E-03 | 9.37E-11 | 1.48E-04 | 1.46E-05 | 2.53E-05 | rs485499 | 1.43E-23 |  |
| PBC | *SERBP1* | 8 | 9.75E-07 | 9.68E-08 | 1.39E-06 | 1.09E-15 | 1.63E-15 | rs1954118 | 2.12E-16 |  |
| PBC | *SH2B3* | 31 | 7.41E-05 | 4.16E-02 | 1.74E-01 | 1.00E+00 | 1.00E+00 | rs11065987 | 3.20E-08 |  |
| PBC | *SKIV2L* | 31 | 3.30E-02 | 1.00E+00 | 1.00E+00 | 6.77E-04 | 1.02E-03 | rs9469099 | 1.35E-32 |  |
| PBC | *SLC39A7* | 31 | 9.02E-20 | 1.47E-10 | 6.15E-07 | 2.58E-19 | 3.88E-19 | rs7774434 | 2.37E-56 |  |
| PBC | *SLX1A* | 31 | 5.66E-03 | 2.11E-03 | 1.00E+00 | 1.00E+00 | 1.00E+00 | rs4465620 | 1.67E-05 | √ |
| PBC | *SMC4* | 31 | 8.26E-01 | 8.74E-03 | 9.67E-03 | 1.00E+00 | 1.00E+00 | rs485499 | 1.43E-23 |  |
| PBC | *SOCS1* | 31 | 1.37E-08 | 8.75E-08 | 5.90E-04 | 9.34E-02 | 1.40E-01 | rs12924729 | 2.39E-14 |  |
| PBC | *SPIB* | 415 | 3.88E-02 | 1.00E+00 | 1.00E+00 | 4.00E-07 | 5.96E-07 | rs3745516 | 1.22E-20 |  |
| PBC | *STAB1* | 415 | 9.65E-01 | 2.13E-02 | 1.00E+00 | 1.00E+00 | 1.00E+00 | rs4475032 | 1.16E-02 | √ |
| PBC | *STK19* | 415 | 9.75E-05 | 2.77E-33 | 1.53E-39 | 5.48E-05 | 8.22E-05 | rs9469099 | 1.35E-32 |  |
| PBC | *STK36* | 29 | 4.95E-03 | 2.91E-13 | 1.00E+00 | 1.00E+00 | 1.00E+00 | rs7557937 | 9.58E-05 | √ |
| PBC | *STRA13* | 29 | 2.58E-01 | 1.67E-06 | 1.00E+00 | 1.00E+00 | 1.00E+00 | rs7216806 | 1.25E-02 | √ |
| PBC | *TAP1* | 382 | 7.77E-02 | 9.41E-06 | 2.06E-05 | 5.65E-06 | 8.32E-06 | rs7774434 | 2.37E-56 |  |
| PBC | *TAP2* | 382 | 4.30E-04 | 1.62E-05 | 2.10E-05 | 3.74E-05 | 5.49E-05 | rs7774434 | 2.37E-56 |  |
| PBC | *TBC1D3G* | 95 | 5.29E-01 | 1.14E-14 | 1.00E+00 | 1.00E+00 | 1.00E+00 | rs9674621 | 2.61E-03 | √ |
| PBC | *TBC1D3H* | 95 | 9.64E-01 | 9.06E-144 | 1.00E+00 | 1.00E+00 | 1.00E+00 | rs9674621 | 2.61E-03 | √ |
| PBC | *TCAP* | 200 | 5.36E-07 | 8.44E-06 | 8.05E-07 | 9.14E-03 | 1.37E-02 | rs7224568 | 4.10E-03 | √ |
| PBC | *TCEB3C* | 200 | 3.87E-02 | 4.72E-108 | 1.00E+00 | 1.00E+00 | 1.00E+00 | rs9967367 | 9.60E-03 | √ |
| PBC | *TCEB3CL* | 200 | 4.48E-02 | 1.50E-146 | 1.00E+00 | 1.00E+00 | 1.00E+00 | rs9967367 | 9.60E-03 | √ |
| PBC | *TCF19* | 114 | 8.02E-01 | 1.00E+00 | 1.00E+00 | 1.03E-03 | 1.54E-03 | rs3763295 | 2.74E-15 |  |
| PBC | *TIMMDC1* | 243 | 1.16E-06 | 1.00E+00 | 1.00E+00 | 1.48E-03 | 2.22E-03 | rs2293370 | 4.26E-15 |  |
| PBC | *TMEM194A* | 243 | 1.05E-02 | 1.58E-03 | 1.00E+00 | 1.00E+00 | 1.00E+00 | rs11172113 | 3.60E-05 | √ |
| PBC | *TMEM39A* | 42 | 9.49E-07 | 1.00E+00 | 1.00E+00 | 2.64E-04 | 3.98E-04 | rs2293370 | 4.26E-15 |  |
| PBC | *TNFAIP2* | 42 | 6.31E-01 | 1.00E+00 | 1.00E+00 | 1.40E-07 | 2.11E-07 | rs2297067 | 6.34E-19 |  |
| PBC | *TNFSF15* | 42 | 7.86E-03 | 4.71E-02 | 9.64E-02 | 1.00E+00 | 1.00E+00 | rs7020333 | 5.49E-03 | √ |
| PBC | *TNPO3* | 42 | 1.07E-08 | 1.49E-08 | 2.30E-07 | 3.44E-11 | 5.16E-11 | rs10488631 | 5.08E-23 |  |
| PBC | *TNXB* | 42 | 5.94E-09 | 1.00E+00 | 1.00E+00 | 5.04E-05 | 7.56E-05 | rs9469099 | 1.35E-32 |  |
| PBC | *TRMT112* | 42 | 1.14E-01 | 5.21E-06 | 4.03E-02 | 1.00E+00 | 1.00E+00 | rs510372 | 1.68E-09 |  |
| PBC | *TSFM* | 42 | 2.02E-05 | 2.13E-02 | 1.31E-02 | 1.00E+00 | 1.00E+00 | rs701008 | 6.76E-05 | √ |
| PBC | *TSPAN31* | 42 | 2.12E-05 | 6.68E-03 | 1.00E+00 | 1.00E+00 | 1.00E+00 | rs701008 | 6.76E-05 | √ |
| PBC | *TTLL4* | 52 | 9.71E-03 | 3.22E-05 | 1.00E+00 | 1.00E+00 | 1.00E+00 | rs7557937 | 9.58E-05 | √ |
| PBC | *TYK2* | 188 | 3.72E-08 | 5.00E-01 | 1.00E+00 | 1.00E+00 | 1.00E+00 | rs2304256 | 1.05E-10 |  |
| PBC | *UBLCP1* | 219 | 1.53E-03 | 1.47E-02 | 1.30E-03 | 1.00E+00 | 1.00E+00 | rs17663721 | 2.27E-03 | √ |
| PBC | *UPK2* | 219 | 1.48E-01 | 1.00E+00 | 1.00E+00 | 3.15E-02 | 4.73E-02 | rs4938573 | 1.15E-13 |  |
| PBC | *VARS* | 75 | 3.48E-07 | 1.00E+00 | 1.00E+00 | 6.94E-07 | 9.93E-07 | rs3117572 | 1.71E-16 |  |
| PBC | *VPS52* | 65 | 8.80E-12 | 1.00E+00 | 1.00E+00 | 2.19E-17 | 3.28E-17 | rs1431402 | 6.21E-39 |  |
| PBC | *VWA7* | 65 | 1.05E-04 | 1.00E+00 | 1.00E+00 | 8.28E-07 | 1.19E-06 | rs3117572 | 1.71E-16 |  |
| PBC | *WDR46* | 66 | 1.42E-10 | 9.52E-09 | 1.00E+00 | 3.07E-19 | 4.61E-19 | rs1431402 | 6.21E-39 |  |
| PBC | *WNT3* | 66 | 9.09E-08 | 4.62E-03 | 1.00E+00 | 1.00E+00 | 1.00E+00 | rs10468514 | 2.06E-03 | √ |
| PBC | *XXbac-BPG181M17.5* | 46 | 2.89E-11 | 6.44E-08 | 6.43E-08 | 1.00E+00 | 1.00E+00 | rs7774434 | 2.37E-56 |  |
| PBC | *XXbac-BPG246D15.9* | 46 | 4.07E-03 | 1.20E-05 | 2.08E-05 | 7.72E-06 | 1.14E-05 | rs7774434 | 2.37E-56 |  |
| PBC | *XXbac-BPG300A18.12* | 46 | 9.26E-19 | 4.19E-01 | 1.00E+00 | 2.97E-05 | 4.46E-05 | rs28421666 | 8.67E-47 |  |
| PBC | *XXbac-BPG32J3.22* | 46 | 4.73E-01 | 2.32E-08 | 1.00E+00 | 2.02E-07 | 2.85E-07 | rs3117572 | 1.71E-16 |  |
| PBC | *YAF2* | 46 | 3.68E-04 | 4.46E-02 | 8.13E-01 | 1.00E+00 | 1.00E+00 | rs11832772 | 7.31E-07 | √ |
| PBC | *YPEL5* | 80 | 5.23E-02 | 1.33E-02 | 1.00E+00 | 1.00E+00 | 1.00E+00 | rs4952108 | 5.05E-08 | √ |
| PBC | *ZBTB12* | 80 | 9.37E-19 | 1.12E-30 | 1.41E-19 | 2.12E-07 | 3.35E-07 | rs9469099 | 1.35E-32 |  |
| PBC | *ZGLP1* | 80 | 2.17E-07 | 1.00E+00 | 1.00E+00 | 1.00E+00 | 1.00E+00 | rs2304256 | 1.05E-10 |  |
| PBC | *ZKSCAN3* | 80 | 7.61E-09 | 1.00E+00 | 1.00E+00 | 1.00E+00 | 1.00E+00 | rs201004 | 1.02E-07 | √ |
| PBC | *ZNF323* | 80 | 3.11E-07 | 1.00E+00 | 1.00E+00 | 1.00E+00 | 1.00E+00 | rs201004 | 1.02E-07 | √ |
| PBC | *ZNHIT3* | 630 | 6.47E-01 | 1.86E-08 | 1.00E+00 | 1.00E+00 | 1.00E+00 | rs9674621 | 2.61E-03 | √ |
| PBC | *ZPBP2* | 326 | 1.02E-09 | 4.55E-08 | 1.00E+00 | 6.91E-02 | 1.04E-01 | rs7224568 | 4.10E-03 | √ |
| PBC | *ZSCAN12* | 326 | 2.79E-07 | 1.00E+00 | 1.00E+00 | 1.00E+00 | 1.00E+00 | rs209181 | 1.91E-07 | √ |
| PBC | *ZSCAN23* | 83 | 1.66E-07 | 2.03E-02 | 7.98E-01 | 1.00E+00 | 1.00E+00 | rs209181 | 1.91E-07 | √ |
| PSC | *ABHD16A* | 83 | 6.95E-05 | 2.76E-131 | 2.66E-108 | 2.46E-203 | 3.69E-203 | rs3130612 | 8.23E-245 |  |
| PSC | *ABT1* | 83 | 4.65E-01 | 7.64E-110 | 1.00E+00 | 5.10E-49 | 7.66E-49 | rs66462181 | 9.20E-81 |  |
| PSC | *AGER* | 83 | 4.76E-02 | 3.75E-115 | 1.50E-15 | 5.23E-194 | 7.84E-194 | rs4713534 | 6.58E-232 |  |
| PSC | *AGPAT1* | 39 | 2.07E-01 | 7.69E-80 | 1.01E-04 | 4.20E-194 | 6.30E-194 | rs4713534 | 6.58E-232 |  |
| PSC | *AIF1* | 39 | 7.38E-01 | 4.51E-293 | 8.47E-176 | 2.14E-226 | 3.21E-226 | rs3130612 | 8.23E-245 |  |
| PSC | *AMIGO3* | 39 | 2.80E-30 | 4.83E-03 | 1.00E+00 | 1.03E-10 | 1.55E-10 | rs9858213 | 2.43E-20 |  |
| PSC | *AMT* | 39 | 3.58E-01 | 4.22E-19 | 8.55E-01 | 3.32E-07 | 5.00E-07 | rs9858213 | 2.43E-20 |  |
| PSC | *APEH* | 39 | 4.13E-32 | 4.70E-14 | 1.00E+00 | 5.01E-11 | 7.52E-11 | rs9858213 | 2.43E-20 |  |
| PSC | *APOM* | 200 | 3.04E-03 | 9.19E-161 | 2.62E-78 | 1.41E-225 | 2.11E-225 | rs3130612 | 8.23E-245 |  |
| PSC | *ARIH2* | 200 | 1.25E-02 | 1.00E+00 | 1.23E-03 | 1.00E+00 | 1.00E+00 | rs7630869 | 3.38E-17 |  |
| PSC | *ARL6IP1* | 200 | 4.73E-01 | 1.22E-04 | 1.00E+00 | 1.00E+00 | 1.00E+00 | rs2239976 | 1.39E-02 | √ |
| PSC | *ATF6B* | 200 | 3.71E-03 | 8.89E-136 | 2.56E-26 | 3.66E-203 | 5.50E-203 | rs4713534 | 6.58E-232 |  |
| PSC | *ATP6V1G2* | 137 | 4.41E-07 | 0.00E+00 | 4.71E-23 | 4.23E-226 | 6.35E-226 | rs3130612 | 8.23E-245 |  |
| PSC | *ATP6V1G2-DDX39B* | 165 | 2.23E-03 | 0.00E+00 | 1.12E-26 | 6.95E-226 | 1.04E-225 | rs3130612 | 8.23E-245 |  |
| PSC | *ATRNL1* | 614 | 8.81E-01 | 4.01E-05 | 1.00E+00 | 1.00E+00 | 1.00E+00 | rs11197272 | 2.64E-04 | √ |
| PSC | *B3GALT4* | 231 | 1.99E-03 | 1.00E+00 | 1.00E+00 | 4.86E-23 | 7.29E-23 | rs4947349 | 4.57E-171 |  |
| PSC | *BACH2* | 363 | 5.77E-01 | 1.71E-03 | 1.00E+00 | 1.00E+00 | 1.00E+00 | rs7750271 | 3.06E-07 | √ |
| PSC | *BAG6* | 185 | 4.43E-05 | 6.14E-291 | 2.78E-99 | 4.63E-226 | 6.94E-226 | rs3130612 | 8.23E-245 |  |
| PSC | *BAK1* | 36 | 4.61E-01 | 1.00E+00 | 1.00E+00 | 1.65E-05 | 3.07E-05 | rs115991849 | 2.53E-41 |  |
| PSC | *BBS12* | 117 | 5.56E-01 | 9.55E-01 | 7.50E-04 | 1.00E+00 | 1.00E+00 | rs13119723 | 2.22E-10 |  |
| PSC | *BRD2* | 127 | 5.19E-03 | 1.00E+00 | 1.00E+00 | 7.20E-70 | 1.08E-69 | rs4713534 | 6.58E-232 |  |
| PSC | *BRWD1* | 127 | 4.00E-01 | 1.00E+00 | 8.03E-01 | 1.45E-05 | 2.20E-05 | rs4817988 | 4.20E-15 |  |
| PSC | *BSN* | 110 | 4.38E-26 | 1.34E-24 | 7.80E-01 | 6.29E-11 | 9.44E-11 | rs9858213 | 2.43E-20 |  |
| PSC | *BTN1A1* | 127 | 8.79E-01 | 4.11E-17 | 1.00E+00 | 1.87E-59 | 2.81E-59 | rs45527431 | 1.12E-80 |  |
| PSC | *BTN2A1* | 205 | 1.36E-02 | 1.82E-08 | 1.00E+00 | 5.66E-59 | 8.49E-59 | rs45527431 | 1.12E-80 |  |
| PSC | *BTN2A2* | 205 | 9.88E-03 | 1.31E-10 | 1.00E+00 | 6.77E-60 | 1.02E-59 | rs45527431 | 1.12E-80 |  |
| PSC | *BTN3A1* | 322 | 6.75E-02 | 4.72E-16 | 9.34E-03 | 7.38E-60 | 1.11E-59 | rs45527431 | 1.12E-80 |  |
| PSC | *BTN3A2* | 322 | 5.76E-03 | 2.34E-08 | 1.00E+00 | 1.96E-59 | 2.94E-59 | rs45527431 | 1.12E-80 |  |
| PSC | *BTN3A3* | 322 | 6.48E-03 | 6.75E-08 | 1.00E+00 | 8.81E-60 | 1.32E-59 | rs45527431 | 1.12E-80 |  |
| PSC | *BTNL2* | 72 | 9.45E-06 | 6.08E-04 | 1.00E+00 | 6.62E-196 | 9.93E-196 | rs4713534 | 6.58E-232 |  |
| PSC | *C10orf28* | 29 | 2.57E-01 | 1.28E-02 | 1.00E+00 | 1.00E+00 | 1.00E+00 | rs117400681 | 1.44E-03 | √ |
| PSC | *C11orf1* | 169 | 1.40E-03 | 8.15E-07 | 1.00E+00 | 1.00E+00 | 1.00E+00 | rs12363179 | 1.29E-04 | √ |
| PSC | *C15orf33* | 94 | 9.33E-01 | 2.43E-09 | 1.00E+00 | 1.00E+00 | 1.00E+00 | rs146484572 | 2.17E-03 | √ |
| PSC | *C1orf43* | 48 | 6.86E-01 | 7.05E-17 | 1.00E+00 | 1.00E+00 | 1.00E+00 | rs4446955 | 5.33E-05 | √ |
| PSC | *C2* | 117 | 7.68E-01 | 4.22E-98 | 2.96E-11 | 5.98E-197 | 8.97E-197 | rs3130612 | 8.23E-245 |  |
| PSC | *C3orf62* | 116 | 5.43E-03 | 1.00E+00 | 1.33E-04 | 2.90E-06 | 4.35E-06 | rs9858213 | 2.43E-20 |  |
| PSC | *C3orf71* | 22 | 1.94E-03 | 1.00E+00 | 7.93E-04 | 1.00E+00 | 1.00E+00 | rs148734725 | 4.98E-17 |  |
| PSC | *C4A* | 22 | 1.90E-01 | 8.25E-72 | 1.35E-11 | 7.68E-202 | 1.15E-201 | rs3130612 | 8.23E-245 |  |
| PSC | *C4B* | 22 | 7.75E-03 | 7.56E-100 | 2.54E-14 | 1.87E-201 | 2.81E-201 | rs9267488 | 2.31E-236 |  |
| PSC | *C6orf47* | 284 | 2.54E-10 | 3.31E-147 | 3.38E-37 | 2.08E-225 | 3.12E-225 | rs3130612 | 8.23E-245 |  |
| PSC | *C6orf48* | 81 | 8.65E-29 | 2.17E-125 | 1.03E-08 | 5.91E-204 | 8.86E-204 | rs3130612 | 8.23E-245 |  |
| PSC | *CASP7* | 81 | 8.04E-01 | 4.40E-08 | 1.00E+00 | 1.00E+00 | 1.00E+00 | rs932653 | 2.02E-05 | √ |
| PSC | *CCDC71* | 81 | 2.73E-01 | 1.00E+00 | 1.86E-03 | 1.00E+00 | 1.00E+00 | rs34762726 | 2.59E-20 |  |
| PSC | *CCHCR1* | 81 | 8.27E-01 | 3.90E-188 | 2.91E-05 | 7.53E-213 | 1.13E-212 | rs3130612 | 8.23E-245 |  |
| PSC | *CFB* | 81 | 2.70E-01 | 1.68E-134 | 2.33E-15 | 3.93E-201 | 5.89E-201 | rs3130612 | 8.23E-245 |  |
| PSC | *CLIC1* | 81 | 5.65E-48 | 3.65E-53 | 2.40E-03 | 2.01E-204 | 3.02E-204 | rs3130612 | 8.23E-245 |  |
| PSC | *CMAHP* | 81 | 9.08E-05 | 2.47E-09 | 1.07E-06 | 2.97E-08 | 5.00E-08 | rs68006638 | 4.49E-64 |  |
| PSC | *COL11A2* | 81 | 6.37E-01 | 1.00E+00 | 1.00E+00 | 9.24E-29 | 1.39E-28 | rs9276348 | 9.17E-199 |  |
| PSC | *CRIPAK* | 138 | 4.25E-02 | 2.63E-07 | 1.00E+00 | 1.00E+00 | 1.00E+00 | rs111631632 | 9.63E-04 | √ |
| PSC | *CRYAB* | 63 | 1.78E-06 | 1.00E+00 | 1.00E+00 | 1.00E+00 | 1.00E+00 | rs12363179 | 1.29E-04 | √ |
| PSC | *CSNK2B* | 63 | 6.29E-07 | 6.71E-148 | 1.56E-67 | 5.58E-225 | 8.37E-225 | rs3130612 | 8.23E-245 |  |
| PSC | *CTA-14H9.5* | 63 | 1.65E-01 | 3.00E-19 | 1.00E+00 | 2.31E-58 | 3.47E-58 | rs45527431 | 1.12E-80 |  |
| PSC | *CUTA* | 408 | 4.89E-01 | 1.00E+00 | 1.00E+00 | 5.56E-18 | 8.34E-18 | rs116747494 | 3.25E-63 |  |
| PSC | *CYP21A2* | 408 | 7.24E-03 | 4.03E-122 | 3.02E-16 | 5.09E-202 | 7.64E-202 | rs9267488 | 2.31E-236 |  |
| PSC | *DAG1* | 408 | 1.70E-07 | 8.76E-25 | 6.19E-01 | 1.90E-08 | 2.85E-08 | rs9858213 | 2.43E-20 |  |
| PSC | *DALRD3* | 471 | 1.01E-01 | 1.00E+00 | 8.56E-03 | 1.00E+00 | 1.00E+00 | rs13062429 | 3.27E-17 |  |
| PSC | *DAXX* | 108 | 1.95E-03 | 1.00E+00 | 1.00E+00 | 6.77E-19 | 1.02E-18 | rs60045856 | 2.92E-97 |  |
| PSC | *DDAH2* | 95 | 2.35E-57 | 7.10E-65 | 4.43E-06 | 2.41E-204 | 3.62E-204 | rs3130612 | 8.23E-245 |  |
| PSC | *DDR1* | 71 | 6.89E-03 | 1.00E+00 | 3.71E-06 | 2.74E-02 | 4.11E-02 | rs2596500 | 7.62E-244 |  |
| PSC | *DDX39B* | 42 | 1.25E-07 | 0.00E+00 | 5.98E-120 | 1.12E-226 | 1.68E-226 | rs3130612 | 8.23E-245 |  |
| PSC | *DHODH* | 177 | 6.59E-01 | 1.37E-02 | 1.00E+00 | 1.00E+00 | 1.00E+00 | rs12597418 | 1.55E-03 | √ |
| PSC | *DOM3Z* | 240 | 7.72E-02 | 1.83E-209 | 7.49E-36 | 2.45E-201 | 3.68E-201 | rs3130612 | 8.23E-245 |  |
| PSC | *EGFL8* | 240 | 5.13E-02 | 4.37E-112 | 1.31E-25 | 2.79E-195 | 4.19E-195 | rs4713534 | 6.58E-232 |  |
| PSC | *EHMT2* | 240 | 4.84E-01 | 7.94E-106 | 1.39E-10 | 2.35E-197 | 3.52E-197 | rs3130612 | 8.23E-245 |  |
| PSC | *FAM212A* | 240 | 1.12E-09 | 1.00E+00 | 1.00E+00 | 1.00E+00 | 1.00E+00 | rs9858213 | 2.43E-20 |  |
| PSC | *FAM213B* | 240 | 2.22E-06 | 1.00E+00 | 1.00E+00 | 1.00E+00 | 1.00E+00 | rs6657596 | 2.70E-08 |  |
| PSC | *FDXACB1* | 240 | 1.74E-03 | 7.23E-12 | 1.00E+00 | 1.00E+00 | 1.00E+00 | rs12363179 | 1.29E-04 | √ |
| PSC | *FGF7* | 240 | 8.90E-01 | 2.15E-10 | 1.00E+00 | 1.00E+00 | 1.00E+00 | rs8025237 | 2.63E-03 | √ |
| PSC | *FKBPL* | 240 | 1.50E-02 | 2.93E-74 | 1.90E-14 | 5.05E-202 | 7.58E-202 | rs4713534 | 6.58E-232 |  |
| PSC | *GABBR1* | 101 | 1.15E-03 | 1.98E-45 | 7.08E-09 | 2.90E-48 | 4.35E-48 | rs2523978 | 9.69E-149 |  |
| PSC | *GBAS* | 24 | 1.13E-01 | 3.76E-04 | 1.00E+00 | 1.00E+00 | 1.00E+00 | rs816416 | 5.95E-05 | √ |
| PSC | *GLIPR1* | 79 | 7.96E-01 | 6.72E-20 | 1.00E+00 | 1.00E+00 | 1.00E+00 | rs7966763 | 1.21E-04 | √ |
| PSC | *GLIPR1L2* | 95 | 1.35E-01 | 5.43E-06 | 1.00E+00 | 1.00E+00 | 1.00E+00 | rs7966763 | 1.21E-04 | √ |
| PSC | *GMPPB* | 126 | 3.10E-26 | 3.67E-04 | 1.00E+00 | 3.20E-10 | 4.79E-10 | rs9858213 | 2.43E-20 |  |
| PSC | *GPANK1* | 126 | 1.32E-11 | 3.03E-287 | 7.28E-156 | 1.58E-224 | 2.36E-224 | rs3130612 | 8.23E-245 |  |
| PSC | *GPSM3* | 143 | 6.90E-04 | 4.72E-112 | 2.96E-25 | 1.66E-194 | 2.50E-194 | rs4713534 | 6.58E-232 |  |
| PSC | *GPX1* | 57 | 2.01E-01 | 4.53E-11 | 3.91E-05 | 5.86E-07 | 8.87E-07 | rs9858213 | 2.43E-20 |  |
| PSC | *GTF2H4* | 394 | 2.08E-02 | 1.00E+00 | 3.72E-06 | 4.03E-02 | 6.05E-02 | rs2596500 | 7.62E-244 |  |
| PSC | *HCG11* | 394 | 1.50E-01 | 6.01E-47 | 1.00E+00 | 5.31E-59 | 7.97E-59 | rs45527431 | 1.12E-80 |  |
| PSC | *HCG22* | 394 | 3.25E-03 | 0.00E+00 | 2.84E-31 | 2.45E-212 | 3.68E-212 | rs3130612 | 8.23E-245 |  |
| PSC | *HCG27* | 394 | 1.32E-01 | 1.28E-86 | 3.03E-93 | 7.82E-211 | 1.17E-210 | rs3130612 | 8.23E-245 |  |
| PSC | *HES5* | 394 | 1.32E-06 | 1.00E+00 | 1.00E+00 | 1.00E+00 | 1.00E+00 | rs6657596 | 2.70E-08 |  |
| PSC | *HFE* | 394 | 2.28E-08 | 2.86E-15 | 2.10E-02 | 1.55E-52 | 2.32E-52 | rs35400317 | 3.94E-80 |  |
| PSC | *HIST1H1B* | 394 | 7.75E-65 | 1.18E-69 | 1.00E+00 | 3.68E-80 | 5.52E-80 | rs13197176 | 4.49E-100 |  |
| PSC | *HIST1H1C* | 92 | 1.57E-15 | 3.08E-17 | 1.14E-01 | 1.60E-53 | 2.40E-53 | rs13195402 | 1.46E-79 |  |
| PSC | *HIST1H1D* | 92 | 1.00E-04 | 2.62E-01 | 1.00E+00 | 5.49E-58 | 8.23E-58 | rs45527431 | 1.12E-80 |  |
| PSC | *HIST1H1E* | 92 | 3.26E-02 | 1.00E+00 | 1.00E+00 | 2.04E-53 | 3.06E-53 | rs45527431 | 1.12E-80 |  |
| PSC | *HIST1H2AB* | 92 | 8.00E-14 | 2.73E-19 | 5.23E-01 | 7.49E-53 | 1.12E-52 | rs13195402 | 1.46E-79 |  |
| PSC | *HIST1H2AC* | 92 | 5.56E-03 | 3.83E-03 | 1.00E+00 | 3.49E-52 | 5.23E-52 | rs45527431 | 1.12E-80 |  |
| PSC | *HIST1H2AE* | 92 | 3.26E-03 | 6.78E-01 | 1.00E+00 | 1.65E-56 | 2.47E-56 | rs45527431 | 1.12E-80 |  |
| PSC | *HIST1H2AG* | 92 | 1.26E-05 | 1.38E-15 | 2.41E-14 | 2.82E-69 | 4.23E-69 | rs34196306 | 6.12E-87 |  |
| PSC | *HIST1H2AH* | 92 | 1.11E-03 | 3.57E-07 | 1.00E+00 | 3.13E-69 | 4.70E-69 | rs34196306 | 6.12E-87 |  |
| PSC | *HIST1H2AI* | 233 | 2.93E-67 | 1.69E-37 | 1.28E-26 | 2.15E-81 | 3.22E-81 | rs13197176 | 4.49E-100 |  |
| PSC | *HIST1H2AJ* | 233 | 7.05E-66 | 2.97E-38 | 6.11E-29 | 3.01E-81 | 4.52E-81 | rs13197176 | 4.49E-100 |  |
| PSC | *HIST1H2AK* | 233 | 4.61E-70 | 3.08E-60 | 1.00E+00 | 7.44E-76 | 1.12E-75 | rs13197176 | 4.49E-100 |  |
| PSC | *HIST1H2AL* | 170 | 3.68E-68 | 1.12E-70 | 1.00E+00 | 8.39E-78 | 1.26E-77 | rs13197176 | 4.49E-100 |  |
| PSC | *HIST1H2AM* | 14 | 5.02E-67 | 1.17E-73 | 1.00E+00 | 1.92E-80 | 2.88E-80 | rs35017208 | 3.88E-101 |  |
| PSC | *HIST1H2BB* | 61 | 3.88E-11 | 2.29E-21 | 9.32E-33 | 5.50E-54 | 8.25E-54 | rs13195402 | 1.46E-79 |  |
| PSC | *HIST1H2BC* | 61 | 1.85E-03 | 6.44E-05 | 1.27E-01 | 1.42E-53 | 2.13E-53 | rs45527431 | 1.12E-80 |  |
| PSC | *HIST1H2BD* | 61 | 5.76E-03 | 1.00E+00 | 1.00E+00 | 2.94E-57 | 4.41E-57 | rs45527431 | 1.12E-80 |  |
| PSC | *HIST1H2BE* | 61 | 1.29E-02 | 1.00E+00 | 1.00E+00 | 6.35E-57 | 9.52E-57 | rs45527431 | 1.12E-80 |  |
| PSC | *HIST1H2BF* | 61 | 7.01E-02 | 1.00E+00 | 1.00E+00 | 5.30E-57 | 7.95E-57 | rs45527431 | 1.12E-80 |  |
| PSC | *HIST1H2BG* | 61 | 8.33E-03 | 1.00E+00 | 1.00E+00 | 6.32E-57 | 9.48E-57 | rs45527431 | 1.12E-80 |  |
| PSC | *HIST1H2BH* | 61 | 5.82E-03 | 1.00E+00 | 3.23E-01 | 3.34E-58 | 5.01E-58 | rs45527431 | 1.12E-80 |  |
| PSC | *HIST1H2BI* | 61 | 2.82E-01 | 1.00E+00 | 8.84E-01 | 1.24E-57 | 1.86E-57 | rs45527431 | 1.12E-80 |  |
| PSC | *HIST1H2BJ* | 87 | 1.77E-07 | 9.31E-12 | 1.00E+00 | 7.05E-69 | 1.06E-68 | rs34196306 | 6.12E-87 |  |
| PSC | *HIST1H2BK* | 87 | 1.58E-03 | 6.78E-11 | 1.00E+00 | 9.60E-70 | 1.44E-69 | rs34196306 | 6.12E-87 |  |
| PSC | *HIST1H2BL* | 87 | 2.59E-64 | 5.77E-31 | 7.95E-25 | 2.54E-79 | 3.81E-79 | rs13197176 | 4.49E-100 |  |
| PSC | *HIST1H2BM* | 87 | 1.18E-65 | 5.25E-44 | 5.13E-36 | 6.46E-80 | 9.69E-80 | rs13197176 | 4.49E-100 |  |
| PSC | *HIST1H2BN* | 87 | 7.20E-71 | 7.11E-51 | 1.00E+00 | 1.53E-76 | 2.29E-76 | rs13197176 | 4.49E-100 |  |
| PSC | *HIST1H2BO* | 87 | 6.60E-67 | 1.97E-76 | 1.00E+00 | 2.83E-80 | 4.25E-80 | rs35017208 | 3.88E-101 |  |
| PSC | *HIST1H3A* | 87 | 2.63E-21 | 4.95E-44 | 1.00E+00 | 4.99E-54 | 7.49E-54 | rs13195402 | 1.46E-79 |  |
| PSC | *HIST1H3B* | 87 | 1.68E-13 | 2.42E-19 | 4.59E-10 | 5.38E-54 | 8.07E-54 | rs13195402 | 1.46E-79 |  |
| PSC | *HIST1H3C* | 43 | 5.19E-10 | 2.06E-08 | 1.00E+00 | 6.33E-53 | 9.49E-53 | rs13195402 | 1.46E-79 |  |
| PSC | *HIST1H3D* | 112 | 2.53E-02 | 2.83E-01 | 1.00E+00 | 3.61E-56 | 5.42E-56 | rs45527431 | 1.12E-80 |  |
| PSC | *HIST1H3E* | 112 | 1.39E-02 | 1.00E+00 | 1.00E+00 | 1.00E-57 | 1.50E-57 | rs45527431 | 1.12E-80 |  |
| PSC | *HIST1H3F* | 112 | 2.76E-03 | 1.00E+00 | 1.00E+00 | 1.82E-58 | 2.72E-58 | rs45527431 | 1.12E-80 |  |
| PSC | *HIST1H3G* | 112 | 7.45E-02 | 1.00E+00 | 1.00E+00 | 2.40E-58 | 3.59E-58 | rs45527431 | 1.12E-80 |  |
| PSC | *HIST1H3H* | 112 | 1.39E-65 | 1.41E-45 | 1.63E-50 | 3.69E-79 | 5.54E-79 | rs13197176 | 4.49E-100 |  |
| PSC | *HIST1H3I* | 112 | 5.61E-70 | 3.15E-73 | 1.00E+00 | 2.06E-80 | 3.08E-80 | rs13197176 | 4.49E-100 |  |
| PSC | *HIST1H3J* | 87 | 1.14E-67 | 2.42E-64 | 1.00E+00 | 5.35E-79 | 8.03E-79 | rs35017208 | 3.88E-101 |  |
| PSC | *HIST1H4A* | 85 | 2.15E-14 | 9.79E-42 | 1.00E+00 | 8.19E-53 | 1.23E-52 | rs13195402 | 1.46E-79 |  |
| PSC | *HIST1H4B* | 85 | 1.24E-13 | 1.93E-15 | 1.00E+00 | 3.23E-53 | 4.85E-53 | rs13195402 | 1.46E-79 |  |
| PSC | *HIST1H4C* | 85 | 6.56E-08 | 3.98E-16 | 2.02E-06 | 1.92E-53 | 2.88E-53 | rs45527431 | 1.12E-80 |  |
| PSC | *HIST1H4D* | 41 | 1.24E-02 | 1.00E+00 | 1.00E+00 | 1.85E-57 | 2.77E-57 | rs45527431 | 1.12E-80 |  |
| PSC | *HIST1H4E* | 61 | 1.51E-01 | 1.00E+00 | 1.00E+00 | 5.34E-56 | 8.01E-56 | rs45527431 | 1.12E-80 |  |
| PSC | *HIST1H4H* | 61 | 1.37E-01 | 1.00E+00 | 1.00E+00 | 1.88E-57 | 2.81E-57 | rs45527431 | 1.12E-80 |  |
| PSC | *HIST1H4I* | 61 | 3.01E-04 | 1.88E-06 | 1.00E+00 | 1.12E-69 | 1.67E-69 | rs34196306 | 6.12E-87 |  |
| PSC | *HIST1H4J* | 61 | 2.05E-71 | 3.85E-65 | 1.00E+00 | 7.97E-78 | 1.20E-77 | rs13197176 | 4.49E-100 |  |
| PSC | *HIST1H4K* | 61 | 1.74E-72 | 1.60E-63 | 1.00E+00 | 1.72E-78 | 2.58E-78 | rs13197176 | 4.49E-100 |  |
| PSC | *HLA-B* | 61 | 7.11E-02 | 1.09E-147 | 7.17E-46 | 4.46E-233 | 6.70E-233 | rs3130612 | 8.23E-245 |  |
| PSC | *HLA-C* | 61 | 5.19E-04 | 8.04E-111 | 1.40E-38 | 1.24E-233 | 1.86E-233 | rs3130612 | 8.23E-245 |  |
| PSC | *HLA-DMA* | 61 | 2.04E-02 | 1.00E+00 | 1.00E+00 | 2.97E-68 | 4.46E-68 | rs4713534 | 6.58E-232 |  |
| PSC | *HLA-DMB* | 195 | 6.81E-02 | 1.00E+00 | 1.00E+00 | 1.08E-76 | 1.62E-76 | rs4713534 | 6.58E-232 |  |
| PSC | *HLA-DOA* | 117 | 9.06E-03 | 1.00E+00 | 1.00E+00 | 2.57E-51 | 3.85E-51 | rs9276348 | 9.17E-199 |  |
| PSC | *HLA-DOB* | 25 | 3.33E-05 | 1.47E-37 | 1.38E-06 | 1.30E-81 | 1.94E-81 | rs4713534 | 6.58E-232 |  |
| PSC | *HLA-DPA1* | 91 | 1.30E-01 | 1.00E+00 | 1.00E+00 | 9.45E-36 | 1.42E-35 | rs9276348 | 9.17E-199 |  |
| PSC | *HLA-DPB1* | 91 | 4.09E-01 | 1.00E+00 | 1.00E+00 | 2.09E-38 | 3.14E-38 | rs9276348 | 9.17E-199 |  |
| PSC | *HLA-DQA1* | 91 | 6.54E-01 | 9.00E-22 | 9.06E-03 | 5.49E-79 | 8.24E-79 | rs4713534 | 6.58E-232 |  |
| PSC | *HLA-DQA2* | 310 | 2.51E-08 | 3.21E-25 | 2.39E-07 | 1.71E-81 | 2.56E-81 | rs4713534 | 6.58E-232 |  |
| PSC | *HLA-DQB1* | 22 | 2.42E-02 | 3.69E-07 | 1.29E-03 | 7.85E-79 | 1.18E-78 | rs4713534 | 6.58E-232 |  |
| PSC | *HLA-DQB2* | 22 | 1.44E-06 | 1.74E-42 | 1.13E-08 | 2.59E-83 | 3.88E-83 | rs4713534 | 6.58E-232 |  |
| PSC | *HLA-DRA* | 75 | 1.21E-08 | 1.00E+00 | 1.00E+00 | 1.19E-11 | 1.79E-11 | rs4713534 | 6.58E-232 |  |
| PSC | *HLA-DRB1* | 116 | 2.32E-01 | 4.08E-06 | 1.00E+00 | 3.37E-79 | 5.06E-79 | rs4713534 | 6.58E-232 |  |
| PSC | *HLA-DRB5* | 116 | 5.48E-02 | 6.18E-12 | 1.00E+00 | 1.90E-79 | 2.84E-79 | rs4713534 | 6.58E-232 |  |
| PSC | *HLA-F* | 116 | 1.02E-02 | 1.76E-64 | 3.37E-03 | 1.90E-46 | 2.85E-46 | rs2517617 | 5.01E-152 |  |
| PSC | *HLA-G* | 116 | 6.81E-01 | 4.87E-07 | 1.00E+00 | 4.90E-39 | 7.34E-39 | rs2517617 | 5.01E-152 |  |
| PSC | *HMG20A* | 116 | 2.37E-01 | 4.19E-04 | 1.00E+00 | 1.00E+00 | 1.00E+00 | rs77952682 | 7.42E-04 | √ |
| PSC | *HMGN4* | 116 | 6.27E-04 | 3.64E-82 | 1.00E+00 | 2.07E-58 | 3.11E-58 | rs45527431 | 1.12E-80 |  |
| PSC | *HSD17B8* | 86 | 3.38E-01 | 1.00E+00 | 1.00E+00 | 6.82E-27 | 1.02E-26 | rs9276348 | 9.17E-199 |  |
| PSC | *HSPA1A* | 86 | 5.51E-37 | 5.51E-95 | 1.41E-04 | 3.01E-204 | 4.51E-204 | rs3130612 | 8.23E-245 |  |
| PSC | *HSPA1B* | 86 | 2.40E-32 | 8.75E-109 | 5.46E-06 | 2.09E-204 | 3.14E-204 | rs3130612 | 8.23E-245 |  |
| PSC | *HSPA1L* | 43 | 7.63E-48 | 2.62E-133 | 9.74E-09 | 3.01E-204 | 4.51E-204 | rs3130612 | 8.23E-245 |  |
| PSC | *IL15RA* | 385 | 9.84E-01 | 1.00E+00 | 1.00E+00 | 1.41E-02 | 2.11E-02 | rs4147359 | 4.06E-13 |  |
| PSC | *IL2RA* | 385 | 7.44E-01 | 2.31E-04 | 1.00E+00 | 2.02E-02 | 3.03E-02 | rs4147359 | 4.06E-13 |  |
| PSC | *IMPDH2* | 143 | 6.23E-02 | 1.00E+00 | 2.81E-03 | 1.00E+00 | 1.00E+00 | rs13062429 | 3.27E-17 |  |
| PSC | *IP6K1* | 143 | 8.90E-30 | 8.50E-07 | 9.04E-02 | 7.52E-11 | 1.13E-10 | rs9858213 | 2.43E-20 |  |
| PSC | *IP6K2* | 103 | 2.13E-04 | 1.00E+00 | 1.68E-02 | 1.00E+00 | 1.00E+00 | rs7621347 | 5.12E-08 | √ |
| PSC | *ITPR3* | 98 | 9.93E-01 | 1.00E+00 | 1.00E+00 | 2.27E-11 | 3.41E-11 | rs73408005 | 1.29E-39 |  |
| PSC | *KDM4A* | 98 | 7.78E-01 | 2.45E-07 | 1.00E+00 | 1.00E+00 | 1.00E+00 | rs80204008 | 1.34E-03 | √ |
| PSC | *KIAA1109* | 174 | 1.72E-01 | 8.82E-17 | 1.00E+00 | 1.00E+00 | 1.00E+00 | rs13119723 | 2.22E-10 |  |
| PSC | *KIAA1530* | 18 | 8.06E-02 | 3.77E-31 | 1.00E+00 | 1.00E+00 | 1.00E+00 | rs111631632 | 9.63E-04 | √ |
| PSC | *KIAA1841* | 282 | 4.13E-01 | 1.70E-06 | 1.00E+00 | 1.00E+00 | 1.00E+00 | rs2901182 | 5.70E-06 | √ |
| PSC | *KIF11* | 282 | 7.87E-01 | 2.99E-06 | 1.00E+00 | 1.00E+00 | 1.00E+00 | rs10882105 | 1.18E-04 | √ |
| PSC | *KIFC1* | 14 | 3.72E-01 | 1.00E+00 | 1.00E+00 | 3.49E-18 | 5.24E-18 | rs56098412 | 9.98E-84 |  |
| PSC | *KLHDC8B* | 571 | 2.33E-01 | 1.00E+00 | 1.55E-03 | 1.00E+00 | 1.00E+00 | rs34762726 | 2.59E-20 |  |
| PSC | *KRR1* | 571 | 7.59E-01 | 6.70E-16 | 1.00E+00 | 1.00E+00 | 1.00E+00 | rs7966763 | 1.21E-04 | √ |
| PSC | *LAMB2* | 571 | 2.08E-01 | 1.00E+00 | 1.91E-03 | 1.00E+00 | 1.00E+00 | rs1873625 | 2.82E-19 |  |
| PSC | *LEMD2* | 30 | 1.09E-01 | 1.00E+00 | 1.00E+00 | 3.93E-09 | 5.90E-09 | rs35283694 | 4.20E-30 |  |
| PSC | *LINC00240* | 108 | 1.84E-12 | 2.53E-11 | 1.35E-14 | 1.52E-69 | 2.28E-69 | rs34196306 | 6.12E-87 |  |
| PSC | *LINC00481* | 57 | 3.21E-01 | 3.14E-10 | 5.71E-08 | 4.68E-212 | 7.02E-212 | rs3130612 | 8.23E-245 |  |
| PSC | *LRRC16A* | 57 | 7.84E-04 | 1.36E-03 | 1.00E+00 | 4.03E-50 | 6.05E-50 | rs17526722 | 1.29E-64 |  |
| PSC | *LSM2* | 57 | 5.19E-41 | 6.78E-110 | 7.39E-07 | 1.47E-204 | 2.21E-204 | rs3130612 | 8.23E-245 |  |
| PSC | *LST1* | 168 | 2.33E-07 | 0.00E+00 | 5.80E-157 | 1.84E-226 | 2.76E-226 | rs3130612 | 8.23E-245 |  |
| PSC | *LTA* | 168 | 6.85E-08 | 0.00E+00 | 8.69E-28 | 8.51E-227 | 1.28E-226 | rs3130612 | 8.23E-245 |  |
| PSC | *LTB* | 168 | 6.76E-07 | 0.00E+00 | 2.26E-55 | 4.76E-226 | 7.14E-226 | rs3130612 | 8.23E-245 |  |
| PSC | *LY6G5B* | 130 | 3.47E-05 | 8.81E-159 | 2.58E-162 | 2.74E-225 | 4.11E-225 | rs3130612 | 8.23E-245 |  |
| PSC | *LY6G5C* | 130 | 2.12E-04 | 4.51E-225 | 6.97E-158 | 2.34E-225 | 3.51E-225 | rs3130612 | 8.23E-245 |  |
| PSC | *MARVELD3* | 469 | 2.20E-01 | 3.34E-17 | 1.00E+00 | 1.00E+00 | 1.00E+00 | rs12597418 | 1.55E-03 | √ |
| PSC | *MICA* | 469 | 3.98E-12 | 0.00E+00 | 5.91E-196 | 5.44E-235 | 8.16E-235 | rs3130612 | 8.23E-245 |  |
| PSC | *MICB* | 474 | 8.43E-03 | 0.00E+00 | 9.12E-97 | 1.79E-226 | 2.68E-226 | rs3130612 | 8.23E-245 |  |
| PSC | *MMEL1* | 474 | 3.23E-06 | 1.00E+00 | 1.00E+00 | 1.00E+00 | 1.00E+00 | rs6657596 | 2.70E-08 |  |
| PSC | *MNF1* | 474 | 5.28E-01 | 3.84E-04 | 1.00E+00 | 8.02E-09 | 1.20E-08 | rs34434446 | 1.24E-38 |  |
| PSC | *MON1A* | 474 | 2.44E-04 | 5.91E-05 | 1.00E+00 | 1.00E+00 | 1.00E+00 | rs9858213 | 2.43E-20 |  |
| PSC | *MRPS17* | 464 | 3.88E-01 | 1.65E-09 | 1.00E+00 | 1.00E+00 | 1.00E+00 | rs816416 | 5.95E-05 | √ |
| PSC | *MSH5* | 250 | 2.72E-63 | 9.74E-72 | 8.90E-05 | 2.43E-204 | 3.64E-204 | rs3130612 | 8.23E-245 |  |
| PSC | *MSH5-SAPCD1* | 250 | 2.16E-58 | 5.07E-55 | 1.64E-03 | 2.82E-204 | 4.22E-204 | rs3130612 | 8.23E-245 |  |
| PSC | *MST1* | 250 | 4.40E-32 | 9.59E-11 | 2.16E-02 | 7.38E-11 | 1.11E-10 | rs9858213 | 2.43E-20 |  |
| PSC | *MST1R* | 250 | 9.96E-08 | 1.00E+00 | 1.00E+00 | 1.00E+00 | 1.00E+00 | rs9858213 | 2.43E-20 |  |
| PSC | *MTERFD1* | 250 | 8.92E-01 | 2.51E-03 | 1.00E+00 | 1.00E+00 | 1.00E+00 | rs920200 | 1.03E-03 | √ |
| PSC | *MTMR4* | 250 | 2.26E-01 | 1.83E-07 | 1.00E+00 | 1.00E+00 | 1.00E+00 | rs8067912 | 3.78E-05 | √ |
| PSC | *MUC22* | 250 | 7.26E-04 | 0.00E+00 | 2.19E-36 | 4.33E-212 | 6.50E-212 | rs3130612 | 8.23E-245 |  |
| PSC | *NCKIPSD* | 250 | 7.01E-04 | 1.00E+00 | 5.86E-03 | 1.00E+00 | 1.00E+00 | rs7621347 | 5.12E-08 | √ |
| PSC | *NCR3* | 53 | 4.80E-09 | 0.00E+00 | 2.84E-22 | 3.88E-226 | 5.81E-226 | rs3130612 | 8.23E-245 |  |
| PSC | *NDUFAF3* | 53 | 2.20E-01 | 1.00E+00 | 6.40E-03 | 1.00E+00 | 1.00E+00 | rs13062429 | 3.27E-17 |  |
| PSC | *NDUFS4* | 53 | 8.84E-01 | 3.34E-04 | 1.00E+00 | 1.00E+00 | 1.00E+00 | rs148306918 | 1.16E-04 | √ |
| PSC | *NEU1* | 53 | 4.12E-02 | 1.29E-71 | 4.13E-05 | 2.81E-204 | 4.22E-204 | rs3130612 | 8.23E-245 |  |
| PSC | *NFKBIL1* | 45 | 4.20E-06 | 0.00E+00 | 1.60E-35 | 4.16E-226 | 6.23E-226 | rs3130612 | 8.23E-245 |  |
| PSC | *NICN1* | 232 | 3.18E-01 | 5.14E-22 | 1.00E+00 | 3.19E-07 | 4.75E-07 | rs9858213 | 2.43E-20 |  |
| PSC | *NKAPL* | 232 | 3.21E-47 | 3.45E-34 | 1.00E+00 | 2.60E-89 | 3.90E-89 | rs1238262 | 2.37E-110 |  |
| PSC | *NMB* | 232 | 9.67E-01 | 1.97E-03 | 1.00E+00 | 1.00E+00 | 1.00E+00 | rs73437980 | 8.53E-05 | √ |
| PSC | *NOTCH4* | 232 | 2.48E-01 | 1.68E-101 | 6.81E-25 | 5.51E-195 | 8.26E-195 | rs4713534 | 6.58E-232 |  |
| PSC | *OR2B6* | 232 | 1.55E-54 | 4.49E-76 | 1.00E+00 | 4.18E-86 | 6.27E-86 | rs35017208 | 3.88E-101 |  |
| PSC | *P4HTM* | 72 | 5.68E-02 | 1.00E+00 | 5.18E-04 | 1.00E+00 | 1.00E+00 | rs7630869 | 3.38E-17 |  |
| PSC | *PANK4* | 72 | 2.72E-06 | 1.00E+00 | 1.00E+00 | 1.00E+00 | 1.00E+00 | rs6657596 | 2.70E-08 |  |
| PSC | *PBX2* | 72 | 5.55E-02 | 5.95E-107 | 1.56E-20 | 3.90E-194 | 5.85E-194 | rs4713534 | 6.58E-232 |  |
| PSC | *PEX13* | 72 | 1.35E-01 | 1.65E-20 | 1.00E+00 | 1.00E+00 | 1.00E+00 | rs2901182 | 5.70E-06 | √ |
| PSC | *PFDN6* | 15 | 2.63E-04 | 1.00E+00 | 1.00E+00 | 1.39E-24 | 2.08E-24 | rs7758736 | 5.32E-166 |  |
| PSC | *PGBD1* | 15 | 1.87E-45 | 6.18E-21 | 6.05E-21 | 2.13E-90 | 3.20E-90 | rs1238262 | 2.37E-110 |  |
| PSC | *PHF1* | 15 | 3.42E-01 | 1.00E+00 | 1.00E+00 | 1.61E-17 | 2.41E-17 | rs56098412 | 9.98E-84 |  |
| PSC | *PLCH2* | 15 | 3.24E-06 | 1.00E+00 | 1.00E+00 | 1.00E+00 | 1.00E+00 | rs6657596 | 2.70E-08 |  |
| PSC | *POM121L2* | 15 | 5.35E-01 | 4.06E-03 | 1.00E+00 | 3.08E-71 | 4.62E-71 | rs13217620 | 3.58E-92 |  |
| PSC | *POU5F1* | 15 | 8.32E-05 | 3.55E-109 | 7.12E-05 | 1.60E-211 | 2.40E-211 | rs3130612 | 8.23E-245 |  |
| PSC | *PPT2* | 15 | 5.66E-02 | 1.57E-112 | 2.11E-22 | 2.64E-194 | 3.96E-194 | rs4713534 | 6.58E-232 |  |
| PSC | *PRKAR2A* | 26 | 1.08E-05 | 1.00E+00 | 8.22E-03 | 1.00E+00 | 1.00E+00 | rs35446411 | 3.23E-09 |  |
| PSC | *PRRC2A* | 26 | 9.04E-02 | 7.20E-279 | 1.47E-174 | 7.23E-226 | 1.08E-225 | rs3130612 | 8.23E-245 |  |
| PSC | *PRRT1* | 26 | 1.14E-01 | 3.91E-79 | 2.26E-06 | 6.29E-194 | 9.43E-194 | rs4713534 | 6.58E-232 |  |
[truncated: 73,441 more chars]
